# Label-free proteomic methodology for the analysis of human kidney stone matrix composition

**DOI:** 10.1186/s12953-016-0093-x

**Published:** 2016-02-27

**Authors:** Frank A. Witzmann, Andrew P. Evan, Fredric L. Coe, Elaine M. Worcester, James E. Lingeman, James C. Williams

**Affiliations:** Department of Cellular and Integrative Physiology, Indiana University School of Medicine, 635 Barnhill Drive, Room 362A, Indianapolis, IN 46202-5120 USA; Department of Anatomy and Cell Biology, Indiana University School of Medicine, Indianapolis, IN USA; Department of Medicine, Nephrology Section, University of Chicago, Chicago, IL USA; International Kidney Stone Institute, Methodist Hospital, Indianapolis, IN USA

**Keywords:** Calcium oxalate, Kidney stone, Label-free quantitative liquid chromatography–tandem mass spectrometry, Matrix protein, Nephrolithiasis, Proteomics

## Abstract

**Background:**

Kidney stone matrix protein composition is an important yet poorly understood aspect of nephrolithiasis. We hypothesized that this proteome is considerably more complex than previous reports have indicated and that comprehensive proteomic profiling of the kidney stone matrix may demonstrate relevant constitutive differences between stones. We have analyzed the matrices of two unique human calcium oxalate stones (CaOx-Ia and CaOx-Id) using a simple but effective chaotropic reducing solution for extraction/solubilization combined with label-free quantitative mass spectrometry to generate a comprehensive profile of their proteomes, including physicochemical and bioinformatic analysis.`

**Results:**

We identified and quantified 1,059 unique protein database entries in the two human kidney stone samples, revealing a more complex proteome than previously reported. Protein composition reflects a common range of proteins related to immune response, inflammation, injury, and tissue repair, along with a more diverse set of proteins unique to each stone.

**Conclusion:**

The use of a simple chaotropic reducing solution and moderate sonication for extraction and solubilization of kidney stone powders combined with label-free quantitative mass spectrometry has yielded the most comprehensive list to date of the proteins that constitute the human kidney stone proteome.

**Electronic supplementary material:**

The online version of this article (doi:10.1186/s12953-016-0093-x) contains supplementary material, which is available to authorized users.

## Background

The organic matrix within urinary stones has long been thought to be an important—if poorly understood—part of stone composition. It has been proposed that the process of stone formation involves the primary deposition of matrix, with crystal formation occurring secondarily within the matrix layer [1, 2]. Others maintain that crystallization is primary and that most, if not all, of the organics in stones are co-precipitated with the crystals in a manner that is in no way causative [3]. Recent work on the interface of the growth of calcium oxalate (CaOx) stones on Randall’s plaque has suggested that matrix deposition is the primary event, at least in the formation of CaOx stones over plaque. The first layer covering plaque that has been exposed to urine is an organic layer that contains Tamm Horsfall protein, aka uromodulin [4]. Thereafter, nucleation of apatite begins and deposition of CaOx follows [4].

Progress on characterizing the composition of stone matrix has been slow, in part because of its insolubility. The work that has been done also has demonstrated that the matrix composition is remarkably variable and complex [5]. However, recent studies of human stone matrix have begun to exploit the power of modern proteomic methods, with some patterns beginning to emerge in the kinds of proteins found [6–17]. Nine of these recent stone proteomic studies contain profiles of CaOx stone- or crystal-associated proteins [6, 7, 9, 11–15, 17] but only one study has attempted to quantify them [17].

Thus far, the number of proteins identified in human stone matrices has been relatively modest, ranging from 30 [11] to 242 [10] distinct proteins identified across a variety of stone mineral types where the proteome has been investigated. In general, where protein profiles of different types of stones were compared, differences across stone types were minimal whereas protein differences within stone types were quite variable. This is likely due to two factors: 1) only the most abundant, common proteins were identified by these studies and 2) individual kidney stone proteomes are by nature variable and diverse.

We hypothesized that the analysis of human stone matrix should yield significantly more proteins than previously detected and that an improved approach may be useful in studying nephrolithiasis. Using a simple but effective chaotropic reducing solution for extraction and solubilization, one that historically works well for a broad range of protein samples [18–20], combined with gentle, intermittent sonication, we have analyzed extracts of two unique human CaOx stones, CaOx-Ia and CaOx-Id, (of different morphologies of CaOx monohydrate, according to the morphoconstitutional classification scheme published by Daudon [21]), using an established label-free quantitative mass spectrometric (LFQMS) method. This method described in detail elsewhere [22, 23] uses individual three-dimensional alignment to determine peptide retention time using a clustering method along with peptide ion peak areas calculated from the extracted ion chromatogram (XIC) generated by liquid chromatography - tandem mass spectrometry (LC-MS/MS). Only two stone matrices were analyzed in this study, by design. Our intent was to 1) improve and simplify the extraction of protein from the stone powder and 2) apply a novel, more comprehensive LFQMS approach to identify and quantify as many proteins as possible. The results indicate a stone matrix proteome that is much larger and more complex than previously observed.

## Results and discussion

### Stone extracts

**Fig. 1:**
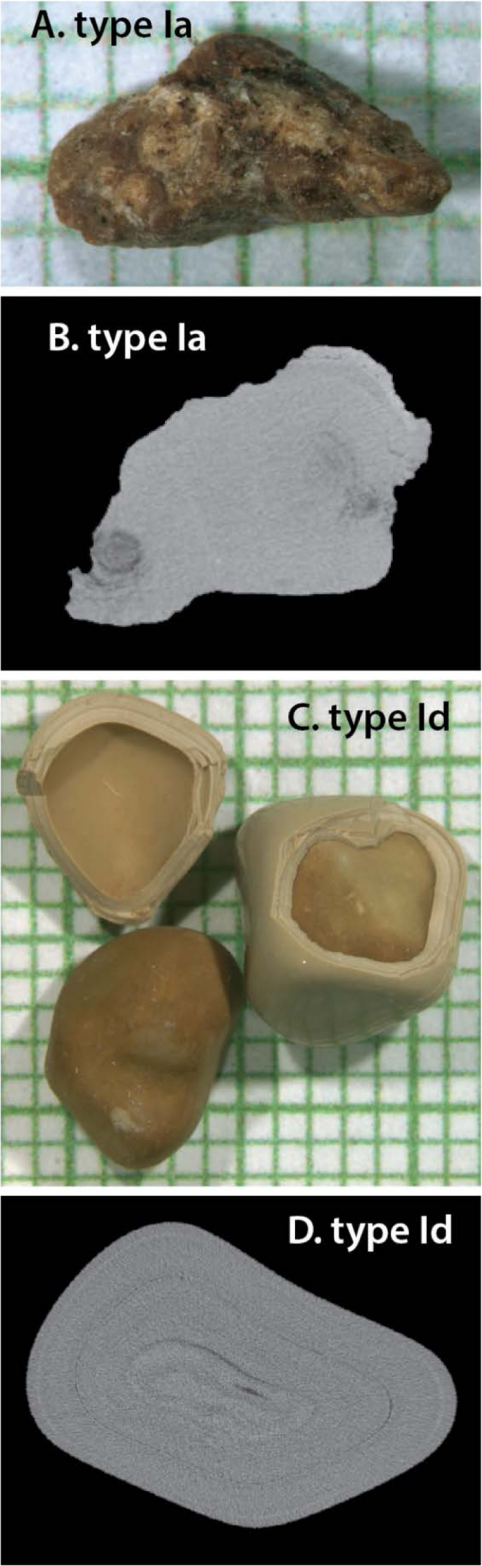
Representative pieces from the two stone specimens used. **a** Fragment from CaOx-Ia, on mm-grid paper. **b** Micro CT slice through fragment in A, showing pure calcium oxalate monohydrate (COM). **c** Two stones from specimen CaOx-Id; stone on right has shell broken off to reveal interior core, and on left is shell (top) and core from another stone in this specimen. For CaOx-Id, only the shell portions were collected for protein extraction. **d** Micro CT slice through CaOx-Id stone, showing pure COM

It is not exactly clear what fraction of the total stone protein can be extracted in a form usable for proteomic analysis. Our extraction method yielded 7.20 μg protein/mg stone powder for CaOx-Ia and 0.81 μg/mg for CaOx-Id. The larger value is 3 times the average extraction yield that we have previously reported for calcium oxalate monohydrate (COM) stones using 9 M urea/1 mM DTT [5]. The increased amount of protein in CaOx-Ia was likely due to the presence of the x-ray lucent material identified by micro CT. Note that most researchers, not using micro CT, would likely be unable to detect the presence of such a non-mineral-rich material, which, in this case, clung tightly to the surface of stone fragments. On the other hand, protein extraction from specimen CaOx-Id yielded a value within the range we found previously for different COM stone specimens [5], though on the low end of that range. The low yield of protein in this specimen might be related to the tightly packed nature of this form of COM stone (Fig. 1d).

The total amount of protein in these types of stones, estimated from complete acid hydrolysis and measurement of amino acids, has been reported to be about 17 μg/mg [2], but of course, such an acid hydrolysis destroys protein identities. It is likely that a considerable amount of stone protein is resistant to extraction by solubilization methods that maintain protein primary structure, as has been previously reported [5]. In comparison, of the previous studies where proteomic analysis was used and extracted protein yields reported [7, 9, 17], the average was 1.5 μg/mg, probably less than 10 % of the total protein contained within the stones.

Whether the extraction resistant proteins are 1) very low abundance components of the matrix proteome trapped within the crystal matrix [15, 16] and thus not detected by conventional means, 2) chemically more hydrophilic and accordingly more avidly bound to crystal surfaces, or 3) merely unextracted replicates of the proteins identified below remains to be determined.

### Label-free quantitative mass spectrometry

**Fig. 2:**
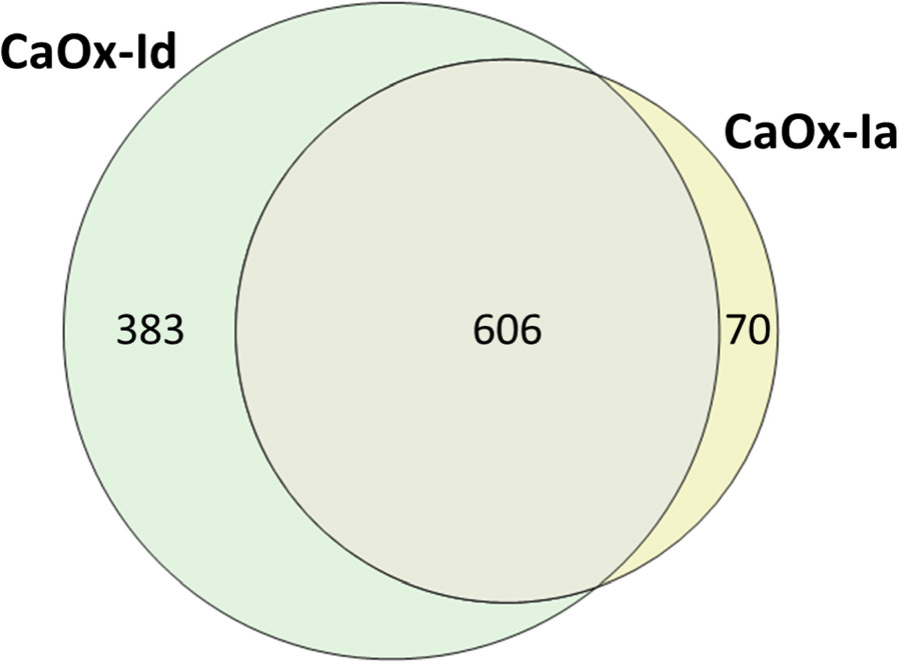
Venn diagrams showing the degree of overlap and exclusivity of proteins identified and quantified in CaOx-Ia and CaOx-Id kidney stone powders

Using a label-free quantitative mass spectrometry platform, we identified and quantified 1,059 unique protein database (UniProt, http://www.uniprot.org) entries including splice variants or isoforms (809 unique gene names), in the two human kidney stone samples with a false discovery rate (FDR) of ≤0.2 %. These proteins are listed in a table in Additional file 1, along with their UniProt identities, gene symbols, protein names, and abundances [22]. As illustrated in Fig. 2, 606 proteins were common to both stone types; 70 proteins were unique to CaOx-Ia, while 383 proteins were detected only in the CaOx-Id stone powder. Specific peptide information for all identified proteins and protein groups including protein coverage, # of unique sequences, # of identified peptides, total # of identified sequences, is available in the table found in Additional file 2.

To account for potential bacterial proteins within the stone matrix, the MS data were searched against *Corynebacterium, Actinomyces, Lactobacillus jensenii, Streptococcus anginosus*, and *Staphylococcus epidermidis* protein databases. No proteins were identified. This strongly suggests that there is no significant bacterial contribution to the stone matrix proteome in the specimens tested.

Since 2008, twelve papers have been published that described various analyses of human kidney/bladder stone matrix proteomes or crystal-associated proteins [6–17]. Compared to those earlier investigations, we found the kidney stone matrix proteome to be larger and much more complex than observed previously. We identified and quantified between 4–35 times as many proteins as found in the other human studies. More specifically, we found 259 proteins that were identified in earlier studies (see the Human Kidney Stone Matrix Proteome Database presented in Additional file 3), and also an additional 577 proteins not identified previously. This database also contains the following information for each protein: isoelectric point (pI), # of negatively charged residues (Asp + Glu), # of positively charged residues (Arg + Lys), Neg/Pos Ratio, aliphatic index, GRAVY score (grand average of hydropathicity), molecular class, biological process, cellular component, and function.

The increased protein identification rate that we achieved in this investigation may be due to two factors. First, several previous studies used a demineralization approach to extract proteins from powdered stones that included EDTA and/or SDS [7–9, 11, 13–17], both of which were subsequently removed by either centrifugation or dialysis steps. Boonla et al. [6] used a commercially available lithium dodecyl sulfate/glycerol solution at 100 °C to extract proteins. Our use of 8 M urea/10 mM DTT with sonication and two repeated overnight extractions of the stone powder required no additional purification steps where proteins might be lost. As mentioned earlier, this approach yielded, on average, more protein/mg of stone powder than in previous studies. But that alone does not account for the differences in the number of proteins detected. A second reason may lie in the protein analysis itself. Several previous studies used 1D and/or 2D gel electrophoresis and subsequent LC-MS/MS or MALDI-MS/MS to identify proteins in gel plugs [6, 11, 13, 14, 16, 17]. Others used LC-MS/MS or MALDI-MS/MS of whole extracts [7–10, 14–17], but used LC gradient profiles of 60 min or less to separate the tryptic peptides. Our use of a 190 min LC gradient combined with the rapid scanning features of the Orbitrap Velos Pro mass spectrometer likely underlie our increased protein identification rate. It should be noted that in a proteomic analysis of rat urinary melamine stone matrix (where urea, thiourea, detergent and DTT were used to extract proteins, a 90 min LC gradient and a high resolution Orbitrap mass spectrometer were used to separate and analyze tryptic peptides), over 1,000 proteins were identified [24].

Interestingly, there are 204 proteins listed in the database in Additional file 3 that were identified in the previous twelve studies, but were not detected in our stone samples. Nearly half of these are accounted for by 96 proteins (of 242) identified by Jou et al. in uric acid stones [10]. The other 146 uric acid stone proteins they detected were also identified in our CaOx stones. This substantial dissimilarity likely is due to the physicochemical differences between these various stone types and the extraction methods and analytical approaches used. Additionally, Merchant et al. analyzed the proteome of CaOx stones obtained from human subjects and identified 158 proteins [14]. Forty-five of these were not detected in our CaOx stones, suggesting that even within similar stone types, the protein composition may be patient-specific and thus differ significantly.

A brief, non-statistical comparison of CaOx-Ia and CaOx-Id stone matrix protein composition is presented below. At this point, it is important to restate that only two stone matrices were analyzed in this study, by design, and that our intent was to 1) improve and simplify the extraction of protein from the stone powder and 2) apply a novel, more comprehensive LFQMS approach to identify and quantify as many proteins as possible. The following data analysis is by no means intended to imply the existence of significant differences between the unique CaOx stones. It is presented here to compare and contrast the results of our new quantitative approach to previous stone matrix proteome studies and not to make inferences on the pathophysiology of CaOx stone formation.

The 50 most abundant proteins from each stone are listed in Table 1. These correspond to many of the proteins reported in previous studies, in particular: serum albumin, apolipoproteins, calgranulins, osteopontins (10-12-fold higher in CaOx-Id), prothrombin, alpha and beta hemoglobin, neutrophil defensin I, complement proteins, and alpha-1-antitrypsin. In contrast, proteins such as fatty acid synthase and numerous cytokeratins were rarely observed in previous studies. Their absence from previous studies may be accounted for by the fact that these proteins were not disclosed as their occurrence is generally thought to be due to contamination from skin and ambient environment. This may be true of the epidermal and cuticular keratins identified in this study (KRT2, KRT10, KRT31, KRT77, KRT81 KRT84, KRT85), while others are well-established components of epithelial cells throughout the kidney [25–27] (KRT1, KRT3, KRT5, KRT6, KRT7, KRT8, KRT9, KRT13, KRT14, KRT17, KRT18, KRT19, KRT31, KRT80).

**Table 1:** Fifty most-abundant proteins in kidney stones CaOx-Ia and CaOx-Id

CaOx-Ia	CaOx-Id
Hemoglobin subunit beta	Prothrombin
Neutrophil defensin 1 (Defensin, Alpha 1)	Hemoglobin subunit beta
Hemoglobin subunit alpha	Hemoglobin subunit alpha
Protein S100-A9 (Calgranulin-B)	Thrombin light chain
Complement C3	Vitronectin
Hemoglobin subunit delta	Complement C3
Protein S100-A8 (Calgranulin-A)	Vitamin K-dependent protein Z
Alpha-1-antitrypsin	Plasminogen
Keratin, type I cytoskeletal 10	Keratin, type I cytoskeletal 10
Prothrombin	Serum albumin
Fibrinogen beta chain	Hemoglobin subunit delta
ATP-dependent RNA helicase A	C4b-binding protein alpha chain
Apolipoprotein A-IV	Alpha-1-antitrypsin
Keratin, type I cytoskeletal 13	Keratin, type II cytoskeletal 1
Serum albumin	Fatty acid synthase
Keratin, type II cytoskeletal 1	Apolipoprotein A-I
Keratin, type I cytoskeletal 9	Mannan-binding lectin serine protease 2
Fatty acid synthase	Osteopontin, Isoform 5
Nucleolin	Myosin-9
Apolipoprotein B-100	Heat shock protein beta-1
Heparin cofactor 2	Fibroleukin (fibrinogen-like 2)
Actin, cytoplasmic 1	Apolipoprotein D
Fibrinogen alpha chain	Histidine-rich glycoprotein
Plasminogen	Tubulin beta chain
Profilin-1	Fibrinogen beta chain
Eosinophil cationic protein	Kininogen-1, Isoform LMW
Coagulation factor XII	Keratin, type I cytoskeletal 9
Complement C4-A	Heat shock protein HSP 90-beta
Complement C4-B	Apolipoprotein B-100
Vitronectin	Pro-epidermal growth factor
Myosin-9	Fibrinogen alpha chain
Apolipoprotein A-I	Neutrophil defensin 1 (Defensin, Alpha 1)
Leukocyte elastase inhibitor	Extracellular superoxide dismutase [Cu-Zn]
Alpha-2-macroglobulin	Argininosuccinate synthase
Apolipoprotein D	Heparin cofactor 2
Thrombin light chain	Complement factor B
Complement factor B	Keratin, type I cytoskeletal 19
Histone H4	Apolipoprotein A-IV
Vesicular integral-membrane protein VIP36	Complement C4-A
Fibrinogen gamma chain	Coagulation factor X
Serum paraoxonase/arylesterase 1	Complement C4-B
Peroxiredoxin-2	Vesicular integral-membrane protein VIP36
Complement C5	Actin, cytoplasmic 1
Complement component C9	Profilin-1
WD repeat-containing protein 1	Nucleolin
Antithrombin-III	Keratin, type II cytoskeletal 7
Mannan-binding lectin serine protease 2	Protein S100-A9 (Calgranulin-B)
Mannosyl-oligosaccharide 1,2-alpha-mannosidase IA	Osteopontin, Isoform B
Vitamin K-dependent protein S	Mannosyl-oligosaccharide 1,2-alpha-mannosidase IA
Keratin, type II cytoskeletal 2	Antithrombin-III

*Proteins listed in order of abundance*

When one examines the database in Additional file 3, of the 257 proteins reported in other papers that also were found in our study, albumin, uromodulin (Tamm-Horsfall Protein, THP), calgranulin-A (Protein S100-A8), and calgranulin-B (Protein S100-A9) were common to all studies. Albumin and THP were similarly abundant in both our CaOx stones while calgranulin-A was nearly ten-fold higher in CaOx-Ia. Lactotransferrin, osteopontin, and prothrombin were detected in ten previous studies and Vitamin K-dependent protein Z was detected in nine. Twenty-two immunoglobulin-related proteins and 28 complement-related proteins were identified and quantified, and most were common to both of our CaOx specimens.

Note that many of the proteins listed are undoubtedly from blood or tissue, but this is also true for proteins found in human urine [28]. Some work that has been done on crystallization of calcium oxalate crystals *in vitro* in human urine has suggested that proteins of blood and tissue are not adsorbed to forming crystals, with the idea that proteins of the blood or tissue are not relevant to stone formation [29]. But simple crystal formation is not the same as the formation of a stone, in which the protein matrix plays an important role [30]. Moreover, injury to the renal papilla may be a normal part of stone formation [31], so the presence of blood and tissue proteins could well be a part of the formation of a true stone.

### Physicochemical analysis

Previous studies have considered the aggregate protein charge and its potential connection to specific stone types [7, 10]. In a comparison of 4 different stone types, Canales et al. [7] failed to observe statistically significant differences between the number of acidic- versus basic-fractionated matrix proteins. In our study (Table 2), the average pI of all proteins associated with the CaOx-Ia stone or the CaOx-Id stone was similar (6.62 vs. 6.49), as was the average pI of all proteins unique to CaOx-Ia or CaOx-Id stones (6.75 vs. 6.33). In comparison, a slightly more alkaline average pI of 7.2 has been observed in uric acid stone matrix proteins [10]. Conclusive comparison of these and other properties requires additional samples and further study. Nevertheless, negative amino acid/positive amino acid ratios, aliphatic indices (a measure of protein stability) and GRAVY scores were similar across all comparisons in the CaOx stones. The mean GRAVY scores, all considerably negative, indicate overall protein hydrophilicity.

**Table 2:** Physicochemical characteristics of stone matrix proteomes

	Isoelectric Point	Neg/Pos Ratio	Aliphatic Index	GRAVY Score
CaOx-Ia All Proteins	6.62 ± 1.65	1.16 ± 0.66	79.07 ± 11.17	−0.416 ± 0.229
CaOx-Id All Proteins	6.49 ± 1.65	1.19 ± 0.60	78.92 ± 11.42	−0.438 ± 0.240
CaOx-Ia Only	6.75 ± 1.71	1.08 ± 0.39	80.14 ± 12.26	−0.340 ± 0.171
CaOx-Id Only	6.33 ± 1.65	1.20 ± 0.39	78.97 ± 15.16	−0.458 ± 0.348

### Bioinformatic analysis

Of the proteins listed in the Human Kidney Stone Matrix Proteome Database (Additional file 3), 680 (82 %) have been detected in kidney cells or tissue (per the Human Proteome Map), 154 (18 %) have not been detected in kidney, so their origin may be considered “extra-renal”, 144 (17 %) are considered to be moderately or highly abundant in kidney, 248 (30 %) are considered to be in the “extracellular component” (kidney or otherwise), and 75 (9 %) are considered to be “cytoskeletal and/or structural” proteins.

Proteins identified in the CaOx stones also represented a broad variety of molecular classes (Table 3). The distribution of classes reflects the prevalence of “cellular proteins” in the matrix, dominated by cytoskeletal & associated proteins, structural proteins, transport/cargo proteins, chaperone/heat shock proteins, and ribonucleoprotein/RNA binding proteins, rather than an excess of urine- or plasma-related proteins. Many of these proteins may stem from the considerable cellular components of stone matrices first observed by Boyce in 1956 [32]. Nevertheless, as Table 1 and Additional file 1 indicate, the individually most abundant proteins in the CaOx stone matrix are blood/plasma derived – and presumably urine – proteins.

**Table 3:** Representation of CaOx stone proteins by molecular class (≥5)

Protein Molecular Class	# of unique proteins identified
Cytoskeletal & associated protein; structural protein	75
Transport/cargo protein	49
Chaperone/Heat shock protein	36
Ribonucleoprotein; RNA binding protein	35
Extracellular matrix protein	31
Transcription factor or regulatory protein	29
Enzyme: Hydrolase	27
Translation regulatory protein	27
G protein; GTPase & associated	26
Immunoglobulin	24
Unclassified	24
Ribosomal subunit	23
Adhesion molecule	21
Enzyme: dehydrogenase	21
Ubiquitin proteasome system	21
Complement protein	20
Protease inhibitor	19
Adapter molecule	18
Calcium binding protein	18
Secreted polypeptide	18
Coagulation factor	16
Cysteine protease	15
DNA binding protein	15
Integral membrane protein	15
Serine/threonine kinase or phosphatase	15
Enzyme: Ligase	14
Enzyme: Oxidoreductase	14
Cell surface receptor	12
Enzyme: Phosphotransferase	12
Serine protease	9
Enzyme: Isomerase	7
Enzyme: Peroxidase	6
Membrane transport protein	6
MHC complex protein	6
ATPase	5
Enzyme: Reductase	5

The following calcium binding proteins were detected: annexins A1, A2, A3, A4, A5, A9, A10, and A11; calcyphosin; calmodulin; calsequestrin-2; cilaggrin-2; cucleobindin-1; osteopontin; Profilaggrin; Protein S100-A2 (S100 calcium-binding protein A2); Protein S100-A6 (Calcyclin); Protein S100-A7 (Psoriasin); Protein S100-A8 (Calgranulin-A); Protein S100-A9 (Calgranulin-B); Protein S100-P (Migration-inducing gene 9 protein); Protein S100-A11 (Calgizzarin); and Protein S100-A12 (Calgranulin-C), and these may have implications in the mineralization process [33, 34]. Additionally, the following urinary proteins known to have the potential to modulate crystal formation and retention [35–37] were identified and quantified as prominent constituents of the stone matrix: Tamm-Horsfall protein; osteopontin; Α-1 microglobulin; calprotectin (protein S100-A8 & 9); serum albumin; prothrombin; inter-α trypsin inhibitor (heavy chains H1, H2, and 4); heparin sulphate proteoglycan; bikunin; CD44, fetuin, and various collagens.

As in two previous studies [10, 14] where inflammatory, coagulation, cell adhesion, and acute-phase response pathways were directly related to high abundance matrix proteins, we used pathway analysis to predict with statistical confidence which pathways might be associated with the matrix proteins identified and quantified in our CaOx stones. These results (79 unique pathways) are presented in the Pathway Data found in Additional file 4. Some of the most statistically significant pathways included LXR/RXR activation, coagulation system, acute phase response signaling, FXR/RXR activation, clathrin-mediated endocytosis signaling, intrinsic prothrombin activation pathway, epithelial adherens junction signaling/remodeling, extrinsic prothrombin activation, complement system, and the production of nitric oxide and reactive oxygen species in macrophages. Analogous observations were made via functional annotation clustering of the CaOx proteins presented in the Functional Annotation Clusters found in Additional file 5, where inflammatory response and immune related functions were most notable and cytoskeletal structural molecule activity, extracellular glycoprotein signaling, wound healing, coagulation, and regulation of body fluid levels annotations were significantly represented.

## Conclusions

The proteomic data presented here corroborate and significantly expand previous observations of the kidney stone matrix protein composition, revealing a more complex matrix proteome than previously reported for human kidney stones of any type. It remains unclear as to whether the identified proteins, their physicochemical properties, and their associated pathways/functions are directly related to mechanisms of stone formation or simply coincident and accumulated through long-term exposure of the growing stone to urine flow. Nonetheless, the comprehensive approach we have developed and reported here will enable us and others to address such questions by analyzing and comparing comprehensive protein profiles in a broad range of stone types in relatively small stone specimens from individual patients who are carefully stratified by phenotype, and for whom important clinical data are known.

## Methods

### Reagents

Urea, DL-Dithiothreitol (DTT), triethylphosphine (TEP), iodoethanol, and ammonium bicarbonate (NH_4_HCO_3_) were purchased from Sigma-Aldrich (St. Louis, MO, USA). LC-MS grade acetonitrile (ACN) with 0.1 % formic acid (v/v) and water (H_2_O) with 0.1 % formic acid (v/v) were purchased from Burdick & Jackson (Muskegon, MI, USA). Modified sequencing grade porcine trypsin was obtained from Princeton Separations (Freehold, NJ, USA). All other reagents used were of the highest quality available.

### Stone specimens, preparation of stone matrix protein extracts, and protein assay

Stones were de-identified specimens analyzed as pure COM and obtained from a stone analysis laboratory (Beck Analytical Services, Indianapolis IN). They were chosen so that several grams of material were available within a single specimen. The specimens were scanned using micro CT (SkyScan 1172, Bruker, Kontich, Belgium) to assess overall mineral purity, and mineral composition was confirmed using Fourier-transform infrared spectroscopy [38].

Figure 1 shows representatives of the two specimens used in the present study. The specimen named CaOx-Ia consisted of multiple fragments of COM that mostly had the morphological form Ia, which indicates a tightly packed COM form that typically is dark brown in color [21]. The CaOx-Ia specimen also contained some x-ray lucent material, identified by infrared spectroscopy to be non-mineralized protein. Specimen CaOx-Id was also pure COM, but consisted of a dozen smooth stones of the morphological form Id, a distinctly different but still tightly packed form of COM [21]. Note that CaOx-Id, due to its morphology, contained no extraneous protein, as did CaOx-Ia. It may also be noteworthy that neither of these CaOx specimens contained any apatite that was visible by micro CT (a very sensitive method for detecting this mineral [38]). Thus, these CaOx specimens were not representative of the kinds of stones that would arise from growth on Randall's plaque [4].

CaOx-Ia and CaOx-Id stones were powdered by hand in small portions using an agate mortar and pestle, grinding the powder to the consistency of fine flour. The portions of powder for each specimen were combined and thoroughly mixed. Aliquots of these stone powders (300 mg) were combined with 1 mL of freshly prepared 8 M urea and 10 mM DTT by vortex mixing (15 s) in Fisherbrand™ skirted microcentrifuge tubes with threaded ends (Catalog No. 02-681-343). The suspension was sonicated (Microson™, Misonix, USA) by 10 intermittent 1 s microprobe pulses at an energy output of 15 W, every 30 min for 4 h at room temperature. The tubes were incubated at room temperature overnight on an orbital shaker (200 rpm), vortexed, and subjected to one more sonication step as described above. The suspension was centrifuged at 3,200 x g for 1 h at room temperature (Jouan GR4i centrifuge, ThermoFisher Scientific, USA). The supernatant was collected and the pellet subjected to a second protein extraction carried out exactly as the first. Extracts were combined and protein concentration in each pooled extract (~2 mL) was determined by the Bradford assay [39]. The solubilized protein extracts were then stored at −80 °C until LFQMS analysis.

### Proteolysis and LC-MS/MS

A 100 μg aliquot of each sample was concentrated using the Vivaspin® 500 Centrifugal Concentrator (Vivaproducts, USA) by centrifugation at 8,000 rpm. The volume of each sample was adjusted to 200 μL (4 M urea) and then reduced and alkylated by triethylphosphine and iodoethanol as previously described [40]. Briefly, 200 μL of the reduction/alkylation cocktail was added to the protein solution. The sample was incubated at 35 °C for 60 min, dried by a Vacuum Concentrator Centrifugal System (RC 10.10, Jouan), and reconstituted with 100 μL of 100 mM NH_4_HCO_3_ at pH 8.0. A 150 μL aliquot of a 20 μg/mL trypsin solution was added to the sample and incubated at 35 °C for 3 h, after which another 150 μL of trypsin was added, and the solution incubated at 35 °C for an additional 3 h.

Sample digests were analyzed using a ThermoScientific Orbitrap Velos Pro hybrid ion trap-Orbitrap mass spectrometer coupled with a Surveyor autosampler and MS HPLC system (ThermoScientific). Tryptic peptides were injected as technical replicates onto a C18 reversed phase column (TSKgel ODS-100 V, 3 μm, 1.0 mm × 150 mm) at a flow rate of 50 μL/min. The mobile phases A, B, and C were 0.1 % formic acid in water, 50 % ACN with 0.1 % formic acid in water, and 80 % ACN with 0.1 % formic acid in water, respectively. The gradient elution profile was as follows: 10 % B (90 % A) for 7 min, 10–67.1 % B (90–32.9 % A) for 163 min, 67.1-100 % B (32.9-0 % A) for 10 min, and 100-50 % B (0-50 % C) for 10 min. It is important to note that the 190 min elution gradient used in this analysis is much longer than conventional 60 min elution profiles used in previous kidney stone proteome studies [7–10, 14–17] where LC-MS/MS was used, and likely accounts for much of the significant improvement in proteome coverage observed here (see Results and Discussion).

The data were collected in the “Data dependent MS/MS” mode of Fourier transform-ion trap (MS-MS/MS) with the electrospray ionization interface using normalized collision energy of 35 % (collision induced dissociation). Dynamic exclusion settings were set to repeat count = 1, repeat duration = 30 s, exclusion duration = 45 s, and exclusion mass width = 10 ppm (low) and 10 ppm (high).

### Protein Identification and ouantification

The acquired data were searched against the UniProt protein sequence database of HUMAN (released on 07/09/2014) using X!Tandem algorithms in the Trans-Proteomic Pipeline (TPP, v. 4.6.3) (http://tools.proteomecenter.org/software.php). General parameters were set to: parent monoisotopic mass error set as 10 ppm, cleavage semi set as yes, missed cleavage sites set at 2, and static modification set as + 44.026215 Da on Cysteine. The peptide and protein identifications made by X!Tandem were validated by PeptideProphet [41] and ProteinProphet [42] in the TransProteomic Pipeline (http://tools.proteomecenter.org). Only validated proteins and peptides with protein probability ≥ 0.9000 and peptide probability ≥ 0.8000 were reported. False discovery rate (FDR) was estimated by a nonparametric concatenated randomized target-decoy database search [43]. As mentioned earlier, for this experiment and those TPP settings, protein identification FDR was ≤0.2 %.

Protein quantity was determined using an in-house label-free quantification software package, IdentiQuantXL [22], developed to individually and accurately align the retention time of each peptide and to apply multiple filters for exclusion of unqualified peptides to enhance label-free protein quantification. As previously described in detail [22], peptide retention time determination using clustering, extraction of peptide intensity using MASIC [44], peptide coefficient of variation calculation, and peptides correlation were all conducted within the software platform to “filter out” unqualified peptides. Using only qualified peptides, protein intensity was calculated using the formula: Protein Intensity = (intensity of peptide 1)/(peptide 1 sharing times) + … + (intensity of peptide n)/(peptide n sharing times). For a peptide shared by different proteins, the intensity of this peptide was divided by the number of times the peptide was shared.

### Validation of protein identity and quantity

Results of the LFQMS analysis were validated immunologically for beta-2-microglobulin, calbindin, clusterin, cystatin-C, glutathione S-transferase P, neutrophil gelatinase-associated lipocalin, and osteopontin. CaOx-Ia and CaOx-Id protein extracts were processed on a Bio-Plex 200 System with High Throughput Fluidics (HTF) Multiplex Array System (Bio-Rad Laboratories, Hercules, CA). The proteins were quantified simultaneously using Bio-Plex Pro™ RBM Human Kidney Toxicity Assays (panels 1 and 2) on the Bio-Plex 200 system (BIO-RAD, USA) according to manufacturer instructions. Results were compared to LFQMS data as the ratio between CaOx-Ia/CaOx-Id and these data are listed in Table 4.

**Table 4:** Validation of LFQMS Results: CaOx-Ia/CaOx-Id Ratio

Protein	LFQMS	ELISA
Beta-2-microglobulin	0.7	0.2
Calbindin	ND^a^	ND^a^
Clusterin	0.4	0.2
Cystatin-C	0.1	0.4
Glutathione S-transferase P	0.9	0.9
Neutrophil gelatinase-associated lipocalin	ND^#^	8.5
Osteopontin, Isoform 5	0.1	0.1

ND^a^ = not detected in either stone; ND^#^ = not detected in CaOx-Id

### Physicochemical properties of matrix proteins

Grand average of hydropathicity (GRAVY) scores, aliphatic indices, and number of negatively (Asp and Glu) and positively (Arg and Lys) charged residues for all identified proteins were calculated using the Protein Identification and Analysis Tools (ProtParam) on the ExPASy Server (http://web.expasy.org/protparam/) [45]. The GRAVY score for a peptide or protein is calculated as the sum of hydropathy values of all the amino acids, divided by the number of residues in the sequence. The aliphatic index of a protein is defined as the relative volume occupied by aliphatic side chains (alanine, valine, isoleucine, and leucine) and may be regarded as a positive factor for increased thermostability of globular proteins.

### Bioinformatic analysis

To investigate the functional relevance of the proteins identified and quantified in the two stones, we used two common bioinformatic tools. Protein gene-symbol lists were uploaded onto the Ingenuity Pathway Analysis (IPA) software server (http://www.ingenuity.com) (Qiagen, US) and analyzed using the Core Analysis module to rank stone-specific proteins into canonical pathways in a statistically significant manner. Additionally, UniProt identifiers were submitted to the Human Proteome Map (http://www.humanproteomemap.org) [46], GeneCards® (http://www.genecards.org) [47], and the Gene Ontology (GO) database (http://go.princeton.edu/cgi-bin/GOTermMapper) [48] via Generic Gene Ontology (GO) Term Mapper for information regarding each protein’s molecular class, biological process, cellular component, tissue specificity, and function.

## Additional files

Additional file 1: Protein quantitation data for 1,059 human kidney stone proteins. (PDF 418 kb) Additional file 2: Peptide sequence/Protein Identification, mass spectral data for 5,957 peptides. (PDF 3099 kb) Additional file 3: Human Kidney Stone Matrix Proteome Database – 1,039 proteins found in human kidney stone matrices from this study and 12 other proteomic papers. Includes details regarding Isoelectric Point, negatively charged residues (Asp + Glu), positively charged residues (Arg + Lys), Neg/Pos Ratio, Aliphatic index, GRAVY score, Molecular Class, Biological Process, Cellular component, and Function. (PDF 316 kb) Additional file 4: Ingenuity Pathway Analysis Results - Canonical Pathways associated with Kidney stone matrix proteins. (PDF 60 kb) Additional file 5: Functional Annotation Clustering of kidney stone matrix proteins obtained from the DAVID database. (PDF 100 kb)

## Abbreviations

ACN: acetonitrile
CaOx: calcium oxalate
COM: calcium oxalate monohydrate
CT: computed tomography
DTT: dithiothreitol
EDTA: ethylenediaminetetraacetic acid
GRAVY: grand average of hydropathicity
LC-MS/MS: liquid chromatography tandem mass spectrometry
LFQMS: label-free quantitative mass spectrometry
MALDI-MS/MS: matrix-assisted laser desorption ionization tandem mass spectrometry
RNA: ribonucleic acid
SDS: sodium dodecyl sulfate
THP: Tamm-Horsfall Protein
TEP: triethylphosphine
