# Supplementary material for: Label-free proteomic methodology for the analysis of human kidney stone matrix composition
Source: Proteome Sci. 2016 Feb 27;14:4. doi: 10.1186/s12953-016-0093-x (PMC4769560; doi:10.1186/s12953-016-0093-x)
Supplement: Additional file 1: — Protein quantitation data for 1,059 human kidney stone proteins. (PDF 418 kb) [file 12953_2016_93_MOESM1_ESM.pdf]

Quantification of all proteins

UniProt Protein ID	UniProt Protein Entry	Gene Name	Protein Name	CaOx-Ia	CaOx-Id
Q15029-3	U5S1_HUMAN	EFTUD2	116 kDa U5 small nuclear ribonucleoprotein component, Isoform 3	-	692,430
K7EJ74	K7EJ74_HUMAN	EFTUD2*	116 kDa U5 small nuclear ribonucleoprotein component, splice variant or isoform	-	398,580
Q9NRX4	PHP14_HUMAN	PHPT1	14 kDa phosphohistidine phosphatase	-	20,179,850
P31946	1433B_HUMAN	YWHAB	14-3-3 protein beta/alpha	4,562,610	10,026,698
P62258	1433E_HUMAN	YWHAE	14-3-3 protein epsilon, splice variant or isoform	4,934,553	11,343,022
K7EM20	K7EM20_HUMAN	YWHAE*	14-3-3 protein epsilon, splice variant or isoform	506,932	-
Q04917	1433F_HUMAN	YWHAH*	14-3-3 protein eta	1,431,850	2,806,250
P61981	1433G_HUMAN	YWHAG	14-3-3 protein gamma	26,191,900	18,830,100
P31947	1433S_HUMAN	SFN	14-3-3 protein sigma	2,761,322	19,743,069
P27348	1433T_HUMAN	YWHAQ	14-3-3 protein theta	9,057,272	4,058,577
P15428-5	PGDH_HUMAN	HPGD	15-hydroxyprostaglandin dehydrogenase [NAD(+)], , Isoform 5	25,361,348	37,475,178
P15428-2	PGDH_HUMAN	HPGD	15-hydroxyprostaglandin dehydrogenase [NAD(+)], Isoform 2	24,395,854	37,306,103
P15428	PGDH_HUMAN	HPGD	15-hydroxyprostaglandin dehydrogenase [NAD(+)], splice variant or isoform	26,839,323	40,936,553
E9PBZ2	E9PBZ2_HUMAN	HPGD	15-hydroxyprostaglandin dehydrogenase [NAD(+)], splice variant or isoform	10,739,607	17,815,736
D6RHF8	D6RHF8_HUMAN	HPGD	15-hydroxyprostaglandin dehydrogenase [NAD(+)], splice variant or isoform	9,170,214	14,223,286
P62333	PRS10_HUMAN	PSMC6	26S protease regulatory subunit 10B	-	20,870,650
B4DR63	B4DR63_HUMAN	PSMC1	26S protease regulatory subunit 4	13,747,520	31,427,450
E9PM69	E9PM69_HUMAN	PSMC3	26S protease regulatory subunit 6A, splice variant or isoform	-	2,682,313
E9PKD5	E9PKD5_HUMAN	PSMC3	26S protease regulatory subunit 6A, splice variant or isoform	-	2,220,563
P62195	PRS8_HUMAN	PSMC5	26S protease regulatory subunit 8, splice variant or isoform	-	12,456,443
J3QSE0	J3QSE0_HUMAN	PSMC5	26S protease regulatory subunit 8, splice variant or isoform	-	471,793
Q99460	PSMD1_HUMAN	PSMD1*	26S proteasome non-ATPase regulatory subunit 1	408,281	419,934
O00231	PSD11_HUMAN	PSMD11*	26S proteasome non-ATPase regulatory subunit 11	449,733	532,950
O00232	PSD12_HUMAN	PSMD12	26S proteasome non-ATPase regulatory subunit 12	-	29,558,700
H7C0L5	H7C0L5_HUMAN	ITIH4	35 kDa inter-alpha-trypsin inhibitor heavy chain H4	613,342	625,834
Q9BUT1	BDH2_HUMAN	BDH2	3-hydroxybutyrate dehydrogenase type 2, splice variant or isoform	-	4,140,908
D6RBF6	D6RBF6_HUMAN	BDH2*	3-hydroxybutyrate dehydrogenase type 2, splice variant or isoform	-	67,858
F5GZQ3	F5GZQ3_HUMAN	HADHB	3-ketoacyl-CoA thiolase, splice variant or isoform	10,552,042	963,050
B5MD38	B5MD38_HUMAN	HADHB*	3-ketoacyl-CoA thiolase, splice variant or isoform	466,042	280,000
P25325	THTM_HUMAN	MPST	3-mercaptopyruvate sulfurtransferase	-	2,125,188
F6U211	F6U211_HUMAN	RPS10*	40S ribosomal protein S10	-	717,593
E9PPT0	E9PPT0_HUMAN	RPS2*	40S ribosomal protein S2	136,445	384,729
D6RD47	D6RD47_HUMAN	RPS23	40S ribosomal protein S23	5,115,525	19,216,225
E9PJH4	E9PJH4_HUMAN	RPS3	40S ribosomal protein S3	2,092,674	1,960,582
D6RAT0	D6RAT0_HUMAN	RPS3A*	40S ribosomal protein S3a	185,427	960,864
P62701	RS4X_HUMAN	RPS4X	40S ribosomal protein S4, X isoform	-	100,825,850
M0QZN2	M0QZN2_HUMAN	RPS5*	40S ribosomal protein S5	-	325,200
A2A3R5	A2A3R5_HUMAN	RPS6*	40S ribosomal protein S6	275,234	643,933
P62081	RS7_HUMAN	RPS7	40S ribosomal protein S7	6,029,700	7,256,900
Q5JR95	Q5JR95_HUMAN	RPS8	40S ribosomal protein S8, splice variant or isoform	-	18,920,000
P62241	RS8_HUMAN	RPS8	40S ribosomal protein S8, splice variant or isoform	-	15,553,750
P08865	RSSA_HUMAN	RPSA*	40S ribosomal protein SA	197,689	-
B4DXY7	B4DXY7_HUMAN	ALDH9A1	4-trimethylaminobutyraldehyde dehydrogenase	9,770,250	28,752,250
P10809	CH60_HUMAN	HSPD1	60 kDa heat shock protein, mitochondrial	-	43,376,192
P05388	RLA0_HUMAN	RPLP0	60S acidic ribosomal protein P0, splice variant or isoform	24,979,701	60,092,132
F8VWS0	F8VWS0_HUMAN	RPLP0*	60S acidic ribosomal protein P0, splice variant or isoform	182,050	249,450
P05386	RLA1_HUMAN	RPLP1	60S acidic ribosomal protein P1	1,612,526	2,760,683
P05387	RLA2_HUMAN	RPLP2	60S acidic ribosomal protein P2	-	33,120,850
E7EPB3	E7EPB3_HUMAN	RPL14	60S ribosomal protein L14	7,525,903	6,364,275
P61313	RL15_HUMAN	RPL15	60S ribosomal protein L15	-	979,995
G3V203	G3V203_HUMAN	RPL18*	60S ribosomal protein L18	-	496,817
K7EKS7	K7EKS7_HUMAN	RPL22*	60S ribosomal protein L22	1,448,957	2,423,858
A8MUS3	A8MUS3_HUMAN	RPL23A	60S ribosomal protein L23a	2,765,117	5,233,867
P39023	RL3_HUMAN	RPL3	60S ribosomal protein L3, splice variant or isoform	-	6,932,825
G5E9G0	G5E9G0_HUMAN	RPL3	60S ribosomal protein L3, splice variant or isoform	-	2,303,775
F8W727	F8W727_HUMAN	RPL32*	60S ribosomal protein L32	174,959	319,536
P46777	RL5_HUMAN	RPL5*	60S ribosomal protein L5	1,714,875	16,326,000
P62424	RL7A_HUMAN	RPL7A	60S ribosomal protein L7a, splice variant or isoform, splice variant or isoform	-	48,045,875
Q5T8U3	Q5T8U3_HUMAN	RPL7A*	60S ribosomal protein L7a, splice variant or isoform, splice variant or isoform	-	349,875
B4DQJ8	B4DQJ8_HUMAN	PGD	6-phosphogluconate dehydrogenase, decarboxylating, splice variant or isoform	5,585,723	-
K7EM49	K7EM49_HUMAN	PGD*	6-phosphogluconate dehydrogenase, decarboxylating, splice variant or isoform	132,357	-
O95336	6PGL_HUMAN	PGLS	6-phosphogluconolactonase, splice variant or isoform	38,116,808	32,759,317
M0R0U3	M0R0U3_HUMAN	PGLS	6-phosphogluconolactonase, splice variant or isoform	6,583,383	9,135,317
P11021	GRP78_HUMAN	HSPA5	78 kDa glucose-regulated protein	6,668,321	16,188,142
A6NFN2	A6NFN2_HUMAN	ABI1*	Abl interactor 1	195,591	845,688
E7EMM4	E7EMM4_HUMAN	ASAH1	Acid ceramidase	10,856,245	57,069,434
Q13510-3	ASAH1_HUMAN	ASAH1	Acid ceramidase, Isoform 3	9,324,420	41,150,910
Q5TB19	Q5TB19_HUMAN	ANP32E*	Acidic (Leucine-rich) nuclear phosphoprotein 32 family, member E	768,963	463,538
H0YN26	H0YN26_HUMAN	ANP32A	Acidic leucine-rich nuclear phosphoprotein 32 family member A	-	45,886,992
Q92688	AN32B_HUMAN	ANP32B	Acidic leucine-rich nuclear phosphoprotein 32 family member B	-	36,529,417
Q9BTT0	AN32E_HUMAN	ANP32E	Acidic leucine-rich nuclear phosphoprotein 32 family member E	2,378,013	2,786,063
P60709	ACTB_HUMAN	ACTB	Actin, cytoplasmic 1	747,279,184	477,710,421
P61160	ARP2_HUMAN	ACTR2	Actin-related protein 2, splice variant or isoform	7,889,596	25,498,508
F5H6T1	F5H6T1_HUMAN	ACTR2	Actin-related protein 2, splice variant or isoform	830,408	3,590,158
F8WDD7	F8WDD7_HUMAN	ARPC4	Actin-related protein 2/3 complex subunit 4, splice variant or isoform	5,560,556	-
R4GN08	R4GN08_HUMAN	ARPC4*	Actin-related protein 2/3 complex subunit 4, splice variant or isoform	3,441,786	-
P61158	ARP3_HUMAN	ACTR3	Actin-related protein 3, splice variant or isoform	10,567,070	18,854,995
B4DXW1	B4DXW1_HUMAN	ACTR3	Actin-related protein 3, splice variant or isoform	1,551,150	5,062,945
Q9P1U1	ARP3B_HUMAN	ACTR3B*	Actin-related protein 3B	409,933	-
P53999	TCP4_HUMAN	SUB1	Activated RNA polymerase II transcriptional coactivator p15	-	24,930,711
Q8WXI4	ACO11_HUMAN	ACOT11	Acyl-coenzyme A thioesterase 11	-	26,930,971
Q8WXI4-2	ACO11_HUMAN	ACOT11	Acyl-coenzyme A thioesterase 11Isoform 2	-	65,718,971

P23526	SAHH_HUMAN	AHCY	Adenosylhomocysteinase	-	17,287,925
P23526-2	SAHH_HUMAN	AHCY*	Adenosylhomocysteinase, Isoform 2	-	771,425
P30520	PURA2_HUMAN	ADSS*	Adenylosuccinate synthetase isozyme 2	2,102,250	2,563,400
Q5T0R1	Q5T0R1_HUMAN	CAP1	Adenylyl cyclase-associated protein	385,287	278,747
Q01518	CAP1_HUMAN	CAP1	Adenylyl cyclase-associated protein 1	19,625,646	32,831,047
P84077	ARF1_HUMAN	ARF1	ADP-ribosylation factor 1	3,409,017	-
F5H0C7	F5H0C7_HUMAN	ARF3	ADP-ribosylation factor 3	2,351,632	-
C9JPM4	C9JPM4_HUMAN	ARF4	ADP-ribosylation factor 4	2,492,196	1,729,953
P18085	ARF4_HUMAN	ARF4	ADP-ribosylation factor 4, splice variant or isoform	11,080,796	10,299,328
C9JAK5	C9JAK5_HUMAN	ARF4	ADP-ribosylation factor 4, splice variant or isoform	-	9,775,859
P84085	ARF5_HUMAN	ARF5	ADP-ribosylation factor 5	784,579	-
O00468	AGRIN_HUMAN	AGRN	Agrin	-	151,314,342
O00468-2	AGRIN_HUMAN	AGRN	Agrin, Isoform 2	-	6,359,236
P14550	AK1A1_HUMAN	AKR1A1	Alcohol dehydrogenase [NADP(+)]	12,589,592	56,919,851
Q04828	AK1C1_HUMAN	AKR1C1	Aldo-keto reductase family 1 member C1	72,181,727	79,395,916
P52895	AK1C2_HUMAN	AKR1C2	Aldo-keto reductase family 1 member C2, splice variant or isoform	-	7,120,645
B4DK69	B4DK69_HUMAN	AKR1C2	Aldo-keto reductase family 1 member C2, splice variant or isoform	-	4,133,329
P42330	AK1C3_HUMAN	AKR1C3	Aldo-keto reductase family 1 member C3	47,055,313	56,560,417
P15121	ALDR_HUMAN	AKR1B1	Aldose reductase	-	147,775,953
B4DQI4	B4DQI4_HUMAN	ABHD14B	Alpha/beta hydrolase domain-containing protein 14B, splice variant or isoform	10,506,491	28,164,517
F8W9U3	F8W9U3_HUMAN	ABHD14B	Alpha/beta hydrolase domain-containing protein 14B, splice variant or isoform	7,777,924	25,525,000
P02763	A1AG1_HUMAN	ORM1	Alpha-1-acid glycoprotein 1	7,484,872	21,912,450
P19652	A1AG2_HUMAN	ORM2	Alpha-1-acid glycoprotein 2	9,672,250	13,103,700
P01011	AACT_HUMAN	SERPINA3	Alpha-1-antichymotrypsin, splice variant or isoform	139,658,660	127,282,492
G3V3A0	G3V3A0_HUMAN	SERPINA3	Alpha-1-antichymotrypsin, splice variant or isoform	75,385,641	22,082,651
G3V5I3	G3V5I3_HUMAN	SERPINA3	Alpha-1-antichymotrypsin, splice variant or isoform	32,504,250	55,687,750
P01009	A1AT_HUMAN	SERPINA1	Alpha-1-antitrypsin	2,011,172,162	1,259,585,313
P04217	A1BG_HUMAN	A1BG	Alpha-1B-glycoprotein	36,675,864	53,657,184
P04217-2	A1BG_HUMAN	A1BG	Alpha-1B-glycoprotein, Isoform 2	24,257,175	45,137,200
P08697	A2AP_HUMAN	SERPINF2	Alpha-2-antiplasmin	55,209,779	75,712,519
P01023	A2MG_HUMAN	A2M	Alpha-2-macroglobulin, splice variant or isoform	320,911,418	244,463,731
F8W7L3	F8W7L3_HUMAN	A2M*	Alpha-2-macroglobulin, splice variant or isoform	509,742	920,925
H9KV75	H9KV75_HUMAN	ACTN1	Alpha-actinin-1, splice variant or isoform	2,635,801	3,441,563
H0YJ11	H0YJ11_HUMAN	ACTN1	Alpha-actinin-1, splice variant or isoform	218,667	575,763
O43707	ACTN4_HUMAN	ACTN4	Alpha-actinin-4, splice variant or isoform	496,911	1,147,153
F5GXS2	F5GXS2_HUMAN	ACTN4	Alpha-actinin-4, splice variant or isoform	352,274	854,053
E9PRA8	E9PRA8_HUMAN	CRYAB	Alpha-crystallin B chain, splice variant or isoform	-	5,835,517
E9PNH7	E9PNH7_HUMAN	CRYAB	Alpha-crystallin B chain, splice variant or isoform	-	4,890,192
P06733	ENOA_HUMAN	ENO1	Alpha-enolase	84,413,185	204,566,624
M0R0Y2	M0R0Y2_HUMAN	NAPA	Alpha-soluble NSF attachment protein	-	2,990,750
Q12904	AIMP1_HUMAN	AIMP1	Aminoacyl tRNA synthase complex-interacting multifunctional protein 1	-	13,051,025
B4DNW0	B4DNW0_HUMAN	ACY1	Aminoacylase-1	-	9,007,378
Q9H4A4	AMPB_HUMAN	RNPEP	Aminopeptidase B, splice variant or isoform	11,776,818	-
A6NKB8	A6NKB8_HUMAN	RNPEP	Aminopeptidase B, splice variant or isoform	11,041,814	-
P05067	A4_HUMAN	APP	Amyloid beta A4 protein	5,382,719	78,856,818
Q06481	APLP2_HUMAN	APLP2	Amyloid-like protein 2	-	44,053,554
J3KPC0	J3KPC0_HUMAN	ZFAND4	AN1-type zinc finger protein 4	30,624,567	16,815,834
Q02763	TIE2_HUMAN	TEK	Angiopoietin-1 receptor	2,436,843	718,709
Q8NCH7	Q8NCH7_HUMAN	ANGPTL2	Angiopoietin-like 2	221,762,312	219,933,464
Q9UKU9	ANGL2_HUMAN	ANGPTL2	Angiopoietin-related protein 2	240,393,462	234,826,614
K7EKF6	K7EKF6_HUMAN	ANGPTL6	Angiopoietin-related protein 6	2,822,100	3,486,000
B4DQW8	B4DQW8_HUMAN	ANKS1A*	Ankyrin repeat and SAM domain-containing protein 1A	1,389,750	784,975
D6RA82	D6RA82_HUMAN	ANXA3*	Annexin A3	2,312,425	10,791,250
Q9UJ72	ANX10_HUMAN	ANXA10	Annexin A10	19,636,950	4,900,300
P50995	ANX11_HUMAN	ANXA11*	Annexin A11	-	1,738,475
P09525	ANXA4_HUMAN	ANXA4	Annexin A4	-	9,488,758
E9PHT9	E9PHT9_HUMAN	ANXA5*	Annexin A5	-	688,950
O76027	ANXA9_HUMAN	ANXA9	Annexin A9	-	11,053,484
H0YMU9	H0YMU9_HUMAN	ANXA2	AnnexinA2	-	1,636,233
O95994	AGR2_HUMAN	AGR2	Anterior gradient protein 2 homolog, splice variant or isoform	-	9,309,079
B5MC07	B5MC07_HUMAN	AGR2	Anterior gradient protein 2 homolog, splice variant or isoform	-	5,890,104
P01008	ANT3_HUMAN	SERPINC1	Antithrombin-III	264,864,557	362,426,761
O00203	AP3B1_HUMAN	AP3B1	AP-3 complex subunit beta-1	-	13,193,675
E7EMM2	E7EMM2_HUMAN	AP3D1	AP-3 complex subunit delta	6,164,957	2,072,365
P02647	APOA1_HUMAN	APOA1	Apolipoprotein A-I	408,731,905	1,117,294,931
P02652	APOA2_HUMAN	APOA2	Apolipoprotein A-II, splice variant or isoform	-	58,582,591
V9GYE3	V9GYE3_HUMAN	APOA2	Apolipoprotein A-II, splice variant or isoform	-	49,812,516
P06727	APOA4_HUMAN	APOA4	Apolipoprotein A-IV	1,123,027,556	525,438,423
P04114	APOB_HUMAN	APOB	Apolipoprotein B-100	760,463,919	722,203,134
B0YIW2	B0YIW2_HUMAN	APOC3*	Apolipoprotein C-III	178,982	530,536
P05090	APOD_HUMAN	APOD	Apolipoprotein D, splice variant or isoform	319,567,262	993,103,025
C9JF17	C9JF17_HUMAN	APOD	Apolipoprotein D, splice variant or isoform	204,796,987	889,591,000
P02649	APOE_HUMAN	APOE	Apolipoprotein E	118,977,682	225,158,171
G8JL88	G8JL88_HUMAN	APOL1*	Apolipoprotein L1	154,654	196,102
O95445	APOM_HUMAN	APOM	Apolipoprotein M	3,077,973	5,001,153
P08519	APOA_HUMAN	LPA	Apolipoprotein(a)	-	30,881,666
B0YIW6	B0YIW6_HUMAN	ARCN1*	Archain 1	1,146,325	3,705,300
P54136	SYRC_HUMAN	RARS	Arginine--tRNA ligase, cytoplasmic, splice variant or isoform	88,336,542	65,302,717
F5H3T8	F5H3T8_HUMAN	RARS	Arginine--tRNA ligase, cytoplasmic, splice variant or isoform	832,536	4,069,617
P00966	ASSY_HUMAN	ASS1	Argininosuccinate synthase, splice variant or isoform	198,487,087	601,203,775
Q5T6L6	Q5T6L6_HUMAN	ASS1	Argininosuccinate synthase, splice variant or isoform	8,067,049	24,891,725
P14868	SYDC_HUMAN	DARS	Aspartate--tRNA ligase, cytoplasmic, splice variant or isoform	-	81,351,050
C9J7S3	C9J7S3_HUMAN	DARS	Aspartate--tRNA ligase, cytoplasmic, splice variant or isoform	-	3,964,263
C9JQM9	C9JQM9_HUMAN	DARS*	Aspartate--tRNA ligase, cytoplasmic, splice variant or isoform	-	2,511,700
P25705-2	ATPA_HUMAN	ATP5A1	ATP synthase subunit alpha, mitochondrial, Isoform 2	641,538	1,603,944
P25705	ATPA_HUMAN	ATP5A1	ATP synthase subunit alpha, mitochondrial, splice variant or isoform	102,997,692	91,313,567
K7EJP1	K7EJP1_HUMAN	ATP5A1	ATP synthase subunit alpha, mitochondrial, splice variant or isoform	247,844	453,544

K7ESA0	K7ESA0_HUMAN	ATP5A1*	ATP synthase subunit alpha, mitochondrial, splice variant or isoform	74,284	89,919
H0YH81	H0YH81_HUMAN	ATP5B	ATP synthase subunit beta	1,386,800	1,861,675
P06576	ATPB_HUMAN	ATP5B	ATP synthase subunit beta, mitochondrial	78,435,590	108,271,762
Q96QE3	ATAD5_HUMAN	ATAD5	ATPase family AAA domain-containing protein 5	2,879,407	10,350,000
Q8NE71	ABCF1_HUMAN	ABCF1	ATP-binding cassette sub-family F member 1, splice variant or isoform	-	9,508,020
Q5STZ7	Q5STZ7_HUMAN	ABCF1	ATP-binding cassette sub-family F member 1, splice variant or isoform	-	9,233,375
P17858	PFKAL_HUMAN	PFKL	ATP-dependent 6-phosphofructokinase, liver type	14,116,855	21,997,280
P08237	PFKAM_HUMAN	PFKM	ATP-dependent 6-phosphofructokinase, muscle type	9,540,088	-
Q01813	PFKAP_HUMAN	PFKP	ATP-dependent 6-phosphofructokinase, platelet type	-	6,103,300
Q08211	DHX9_HUMAN	DHX9	ATP-dependent RNA helicase A	1,193,644,000	116,433,750
Q92499	DDX1_HUMAN	DDX1	ATP-dependent RNA helicase DDX1	-	9,752,750
O00571	DDX3X_HUMAN	DDX3X	ATP-dependent RNA helicase DDX3X	-	46,676,534
O15523	DDX3Y_HUMAN	DDX3Y	ATP-dependent RNA helicase DDX3Y	-	3,299,363
P20160	CAP7_HUMAN	AZU1	Azurocidin	127,221,938	-
P02730	B3AT_HUMAN	SLC4A1	Band 3 anion transport protein	20,623,731	27,460,643
Q9H4G0	E41L1_HUMAN	EPB41L1	Band 4.1-like protein 1, splice variant or isoform	651,936	1,163,677
H0Y482	H0Y482_HUMAN	EPB41L1*	Band 4.1-like protein 1, splice variant or isoform	198,873	313,810
O75531	BAF_HUMAN	BANF1*	Barrier-to-autointegration factor	-	1,088,566
P98160	PGBM_HUMAN	HSPG2	Basement membrane-specific heparan sulfate proteoglycan core protein	67,731,700	123,288,864
Q86UU0	BCL9L_HUMAN	BCL9L*	B-cell CLL/lymphoma 9-like protein	12,329,750	6,183,750
P61769	B2MG_HUMAN	B2M	Beta-2-microglobulin	6,408,684	9,388,976
H0YLF3	H0YLF3_HUMAN	B2M*	Beta-2-microglobulin form pI 5.3	171,539	308,001
Q562R1	ACTBL_HUMAN	ACTBL2	Beta-actin-like protein 2	69,583,667	78,504,667
P60022	DEFB1_HUMAN	DEFB1*	Beta-defensin 1	720,658	-
P07814	SYEP_HUMAN	EPRS	Bifunctional glutamate/proline--tRNA ligase, splice variant or isoform, splice variant o	-	15,320,875
V9GYZ6	V9GYZ6_HUMAN	EPRS	Bifunctional glutamate/proline--tRNA ligase, splice variant or isoform, splice variant o	-	3,659,625
P31939	PUR9_HUMAN	ATIC	Bifunctional purine biosynthesis protein PURH	-	78,374,720
P21810	PGS1_HUMAN	BGN	Biglycan	-	124,331,530
Q8NFC6	BD1L1_HUMAN	BOD1L1	Biorientation of chromosomes in cell division protein 1-like 1	44,179,700	28,829,400
P13727	PRG2_HUMAN	PRG2	Bone marrow proteoglycan	102,544,072	-
B7Z1K0	B7Z1K0_HUMAN	BAI3	Brain-specific angiogenesis inhibitor 3	7,407,633	24,771,500
F5H2F4	F5H2F4_HUMAN	MTHFD1	C-1-tetrahydrofolate synthase, cytoplasmic	625,106	1,813,222
P04003	C4BPA_HUMAN	C4BPA	C4b-binding protein alpha chain	187,995,829	1,301,320,701
P20851	C4BPB_HUMAN	C4BPB	C4b-binding protein beta chain	-	165,317,508
Q9UII8	Q9UII8_HUMAN	CDH1	Cadherin 1, type 1, E-cadherin (Epithelial)	2,143,841	6,267,225
P55291	CAD15_HUMAN	CDH15	Cadherin-15	-	5,429,750
D6R938	D6R938_HUMAN	CAMK2D	Calcium/calmodulin-dependent protein kinase (CaM kinase) II delta	-	616,720
K7EL21	K7EL21_HUMAN	CAPS	Calcyphosin	-	65,966,759
Q13938-3	CAYP1_HUMAN	CAPS	Calcyphosin, Isoform 2	-	42,104,151
B4DGP8	B4DGP8_HUMAN	CANX	Calnexin	-	1,029,933
K7ELJ7	K7ELJ7_HUMAN	CAPNS1	Calpain small subunit 1, splice variant or isoform	2,424,574	3,107,765
K7EIV0	K7EIV0_HUMAN	CAPNS1	Calpain small subunit 1, splice variant or isoform	2,285,621	2,804,105
K7EM73	K7EM73_HUMAN	CAPNS1	Calpain small subunit 1, splice variant or isoform	411,591	1,110,548
K7EKD8	K7EKD8_HUMAN	CAPNS1*	Calpain small subunit 1, splice variant or isoform	138,953	303,660
P07384	CAN1_HUMAN	CAPN1	Calpain-1 catalytic subunit, splice variant or isoform	-	152,230,000
E9PRM1	E9PRM1_HUMAN	CAPN1	Calpain-1 catalytic subunit, splice variant or isoform	-	8,739,129
P27797	CALR_HUMAN	CALR	Calreticulin	180,330,287	192,368,347
P00915	CAH1_HUMAN	CA1	Carbonic anhydrase 1	3,044,321	3,953,125
P00918	CAH2_HUMAN	CA2	Carbonic anhydrase 2	4,849,550	102,054,750
P16152	CBR1_HUMAN	CBR1	Carbonyl reductase [NADPH] 1	-	86,066,261
Q96DG6	CMBL_HUMAN	CMBL	Carboxymethylenebutenolidase homolog	-	21,575,137
Q96IY4	CBPB2_HUMAN	CPB2	Carboxypeptidase B2	8,217,900	55,303,800
Q9Y646	CBPQ_HUMAN	CPQ	Carboxypeptidase Q	-	3,331,710
Q13985	Q13985_HUMAN	CEA*	Carcinoembryonic antigen	241,923	664,925
P40199	CEAM6_HUMAN	CEACAM6	Carcinoembryonic antigen-related cell adhesion molecule 6	3,792,626	8,269,575
P31997	CEAM8_HUMAN	CEACAM8	Carcinoembryonic antigen-related cell adhesion molecule 8	-	36,045,600
B4DKJ3	B4DKJ3_HUMAN	COMP	Cartilage oligomeric matrix protein	-	1,886,217
P04040	CATA_HUMAN	CAT	Catalase	-	27,632,850
P21964	COMT_HUMAN	COMT	Catechol O-methyltransferase, splice variant or isoform	11,101,184	24,686,460
E7EMS6	E7EMS6_HUMAN	COMT	Catechol O-methyltransferase, splice variant or isoform	1,446,032	4,785,910
F8W845	F8W845_HUMAN	CTNNA1	Catenin alpha-1	1,177,350	2,585,888
C9JZR2	C9JZR2_HUMAN	CTNND1	Catenin delta-1	8,418,902	1,703,216
J3KNB4	J3KNB4_HUMAN	CAMP*	Cathelicidin antimicrobial peptide	315,714	-
P07858	CATB_HUMAN	CTSB	Cathepsin B	-	337,800,764
P07339	CATD_HUMAN	CTSD	Cathepsin D	-	130,075,666
P08311	CATG_HUMAN	CTSG	Cathepsin G	210,068,194	120,361,928
P07711	CATL1_HUMAN	CTSL	Cathepsin L1	-	37,410,570
Q9UBR2	CATZ_HUMAN	CTSZ	Cathepsin Z	-	44,164,900
E7EPC6	E7EPC6_HUMAN	CD44*	CD44 antigen	-	292,964
B1AP15	B1AP15_HUMAN	CD55	CD55 antigen, decay accelerating factor for complement (Cromer blood group)	831,351	1,416,975
E9PNW4	E9PNW4_HUMAN	CD59	CD59 glycoprotein	-	21,573,392
Q9NZ45	CISD1_HUMAN	CISD1*	CDGSH iron-sulfur domain-containing protein 1	1,220,067	1,529,750
Q8N111	CEND_HUMAN	CEND1*	Cell cycle exit and neuronal differentiation protein 1	45,947,000	8,813,000
P60953	CDC42_HUMAN	CDC42	Cell division control protein 42 homolog, splice variant or isoform	6,025,017	8,911,925
Q5JYX0	Q5JYX0_HUMAN	CDC42	Cell division control protein 42 homolog, splice variant or isoform	4,233,617	7,335,425
E9PFZ2	E9PFZ2_HUMAN	CP	Ceruloplasmin, splice variant or isoform	16,273,327	41,302,410
D6RE86	D6RE86_HUMAN	CP*	Ceruloplasmin, splice variant or isoform	824,984	2,323,700
H7C5R1	H7C5R1_HUMAN	CP	Ceruloplasmin, splice variant or isoform	-	9,508,672
B7Z1C9	B7Z1C9_HUMAN	CCT7	Chaperonin containing TCP1, subunit 7 (Eta)	5,344,475	5,473,926
O00299	CLIC1_HUMAN	CLIC1	Chloride intracellular channel protein 1	33,107,513	-
Q13185	CBX3_HUMAN	CBX3*	Chromobox protein homolog 3	-	891,525
Q00610	CLH1_HUMAN	CLTC	Clathrin heavy chain 1	29,140,916	35,934,881
P10909	CLUS_HUMAN	CLU	Clusterin	36,565,862	82,764,754
P10909-2	CLUS_HUMAN	CLU	Clusterin, Isoform 2	57,914,845	194,838,902
P00740	FA9_HUMAN	F9	Coagulation factor IX	54,913,541	119,429,550
P00740-2	FA9_HUMAN	F9	Coagulation factor IX, Isoform 2	6,706,358	26,675,850
P12259	FA5_HUMAN	F5*	Coagulation factor V	987,183	1,764,550

P08709	FA7_HUMAN	F7	Coagulation factor VII	-	7,780,566
P00742	FA10_HUMAN	F10	Coagulation factor X	96,339,711	513,013,791
E9PGP2	E9PGP2_HUMAN	F11	Coagulation factor XI	142,356,025	38,303,101
P00748	FA12_HUMAN	F12	Coagulation factor XII	567,722,450	88,396,830
P00488	F13A_HUMAN	F13A1	Coagulation factor XIII A chain	4,255,698	4,081,900
B4DZI8	B4DZI8_HUMAN	COPB2	Coatomer protein complex, subunit beta 2 (Beta prime)	-	26,185,375
P53621	COPA_HUMAN	COPA	Coatomer subunit alpha	-	12,852,700
Q9Y678	COPG1_HUMAN	COPG1	Coatomer subunit gamma-1	-	3,430,807
Q9UBF2	COPG2_HUMAN	COPG2*	Coatomer subunit gamma-2	-	594,157
G3V1A4	G3V1A4_HUMAN	CFL1	Cofilin 1 (Non-muscle)	36,232,657	11,282,335
E9PP50	E9PP50_HUMAN	CFL1	Cofilin-1, splice variant or isoform	117,207	268,969
E9PLJ3	E9PLJ3_HUMAN	CFL1*	Cofilin-1, splice variant or isoform	42,383	138,092
Q16204	CCDC6_HUMAN	CCDC6*	Coiled-coil domain-containing protein 6	-	1,165,850
E9PKN4	E9PKN4_HUMAN	CSDE1*	Cold shock domain-containing protein E1	146,595	263,142
O75534	CSDE1_HUMAN	CSDE1	Cold shock domain-containing protein E1, splice variant or isoform, splice variant or is	-	1,646,802
E9PLT0	E9PLT0_HUMAN	CSDE1	Cold shock domain-containing protein E1, splice variant or isoform, splice variant or is	-	1,383,660
P20908	CO5A1_HUMAN	COL5A1	Collagen alpha-1(V) chain	46,072,150	31,089,900
P12109	CO6A1_HUMAN	COL6A1	Collagen alpha-1(VI) chain	59,750,950	-
Q05707	COEA1_HUMAN	COL14A1	Collagen alpha-1(XIV) chain	6,486,000	7,106,900
P39059	COFA1_HUMAN	COL15A1	Collagen alpha-1(XV) chain	159,570,181	-
P39060	COIA1_HUMAN	COL18A1	Collagen alpha-1(XVIII) chain	-	10,203,983
P02745	C1QA_HUMAN	C1QA	Complement C1q subcomponent subunit A, splice variant or isoform, splice variant or	-	12,967,075
X6RLJ0	X6RLJ0_HUMAN	C1QA*	Complement C1q subcomponent subunit A, splice variant or isoform, splice variant or	-	3,959,475
D6R934	D6R934_HUMAN	C1QB	Complement C1q subcomponent subunit B, splice variant or isoform	12,853,782	22,681,458
D6RGJ1	D6RGJ1_HUMAN	C1QB	Complement C1q subcomponent subunit B, splice variant or isoform	8,517,884	12,663,483
P02747	C1QC_HUMAN	C1QC*	Complement C1q subcomponent subunit C	3,886,050	3,693,800
H0YFH3	H0YFH3_HUMAN	C1R	Complement C1r subcomponent, splice variant or isoform	965,989	2,143,588
R4GMN6	R4GMN6_HUMAN	C1R	Complement C1r subcomponent, splice variant or isoform	799,977	1,983,700
F5H1V0	F5H1V0_HUMAN	C1R*	Complement C1r subcomponent, splice variant or isoform	-	159,888
P09871	C1S_HUMAN	C1S	Complement C1s subcomponent, splice variant or isoform	89,666,262	37,969,859
F8WCZ6	F8WCZ6_HUMAN	C1S	Complement C1s subcomponent, splice variant or isoform	19,717,265	11,756,784
H0Y5D1	H0Y5D1_HUMAN	C1S	Complement C1s subcomponent, splice variant or isoform	13,902,567	5,930,784
F5H7T4	F5H7T4_HUMAN	C1S	Complement C1s subcomponent, splice variant or isoform	12,466,500	4,562,525
P06681	CO2_HUMAN	C2	Complement C2, splice variant or isoform	137,452,500	20,420,250
P06681	CO2_HUMAN	C2*	Complement C2, splice variant or isoform	16,148,884	16,551,971
H0Y3H6	H0Y3H6_HUMAN	C2	Complement C2b fragment	15,772,928	15,908,896
P01024	CO3_HUMAN	C3	Complement C3	3,845,940,227	2,208,772,449
P0C0L4	CO4A_HUMAN	C4A	Complement C4-A	470,611,649	515,933,209
P0C0L5	CO4B_HUMAN	C4B	Complement C4-B	458,451,101	512,279,159
P01031	CO5_HUMAN	C5	Complement C5	275,901,762	158,275,187
I3L3B0	I3L3B0_HUMAN	C1QBP*	Complement component 1 Q subcomponent-binding protein, mitochondrial	-	3,513,567
P13671	CO6_HUMAN	C6*	Complement component C6	11,827,000	16,499,500
P10643	CO7_HUMAN	C7*	Complement component C7	581,116	1,028,524
F5GY80	F5GY80_HUMAN	C8B	Complement component C8 beta chain	12,283,610	31,838,737
P02748	CO9_HUMAN	C9	Complement component C9	275,486,989	234,830,149
B4E1Z4	B4E1Z4_HUMAN	CFB	Complement factor B	312,879,551	534,637,261
P08603	CFAH_HUMAN	CFH	Complement factor H	9,278,850	42,098,500
B1AKG0	B1AKG0_HUMAN	CFHR1	Complement factor H-related protein 1	20,949,527	8,149,075
Q5VYL6	Q5VYL6_HUMAN	CFHR5*	Complement factor H-related protein 5	125,277	392,325
C9JFE4	C9JFE4_HUMAN	GPS1	COP9 signalosome complex subunit 1	1,095,427	1,524,220
Q9BT78-2	CSN4_HUMAN	COPS4	COP9 signalosome complex subunit 4, Isoform 2	-	5,898,440
D6RAX7	D6RAX7_HUMAN	COPS4	COP9 signalosome complex subunit 4, splice variant or isoform, splice variant or isofo	-	6,041,476
D6RD63	D6RD63_HUMAN	COPS4	COP9 signalosome complex subunit 4, splice variant or isoform, splice variant or isofo	-	3,474,876
H3BRY3	H3BRY3_HUMAN	CORO1A	Coronin, splice variant or isoform	14,780,836	28,761,054
E7EW44	E7EW44_HUMAN	CORO1B	Coronin, splice variant or isoform	1,635,036	2,362,075
H3BNA2	H3BNA2_HUMAN	CORO1A*	Coronin, splice variant or isoform	419,936	221,607
Q9BR76	COR1B_HUMAN	CORO1B	Coronin-1B	7,397,315	18,902,425
P08185	CBG_HUMAN	SERPINA6	Corticosteroid-binding globulin	5,423,917	-
P12277	KCRB_HUMAN	CKB	Creatine kinase B-type	-	87,150,760
Q86VP6	CAND1_HUMAN	CAND1	Cullin-associated NEDD8-dissociated protein 1	-	82,675,700
P04080	CYTB_HUMAN	CSTB	Cystatin-B	-	17,446,050
P01034	CYTC_HUMAN	CST3	Cystatin-C	3,012,167	39,291,800
P00167	CYB5_HUMAN	CYB5A	Cytochrome b5	-	2,116,984
H3BRG4	H3BRG4_HUMAN	UQCRC2*	Cytochrome b-c1 complex subunit 2, mitochondrial	-	363,757
Q14204	DYHC1_HUMAN	DYNC1H1	Cytoplasmic dynein 1 heavy chain 1, splice variant or isoform	117,882,391	125,864,533
H0YJ21	H0YJ21_HUMAN	DYNC1H1*	Cytoplasmic dynein 1 heavy chain 1, splice variant or isoform	978,800	1,004,275
H0YNP0	H0YNP0_HUMAN	CYFIP1*	Cytoplasmic FMR1-interacting protein 1	-	2,236,458
P28838	AMPL_HUMAN	LAP3	Cytosol aminopeptidase, splice variant or isoform, splice variant or isoform	-	138,381,459
H0Y9Q1	H0Y9Q1_HUMAN	LAP3*	Cytosol aminopeptidase, splice variant or isoform, splice variant or isoform	-	1,135,634
O75891	AL1L1_HUMAN	ALDH1L1	Cytosolic 10-formyltetrahydrofolate dehydrogenase	-	22,815,795
O75891-2	AL1L1_HUMAN	ALDH1L1	Cytosolic 10-formyltetrahydrofolate dehydrogenase, Isoform 2	-	17,601,620
J3QKT2	J3QKT2_HUMAN	CNDP2*	Cytosolic non-specific dipeptidase	2,771,967	11,094,167
Q96KP4	CNDP2_HUMAN	CNDP2	Cytosolic non-specific dipeptidase, splice variant or isoform	-	168,445,794
P49902	5NTC_HUMAN	NT5C2	Cytosolic purine 5'-nucleotidase, splice variant or isoform, splice variant or isoform	-	747,027
H0YHR8	H0YHR8_HUMAN	NT5C2*	Cytosolic purine 5'-nucleotidase, splice variant or isoform, splice variant or isoform	-	181,244
O43175	SERA_HUMAN	PHGDH	D-3-phosphoglycerate dehydrogenase, splice variant or isoform, splice variant or isofo	-	66,648,141
Q5SZU1	Q5SZU1_HUMAN	PHGDH	D-3-phosphoglycerate dehydrogenase, splice variant or isoform, splice variant or isofo	-	63,797,341
Q96B18	DACT3_HUMAN	DACT3	Dapper homolog 3	17,421,250	58,138,700
Q13268	DHRS2_HUMAN	DHRS2	Dehydrogenase/reductase SDR family member 2, mitochondrial, splice variant or isofo	33,313,294	28,369,457
C9JZP6	C9JZP6_HUMAN	DHRS2	Dehydrogenase/reductase SDR family member 2, mitochondrial, splice variant or isofo	22,836,915	14,885,057
Q9NZW4	DSPP_HUMAN	DSPP*	Dentin sialophosphoprotein	331,140	13,226,500
B7Z4K6	B7Z4K6_HUMAN	DNASE2	Deoxyribonuclease-2-alpha	-	13,566,467
Q07507	DERM_HUMAN	DPT*	Dermatopontin	1,238,706	1,921,200
P81605	DCD_HUMAN	DCD	Dermcidin	-	9,112,439
P81605-2	DCD_HUMAN	DCD	Dermcidin, Isoform 2	-	1,025,039
Q02487	DSC2_HUMAN	DSC2	Desmocollin-2	1,126,278	2,880,925
F6RFD5	F6RFD5_HUMAN	DSTN	Destrin	-	11,295,367

Q14117	DPYS_HUMAN	DPYS*	Dihydropyrimidinase	1,089,492	1,715,400
Q12882	DPYD_HUMAN	DPYD*	Dihydropyrimidine dehydrogenase [NADP(+)]	10,460,950	27,248,500
P16444	DPEP1_HUMAN	DPEP1*	Dipeptidase 1	893,393	3,197,150
P53634	CATC_HUMAN	CTSC	Dipeptidyl peptidase 1	-	71,698,969
B7Z5C0	B7Z5C0_HUMAN	DNAJA1*	DnaJ homolog subfamily A member 1	-	641,567
O60884	DNJA2_HUMAN	DNAJA2*	DnaJ homolog subfamily A member 2	-	986,361
U3KQ84	U3KQ84_HUMAN	DDOST	Dolichyl-diphosphooligosaccharide--protein glycosyltransferase 48 kDa subunit, splice	2,580,575	6,885,950
E7EWT1	E7EWT1_HUMAN	DDOST*	Dolichyl-diphosphooligosaccharide--protein glycosyltransferase 48 kDa subunit, splice	1,119,050	1,792,450
B7Z4L4	B7Z4L4_HUMAN	RPN1*	Dolichyl-diphosphooligosaccharide--protein glycosyltransferase subunit 1	2,017,775	3,647,325
P04844	RPN2_HUMAN	RPN2	Dolichyl-diphosphooligosaccharide--protein glycosyltransferase subunit 2, splice varia	5,526,308	10,367,000
F2Z3K5	F2Z3K5_HUMAN	RPN2*	Dolichyl-diphosphooligosaccharide--protein glycosyltransferase subunit 2, splice varia	228,811	810,050
B4DDD6	B4DDD6_HUMAN	DBNL	Drebrin-like protein	747,000	811,300
P46734	MP2K3_HUMAN	MAP2K3	Dual specificity mitogen-activated protein kinase kinase 3	3,069,184	21,204,250
G8JLD5	G8JLD5_HUMAN	DNM1L	Dynamin-1-like protein	-	672,889
O00429-7	DNM1L_HUMAN	DNM1L	Dynamin-1-like protein, Isoform 7	-	8,672,189
F5H4R9	F5H4R9_HUMAN	DNM2	Dynamin-2	-	8,292,194
Q14258	TRI25_HUMAN	TRIM25*	E3 ubiquitin/ISG15 ligase TRIM25	1,826,950	6,746,150
Q5T4S7	UBR4_HUMAN	UBR4*	E3 ubiquitin-protein ligase UBR4	-	139,608
Q15075	EEA1_HUMAN	EEA1	Early endosome antigen 1	21,994,550	69,206,000
B5MBZ0	B5MBZ0_HUMAN	EML4	Echinoderm microtubule-associated protein-like 4	3,082,250	2,665,517
Q9H223	EHD4_HUMAN	EHD4	EH domain-containing protein 4	-	59,578,600
P68104	EF1A1_HUMAN	EEF1A1	Elongation factor 1-alpha 1	79,555,735	232,404,375
P24534	EF1B_HUMAN	EEF1B2	Elongation factor 1-beta	-	15,947,559
E9PRY8	E9PRY8_HUMAN	EEF1D	Elongation factor 1-delta	-	23,117,821
P29692-3	EF1D_HUMAN	EEF1D	Elongation factor 1-delta, Isoform 3	-	22,377,321
B4DTG2	B4DTG2_HUMAN	EEF1G	Elongation factor 1-gamma	8,531,986	7,697,503
P13639	EF2_HUMAN	EEF2	Elongation factor 2	99,297,240	126,625,545
B7ZC38	B7ZC38_HUMAN	SH3GLB2	Endophilin-B2, splice variant or isoform	3,237,984	3,297,854
G5E9J1	G5E9J1_HUMAN	SH3GLB2*	Endophilin-B2, splice variant or isoform	1,382,584	1,919,684
P14625	ENPL_HUMAN	HSP90B1	Endoplasmin	-	113,076,857
H7BXV5	H7BXV5_HUMAN	COL18A1	Endostatin	-	5,767,270
Q9UNN8	EPCR_HUMAN	PROCR*	Endothelial protein C receptor	4,785,350	3,687,100
P30084	ECHM_HUMAN	ECHS1	Enoyl-CoA hydratase, mitochondrial	10,173,494	9,325,900
P12724	ECP_HUMAN	RNASE3	Eosinophil cationic protein	571,899,839	-
P11678	PERE_HUMAN	EPX	Eosinophil peroxidase	180,864,070	-
B4DQV7	B4DQV7_HUMAN	NPC2*	Epididymal secretory protein E1	-	122,775
P58107	EPIPL_HUMAN	EPPK1	Epiplakin	-	76,189,957
O15083	ERC2_HUMAN	ERC2*	ERC protein 2	44,612	39,681
O75477	ERLN1_HUMAN	ERLIN1*	Erlin-1	-	930,813
P60842	IF4A1_HUMAN	EIF4A1	Eukaryotic initiation factor 4A-I	574,422	-
Q9NZE6	Q9NZE6_HUMAN	EIF4A2*	Eukaryotic initiation factor 4A-II	2,033,910	-
P15170	ERF3A_HUMAN	GSPT1	Eukaryotic peptide chain release factor GTP-binding subunit ERF3A, splice variant or is	-	9,078,975
H3BSV8	H3BSV8_HUMAN	GSPT1*	Eukaryotic peptide chain release factor GTP-binding subunit ERF3A, splice variant or is	-	2,625,750
Q8IYD1	ERF3B_HUMAN	GSPT2	Eukaryotic peptide chain release factor GTP-binding subunit ERF3B	-	8,418,050
D6RBD7	D6RBD7_HUMAN	EEF1E1*	Eukaryotic translation elongation factor 1 epsilon-1	2,255,133	1,365,642
P41091	IF2G_HUMAN	EIF2S3*	Eukaryotic translation initiation factor 2 subunit 3	2,844,000	8,315,750
F5H335	F5H335_HUMAN	EIF3A	Eukaryotic translation initiation factor 3 subunit A	-	20,243,473
P55884	EIF3B_HUMAN	EIF3B	Eukaryotic translation initiation factor 3 subunit B	-	2,316,988
H3BRV0	H3BRV0_HUMAN	EIF3C	Eukaryotic translation initiation factor 3 subunit C	-	5,654,623
B4DVY1	B4DVY1_HUMAN	EIF3D	Eukaryotic translation initiation factor 3 subunit D	-	16,668,586
P60228	EIF3E_HUMAN	EIF3E	Eukaryotic translation initiation factor 3 subunit E, splice variant or isoform	-	2,042,510
E5RII3	E5RII3_HUMAN	EIF3E	Eukaryotic translation initiation factor 3 subunit E, splice variant or isoform	-	347,010
E5RIP5	E5RIP5_HUMAN	EIF3E*	Eukaryotic translation initiation factor 3 subunit E, splice variant or isoform	-	52,598
B3KSH1	B3KSH1_HUMAN	EIF3F	Eukaryotic translation initiation factor 3 subunit F, splice variant or isoform	-	16,259,033
B4DEW9	B4DEW9_HUMAN	EIF3F	Eukaryotic translation initiation factor 3 subunit F, splice variant or isoform	-	5,885,783
Q13347	EIF3I_HUMAN	EIF3I	Eukaryotic translation initiation factor 3 subunit I	29,030,728	23,661,601
B0QY89	B0QY89_HUMAN	EIF3L*	Eukaryotic translation initiation factor 3 subunit L	-	315,680
B4E2Q4	B4E2Q4_HUMAN	EIF3M*	Eukaryotic translation initiation factor 3 subunit M	6,638,833	1,217,734
P55010	IF5_HUMAN	EIF5	Eukaryotic translation initiation factor 5	-	6,663,500
P63241	IF5A1_HUMAN	EIF5A	Eukaryotic translation initiation factor 5A-1, splice variant or isoform	-	6,375,945
I3L504	I3L504_HUMAN	EIF5A*	Eukaryotic translation initiation factor 5A-1, splice variant or isoform	-	353,445
Q6IS14	IF5AL_HUMAN	EIF5AL1	Eukaryotic translation initiation factor 5A-1-like	-	4,343,145
O60841	IF2P_HUMAN	EIF5B	Eukaryotic translation initiation factor 5B	-	22,002,929
O14980	XPO1_HUMAN	XPO1	Exportin-1	2,636,273	6,180,600
P08294	SODE_HUMAN	SOD3	Extracellular superoxide dismutase [Cu-Zn]	-	610,254,474
E7EQR4	E7EQR4_HUMAN	EZR	Ezrin	26,298,244	13,282,805
P49327	FAS_HUMAN	FASN	Fatty acid synthase	766,871,040	1,117,477,850
Q86UX7	URP2_HUMAN	FERMT3*	Fermitin family homolog 3	232,507	-
Q6ZNA5	FRRS1_HUMAN	FRRS1*	Ferric-chelate reductase 1	11,108,250	2,249,325
Q9UGM5	FETUB_HUMAN	FETUB	Fetuin-B	15,924,500	8,598,100
P35555	FBN1_HUMAN	FBN1*	Fibrillin-1	-	1,117,545
P02671	FIBA_HUMAN	FGA	Fibrinogen alpha chain	712,902,170	675,720,143
P02675	FIBB_HUMAN	FGB	Fibrinogen beta chain	1,363,608,548	918,843,617
P02679	FIBG_HUMAN	FGG	Fibrinogen gamma chain, splice variant or isoform	296,016,856	289,481,258
C9JC84	C9JC84_HUMAN	FGG	Fibrinogen gamma chain, splice variant or isoform	295,372,215	285,855,108
Q14314	FGL2_HUMAN	FGL2	Fibroleukin (fibrinogen-like 2)	-	994,572,330
P02751	FINC_HUMAN	FN1	Fibronectin	3,126,106	-
B1AHL2	B1AHL2_HUMAN	FBLN1*	Fibulin-1	37,639	73,732
Q15485	FCN2_HUMAN	FCN2	Ficolin-2	25,540,779	80,335,373
O75636	FCN3_HUMAN	FCN3	Ficolin-3	7,697,009	-
P30043	BLVRB_HUMAN	BLVRB	Flavin reductase (NADPH), splice variant or isoform	21,607,431	65,082,683
M0QZL1	M0QZL1_HUMAN	BLVRB*	Flavin reductase (NADPH), splice variant or isoform	329,431	504,183
Q6MZW2	FSTL4_HUMAN	FSTL4	Follistatin-related protein 4	-	3,459,316
Q8N475	FSTL5_HUMAN	FSTL5*	Follistatin-related protein 5	57,165,000	66,630,000
B7WPK3	B7WPK3_HUMAN	FTCD	Formimidoyltransferase-cyclodeaminase	-	5,549,188
O95954-2	FTCD_HUMAN	FTCD	Formimidoyltransferase-cyclodeaminase, Isoform C	-	8,341,138
O95954-4	FTCD_HUMAN	FTCD	Formimidoyltransferase-cyclodeaminase, Isoform E	-	2,018,000

Q5T7D5	Q5T7D5_HUMAN	ALDOB	Fructose-bisphosphate aldolase	-	945,957
P05062	ALDOB_HUMAN	ALDOB	Fructose-bisphosphate aldolase B	-	4,936,807
Q08380	LG3BP_HUMAN	LGALS3BP	Galectin-3-binding protein, splice variant or isoform	-	8,290,545
K7EKQ5	K7EKQ5_HUMAN	LGALS3BP	Galectin-3-binding protein, splice variant or isoform	-	1,330,545
K7EJD3	K7EJD3_HUMAN	LGALS3BP	Galectin-3-binding protein, splice variant or isoform	-	794,145
Q92820	GGH_HUMAN	GGH	Gamma-glutamyl hydrolase	-	42,476,008
E9PG40	E9PG40_HUMAN	APP	Gamma-secretase C-terminal fragment 59	3,904,389	76,087,058
P17900	SAP3_HUMAN	GM2A	Ganglioside GM2 activator	-	5,614,636
E9PP60	E9PP60_HUMAN	TSTA3*	GDP-L-fucose synthase	-	550,500
P06396	GELS_HUMAN	GSN	Gelsolin	71,752,957	50,768,075
F5GYR8	F5GYR8_HUMAN	USO1	General vesicular transport factor p115	-	1,570,999
M0QX47	M0QX47_HUMAN	GMFG*	Glia maturation factor gamma	152,215	-
P14136	GFAP_HUMAN	GFAP*	Glial fibrillary acidic protein	59,855,000	18,654,667
Q86VQ1	GLCI1_HUMAN	GLCCI1*	Glucocorticoid-induced transcript 1 protein	2,255,950	14,175,000
P11413	G6PD_HUMAN	G6PD	Glucose-6-phosphate 1-dehydrogenase	7,376,570	-
K7EQ48	K7EQ48_HUMAN	GPI	Glucose-6-phosphate isomerase	4,206,042	14,104,608
K7ELL7	K7ELL7_HUMAN	PRKCSH	Glucosidase 2 subunit beta	-	5,502,267
P48506	GSH1_HUMAN	GCLC*	Glutamate--cysteine ligase catalytic subunit	6,653,250	23,913,000
P48507	GSH0_HUMAN	GCLM*	Glutamate--cysteine ligase regulatory subunit	-	6,627,700
Q06210	GFPT1_HUMAN	GFPT1	Glutamine--fructose-6-phosphate aminotransferase [isomerizing] 1	-	2,066,575
P47897	SYQ_HUMAN	QARS	Glutamine--tRNA ligase	-	19,337,895
P07203	GPX1_HUMAN	GPX1	Glutathione peroxidase 1	-	34,643,450
P00390	GSHR_HUMAN	GSR	Glutathione reductase, mitochondrial	2,669,610	4,831,690
P08263	GSTA1_HUMAN	GSTA1	Glutathione S-transferase A1	-	44,744,492
Q16772	GSTA3_HUMAN	GSTA3	Glutathione S-transferase A3	-	24,778,317
P21266	GSTM3_HUMAN	GSTM3	Glutathione S-transferase Mu 3	-	21,152,050
P09211	GSTP1_HUMAN	GSTP1	Glutathione S-transferase P, splice variant or isoform	33,538,534	37,545,600
A8MX94	A8MX94_HUMAN	GSTP1	Glutathione S-transferase P, splice variant or isoform	20,126,734	21,491,550
P04406-2	G3P_HUMAN	GAPDH	Glyceraldehyde-3-phosphate dehydrogenase, Isoform 2	53,995,487	88,278,744
P04406	G3P_HUMAN	GAPDH	Glyceraldehyde-3-phosphate dehydrogenase, splice variant or isoform	93,027,837	124,512,144
E7EUT5	E7EUT5_HUMAN	GAPDH	Glyceraldehyde-3-phosphate dehydrogenase, splice variant or isoform	19,215,328	49,897,002
O14556	G3PT_HUMAN	GAPDHS	Glyceraldehyde-3-phosphate dehydrogenase, testis-specific	-	8,359,099
Q7L5L3	GDPD3_HUMAN	GDPD3	Glycerophosphodiester phosphodiesterase domain-containing protein 3	6,220,650	6,386,700
P11216	PYGB_HUMAN	PYGB*	Glycogen phosphorylase, brain form	1,443,700	1,039,576
P06737	PYGL_HUMAN	PYGL*	Glycogen phosphorylase, liver form	277,385	330,419
Q9UBQ7	GRHPR_HUMAN	GRHPR	Glyoxylate reductase/hydroxypyruvate reductase	5,218,374	6,284,275
P51654	GPC3_HUMAN	GPC3	Glypican-3	-	8,579,075
O75487	GPC4_HUMAN	GPC4	Glypican-4	-	30,575,143
P28799	GRN_HUMAN	GRN	Granulins	4,934,525	-
P28799-3	GRN_HUMAN	GRN*	Granulins, Isoform 3	666,750	587,117
J3KP07	J3KP07_HUMAN	GAS6	Growth arrest-specific protein 6	-	20,270,159
Q99988	GDF15_HUMAN	GDF15	Growth/differentiation factor 15	-	3,149,650
B5MDF5	B5MDF5_HUMAN	RAN	GTP-binding nuclear protein Ran	13,387,001	32,194,834
P63096	GNAI1_HUMAN	GNAI1	Guanine nucleotide-binding protein G(i) subunit alpha-1	-	3,692,228
P63096-2	GNAI1_HUMAN	GNAI1	Guanine nucleotide-binding protein G(i) subunit alpha-1, Isoform 2	-	699,447
P04899	GNAI2_HUMAN	GNAI2	Guanine nucleotide-binding protein G(i) subunit alpha-2	-	3,961,995
P04899-2	GNAI2_HUMAN	GNAI2	Guanine nucleotide-binding protein G(i) subunit alpha-2, Isoform 2	-	1,013,620
P04899-4	GNAI2_HUMAN	GNAI2	Guanine nucleotide-binding protein G(i) subunit alpha-2, Isoform sGi2	-	3,569,872
P62873-2	GBB1_HUMAN	GNB1	Guanine nucleotide-binding protein G(I)/G(S)/G(T) subunit beta-1, Isoform 2	-	6,650,666
F6UT28	F6UT28_HUMAN	GNB1*	Guanine nucleotide-binding protein G(I)/G(S)/G(T) subunit beta-1, splice variant or isc	146,885	189,937
P62873	GBB1_HUMAN	GNB1	Guanine nucleotide-binding protein G(I)/G(S)/G(T) subunit beta-1, splice variant or isc	-	7,042,416
E7EP32	E7EP32_HUMAN	GNB2	Guanine nucleotide-binding protein G(I)/G(S)/G(T) subunit beta-2, splice variant or isc	41,436,341	12,249,350
B3KPU1	B3KPU1_HUMAN	GNB2	Guanine nucleotide-binding protein G(I)/G(S)/G(T) subunit beta-2, splice variant or isc	3,298,916	4,334,400
C9JIS1	C9JIS1_HUMAN	GNB2*	Guanine nucleotide-binding protein G(I)/G(S)/G(T) subunit beta-2, splice variant or isc	2,129,080	3,575,300
P63244	GBLP_HUMAN	GNB2L1	Guanine nucleotide-binding protein subunit beta-2-like 1, splice variant or isoform	20,360,549	41,207,224
J3KPE3	J3KPE3_HUMAN	GNB2L1	Guanine nucleotide-binding protein subunit beta-2-like 1, splice variant or isoform	7,953,724	13,524,274
H0Y8W2	H0Y8W2_HUMAN	GNB2L1	Guanine nucleotide-binding protein subunit beta-2-like 1, splice variant or isoform	4,575,224	3,240,024
D6RAC2	D6RAC2_HUMAN	GNB2L1	Guanine nucleotide-binding protein subunit beta-2-like 1, splice variant or isoform	3,506,286	10,498,086
P36915	GNL1_HUMAN	GNL1	Guanine nucleotide-binding protein-like 1	5,944,232	5,993,000
P00738	HPT_HUMAN	HP	Haptoglobin, splice variant or isoform	19,388,844	23,565,353
H0Y300	H0Y300_HUMAN	HP	Haptoglobin, splice variant or isoform	18,767,377	21,672,953
P00739	HPTR_HUMAN	HPR	Haptoglobin-related protein	41,866,615	64,268,690
P34932	HSP74_HUMAN	HSPA4	Heat shock 70 kDa protein 4	-	2,417,150
P11142-2	HSP7C_HUMAN	HSPA8	Heat shock cognate 71 kDa protein, Isoform 2	806,648	2,173,259
E9PNE6	E9PNE6_HUMAN	HSPA8	Heat shock cognate 71 kDa protein, splice variant or isoform	4,804,265	7,252,408
E9PN89	E9PN89_HUMAN	HSPA8*	Heat shock cognate 71 kDa protein, splice variant or isoform	356,806	682,883
R4GN69	R4GN69_HUMAN	HSPH1*	Heat shock protein 105 kDa	-	1,096,575
Q92598-3	HS105_HUMAN	HSPH1	Heat shock protein 105 kDa, Isoform 3	-	1,600,125
Q92598-2	HS105_HUMAN	HSPH1	Heat shock protein 105 kDa, Isoform Beta	-	8,596,575
P04792	HSPB1_HUMAN	HSPB1	Heat shock protein beta-1, splice variant or isoform	98,289,678	1,016,815,620
F8WE04	F8WE04_HUMAN	HSPB1	Heat shock protein beta-1, splice variant or isoform	4,668,329	1,105,810
P07900	HS90A_HUMAN	HSP90AA1	Heat shock protein HSP 90-alpha	171,164,571	203,703,408
P08238	HS90B_HUMAN	HSP90AB1	Heat shock protein HSP 90-beta	222,520,643	752,553,199
P69905	HBA_HUMAN	HBA1	Hemoglobin subunit alpha	4,877,618,644	4,395,413,540
P68871	HBB_HUMAN	HBB	Hemoglobin subunit beta	10,279,232,817	7,655,027,420
P02042	HBD_HUMAN	HBD	Hemoglobin subunit delta, splice variant or isoform	2,794,864,742	1,591,882,220
E9PEW8	E9PEW8_HUMAN	HBD*	Hemoglobin subunit delta, splice variant or isoform	974,565	10,083,700
P02100	HBE_HUMAN	HBE1*	Hemoglobin subunit epsilon	-	16,401,500
P69891	HBG1_HUMAN	HBG1	Hemoglobin subunit gamma-1	5,547,367	62,307,467
E9PBW4	E9PBW4_HUMAN	HBG2	Hemoglobin subunit gamma-2	-	9,263,217
P02790	HEMO_HUMAN	HPX	Hemopexin	181,535,625	102,568,190
P05546	HEP2_HUMAN	SERPIND1	Heparin cofactor 2	754,133,247	585,635,653
Q13151	ROA0_HUMAN	HNRNPA0*	Heterogeneous nuclear ribonucleoprotein A0	-	7,812,500
F8W6I7	F8W6I7_HUMAN	HNRNPA1	Heterogeneous nuclear ribonucleoprotein A1, splice variant or isoform	3,114,734	2,750,790
F8W646	F8W646_HUMAN	HNRNPA1	Heterogeneous nuclear ribonucleoprotein A1, splice variant or isoform	2,820,124	2,382,590
F8VTQ5	F8VTQ5_HUMAN	HNRNPA1	Heterogeneous nuclear ribonucleoprotein A1, splice variant or isoform	631,100	822,844
F8VZ49	F8VZ49_HUMAN	HNRNPA1*	Heterogeneous nuclear ribonucleoprotein A1, splice variant or isoform	113,414	212,276

B4DTC3	B4DTC3_HUMAN	HNRNPD*	Heterogeneous nuclear ribonucleoprotein D0	116,189	61,109
P52597	HNRPF_HUMAN	HNRNPF*	Heterogeneous nuclear ribonucleoprotein F	-	387,578
E9PCY7	E9PCY7_HUMAN	HNRNPH1	Heterogeneous nuclear ribonucleoprotein H	-	1,394,108
P61978	HNRPK_HUMAN	HNRNPK	Heterogeneous nuclear ribonucleoprotein K	4,081,668	5,311,125
P14866	HNRPL_HUMAN	HNRNPL*	Heterogeneous nuclear ribonucleoprotein L	-	277,570
P52272	HNRPM_HUMAN	HNRNPM*	Heterogeneous nuclear ribonucleoprotein M	338,800	584,100
B4DT28	B4DT28_HUMAN	HNRNPR*	Heterogeneous nuclear ribonucleoprotein R	2,035,667	2,337,167
P22626	ROA2_HUMAN	HNRNPA2B1	Heterogeneous nuclear ribonucleoproteins A2/B1	26,116,327	24,881,164
B4DY08	B4DY08_HUMAN	HNRNPC	Heterogeneous nuclear ribonucleoproteins C1/C2	-	4,364,884
E9PB90	E9PB90_HUMAN	HK2	Hexokinase-2	9,862,375	21,632,175
P52790	HXK3_HUMAN	HK3*	Hexokinase-3	1,921,150	4,527,450
P09429	HMGB1_HUMAN	HMGB1	High mobility group protein B1	-	16,520,717
P26583	HMGB2_HUMAN	HMGB2*	High mobility group protein B2	535,836	3,114,300
P04196	HRG_HUMAN	HRG	Histidine-rich glycoprotein	175,890,832	984,961,871
H0YFX9	H0YFX9_HUMAN	H2AFJ	Histone H2A	645,388	1,735,546
Q6FI13	H2A2A_HUMAN	HIST2H2AA3*	Histone H2A type 2-A	8,181,000	2,064,300
Q9BTM1	H2AJ_HUMAN	H2AFJ	Histone H2A.J	3,316,847	3,678,123
Q96A08	H2B1A_HUMAN	HIST1H2BA*	Histone H2B type 1-A	1,557,972	703,222
P33778	H2B1B_HUMAN	HIST1H2BB	Histone H2B type 1-B	3,146,466	2,189,711
P57053	H2BFS_HUMAN	H2BFS	Histone H2B type F-S	5,296,237	5,207,561
P62805	H4_HUMAN	HIST1H4A	Histone H4	306,801,206	167,931,675
P30447	1A23_HUMAN	HLA-A	HLA class I histocompatibility antigen, A-23 alpha chain	-	54,375,225
P05534	1A24_HUMAN	HLA-A	HLA class I histocompatibility antigen, A-24 alpha chain	-	34,874,525
P04233	HG2A_HUMAN	CD74*	HLA class II histocompatibility antigen gamma chain	183,332	559,300
B7Z5N8	B7Z5N8_HUMAN	HOXB3*	Homeobox protein Hox-B3	208,362	1,051,350
O95343	SIX3_HUMAN	SIX3*	Homeobox protein SIX3	2,361,750	1,660,900
P50502	F10A1_HUMAN	ST13	Hsc70-interacting protein	-	1,044,355
Q14520	HABP2_HUMAN	HABP2	Hyaluronan-binding protein 2	-	15,569,667
B7Z8Q5	B7Z8Q5_HUMAN	HABP2	Hyaluronan-binding protein 2 50 kDa heavy chain	-	2,294,667
P54868	HMCS2_HUMAN	HMGCS2	Hydroxymethylglutaryl-CoA synthase, mitochondrial	-	2,331,200
B7Z909	B7Z909_HUMAN	HYOU1*	Hypoxia up-regulated protein 1	625,025	516,375
P01876	IGHA1_HUMAN	IGHA1	Ig alpha-1 chain C region	69,159,533	24,583,105
P01857	IGHG1_HUMAN	IGHG1	Ig gamma-1 chain C region	215,375,027	146,063,010
P01859	IGHG2_HUMAN	IGHG2	Ig gamma-2 chain C region	174,828,035	112,225,434
P01860	IGHG3_HUMAN	IGHG3	Ig gamma-3 chain C region	89,060,164	40,771,309
P01861	IGHG4_HUMAN	IGHG4	Ig gamma-4 chain C region	154,582,315	43,361,334
P01743	HV102_HUMAN		Ig heavy chain V-I region HG3	7,258,750	7,291,900
P06331	HV209_HUMAN	*	Ig heavy chain V-II region ARH-77	226,694	713,014
P01766	HV305_HUMAN	*	Ig heavy chain V-III region BRO	3,184,784	-
P01834	IGKC_HUMAN	IGKC	Ig kappa chain C region	110,995,668	194,049,644
P01593	KV101_HUMAN	*	Ig kappa chain V-I region AG	47,176	141,606
P01605	KV113_HUMAN	*	Ig kappa chain V-I region Lay	5,919,875	6,629,750
P01614	KV201_HUMAN		Ig kappa chain V-II region Cum	4,476,268	3,555,538
P01616	KV203_HUMAN		Ig kappa chain V-II region MIL	6,294,900	-
P06310	KV206_HUMAN	*	Ig kappa chain V-II region RPMI 6410	200,643	406,088
P04206	KV307_HUMAN		Ig kappa chain V-III region GOL	444,290	-
P18135	KV312_HUMAN		Ig kappa chain V-III region HAH	6,262,853	-
P06314	KV404_HUMAN		Ig kappa chain V-IV region B17	14,950,375	5,326,263
P80748	LV302_HUMAN	*	Ig lambda chain V-III region LOI	5,107,300	6,557,550
P0CG04	LAC1_HUMAN	IGLC1	Ig lambda-1 chain C regions	1,670,618	4,124,363
P0CG05	LAC2_HUMAN	IGLC2	Ig lambda-2 chain C regions	11,870,090	11,942,286
B9A064	IGLL5_HUMAN	IGLL5	Immunoglobulin lambda-like polypeptide 5	12,948,355	11,360,634
Q969P0	IGSF8_HUMAN	IGSF8	Immunoglobulin superfamily member 8	6,562,275	5,876,239
Q14974	IMB1_HUMAN	KPNB1*	Importin subunit beta-1	440,563	1,747,150
P12268	IMDH2_HUMAN	IMPDH2	Inosine-5'-monophosphate dehydrogenase 2, splice variant or isoform	-	36,320,226
E7ETK5	E7ETK5_HUMAN	IMPDH2	Inosine-5'-monophosphate dehydrogenase 2, splice variant or isoform	-	14,354,401
P35858	ALS_HUMAN	IGFALS	Insulin-like growth factor-binding protein complex acid labile subunit	-	7,315,725
Q9Y287	ITM2B_HUMAN	ITM2B*	Integral membrane protein 2B	-	1,709,100
P11215	ITAM_HUMAN	ITGAM*	Integrin alpha-M	6,870,250	-
A8MYE6	A8MYE6_HUMAN	ITGB2	Integrin beta	2,725,165	2,901,261
E9PM54	E9PM54_HUMAN	ILK*	Integrin-linked protein kinase	239,938	787,625
P19827	ITIH1_HUMAN	ITIH1	Inter-alpha-trypsin inhibitor heavy chain H1	101,497,889	97,204,141
P19823	ITIH2_HUMAN	ITIH2	Inter-alpha-trypsin inhibitor heavy chain H2, splice variant or isoform	45,005,838	234,570,258
Q5T987	Q5T987_HUMAN	ITIH2	Inter-alpha-trypsin inhibitor heavy chain H2, splice variant or isoform	17,268,067	11,748,267
Q14624	ITIH4_HUMAN	ITIH4	Inter-alpha-trypsin inhibitor heavy chain H4	10,550,409	10,549,702
Q14624-4	ITIH4_HUMAN	ITIH4	Inter-alpha-trypsin inhibitor heavy chain H4, Isoform 4	1,713,632	2,337,445
B4DFL2	B4DFL2_HUMAN	IDH2*	Isocitrate dehydrogenase [NADP]	304,321	467,975
P41252	SYIC_HUMAN	IARS	Isoleucine--tRNA ligase, cytoplasmic, splice variant or isoform	-	11,108,950
J3KR24	J3KR24_HUMAN	IARS	Isoleucine--tRNA ligase, cytoplasmic, splice variant or isoform	-	7,076,800
Q6H9L7-3	ISM2_HUMAN	ISM2*	Isthmin-2, Isoform 3	145,073	854,463
H3BVG3	H3BVG3_HUMAN	JMJD8*	JmjC domain-containing protein 8	600,475	1,275,600
P14923	PLAK_HUMAN	JUP	Junction plakoglobin	-	43,579,600
E7EQB2	E7EQB2_HUMAN	LTF	Kaliocin-1, splice variant or isoform	101,259,248	97,000,760
E7ER44	E7ER44_HUMAN	LTF	Kaliocin-1, splice variant or isoform	100,478,173	94,721,985
P29622	KAIN_HUMAN	SERPINA4	Kallistatin	30,711,793	-
Q15323	K1H1_HUMAN	KRT31	Keratin, type I cuticular Ha1	1,223,831	2,508,606
P13645	K1C10_HUMAN	KRT10	Keratin, type I cytoskeletal 10	1,584,625,082	1,611,373,194
P13646-3	K1C13_HUMAN	KRT13	Keratin, type I cytoskeletal 13, Isoform 3	1,051,988,703	164,458,954
P02533	K1C14_HUMAN	KRT14	Keratin, type I cytoskeletal 14	117,385,387	144,483,061
P19012	K1C15_HUMAN	KRT15*	Keratin, type I cytoskeletal 15	6,573,400	6,114,700
P08779	K1C16_HUMAN	KRT16	Keratin, type I cytoskeletal 16	94,237,332	141,757,808
Q04695	K1C17_HUMAN	KRT17	Keratin, type I cytoskeletal 17	66,472,064	108,415,284
F8VZY9	F8VZY9_HUMAN	KRT18	Keratin, type I cytoskeletal 18	190,252,125	220,035,841
P08727	K1C19_HUMAN	KRT19	Keratin, type I cytoskeletal 19	193,810,196	526,282,667
P35527	K1C9_HUMAN	KRT9	Keratin, type I cytoskeletal 9	815,618,271	760,301,079
Q14533	KRT81_HUMAN	KRT81*	Keratin, type II cuticular Hb1	346,180	477,490
Q9NSB2	KRT84_HUMAN	KRT84*	Keratin, type II cuticular Hb4	3,983,700	15,085,500

F5GYI5	F5GYI5_HUMAN	KRT85	Keratin, type II cuticular Hb5	1,205,755	1,252,440
P04264	K2C1_HUMAN	KRT1	Keratin, type II cytoskeletal 1	981,139,437	1,245,411,110
Q7Z794	K2C1B_HUMAN	KRT77*	Keratin, type II cytoskeletal 1b	2,864,200	1,150,613
P35908	K22E_HUMAN	KRT2	Keratin, type II cytoskeletal 2 epidermal	242,531,083	261,923,179
J3QST3	J3QST3_HUMAN	KRT3*	Keratin, type II cytoskeletal 3	247,949	305,183
P13647	K2C5_HUMAN	KRT5	Keratin, type II cytoskeletal 5	115,340,397	135,826,234
P02538	K2C6A_HUMAN	KRT6A	Keratin, type II cytoskeletal 6A	41,244,689	48,186,507
P04259	K2C6B_HUMAN	KRT6B	Keratin, type II cytoskeletal 6B	6,881,542	13,327,695
P48668	K2C6C_HUMAN	KRT6C	Keratin, type II cytoskeletal 6C	103,413,217	117,290,450
P08729	K2C7_HUMAN	KRT7	Keratin, type II cytoskeletal 7	239,811,805	419,160,810
P05787-2	K2C8_HUMAN	KRT8	Keratin, type II cytoskeletal 8, Isoform 2	56,197,372	147,905,143
Q6KB66-2	K2C80_HUMAN	KRT80*	Keratin, type II cytoskeletal 80, Isoform 2	6,438,667	5,938,667
P50053-2	KHK_HUMAN	KHK*	Ketohexokinase, Isoform C	14,123,000	5,420,500
P33176	KINH_HUMAN	KIF5B	Kinesin-1 heavy chain	28,509,900	25,709,450
Q9NQT8	KI13B_HUMAN	KIF13B	Kinesin-like protein KIF13B	-	11,852,050
Q02241-2	KIF23_HUMAN	KIF23*	Kinesin-like protein KIF23, Isoform 2	422,713	64,715,000
P01042-3	KNG1_HUMAN	KNG1	Kininogen-1, Isoform 3	53,442,082	99,682,538
P01042-2	KNG1_HUMAN	KNG1	Kininogen-1, Isoform LMW	201,175,051	805,216,669
P02788-2	TRFL_HUMAN	LTF	Lactotransferrin, Isoform DeltaLf	1,331,703	2,044,650
Q9Y2S2-2	CRYL1_HUMAN	CRYL1	Lambda-crystallin homolog, Isoform 2	-	6,926,896
Q16787-3	LAMA3_HUMAN	LAMA3	Laminin subunit alpha-3, Isoform 3	2,440,218	39,992,350
P11047	LAMC1_HUMAN	LAMC1	Laminin subunit gamma-1	-	45,125,350
Q8ND23	LR16B_HUMAN	LRRC16B	Leucine-rich repeat-containing protein 16B	25,238,000	9,932,950
Q8N1G4	LRC47_HUMAN	LRRC47	Leucine-rich repeat-containing protein 47	-	24,680,250
B4DER1	B4DER1_HUMAN	LARS	Leucine--tRNA ligase, cytoplasmic	-	3,115,263
P30740	ILEU_HUMAN	SERPINB1	Leukocyte elastase inhibitor	355,136,751	-
P09960-2	LKHA4_HUMAN	LTA4H	Leukotriene A-4 hydrolase, Isoform 2	11,210,619	-
Q9UHB6-4	LIMA1_HUMAN	LIMA1	LIM domain and actin-binding protein 1, Isoform 4	1,079,300	2,248,960
O75112	LDB3_HUMAN	LDB3	LIM domain-binding protein 3	120,280,500	119,233,500
Q9P260-2	K1468_HUMAN	KIAA1468	LisH domain and HEAT repeat-containing protein KIAA1468, Isoform 2	1,016,603	4,902,975
A8MW50	A8MW50_HUMAN	LDHB	L-lactate dehydrogenase	-	2,601,850
F5H5J4	F5H5J4_HUMAN	LDHA*	L-lactate dehydrogenase A chain	103,746	122,640
P00338-2	LDHA_HUMAN	LDHA	L-lactate dehydrogenase A chain, Isoform 2	3,791,246	957,828
P00338-3	LDHA_HUMAN	LDHA	L-lactate dehydrogenase A chain, Isoform 3	6,847,829	2,760,528
P00338-4	LDHA_HUMAN	LDHA	L-lactate dehydrogenase A chain, Isoform 4	3,160,329	1,925,340
P07195	LDHB_HUMAN	LDHB	L-lactate dehydrogenase B chain, splice variant or isoform	-	5,651,041
C9J7H8	C9J7H8_HUMAN	LDHB	L-lactate dehydrogenase B chain, splice variant or isoform	-	751,459
P98164	LRP2_HUMAN	LRP2	Low-density lipoprotein receptor-related protein 2	-	49,026,500
E9PFL9	E9PFL9_HUMAN	SSB	Lupus La protein, splice variant or isoform	-	5,527,248
E9PGX9	E9PGX9_HUMAN	SSB*	Lupus La protein, splice variant or isoform	-	398,898
P01229	LSHB_HUMAN	LHB*	Lutropin subunit beta	-	1,136,767
Q7Z4W1	DCXR_HUMAN	DCXR*	L-xylulose reductase	328,776	331,797
Q14210	LY6D_HUMAN	LY6D*	Lymphocyte antigen 6D	258,172	706,444
P10253	LYAG_HUMAN	GAA	Lysosomal alpha-glucosidase	8,718,278	5,027,998
P61626	LYSC_HUMAN	LYZ	Lysozyme C, splice variant or isoform	46,102,725	-
F8VV32	F8VV32_HUMAN	LYZ	Lysozyme C, splice variant or isoform	14,059,400	-
P14174	MIF_HUMAN	MIF*	Macrophage migration inhibitory factor	12,481,500	13,562,000
Q14764	MVP_HUMAN	MVP	Major vault protein	-	242,748,036
P40925-2	MDHC_HUMAN	MDH1	Malate dehydrogenase, cytoplasmic, Isoform 2	1,398,120	3,609,286
F5GX14	F5GX14_HUMAN	MLEC	Malectin, splice variant or isoform	-	1,244,500
H0YG07	H0YG07_HUMAN	MLEC*	Malectin, splice variant or isoform	-	703,950
Q9UM22	EPDR1_HUMAN	EPDR1	Mammalian ependymin-related protein 1	6,882,625	6,674,800
O00187	MASP2_HUMAN	MASP2	Mannan-binding lectin serine protease 2	263,885,775	1,084,817,779
O00187-2	MASP2_HUMAN	MASP2	Mannan-binding lectin serine protease 2, Isoform 2	262,441,025	1,077,947,279
P33908	MA1A1_HUMAN	MAN1A1	Mannosyl-oligosaccharide 1,2-alpha-mannosidase IA	251,874,810	376,572,355
P14780	MMP9_HUMAN	MMP9	Matrix metalloproteinase-9	1,811,973	3,178,400
Q9BRK3-2	MXRA8_HUMAN	MXRA8	Matrix-remodeling-associated protein 8, Isoform 2	88,274,691	164,373,304
Q9BRK3-4	MXRA8_HUMAN	MXRA8	Matrix-remodeling-associated protein 8, Isoform 4	27,312,263	37,849,250
Q96HR3-2	MED30_HUMAN	MED30*	Mediator of RNA polymerase II transcription subunit 30, Isoform 2	-	2,601,400
P08582-2	TRFM_HUMAN	MFI2	Melanotransferrin, Isoform 2	19,255,968	9,631,623
P08582	TRFM_HUMAN	MFI2	Melanotransferrin, splice variant or isoform	37,346,060	39,215,773
C9JVN1	C9JVN1_HUMAN	MFI2*	Melanotransferrin, splice variant or isoform	368,876	472,717
A6NC17	A6NC17_HUMAN	MARS	Methionine--tRNA ligase, cytoplasmic	549,875	1,550,207
E9PJF4	E9PJF4_HUMAN	CLNS1A	Methylosome subunit pICln	910,301	3,812,513
P55083-2	MFAP4_HUMAN	MFAP4	Microfibril-associated glycoprotein 4, Isoform 2	-	1,115,617
B3KR41	B3KR41_HUMAN	MAD1L1	Mitotic spindle assembly checkpoint protein MAD1	35,329,417	-
P26038	MOES_HUMAN	MSN	Moesin	40,829,584	34,362,083
B5ME49	B5ME49_HUMAN	MUC16	Mucin-16	35,407,250	15,756,500
Q9H8L6	MMRN2_HUMAN	MMRN2*	Multimerin-2	7,709,050	5,493,250
P24158	PRTN3_HUMAN	PRTN3	Myeloblastin	101,866,883	-
P41218	MNDA_HUMAN	MNDA	Myeloid cell nuclear differentiation antigen	2,636,393	-
P05164-2	PERM_HUMAN	MPO	Myeloperoxidase, Isoform H14	7,457,757	-
P60660-2	MYL6_HUMAN	MYL6	Myosin light polypeptide 6, Isoform Smooth muscle	138,275,499	-
G3V1V0	G3V1V0_HUMAN	MYL6	Myosin light polypeptide 6, splice variant or isoform	139,935,599	-
B7Z6Z4	B7Z6Z4_HUMAN	MYL6	Myosin light polypeptide 6, splice variant or isoform	129,065,399	-
H0YI43	H0YI43_HUMAN	MYL6	Myosin light polypeptide 6, splice variant or isoform	2,474,948	18,217,000
F8VPF3	F8VPF3_HUMAN	MYL6	Myosin light polypeptide 6, splice variant or isoform	1,390,904	-
J3QRS3	J3QRS3_HUMAN	MYL12A	Myosin regulatory light chain 12A	-	1,779,417
P35580-2	MYH10_HUMAN	MYH10	Myosin-10, Isoform 2	3,729,207	10,318,544
Q7Z406-2	MYH14_HUMAN	MYH14	Myosin-14, Isoform 2	-	57,996,683
P35579	MYH9_HUMAN	MYH9	Myosin-9	419,928,421	1,040,480,443
P35579-2	MYH9_HUMAN	MYH9	Myosin-9, Isoform 2	11,640,513	43,000,250
O94760-2	DDAH1_HUMAN	DDAH1*	N(G),N(G)-dimethylarginine dimethylaminohydrolase 1, Isoform 2	2,177,163	11,700,500
O95865	DDAH2_HUMAN	DDAH2	N(G),N(G)-dimethylarginine dimethylaminohydrolase 2, splice variant or isoform	42,165,290	68,806,860
H0Y7N1	H0Y7N1_HUMAN	DDAH2	N(G),N(G)-dimethylarginine dimethylaminohydrolase 2, splice variant or isoform	8,586,783	17,942,863
Q9UJ70-2	NAGK_HUMAN	NAGK	N-acetyl-D-glucosamine kinase, Isoform 2	-	3,589,707
H7C3G9	H7C3G9_HUMAN	NAGK	N-acetyl-D-glucosamine kinase, splice variant or isoform	4,785,084	3,151,208

C9JEV6	C9JEV6_HUMAN	NAGK	N-acetyl-D-glucosamine kinase, splice variant or isoform	818,080	2,501,190
H7C286	H7C286_HUMAN	NAGK*	N-acetyl-D-glucosamine kinase, splice variant or isoform	490,459	221,333
H7C1L7	H7C1L7_HUMAN	NAGK	N-acetyl-D-glucosamine kinase, splice variant or isoform	-	5,209,732
H0YE82	H0YE82_HUMAN	NAGK*	N-acetyl-D-glucosamine kinase, splice variant or isoform	-	2,279,857
Q8N159	NAGS_HUMAN	NAGS	N-acetylglutamate synthase, mitochondrial	48,337,950	88,246,000
O96009	NAPSA_HUMAN	NAPSA	Napsin-A	-	127,901,350
E9PAV3-2	NACAM_HUMAN	NACA	Nascent polypeptide-associated complex subunit alpha, muscle-specific form, Isoform	-	1,724,788
Q92692-2	PVRL2_HUMAN	PVRL2*	Nectin-2, Isoform Alpha	1,352,567	-
Q96JN8-2	NEUL4_HUMAN	NEURL4	Neuralized-like protein 4, Isoform 2	11,239,135	3,568,681
Q6ZNJ1-2	NBEL2_HUMAN	NBEAL2*	Neurobeachin-like protein 2, Isoform 2	457,975	1,455,850
Q14697-2	GANAB_HUMAN	GANAB	Neutral alpha-glucosidase AB, Isoform 2	-	15,292,894
P59665	DEF1_HUMAN	DEFA1	Neutrophil defensin 1 (Defensin, Alpha 1)	6,081,323,925	616,932,475
P12838	DEF4_HUMAN	DEFA4	Neutrophil defensin 4	13,719,083	-
P08246	ELNE_HUMAN	ELANE	Neutrophil elastase	240,815,768	-
H9KV70	H9KV70_HUMAN	LCN2	Neutrophil gelatinase-associated lipocalin	2,795,023	-
P80188-2	NGAL_HUMAN	LCN2	Neutrophil gelatinase-associated lipocalin, Isoform 2	471,018	-
Q96TA1-2	NIBL1_HUMAN	FAM129B	Niban-like protein 1, Isoform 2	-	3,000,450
P43490	NAMPT_HUMAN	NAMPT	Nicotinamide phosphoribosyltransferase	-	2,930,765
P14543-2	NID1_HUMAN	NID1	Nidogen-1, Isoform 2	32,459,580	80,785,529
Q15233-2	NONO_HUMAN	NONO*	Non-POU domain-containing octamer-binding protein, Isoform 2	176,146	285,575
P10153	RNAS2_HUMAN	RNASE2	Non-secretory ribonuclease	19,612,200	99,546,750
P61970	NTF2_HUMAN	NUTF2	Nuclear transport factor 2	45,167,126	143,823,764
Q02818	NUCB1_HUMAN	NUCB1	Nucleobindin-1	646,343	1,728,698
P19338	NUCL_HUMAN	NCL	Nucleolin, splice variant or isoform	763,657,332	430,299,923
H7BY16	H7BY16_HUMAN	NCL	Nucleolin, splice variant or isoform	2,247,350	11,648,375
P06748-2	NPM_HUMAN	NPM1	Nucleophosmin, Isoform 2	-	41,741,528
P15531-2	NDKA_HUMAN	NME1*	Nucleoside diphosphate kinase A, Isoform 2	1,006,212	1,067,950
P55209-2	NP1L1_HUMAN	NAP1L1	Nucleosome assembly protein 1-like 1, Isoform 2	-	299,818
F8W543	F8W543_HUMAN	NAP1L1	Nucleosome assembly protein 1-like 1, splice variant or isoform	-	137,646
F8VRJ2	F8VRJ2_HUMAN	NAP1L1*	Nucleosome assembly protein 1-like 1, splice variant or isoform	-	40,227
C9J6D1	C9J6D1_HUMAN	NAP1L4	Nucleosome assembly protein 1-like 4	-	1,808,207
Q99733-2	NP1L4_HUMAN	NAP1L4	Nucleosome assembly protein 1-like 4, Isoform 2	-	474,352
J3KQ32	J3KQ32_HUMAN	OLA1	Obg-like ATPase 1	-	5,309,781
Q9NTK5-3	OLA1_HUMAN	OLA1	Obg-like ATPase 1, Isoform 3	-	817,131
Q9NQR4	NIT2_HUMAN	NIT2	Omega-amidase NIT2	11,264,350	6,926,225
Q92882	OSTF1_HUMAN	OSTF1*	Osteoclast-stimulating factor 1	-	660,738
P10451-5	OSTP_HUMAN	SPP1	Osteopontin, Isoform 5	101,059,363	1,058,784,138
P10451-2	OSTP_HUMAN	SPP1	Osteopontin, Isoform B	32,870,606	380,703,806
P10451-3	OSTP_HUMAN	SPP1	Osteopontin, Isoform C	13,000,106	-
P10451-4	OSTP_HUMAN	SPP1	Osteopontin, Isoform D	15,352,571	189,005,356
H9KVB3	H9KVB3_HUMAN	OTOG	Otogelin	16,235,000	10,968,568
F5GXR3	F5GXR3_HUMAN	PTMS*	Parathymosin	3,374,817	31,764,334
O75594	PGRP1_HUMAN	PGLYRP1	Peptidoglycan recognition protein 1	2,878,128	-
P19021-2	AMD_HUMAN	PAM	Peptidyl-glycine alpha-amidating monooxygenase, Isoform 2	653,391	2,866,300
C9J5S7	C9J5S7_HUMAN	PPIA*	Peptidyl-prolyl cis-trans isomerase	17,056,000	-
P62937	PPIA_HUMAN	PPIA	Peptidyl-prolyl cis-trans isomerase A	78,292,503	125,575,708
P23284	PPIB_HUMAN	PPIB	Peptidyl-prolyl cis-trans isomerase B	17,555,550	54,169,000
Q02790	FKBP4_HUMAN	FKBP4	Peptidyl-prolyl cis-trans isomerase FKBP4	-	5,808,244
K7EKI8	K7EKI8_HUMAN	PPL	Periplakin, splice variant or isoform	2,108,751	1,929,952
K7EQ71	K7EQ71_HUMAN	PPL	Periplakin, splice variant or isoform	514,376	988,727
Q06830	PRDX1_HUMAN	PRDX1	Peroxiredoxin-1	10,478,400	18,672,500
P32119	PRDX2_HUMAN	PRDX2	Peroxiredoxin-2	279,768,700	193,475,296
P30041	PRDX6_HUMAN	PRDX6	Peroxiredoxin-6	13,256,191	19,203,700
E7ER27	E7ER27_HUMAN	HSD17B4	Peroxisomal multifunctional enzyme type 2	5,316,050	15,104,525
B4E363	B4E363_HUMAN	FARSA	Phenylalanine--tRNA ligase alpha subunit	-	193,474
B7Z7A9	B7Z7A9_HUMAN	PGK1	Phosphoglycerate kinase	16,046,289	35,639,422
P18669	PGAM1_HUMAN	PGAM1	Phosphoglycerate mutase 1	13,947,500	3,986,560
Q8IV08	PLD3_HUMAN	PLD3*	Phospholipase D3	2,564,475	4,834,150
F5GWY2	F5GWY2_HUMAN	ATIC	Phosphoribosylaminoimidazolecarboxamide formyltransferase, splice variant or isofor	-	21,000,415
C9JLK0	C9JLK0_HUMAN	ATIC	Phosphoribosylaminoimidazolecarboxamide formyltransferase, splice variant or isofor	-	8,844,988
Q96BW5-2	PTER_HUMAN	PTER	Phosphotriesterase-related protein, Isoform 2	67,957,175	46,550,325
P36955	PEDF_HUMAN	SERPINF1	Pigment epithelium-derived factor	106,201,808	111,879,250
P03952	KLKB1_HUMAN	KLKB1	Plasma kallikrein	5,553,794	-
E9PBC5	E9PBC5_HUMAN	KLKB1*	Plasma kallikrein heavy chain	167,219	-
Q01814	AT2B2_HUMAN	ATP2B2	Plasma membrane calcium-transporting ATPase 2	74,720,550	104,285,550
B4E1F0	B4E1F0_HUMAN	SERPING1	Plasma protease C1 inhibitor, splice variant or isoform	18,573,754	32,708,833
H9KV48	H9KV48_HUMAN	SERPING1	Plasma protease C1 inhibitor, splice variant or isoform	17,602,088	29,563,316
P05154	IPSP_HUMAN	SERPINA5	Plasma serine protease inhibitor	69,531,646	115,557,250
P00747	PLMN_HUMAN	PLG	Plasminogen	595,371,160	1,637,340,383
P43034-2	LIS1_HUMAN	PAFAH1B1*	Platelet-activating factor acetylhydrolase IB subunit alpha, Isoform 2	-	356,188
P68402	PA1B2_HUMAN	PAFAH1B2*	Platelet-activating factor acetylhydrolase IB subunit beta	1,370,050	-
Q15102	PA1B3_HUMAN	PAFAH1B3*	Platelet-activating factor acetylhydrolase IB subunit gamma	771,875	519,291
Q15149	PLEC_HUMAN	PLEC	Plectin	10,990,156	6,010,000
Q15149-2	PLEC_HUMAN	PLEC*	Plectin, Isoform 2	402,106	411,100
Q15365	PCBP1_HUMAN	PCBP1	Poly(rC)-binding protein 1	6,751,915	-
F8VTZ0	F8VTZ0_HUMAN	PCBP2*	Poly(rC)-binding protein 2	13,315	-
Q15366-2	PCBP2_HUMAN	PCBP2	Poly(rC)-binding protein 2, Isoform 2	240,747	-
Q13310-2	PABP4_HUMAN	PABPC4	Polyadenylate-binding protein 4, Isoform 2	2,572,840	3,707,750
Q6P9A2	GLT18_HUMAN	GALNT18*	Polypeptide N-acetylgalactosaminyltransferase 18	-	1,821,200
P26599-2	PTBP1_HUMAN	PTBP1	Polypyrimidine tract-binding protein 1, Isoform 2	705,506	723,183
P02545-6	LMNA_HUMAN	LMNA	Prelamin-A/C, Isoform 6	-	39,283,425
P02545-3	LMNA_HUMAN	LMNA	Prelamin-A/C, Isoform ADelta10	-	40,115,550
B7Z3Y2	B7Z3Y2_HUMAN	PCYOX1*	Prenylcysteine oxidase 1	1,345,383	3,131,484
Q92841-1	DDX17_HUMAN	DDX17*	Probable ATP-dependent RNA helicase DDX17, Isoform 2	6,023,667	32,776,667
Q9H3G5	CPVL_HUMAN	CPVL	Probable serine carboxypeptidase CPVL	-	66,536,862
P01133-2	EGF_HUMAN	EGF	Pro-epidermal growth factor, Isoform 2	48,085,549	171,863,644
P01133-3	EGF_HUMAN	EGF	Pro-epidermal growth factor, Isoform 3	164,411,021	706,411,446

C9JQ45	C9JQ45_HUMAN	PFN2*	Profilin	-	1,587,425
K7EJ44	K7EJ44_HUMAN	PFN1*	Profilin 1	525,180	1,292,075
P07737	PROF1_HUMAN	PFN1	Profilin-1	577,422,287	433,907,411
P35080-2	PROF2_HUMAN	PFN2	Profilin-2, Isoform Iib	-	1,883,648
Q8WUM4-2	PDC6I_HUMAN	PDCD6IP*	Programmed cell death 6-interacting protein, Isoform 2	382,882	540,425
P35232	PHB_HUMAN	PHB	Prohibitin	99,688,500	62,799,000
P12273	PIP_HUMAN	PIP	Prolactin-inducible protein	3,276,749	10,852,450
P48147	PPCE_HUMAN	PREP	Prolyl endopeptidase	28,831,998	-
P07602-2	SAP_HUMAN	PSAP	Prosaposin, Isoform Sap-mu-6	-	52,579,112
Q15185-2	TEBP_HUMAN	PTGES3	Prostaglandin E synthase 3, Isoform 2	-	8,514,113
Q15185-3	TEBP_HUMAN	PTGES3	Prostaglandin E synthase 3, Isoform 3	-	9,628,713
Q14914-2	PTGR1_HUMAN	PTGR1	Prostaglandin reductase 1, Isoform 2	2,716,625	3,172,875
P41222	PTGDS_HUMAN	PTGDS	Prostaglandin-H2 D-isomerase, splice variant or isoform	-	1,926,607
F8W7I2	F8W7I2_HUMAN	PTGDS	Prostaglandin-H2 D-isomerase, splice variant or isoform	-	1,109,132
Q5SQ12	Q5SQ12_HUMAN	PTGDS*	Prostaglandin-H2 D-isomerase, splice variant or isoform	-	642,460
O43653	PSCA_HUMAN	PSCA	Prostate stem cell antigen	-	239,838,700
Q9UL46	PSME2_HUMAN	PSME2	Proteasome activator complex subunit 2	3,761,460	17,674,200
C9JCK5	C9JCK5_HUMAN	PSMA2*	Proteasome subunit alpha type-2	288,070	1,083,825
P25788-2	PSA3_HUMAN	PSMA3*	Proteasome subunit alpha type-3, Isoform 2	3,551,575	53,667,500
P28066	PSA5_HUMAN	PSMA5*	Proteasome subunit alpha type-5	-	1,314,541
P28074-3	PSB5_HUMAN	PSMB5*	Proteasome subunit beta type-5, Isoform 3	516,737	1,050,350
P02760	AMBP_HUMAN	AMBP	Protein AMBP (Alpha-1-Microglobulin/Bikunin Precursor)	56,350,228	250,472,087
P30101	PDIA3_HUMAN	PDIA3	Protein disulfide-isomerase A3	-	111,682,110
P13667	PDIA4_HUMAN	PDIA4	Protein disulfide-isomerase A4	-	25,169,879
P07237	PDIA1_HUMAN	P4HB	Protein disulfide-isomerase, splice variant or isoform	-	97,429,175
I3L312	I3L312_HUMAN	P4HB*	Protein disulfide-isomerase, splice variant or isoform	-	19,200,875
G5EA52	G5EA52_HUMAN	PDIA3	Protein disulfide-isomerase, splice variant or isoform	-	18,095,425
H7BZ94	H7BZ94_HUMAN	P4HB	Protein disulfide-isomerase, splice variant or isoform	-	10,754,217
I3L3P5	I3L3P5_HUMAN	P4HB	Protein disulfide-isomerase, splice variant or isoform	-	361,250
P49257	LMAN1_HUMAN	LMAN1*	Protein ERGIC-53	10,737,900	53,550,500
Q15435-2	PP1R7_HUMAN	PPP1R7*	Protein phosphatase 1 regulatory subunit 7, Isoform 2	423,589	787,456
Q8TE77	SSH3_HUMAN	SSH3	Protein phosphatase Slingshot homolog 3	-	11,245,575
Q8TE77-2	SSH3_HUMAN	SSH3	Protein phosphatase Slingshot homolog 3, Isoform 2	-	9,338,975
Q9P258	RCC2_HUMAN	RCC2	Protein RCC2	-	24,806,850
P31949	S10AB_HUMAN	S100A11	Protein S100-A11 (Calgizzarin)	8,704,835	6,277,158
P80511	S10AC_HUMAN	S100A12	Protein S100-A12 (Calgranulin-C)	190,946,016	-
P29034	S10A2_HUMAN	S100A2	Protein S100-A2 (S100 calcium-binding protein A2)	3,156,250	18,920,100
R4GN98	R4GN98_HUMAN	S100A6*	Protein S100-A6 (Calcyclin), splice variant or isoform	5,524,250	12,371,750
P06703	S10A6_HUMAN	S100A6	Protein S100-A6 (Calcyclin), splice variant or isoform	-	10,294,650
P05109	S10A8_HUMAN	S100A8	Protein S100-A8 (Calgranulin-A)	2,173,741,598	238,812,631
P06702	S10A9_HUMAN	S100A9	Protein S100-A9 (Calgranulin-B)	3,880,813,101	401,926,400
P25815	S100P_HUMAN	S100P	Protein S100-P (Migration-inducing gene 9 protein)	37,070,061	44,864,139
G3V453	G3V453_HUMAN	SAV1*	Protein salvador homolog 1	48,451,000	28,066,000
Q01105-2	SET_HUMAN	SET	Protein SET, Isoform 2	6,977,737	7,704,843
P0DME0	SETLP_HUMAN	SETSIP	Protein SETSIP	2,387,987	5,250,406
G3V2W1	G3V2W1_HUMAN	SERPINA10*	Protein Z-dependent protease inhibitor	166,586	460,234
B4DIT7	B4DIT7_HUMAN	TGM2	Protein-glutamine gamma-glutamyltransferase 2	-	8,367,786
Q13882-2	PTK6_HUMAN	PTK6	Protein-tyrosine kinase 6, Isoform 2	-	18,871,973
Q9Y2Y8	PRG3_HUMAN	PRG3	Proteoglycan 3	48,549,965	-
P00734	THRB_HUMAN	F2	Prothrombin	1,458,024,103	8,139,053,926
Q9UN67	PCDBA_HUMAN	PCDHB10*	Protocadherin beta-10	957,367	3,628,500
Q9Y5H0-2	PCDG3_HUMAN	PCDHGA3*	Protocadherin gamma-A3, Isoform 2	45,033,750	312,112,500
Q9UN70-2	PCDGK_HUMAN	PCDHGC3	Protocadherin gamma-C3, Isoform 2	10,422,600	37,228,784
P12931-2	SRC_HUMAN	SRC*	Proto-oncogene tyrosine-protein kinase Src, Isoform 2	1,219,075	2,277,100
P00491	PNPH_HUMAN	PNP*	Purine nucleoside phosphorylase	975,292	3,559,650
O43865	SAHH2_HUMAN	AHCYL1	Putative adenosylhomocysteinase 2	6,520,724	15,635,500
P48741	HSP77_HUMAN	HSPA7	Putative heat shock 70 kDa protein 7	2,019,482	4,534,959
Q58FF6	H90B4_HUMAN	HSP90AB4P*	Putative heat shock protein HSP 90-beta 4	1,892,550	11,278,000
O43143	DHX15_HUMAN	DHX15	Putative pre-mRNA-splicing factor ATP-dependent RNA helicase DHX15	6,164,823	46,611,950
Q8NFI4	F10A5_HUMAN	ST13P5	Putative protein FAM10A5	-	600,780
Q9Y383-2	LC7L2_HUMAN	LUC7L2	Putative RNA-binding protein Luc7-like 2, Isoform 2	13,754,263	6,014,888
F2Z2Y4	F2Z2Y4_HUMAN	PDXK	Pyridoxal kinase	-	4,476,525
E7EMH5	E7EMH5_HUMAN	PDXDC1*	Pyridoxal-dependent decarboxylase domain-containing protein 1	208,584	649,817
P14618	KPYM_HUMAN	PKM	Pyruvate kinase PKM	73,895,187	65,650,482
P50395-2	GDIB_HUMAN	GDI2	Rab GDP dissociation inhibitor beta, Isoform 2	-	6,297,405
P46060	RAGP1_HUMAN	RANGAP1	Ran GTPase-activating protein 1, splice variant or isoform	-	41,960,534
F8W7I9	F8W7I9_HUMAN	RANGAP1	Ran GTPase-activating protein 1, splice variant or isoform	-	7,059,534
F5H4D6	F5H4D6_HUMAN	G3BP1*	Ras GTPase-activating protein-binding protein 1	2,636,725	7,574,325
P46940	IQGA1_HUMAN	IQGAP1	Ras GTPase-activating-like protein IQGAP1, splice variant or isoform	107,904,876	122,263,417
H0YLE8	H0YLE8_HUMAN	IQGAP1	Ras GTPase-activating-like protein IQGAP1, splice variant or isoform	2,151,402	6,575,744
P63000-2	RAC1_HUMAN	RAC1	Ras-related C3 botulinum toxin substrate 1, Isoform B	34,905,400	19,907,950
P15153	RAC2_HUMAN	RAC2	Ras-related C3 botulinum toxin substrate 2, splice variant or isoform	46,340,050	51,869,550
B1AH78	B1AH78_HUMAN	RAC2	Ras-related C3 botulinum toxin substrate 2, splice variant or isoform	12,842,150	39,933,575
P61026	RAB10_HUMAN	RAB10	Ras-related protein Rab-10	-	3,632,656
P62491-2	RB11A_HUMAN	RAB11A	Ras-related protein Rab-11A, Isoform 2	17,093,550	14,952,707
P62820-3	RAB1A_HUMAN	RAB1A	Ras-related protein Rab-1A, Isoform 3	1,083,244	2,437,366
P62820	RAB1A_HUMAN	RAB1A	Ras-related protein Rab-1A, splice variant or isoform	3,136,574	5,036,341
B7Z8M7	B7Z8M7_HUMAN	RAB1A	Ras-related protein Rab-1A, splice variant or isoform	1,484,119	2,897,391
C9J8S3	C9J8S3_HUMAN	RAB7A	Ras-related protein Rab-7a, splice variant or isoform	-	1,693,913
C9J4S4	C9J4S4_HUMAN	RAB7A*	Ras-related protein Rab-7a, splice variant or isoform	-	402,238
P62834	RAP1A_HUMAN	RAP1A	Ras-related protein Rap-1A	453,998	1,338,966
F5GYB5	F5GYB5_HUMAN	RAP1B*	Ras-related protein Rap-1b	320,842	746,816
B7Z413	B7Z413_HUMAN	ATP6AP2	Renin receptor	23,876,241	29,770,450
Q9HD89	RETN_HUMAN	RETN	Resistin	28,659,475	33,509,477
Q9HB40	RISC_HUMAN	SCPEP1	Retinoid-inducible serine carboxypeptidase	-	17,522,550
Q5VY30	Q5VY30_HUMAN	RBP4	Retinol binding protein 4, plasma	3,378,600	-
P52566	GDIR2_HUMAN	ARHGDIB*	Rho GDP-dissociation inhibitor 2	4,538,600	1,310,310

Q9HCE6-2	ARGAL_HUMAN	ARHGEF10L	Rho guanine nucleotide exchange factor 10-like protein, Isoform 2	7,270,779	16,146,530
P62745	RHOB_HUMAN	RHOB*	Rho-related GTP-binding protein RhoB	66,093	69,838
P08134	RHOC_HUMAN	RHOC	Rho-related GTP-binding protein RhoC	8,726,896	8,279,369
E9PQH6	E9PQH6_HUMAN	RHOC	Rho-related GTP-binding protein RhoC	5,826,230	5,762,819
Q8WZ75-2	ROBO4_HUMAN	ROBO4	Roundabout homolog 4, Isoform 2	-	3,271,995
B3KQ59	B3KQ59_HUMAN	RUVBL2	RuvB-like 2, splice variant or isoform	3,003,186	6,096,021
X6R2L4	X6R2L4_HUMAN	RUVBL2*	RuvB-like 2, splice variant or isoform	846,136	307,188
P16615-2	AT2A2_HUMAN	ATP2A2	Sarcoplasmic/endoplasmic reticulum calcium ATPase 2, Isoform 2	-	1,345,145
Q93084-2	AT2A3_HUMAN	ATP2A3*	Sarcoplasmic/endoplasmic reticulum calcium ATPase 3, Isoform SERCA3A	-	150,612
J3QQU6	J3QQU6_HUMAN	SECTM1	Secreted and transmembrane protein 1	12,246,746	15,359,086
Q13103	SPP24_HUMAN	SPP2	Secreted phosphoprotein 24, splice variant or isoform	-	58,294,350
C9J6K0	C9J6K0_HUMAN	SPP2*	Secreted phosphoprotein 24, splice variant or isoform	-	18,100,000
P05060	SCG1_HUMAN	CHGB*	Secretogranin-1	-	1,468,500
Q15019-2	SEPT2_HUMAN	SEPT2	Septin-2, Isoform 2	-	434,028
Q9UHD8-2	SEPT9_HUMAN	SEPT9*	Septin-9, Isoform 2	4,174,250	6,936,625
O15269	SPTC1_HUMAN	SPTLC1*	Serine palmitoyltransferase 1	-	1,832,250
Q07955	SRSF1_HUMAN	SRSF1	Serine/arginine-rich splicing factor 1	40,541,970	63,616,501
O75494-2	SRS10_HUMAN	SRSF10	Serine/arginine-rich splicing factor 10, Isoform 2	-	631,129
Q05519-2	SRS11_HUMAN	SRSF11	Serine/arginine-rich splicing factor 11, Isoform 2	-	1,563,672
P84103	SRSF3_HUMAN	SRSF3	Serine/arginine-rich splicing factor 3	35,858,611	44,384,407
Q13247-3	SRSF6_HUMAN	SRSF6	Serine/arginine-rich splicing factor 6, Isoform SRP55-3	-	44,356,700
Q16629-2	SRSF7_HUMAN	SRSF7	Serine/arginine-rich splicing factor 7, Isoform 2	2,573,270	6,956,207
Q9BRL6-2	SRSF8_HUMAN	SRSF8*	Serine/arginine-rich splicing factor 8, Isoform 2	-	3,248,417
J3KSR8	J3KSR8_HUMAN	SRSF1	Serine/arginine-rich-splicing factor 1	272,695	1,767,651
B4DN89	B4DN89_HUMAN	SFRS2	Serine/arginine-rich-splicing factor 2	-	4,020,129
H0Y630	H0Y630_HUMAN	STK24	Serine/threonine-protein kinase 24 12 kDa subunit, splice variant or isoform	-	4,056,487
Q5JV98	Q5JV98_HUMAN	STK24	Serine/threonine-protein kinase 24 12 kDa subunit, splice variant or isoform	-	1,018,862
Q9Y6E0-2	STK24_HUMAN	STK24	Serine/threonine-protein kinase 24, Isoform A	-	8,152,337
Q9P289-2	MST4_HUMAN	MST4	Serine/threonine-protein kinase MST4, Isoform 2	-	347,054
H0Y494	H0Y494_HUMAN	SIK3*	Serine/threonine-protein kinase SIK3	2,712,000	2,540,500
C9J9S3	C9J9S3_HUMAN	PPP1CB	Serine/threonine-protein phosphatase	-	3,940,353
P63151	2ABA_HUMAN	PPP2R2A	Serine/threonine-protein phosphatase 2A 55 kDa regulatory subunit B alpha isoform	5,748,926	11,699,325
E5RIY1	E5RIY1_HUMAN	PPP2R2A*	Serine/threonine-protein phosphatase 2A 55 kDa regulatory subunit B alpha isoform	-	358,675
Q66LE6	2ABD_HUMAN	PPP2R2D*	Serine/threonine-protein phosphatase 2A 55 kDa regulatory subunit B delta isoform	1,950,100	5,854,000
P30153	2AAA_HUMAN	PPP2R1A	Serine/threonine-protein phosphatase 2A 65 kDa regulatory subunit A alpha isoform	193,145,652	62,667,738
C9J9C1	C9J9C1_HUMAN	PPP2R1A	Serine/threonine-protein phosphatase 2A 65 kDa regulatory subunit A alpha isoform	17,933,541	7,476,167
P67775-2	PP2AA_HUMAN	PPP2CA	Serine/threonine-protein phosphatase 2A catalytic subunit alpha isoform, Isoform 2	-	3,856,831
P62714	PP2AB_HUMAN	PPP2CB	Serine/threonine-protein phosphatase 2A catalytic subunit beta isoform	-	8,948,300
P62136-2	PP1A_HUMAN	PPP1CA*	Serine/threonine-protein phosphatase PP1-alpha catalytic subunit, Isoform 2	-	33,853
P62140	PP1B_HUMAN	PPP1CB	Serine/threonine-protein phosphatase PP1-beta catalytic subunit	-	42,177,803
P02787	TRFE_HUMAN	TF	Serotransferrin	166,048,740	268,424,446
E9PPV6	E9PPV6_HUMAN	SERPINH1	Serpin H1	1,191,561	2,814,322
P02768	ALBU_HUMAN	ALB	Serum albumin	991,522,378	1,608,428,499
P02743	SAMP_HUMAN	APCS	Serum amyloid P-component	93,853,629	81,426,000
P27169	PON1_HUMAN	PON1	Serum paraoxonase/arylesterase 1	281,120,032	299,788,401
Q9HAT2-2	SIAE_HUMAN	SIAE	Sialate O-acetylesterase, Isoform 2	-	2,519,301
E9PIF4	E9PIF4_HUMAN	NEU1	Sialidase-1	19,058,550	19,207,825
F5H5Y3	F5H5Y3_HUMAN	SRP68	Signal recognition particle subunit SRP68	-	2,078,700
Q9UHB9-2	SRP68_HUMAN	SRP68*	Signal recognition particle subunit SRP68, Isoform 2	-	869,500
Q9UHB9-4	SRP68_HUMAN	SRP68	Signal recognition particle subunit SRP68, Isoform 4	-	4,917,650
P42224	STAT1_HUMAN	STAT1	Signal transducer and activator of transcription 1-alpha/beta	-	40,520,350
P42226-2	STAT6_HUMAN	STAT6*	Signal transducer and activator of transcription 6, Isoform 2	1,097,038	3,587,875
G3V3E1	G3V3E1_HUMAN	SKOR1	SKI family transcriptional corepressor 1	4,381,134	159,346,967
Q9UIB8-2	SLAF5_HUMAN	CD84	SLAM family member 5, Isoform 2	-	2,428,592
C9J3M6	C9J3M6_HUMAN	SLC9C1*	Sodium/hydrogen exchanger 10	61,596	76,534
P05023-3	AT1A1_HUMAN	ATP1A1	Sodium/potassium-transporting ATPase subunit alpha-1, Isoform 3	-	22,222,002
B1AKY9	B1AKY9_HUMAN	ATP1A2	Sodium/potassium-transporting ATPase subunit alpha-2	-	3,870,735
Q13813-2	SPTN1_HUMAN	SPTAN1	Spectrin alpha chain, non-erythrocytic 1, Isoform 2	9,404,427	8,683,672
Q01082-3	SPTB2_HUMAN	SPTBN1	Spectrin beta chain, non-erythrocytic 1, Isoform 2	-	6,410,151
P26368	U2AF2_HUMAN	U2AF2	Splicing factor U2AF 65 kDa subunit	-	11,155,633
Q8WXA9-2	SREK1_HUMAN	SREK1*	Splicing regulatory glutamine/lysine-rich protein 1, Isoform 2	-	5,061,300
Q7KZF4	SND1_HUMAN	SND1*	Staphylococcal nuclease domain-containing protein 1	39,424,250	11,475,750
Q9UEW8	STK39_HUMAN	STK39	STE20/SPS1-related proline-alanine-rich protein kinase	-	7,321,221
P31040-2	SDHA_HUMAN	SDHA*	Succinate dehydrogenase [ubiquinone] flavoprotein subunit, mitochondrial, Isoform 2	-	161,465
P53597	SUCA_HUMAN	SUCLG1*	Succinyl-CoA ligase [ADP/GDP-forming] subunit alpha, mitochondrial	8,666,900	7,283,850
B1AH49	B1AH49_HUMAN	MPST	Sulfurtransferase	-	1,098,200
P00441	SODC_HUMAN	SOD1	Superoxide dismutase [Cu-Zn]	-	17,091,500
Q9UGT4	SUSD2_HUMAN	SUSD2	Sushi domain-containing protein 2	3,085,671	15,385,400
E9PHH3	E9PHH3_HUMAN	SDC1	Syndecan-1	-	19,463,516
P31431-2	SDC4_HUMAN	SDC4	Syndecan-4, Isoform 2	-	22,142,325
E9PLE5	E9PLE5_HUMAN	RIC8A*	Synembryn-A	-	363,459
Q9NPQ8-3	RIC8A_HUMAN	RIC8A	Synembryn-A, Isoform 3	-	3,020,709
Q5TCU6	Q5TCU6_HUMAN	TLN1	Talin-1	3,583,749	5,092,449
P17987	TCPA_HUMAN	TCP1	T-complex protein 1 subunit alpha, splice variant or isoform	30,337,927	69,314,234
E7EQR6	E7EQR6_HUMAN	TCP1	T-complex protein 1 subunit alpha, splice variant or isoform	7,314,883	12,615,534
P78371-2	TCPB_HUMAN	CCT2*	T-complex protein 1 subunit beta, Isoform 2	247,079	3,569,250
P78371	TCPB_HUMAN	CCT2	T-complex protein 1 subunit beta, splice variant or isoform	130,150,938	150,570,006
F5GWF6	F5GWF6_HUMAN	CCT2	T-complex protein 1 subunit beta, splice variant or isoform	557,414	3,921,867
B7Z2F4	B7Z2F4_HUMAN	CCT4	T-complex protein 1 subunit delta	9,022,688	11,493,531
P50991-2	TCPD_HUMAN	CCT4	T-complex protein 1 subunit delta, Isoform 2	9,897,473	14,526,781
P48643-2	TCPE_HUMAN	CCT5	T-complex protein 1 subunit epsilon, Isoform 2	-	2,695,540
Q99832-3	TCPH_HUMAN	CCT7	T-complex protein 1 subunit eta, Isoform 3	5,549,791	7,240,551
P49368-2	TCPG_HUMAN	CCT3	T-complex protein 1 subunit gamma, Isoform 2	-	13,695,485
P50990-2	TCPQ_HUMAN	CCT8*	T-complex protein 1 subunit theta, Isoform 2	2,790,167	5,204,334
P40227-2	TCPZ_HUMAN	CCT6A	T-complex protein 1 subunit zeta, Isoform 2	-	14,553,686
P40227	TCPZ_HUMAN	CCT6A	T-complex protein 1 subunit zeta, splice variant or isoform	-	54,303,436
B4DPJ8	B4DPJ8_HUMAN	CCT6A	T-complex protein 1 subunit zeta, splice variant or isoform	-	41,079,106

P24821-2	TENA_HUMAN	TNC*	Tenascin, Isoform 2	-	390,639
F5H283	F5H283_HUMAN	SPOCK2	Testican-2	-	49,282,850
E9PHK0	E9PHK0_HUMAN	CLEC3B	Tetranectin	7,928,165	-
P30048-2	PRDX3_HUMAN	PRDX3*	Thioredoxin-dependent peroxide reductase, mitochondrial, Isoform 2	-	772,975
E9PIT3	E9PIT3_HUMAN	F2	Thrombin light chain	314,527,489	4,084,473,868
P07996	TSP1_HUMAN	THBS1	Thrombospondin-1	-	216,602,642
P35442	TSP2_HUMAN	THBS2*	Thrombospondin-2	34,843	450,179
E7ES19	E7ES19_HUMAN	THBS4	Thrombospondin-4	-	5,021,150
P05543	THBG_HUMAN	SERPINA7	Thyroxine-binding globulin	1,505,050	2,058,569
Q9UDY2-6	ZO2_HUMAN	TJP2	Tight junction protein ZO-2, Isoform 6	-	9,097,186
Q86XR7-2	TCAM2_HUMAN	TICAM2*	TIR domain-containing adapter molecule 2, Isoform 2	151,361	294,359
Q8WZ42-10	TITIN_HUMAN	TTN	Titin, Isoform 10	1,415,633	-
P20290-2	BTF3_HUMAN	BTF3*	Transcription factor BTF3, Isoform 2	134,864	726,350
P35716	SOX11_HUMAN	SOX11*	Transcription factor SOX-11	3,831,650	12,927,000
Q06945	SOX4_HUMAN	SOX4*	Transcription factor SOX-4	13,319,000	-
J3KNC0	J3KNC0_HUMAN	GTF2A1*	Transcription initiation factor IIA beta chain	-	1,550,950
Q00577	PURA_HUMAN	PURA*	Transcriptional activator protein Pur-alpha	1,016,861	1,269,300
Q13595-2	TRA2A_HUMAN	TRA2A	Transformer-2 protein homolog alpha, Isoform Short	-	3,135,000
P62995-3	TRA2B_HUMAN	TRA2B	Transformer-2 protein homolog beta, Isoform 3	-	20,075,175
P61586	RHOA_HUMAN	RHOA	Transforming protein RhoA, splice variant or isoform	11,123,213	84,825,653
C9JX21	C9JX21_HUMAN	RHOA	Transforming protein RhoA, splice variant or isoform	1,971,896	2,895,103
C9JRM1	C9JRM1_HUMAN	RHOA	Transforming protein RhoA, splice variant or isoform	1,902,693	2,726,571
P55072	TERA_HUMAN	VCP	Transitional endoplasmic reticulum ATPase	-	164,777,603
P29401-2	TKT_HUMAN	TKT	Transketolase, Isoform 2	18,172,681	18,007,984
P43307-2	SSRA_HUMAN	SSR1*	Translocon-associated protein subunit alpha, Isoform 2	275,472	208,372
P02766	TTHY_HUMAN	TTR	Transthyretin	8,481,736	30,431,950
P40939	ECHA_HUMAN	HADHA*	Trifunctional enzyme subunit alpha, mitochondrial	1,194,377	6,767,400
P60174-1	TPIS_HUMAN	TPI1	Triosephosphate isomerase, Isoform 2	21,329,453	58,313,641
P60174-4	TPIS_HUMAN	TPI1	Triosephosphate isomerase, Isoform 4	3,985,319	12,810,983
B3KP96	B3KP96_HUMAN	TRIM16	Tripartite motif-containing protein 16, splice variant or isoform	-	4,552,587
K7EL43	K7EL43_HUMAN	TRIM16	Tripartite motif-containing protein 16, splice variant or isoform	-	2,612,920
O14773-2	TPP1_HUMAN	TPP1	Tripeptidyl-peptidase 1, Isoform 2	50,114,860	260,657,402
P06753-2	TPM3_HUMAN	TPM3	Tropomyosin alpha-3 chain, Isoform 2	20,710,875	36,602,188
P68363	TBA1B_HUMAN	TUBA1B	Tubulin alpha-1B chain	5,452,370	28,041,806
F5H5D3	F5H5D3_HUMAN	TUBA1C	Tubulin alpha-1C chain	21,751,875	63,084,743
P68366-2	TBA4A_HUMAN	TUBA4A	Tubulin alpha-4A chain, Isoform 2	13,093,579	56,128,114
P07437	TBB5_HUMAN	TUBB	Tubulin beta chain	156,170,396	932,888,690
Q13885	TBB2A_HUMAN	TUBB2A	Tubulin beta-2A chain	-	114,110,975
Q9BVA1	TBB2B_HUMAN	TUBB2B	Tubulin beta-2B chain	-	46,051,250
Q13509	TBB3_HUMAN	TUBB3	Tubulin beta-3 chain	-	21,267,400
P04350	TBB4A_HUMAN	TUBB4A	Tubulin beta-4A chain	-	3,061,033
P68371	TBB4B_HUMAN	TUBB4B	Tubulin beta-4B chain	36,247,278	296,070,225
Q9BUF5	TBB6_HUMAN	TUBB6	Tubulin beta-6 chain	-	21,877,604
Q14166	TTL12_HUMAN	TTLL12	Tubulin--tyrosine ligase-like protein 12	10,041,650	13,307,400
P09758	TACD2_HUMAN	TACSTD2	Tumor-associated calcium signal transducer 2	-	6,725,624
Q12923-3	PTN13_HUMAN	PTPN13	Tyrosine-protein phosphatase non-receptor type 13, Isoform 3	1,187,412	4,717,551
F5GY79	F5GY79_HUMAN	PTPN6*	Tyrosine-protein phosphatase non-receptor type 6	361,971	379,950
P29350-3	PTN6_HUMAN	PTPN6	Tyrosine-protein phosphatase non-receptor type 6, Isoform 2	497,974	440,035
J3QTR3	J3QTR3_HUMAN	RPS27A	Ubiquitin	504,778	3,221,572
Q70CQ4	UBP31_HUMAN	USP31*	Ubiquitin carboxyl-terminal hydrolase 31	31,726,000	18,423,650
P45974-2	UBP5_HUMAN	USP5*	Ubiquitin carboxyl-terminal hydrolase 5, Isoform Short	807,300	2,800,350
P15374	UCHL3_HUMAN	UCHL3	Ubiquitin carboxyl-terminal hydrolase isozyme L3	17,098,400	13,710,950
F5GYJ8	F5GYJ8_HUMAN	OTUB1	Ubiquitin thioesterase OTUB1	-	4,724,602
Q9UGI0	ZRAN1_HUMAN	ZRANB1*	Ubiquitin thioesterase ZRANB1	15,768,500	4,272,850
M0R1V7	M0R1V7_HUMAN	UBA52*	Ubiquitin-60S ribosomal protein L40	21,282	69,014
P62256-2	UBE2H_HUMAN	UBE2H	Ubiquitin-conjugating enzyme E2 H, Isoform 2	-	1,367,534
P22314	UBA1_HUMAN	UBA1	Ubiquitin-like modifier-activating enzyme 1, splice variant or isoform	87,952,089	81,313,664
Q5JRR9	Q5JRR9_HUMAN	UBA1*	Ubiquitin-like modifier-activating enzyme 1, splice variant or isoform	1,833,083	2,196,750
P41226	UBA7_HUMAN	UBA7*	Ubiquitin-like modifier-activating enzyme 7	-	2,523,250
Q14376	GALE_HUMAN	GALE	UDP-glucose 4-epimerase, splice variant or isoform	-	36,674,000
Q5QPP4	Q5QPP4_HUMAN	GALE	UDP-glucose 4-epimerase, splice variant or isoform	-	9,371,500
Q5QPP9	Q5QPP9_HUMAN	GALE	UDP-glucose 4-epimerase, splice variant or isoform	-	2,838,000
P22309-2	UD11_HUMAN	UGT1A1*	UDP-glucuronosyltransferase 1-1, Isoform 2	-	92,450
P19224-3	UD16_HUMAN	UGT1A6	UDP-glucuronosyltransferase 1-6, Isoform 3	-	9,016,775
I3L2A7	I3L2A7_HUMAN	KIAA0753*	Uncharacterized protein KIAA0753	176,266,667	45,530,000
O00159-2	MYO1C_HUMAN	MYO1C	Unconventional myosin-Ic, Isoform 2	-	3,962,805
I3L501	I3L501_HUMAN	MYO1C	Unconventional myosin-Ic, splice variant or isoform	-	4,681,805
I3L168	I3L168_HUMAN	MYO1C*	Unconventional myosin-Ic, splice variant or isoform	-	156,055
Q9UM54-1	MYO6_HUMAN	MYO6	Unconventional myosin-VI, Isoform 1	-	3,560,650
P07911-3	UROM_HUMAN	UMOD	Uromodulin, Isoform 3	53,707,569	47,021,585
P07911-4	UROM_HUMAN	UMOD	Uromodulin, Isoform 4	75,910,148	81,675,080
O00526	UPK2_HUMAN	UPK2*	Uroplakin-2	2,736,750	7,012,200
O75631	UPK3A_HUMAN	UPK3A	Uroplakin-3a	-	59,233,250
Q16851-2	UGPA_HUMAN	UGP2	UTP--glucose-1-phosphate uridylyltransferase, Isoform 2	5,061,550	5,806,400
Q709C8-2	VP13C_HUMAN	VPS13C*	Vacuolar protein sorting-associated protein 13C, Isoform 2	111,867	387,113
Q96QK1	VPS35_HUMAN	VPS35	Vacuolar protein sorting-associated protein 35	10,113,200	18,447,250
O75351	VPS4B_HUMAN	VPS4B*	Vacuolar protein sorting-associated protein 4B	662,401	2,980,750
P26640	SYVC_HUMAN	VARS	Valine--tRNA ligase	-	244,758,600
B4DFA2	B4DFA2_HUMAN	NSF	Vesicle-fusing ATPase	43,248,700	52,723,676
I3L2G1	I3L2G1_HUMAN	NSF	Vesicle-fusing ATPase	935,870	2,975,710
D6RBV2	D6RBV2_HUMAN	LMAN2	Vesicular integral-membrane protein VIP36	297,167,233	482,547,231
P18206-2	VINC_HUMAN	VCL*	Vinculin, Isoform 1	789,767	589,450
P04070-2	PROC_HUMAN	PROC	Vitamin K-dependent protein C, Isoform 2	13,134,413	69,580,718
P07225	PROS_HUMAN	PROS1	Vitamin K-dependent protein S, splice variant or isoform	245,772,617	240,873,106
P07225	PROS_HUMAN	PROS1	Vitamin K-dependent protein S, splice variant or isoform	166,848,241	184,747,788
P22891-2	PROZ_HUMAN	PROZ	Vitamin K-dependent protein Z, Isoform 2	111,400,138	1,928,709,853
Q7Z5L0	VMO1_HUMAN	VMO1	Vitelline membrane outer layer protein 1 homolog	25,242,316	84,014,362

P04004	VTNC_HUMAN	VTN	Vitronectin	431,537,552	2,439,437,791
P21796	VDAC1_HUMAN	VDAC1	Voltage-dependent anion-selective channel protein 1	-	57,906,906
Q8WXS5	CCG8_HUMAN	CACNG8*	Voltage-dependent calcium channel gamma-8 subunit	1,812,250	1,932,450
J3QRR0	J3QRR0_HUMAN	VWA1	von Willebrand factor A domain-containing protein 1, splice variant or isoform	1,048,834	1,347,767
Q6PCB0	VWA1_HUMAN	VWA1	von Willebrand factor A domain-containing protein 1, splice variant or isoform	-	8,759,716
J3QLP3	J3QLP3_HUMAN	VWA1	von Willebrand factor A domain-containing protein 1, splice variant or isoform	-	2,884,267
B7Z1R5	B7Z1R5_HUMAN	ATP6V1A	V-type proton ATPase catalytic subunit A	-	10,695,300
P21281	VATB2_HUMAN	ATP6V1B2	V-type proton ATPase subunit B, brain isoform	4,846,124	5,805,775
E7EQI7	E7EQI7_HUMAN	KIAA0196*	WASH complex subunit strumpellin	1,305,525	2,439,200
O75083	WDR1_HUMAN	WDR1	WD repeat-containing protein 1, splice variant or isoform	265,599,512	180,166,720
D6RD66	D6RD66_HUMAN	WDR1	WD repeat-containing protein 1, splice variant or isoform	9,230,529	16,877,325
P12955-2	PEPD_HUMAN	PEPD*	Xaa-Pro dipeptidase, Isoform 2	24,550,500	11,489,667
P13010	XRCC5_HUMAN	XRCC5*	X-ray repair cross-complementing protein 5	3,224,525	3,100,025
P12956	XRCC6_HUMAN	XRCC6	X-ray repair cross-complementing protein 6, splice variant or isoform	-	5,846,000
B1AHC9	B1AHC9_HUMAN	XRCC6	X-ray repair cross-complementing protein 6, splice variant or isoform	-	3,103,450
Q9Y2T7	YBOX2_HUMAN	YBX2	Y-box-binding protein 2	19,039,050	22,699,500
Q14929	ZN169_HUMAN	ZNF169	Zinc finger protein 169	15,616,900	172,307,500
Q9Y2X9-2	ZN281_HUMAN	ZNF281	Zinc finger protein 281, Isoform 2	790,671	9,025,300
Q9UDV7	ZN282_HUMAN	ZNF282*	Zinc finger protein 282	366,661	2,575,000
Q9Y493-2	ZAN_HUMAN	ZAN*	Zonadhesin, Isoform 1	660,800	415,445

*proteins identified and quantified by 1 peptide; average protein probability 0.977375573
