# Supplementary material for: Label-free proteomic methodology for the analysis of human kidney stone matrix composition
Source: Proteome Sci. 2016 Feb 27;14:4. doi: 10.1186/s12953-016-0093-x (PMC4769560; doi:10.1186/s12953-016-0093-x)
Supplement: Additional file 2: — Peptide sequence/Protein Identification, mass spectral data for 5,957 peptides. (PDF 3099 kb) [file 12953_2016_93_MOESM2_ESM.pdf]

SUPPLEMENTAL TABLE 2. Peptide/Protein Identification Data.

Entry Q#	UniProt Protein ID	UniProt Protein Entry	Gene Name	Protein Name	Protein Probability	Protein Coverage (%)	# of Unique Sequences	# of Identified Peptides	Total # of Identified Spectra
						Peptide Sequence	Peptide Charge	Peptide Probability	Number of Spectra
Q$1-1	E9PNH7	E9PNH7_HUMAN	CRYAB	Alpha-crystallin B chain (Fragment)	1.0000	15.09	2	2	2
Q$1-2	A0A024R3B9	A0A024R3B9_HUMAN	CRYAB	Crystallin, alpha B, isoform CRA_c	1.0000	14.81	2	2	2
						FSVNLDVK	2	0.9658	1
						HFSPEELK	2	0.9879	1
Q$2-1	A2A3R5	A2A3R5_HUMAN	RPS6	40S ribosomal protein S6	0.9863	6.88	1	1	2
Q$2-2	A2A3R7	A2A3R7_HUMAN	RPS6	40S ribosomal protein S6	0.9863	16.48	1	1	2
Q$2-3	P62753	RS6_HUMAN	RPS6	40S ribosomal protein S6	0.9863	6.02	1	1	2
						MATEVAADALGEEWK	2	0.9863	2
Q$3-1	P36915	GNL1_HUMAN	GNL1	Guanine nucleotide-binding protein-like 1	0.9999	4.94	2	2	3
Q$3-2	A2AB27	A2AB27_HUMAN	GNL1	Guanine nucleotide-binding protein-like 1 (Fragment)	0.9999	11.36	2	2	3
						EQVLQPVSAELLELDIR	2	0.9952	1
						EVYQPGSVLDFPR	2	0.9883	2
Q$4-1	Q9UM22	EPDR1_HUMAN	EPDR1	Mammalian ependymin-related protein 1	0.9973	10.27	2	2	2
Q$4-2	A4D1W8	A4D1W8_HUMAN	UCC1	Ependymin related protein 1 (Zebrafish), isoform CRA_b	0.9973	6.69	2	2	2
						ALLSYDGLNQR	2	0.9669	1
						SYETWIGIYTVK	2	0.9191	1
Q$5-1	A6NC17	A6NC17_HUMAN	MARS	Methionine--tRNA ligase, cytoplasmic	1.0000	3.62	2	2	3
Q$5-2	B3KVK7	B3KVK7_HUMAN	MARS	Methionine--tRNA ligase, cytoplasmic	1.0000	4.21	2	2	3
Q$5-3	P56192	SYMC_HUMAN	MARS	Methionine--tRNA ligase, cytoplasmic	1.0000	2.56	2	2	3
Q$5-4	H0YHV5	H0YHV5_HUMAN	MARS	Methionine--tRNA ligase, cytoplasmic (Fragment)	1.0000	9.87	2	2	3
						GFVLQDTVEQLR	2	0.9703	1
						WFNISFDIFGR	2	0.9993	2
Q$6-1	P08865	RSSA_HUMAN	RPSA	40S ribosomal protein SA	0.9150	5.76	1	1	1
Q$6-2	C9J9K3	C9J9K3_HUMAN	RPSA	40S ribosomal protein SA (Fragment)	0.9150	6.46	1	1	1
Q$6-3	A6NE09	A6NE09_HUMAN	RPSAP58	40S ribosomal protein SA	0.9150	5.76	1	1	1
						AIVAIENPADVSVISSR	2	0.915	1
Q$7-1	A6NFN2	A6NFN2_HUMAN	ABI1	Abl interactor 1	0.9993	2.16	1	1	1
Q$7-2	F5H1G9	F5H1G9_HUMAN	ABI1	Abl interactor 1	0.9993	2.79	1	1	1
Q$7-3	F8WA55	F8WA55_HUMAN	ABI1	Abl interactor 1	0.9993	2.6	1	1	1
Q$7-4	F8WA56	F8WA56_HUMAN	ABI1	Abl interactor 1	0.9993	2.28	1	1	1
Q$7-5	Q8IZP0	ABI1_HUMAN	ABI1	Abl interactor 1	0.9993	2.17	1	1	1
Q$7-6	Q8IZP0-10	ABI1_HUMAN	ABI1	Isoform 10 of Abl interactor 1	0.9993	2.84	1	1	1
Q$7-7	Q8IZP0-11	ABI1_HUMAN	ABI1	Isoform 11 of Abl interactor 1	0.9993	3.34	1	1	1
Q$7-8	Q8IZP0-12	ABI1_HUMAN	ABI1	Isoform 12 of Abl interactor 1	0.9993	2.22	1	1	1
Q$7-9	Q8IZP0-2	ABI1_HUMAN	ABI1	Isoform 2 of Abl interactor 1	0.9993	2.47	1	1	1
Q$7-10	Q8IZP0-3	ABI1_HUMAN	ABI1	Isoform 3 of Abl interactor 1	0.9993	2.43	1	1	1
Q$7-11	Q8IZP0-4	ABI1_HUMAN	ABI1	Isoform 4 of Abl interactor 1	0.9993	2.44	1	1	1
Q$7-12	Q8IZP0-5	ABI1_HUMAN	ABI1	Isoform 5 of Abl interactor 1	0.9993	2.31	1	1	1
Q$7-13	Q8IZP0-6	ABI1_HUMAN	ABI1	Isoform 6 of Abl interactor 1	0.9993	2.29	1	1	1
Q$7-14	Q8IZP0-7	ABI1_HUMAN	ABI1	Isoform 7 of Abl interactor 1	0.9993	2.61	1	1	1
Q$7-15	Q8IZP0-8	ABI1_HUMAN	ABI1	Isoform 8 of Abl interactor 1	0.9993	2.8	1	1	1
Q$7-16	Q8IZP0-9	ABI1_HUMAN	ABI1	Isoform 9 of Abl interactor 1	0.9993	2.29	1	1	1
						ALIESYQNLTR	2	0.9969	1
Q$8-1	Q13813-2	SPTN1_HUMAN	SPTAN1	Isoform 2 of Spectrin alpha chain, non-erythrocytic 1	1.0000	2.46	6	6	9
Q$8-2	Q13813-3	SPTN1_HUMAN	SPTAN1	Isoform 3 of Spectrin alpha chain, non-erythrocytic 1	1.0000	2.49	6	6	9
Q$8-3	A6NG51	A6NG51_HUMAN	SPTAN1	Spectrin alpha chain, non-erythrocytic 1	1.0000	2.46	6	6	9
Q$8-4	Q13813	SPTN1_HUMAN	SPTAN1	Spectrin alpha chain, non-erythrocytic 1	1.0000	2.47	6	6	9
						AALLELWELR	2	0.9996	2
						AALLELWELRR	3	0.9578	1
						DVDEIEAWISEK	2	0.9931	1
						ELPTAFDYVEFTR	2	0.9989	3
						LIQSHPESAEDLQEK	2	0.8988	1
						SSEEIESAFR	2	0.8149	1
Q$9-1	A6NKB8	A6NKB8_HUMAN	RNPEP	Aminopeptidase B	1.0000	11.62	6	6	8
						AEFGPPGPGAGSR	2	0.9907	1
						AFELLHLHL	3	0.8938	1
						CLEPEGAAELR	2	0.8422	1
						GLSGTAVLDLR	2	0.9983	2
						ISTILFGAAYTCLEAATGR	3	0.9104	1
						LQVLLTYR	2	0.9681	2
Q$10-1	A8MUS3	A8MUS3_HUMAN	RPL23A	60S ribosomal protein L23a	0.9969	12.89	2	2	2
Q$10-2	K7EMA7	K7EMA7_HUMAN	RPL23A	60S ribosomal protein L23a	0.9969	35.71	2	2	2
Q$10-3	P62750	RL23A_HUMAN	RPL23A	60S ribosomal protein L23a	0.9969	16.03	2	2	2
Q$10-4	H7BY10	H7BY10_HUMAN	RPL23A	60S ribosomal protein L23a (Fragment)	0.9969	15.82	2	2	2
Q$10-5	K7EJV9	K7EJV9_HUMAN	RPL23A	60S ribosomal protein L23a (Fragment)	0.9969	14.71	2	2	2
Q$10-6	K7ERT8	K7ERT8_HUMAN	RPL23A	60S ribosomal protein L23a (Fragment)	0.9969	14.29	2	2	2
						LAPDYDALDVANK	2	0.9543	1
						VNTLIRPDGEKK	2	0.9315	1
Q$11-1	A8MW50	A8MW50_HUMAN	LDHB	L-lactate dehydrogenase (Fragment)	1.0000	10.34	2	2	2
						IVVVTAGVR	2	0.9019	1
						SLADELALVDVLEDK	2	0.9993	1
Q$12-1	A8MX94	A8MX94_HUMAN	GSTP1	Glutathione S-transferase P	1.0000	21.26	4	4	13
						ASCLYGQLPK	2	0.9996	3
						FQDGDLTLYQSNTILR	2	0.9997	6
						PPYTVVYFPVR	2	0.9984	3
						SCLYGQLPK	2	0.983	1
Q$13-1	Q99733-2	NP1L4_HUMAN	NAP1L4	Isoform 2 of Nucleosome assembly protein 1-like 4	1.0000	7.25	2	2	4
Q$13-2	Q99733	NP1L4_HUMAN	NAP1L4	Nucleosome assembly protein 1-like 4	1.0000	7.47	2	2	4
Q$13-3	A8MXH2	A8MXH2_HUMAN	NAP1L4	Nucleosome assembly protein 1-like 4 (Fragment)	1.0000	17.95	2	2	4
Q$13-4	C9JZI7	C9JZI7_HUMAN	NAP1L4	Nucleosome assembly protein 1-like 4 (Fragment)	1.0000	10.07	2	2	4
						FYEEVHDLER	2	0.9994	2
						NVDMLSELVQEYDEPILK	2	0.9987	2
Q$14-1	A8MYE6	A8MYE6_HUMAN	ITGB2	Integrin beta	0.9945	3.37	2	2	2
Q$14-2	P05107	ITB2_HUMAN	ITGB2	Integrin beta-2	0.9945	3.12	2	2	2
						TTEGCLNPR	2	0.9689	1
						VTATECIQEQSFVIR	2	0.8237	1
Q$15-1	B0QY89	B0QY89_HUMAN	EIF3L	Eukaryotic translation initiation factor 3 subunit L	0.9741	2.31	1	1	3
Q$15-2	Q9Y262	EIF3L_HUMAN	EIF3L	Eukaryotic translation initiation factor 3 subunit L	0.9741	2.48	1	1	3
Q$15-3	C9JHP4	C9JHP4_HUMAN	EIF3L	Eukaryotic translation initiation factor 3 subunit L (Fragment	0.9741	9.33	1	1	3
Q$15-4	C9K0Q7	C9K0Q7_HUMAN	EIF3L	Eukaryotic translation initiation factor 3 subunit L (Fragment	0.9741	7.65	1	1	3
Q$15-5	Q9Y262-2	EIF3L_HUMAN	EIF3L	Isoform 2 of Eukaryotic translation initiation factor 3 subunit	0.9741	2.71	1	1	3
						VYEIQDIYENSWTK	2	0.9741	3
Q$16-1	O75477	ERLN1_HUMAN	ERLIN1	Erlin-1	0.9813	3.47	1	1	2
Q$16-2	B0QZ43	B0QZ43_HUMAN	ERLIN1	Erlin-1 (Fragment)	0.9813	4.36	1	1	2
Q$16-3	O94905	ERLN2_HUMAN	ERLIN2	Erlin-2	0.9813	3.54	1	1	2
Q$16-4	E5RHW4	E5RHW4_HUMAN	ERLIN2	Erlin-2 (Fragment)	0.9813	3.55	1	1	2
						ISEIEDAAFLAR	2	0.9813	2
Q$17-1	B0YIW2	B0YIW2_HUMAN	APOC3	Apolipoprotein C-III	0.9819	13.68	1	1	2
Q$17-2	P02656	APOC3_HUMAN	APOC3	Apolipoprotein C-III	0.9819	16.16	1	1	2
						DALSSVQESQVAQQAR	2	0.9819	2
Q$18-1	B0YIW6	B0YIW6_HUMAN	ARCN1	Archain 1, isoform CRA_a	0.9849	2.17	1	1	1
Q$18-2	P48444	COPD_HUMAN	ARCN1	Coatomer subunit delta	0.9849	2.35	1	1	1
						NSNILEDLETLR	2	0.9849	1
Q$19-1	B1AH49	B1AH49_HUMAN	MPST	Sulfurtransferase	1.0000	12.95	2	2	6
						AGQPLQLLDASWYLPK	2	0.9996	4
						ALVSAQWVAEALR	2	0.9997	2
Q$20-1	B1AH78	B1AH78_HUMAN	RAC2	Ras-related C3 botulinum toxin substrate 2 (Fragment)	1.0000	19.88	3	3	6
						CVVVGDGAVGK	2	0.9993	4
						SLVSPASYENVR	2	0.9155	1
						YLECSALTQR	2	0.9867	1
Q$21-1	B1AHC9	B1AHC9_HUMAN	XRCC6	X-ray repair cross-complementing protein 6	1.0000	5.01	2	2	3
						NIYVLQELDNPGAK	2	0.9997	2
						SDSFENPVLQQHFR	2	0.9987	1
Q$22-1	B1AHL2	B1AHL2_HUMAN	FBLN1	Fibulin-1	0.9391	1.39	1	1	2
Q$22-2	F8W7M9	F8W7M9_HUMAN	FBLN1	Fibulin-1	0.9391	1.42	1	1	2
Q$22-3	P23142	FBLN1_HUMAN	FBLN1	Fibulin-1	0.9391	1.42	1	1	2

Q$22-4	P23142-2	FBLN1_HUMAN	FBLN1	Isoform A of Fibulin-1	0.9391	1.77	1	1	2
Q$22-5	P23142-3	FBLN1_HUMAN	FBLN1	Isoform B of Fibulin-1	0.9391	1.66	1	1	2
Q$22-6	P23142-4	FBLN1_HUMAN	FBLN1	Isoform C of Fibulin-1	0.9391	1.46	1	1	2
						GYHLNEEGTR	2	0.9391	2
Q$23-1	B1AKG0	B1AKG0_HUMAN	CFHR1	Complement factor H-related protein 1	1.0000	8.86	2	2	3
Q$23-2	Q03591	FHR1_HUMAN	CFHR1	Complement factor H-related protein 1	1.0000	7.27	2	2	3
						INHGILYDEEK	2	0.9786	1
						ITCTEEGWSPTPK	2	0.9991	2
Q$24-1	B1AKY9	B1AKY9_HUMAN	ATP1A2	Sodium/potassium-transporting ATPase subunit alpha-2	1.0000	2.68	2	2	3
Q$24-2	P50993	AT1A2_HUMAN	ATP1A2	Sodium/potassium-transporting ATPase subunit alpha-2	1.0000	2.65	2	2	3
Q$24-3	H0Y7C1	H0Y7C1_HUMAN	ATP1A2	Sodium/potassium-transporting ATPase subunit alpha-2 (Fra	1.0000	3.78	2	2	3
Q$24-4	P13637-2	AT1A3_HUMAN	ATP1A3	Isoform 2 of Sodium/potassium-transporting ATPase subunit	1.0000	2.64	2	2	3
Q$24-5	P13637-3	AT1A3_HUMAN	ATP1A3	Isoform 3 of Sodium/potassium-transporting ATPase subunit	1.0000	2.63	2	2	3
Q$24-6	M0R116	M0R116_HUMAN	ATP1A3	Sodium/potassium-transporting ATPase subunit alpha-3	1.0000	2.75	2	2	3
Q$24-7	P13637	AT1A3_HUMAN	ATP1A3	Sodium/potassium-transporting ATPase subunit alpha-3	1.0000	2.67	2	2	3
						GVGIISEGNETVEDIAAR	2	0.9996	2
						LIIVEGCQR	2	0.9945	1
Q$25-1	Q13310-2	PABP4_HUMAN	PABPC4	Isoform 2 of Polyadenylate-binding protein 4	0.9999	4.44	2	2	3
Q$25-2	Q13310-3	PABP4_HUMAN	PABPC4	Isoform 3 of Polyadenylate-binding protein 4	0.9999	4.24	2	2	3
Q$25-3	B1ANR0	B1ANR0_HUMAN	PABPC4	Poly(A) binding protein, cytoplasmic 4 (Inducible form), isofo	0.9999	4.55	2	2	3
Q$25-4	Q13310	PABP4_HUMAN	PABPC4	Polyadenylate-binding protein 4	0.9999	4.35	2	2	3
Q$25-5	B1ANR1	B1ANR1_HUMAN	PABPC4	Polyadenylate-binding protein 4 (Fragment)	0.9999	21.71	2	2	3
						FSPAGPVLSIR	2	0.9309	2
						SLGYAYVNFQQPADAER	2	0.9986	1
Q$26-1	B1AP15	B1AP15_HUMAN	CD55	CD55 antigen, decay accelerating factor for complement (Cro	0.9991	9.78	2	2	4
Q$26-2	B1AP13	B1AP13_HUMAN	CD55	Complement decay-accelerating factor	0.9991	6.98	2	2	4
Q$26-3	E9PSH2	E9PSH2_HUMAN	CD55	Complement decay-accelerating factor	0.9991	8.22	2	2	4
Q$26-4	H7BY55	H7BY55_HUMAN	CD55	Complement decay-accelerating factor	0.9991	24.03	2	2	4
Q$26-5	P08174	DAF_HUMAN	CD55	Complement decay-accelerating factor	0.9991	8.14	2	2	4
Q$26-6	H3BLV0	H3BLV0_HUMAN	CD55	Complement decay-accelerating factor (Fragment)	0.9991	9.51	2	2	4
Q$26-7	P08174-2	DAF_HUMAN	CD55	Isoform 1 of Complement decay-accelerating factor	0.9991	7.05	2	2	4
Q$26-8	P08174-3	DAF_HUMAN	CD55	Isoform 3 of Complement decay-accelerating factor	0.9991	8.07	2	2	4
Q$26-9	P08174-4	DAF_HUMAN	CD55	Isoform 4 of Complement decay-accelerating factor	0.9991	8.52	2	2	4
Q$26-10	P08174-5	DAF_HUMAN	CD55	Isoform 5 of Complement decay-accelerating factor	0.9991	7.06	2	2	4
Q$26-11	P08174-6	DAF_HUMAN	CD55	Isoform 6 of Complement decay-accelerating factor	0.9991	5.9	2	2	4
Q$26-12	P08174-7	DAF_HUMAN	CD55	Isoform 7 of Complement decay-accelerating factor	0.9991	5.63	2	2	4
						CEESFVKIPGEKDSVICLK	3	0.994	3
						TSFPEDTVITYK	2	0.8565	1
Q$27-1	P07602-2	SAP_HUMAN	PSAP	Isoform Sap-mu-6 of Prosaposin	1.0000	28.71	11	11	23
Q$27-2	P07602-3	SAP_HUMAN	PSAP	Isoform Sap-mu-9 of Prosaposin	1.0000	28.65	11	11	23
Q$27-3	C9JIZ6	C9JIZ6_HUMAN	PSAP	Prosaposin	1.0000	28.71	11	11	23
Q$27-4	P07602	SAP_HUMAN	PSAP	Prosaposin	1.0000	28.82	11	11	23
Q$27-5	B1AVU8	B1AVU8_HUMAN	PSAP	Saposin-D	1.0000	27.01	11	11	23
						DGGFCEVCKK	2	0.9409	1
						DVYCEVCEFLVK	2	0.8363	1
						EICALVGFCDE	2	0.8518	1
						EIVDSYLPVILDIIK	2	0.9997	3
						GCSFLPDPYQK	2	0.9996	3
						GDVCQDCIQMVTDIQTAVR	2	0.9983	4
						GEMSRPGEVCSALNLCES	2	0.995	3
						KLVGYLDR	2	0.9932	1
						LGPGMADICK	2	0.998	1
						QCDQFVAEYEPVLIEILVEVMDPSFVCLK	3	0.9996	4
						QEILAALEK	2	0.9815	1
Q$28-1	B3KP96	B3KP96_HUMAN	TRIM16	Tripartite motif-containing protein 16	1.0000	8.76	3	3	5
Q$28-2	O95361	TRI16_HUMAN	TRIM16	Tripartite motif-containing protein 16	1.0000	6.74	3	3	5
Q$28-3	J3KPT5	J3KPT5_HUMAN	TRIM16L	Tripartite motif-containing protein 16-like protein	1.0000	9.45	3	3	5
						AVAEMQFGELLAAVR	2	0.9994	1
						FSEPVYAAFWLSK	2	0.9957	2
						SVLVSVSEVK	2	0.9846	2
Q$29-1	B3KPU1	B3KPU1_HUMAN	GNB2	Guanine nucleotide-binding protein G(I)/G(S)/G(T) subunit be	1.0000	24.58	3	3	5
						ADQELLMYSHDNIICGITSVAFSR	3	0.9995	3
						TFVSGACDASIK	2	0.9995	1
						VSCLGVTDDGMAVATGSWDSFLK	2	0.9993	1
Q$30-1	B3KQ59	B3KQ59_HUMAN	RUVBL2	RuvB-like 2	1.0000	6.94	2	2	3
Q$30-2	M0R0Y3	M0R0Y3_HUMAN	RUVBL2	RuvB-like 2	1.0000	8.19	2	2	3
Q$30-3	Q9Y230	RUVB2_HUMAN	RUVBL2	RuvB-like 2	1.0000	6.26	2	2	3
						TQGFLALFSGDTGEIK	2	0.9987	1
						TTEMETIYDLGTK	2	0.9976	2
Q$31-1	B3KR41	B3KR41_HUMAN	MAD1L1	Mitotic spindle assembly checkpoint protein MAD1	0.9979	3.99	2	2	4
Q$31-2	Q9Y6D9	MD1L1_HUMAN	MAD1L1	Mitotic spindle assembly checkpoint protein MAD1	0.9979	3.48	2	2	4
						ETNGLLQEELEGLQR	2	0.9827	2
						LDQTMGLSIR	2	0.9247	2
Q$32-1	B3KSH1	B3KSH1_HUMAN	EIF3F	Eukaryotic translation initiation factor 3 subunit F	1.0000	11.29	3	3	5
Q$32-2	O00303	EIF3F_HUMAN	EIF3F	Eukaryotic translation initiation factor 3 subunit F	1.0000	11.76	3	3	5
						IGVDLIMK	2	0.9969	1
						IQDALSTVLQYAEDVLSGK	3	0.9995	2
						LHPVILASIVDSYER	3	0.9969	2
Q$33-1	P55209-2	NP1L1_HUMAN	NAP1L1	Isoform 2 of Nucleosome assembly protein 1-like 1	1.0000	10.33	3	3	5
Q$33-2	B3KV44	B3KV44_HUMAN	NAP1L1	Nucleosome assembly protein 1-like 1	1.0000	11.76	3	3	5
Q$33-3	B7Z9C2	B7Z9C2_HUMAN	NAP1L1	Nucleosome assembly protein 1-like 1	1.0000	10.89	3	3	5
Q$33-4	F5H4R6	F5H4R6_HUMAN	NAP1L1	Nucleosome assembly protein 1-like 1	1.0000	9.92	3	3	5
Q$33-5	F8VV59	F8VV59_HUMAN	NAP1L1	Nucleosome assembly protein 1-like 1	1.0000	11.62	3	3	5
Q$33-6	F8W0J6	F8W0J6_HUMAN	NAP1L1	Nucleosome assembly protein 1-like 1	1.0000	10.03	3	3	5
Q$33-7	P55209	NP1L1_HUMAN	NAP1L1	Nucleosome assembly protein 1-like 1	1.0000	9.72	3	3	5
Q$33-8	F8VUX1	F8VUX1_HUMAN	NAP1L1	Nucleosome assembly protein 1-like 1 (Fragment)	1.0000	16.03	3	3	5
Q$33-9	F8VXI6	F8VXI6_HUMAN	NAP1L1	Nucleosome assembly protein 1-like 1 (Fragment)	1.0000	17.67	3	3	5
Q$33-10	F8VY35	F8VY35_HUMAN	NAP1L1	Nucleosome assembly protein 1-like 1 (Fragment)	1.0000	14.39	3	3	5
Q$33-11	F8W020	F8W020_HUMAN	NAP1L1	Nucleosome assembly protein 1-like 1 (Fragment)	1.0000	18.36	3	3	5
Q$33-12	F8W118	F8W118_HUMAN	NAP1L1	Nucleosome assembly protein 1-like 1 (Fragment)	1.0000	18.18	3	3	5
Q$33-13	H0YHC3	H0YHC3_HUMAN	NAP1L1	Nucleosome assembly protein 1-like 1 (Fragment)	1.0000	19.19	3	3	5
Q$33-14	H0YIV4	H0YIV4_HUMAN	NAP1L1	Nucleosome assembly protein 1-like 1 (Fragment)	1.0000	9.87	3	3	5
						FYEEVHDLER	2	0.9994	2
						GIPEFWLTVFK	2	0.9985	2
						LDGLVETPTGYIESLPR	2	0.9689	1
Q$34-1	B4DDD6	B4DDD6_HUMAN	DBNL	Drebrin-like protein	1.0000	9.36	2	2	2
Q$34-2	B4DDP6	B4DDP6_HUMAN	DBNL	Drebrin-like protein	1.0000	9.95	2	2	2
Q$34-3	F8WBG8	F8WBG8_HUMAN	DBNL	Drebrin-like protein	1.0000	29.69	2	2	2
Q$34-4	Q9UJU6	DBNL_HUMAN	DBNL	Drebrin-like protein	1.0000	8.84	2	2	2
Q$34-5	Q9UJU6-2	DBNL_HUMAN	DBNL	Isoform 2 of Drebrin-like protein	1.0000	8.82	2	2	2
Q$34-6	Q9UJU6-3	DBNL_HUMAN	DBNL	Isoform 3 of Drebrin-like protein	1.0000	8.66	2	2	2
						SPTDWALFTYEGNSNDIR	2	0.9978	1
						VAGTGEGGLEEMVEELNSGK	2	0.9974	1
Q$35-1	B4DER1	B4DER1_HUMAN	LARS	Leucine--tRNA ligase, cytoplasmic	1.0000	4.79	4	4	9
Q$35-2	B4DJ10	B4DJ10_HUMAN	LARS	Leucine--tRNA ligase, cytoplasmic	1.0000	4.9	4	4	9
Q$35-3	F5H698	F5H698_HUMAN	LARS	Leucine--tRNA ligase, cytoplasmic	1.0000	4.87	4	4	9
Q$35-4	Q9P2J5	SYLC_HUMAN	LARS	Leucine--tRNA ligase, cytoplasmic	1.0000	4.68	4	4	9
						AVLMENIVYLTNSLELEHIEVK	3	0.9973	3
						ILDLQLEFDEK	2	0.9993	2
						QGDNCDSIIR	2	0.9268	1
						VDIGDTIIYLVH	2	0.9997	3
Q$36-1	B4DEW9	B4DEW9_HUMAN	EIF3F	Eukaryotic translation initiation factor 3 subunit F	1.0000	12.98	2	2	3
						IGVDLIMK	2	0.9969	1
						IQDALSTVLQYAEDVLSGK	3	0.9995	2
Q$37-1	P43034-2	LIS1_HUMAN	PAFAH1B1	Isoform 2 of Platelet-activating factor acetylhydrolase IB sub	0.9784	7.48	1	1	3
Q$37-2	B4DF38	B4DF38_HUMAN	PAFAH1B1	Platelet-activating factor acetylhydrolase IB subunit alpha	0.9784	5.37	1	1	3
Q$37-3	P43034	LIS1_HUMAN	PAFAH1B1	Platelet-activating factor acetylhydrolase IB subunit alpha	0.9784	2.68	1	1	3
Q$37-4	I3L3N5	I3L3N5_HUMAN	PAFAH1B1	Platelet-activating factor acetylhydrolase IB subunit alpha (Fr	0.9784	5.29	1	1	3
						LWDFQGFECIR	2	0.9784	3
Q$38-1	B4DFA2	B4DFA2_HUMAN	NSF	Vesicle-fusing ATPase	1.0000	13.66	8	10	13
Q$38-2	I3L0N3	I3L0N3_HUMAN	NSF	Vesicle-fusing ATPase	1.0000	11.91	8	10	13
Q$38-3	P46459	NSF_HUMAN	NSF	Vesicle-fusing ATPase	1.0000	11.83	8	10	13
						GHQLLSADVDIK	2	0.9992	1

						GHQLLSADVDIK	3	0.9946	1
						KAPPQGR	2	0.961	1
						LFGLLVK	2	0.9749	1
						LGANSGLHIIIFDEIDAICK	3	0.9995	3
						LLDYVPIGPR	2	0.8999	1
						LQILHIHTAR	2	0.9959	1
						LQILHIHTAR	3	0.9825	1
						NFSGAELEGLVR	2	0.9979	1
						YVGESEANIR	2	0.9982	2
Q$39-1	B4DFL2	B4DFL2_HUMAN	IDH2	Isocitrate dehydrogenase [NADP]	0.9901	3.25	1	1	1
Q$39-2	B4DSZ6	B4DSZ6_HUMAN	IDH2	Isocitrate dehydrogenase [NADP], mitochondrial	0.9901	4.04	1	1	1
Q$39-3	H0YL11	H0YL11_HUMAN	IDH2	Isocitrate dehydrogenase [NADP], mitochondrial	0.9901	4.17	1	1	1
Q$39-4	P48735	IDHP_HUMAN	IDH2	Isocitrate dehydrogenase [NADP], mitochondrial	0.9901	2.88	1	1	1
						TIEAEAAHGTVTR	2	0.9901	1
Q$40-1	B4DGP8	B4DGP8_HUMAN	CANX	Calnexin	1.0000	4.94	2	2	8
Q$40-2	P27824	CALX_HUMAN	CANX	Calnexin	1.0000	5.24	2	2	8
						APVPTGEVYFADSFDR	2	0.9997	2
						KIPNPDFFEDLEPFR	3	0.9996	6
Q$41-1	O94760-2	DDAH1_HUMAN	DDAH1	Isoform 2 of N(G),N(G)-dimethylarginine dimethylaminohydr	1.0000	4.95	1	1	1
Q$41-2	B4DGT0	B4DGT0_HUMAN	DDAH1	N(G),N(G)-dimethylarginine dimethylaminohydrolase 1	1.0000	4.69	1	1	1
Q$41-3	B4DYP1	B4DYP1_HUMAN	DDAH1	N(G),N(G)-dimethylarginine dimethylaminohydrolase 1	1.0000	4.86	1	1	1
Q$41-4	O94760	DDAH1_HUMAN	DDAH1	N(G),N(G)-dimethylarginine dimethylaminohydrolase 1	1.0000	3.16	1	1	1
						EFFVGLSKR	2	0.9614	1
Q$42-1	B4DIT7	B4DIT7_HUMAN	TGM2	Protein-glutamine gamma-glutamyltransferase 2	1.0000	9.24	4	4	7
Q$42-2	P21980	TGM2_HUMAN	TGM2	Protein-glutamine gamma-glutamyltransferase 2	1.0000	8.15	4	4	7
						ALLVEPVINSYLLAER	2	0.9935	1
						RGQPFWLTLHFEGR	3	0.9995	2
						SEGTYCCGPVPVR	2	0.9988	1
						YLLNLNLEPFSEK	2	0.9993	3
Q$43-1	B4DK69	B4DK69_HUMAN	AKR1C2	Aldo-keto reductase family 1 member C2	1.0000	16.16	3	3	6
						LNDGHFMPVLGFGTYAPAEVPK	3	0.9336	1
						QNVQVFEFQLTSEEMK	2	0.9997	3
						SIGVSNFNHR	2	0.9954	2
Q$44-1	B4DKJ3	B4DKJ3_HUMAN	COMP	Cartilage oligomeric matrix protein	1.0000	3.98	2	2	3
Q$44-2	G3XAP6	G3XAP6_HUMAN	COMP	Cartilage oligomeric matrix protein	1.0000	3.87	2	2	3
Q$44-3	P49747	COMP_HUMAN	COMP	Cartilage oligomeric matrix protein	1.0000	3.7	2	2	3
						DGVGDVCQDDFDADK	2	0.9926	1
						ELQETNAALQDVR	2	0.9995	2
Q$45-1	Q15366-2	PCBP2_HUMAN	PCBP2	Isoform 2 of Poly(rC)-binding protein 2	0.9995	6.56	2	2	3
Q$45-2	Q15366-3	PCBP2_HUMAN	PCBP2	Isoform 3 of Poly(rC)-binding protein 2	0.9995	6.63	2	2	3
Q$45-3	Q15366-4	PCBP2_HUMAN	PCBP2	Isoform 4 of Poly(rC)-binding protein 2	0.9995	7.25	2	2	3
Q$45-4	Q15366-5	PCBP2_HUMAN	PCBP2	Isoform 5 of Poly(rC)-binding protein 2	0.9995	7.16	2	2	3
Q$45-5	Q15366-6	PCBP2_HUMAN	PCBP2	Isoform 6 of Poly(rC)-binding protein 2	0.9995	6.65	2	2	3
Q$45-6	Q15366-7	PCBP2_HUMAN	PCBP2	Isoform 7 of Poly(rC)-binding protein 2	0.9995	7.55	2	2	3
Q$45-7	B4DLC0	B4DLC0_HUMAN	PCBP2	Poly(rC)-binding protein 2	0.9995	7.97	2	2	3
Q$45-8	B4DXP5	B4DXP5_HUMAN	PCBP2	Poly(rC)-binding protein 2	0.9995	7.45	2	2	3
Q$45-9	F8VZX2	F8VZX2_HUMAN	PCBP2	Poly(rC)-binding protein 2	0.9995	7.48	2	2	3
Q$45-10	Q15366	PCBP2_HUMAN	PCBP2	Poly(rC)-binding protein 2	0.9995	6.58	2	2	3
Q$45-11	F8VXH9	F8VXH9_HUMAN	PCBP2	Poly(rC)-binding protein 2 (Fragment)	0.9995	15	2	2	3
Q$45-12	F8W0G4	F8W0G4_HUMAN	PCBP2	Poly(rC)-binding protein 2 (Fragment)	0.9995	15.19	2	2	3
Q$45-13	F8W1G6	F8W1G6_HUMAN	PCBP2	Poly(rC)-binding protein 2 (Fragment)	0.9995	11.88	2	2	3
Q$45-14	H3BRU6	H3BRU6_HUMAN	PCBP2	Poly(rC)-binding protein 2 (Fragment)	0.9995	7.97	2	2	3
						IITLAGPTNAIFK	2	0.9114	1
						INISEGNCPER	2	0.9946	2
Q$46-1	P62491-2	RB11A_HUMAN	RAB11A	Isoform 2 of Ras-related protein Rab-11A	1.0000	34.19	5	5	8
Q$46-2	H3BSC1	H3BSC1_HUMAN	RAB11A	Ras-related protein Rab-11A	1.0000	26.77	5	5	8
Q$46-3	J3KQP6	J3KQP6_HUMAN	RAB11A	Ras-related protein Rab-11A	1.0000	34.19	5	5	8
Q$46-4	P62491	RB11A_HUMAN	RAB11A	Ras-related protein Rab-11A	1.0000	24.54	5	5	8
Q$46-5	H3BMH2	H3BMH2_HUMAN	RAB11A	Ras-related protein Rab-11A (Fragment)	1.0000	34.19	5	5	8
Q$46-6	B4DMK0	B4DMK0_HUMAN	RAB11B	Ras-related protein Rab-11B	1.0000	29.61	5	5	8
Q$46-7	Q15907	RB11B_HUMAN	RAB11B	Ras-related protein Rab-11B	1.0000	24.31	5	5	8
						AQIWDTAGQER	2	0.9969	1
						GAVGALLVYDIAK	2	0.9992	2
						NEFNLESK	2	0.9217	1
						STIGVEFATR	2	0.9985	3
						VVLIGDSGVGK	2	0.9982	1
Q$47-1	B4DN89	B4DN89_HUMAN	SFRS2	Serine/arginine-rich-splicing factor 2	0.9991	11.96	2	2	2
Q$47-2	Q01130	SRSF2_HUMAN	SRSF2	Serine/arginine-rich splicing factor 2	0.9991	11.31	2	2	2
Q$47-3	J3KP15	J3KP15_HUMAN	SRSF2	Serine/arginine-rich splicing factor 2 (Fragment)	0.9991	18.8	2	2	2
Q$47-4	J3QL05	J3QL05_HUMAN	SRSF2	Serine/arginine-rich splicing factor 2 (Fragment)	0.9991	19.23	2	2	2
						DAEDAMDAMDGAVLDGR	2	0.9721	1
						VGDVYIPR	2	0.9789	1
Q$48-1	B4DNW0	B4DNW0_HUMAN	ACY1	Aminoacylase-1	1.0000	13.45	4	5	9
Q$48-2	F8WC59	F8WC59_HUMAN	ACY1	Aminoacylase-1	1.0000	33.33	4	5	9
Q$48-3	Q03154	ACY1_HUMAN	ACY1	Aminoacylase-1	1.0000	16.42	4	5	9
Q$48-4	C9JYZ0	C9JYZ0_HUMAN	ACY1	Aminoacylase-1 (Fragment)	1.0000	29.39	4	5	9
Q$48-5	Q03154-3	ACY1_HUMAN	ACY1	Isoform 3 of Aminoacylase-1	1.0000	19.53	4	5	9
						AGFALDEGIANPTDAFTVFYSER	2	0.9997	2
						AGFALDEGIANPTDAFTVFYSER	3	0.8982	1
						CVSIQYLEAVR	2	0.9892	2
						GPEEEHPSVTLFR	3	0.8485	1
						TVQPKPDYGAAVAFFEETAR	3	0.9991	3
Q$49-1	B4DPJ8	B4DPJ8_HUMAN	CCT6A	T-complex protein 1 subunit zeta	1.0000	15.8	5	5	9
						AQLGVQAFADALLIIPK	2	0.9996	3
						GIDPFSLDALSK	2	0.9945	1
						QADLYISEGLHPR	3	0.9927	1
						VHAELADVLTEAVVDSILAIK	3	0.9967	1
						VLAQNSGFDLQETLVK	2	0.9997	3
Q$50-1	B4DQI4	B4DQI4_HUMAN	ABHD14B	Alpha/beta hydrolase domain-containing protein 14B	1.0000	32.45	6	7	12
Q$50-2	Q96IU4	ABHEB_HUMAN	ABHD14B	Alpha/beta hydrolase domain-containing protein 14B	1.0000	29.05	6	7	12
						AVAIDLPGLGHSK	3	0.9055	1
						EALPGSGQAR	2	0.9926	1
						EGTIQVQGQALFFR	2	0.9994	4
						FSSETWQNLGTLHR	2	0.9797	1
						FSVLLLHGIR	2	0.9996	2
						FSVLLLHGIR	3	0.9984	2
						SVLLLHGIR	2	0.888	1
Q$51-1	Q13595-2	TRA2A_HUMAN	TRA2A	Isoform Short of Transformer-2 protein homolog alpha	1.0000	20.35	2	2	2
Q$51-2	B4DQI6	B4DQI6_HUMAN	TRA2A	Transformer-2 protein homolog alpha	1.0000	12.78	2	2	2
Q$51-3	B4DUA9	B4DUA9_HUMAN	TRA2A	Transformer-2 protein homolog alpha	1.0000	12.71	2	2	2
Q$51-4	Q13595	TRA2A_HUMAN	TRA2A	Transformer-2 protein homolog alpha	1.0000	8.16	2	2	2
						GFAFVYFER	2	0.9996	1
						YGPLSGVNVVYDQR	2	0.9922	1
Q$52-1	B4DQJ8	B4DQJ8_HUMAN	PGD	6-phosphogluconate dehydrogenase, decarboxylating	1.0000	7.87	3	3	5
Q$52-2	P52209	6PGD_HUMAN	PGD	6-phosphogluconate dehydrogenase, decarboxylating	1.0000	7.66	3	3	5
Q$52-3	F5H7U0	F5H7U0_HUMAN	PGD	Uncharacterized protein	1.0000	8.03	3	3	5
						GILFVGSGVSGGEEGAR	2	0.9993	2
						IISYAQGFMLLR	2	0.9993	2
						SFLEDIRK	2	0.9487	1
Q$53-1	B4DQV7	B4DQV7_HUMAN	NPC2	Epididymal secretory protein E1	0.9844	12.8	1	1	3
Q$53-2	E7EMS2	E7EMS2_HUMAN	NPC2	Epididymal secretory protein E1	0.9844	7.96	1	1	3
Q$53-3	G3V3E8	G3V3E8_HUMAN	NPC2	Epididymal secretory protein E1	0.9844	9.2	1	1	3
Q$53-4	J3KMY5	J3KMY5_HUMAN	NPC2	Epididymal secretory protein E1	0.9844	10.81	1	1	3
Q$53-5	P61916	NPC2_HUMAN	NPC2	Epididymal secretory protein E1	0.9844	10.6	1	1	3
Q$53-6	G3V2V8	G3V2V8_HUMAN	NPC2	Epididymal secretory protein E1 (Fragment)	0.9844	13.11	1	1	3
Q$53-7	G3V3D1	G3V3D1_HUMAN	NPC2	Epididymal secretory protein E1 (Fragment)	0.9844	7.24	1	1	3
Q$53-8	H0YIZ1	H0YIZ1_HUMAN	NPC2	Epididymal secretory protein E1 (Fragment)	0.9844	9.25	1	1	3
						EVNVSPCPTQPCQLSK	2	0.9844	3
Q$54-1	B4DQW8	B4DQW8_HUMAN	ANKS1A	Ankyrin repeat and SAM domain-containing protein 1A	0.9819	13.44	1	1	1
Q$54-2	Q92625	ANS1A_HUMAN	ANKS1A	Ankyrin repeat and SAM domain-containing protein 1A	0.9819	3.79	1	1	1
						RLSSGFGGGGGGGSGGGGGGSGGGGGGLGSSSHPLSSLLSMWR	3	0.9736	1
Q$55-1	P57053	H2BFS_HUMAN	H2BFS	Histone H2B type F-S	1.0000	26.98	7	7	11
Q$55-2	P62807	H2B1C_HUMAN	HIST1H2BC	Histone H2B type 1-C/E/F/G/I	1.0000	26.98	7	7	11

Q$55-3	P58876	H2B1D_HUMAN	HIST1H2BD	Histone H2B type 1-D	1.0000	26.98	7	7	11
Q$55-4	Q93079	H2B1H_HUMAN	HIST1H2BH	Histone H2B type 1-H	1.0000	26.98	7	7	11
Q$55-5	O60814	H2B1K_HUMAN	HIST1H2BK	Histone H2B type 1-K	1.0000	26.98	7	7	11
Q$55-6	Q99880	H2B1L_HUMAN	HIST1H2BL	Histone H2B type 1-L	1.0000	26.98	7	7	11
Q$55-7	Q99879	H2B1M_HUMAN	HIST1H2BM	Histone H2B type 1-M	1.0000	26.98	7	7	11
Q$55-8	U3KQK0	U3KQK0_HUMAN	HIST1H2BN	Histone H2B	1.0000	20.48	7	7	11
Q$55-9	Q99877	H2B1N_HUMAN	HIST1H2BN	Histone H2B type 1-N	1.0000	26.98	7	7	11
Q$55-10	B4DR52	B4DR52_HUMAN	HIST2H2BF	Histone H2B	1.0000	20.48	7	7	11
Q$55-11	Q5QNW6	H2B2F_HUMAN	HIST2H2BF	Histone H2B type 2-F	1.0000	26.98	7	7	11
Q$55-12	Q5QNW6-2	H2B2F_HUMAN	HIST2H2BF	Isoform 2 of Histone H2B type 2-F	1.0000	25.37	7	7	11
						AMGIMNSFVNDIFER	2	0.9995	4
						ESYSVYVYK	2	0.8252	1
						KESYSVYVYK	2	0.941	1
						LLLPGELAK	2	0.9986	2
						MNSFVNDIFER	2	0.8689	1
						NSFVNDIFER	2	0.814	1
						SFVNDIFER	2	0.9578	1
Q$56-1	B4DR63	B4DR63_HUMAN	PSMC1	26S protease regulatory subunit 4	0.9999	12.26	3	3	3
Q$56-2	P62191	PRS4_HUMAN	PSMC1	26S protease regulatory subunit 4	0.9999	10.23	3	3	3
						AICTEAGLMALR	2	0.9167	1
						AVANQTSATFLR	2	0.9637	1
						VAEEHAPSIVFIDEIDAIGTK	3	0.9982	1
Q$57-1	Q9Y6E0-2	STK24_HUMAN	STK24	Isoform A of Serine/threonine-protein kinase 24	1.0000	28.07	8	9	17
Q$57-2	Q9Y6E0	STK24_HUMAN	STK24	Serine/threonine-protein kinase 24	1.0000	27.31	8	9	17
Q$57-3	B4DR80	B4DR80_HUMAN	STK24	Serine/threonine-protein kinase 24 12 kDa subunit	1.0000	29.37	8	9	17
						AANVLLSEHGEVK	2	0.9963	1
						ADIWSLGITAIELAR	2	0.9997	3
						ADIWSLGITAIELAR	3	0.9996	2
						GAIYLAEEACPGISDTMVAQLVQR	3	0.9952	2
						LADFGVAGQLTDTQIKR	3	0.9532	1
						RPFSQCLSTIISPLFAELK	3	0.9948	3
						SQACGGNLGSIEELR	2	0.9997	3
						VLFLIPK	2	0.9889	1
						YSLSGGGTSSH	2	0.9992	1
Q$58-1	B4DT28	B4DT28_HUMAN	HNRNPR	Heterogeneous nuclear ribonucleoprotein R	0.9051	2.63	1	1	1
Q$58-2	E7ETM7	E7ETM7_HUMAN	HNRNPR	Heterogeneous nuclear ribonucleoprotein R	0.9051	2.75	1	1	1
Q$58-3	O43390	HNRPR_HUMAN	HNRNPR	Heterogeneous nuclear ribonucleoprotein R	0.9051	2.05	1	1	1
Q$58-4	O43390-2	HNRPR_HUMAN	HNRNPR	Isoform 2 of Heterogeneous nuclear ribonucleoprotein R	0.9051	2.04	1	1	1
Q$58-5	O43390-3	HNRPR_HUMAN	HNRNPR	Isoform 3 of Heterogeneous nuclear ribonucleoprotein R	0.9051	2.18	1	1	1
Q$58-6	O43390-4	HNRPR_HUMAN	HNRNPR	Isoform 4 of Heterogeneous nuclear ribonucleoprotein R	0.9051	2.43	1	1	1
						NLATTVTEEILEK	2	0.9051	1
Q$59-1	B4DTC3	B4DTC3_HUMAN	HNRNPD	Heterogeneous nuclear ribonucleoprotein D0	0.9333	2.64	1	1	1
Q$59-2	Q14103	HNRPD_HUMAN	HNRNPD	Heterogeneous nuclear ribonucleoprotein D0	0.9333	2.25	1	1	1
Q$59-3	D6RAF8	D6RAF8_HUMAN	HNRNPD	Heterogeneous nuclear ribonucleoprotein D0 (Fragment)	0.9333	3.62	1	1	1
Q$59-4	D6RBQ9	D6RBQ9_HUMAN	HNRNPD	Heterogeneous nuclear ribonucleoprotein D0 (Fragment)	0.9333	5.16	1	1	1
Q$59-5	D6RF44	D6RF44_HUMAN	HNRNPD	Heterogeneous nuclear ribonucleoprotein D0 (Fragment)	0.9333	7.21	1	1	1
Q$59-6	H0Y8G5	H0Y8G5_HUMAN	HNRNPD	Heterogeneous nuclear ribonucleoprotein D0 (Fragment)	0.9333	3.08	1	1	1
Q$59-7	H0YA96	H0YA96_HUMAN	HNRNPD	Heterogeneous nuclear ribonucleoprotein D0 (Fragment)	0.9333	3.81	1	1	1
Q$59-8	Q14103-2	HNRPD_HUMAN	HNRNPD	Isoform 2 of Heterogeneous nuclear ribonucleoprotein D0	0.9333	2.38	1	1	1
Q$59-9	Q14103-3	HNRPD_HUMAN	HNRNPD	Isoform 3 of Heterogeneous nuclear ribonucleoprotein D0	0.9333	2.61	1	1	1
Q$59-10	Q14103-4	HNRPD_HUMAN	HNRNPD	Isoform 4 of Heterogeneous nuclear ribonucleoprotein D0	0.9333	2.79	1	1	1
Q$59-11	O14979	HNRDL_HUMAN	HNRNPDL	Heterogeneous nuclear ribonucleoprotein D-like	0.9333	1.9	1	1	1
Q$59-12	O14979-2	HNRDL_HUMAN	HNRNPDL	Isoform 2 of Heterogeneous nuclear ribonucleoprotein D-like	0.9333	2.66	1	1	1
Q$59-13	O14979-3	HNRDL_HUMAN	HNRNPDL	Isoform 3 of Heterogeneous nuclear ribonucleoprotein D-like	0.9333	3.28	1	1	1
						GFGFVLFK	2	0.9333	1
Q$60-1	B4DTG2	B4DTG2_HUMAN	EEF1G	Elongation factor 1-gamma	1.0000	14.78	6	7	11
Q$60-2	P26641	EF1G_HUMAN	EEF1G	Elongation factor 1-gamma	1.0000	16.48	6	7	11
						EYFSWEGAFQHVGK	2	0.9956	2
						GQELAFPLSPDWQVDYESYTWR	2	0.9594	1
						GQELAFPLSPDWQVDYESYTWR	3	0.9128	1
						ILGLLDAYLK	2	0.9957	2
						KLDPGSEETQTLVR	2	0.9991	2
						LDPGSEETQTLVR	2	0.997	2
						WFLTCINQPQFR	2	0.9799	1
Q$61-1	P49368-2	TCPG_HUMAN	CCT3	Isoform 2 of T-complex protein 1 subunit gamma	1.0000	13.21	6	6	9
Q$61-2	B4DUR8	B4DUR8_HUMAN	CCT3	T-complex protein 1 subunit gamma	1.0000	13.4	6	6	9
Q$61-3	P49368	TCPG_HUMAN	CCT3	T-complex protein 1 subunit gamma	1.0000	12.29	6	6	9
						ELGIWEPLAVK	2	0.9965	1
						IDDIVSGHK	2	0.9978	2
						IVLLDSSLEYK	2	0.9251	1
						NLQDAMQVCR	2	0.9889	1
						TLIQNCGASTIR	2	0.9988	2
						WSSLACNIALDAVK	2	0.9994	2
Q$62-1	J3QTR3	J3QTR3_HUMAN	RPS27A	Ubiquitin (Fragment)	1.0000	37.74	4	4	15
Q$62-2	P62979	RS27A_HUMAN	RPS27A	Ubiquitin-40S ribosomal protein S27a	1.0000	25.64	4	4	15
Q$62-3	P62987	RL40_HUMAN	UBA52	Ubiquitin-60S ribosomal protein L40	1.0000	31.25	4	4	15
Q$62-4	P0CG47	UBB_HUMAN	UBB	Polyubiquitin-B	1.0000	17.47	4	4	15
Q$62-5	B4DV12	B4DV12_HUMAN	UBB	Ubiquitin	1.0000	26.14	4	4	15
Q$62-6	J3QKN0	J3QKN0_HUMAN	UBB	Ubiquitin (Fragment)	1.0000	19.42	4	4	15
Q$62-7	J3QS39	J3QS39_HUMAN	UBB	Ubiquitin (Fragment)	1.0000	43.01	4	4	15
Q$62-8	J3QLP7	J3QLP7_HUMAN	UBBP4	Protein UBBP4	1.0000	17.86	4	4	15
Q$62-9	J3QRK5	J3QRK5_HUMAN	UBBP4	Protein UBBP4	1.0000	17.47	4	4	15
Q$62-10	F5H041	F5H041_HUMAN	UBC	Polyubiquitin-C	1.0000	6.57	4	4	15
Q$62-11	P0CG48	UBC_HUMAN	UBC	Polyubiquitin-C	1.0000	5.84	4	4	15
Q$62-12	Q96C32	Q96C32_HUMAN	UBC	Polyubiquitin-C	1.0000	13.11	4	4	15
Q$62-13	F5GXK7	F5GXK7_HUMAN	UBC	Polyubiquitin-C (Fragment)	1.0000	23.67	4	4	15
Q$62-14	F5GYU3	F5GYU3_HUMAN	UBC	Polyubiquitin-C (Fragment)	1.0000	29.85	4	4	15
Q$62-15	F5H265	F5H265_HUMAN	UBC	Polyubiquitin-C (Fragment)	1.0000	26.85	4	4	15
Q$62-16	F5H2Z3	F5H2Z3_HUMAN	UBC	Polyubiquitin-C (Fragment)	1.0000	29.41	4	4	15
Q$62-17	F5H388	F5H388_HUMAN	UBC	Polyubiquitin-C (Fragment)	1.0000	25.81	4	4	15
Q$62-18	F5H6Q2	F5H6Q2_HUMAN	UBC	Polyubiquitin-C (Fragment)	1.0000	32.79	4	4	15
Q$62-19	F5H747	F5H747_HUMAN	UBC	Polyubiquitin-C (Fragment)	1.0000	25	4	4	15
						ESTLHLVLR	2	0.9996	1
						IQDKEGIPPDQQR	2	0.9994	10
						TITLEVEPSDTIENVK	2	0.9996	3
						TITLEVEPSDTIENVKAK	2	0.9915	1
Q$63-1	B4DVY1	B4DVY1_HUMAN	EIF3D	Eukaryotic translation initiation factor 3 subunit D	1.0000	13.83	6	7	11
Q$63-2	O15371	EIF3D_HUMAN	EIF3D	Eukaryotic translation initiation factor 3 subunit D	1.0000	12.59	6	7	11
						IFHTVTTTDDPVIR	3	0.9841	1
						LGDDIDLIVR	2	0.9996	1
						NLAMEATYINHNFSQQCLR	3	0.8839	1
						SVYSWDIVVQR	2	0.9996	2
						VADWTGATYQDKR	2	0.9995	2
						WKLGDDIDLIVR	2	0.9209	1
						WKLGDDIDLIVR	3	0.9971	3
Q$64-1	B4DXW1	B4DXW1_HUMAN	ACTR3	Actin-related protein 3	1.0000	5.72	2	2	3
Q$64-2	F5H3P5	F5H3P5_HUMAN	ACTR3	Actin-related protein 3	1.0000	5.9	2	2	3
						DITYFIQQLLR	2	0.9994	2
						YSYVCPDLVK	2	0.9973	1
Q$65-1	O15523	DDX3Y_HUMAN	DDX3Y	ATP-dependent RNA helicase DDX3Y	1.0000	2.73	2	2	2
Q$65-2	B4DXX7	B4DXX7_HUMAN	DDX3Y	Uncharacterized protein	1.0000	2.74	2	2	2
						GCHLLVATPGR	2	0.999	1
						HAIPIIK	2	0.9983	1
Q$66-1	B4DXY7	B4DXY7_HUMAN	ALDH9A1	4-trimethylaminobutyraldehyde dehydrogenase	0.9981	5.9	2	2	2
Q$66-2	P49189	AL9A1_HUMAN	ALDH9A1	4-trimethylaminobutyraldehyde dehydrogenase	0.9981	5.06	2	2	2
						ANDTTFGLAAGVFTR	2	0.9179	1
						VTIEYYSQLK	2	0.9769	1
Q$67-1	B4DY08	B4DY08_HUMAN	HNRNPC	Heterogeneous nuclear ribonucleoproteins C1/C2	1.0000	20.49	6	6	10
Q$67-2	G3V2Q1	G3V2Q1_HUMAN	HNRNPC	Heterogeneous nuclear ribonucleoproteins C1/C2	1.0000	19.34	6	6	10
Q$67-3	G3V4C1	G3V4C1_HUMAN	HNRNPC	Heterogeneous nuclear ribonucleoproteins C1/C2	1.0000	20.21	6	6	10
Q$67-4	G3V576	G3V576_HUMAN	HNRNPC	Heterogeneous nuclear ribonucleoproteins C1/C2	1.0000	25.54	6	6	10
Q$67-5	P07910	HNRPC_HUMAN	HNRNPC	Heterogeneous nuclear ribonucleoproteins C1/C2	1.0000	19.28	6	6	10
Q$67-6	G3V4W0	G3V4W0_HUMAN	HNRNPC	Heterogeneous nuclear ribonucleoproteins C1/C2 (Fragment)	1.0000	22.52	6	6	10

Q$67-7	P07910-2	HNRPC_HUMAN	HNRNPC	Isoform C1 of Heterogeneous nuclear ribonucleoproteins C1/	1.0000	20.14	6	6	10
						GDDLQAIKK	2	0.9771	1
						GFAFVQYVNER	2	0.9996	2
						LKGDDLQAIKK	2	0.9865	1
						MIAGQVLDINLAAEPK	2	0.9994	3
						SDVEAIFSK	2	0.9715	1
						VFIGNLNTLVVK	2	0.9994	2
Q$68-1	B4DZI8	B4DZI8_HUMAN	COPB2	Coatomer protein complex, subunit beta 2 (Beta prime), isofo	1.0000	5.47	4	4	6
Q$68-2	P35606	COPB2_HUMAN	COPB2	Coatomer subunit beta'	1.0000	5.3	4	4	6
						EAFVVEEWVK	2	0.9635	1
						GSNNVALGYDEGSIIVK	2	0.9989	1
						LESTLNYGMER	2	0.9968	2
						TFEVCDLPVR	2	0.998	2
Q$69-1	P29401-2	TKT_HUMAN	TKT	Isoform 2 of Transketolase	1.0000	22.19	9	10	27
Q$69-2	B4E022	B4E022_HUMAN	TKT	Transketolase	1.0000	24.31	9	10	27
Q$69-3	P29401	TKT_HUMAN	TKT	Transketolase	1.0000	22.47	9	10	27
						AVWAEAGFLAEAELLNLR	2	0.9946	3
						AVWAEAGFLAEAELLNLR	3	0.9835	4
						CEAFGWHAIIVDGHSVEELCK	3	0.9978	3
						ILTVEDHYYEGGIGEAV	2	0.8575	1
						LDNLVAILDINR	2	0.9993	5
						SGKPAELLK	2	0.9967	2
						SKDDQVTVIGAGVTLHEALAAAELLKK	4	0.9351	1
						STFAAFFTR	2	0.8236	1
						TSRPENAIIYNNNEDFQVGQAK	3	0.9979	3
						TVPFCSTFAAFFTR	2	0.9995	4
Q$70-1	Q9P289-2	MST4_HUMAN	MST4	Isoform 2 of Serine/threonine-protein kinase MST4	1.0000	6.49	2	2	2
Q$70-2	B4E0Y9	B4E0Y9_HUMAN	MST4	Serine/threonine-protein kinase MST4	1.0000	5.02	2	2	2
Q$70-3	Q8NBY1	Q8NBY1_HUMAN	MST4	Serine/threonine-protein kinase MST4	1.0000	5.61	2	2	2
Q$70-4	Q9P289	MST4_HUMAN	MST4	Serine/threonine-protein kinase MST4	1.0000	5.29	2	2	2
Q$70-5	O00506-2	STK25_HUMAN	STK25	Isoform 2 of Serine/threonine-protein kinase 25	1.0000	6.3	2	2	2
Q$70-6	O00506-3	STK25_HUMAN	STK25	Isoform 3 of Serine/threonine-protein kinase 25	1.0000	6.63	2	2	2
Q$70-7	O00506	STK25_HUMAN	STK25	Serine/threonine-protein kinase 25	1.0000	5.16	2	2	2
Q$70-8	C9J232	C9J232_HUMAN	STK25	Serine/threonine-protein kinase 25 (Fragment)	1.0000	12.43	2	2	2
Q$70-9	C9J6L2	C9J6L2_HUMAN	STK25	Serine/threonine-protein kinase 25 (Fragment)	1.0000	13.66	2	2	2
Q$70-10	C9JCC0	C9JCC0_HUMAN	STK25	Serine/threonine-protein kinase 25 (Fragment)	1.0000	13.5	2	2	2
Q$70-11	C9JDH9	C9JDH9_HUMAN	STK25	Serine/threonine-protein kinase 25 (Fragment)	1.0000	14.86	2	2	2
Q$70-12	H7C279	H7C279_HUMAN	STK25	Serine/threonine-protein kinase 25 (Fragment)	1.0000	8.91	2	2	2
						ADIWSLGITAIELAK	2	0.9973	1
						VLFLIPK	2	0.9889	1
Q$71-1	B4E1F0	B4E1F0_HUMAN	SERPING1	Plasma protease C1 inhibitor	1.0000	22.18	12	13	29
Q$71-2	B4E1H2	B4E1H2_HUMAN	SERPING1	Plasma protease C1 inhibitor	1.0000	25	12	13	29
Q$71-3	P05155	IC1_HUMAN	SERPING1	Plasma protease C1 inhibitor	1.0000	22.4	12	13	29
						DFTCVHQALK	2	0.9995	1
						FQPTLLTLPR	2	0.9458	1
						GVTSVSQIFHSPDLAIR	2	0.9997	3
						GVTSVSQIFHSPDLAIR	3	0.995	1
						HRLEDMEQALSPSVFK	3	0.9135	1
						LLDSLPSDTR	2	0.9988	4
						LVLLNAIYLSAK	2	0.9996	3
						TLLVFEVQQPF	2	0.9254	1
						TLLVFEVQQPFL	2	0.9916	2
						TLLVFEVQQPFLF	2	0.9859	3
						TNLESILSYPK	2	0.9995	4
						TNLESILSYPKDFTCVHQALK	3	0.9887	2
						VTTSQDMLSIMEK	2	0.9994	3
Q$72-1	Q02241-2	KIF23_HUMAN	KIF23	Isoform 2 of Kinesin-like protein KIF23	0.9064	2.34	1	1	2
Q$72-2	B4E1K0	B4E1K0_HUMAN	KIF23	Kinesin-like protein KIF23	0.9064	2.97	1	1	2
Q$72-3	H7BYN4	H7BYN4_HUMAN	KIF23	Kinesin-like protein KIF23	0.9064	2.1	1	1	2
Q$72-4	Q02241	KIF23_HUMAN	KIF23	Kinesin-like protein KIF23	0.9064	2.08	1	1	2
						MIVCVNPKAEDYEENLQVMR	2	0.9064	2
Q$73-1	B4E1Z4	B4E1Z4_HUMAN	CFB	Complement factor B	1.0000	34.83	40	44	91
						AIHCPRPHDFENGEYWPR	2	0.9986	1
						AIHCPRPHDFENGEYWPR	3	0.9985	4
						AIHCPRPHDFENGEYWPR	4	0.9981	2
						ALFVSEEEK	2	0.9972	2
						ALFVSEEEKK	2	0.9993	2
						ALRLPPTTTCQQQKEELLPAQDIK	3	0.9992	3
						ARPQGSCSLEGVEIK	2	0.9387	2
						AVISPGFDVFAK	2	0.9893	2
						CLPCTEGTTR	2	0.8794	1
						CLVNLIEK	2	0.9935	2
						CSSNLVLTGSSER	2	0.9988	3
						DAQYAPGYDKVK	2	0.9964	3
						DFHINLFQVLPWLK	3	0.9971	1
						DISEVVTPR	2	0.9996	2
						DLEIEVVLFHPNY	2	0.8443	1
						DLLYIGKDR	2	0.9977	2
						DNEQHVFK	2	0.9854	1
						EAGIPEFYDYDVALIK	2	0.9997	5
						EAGIPEFYDYDVALIK	3	0.9453	1
						EDYLDVYVFGVGPLVNQVNINALASK	3	0.9962	2
						EDYLDVYVFGVGPLVNQVNINALASKK	3	0.9979	2
						FIQVGVISWGVVDVCK	2	0.8418	1
						FLCTGGVSPYADPNTCR	2	0.9997	3
						GDSGGPLIVHK	2	0.9989	2
						GDSGGPLIVHKR	2	0.9995	4
						GLVTYATYPK	2	0.9551	1
						HAFILQDTK	2	0.9986	3
						HAIILLTDGK	2	0.9971	1
						KNQGILEFYGDDIALLK	3	0.9966	2
						LEDSVTYHCSR	2	0.9996	3
						LLGMETMAWQEIR	2	0.9996	2
						LLQEGQALEYVCPSGFYPYPVQTR	3	0.9996	2
						NDYLDIYAIGVGK	2	0.9944	1
						NPREDYLDVYVFGVGPLVNQVNINALASK	3	0.9975	2
						QLNEINYEDHK	2	0.9988	1
						QLNEINYEDHKLK	2	0.9609	2
						SLARPQGSCSLEGVEIK	3	0.912	1
						SSGQWQTPGATR	2	0.9516	1
						STGSWSTLK	2	0.9813	1
						VASYGVKPR	2	0.9997	1
						VSEADSSNADWVTK	2	0.9997	4
						YGLVTYATYPK	2	0.9997	4
						YGQTIRPICLPCTEGTTR	2	0.9993	3
						YGQTIRPICLPCTEGTTR	3	0.9993	2
Q$74-1	P84103	SRSF3_HUMAN	SRSF3	Serine/arginine-rich splicing factor 3	1.0000	23.17	4	5	12
Q$74-2	B4E241	B4E241_HUMAN	SRSF3	Serine/arginine-rich-splicing factor 3	1.0000	30.65	4	5	12
						AFGYYGPLR	2	0.9997	3
						DSCPLDCK	2	0.9938	1
						NPPGFAFVEFEDPR	2	0.9997	3
						NPPGFAFVEFEDPR	3	0.9993	2
						NPPGFAFVEFEDPRDAADAVR	3	0.9981	3
Q$75-1	O75487	GPC4_HUMAN	GPC4	Glypican-4	0.9999	14.21	5	5	5
Q$75-2	B4E2C0	B4E2C0_HUMAN	GPC4	Secreted glypican-4	0.9999	16.26	5	5	5
						KFWSSLPSNVCNDER	3	0.9131	1
						LEGPFNIESVMDPIDVK	2	0.9897	1
						VFQGCGPPKPLPAG	2	0.8852	1
						VFQGCGPPKPLPAGR	2	0.9944	1
						YLFAVTGNGLANQGNNPEVQVDTSKPDILILR	3	0.8767	1
Q$76-1	B4E2Q4	B4E2Q4_HUMAN	EIF3M	Eukaryotic translation initiation factor 3 subunit M	0.9428	4.96	1	1	1
Q$76-2	Q7L2H7	EIF3M_HUMAN	EIF3M	Eukaryotic translation initiation factor 3 subunit M	0.9428	3.21	1	1	1
Q$76-3	H0YCQ8	H0YCQ8_HUMAN	EIF3M	Eukaryotic translation initiation factor 3 subunit M (Fragmen	0.9428	5.45	1	1	1

							LLTFMGMAVENK	2	0.9428	1
Q$77-1	B4E363	B4E363_HUMAN	FARSA	Phenylalanine--tRNA ligase alpha subunit	1.0000		2.94	1	2	5
Q$77-2	K7ER00	K7ER00_HUMAN	FARSA	Phenylalanine--tRNA ligase alpha subunit	1.0000		2.55	1	2	5
Q$77-3	K7ER16	K7ER16_HUMAN	FARSA	Phenylalanine--tRNA ligase alpha subunit	1.0000		5.96	1	2	5
Q$77-4	Q9Y285	SYFA_HUMAN	FARSA	Phenylalanine--tRNA ligase alpha subunit	1.0000		2.76	1	2	5
Q$77-5	K7EK06	K7EK06_HUMAN	FARSA	Phenylalanine--tRNA ligase alpha subunit (Fragment)	1.0000		8.33	1	2	5
							SLQALGEVIEAELR	2	0.9996	3
							SLQALGEVIEAELR	3	0.991	2
Q$78-1	B5MBZ0	B5MBZ0_HUMAN	EML4	Echinoderm microtubule-associated protein-like 4	0.9999		1.71	2	2	3
Q$78-2	Q9HC35	EMAL4_HUMAN	EML4	Echinoderm microtubule-associated protein-like 4	0.9999		1.73	2	2	3
Q$78-3	Q9HC35-2	EMAL4_HUMAN	EML4	Isoform 2 of Echinoderm microtubule-associated protein-like	0.9999		1.84	2	2	3
							AALADVLR	2	0.9867	1
							WFVLDAETR	2	0.9937	2
Q$79-1	Q15435-2	PP1R7_HUMAN	PPP1R7	Isoform 2 of Protein phosphatase 1 regulatory subunit 7	0.9489		3.47	1	1	1
Q$79-2	Q15435-3	PP1R7_HUMAN	PPP1R7	Isoform 3 of Protein phosphatase 1 regulatory subunit 7	0.9489		3.93	1	1	1
Q$79-3	Q15435-4	PP1R7_HUMAN	PPP1R7	Isoform 4 of Protein phosphatase 1 regulatory subunit 7	0.9489		4.64	1	1	1
Q$79-4	B5MBZ8	B5MBZ8_HUMAN	PPP1R7	Protein phosphatase 1 regulatory subunit 7	0.9489		4.01	1	1	1
Q$79-5	B5MCY6	B5MCY6_HUMAN	PPP1R7	Protein phosphatase 1 regulatory subunit 7	0.9489		4.98	1	1	1
Q$79-6	Q15435	PP1R7_HUMAN	PPP1R7	Protein phosphatase 1 regulatory subunit 7	0.9489		3.06	1	1	1
Q$79-7	C9J177	C9J177_HUMAN	PPP1R7	Protein phosphatase 1 regulatory subunit 7 (Fragment)	0.9489		3.81	1	1	1
Q$79-8	C9JD73	C9JD73_HUMAN	PPP1R7	Protein phosphatase 1 regulatory subunit 7 (Fragment)	0.9489		3.9	1	1	1
Q$79-9	H7C003	H7C003_HUMAN	PPP1R7	Protein phosphatase 1 regulatory subunit 7 (Fragment)	0.9489		3.28	1	1	1
							IEGLQNLVNLR	2	0.9489	1
Q$80-1	B5MC07	B5MC07_HUMAN	AGR2	Anterior gradient protein 2 homolog	1.0000		24.47	4	6	11
Q$80-2	C9J3E2	C9J3E2_HUMAN	AGR2	Anterior gradient protein 2 homolog (Fragment)	1.0000		35.11	4	6	11
							GWGDQLIWTQTYEEALYK	2	0.9997	3
							HLSPDGQYVPR	2	0.9917	1
							LAEQFVLLNLVYETTDK	2	0.9997	3
							LAEQFVLLNLVYETTDK	3	0.9995	2
							LAEQFVLLNLVYETTDKHLSPDGQYVPR	3	0.9737	1
							LAEQFVLLNLVYETTDKHLSPDGQYVPR	4	0.987	1
Q$81-1	P22309-2	UD11_HUMAN	UGT1A1	Isoform 2 of UDP-glucuronosyltransferase 1-1	1.0000		4.73	1	1	2
Q$81-2	P22309	UD11_HUMAN	UGT1A1	UDP-glucuronosyltransferase 1-1	1.0000		3.94	1	1	2
Q$81-3	Q9HAW8-2	UD110_HUMAN	UGT1A10	Isoform 2 of UDP-glucuronosyltransferase 1-10	1.0000		4.76	1	1	2
Q$81-4	Q9HAW8	UD110_HUMAN	UGT1A10	UDP-glucuronosyltransferase 1-10	1.0000		3.96	1	1	2
Q$81-5	P35503-3	UD13_HUMAN	UGT1A3	Isoform 2 of UDP-glucuronosyltransferase 1-3	1.0000		4.72	1	1	2
Q$81-6	P35503	UD13_HUMAN	UGT1A3	UDP-glucuronosyltransferase 1-3	1.0000		3.93	1	1	2
Q$81-7	P22310-2	UD14_HUMAN	UGT1A4	Isoform 2 of UDP-glucuronosyltransferase 1-4	1.0000		4.72	1	1	2
Q$81-8	P22310	UD14_HUMAN	UGT1A4	UDP-glucuronosyltransferase 1-4	1.0000		3.93	1	1	2
Q$81-9	P35504-2	UD15_HUMAN	UGT1A5	Isoform 2 of UDP-glucuronosyltransferase 1-5	1.0000		4.72	1	1	2
Q$81-10	P35504	UD15_HUMAN	UGT1A5	UDP-glucuronosyltransferase 1-5	1.0000		3.93	1	1	2
Q$81-11	P19224-2	UD16_HUMAN	UGT1A6	Isoform 2 of UDP-glucuronosyltransferase 1-6	1.0000		7.92	1	1	2
Q$81-12	B5MCT4	B5MCT4_HUMAN	UGT1A6	UDP-glucuronosyltransferase 1-6	1.0000		11.93	1	1	2
Q$81-13	Q9HAW7-2	UD17_HUMAN	UGT1A7	Isoform 2 of UDP-glucuronosyltransferase 1-7	1.0000		4.76	1	1	2
Q$81-14	Q9HAW7	UD17_HUMAN	UGT1A7	UDP-glucuronosyltransferase 1-7	1.0000		3.96	1	1	2
Q$81-15	Q9HAW9-2	UD18_HUMAN	UGT1A8	Isoform 2 of UDP-glucuronosyltransferase 1-8	1.0000		4.76	1	1	2
Q$81-16	Q9HAW9	UD18_HUMAN	UGT1A8	UDP-glucuronosyltransferase 1-8	1.0000		3.96	1	1	2
Q$81-17	O60656-2	UD19_HUMAN	UGT1A9	Isoform 2 of UDP-glucuronosyltransferase 1-9	1.0000		4.76	1	1	2
Q$81-18	O60656	UD19_HUMAN	UGT1A9	UDP-glucuronosyltransferase 1-9	1.0000		3.96	1	1	2
							GAGVTLNVLEMTSEDLENALK	2	0.9993	2
Q$82-1	Q15019-2	SEPT2_HUMAN	SEPT2	Isoform 2 of Septin-2	1.0000		7.07	2	2	3
Q$82-2	Q15019-3	SEPT2_HUMAN	SEPT2	Isoform 3 of Septin-2	1.0000		7.55	2	2	3
Q$82-3	B5MCX3	B5MCX3_HUMAN	SEPT2	Septin-2	1.0000		8.72	2	2	3
Q$82-4	Q15019	SEPT2_HUMAN	SEPT2	Septin-2	1.0000		7.76	2	2	3
Q$82-5	C9IY94	C9IY94_HUMAN	SEPT2	Septin-2 (Fragment)	1.0000		23.14	2	2	3
Q$82-6	C9IZU3	C9IZU3_HUMAN	SEPT2	Septin-2 (Fragment)	1.0000		21.37	2	2	3
Q$82-7	C9J2Q4	C9J2Q4_HUMAN	SEPT2	Septin-2 (Fragment)	1.0000		15.22	2	2	3
Q$82-8	C9J938	C9J938_HUMAN	SEPT2	Septin-2 (Fragment)	1.0000		18.92	2	2	3
Q$82-9	C9JB25	C9JB25_HUMAN	SEPT2	Septin-2 (Fragment)	1.0000		16.18	2	2	3
Q$82-10	C9JQJ4	C9JQJ4_HUMAN	SEPT2	Septin-2 (Fragment)	1.0000		19.05	2	2	3
Q$82-11	H7C310	H7C310_HUMAN	SEPT2	Septin-2 (Fragment)	1.0000		17.39	2	2	3
							LTVVDTPGYGDAINCR	2	0.9991	1
							TIISYIDEQFER	2	0.9996	2
Q$83-1	B5MD38	B5MD38_HUMAN	HADHB	3-ketoacyl-CoA thiolase	0.9998		4.56	1	1	1
Q$83-2	C9JE81	C9JE81_HUMAN	HADHB	Trifunctional enzyme subunit beta, mitochondrial (Fragment)	0.9998		9.14	1	1	1
Q$83-3	C9JEY0	C9JEY0_HUMAN	HADHB	Trifunctional enzyme subunit beta, mitochondrial (Fragment)	0.9998		10.74	1	1	1
							EVVDYIIFGTVIQEVK	2	0.9932	1
Q$84-1	B5MDF5	B5MDF5_HUMAN	RAN	GTP-binding nuclear protein Ran	0.9860		10.3	2	2	2
Q$84-2	P62826	RAN_HUMAN	RAN	GTP-binding nuclear protein Ran	0.9860		11.11	2	2	2
Q$84-3	J3KQE5	J3KQE5_HUMAN	RAN	GTP-binding nuclear protein Ran (Fragment)	0.9860		10.26	2	2	2
							LVLVGDGGTGK	2	0.8895	1
							VCENIPIVLCGNK	2	0.8728	1
Q$85-1	H3BRV0	H3BRV0_HUMAN	EIF3C	Eukaryotic translation initiation factor 3 subunit C	1.0000		5.09	4	4	7
Q$85-2	Q99613	EIF3C_HUMAN	EIF3C	Eukaryotic translation initiation factor 3 subunit C	1.0000		5.04	4	4	7
Q$85-3	B5ME19	EIFCL_HUMAN	EIF3CL	Eukaryotic translation initiation factor 3 subunit C-like protei	1.0000		5.03	4	4	7
							GCILTLVER	2	0.9993	2
							LGSLVENNER	2	0.9924	2
							TEPTAQQNLALQLAEK	2	0.9992	1
							TMVQLGICAFR	2	0.9996	2
Q$86-1	B5ME49	B5ME49_HUMAN	MUC16	Mucin-16	0.9815		0.21	2	2	2
Q$86-2	Q8WXI7	MUC16_HUMAN	MUC16	Mucin-16	0.9815		0.14	2	2	2
							SISGSAESTMLPEISTTR	2	0.8521	1
							VPGVVTSLVTSSR	2	0.84	1
Q$87-1	B7WPK3	B7WPK3_HUMAN	FTCD	Formimidoyltransferase-cyclodeaminase	1.0000		19.8	5	6	8
Q$87-2	O95954	FTCD_HUMAN	FTCD	Formimidoyltransferase-cyclodeaminase	1.0000		18.11	5	6	8
Q$87-3	O95954-3	FTCD_HUMAN	FTCD	Isoform D of Formimidoyltransferase-cyclodeaminase	1.0000		19.8	5	6	8
							GVSVDECVLCAQAFGQR	2	0.9985	2
							LAEELDVPVYLYGEAAR	2	0.9967	1
							LGLDSLCPFSPK	2	0.9797	1
							NQEVIDAISGAITQTPGCVLLDVDAGPSTNR	3	0.9664	1
							TVYTFVGPPECVVEGALNAAR	2	0.9984	2
							TVYTFVGPPECVVEGALNAAR	3	0.9894	1
Q$88-1	B7Z1C9	B7Z1C9_HUMAN	CCT7	Chaperonin containing TCP1, subunit 7 (Eta), isoform CRA_a	1.0000		16.87	5	5	8
Q$88-2	Q99832-2	TCPH_HUMAN	CCT7	Isoform 2 of T-complex protein 1 subunit eta	1.0000		20.65	5	5	8
Q$88-3	Q99832-4	TCPH_HUMAN	CCT7	Isoform 4 of T-complex protein 1 subunit eta	1.0000		15.35	5	5	8
Q$88-4	F5GZK5	F5GZK5_HUMAN	CCT7	T-complex protein 1 subunit eta	1.0000		15.8	5	5	8
							CQVFEETQIGGER	2	0.9996	2
							GGAEQFMEETER	2	0.9606	1
							IALLNVELELK	2	0.9824	1
							INALTAASEAACLIVSVDETIKNPR	3	0.9993	3
							YNFFTGCPK	2	0.9076	1
Q$89-1	B7Z1K0	B7Z1K0_HUMAN	BAI3	Brain-specific angiogenesis inhibitor 3	0.9818		6.17	2	2	2
Q$89-2	J3KMY8	J3KMY8_HUMAN	BAI3	Brain-specific angiogenesis inhibitor 3	0.9818		4.12	2	2	2
Q$89-3	O60242	BAI3_HUMAN	BAI3	Brain-specific angiogenesis inhibitor 3	0.9818		1.97	2	2	2
							DSLQGFVIVMVHCILR	2	0.843	1
							SETGSTISMSSLER	2	0.8839	1
Q$90-1	B7Z1R5	B7Z1R5_HUMAN	ATP6V1A	V-type proton ATPase catalytic subunit A	1.0000		7.36	3	3	4
Q$90-2	P38606	VATA_HUMAN	ATP6V1A	V-type proton ATPase catalytic subunit A	1.0000		6.97	3	3	4
Q$90-3	C9JA17	C9JA17_HUMAN	ATP6V1A	V-type proton ATPase catalytic subunit A (Fragment)	1.0000		23.37	3	3	4
Q$90-4	C9JVW8	C9JVW8_HUMAN	ATP6V1A	V-type proton ATPase catalytic subunit A (Fragment)	1.0000		19.46	3	3	4
							FTMVQVWPVR	2	0.9988	2
							VGHSELVGEIIR	3	0.9702	1
							VGSHITGGDIYGIVSENSLIK	3	0.9908	1
Q$91-1	B7Z2F4	B7Z2F4_HUMAN	CCT4	T-complex protein 1 subunit delta	1.0000		15.42	4	5	9
							ALIAGGGAPEIELALR	2	0.9996	3
							IGLIQFCLSAPK	2	0.9995	2
							LGGTIDDCELVEGLVLTQK	2	0.9992	1
							LGGTIDDCELVEGLVLTQK	3	0.9675	2
							VIDPATATSVDLR	2	0.9966	1
Q$92-1	B7Z3Y2	B7Z3Y2_HUMAN	PCYOX1	Prenylcysteine oxidase 1	0.9622		3.04	1	1	1
Q$92-2	B7Z9P8	B7Z9P8_HUMAN	PCYOX1	Prenylcysteine oxidase 1	0.9622		3.04	1	1	1
Q$92-3	Q9UHG3	PCYOX_HUMAN	PCYOX1	Prenylcysteine oxidase 1	0.9622		2.57	1	1	1
							MYEVVYQIGTETR	2	0.9622	1

Q$93-1	B7Z413	B7Z413_HUMAN	ATP6AP2	Renin receptor	1.0000	34.31	6	7	13
Q$93-2	B7Z9I3	B7Z9I3_HUMAN	ATP6AP2	Renin receptor	1.0000	29.56	6	7	13
Q$93-3	O75787	RENR_HUMAN	ATP6AP2	Renin receptor	1.0000	26.86	6	7	13
Q$93-4	H7C3E1	H7C3E1_HUMAN	ATP6AP2	Renin receptor (Fragment)	1.0000	36.29	6	7	13
						ANSVFEDLSVTLR	2	0.9996	3
						DHSPDLYSLELAGLDEIGKR	3	0.8415	1
						FADDMYSLYGGNAVVELVTVK	2	0.9995	3
						FADDMYSLYGGNAVVELVTVK	3	0.9959	1
						ILVDALQK	2	0.9887	1
						NNEVDLLFLSELQVLHDISSLLSR	3	0.9988	3
						SFDTSLIR	2	0.983	1
Q$94-1	B7Z4K6	B7Z4K6_HUMAN	DNASE2	Deoxyribonuclease-2-alpha	1.0000	9.84	2	2	3
Q$94-2	O00115	DNS2A_HUMAN	DNASE2	Deoxyribonuclease-2-alpha	1.0000	8.33	2	2	3
Q$94-3	K7ENE5	K7ENE5_HUMAN	DNASE2	Deoxyribonuclease-2-alpha (Fragment)	1.0000	13.76	2	2	3
						ALINSPEGAVGR	2	0.9992	1
						LTCYGDSGQPVDWFVVYK	2	0.9851	2
Q$95-1	B7Z4L4	B7Z4L4_HUMAN	RPN1	Dolichyl-diphosphooligosaccharide--protein glycosyltransfera	0.9834	2.53	1	1	1
Q$95-2	P04843	RPN1_HUMAN	RPN1	Dolichyl-diphosphooligosaccharide--protein glycosyltransfera	0.9834	1.81	1	1	1
						SEDLLDYGPFR	2	0.9728	1
Q$96-1	B7Z5C0	B7Z5C0_HUMAN	DNAJA1	DnaJ homolog subfamily A member 1	0.9794	5.42	1	1	2
Q$96-2	P31689	DNJA1_HUMAN	DNAJA1	DnaJ homolog subfamily A member 1	0.9794	3.27	1	1	2
Q$96-3	P31689-2	DNJA1_HUMAN	DNAJA1	Isoform 2 of DnaJ homolog subfamily A member 1	0.9794	3.93	1	1	2
						TIVITSHPGQIVK	2	0.9794	2
Q$97-1	B7Z5N8	B7Z5N8_HUMAN	HOXB3	Homeobox protein Hox-B3	0.9199	15.38	1	1	1
Q$97-2	B7ZAD0	B7ZAD0_HUMAN	HOXB3	Homeobox protein Hox-B3	0.9199	12.85	1	1	1
Q$97-3	P14651	HXB3_HUMAN	HOXB3	Homeobox protein Hox-B3	0.9199	10.67	1	1	1
						NNSPGTAEGCGGGGGGGGGGGSGGSGGGGGGGGGGDKSPPGSAASK	3	0.9199	1
Q$98-1	B7Z6Z4	B7Z6Z4_HUMAN	MYL6	Myosin light polypeptide 6	1.0000	18.07	4	4	7
Q$98-2	F8W1R7	F8W1R7_HUMAN	MYL6	Myosin light polypeptide 6	1.0000	29.66	4	4	7
Q$98-3	J3KND3	J3KND3_HUMAN	MYL6	Myosin light polypeptide 6	1.0000	28.29	4	4	7
Q$98-4	P60660	MYL6_HUMAN	MYL6	Myosin light polypeptide 6	1.0000	28.48	4	4	7
						ALGQNPTNAEVLK	2	0.9994	2
						DQGTYEDYVEGLR	2	0.9909	2
						EAFQLFDR	2	0.9981	2
						HVLVTLGEK	2	0.9993	1
Q$99-1	B7Z7A9	B7Z7A9_HUMAN	PGK1	Phosphoglycerate kinase	1.0000	42.67	14	17	37
Q$99-2	P00558	PGK1_HUMAN	PGK1	Phosphoglycerate kinase 1	1.0000	39.81	14	17	37
						ACANPAAGSVILLENLR	2	0.9997	4
						ACANPAAGSVILLENLR	3	0.9994	3
						AEPAKIEAFR	2	0.9662	1
						ALESPERPFL	2	0.9618	2
						ALESPERPFLAILGGAK	3	0.9995	4
						ALMDEVVK	2	0.9802	1
						GCITIIGGGDTATCCAK	2	0.9997	5
						GPVGVFEWEAFAR	2	0.8619	2
						LGDVYVNDAFGTAHR	2	0.9933	1
						LGDVYVNDAFGTAHR	3	0.9926	1
						QIVWNGPVGVFEWEAFAR	2	0.9997	5
						QIVWNGPVGVFEWEAFAR	3	0.9942	2
						SLLGKDVLFLK	3	0.9909	2
						VLNNMEIGTSLFDEEGAK	2	0.9984	1
						VLPGVDALSNI	2	0.9986	1
						VSHVSTGGGASLELLEGK	3	0.9957	1
						WNTEDKVSHVSTGGGASLELLEGK	3	0.8025	1
Q$100-1	B7Z8M7	B7Z8M7_HUMAN	RAB1A	Ras-related protein Rab-1A	1.0000	26.59	3	3	6
Q$100-2	E9PLD0	E9PLD0_HUMAN	RAB1B	Ras-related protein Rab-1B	1.0000	27.22	3	3	6
Q$100-3	Q9H0U4	RAB1B_HUMAN	RAB1B	Ras-related protein Rab-1B	1.0000	22.89	3	3	6
						EFADSLGIPFLETSAK	2	0.9674	2
						FADDTYTESYISTIGVDFK	2	0.9994	2
						LLLIGDSGVGK	2	0.985	2
Q$101-1	B7Z8Q5	B7Z8Q5_HUMAN	HABP2	Hyaluronan-binding protein 2 50 kDa heavy chain	1.0000	11.07	2	2	4
						FCEIGSDDCYVGDGYSYR	2	0.9996	1
						GQCLITQSPPYYR	2	0.9942	3
Q$102-1	B7Z909	B7Z909_HUMAN	HYOU1	Hypoxia up-regulated protein 1	0.9304	2.33	1	1	1
Q$102-2	E9PL22	E9PL22_HUMAN	HYOU1	Hypoxia up-regulated protein 1	0.9304	1.39	1	1	1
Q$102-3	Q9Y4L1	HYOU1_HUMAN	HYOU1	Hypoxia up-regulated protein 1	0.9304	1.3	1	1	1
Q$102-4	E9PJ21	E9PJ21_HUMAN	HYOU1	Hypoxia up-regulated protein 1 (Fragment)	0.9304	1.98	1	1	1
						VEFEELCADLFER	2	0.9304	1
Q$103-1	P50991-2	TCPD_HUMAN	CCT4	Isoform 2 of T-complex protein 1 subunit delta	1.0000	14.34	5	7	12
Q$103-2	B7Z9L0	B7Z9L0_HUMAN	CCT4	T-complex protein 1 subunit delta	1.0000	15.11	5	7	12
Q$103-3	P50991	TCPD_HUMAN	CCT4	T-complex protein 1 subunit delta	1.0000	13.54	5	7	12
						ALIAGGGAPEIELALR	2	0.9996	3
						GIHPTIISESFQK	2	0.9916	2
						GIHPTIISESFQK	3	0.9302	1
						IGLIQFCLSAPK	2	0.9995	2
						LGGTIDDCELVEGLVLTQK	2	0.9992	1
						LGGTIDDCELVEGLVLTQK	3	0.9675	2
						VIDPATATSVDLR	2	0.9966	1
Q$104-1	P48643-2	TCPE_HUMAN	CCT5	Isoform 2 of T-complex protein 1 subunit epsilon	1.0000	6.25	2	2	2
Q$104-2	B7ZAR1	B7ZAR1_HUMAN	CCT5	T-complex protein 1 subunit epsilon	1.0000	5.57	2	2	2
Q$104-3	E7ENZ3	E7ENZ3_HUMAN	CCT5	T-complex protein 1 subunit epsilon	1.0000	5.76	2	2	2
Q$104-4	E9PCA1	E9PCA1_HUMAN	CCT5	T-complex protein 1 subunit epsilon	1.0000	5.38	2	2	2
Q$104-5	P48643	TCPE_HUMAN	CCT5	T-complex protein 1 subunit epsilon	1.0000	5.18	2	2	2
						DVDFELIKVEGK	2	0.9959	1
						LGFAGLVQEISFGTTK	2	0.9955	1
Q$105-1	B7ZC38	B7ZC38_HUMAN	SH3GLB2	Endophilin-B2	0.9978	7.5	2	2	2
Q$105-2	B7ZC39	B7ZC39_HUMAN	SH3GLB2	Endophilin-B2	0.9978	8.02	2	2	2
Q$105-3	F8WFB9	F8WFB9_HUMAN	SH3GLB2	Endophilin-B2	0.9978	23.62	2	2	2
Q$105-4	Q9NR46	SHLB2_HUMAN	SH3GLB2	Endophilin-B2	0.9978	7.59	2	2	2
Q$105-5	Q9NR46-2	SHLB2_HUMAN	SH3GLB2	Isoform 2 of Endophilin-B2	0.9978	7.43	2	2	2
						FGQAEKTELDAHFENLLAR	3	0.806	1
						LASDAGIFFTR	2	0.9884	1
Q$106-1	B7ZKJ8	B7ZKJ8_HUMAN	ITIH4	35 kDa inter-alpha-trypsin inhibitor heavy chain H4	1.0000	19.36	16	17	33
Q$106-2	Q14624	ITIH4_HUMAN	ITIH4	Inter-alpha-trypsin inhibitor heavy chain H4	1.0000	19.46	16	17	33
Q$106-3	Q14624-2	ITIH4_HUMAN	ITIH4	Isoform 2 of Inter-alpha-trypsin inhibitor heavy chain H4	1.0000	19.8	16	17	33
Q$106-4	Q14624-3	ITIH4_HUMAN	ITIH4	Isoform 3 of Inter-alpha-trypsin inhibitor heavy chain H4	1.0000	20.11	16	17	33
						AGFSWIEVTFK	2	0.9994	4
						ANTVQEATFQMELPK	2	0.9996	4
						DQFNLIVFSTEATQWRPSLVPASAENVNK	3	0.9312	1
						EKAEAQAQYSAAVAK	2	0.9909	1
						ETLFSVMPGLK	2	0.8496	1
						GLLFWDGR	2	0.9064	1
						ILDDLSPR	2	0.9877	1
						ITFELVYEELLK	2	0.9995	3
						ITFELVYEELLKR	2	0.8653	1
						LGVYELLLK	2	0.9995	3
						QGPVNLLSDPEQGVEVTGQYER	2	0.9996	1
						QGPVNLLSDPEQGVEVTGQYER	3	0.9402	1
						RLDYQEGPPGVEISCWSVEL	2	0.9995	5
						RLGVYELLLK	3	0.9989	2
						SDPEQGVEVTGQYER	2	0.9868	1
						SPEQQETVLDGNLIIR	2	0.9995	2
						VTIGLLFWDGR	2	0.9977	1
Q$107-1	P40925-2	MDHC_HUMAN	MDH1	Isoform 2 of Malate dehydrogenase, cytoplasmic	1.0000	12.65	3	3	4
Q$107-2	P40925-3	MDHC_HUMAN	MDH1	Isoform 3 of Malate dehydrogenase, cytoplasmic	1.0000	8.81	3	3	4
Q$107-3	B8ZZ51	B8ZZ51_HUMAN	MDH1	Malate dehydrogenase, cytoplasmic	1.0000	18.34	3	3	4
Q$107-4	B9A041	B9A041_HUMAN	MDH1	Malate dehydrogenase, cytoplasmic	1.0000	14.76	3	3	4
Q$107-5	P40925	MDHC_HUMAN	MDH1	Malate dehydrogenase, cytoplasmic	1.0000	9.28	3	3	4
						FVEGLPINDFSR	2	0.874	2
						GEFVTTVQQR	2	0.9973	1
						LGVTANDVK	2	0.965	1
Q$108-1	B9A064	IGLL5_HUMAN	IGLL5	Immunoglobulin lambda-like polypeptide 5	1.0000	23.36	7	8	21
						ATLVCLISDFYPGAVTV	2	0.9435	1

						ATLVCLISDFYPGAVTVAWK	2	0.9997	4
						ATLVCLISDFYPGAVTVAWK	3	0.9997	5
						CLISDFYPGAVTVAWK	2	0.8947	1
						SYSCQVTHEGSTV	2	0.9899	2
						SYSCQVTHEGSTVE	2	0.9852	2
						SYSCQVTHEGSTVEK	2	0.9997	2
						YAASSYLSLTPEQWK	2	0.9997	4
Q$109-1	Q9P1U1	ARP3B_HUMAN	ACTR3B	Actin-related protein 3B	0.9999	2.63	1	1	2
Q$109-2	Q9P1U1-2	ARP3B_HUMAN	ACTR3B	Isoform 2 of Actin-related protein 3B	0.9999	3.32	1	1	2
Q$109-3	Q9P1U1-3	ARP3B_HUMAN	ACTR3B	Isoform 3 of Actin-related protein 3B	0.9999	3.16	1	1	2
Q$109-4	Q9C0K3	ARP3C_HUMAN	ACTR3C	Actin-related protein 3C	0.9999	5.24	1	1	2
Q$109-5	C9IZN3	C9IZN3_HUMAN	ACTR3C	Actin-related protein 3C (Fragment)	0.9999	4.95	1	1	2
Q$109-6	H7C4J1	H7C4J1_HUMAN	ACTR3C	Actin-related protein 3C (Fragment)	0.9999	5.29	1	1	2
						DITYFIQQLLR	2	0.9994	2
Q$110-1	P43307-2	SSRA_HUMAN	SSR1	Isoform 2 of Translocon-associated protein subunit alpha	0.9856	5.79	1	1	2
Q$110-2	C9IZQ1	C9IZQ1_HUMAN	SSR1	Translocon-associated protein subunit alpha	0.9856	5.03	1	1	2
Q$110-3	C9J3L8	C9J3L8_HUMAN	SSR1	Translocon-associated protein subunit alpha	0.9856	5.66	1	1	2
Q$110-4	C9J5W0	C9J5W0_HUMAN	SSR1	Translocon-associated protein subunit alpha	0.9856	5.64	1	1	2
Q$110-5	E9PAL7	E9PAL7_HUMAN	SSR1	Translocon-associated protein subunit alpha	0.9856	5.15	1	1	2
Q$110-6	F5H5Y2	F5H5Y2_HUMAN	SSR1	Translocon-associated protein subunit alpha	0.9856	5.79	1	1	2
Q$110-7	P43307	SSRA_HUMAN	SSR1	Translocon-associated protein subunit alpha	0.9856	5.24	1	1	2
						GTEDFIVESLDASFR	2	0.9856	2
Q$111-1	P35080-2	PROF2_HUMAN	PFN2	Isoform IIb of Profilin-2	1.0000	16.43	2	2	5
Q$111-2	G5E9Q6	G5E9Q6_HUMAN	PFN2	Profilin	1.0000	12.23	2	2	5
Q$111-3	C9J0J7	C9J0J7_HUMAN	PFN2	Profilin-2	1.0000	25.27	2	2	5
						AYELALYLR	2	0.9994	3
						SQGGEPTYNVAVGR	2	0.9996	2
Q$112-1	U3KQA9	U3KQA9_HUMAN		Uncharacterized protein (Fragment)	1.0000	8.47	3	3	6
Q$112-2	U3KQV3	U3KQV3_HUMAN		Uncharacterized protein (Fragment)	1.0000	7.75	3	3	6
Q$112-3	C9JRM1	C9JRM1_HUMAN	RHOA	Transforming protein RhoA	1.0000	23.33	3	3	6
Q$112-4	C9J1T2	C9J1T2_HUMAN	RHOA	Transforming protein RhoA (Fragment)	1.0000	24.42	3	3	6
Q$112-5	E9PLA2	E9PLA2_HUMAN	RHOC	Rho-related GTP-binding protein RhoC	1.0000	31.82	3	3	6
Q$112-6	E9PN11	E9PN11_HUMAN	RHOC	Rho-related GTP-binding protein RhoC (Fragment)	1.0000	16.28	3	3	6
Q$112-7	Q5JR06	Q5JR06_HUMAN	RHOC	Rho-related GTP-binding protein RhoC (Fragment)	1.0000	23.08	3	3	6
						KLVIVGDGACGK	2	0.9964	1
						LVIVGDGACGK	2	0.984	2
						TCLLIVFSK	2	0.9996	3
Q$113-1	D6RBD7	D6RBD7_HUMAN	EEF1E1	Eukaryotic translation elongation factor 1 epsilon-1	0.9407	6.67	1	1	1
Q$113-2	O43324	MCA3_HUMAN	EEF1E1	Eukaryotic translation elongation factor 1 epsilon-1	0.9407	5.75	1	1	1
Q$113-3	D6RCQ0	D6RCQ0_HUMAN	EEF1E1	Eukaryotic translation elongation factor 1 epsilon-1 (Fragmer	0.9407	10.64	1	1	1
Q$113-4	H0YAL7	H0YAL7_HUMAN	EEF1E1	Eukaryotic translation elongation factor 1 epsilon-1 (Fragmer	0.9407	7.35	1	1	1
Q$113-5	O43324-2	MCA3_HUMAN	EEF1E1	Isoform 2 of Eukaryotic translation elongation factor 1 epsilo	0.9407	7.19	1	1	1
Q$113-6	C9J1V9	C9J1V9_HUMAN	EEF1E1-BLOC1S5	HCG2043275	0.9407	6.62	1	1	1
						AIVQQWLEYR	2	0.9407	1
Q$114-1	P84085	ARF5_HUMAN	ARF5	ADP-ribosylation factor 5	1.0000	17.78	2	2	4
Q$114-2	C9J1Z8	C9J1Z8_HUMAN	ARF5	ADP-ribosylation factor 5 (Fragment)	1.0000	21.33	2	2	4
						ILMVGLDAAGK	2	0.9979	2
						LGEIVTTIPTIGFNVETVEYK	2	0.9984	2
Q$115-1	Q02818	NUCB1_HUMAN	NUCB1	Nucleobindin-1	0.9977	5.64	2	2	3
Q$115-2	C9J3C1	C9J3C1_HUMAN	NUCB1	Nucleobindin-1 (Fragment)	0.9977	20.97	2	2	3
Q$115-3	C9JBD3	C9JBD3_HUMAN	NUCB1	Nucleobindin-1 (Fragment)	0.9977	23.64	2	2	3
Q$115-4	C9JKZ2	C9JKZ2_HUMAN	NUCB1	Nucleobindin-1 (Fragment)	0.9977	9.74	2	2	3
Q$115-5	H7BZI1	H7BZI1_HUMAN	NUCB1	Nucleobindin-1 (Fragment)	0.9977	8.81	2	2	3
						LQAANAEDIK	2	0.956	1
						YLQEVIDVLETDGHFR	3	0.9471	2
Q$116-1	C9J3M6	C9J3M6_HUMAN	SLC9C1	Sodium/hydrogen exchanger 10 (Fragment)	0.9458	57.65	1	1	1
						KLFWQILLISIPGFLVNYILVLWHLASVNQLLLKPTQWLLFSAILVSSD	4	0.9458	1
Q$117-1	C9J4S4	C9J4S4_HUMAN	RAB7A	Ras-related protein Rab-7a	1.0000	14.29	1	1	2
Q$117-2	C9J4V0	C9J4V0_HUMAN	RAB7A	Ras-related protein Rab-7a	1.0000	10.45	1	1	2
						EAINVEQAFQTIAR	2	0.9994	2
Q$118-1	I3L504	I3L504_HUMAN	EIF5A	Eukaryotic translation initiation factor 5A-1	1.0000	6.45	1	1	2
Q$118-2	I3L397	I3L397_HUMAN	EIF5A	Eukaryotic translation initiation factor 5A-1 (Fragment)	1.0000	8.22	1	1	2
Q$118-3	C9J7B5	C9J7B5_HUMAN	EIF5A2	Eukaryotic translation initiation factor 5A-2	1.0000	11.01	1	1	2
Q$118-4	F8WCJ1	F8WCJ1_HUMAN	EIF5A2	Eukaryotic translation initiation factor 5A-2	1.0000	11.43	1	1	2
Q$118-5	Q9GZV4	IF5A2_HUMAN	EIF5A2	Eukaryotic translation initiation factor 5A-2	1.0000	7.84	1	1	2
Q$118-6	C9J4W5	C9J4W5_HUMAN	EIF5A2	Eukaryotic translation initiation factor 5A-2 (Fragment)	1.0000	10.43	1	1	2
						VHLVGIDIFTGK	3	0.9992	2
Q$119-1	C9J5S7	C9J5S7_HUMAN	PPIA	Peptidyl-prolyl cis-trans isomerase	1.0000	9.09	1	1	2
Q$119-2	F8WE65	F8WE65_HUMAN	PPIA	Peptidyl-prolyl cis-trans isomerase	1.0000	9.17	1	1	2
						DIAVDGEPLGR	2	0.9791	2
Q$120-1	C9J6D1	C9J6D1_HUMAN	NAP1L4	Nucleosome assembly protein 1-like 4 (Fragment)	1.0000	11.8	2	2	3
						FYEEVHDLER	2	0.9994	2
						GIPEFWFTIFR	2	0.9989	1
Q$121-1	C9J6K0	C9J6K0_HUMAN	SPP2	Secreted phosphoprotein 24 (Fragment)	1.0000	7.63	1	1	1
						DYYVSTAVCR	2	0.9991	1
Q$122-1	C9JQ45	C9JQ45_HUMAN	PFN2	Profilin	1.0000	12.73	1	1	2
Q$122-2	C9J712	C9J712_HUMAN	PFN2	Profilin-2	1.0000	15.38	1	1	2
Q$122-3	P35080	PROF2_HUMAN	PFN2	Profilin-2	1.0000	10	1	1	2
						SQGGEPTYNVAVGR	2	0.9996	2
Q$123-1	C9J7H8	C9J7H8_HUMAN	LDHB	L-lactate dehydrogenase B chain (Fragment)	1.0000	22.79	2	2	3
Q$123-2	F5H793	F5H793_HUMAN	LDHB	L-lactate dehydrogenase B chain (Fragment)	1.0000	30.39	2	2	3
						LIAPVAEEEATVPNNK	2	0.9996	2
						SLADELALVDVLEDK	2	0.9993	1
Q$124-1	C9J7S3	C9J7S3_HUMAN	DARS	Aspartate--tRNA ligase, cytoplasmic (Fragment)	1.0000	11.43	2	2	3
Q$124-2	C9JLC1	C9JLC1_HUMAN	DARS	Aspartate--tRNA ligase, cytoplasmic (Fragment)	1.0000	10.99	2	2	3
						IYVISLAEPR	2	0.9987	2
						LQSGICHLFR	2	0.964	1
Q$125-1	C9J8S3	C9J8S3_HUMAN	RAB7A	Ras-related protein Rab-7a	1.0000	17.5	2	2	4
Q$125-2	P51149	RAB7A_HUMAN	RAB7A	Ras-related protein Rab-7a	1.0000	13.53	2	2	4
						EAINVEQAFQTIAR	2	0.9994	2
						LVTMQIWDTAGQER	2	0.9989	2
Q$126-1	Q969P0	IGSF8_HUMAN	IGSF8	Immunoglobulin superfamily member 8	1.0000	6.36	3	3	5
Q$126-2	C9J8Z4	C9J8Z4_HUMAN	IGSF8	Immunoglobulin superfamily member 8 (Fragment)	1.0000	12.96	3	3	5
Q$126-3	Q969P0-3	IGSF8_HUMAN	IGSF8	Isoform 3 of Immunoglobulin superfamily member 8	1.0000	7.41	3	3	5
						DTQFSYAVFK	2	0.9993	2
						LQAQDAGIYECHTPSTDTR	3	0.9945	2
						VVAGEVQVQR	2	0.9997	1
Q$127-1	F5H3X9	F5H3X9_HUMAN	PPP2R1A	Serine/threonine-protein phosphatase 2A 65 kDa regulatory	1.0000	6.74	4	4	8
Q$127-2	C9J9C1	C9J9C1_HUMAN	PPP2R1A	Serine/threonine-protein phosphatase 2A 65 kDa regulatory	1.0000	9.11	4	4	8
						FSVCYPR	2	0.8942	1
						LAGGDWFTSR	2	0.993	4
						TSACGLFSVCYPR	2	0.9992	2
						VKEFCENLSADCR	2	0.9033	1
Q$128-1	C9J9S3	C9J9S3_HUMAN	PPP1CB	Serine/threonine-protein phosphatase (Fragment)	1.0000	21.6	2	2	4
Q$128-2	C9JP48	C9JP48_HUMAN	PPP1CB	Serine/threonine-protein phosphatase (Fragment)	1.0000	19.57	2	2	4
						EIFLSQPILLELEAPLK	2	0.9997	3
						IVQMTEAEVR	2	0.9977	1
Q$129-1	Q16629-2	SRSF7_HUMAN	SRSF7	Isoform 2 of Serine/arginine-rich splicing factor 7	1.0000	34.81	4	5	8
Q$129-2	Q16629-3	SRSF7_HUMAN	SRSF7	Isoform 3 of Serine/arginine-rich splicing factor 7	1.0000	35.61	4	5	8
Q$129-3	Q16629-4	SRSF7_HUMAN	SRSF7	Isoform 4 of Serine/arginine-rich splicing factor 7	1.0000	20.8	4	5	8
Q$129-4	Q16629	SRSF7_HUMAN	SRSF7	Serine/arginine-rich splicing factor 7	1.0000	19.75	4	5	8
Q$129-5	C9JAB2	C9JAB2_HUMAN	SRSF7	Serine/arginine-rich-splicing factor 7	1.0000	20	4	5	8
						AFSYYGPLR	2	0.9864	1
						NPPGFAFVEFEDPR	2	0.9997	3
						NPPGFAFVEFEDPR	3	0.9993	2
						NPPGFAFVEFEDPRDAEDAVR	3	0.8895	1
						VYVGNLGTGAGKGELER	2	0.9991	1
Q$130-1	C9JAK5	C9JAK5_HUMAN	ARF4	ADP-ribosylation factor 4	1.0000	46.41	5	5	7
						DAVLLLFANK	2	0.9979	1
						IQEVADELQK	2	0.9944	1
						MLLVDELR	2	0.9955	1
						NICFTVWDVGGQDR	2	0.9784	2
						TWYVQATCATQGTGLYEGLDWLSNELSKR	3	0.999	2

Q$131-1	C9JC84	C9JC84_HUMAN	FGG	Fibrinogen gamma chain	1.0000	51.41	46	51	118
Q$131-2	C9JEU5	C9JEU5_HUMAN	FGG	Fibrinogen gamma chain	1.0000	53.26	46	51	118
						AIQLTYNPDESSKP	2	0.936	1
						AIQLTYNPDESSKPNMIDAATLK	3	0.9968	2
						ALRVELEDWNGR	3	0.9007	1
						ASILTHDSSIR	2	0.9082	1
						ASTPNGYDNGIIWATWK	2	0.9971	4
						CHAGHLNGVYYQGGTYSK	3	0.9979	3
						DNCCILDER	2	0.9997	5
						DTVQIHDITGK	2	0.999	1
						EASILTHDSSIR	2	0.843	1
						EGFGHLSPTGTTEFWLGNEK	2	0.9997	4
						EGFGHLSPTGTTEFWLGNEK	3	0.9996	4
						FGSYCPTTCGIADFL	2	0.9461	2
						FGSYCPTTCGIADFLSTY	2	0.9962	2
						FGSYCPTTCGIADFLSTYQ	2	0.9834	2
						FGSYCPTTCGIADFLSTYQT	2	0.9978	2
						FGSYCPTTCGIADFLSTYQTK	2	0.9998	7
						FGSYCPTTCGIADFLSTYQTK	3	0.9996	4
						FGSYCPTTCGIADFLSTYQTKV	2	0.9937	2
						GIADFLSTYQTK	2	0.9958	2
						IHLISTQ	2	0.976	1
						IHLISTQSAIPYALR	2	0.9997	4
						IHLISTQSAIPYALR	3	0.9995	2
						IHLISTQSAIPYALRV	2	0.9677	1
						IHLISTQSAIPYALRVELEDWNGR	3	0.997	5
						KEGFGHLSPTGTTEFWLGNEK	3	0.9592	2
						KMLEEIMKYEASILTHDSSIR	3	0.9918	2
						LDGSVDFK	2	0.9988	4
						LDGSVDFKK	2	0.9991	2
						LQEIYNSNNQK	2	0.9914	1
						LSPTGTTEFWLGNEK	2	0.9199	1
						LTIGEGQQHHLGGAK	2	0.9994	2
						MLEEIMKYEASILTHDSSIR	3	0.9988	3
						QSGLYFIKPLK	2	0.9997	2
						QSGLYFIKPLK	3	0.9664	1
						RLDGSVDFK	2	0.9992	2
						RVELEDWNGR	2	0.9422	1
						RYLQEIYNSNNQK	2	0.8573	1
						SILTHDSSIR	2	0.9949	4
						SPTGTTEFWLGNEK	2	0.9861	3
						STQSAIPYALR	2	0.9526	1
						TGTTEFWLGNEK	2	0.9087	1
						TSTADYAMFK	2	0.9992	1
						VAQLEAQCQEPCK	2	0.9997	2
						VAQLEAQCQEPCKD	2	0.9969	2
						VAQLEAQCQEPCKDT	2	0.9829	2
						VAQLEAQCQEPCKDTV	2	0.9777	2
						VAQLEAQCQEPCKDTVQIHDITGK	3	0.9987	3
						VELEDWNGR	2	0.9996	2
						YEASILTHDSSIR	2	0.9997	4
						YEASILTHDSSIR	3	0.9956	1
						YLQEIYNSNNQK	2	0.9997	3
Q$132-1	C9JCK5	C9JCK5_HUMAN	PSMA2	Proteasome subunit alpha type-2	0.9729	11.59	1	1	3
Q$132-2	P25787	PSA2_HUMAN	PSMA2	Proteasome subunit alpha type-2	0.9729	8.12	1	1	3
						YNEDLELEDAIHTAILTLK	3	0.9729	3
Q$133-1	P50053-2	KHK_HUMAN	KHK	Isoform C of Ketohexokinase	0.9715	4.03	1	1	1
Q$133-2	C9JDL1	C9JDL1_HUMAN	KHK	Ketohexokinase (Fragment)	0.9715	4.3	1	1	1
						GVDVSQVAWQSK	2	0.9715	1
Q$134-1	C9JEV6	C9JEV6_HUMAN	NAGK	N-acetyl-D-glucosamine kinase	1.0000	9.22	2	2	3
						EGFLLALTQGR	2	0.8813	1
						SLGLSLSGGDQEDAGR	2	0.9996	2
Q$135-1	C9JF17	C9JF17_HUMAN	APOD	Apolipoprotein D (Fragment)	1.0000	36.28	14	19	54
						CPNPPVQENFDVNK	2	0.9997	5
						CPNPPVQENFDVNK	3	0.9993	2
						CPNPPVQENFDVNKYLGR	2	0.9995	5
						CPNPPVQENFDVNKYLGR	3	0.9994	3
						DSLKNILTSNNIDVK	2	0.9402	1
						IKVLNQELR	2	0.9804	1
						IKVLNQELR	3	0.8852	1
						KMTVTDQVNCPK	2	0.9997	2
						KMTVTDQVNCPK	3	0.9994	1
						MTVTDQVNCPK	2	0.9996	2
						NILTSNNIDVK	2	0.9997	4
						NILTSNNIDVKK	2	0.9997	1
						NPNLPPETVDSLK	2	0.9997	11
						NPNLPPETVDSLKNILTSNNIDVK	3	0.986	2
						NPPVQENFDVNK	2	0.9905	2
						VLNQELR	2	0.9994	2
						WYEIEKIPTTFE	2	0.963	2
						WYEIEKIPTTFENGR	2	0.9992	6
						WYEIEKIPTTFENGR	3	0.9986	1
Q$136-1	C9JFE4	C9JFE4_HUMAN	GPS1	COP9 signalosome complex subunit 1	0.9994	4.25	2	2	2
Q$136-2	Q13098	CSN1_HUMAN	GPS1	COP9 signalosome complex subunit 1	0.9994	4.07	2	2	2
Q$136-3	J3KRE8	J3KRE8_HUMAN	GPS1	COP9 signalosome complex subunit 1 (Fragment)	0.9994	10.75	2	2	2
Q$136-4	J3KRJ4	J3KRJ4_HUMAN	GPS1	COP9 signalosome complex subunit 1 (Fragment)	0.9994	7.91	2	2	2
Q$136-5	J3KSA5	J3KSA5_HUMAN	GPS1	COP9 signalosome complex subunit 1 (Fragment)	0.9994	9.52	2	2	2
Q$136-6	J3KTB0	J3KTB0_HUMAN	GPS1	COP9 signalosome complex subunit 1 (Fragment)	0.9994	14.39	2	2	2
Q$136-7	J3QL53	J3QL53_HUMAN	GPS1	COP9 signalosome complex subunit 1 (Fragment)	0.9994	11.9	2	2	2
Q$136-8	J3QLE8	J3QLE8_HUMAN	GPS1	COP9 signalosome complex subunit 1 (Fragment)	0.9994	12.05	2	2	2
Q$136-9	J3QLT0	J3QLT0_HUMAN	GPS1	COP9 signalosome complex subunit 1 (Fragment)	0.9994	11.9	2	2	2
Q$136-10	J3QS84	J3QS84_HUMAN	GPS1	COP9 signalosome complex subunit 1 (Fragment)	0.9994	19.8	2	2	2
Q$136-11	J3QS88	J3QS88_HUMAN	GPS1	COP9 signalosome complex subunit 1 (Fragment)	0.9994	14.93	2	2	2
Q$136-12	Q13098-7	CSN1_HUMAN	GPS1	Isoform 2 of COP9 signalosome complex subunit 1	0.9994	3.8	2	2	2
Q$136-13	Q13098-5	CSN1_HUMAN	GPS1	Isoform 4 of COP9 signalosome complex subunit 1	0.9994	4.11	2	2	2
						LQFIADHCPTLR	3	0.9428	1
						MALSFVQR	2	0.9893	1
Q$137-1	C9JIS1	C9JIS1_HUMAN	GNB2	Guanine nucleotide-binding protein G(I)/G(S)/G(T) subunit be	1.0000	5.17	1	1	1
Q$137-2	C9JXA5	C9JXA5_HUMAN	GNB2	Guanine nucleotide-binding protein G(I)/G(S)/G(T) subunit be	1.0000	4.78	1	1	1
						TFVSGACDASIK	2	0.9995	1
Q$138-1	C9JLK0	C9JLK0_HUMAN	ATIC	Phosphoribosylaminoimidazolecarboxamide formyltransferas	1.0000	10.59	2	2	5
						LDFNLIR	2	0.9996	2
						VVACNLYPFVK	2	0.9995	3
Q$139-1	Q92841-1	DDX17_HUMAN	DDX17	Isoform 2 of Probable ATP-dependent RNA helicase DDX17	0.9998	1.38	1	1	2
Q$139-2	Q92841-2	DDX17_HUMAN	DDX17	Isoform 3 of Probable ATP-dependent RNA helicase DDX17	0.9998	1.38	1	1	2
Q$139-3	Q92841-3	DDX17_HUMAN	DDX17	Isoform 4 of Probable ATP-dependent RNA helicase DDX17	0.9998	1.38	1	1	2
Q$139-4	C9JMU5	C9JMU5_HUMAN	DDX17	Probable ATP-dependent RNA helicase DDX17	0.9998	1.38	1	1	2
Q$139-5	H3BLZ8	H3BLZ8_HUMAN	DDX17	Probable ATP-dependent RNA helicase DDX17	0.9998	1.23	1	1	2
Q$139-6	Q92841	DDX17_HUMAN	DDX17	Probable ATP-dependent RNA helicase DDX17	0.9998	1.23	1	1	2
						LIDFLESGK	2	0.9406	2
Q$140-1	C9JX21	C9JX21_HUMAN	RHOA	Transforming protein RhoA	1.0000	19.25	4	4	8
Q$140-2	C9JNR4	C9JNR4_HUMAN	RHOA	Transforming protein RhoA (Fragment)	1.0000	27.91	4	4	8
Q$140-3	Q5JR05	Q5JR05_HUMAN	RHOC	Rho-related GTP-binding protein RhoC	1.0000	18.09	4	4	8
Q$140-4	Q5JR07	Q5JR07_HUMAN	RHOC	Rho-related GTP-binding protein RhoC (Fragment)	1.0000	28.35	4	4	8
						HFCPNVPIILVGNKK	3	0.9995	2
						KLVIVGDGACGK	2	0.9964	1
						LVIVGDGACGK	2	0.984	2
						TCLLIVFSK	2	0.9996	3
Q$141-1	C9JPM4	C9JPM4_HUMAN	ARF4	ADP-ribosylation factor 4 (Fragment)	1.0000	33.07	3	3	5
						ILMVGLDAAGK	2	0.9979	2
						IQEVADELQK	2	0.9944	1
						LGEIVTTIPTIGFNVETVEYK	2	0.9984	2
Q$142-1	C9JQM9	C9JQM9_HUMAN	DARS	Aspartate--tRNA ligase, cytoplasmic (Fragment)	1.0000	7.41	1	1	2

						IYVISLAEPR	2	0.9987	2
Q$143-1	Q9NTK5-3	OLA1_HUMAN	OLA1	Isoform 3 of Obg-like ATPase 1	1.0000	5.4	1	2	4
Q$143-2	C9JTK6	C9JTK6_HUMAN	OLA1	Obg-like ATPase 1 (Fragment)	1.0000	12.93	1	2	4
						IPAFLNVVDIAGLVK	2	0.9997	3
						IPAFLNVVDIAGLVK	3	0.9488	1
Q$144-1	C9JVN1	C9JVN1_HUMAN	MFI2	Melanotransferrin (Fragment)	1.0000	8.84	1	1	3
						TVGWNVPVGYLVESGR	2	0.9994	3
Q$145-1	Q15233-2	NONO_HUMAN	NONO	Isoform 2 of Non-POU domain-containing octamer-binding pı	0.9511	3.66	1	1	2
Q$145-2	Q15233	NONO_HUMAN	NONO	Non-POU domain-containing octamer-binding protein	0.9511	2.97	1	1	2
Q$145-3	C9JYS8	C9JYS8_HUMAN	NONO	Non-POU domain-containing octamer-binding protein (Fragm	0.9511	5.67	1	1	2
Q$145-4	H7C367	H7C367_HUMAN	NONO	Non-POU domain-containing octamer-binding protein (Fragm	0.9511	5.98	1	1	2
						FAQPGSFEYEYAMR	2	0.9511	2
Q$146-1	P13010	XRCC5_HUMAN	XRCC5	X-ray repair cross-complementing protein 5	0.9472	1.5	1	1	1
Q$146-2	C9JZ81	C9JZ81_HUMAN	XRCC5	X-ray repair cross-complementing protein 5 (Fragment)	0.9472	8.4	1	1	1
						HIEIFTDLSSR	2	0.9472	1
Q$147-1	P62256-2	UBE2H_HUMAN	UBE2H	Isoform 2 of Ubiquitin-conjugating enzyme E2 H	1.0000	19.74	2	3	4
Q$147-2	P62256	UBE2H_HUMAN	UBE2H	Ubiquitin-conjugating enzyme E2 H	1.0000	16.39	2	3	4
Q$147-3	C9JZG9	C9JZG9_HUMAN	UBE2H	Ubiquitin-conjugating enzyme E2 H (Fragment)	1.0000	29.13	2	3	4
Q$147-4	C9JZY6	C9JZY6_HUMAN	UBE2H	Ubiquitin-conjugating enzyme E2 H (Fragment)	1.0000	24.39	2	3	4
Q$147-5	H7C4M9	H7C4M9_HUMAN	UBE2H	Ubiquitin-conjugating enzyme E2 H (Fragment)	1.0000	48.39	2	3	4
						FYGPQGTPYEGGVWK	2	0.9718	1
						HEVTILGGLNEFVVK	2	0.9915	1
						HEVTILGGLNEFVVK	3	0.9995	2
Q$148-1	C9JZP6	C9JZP6_HUMAN	DHRS2	Dehydrogenase/reductase SDR family member 2, mitochond	1.0000	40.44	8	8	11
Q$148-2	Q13268-2	DHRS2_HUMAN	DHRS2	Isoform 2 of Dehydrogenase/reductase SDR family member 2	1.0000	30.33	8	8	11
						AGIVCHVGK	2	0.886	1
						LQGEGLSVAGIVCHVGK	3	0.9921	1
						LSVAGIVCHVGK	2	0.9442	1
						SAGVNPLVGSTLGTSEQIWDK	2	0.9922	1
						SPALLLSQLLPYMENRR	3	0.9954	2
						TLALELAPK	2	0.9656	1
						VAVVTGSTSGIGFAIAR	2	0.9996	3
						VNCVVPGIIK	2	0.9975	1
Q$149-1	C9JZR2	C9JZR2_HUMAN	CTNND1	Catenin delta-1	0.9943	1.92	2	2	2
Q$149-2	O60716	CTND1_HUMAN	CTNND1	Catenin delta-1	0.9943	1.86	2	2	2
Q$149-3	O60716-8	CTND1_HUMAN	CTNND1	Isoform 1 of Catenin delta-1	0.9943	1.97	2	2	2
Q$149-4	O60716-5	CTND1_HUMAN	CTNND1	Isoform 1A of Catenin delta-1	0.9943	1.93	2	2	2
Q$149-5	O60716-2	CTND1_HUMAN	CTNND1	Isoform 1AB of Catenin delta-1	0.9943	1.87	2	2	2
Q$149-6	O60716-3	CTND1_HUMAN	CTNND1	Isoform 1AC of Catenin delta-1	0.9943	1.92	2	2	2
Q$149-7	O60716-6	CTND1_HUMAN	CTNND1	Isoform 1B of Catenin delta-1	0.9943	1.91	2	2	2
Q$149-8	O60716-4	CTND1_HUMAN	CTNND1	Isoform 1BC of Catenin delta-1	0.9943	1.9	2	2	2
Q$149-9	O60716-7	CTND1_HUMAN	CTNND1	Isoform 1C of Catenin delta-1	0.9943	1.96	2	2	2
Q$149-10	O60716-16	CTND1_HUMAN	CTNND1	Isoform 2 of Catenin delta-1	0.9943	2.1	2	2	2
Q$149-11	O60716-13	CTND1_HUMAN	CTNND1	Isoform 2A of Catenin delta-1	0.9943	2.05	2	2	2
Q$149-12	O60716-10	CTND1_HUMAN	CTNND1	Isoform 2AB of Catenin delta-1	0.9943	1.98	2	2	2
Q$149-13	O60716-9	CTND1_HUMAN	CTNND1	Isoform 2ABC of Catenin delta-1	0.9943	1.97	2	2	2
Q$149-14	O60716-11	CTND1_HUMAN	CTNND1	Isoform 2AC of Catenin delta-1	0.9943	2.03	2	2	2
Q$149-15	O60716-14	CTND1_HUMAN	CTNND1	Isoform 2B of Catenin delta-1	0.9943	2.03	2	2	2
Q$149-16	O60716-12	CTND1_HUMAN	CTNND1	Isoform 2BC of Catenin delta-1	0.9943	2.02	2	2	2
Q$149-17	O60716-15	CTND1_HUMAN	CTNND1	Isoform 2C of Catenin delta-1	0.9943	2.08	2	2	2
Q$149-18	O60716-24	CTND1_HUMAN	CTNND1	Isoform 3 of Catenin delta-1	0.9943	2.22	2	2	2
Q$149-19	O60716-21	CTND1_HUMAN	CTNND1	Isoform 3A of Catenin delta-1	0.9943	2.16	2	2	2
Q$149-20	O60716-18	CTND1_HUMAN	CTNND1	Isoform 3AB of Catenin delta-1	0.9943	2.09	2	2	2
Q$149-21	O60716-17	CTND1_HUMAN	CTNND1	Isoform 3ABC of Catenin delta-1	0.9943	2.08	2	2	2
Q$149-22	O60716-19	CTND1_HUMAN	CTNND1	Isoform 3AC of Catenin delta-1	0.9943	2.15	2	2	2
Q$149-23	O60716-22	CTND1_HUMAN	CTNND1	Isoform 3B of Catenin delta-1	0.9943	2.14	2	2	2
Q$149-24	O60716-20	CTND1_HUMAN	CTNND1	Isoform 3BC of Catenin delta-1	0.9943	2.13	2	2	2
Q$149-25	O60716-23	CTND1_HUMAN	CTNND1	Isoform 3C of Catenin delta-1	0.9943	2.2	2	2	2
Q$149-26	O60716-32	CTND1_HUMAN	CTNND1	Isoform 4 of Catenin delta-1	0.9943	3.06	2	2	2
Q$149-27	O60716-29	CTND1_HUMAN	CTNND1	Isoform 4A of Catenin delta-1	0.9943	2.95	2	2	2
Q$149-28	O60716-26	CTND1_HUMAN	CTNND1	Isoform 4AB of Catenin delta-1	0.9943	2.82	2	2	2
Q$149-29	O60716-25	CTND1_HUMAN	CTNND1	Isoform 4ABC of Catenin delta-1	0.9943	2.79	2	2	2
Q$149-30	O60716-27	CTND1_HUMAN	CTNND1	Isoform 4AC of Catenin delta-1	0.9943	2.92	2	2	2
Q$149-31	O60716-30	CTND1_HUMAN	CTNND1	Isoform 4B of Catenin delta-1	0.9943	2.91	2	2	2
Q$149-32	O60716-28	CTND1_HUMAN	CTNND1	Isoform 4BC of Catenin delta-1	0.9943	2.88	2	2	2
Q$149-33	O60716-31	CTND1_HUMAN	CTNND1	Isoform 4C of Catenin delta-1	0.9943	3.03	2	2	2
						LVENCVCLLR	2	0.8892	1
						NLSYQVHR	2	0.9485	1
Q$150-1	F8W727	F8W727_HUMAN	RPL32	60S ribosomal protein L32	0.9753	8.5	1	1	2
Q$150-2	P62910	RL32_HUMAN	RPL32	60S ribosomal protein L32	0.9753	9.63	1	1	2
Q$150-3	D3YTB1	D3YTB1_HUMAN	RPL32	60S ribosomal protein L32 (Fragment)	0.9753	9.77	1	1	2
						SYCAEIAHNVSSK	2	0.9753	2
Q$151-1	D6R934	D6R934_HUMAN	C1QB	Complement C1q subcomponent subunit B	1.0000	25.9	6	7	8
Q$151-2	P02746	C1QB_HUMAN	C1QB	Complement C1q subcomponent subunit B	1.0000	25.69	6	7	8
						DYAYNTFQVTTGGMVLK	2	0.9529	1
						IAFSATR	2	0.9987	1
						LEQGENVFLQATDK	2	0.9997	1
						TINVPLRR	2	0.9825	1
						VPGLYYFTYHASSR	2	0.9964	1
						VVTFCDYAYNTFQVTTGGMVLK	2	0.9847	2
						VVTFCDYAYNTFQVTTGGMVLK	3	0.898	1
Q$152-1	D6R938	D6R938_HUMAN	CAMK2D	Calcium/calmodulin-dependent protein kinase (CaM kinase)	1.0000	7.63	2	2	2
Q$152-2	E9PBG7	E9PBG7_HUMAN	CAMK2D	Calcium/calmodulin-dependent protein kinase type II subunit	1.0000	7.42	2	2	2
Q$152-3	E9PF82	E9PF82_HUMAN	CAMK2D	Calcium/calmodulin-dependent protein kinase type II subunit	1.0000	7.13	2	2	2
Q$152-4	Q13557	KCC2D_HUMAN	CAMK2D	Calcium/calmodulin-dependent protein kinase type II subunit	1.0000	7.62	2	2	2
Q$152-5	Q13557-10	KCC2D_HUMAN	CAMK2D	Isoform Delta 10 of Calcium/calmodulin-dependent protein k	1.0000	7.72	2	2	2
Q$152-6	Q13557-11	KCC2D_HUMAN	CAMK2D	Isoform Delta 11 of Calcium/calmodulin-dependent protein k	1.0000	7.25	2	2	2
Q$152-7	Q13557-12	KCC2D_HUMAN	CAMK2D	Isoform Delta 12 of Calcium/calmodulin-dependent protein k	1.0000	7.95	2	2	2
Q$152-8	Q13557-3	KCC2D_HUMAN	CAMK2D	Isoform Delta 3 of Calcium/calmodulin-dependent protein kiı	1.0000	7.45	2	2	2
Q$152-9	Q13557-4	KCC2D_HUMAN	CAMK2D	Isoform Delta 4 of Calcium/calmodulin-dependent protein kiı	1.0000	7.32	2	2	2
Q$152-10	Q13557-8	KCC2D_HUMAN	CAMK2D	Isoform Delta 6 of Calcium/calmodulin-dependent protein kiı	1.0000	7.95	2	2	2
Q$152-11	Q13557-9	KCC2D_HUMAN	CAMK2D	Isoform Delta 7 of Calcium/calmodulin-dependent protein kiı	1.0000	7.77	2	2	2
Q$152-12	Q13557-5	KCC2D_HUMAN	CAMK2D	Isoform Delta 8 of Calcium/calmodulin-dependent protein kiı	1.0000	7.63	2	2	2
Q$152-13	Q13557-6	KCC2D_HUMAN	CAMK2D	Isoform Delta 9 of Calcium/calmodulin-dependent protein kiı	1.0000	7.41	2	2	2
						FTDEYQLFEELGK	2	0.9997	1
						ICDPGLTAFEPEALGNLVEGMDFHR	3	0.9941	1
Q$153-1	P26583	HMGB2_HUMAN	HMGB2	High mobility group protein B2	0.9738	6.22	1	1	1
Q$153-2	D6R9A6	D6R9A6_HUMAN	HMGB2	High mobility group protein B2 (Fragment)	0.9738	9.7	1	1	1
						SEHPGLSIGDTAK	2	0.9529	1
Q$154-1	P10451-3	OSTP_HUMAN	SPP1	Isoform C of Osteopontin	1.0000	9.06	3	3	28
Q$154-2	D6R9C5	D6R9C5_HUMAN	SPP1	Osteopontin (Fragment)	1.0000	10.79	3	3	28
						AIPVAQDLNAPSDWDSR	2	0.9997	22
						GDSVVYGLR	2	0.9997	3
						LNAPSDWDSR	2	0.9849	3
Q$155-1	D6RBF6	D6RBF6_HUMAN	BDH2	3-hydroxybutyrate dehydrogenase type 2	1.0000	32.81	1	1	3
Q$155-2	D6RFG2	D6RFG2_HUMAN	BDH2	3-hydroxybutyrate dehydrogenase type 2	1.0000	23.33	1	1	3
Q$155-3	D6R9U8	D6R9U8_HUMAN	BDH2	3-hydroxybutyrate dehydrogenase type 2 (Fragment)	1.0000	18.1	1	1	3
Q$155-4	D6RIR6	D6RIR6_HUMAN	BDH2	3-hydroxybutyrate dehydrogenase type 2 (Fragment)	1.0000	16.15	1	1	3
Q$155-5	Q9BUT1-2	BDH2_HUMAN	BDH2	Isoform 2 of 3-hydroxybutyrate dehydrogenase type 2	1.0000	12.14	1	1	3
						VIILTAAAQGIGQAAALAFAR	3	0.9996	3
Q$156-1	D6RA82	D6RA82_HUMAN	ANXA3	Annexin	0.9999	4.58	1	1	1
Q$156-2	P12429	ANXA3_HUMAN	ANXA3	Annexin A3	0.9999	4.02	1	1	1
						SDTSGDYEITLLK	2	0.9876	1
Q$157-1	D6RAC2	D6RAC2_HUMAN	GNB2L1	Guanine nucleotide-binding protein subunit beta-2-like 1	1.0000	7.81	2	2	3
Q$157-2	D6RF23	D6RF23_HUMAN	GNB2L1	Guanine nucleotide-binding protein subunit beta-2-like 1	1.0000	20.79	2	2	3
Q$157-3	H0Y8R5	H0Y8R5_HUMAN	GNB2L1	Guanine nucleotide-binding protein subunit beta-2-like 1 (Fra	1.0000	15.22	2	2	3
						VWQVTIGTR	2	0.9992	2
						YWLCAATGPSIK	2	0.9997	1
Q$158-1	D6RAT0	D6RAT0_HUMAN	RPS3A	40S ribosomal protein S3a	0.9467	7.49	1	1	1
Q$158-2	D6RI02	D6RI02_HUMAN	RPS3A	40S ribosomal protein S3a	0.9467	17.89	1	1	1
Q$158-3	E9PFI5	E9PFI5_HUMAN	RPS3A	40S ribosomal protein S3a	0.9467	8.29	1	1	1
Q$158-4	F5H4F9	F5H4F9_HUMAN	RPS3A	40S ribosomal protein S3a	0.9467	9.71	1	1	1
Q$158-5	P61247	RS3A_HUMAN	RPS3A	40S ribosomal protein S3a	0.9467	6.44	1	1	1

Q$158-6	D6RAS7	D6RAS7_HUMAN	RPS3A	40S ribosomal protein S3a (Fragment)	0.9467	13.6	1	1	1
Q$158-7	D6RB09	D6RB09_HUMAN	RPS3A	40S ribosomal protein S3a (Fragment)	0.9467	8.81	1	1	1
Q$158-8	D6RED7	D6RED7_HUMAN	RPS3A	40S ribosomal protein S3a (Fragment)	0.9467	16.83	1	1	1
Q$158-9	D6RG13	D6RG13_HUMAN	RPS3A	40S ribosomal protein S3a (Fragment)	0.9467	7.62	1	1	1
Q$158-10	D6RGE0	D6RGE0_HUMAN	RPS3A	40S ribosomal protein S3a (Fragment)	0.9467	26.15	1	1	1
Q$158-11	H0Y8L7	H0Y8L7_HUMAN	RPS3A	40S ribosomal protein S3a (Fragment)	0.9467	8.95	1	1	1
						VFEVSLADLQNDEVAFR	2	0.9467	1
Q$159-1	D6RAX7	D6RAX7_HUMAN	COPS4	COP9 signalosome complex subunit 4	1.0000	10	3	4	6
Q$159-2	D6RFN0	D6RFN0_HUMAN	COPS4	COP9 signalosome complex subunit 4	1.0000	9.59	3	4	6
Q$159-3	Q9BT78	CSN4_HUMAN	COPS4	COP9 signalosome complex subunit 4	1.0000	10.34	3	4	6
						CQQLAAYGILEK	2	0.857	1
						IASQMITEGR	2	0.9894	2
						LYNNITFEELGALLEIPAAK	2	0.9994	1
						LYNNITFEELGALLEIPAAK	3	0.999	2
Q$160-1	D6RBV2	D6RBV2_HUMAN	LMAN2	Vesicular integral-membrane protein VIP36	1.0000	48	21	26	75
Q$160-2	Q12907	LMAN2_HUMAN	LMAN2	Vesicular integral-membrane protein VIP36	1.0000	43.82	21	26	75
						DGIALWYTR	2	0.8412	2
						DHDTFLAVR	2	0.9995	2
						DITDGNSEHLKR	2	0.9681	1
						DNFHGLAIFLDTYPNDETTER	2	0.9997	2
						DRLVPGPVFGSK	2	0.999	2
						DRLVPGPVFGSK	3	0.9986	2
						DRLVPGPVFGSKDNFHGLAIFLDTYPNDETTER	4	0.9637	1
						DTYPNDETTER	2	0.9856	4
						LAGCTADFR	2	0.8968	1
						LDTYPNDETTER	2	0.967	2
						LFQLMVEHTPDEESIDWTK	2	0.9997	1
						LFQLMVEHTPDEESIDWTK	3	0.9992	3
						LPTGYYFGASAGTGDLSDNHDIISMK	3	0.9997	12
						LTVMTDLEDKNEWK	2	0.9995	4
						LTVMTDLEDKNEWK	3	0.921	1
						LVPGPVFGSK	2	0.997	1
						LVPGPVFGSKDNFHGLAIFLDTYPNDETTER	3	0.9969	4
						NCIDITGVR	2	0.9993	2
						NCIDITGVRLPTG	2	0.9302	1
						NCIDITGVRLPTGYY	2	0.8746	1
						NLHGDGIALWYTR	2	0.9996	4
						NLHGDGIALWYTR	3	0.9994	5
						NRDHDTFLAVR	2	0.9989	3
						NRDHDTFLAVR	3	0.9994	4
						VFPYISV	2	0.8189	1
						WTELAGCTADFR	2	0.9998	9
Q$161-1	D6RD47	D6RD47_HUMAN	RPS23	40S ribosomal protein S23	0.9993	8.96	2	2	2
Q$161-2	P62266	RS23_HUMAN	RPS23	40S ribosomal protein S23	0.9993	8.39	2	2	2
						GHAVGDIPGVR	2	0.9328	1
						KGHAVGDIPGVR	2	0.9894	1
Q$162-1	D6RD63	D6RD63_HUMAN	COPS4	COP9 signalosome complex subunit 4 (Fragment)	1.0000	15.27	3	3	4
						CQQLAAYGILEK	2	0.857	1
						IASQMITEGR	2	0.9894	2
						LYNNITFEELGALLEIPAAK	2	0.9994	1
Q$163-1	D6RD66	D6RD66_HUMAN	WDR1	WD repeat-containing protein 1 (Fragment)	1.0000	19.59	4	4	8
						DIAWTEDSKR	2	0.9981	2
						DIYTEHAHQVVVAK	2	0.9728	1
						IAVVGEGR	2	0.9996	2
						YAPSGFYIASGDVSGK	2	0.9997	3
Q$164-1	P20290-2	BTF3_HUMAN	BTF3	Isoform 2 of Transcription factor BTF3	0.9867	11.73	1	1	1
Q$164-2	P20290	BTF3_HUMAN	BTF3	Transcription factor BTF3	0.9867	9.22	1	1	1
Q$164-3	D6RDG3	D6RDG3_HUMAN	BTF3	Transcription factor BTF3 (Fragment)	0.9867	17.27	1	1	1
Q$164-4	H0Y9Y1	H0Y9Y1_HUMAN	BTF3	Transcription factor BTF3 (Fragment)	0.9867	22.09	1	1	1
						VQASLAANTFTITGHAETK	2	0.9867	1
Q$165-1	D6RE86	D6RE86_HUMAN	CP	Ceruloplasmin (Fragment)	1.0000	6.74	1	1	2
						ALYLQYTDETFR	2	0.9993	2
Q$166-1	P31040-2	SDHA_HUMAN	SDHA	Isoform 2 of Succinate dehydrogenase [ubiquinone] flavoprot	0.9850	2.27	1	1	2
Q$166-2	P31040-3	SDHA_HUMAN	SDHA	Isoform 3 of Succinate dehydrogenase [ubiquinone] flavoprot	0.9850	2.7	1	1	2
Q$166-3	D6RFM5	D6RFM5_HUMAN	SDHA	Succinate dehydrogenase [ubiquinone] flavoprotein subunit,	0.9850	2.4	1	1	2
Q$166-4	P31040	SDHA_HUMAN	SDHA	Succinate dehydrogenase [ubiquinone] flavoprotein subunit,	0.9850	2.11	1	1	2
Q$166-5	H0Y8X1	H0Y8X1_HUMAN	SDHA	Succinate dehydrogenase [ubiquinone] flavoprotein subunit,	0.9850	11.11	1	1	2
						LGANSLLDLVVFGR	2	0.985	2
Q$167-1	D6RGJ1	D6RGJ1_HUMAN	C1QB	Complement C1q subcomponent subunit B (Fragment)	1.0000	9.8	2	2	2
						IAFSATR	2	0.9987	1
						TINVPLRR	2	0.9825	1
Q$168-1	D6RHF8	D6RHF8_HUMAN	HPGD	15-hydroxyprostaglandin dehydrogenase [NAD(+)]	1.0000	59.46	2	2	3
Q$168-2	E9PD69	E9PD69_HUMAN	HPGD	15-hydroxyprostaglandin dehydrogenase [NAD(+)]	1.0000	25.29	2	2	3
						AFAEALLLK	2	0.9996	2
						VALVTGAAQGIGR	2	0.9997	1
Q$171-1	P00915	CAH1_HUMAN	CA1	Carbonic anhydrase 1	1.0000	13.41	3	3	5
Q$171-2	E5RFE7	E5RFE7_HUMAN	CA1	Carbonic anhydrase 1 (Fragment)	1.0000	18.04	3	3	5
Q$171-3	E5RHP7	E5RHP7_HUMAN	CA1	Carbonic anhydrase 1 (Fragment)	1.0000	13.94	3	3	5
Q$171-4	E5RJI8	E5RJI8_HUMAN	CA1	Carbonic anhydrase 1 (Fragment)	1.0000	25.55	3	3	5
						ADGLAVIGVLMK	2	0.993	2
						ESISVSSEQLAQFR	2	0.9989	2
						VLDALQAIK	2	0.8972	1
Q$172-1	H0YNP0	H0YNP0_HUMAN	CYFIP1	Cytoplasmic FMR1-interacting protein 1	1.0000	11.69	1	1	1
Q$172-2	Q7L576	CYFP1_HUMAN	CYFIP1	Cytoplasmic FMR1-interacting protein 1	1.0000	0.72	1	1	1
Q$172-3	H0YL39	H0YL39_HUMAN	CYFIP1	Cytoplasmic FMR1-interacting protein 1 (Fragment)	1.0000	5.59	1	1	1
Q$172-4	H0YLJ5	H0YLJ5_HUMAN	CYFIP1	Cytoplasmic FMR1-interacting protein 1 (Fragment)	1.0000	5.96	1	1	1
Q$172-5	E5RFQ0	E5RFQ0_HUMAN	CYFIP2	Cytoplasmic FMR1-interacting protein 2	1.0000	9.47	1	1	1
Q$172-6	E5RJW3	E5RJW3_HUMAN	CYFIP2	Cytoplasmic FMR1-interacting protein 2	1.0000	12.68	1	1	1
Q$172-7	E7EVF4	E7EVF4_HUMAN	CYFIP2	Cytoplasmic FMR1-interacting protein 2	1.0000	0.72	1	1	1
Q$172-8	E7EVJ5	E7EVJ5_HUMAN	CYFIP2	Cytoplasmic FMR1-interacting protein 2	1.0000	0.73	1	1	1
Q$172-9	E7EWA4	E7EWA4_HUMAN	CYFIP2	Cytoplasmic FMR1-interacting protein 2	1.0000	0.7	1	1	1
Q$172-10	Q96F07	CYFP2_HUMAN	CYFIP2	Cytoplasmic FMR1-interacting protein 2	1.0000	0.7	1	1	1
Q$172-11	Q96F07-2	CYFP2_HUMAN	CYFIP2	Isoform 2 of Cytoplasmic FMR1-interacting protein 2	1.0000	0.72	1	1	1
Q$172-12	E7EW33	E7EW33_HUMAN	CYFIP2	Uncharacterized protein	1.0000	0.85	1	1	1
						NAFVTGIAR	2	0.9885	1
Q$173-1	Q8WXA9-2	SREK1_HUMAN	SREK1	Isoform 2 of Splicing regulatory glutamine/lysine-rich protein	0.9856	2.08	1	1	1
Q$173-2	E5RFV3	E5RFV3_HUMAN	SREK1	Splicing regulatory glutamine/lysine-rich protein 1	0.9856	9.85	1	1	1
						TLFSFLGEIEELR	2	0.9856	1
Q$174-1	Q9Y646	CBPQ_HUMAN	CPQ	Carboxypeptidase Q	1.0000	4.45	2	2	2
Q$174-2	E5RH35	E5RH35_HUMAN	CPQ	Carboxypeptidase Q (Fragment)	1.0000	16.15	2	2	2
Q$174-3	E5RJA8	E5RJA8_HUMAN	CPQ	Carboxypeptidase Q (Fragment)	1.0000	11.29	2	2	2
Q$174-4	E5RJP8	E5RJP8_HUMAN	CPQ	Carboxypeptidase Q (Fragment)	1.0000	15.33	2	2	2
Q$174-5	E5RJZ7	E5RJZ7_HUMAN	CPQ	Carboxypeptidase Q (Fragment)	1.0000	16.03	2	2	2
						AIINLAVYGK	2	0.9735	1
						LALLVDTVGPR	2	0.9994	1
Q$175-1	E5RIP5	E5RIP5_HUMAN	EIF3E	Eukaryotic translation initiation factor 3 subunit E	1.0000	28.89	1	1	3
Q$175-2	E5RJ25	E5RJ25_HUMAN	EIF3E	Eukaryotic translation initiation factor 3 subunit E	1.0000	23.21	1	1	3
Q$175-3	E5RHS5	E5RHS5_HUMAN	EIF3E	Eukaryotic translation initiation factor 3 subunit E (Fragment	1.0000	5.75	1	1	3
						HLVFPLLEFLSVK	3	0.9994	3
Q$176-1	E5RII3	E5RII3_HUMAN	EIF3E	Eukaryotic translation initiation factor 3 subunit E	1.0000	41.33	2	2	4
Q$176-2	H0YBP5	H0YBP5_HUMAN	EIF3E	Eukaryotic translation initiation factor 3 subunit E (Fragment	1.0000	41.89	2	2	4
						HLVFPLLEFLSVK	3	0.9994	3
						LDLLSDTNMVDFAMDVYK	2	0.9754	1
Q$177-1	E5RIY1	E5RIY1_HUMAN	PPP2R2A	Serine/threonine-protein phosphatase 2A 55 kDa regulatory	1.0000	8.33	1	1	3
						VVIFQQEQENK	2	0.9989	3
Q$178-1	P11413	G6PD_HUMAN	G6PD	Glucose-6-phosphate 1-dehydrogenase	1.0000	12.82	6	6	7
Q$178-2	E7EM57	E7EM57_HUMAN	G6PD	Glucose-6-phosphate 1-dehydrogenase (Fragment)	1.0000	20.63	6	6	7
Q$178-3	E7EUI8	E7EUI8_HUMAN	G6PD	Glucose-6-phosphate 1-dehydrogenase (Fragment)	1.0000	19.53	6	6	7
Q$178-4	P11413-3	G6PD_HUMAN	G6PD	Isoform 3 of Glucose-6-phosphate 1-dehydrogenase	1.0000	12.11	6	6	7
Q$178-5	P11413-2	G6PD_HUMAN	G6PD	Isoform Long of Glucose-6-phosphate 1-dehydrogenase	1.0000	11.76	6	6	7
						DGLLPENTFIVGYAR	2	0.9596	1
						GGYFDEFGIIR	2	0.9995	2
						IDHYLGK	2	0.9762	1

						LFYLALPPTVYEAVTK	2	0.8824	1
						LSNHISSLFR	3	0.9909	1
						LTVADIR	2	0.9977	1
Q$179-1	E7EMH5	E7EMH5_HUMAN	PDXDC1	Pyridoxal-dependent decarboxylase domain-containing prote	0.9797	2.01	1	1	2
Q$179-2	E7EPL4	E7EPL4_HUMAN	PDXDC1	Pyridoxal-dependent decarboxylase domain-containing prote	0.9797	1.84	1	1	2
Q$179-3	H3BND4	H3BND4_HUMAN	PDXDC1	Pyridoxal-dependent decarboxylase domain-containing prote	0.9797	1.74	1	1	2
Q$179-4	H3BNZ1	H3BNZ1_HUMAN	PDXDC1	Pyridoxal-dependent decarboxylase domain-containing prote	0.9797	1.84	1	1	2
Q$179-5	J3KNK7	J3KNK7_HUMAN	PDXDC1	Pyridoxal-dependent decarboxylase domain-containing prote	0.9797	1.81	1	1	2
Q$179-6	Q6P996	PDXD1_HUMAN	PDXDC1	Pyridoxal-dependent decarboxylase domain-containing prote	0.9797	1.78	1	1	2
						GEDVDQLVACIESK	2	0.9797	2
Q$180-1	E7EMM2	E7EMM2_HUMAN	AP3D1	AP-3 complex subunit delta	1.0000	1.83	2	2	3
Q$180-2	G5E988	G5E988_HUMAN	AP3D1	AP-3 complex subunit delta	1.0000	1.62	2	2	3
Q$180-3	O14617	AP3D1_HUMAN	AP3D1	AP-3 complex subunit delta-1	1.0000	1.56	2	2	3
Q$180-4	O14617-2	AP3D1_HUMAN	AP3D1	Isoform 2 of AP-3 complex subunit delta-1	1.0000	1.62	2	2	3
Q$180-5	O14617-3	AP3D1_HUMAN	AP3D1	Isoform 3 of AP-3 complex subunit delta-1	1.0000	1.83	2	2	3
Q$180-6	O14617-4	AP3D1_HUMAN	AP3D1	Isoform 4 of AP-3 complex subunit delta-1	1.0000	1.76	2	2	3
Q$180-7	O14617-5	AP3D1_HUMAN	AP3D1	Isoform 5 of AP-3 complex subunit delta-1	1.0000	1.48	2	2	3
						ASCILQLVK	2	0.9986	1
						LYASILQQK	2	0.9924	2
Q$181-1	E7EMM4	E7EMM4_HUMAN	ASAH1	Acid ceramidase	1.0000	27.57	8	8	15
Q$181-2	E7ERV9	E7ERV9_HUMAN	ASAH1	Acid ceramidase	1.0000	33.44	8	8	15
Q$181-3	Q13510	ASAH1_HUMAN	ASAH1	Acid ceramidase	1.0000	25.82	8	8	15
Q$181-4	Q13510-2	ASAH1_HUMAN	ASAH1	Isoform 2 of Acid ceramidase	1.0000	24.82	8	8	15
						ESLDVYELDAK	2	0.9992	1
						GQFETYLR	2	0.9932	1
						LPGLLGNFPGPFEEEMK	2	0.9996	3
						LTVYTTLIDVTK	2	0.9997	3
						NMINTFVPSGK	2	0.9753	1
						TSQENISFETMYDVLSTKPVLNK	3	0.9988	3
						WKHPFFLDDR	3	0.9442	2
						WYVVQTNYDR	2	0.9938	1
Q$182-1	E7EMS6	E7EMS6_HUMAN	COMT	Catechol O-methyltransferase (Fragment)	1.0000	13.9	2	4	18
						LITIEINPDCAAITQR	2	0.9997	7
						LITIEINPDCAAITQR	3	0.9994	3
						YLPDTLLLEECGLLR	2	0.9997	5
						YLPDTLLLEECGLLR	3	0.9995	3
Q$183-1	P04070-2	PROC_HUMAN	PROC	Isoform 2 of Vitamin K-dependent protein C	1.0000	23.64	10	14	34
Q$183-2	P04070	PROC_HUMAN	PROC	Vitamin K-dependent protein C	1.0000	26.46	10	14	34
Q$183-3	E7END6	E7END6_HUMAN	PROC	Vitamin K-dependent protein C heavy chain	1.0000	24.65	10	14	34
						DTEDQEDQVDPR	2	0.9996	3
						ELNQAGQETLVTGWGYHSSR	2	0.9997	3
						ELNQAGQETLVTGWGYHSSR	3	0.985	1
						LGDDLLQCHPAVK	2	0.9996	4
						LGDDLLQCHPAVK	3	0.9972	1
						LGEYDLR	2	0.9988	2
						RGDSPWQVVLLDSK	2	0.9992	2
						RGDSPWQVVLLDSK	3	0.9995	3
						RGDSPWQVVLLDSKK	3	0.9984	2
						RWEKWELDLDIK	2	0.9356	2
						RWEKWELDLDIK	3	0.8513	1
						STTDNDIALLHLAQPATLSQTIVPICLPDSGLAER	3	0.9991	6
						TFVLNFIK	2	0.9997	2
						WELDLDIK	2	0.9926	2
Q$184-1	P61313	RL15_HUMAN	RPL15	60S ribosomal protein L15	1.0000	12.75	2	2	4
Q$184-2	E7ENU7	E7ENU7_HUMAN	RPL15	Ribosomal protein L15 (Fragment)	1.0000	14.86	2	2	4
Q$184-3	E7EQV9	E7EQV9_HUMAN	RPL15	Ribosomal protein L15 (Fragment)	1.0000	14.94	2	2	4
						FFEVILIDPFHK	3	0.9992	2
						VLNSYWVGEDSTYK	2	0.9996	2
Q$185-1	E7EP32	E7EP32_HUMAN	GNB2	Guanine nucleotide-binding protein G(I)/G(S)/G(T) subunit be	1.0000	33.78	6	6	8
Q$185-2	P62879	GBB2_HUMAN	GNB2	Guanine nucleotide-binding protein G(I)/G(S)/G(T) subunit be	1.0000	29.41	6	6	8
						ADQELLMYSHDNIICGITSVAFSR	3	0.9995	3
						ELPGHTGYLSCCR	2	0.9377	1
						LLLAGYDDFNCNIWDAMK	2	0.9976	1
						LLVSASQDGK	2	0.9815	1
						TFVSGACDASIK	2	0.9995	1
						VSCLGVTDDGMAVATGSWDSFLK	2	0.9993	1
Q$186-1	E7EPB3	E7EPB3_HUMAN	RPL14	60S ribosomal protein L14	1.0000	18.55	2	3	5
Q$186-2	P50914	RL14_HUMAN	RPL14	60S ribosomal protein L14	1.0000	10.7	2	3	5
						ALVDGPCTQVR	2	0.979	2
						LVAIVDVIDQNR	2	0.9997	2
						LVAIVDVIDQNR	3	0.9493	1
Q$187-1	E7EPC6	E7EPC6_HUMAN	CD44	CD44 antigen	0.9826	1.68	1	1	2
Q$187-2	H0YD13	H0YD13_HUMAN	CD44	CD44 antigen	0.9826	5.83	1	1	2
Q$187-3	P16070	CD44_HUMAN	CD44	CD44 antigen	0.9826	1.62	1	1	2
Q$187-4	H0Y2P0	H0Y2P0_HUMAN	CD44	CD44 antigen (Fragment)	0.9826	4.18	1	1	2
Q$187-5	H0Y5E4	H0Y5E4_HUMAN	CD44	CD44 antigen (Fragment)	0.9826	4.08	1	1	2
Q$187-6	H0YCV9	H0YCV9_HUMAN	CD44	CD44 antigen (Fragment)	0.9826	4.82	1	1	2
Q$187-7	H0YD17	H0YD17_HUMAN	CD44	CD44 antigen (Fragment)	0.9826	5.24	1	1	2
Q$187-8	H0YDW7	H0YDW7_HUMAN	CD44	CD44 antigen (Fragment)	0.9826	5	1	1	2
Q$187-9	H0YDX6	H0YDX6_HUMAN	CD44	CD44 antigen (Fragment)	0.9826	6.63	1	1	2
Q$187-10	P16070-10	CD44_HUMAN	CD44	Isoform 10 of CD44 antigen	0.9826	2.43	1	1	2
Q$187-11	P16070-11	CD44_HUMAN	CD44	Isoform 11 of CD44 antigen	0.9826	2.8	1	1	2
Q$187-12	P16070-12	CD44_HUMAN	CD44	Isoform 12 of CD44 antigen	0.9826	3.32	1	1	2
Q$187-13	P16070-13	CD44_HUMAN	CD44	Isoform 13 of CD44 antigen	0.9826	2.82	1	1	2
Q$187-14	P16070-14	CD44_HUMAN	CD44	Isoform 14 of CD44 antigen	0.9826	3.03	1	1	2
Q$187-15	P16070-15	CD44_HUMAN	CD44	Isoform 15 of CD44 antigen	0.9826	4.08	1	1	2
Q$187-16	P16070-16	CD44_HUMAN	CD44	Isoform 16 of CD44 antigen	0.9826	1.8	1	1	2
Q$187-17	P16070-17	CD44_HUMAN	CD44	Isoform 17 of CD44 antigen	0.9826	1.74	1	1	2
Q$187-18	P16070-18	CD44_HUMAN	CD44	Isoform 18 of CD44 antigen	0.9826	3.53	1	1	2
Q$187-19	P16070-3	CD44_HUMAN	CD44	Isoform 3 of CD44 antigen	0.9826	1.69	1	1	2
Q$187-20	P16070-4	CD44_HUMAN	CD44	Isoform 4 of CD44 antigen	0.9826	1.72	1	1	2
Q$187-21	P16070-5	CD44_HUMAN	CD44	Isoform 5 of CD44 antigen	0.9826	1.63	1	1	2
Q$187-22	P16070-6	CD44_HUMAN	CD44	Isoform 6 of CD44 antigen	0.9826	1.72	1	1	2
Q$187-23	P16070-7	CD44_HUMAN	CD44	Isoform 7 of CD44 antigen	0.9826	1.68	1	1	2
Q$187-24	P16070-8	CD44_HUMAN	CD44	Isoform 8 of CD44 antigen	0.9826	1.78	1	1	2
Q$187-25	P16070-9	CD44_HUMAN	CD44	Isoform 9 of CD44 antigen	0.9826	1.78	1	1	2
						YGFIEGHVVIPR	3	0.9826	2
Q$188-1	E7EQ64	E7EQ64_HUMAN	PRSS1	Trypsin-1	0.9359	3.83	1	1	1
Q$188-2	P07477	TRY1_HUMAN	PRSS1	Trypsin-1	0.9359	4.05	1	1	1
Q$188-3	H0Y8D1	H0Y8D1_HUMAN	PRSS1	Trypsin-1 (Fragment)	0.9359	7.04	1	1	1
						TLNNDIMLIK	2	0.9359	1
Q$189-1	E7EQB2	E7EQB2_HUMAN	LTF	Kaliocin-1 (Fragment)	1.0000	55.32	30	35	79
Q$189-2	P02788	TRFL_HUMAN	LTF	Lactotransferrin	1.0000	54.23	30	35	79
						ADAVTLDGGFIYEAGLAPYK	2	0.9937	1
						CAFSSQEPYFSYSGAFK	2	0.9996	4
						CGLVPVLAENYK	2	0.9991	1
						CLAENAGDVAFVK	2	0.9995	3
						CSTSPLLEACEFLR	2	0.9869	2
						DEYFSQSCAPGSDPR	2	0.9934	2
						DGAGDVAFIR	2	0.9985	2
						DGAGDVAFIRE	2	0.8743	2
						DLLFKDSAIGFSR	3	0.8982	1
						DSPIQCIQAIAENR	2	0.999	4
						DSPIQCIQAIAENR	3	0.8833	1
						ESTVFEDLSDEAERDEYELLCPDNTR	3	0.9601	2
						GEADAMSLDGGYVYTAGK	2	0.9996	3
						GGSFQLNELQGLK	2	0.999	3
						IDSGLYLGSGYFTAIQNLR	2	0.9996	3
						IDSGLYLGSGYFTAIQNLR	3	0.9962	3
						KCNQWSGLSEGSVTCSSASTTEDCIALVLK	3	0.9633	1
						KGGSFQLNELQGLK	2	0.9974	3
						KGGSFQLNELQGLK	3	0.9823	1
						LADFALLCLDGK	2	0.9995	3

						LLFNDNTECLAR	2	0.9723	2
						LRPVAAEVYGTER	2	0.999	2
						LRPVAAEVYGTER	3	0.9989	1
						NLLFNDNTECLAR	2	0.9994	4
						SDTSLTWNSVK	2	0.9971	2
						SNLCALCIGDEQGENK	2	0.9996	2
						SQQSSDPDPNCVDRPVEGYLAVAVVR	3	0.9986	4
						SVQWCAVSQPEATK	2	0.9996	2
						THYYAVAVVK	2	0.9902	1
						THYYAVAVVK	3	0.9251	1
						TLDGGFIYEAGLAPYK	2	0.9876	3
						VVWCAVGEQELR	2	0.9995	4
						VVWCAVGEQELRK	2	0.999	2
						YLGPQYVAGITNLK	2	0.9991	2
						YYGYTGAFR	2	0.9983	2
Q$190-1	P60842	IF4A1_HUMAN	EIF4A1	Eukaryotic initiation factor 4A-I	1.0000	7.39	2	3	3
Q$190-2	E7EQG2	E7EQG2_HUMAN	EIF4A2	Eukaryotic initiation factor 4A-II	1.0000	8.29	2	3	3
Q$190-3	Q14240	IF4A2_HUMAN	EIF4A2	Eukaryotic initiation factor 4A-II	1.0000	7.37	2	3	3
Q$190-4	Q14240-2	IF4A2_HUMAN	EIF4A2	Isoform 2 of Eukaryotic initiation factor 4A-II	1.0000	7.35	2	3	3
						GIYAYGFEKPSAIQQR	2	0.9996	1
						GIYAYGFEKPSAIQQR	3	0.9939	1
						GYDVIAQAQSGTGK	2	0.9982	1
Q$191-1	E7EQI7	E7EQI7_HUMAN	KIAA0196	WASH complex subunit strumpellin	0.9977	1.48	1	1	2
Q$191-2	Q12768	STRUM_HUMAN	KIAA0196	WASH complex subunit strumpellin	0.9977	1.29	1	1	2
						SFEYIQDYVNIYGLK	2	0.9902	2
Q$192-1	E7EQR4	E7EQR4_HUMAN	EZR	Ezrin	1.0000	15.52	7	7	14
Q$192-2	P15311	EZRI_HUMAN	EZR	Ezrin	1.0000	14.68	7	7	14
						AKFYPEDVAEELIQDITQK	3	0.9977	3
						APDFVFYAPR	2	0.9896	1
						IALLEEAR	2	0.9929	1
						IAQDLEMYGINYFEIK	2	0.9996	3
						IGFPWSEIR	2	0.9994	2
						IQVWHAEHR	2	0.9996	2
						SQEQLAAELAEYTAK	2	0.9996	2
Q$193-1	E7EQR6	E7EQR6_HUMAN	TCP1	T-complex protein 1 subunit alpha	1.0000	7.23	3	3	5
Q$193-2	F5H282	F5H282_HUMAN	TCP1	T-complex protein 1 subunit alpha	1.0000	8.73	3	3	5
						EQLAIAEFAR	2	0.9985	2
						FATEAAITILR	2	0.9996	2
						TSASIILR	2	0.9936	1
Q$194-1	E7ER27	E7ER27_HUMAN	HSD17B4	Peroxisomal multifunctional enzyme type 2	1.0000	4.4	2	2	2
Q$194-2	P51659	DHB4_HUMAN	HSD17B4	Peroxisomal multifunctional enzyme type 2	1.0000	2.99	2	2	2
						AYALAFAER	2	0.9946	1
						VVLVTGAGAGLGR	2	0.9992	1
Q$195-1	E7ER44	E7ER44_HUMAN	LTF	Kaliocin-1	1.0000	50.28	29	34	78
						ADAVTLDGGFIYEAGLAPYK	2	0.9937	1
						CAFSSQEPYFSYSGAFK	2	0.9996	4
						CGLVPVLAENYK	2	0.9991	1
						CLAENAGDVAFVK	2	0.9995	3
						CSTSPLLEACEFLR	2	0.9869	2
						DEYFSQSCAPGSDPR	2	0.9934	2
						DGAGDVAFIR	2	0.9985	2
						DGAGDVAFIRE	2	0.8743	2
						DLLFKDSAIGFSR	3	0.8982	1
						DSPIQCIQAIAENR	2	0.999	4
						DSPIQCIQAIAENR	3	0.8833	1
						ESTVFEDLSDEAERDEYELLCPDNTR	3	0.9601	2
						GEADAMSLDGGYVYTAGK	2	0.9996	3
						GGSFQLNELQGLK	2	0.999	3
						IDSGLYLGSGYFTAIQNLR	2	0.9996	3
						IDSGLYLGSGYFTAIQNLR	3	0.9962	3
						KGGSFQLNELQGLK	2	0.9974	3
						KGGSFQLNELQGLK	3	0.9823	1
						LADFALLCLDGK	2	0.9995	3
						LLFNDNTECLAR	2	0.9723	2
						LRPVAAEVYGTER	2	0.999	2
						LRPVAAEVYGTER	3	0.9989	1
						NLLFNDNTECLAR	2	0.9994	4
						SDTSLTWNSVK	2	0.9971	2
						SNLCALCIGDEQGENK	2	0.9996	2
						SQQSSDPDPNCVDRPVEGYLAVAVVR	3	0.9986	4
						SVQWCAVSQPEATK	2	0.9996	2
						THYYAVAVVK	2	0.9902	1
						THYYAVAVVK	3	0.9251	1
						TLDGGFIYEAGLAPYK	2	0.9876	3
						VVWCAVGEQELR	2	0.9995	4
						VVWCAVGEQELRK	2	0.999	2
						YLGPQYVAGITNLK	2	0.9991	2
						YYGYTGAFR	2	0.9983	2
Q$196-1	E9PFL9	E9PFL9_HUMAN	SSB	Lupus La protein	1.0000	22.39	2	3	6
Q$196-2	P05455	LA_HUMAN	SSB	Lupus La protein	1.0000	7.35	2	3	6
Q$196-3	E7ERC4	E7ERC4_HUMAN	SSB	Lupus La protein (Fragment)	1.0000	16.3	2	3	6
						ICHQIEYYFGDFNLPR	2	0.9695	1
						ICHQIEYYFGDFNLPR	3	0.9717	2
						LTTDFNVIVEALSK	2	0.9996	3
Q$197-1	P15531-2	NDKA_HUMAN	NME1	Isoform 2 of Nucleoside diphosphate kinase A	0.9508	5.08	1	1	1
Q$197-2	E7ERL0	E7ERL0_HUMAN	NME1	Nucleoside diphosphate kinase A	0.9508	6.47	1	1	1
Q$197-3	P15531	NDKA_HUMAN	NME1	Nucleoside diphosphate kinase A	0.9508	5.92	1	1	1
Q$197-4	F6XY72	F6XY72_HUMAN	NME1-NME2	Nucleoside diphosphate kinase	0.9508	11.69	1	1	1
Q$197-5	Q32Q12	Q32Q12_HUMAN	NME1-NME2	Nucleoside diphosphate kinase	0.9508	3.08	1	1	1
Q$197-6	P22392-2	NDKB_HUMAN	NME2	Isoform 3 of Nucleoside diphosphate kinase B	0.9508	3.37	1	1	1
Q$197-7	J3KPD9	J3KPD9_HUMAN	NME2	Nucleoside diphosphate kinase B	0.9508	4.57	1	1	1
Q$197-8	P22392	NDKB_HUMAN	NME2	Nucleoside diphosphate kinase B	0.9508	5.92	1	1	1
Q$197-9	O60361	NDK8_HUMAN	NME2P1	Putative nucleoside diphosphate kinase	0.9508	6.57	1	1	1
						GDFCIQVGR	2	0.9508	1
Q$198-1	E7ES19	E7ES19_HUMAN	THBS4	Thrombospondin-4	1.0000	7.01	3	3	5
Q$198-2	P35443	TSP4_HUMAN	THBS4	Thrombospondin-4	1.0000	6.35	3	3	5
						DGIGDECDDDDDNDGIPDLVPPGPDNCR	3	0.8176	1
						DVDIDSYPDEELPCSAR	2	0.9994	3
						PELNPCSVNAQCIEER	2	0.8175	1
Q$199-1	F5GYB5	F5GYB5_HUMAN	RAP1B	Ras-related protein Rap-1b	1.0000	8.09	1	1	1
Q$199-2	F5H7Y6	F5H7Y6_HUMAN	RAP1B	Ras-related protein Rap-1b	1.0000	6.15	1	1	1
Q$199-3	E7ESV4	E7ESV4_HUMAN	RAP1B	Ras-related protein Rap-1b (Fragment)	1.0000	7.01	1	1	1
Q$199-4	F5GWU8	F5GWU8_HUMAN	RAP1B	Ras-related protein Rap-1b (Fragment)	1.0000	12.64	1	1	1
Q$199-5	F5GX62	F5GX62_HUMAN	RAP1B	Ras-related protein Rap-1b (Fragment)	1.0000	7.91	1	1	1
Q$199-6	F5GYH7	F5GYH7_HUMAN	RAP1B	Ras-related protein Rap-1b (Fragment)	1.0000	20.75	1	1	1
Q$199-7	F5H004	F5H004_HUMAN	RAP1B	Ras-related protein Rap-1b (Fragment)	1.0000	8.66	1	1	1
Q$199-8	F5H077	F5H077_HUMAN	RAP1B	Ras-related protein Rap-1b (Fragment)	1.0000	13.1	1	1	1
Q$199-9	F5H0B7	F5H0B7_HUMAN	RAP1B	Ras-related protein Rap-1b (Fragment)	1.0000	11.22	1	1	1
Q$199-10	F5H491	F5H491_HUMAN	RAP1B	Ras-related protein Rap-1b (Fragment)	1.0000	11.83	1	1	1
Q$199-11	F5H4H0	F5H4H0_HUMAN	RAP1B	Ras-related protein Rap-1b (Fragment)	1.0000	22.92	1	1	1
Q$199-12	F5H500	F5H500_HUMAN	RAP1B	Ras-related protein Rap-1b (Fragment)	1.0000	10.89	1	1	1
Q$199-13	F5H6R7	F5H6R7_HUMAN	RAP1B	Ras-related protein Rap-1b (Fragment)	1.0000	9.02	1	1	1
Q$199-14	F8WBC0	F8WBC0_HUMAN	RAP1B	Ras-related protein Rap-1b (Fragment)	1.0000	34.38	1	1	1
						LVVLGSGGVGK	2	0.9992	1
Q$200-1	P62136-2	PP1A_HUMAN	PPP1CA	Isoform 2 of Serine/threonine-protein phosphatase PP1-alph	1.0000	4.99	1	1	3
Q$200-2	E9PMD7	E9PMD7_HUMAN	PPP1CA	Serine/threonine-protein phosphatase (Fragment)	1.0000	6.72	1	1	3
Q$200-3	P62136	PP1A_HUMAN	PPP1CA	Serine/threonine-protein phosphatase PP1-alpha catalytic su	1.0000	5.15	1	1	3
Q$200-4	F5H037	F5H037_HUMAN	PPP1CA	Serine/threonine-protein phosphatase PP1-alpha catalytic su	1.0000	9.5	1	1	3
Q$200-5	F5H1L6	F5H1L6_HUMAN	PPP1CA	Serine/threonine-protein phosphatase PP1-alpha catalytic su	1.0000	10.06	1	1	3
Q$200-6	E7ETD8	E7ETD8_HUMAN	PPP1CB	Serine/threonine-protein phosphatase (Fragment)	1.0000	10.06	1	1	3
Q$200-7	F8WE71	F8WE71_HUMAN	PPP1CB	Serine/threonine-protein phosphatase PP1-beta catalytic sub	1.0000	26.56	1	1	3
Q$200-8	P36873-2	PP1G_HUMAN	PPP1CC	Isoform Gamma-2 of Serine/threonine-protein phosphatase I	1.0000	5.04	1	1	3
Q$200-9	F8VR82	F8VR82_HUMAN	PPP1CC	Serine/threonine-protein phosphatase	1.0000	6.3	1	1	3

Q$200-10	F8VYE8	F8VYE8_HUMAN	PPP1CC	Serine/threonine-protein phosphatase	1.0000	5.59	1	1	3
Q$200-11	F8W0W8	F8W0W8_HUMAN	PPP1CC	Serine/threonine-protein phosphatase	1.0000	5.12	1	1	3
Q$200-12	P36873	PP1G_HUMAN	PPP1CC	Serine/threonine-protein phosphatase PP1-gamma catalytic	1.0000	5.26	1	1	3
						EIFLSQPILLELEAPLK	2	0.9997	3
Q$201-1	P62820-3	RAB1A_HUMAN	RAB1A	Isoform 3 of Ras-related protein Rab-1A	1.0000	23.26	2	2	4
Q$201-2	E7ETK2	E7ETK2_HUMAN	RAB1A	Ras-related protein Rab-1A	1.0000	23.26	2	2	4
						FADDTYTESYISTIGVDFK	2	0.9994	2
						LLLIGDSGVGK	2	0.985	2
Q$202-1	E7ETK5	E7ETK5_HUMAN	IMPDH2	Inosine-5'-monophosphate dehydrogenase 2 (Fragment)	1.0000	12.54	3	4	6
						HGFCGIPITDTGR	2	0.9992	1
						LVGIISSR	2	0.996	1
						REDLVVAPAGITLK	2	0.9996	2
						REDLVVAPAGITLK	3	0.998	2
Q$203-1	P06681	CO2_HUMAN	C2	Complement C2	1.0000	1.35	1	1	2
						CPAPVSFENGIYTPR	2	0.9991	2
Q$204-1	P50395-2	GDIB_HUMAN	GDI2	Isoform 2 of Rab GDP dissociation inhibitor beta	1.0000	17.5	6	7	10
Q$204-2	E7EU23	E7EU23_HUMAN	GDI2	Rab GDP dissociation inhibitor beta	1.0000	15.59	6	7	10
Q$204-3	P50395	GDIB_HUMAN	GDI2	Rab GDP dissociation inhibitor beta	1.0000	15.73	6	7	10
						DLGTESQIFISR	2	0.9995	2
						FDLGQDVIDFTGHALALYR	3	0.9737	1
						FVSISDLLVPK	2	0.9936	1
						KFDLGQDVIDFTGHALALYR	3	0.9996	3
						KFDLGQDVIDFTGHALALYR	4	0.9903	1
						LYSESLAR	2	0.976	1
						SPYLYPLYGLGELPQGFAR	2	0.9995	1
Q$205-1	Q16851-2	UGPA_HUMAN	UGP2	Isoform 2 of UTP--glucose-1-phosphate uridylyltransferase	0.9997	7.85	3	3	3
Q$205-2	E7EUC7	E7EUC7_HUMAN	UGP2	UTP--glucose-1-phosphate uridylyltransferase	0.9997	7.54	3	3	3
Q$205-3	Q16851	UGPA_HUMAN	UGP2	UTP--glucose-1-phosphate uridylyltransferase	0.9997	7.68	3	3	3
						LVEIAQVPK	2	0.9953	1
						NENTFLDLTVQQIEHLNK	3	0.982	1
						SFENSLGINVPR	2	0.9063	1
Q$206-1	E7EUT5	E7EUT5_HUMAN	GAPDH	Glyceraldehyde-3-phosphate dehydrogenase	1.0000	66.92	14	18	69
						AGAEYVVESTGVFTTMEK	2	0.8445	1
						GALQNIIPASTGAAK	2	0.9997	3
						GILGYTEHQVVSSDFNSDTHSSTFDAGAGIALNDHFVK	4	0.9881	3
						IISNASCTTNCLAPLAK	2	0.9997	4
						LEKPAKYDDIK	2	0.9917	3
						LEKPAKYDDIKK	2	0.999	4
						LISWYDNEFGYSNR	2	0.9998	9
						LISWYDNEFGYSNR	3	0.9993	3
						LTGMAFRVPTANVSVVDLTCR	3	0.9994	3
						SWYDNEFGYSNR	2	0.9964	3
						VIHDNFGIVEGLMTTV	2	0.9446	1
						VIHDNFGIVEGLMTTVHAITATQK	3	0.9996	11
						VIHDNFGIVEGLMTTVHAITATQK	4	0.9981	4
						VPTANVSVVDLTCR	2	0.9998	5
						VVDLMAHMASKE	2	0.9997	2
						VVDLMAHMASKE	3	0.9841	2
						WGDAGAEYVVESTGVFTTMEK	2	0.9997	5
						WGDAGAEYVVESTGVFTTMEK	3	0.9996	3
Q$207-1	Q9UM54-1	MYO6_HUMAN	MYO6	Isoform 1 of Unconventional myosin-VI	1.0000	6.77	5	6	8
Q$207-2	Q9UM54-2	MYO6_HUMAN	MYO6	Isoform 2 of Unconventional myosin-VI	1.0000	6.89	5	6	8
Q$207-3	Q9UM54-4	MYO6_HUMAN	MYO6	Isoform 4 of Unconventional myosin-VI	1.0000	6.77	5	6	8
Q$207-4	Q9UM54-5	MYO6_HUMAN	MYO6	Isoform 5 of Unconventional myosin-VI	1.0000	6.94	5	6	8
Q$207-5	Q9UM54-6	MYO6_HUMAN	MYO6	Isoform 6 of Unconventional myosin-VI	1.0000	6.77	5	6	8
Q$207-6	E7EW20	E7EW20_HUMAN	MYO6	Unconventional myosin-VI	1.0000	6.77	5	6	8
Q$207-7	Q9UM54	MYO6_HUMAN	MYO6	Unconventional myosin-VI	1.0000	6.72	5	6	8
						IAQSEAELISDEAQADLALR	2	0.9996	2
						LCAGASEDIREK	2	0.9782	2
						MSQSIIVSGESGAGKTENTK	2	0.9977	1
						SAQSLEYCAELLGLDQDDLR	2	0.9989	1
						SAQSLEYCAELLGLDQDDLR	3	0.9987	1
						YLTESYGTGQDIDDR	2	0.9989	1
Q$208-1	E7EW44	E7EW44_HUMAN	CORO1B	Coronin	1.0000	8.3	2	2	4
Q$208-2	F5H0D2	F5H0D2_HUMAN	CORO1B	Coronin	1.0000	13.11	2	2	4
Q$208-3	F5H390	F5H390_HUMAN	CORO1B	Coronin	1.0000	8.51	2	2	4
						VGIIAWHPTAR	3	0.9992	2
						VTWDSTFCAVNPK	2	0.9996	2
Q$209-1	E7EWT1	E7EWT1_HUMAN	DDOST	Dolichyl-diphosphooligosaccharide--protein glycosyltransfera	1.0000	2.63	1	1	2
						TLVLLDNLNVR	2	0.9994	2
Q$210-1	E9PAV3-2	NACAM_HUMAN	NACA	Isoform 2 of Nascent polypeptide-associated complex subuni	1.0000	2.92	2	2	4
Q$210-2	Q13765	NACA_HUMAN	NACA	Nascent polypeptide-associated complex subunit alpha	1.0000	12.56	2	2	4
Q$210-3	E9PAV3	NACAM_HUMAN	NACA	Nascent polypeptide-associated complex subunit alpha, mus	1.0000	1.3	2	2	4
Q$210-4	F8VZJ2	F8VZJ2_HUMAN	NACA	Nascent polypeptide-associated complex subunit alpha, mus	1.0000	19.85	2	2	4
Q$210-5	F8VNW4	F8VNW4_HUMAN	NACA	Nascent polypeptide-associated complex subunit alpha, mus	1.0000	18.24	2	2	4
Q$210-6	F8W0W4	F8W0W4_HUMAN	NACA	Nascent polypeptide-associated complex subunit alpha, mus	1.0000	13.64	2	2	4
Q$210-7	F8W1N5	F8W1N5_HUMAN	NACA	Nascent polypeptide-associated complex subunit alpha, mus	1.0000	38.03	2	2	4
Q$210-8	H0YHX9	H0YHX9_HUMAN	NACA	Nascent polypeptide-associated complex subunit alpha, mus	1.0000	12.68	2	2	4
						NILFVITKPDVYK	2	0.9979	2
						SPASDTYIVFGEAK	2	0.9997	2
Q$211-1	E9PB90	E9PB90_HUMAN	HK2	Hexokinase-2	0.9999	2.47	2	2	2
Q$211-2	P52789	HXK2_HUMAN	HK2	Hexokinase-2	0.9999	2.4	2	2	2
						GAALITAVACR	2	0.9987	1
						NVELVEGEEGR	2	0.9554	1
Q$212-1	E9PBC5	E9PBC5_HUMAN	KLKB1	Plasma kallikrein heavy chain	1.0000	2.54	1	1	2
						IAYGTQGSSGYSLR	2	0.9994	2
Q$213-1	E9PBW4	E9PBW4_HUMAN	HBG2	Hemoglobin subunit gamma-2	1.0000	16.06	2	2	3
						EFTPEVQASWQK	2	0.9978	2
						VLTSLGDAIK	2	0.9761	1
Q$214-1	E9PBZ2	E9PBZ2_HUMAN	HPGD	15-hydroxyprostaglandin dehydrogenase [NAD(+)]	1.0000	30.68	5	5	10
Q$214-2	P15428-4	PGDH_HUMAN	HPGD	Isoform 4 of 15-hydroxyprostaglandin dehydrogenase [NAD(	1.0000	37.76	5	5	10
						AALDEQFEPQK	2	0.9997	4
						AFAEALLK	2	0.9996	2
						LDILVNNAGVNNEK	2	0.9997	2
						VALVTGAAQGIGR	2	0.9997	1
						VVDHFGR	2	0.9946	1
Q$215-1	P24821-2	TENA_HUMAN	TNC	Isoform 2 of Tenascin	0.9992	0.69	1	1	2
Q$215-2	P24821-3	TENA_HUMAN	TNC	Isoform 3 of Tenascin	0.9992	0.65	1	1	2
Q$215-3	P24821-4	TENA_HUMAN	TNC	Isoform 4 of Tenascin	0.9992	0.57	1	1	2
Q$215-4	P24821-5	TENA_HUMAN	TNC	Isoform 5 of Tenascin	0.9992	0.73	1	1	2
Q$215-5	P24821-6	TENA_HUMAN	TNC	Isoform 6 of Tenascin	0.9992	0.77	1	1	2
Q$215-6	E9PC84	E9PC84_HUMAN	TNC	Tenascin	0.9992	0.62	1	1	2
Q$215-7	F5H7V9	F5H7V9_HUMAN	TNC	Tenascin	0.9992	0.65	1	1	2
Q$215-8	J3QSU6	J3QSU6_HUMAN	TNC	Tenascin	0.9992	0.59	1	1	2
Q$215-9	P24821	TENA_HUMAN	TNC	Tenascin	0.9992	0.55	1	1	2
						LEELENLVSSLR	2	0.9987	2
Q$216-1	E9PCY7	E9PCY7_HUMAN	HNRNPH1	Heterogeneous nuclear ribonucleoprotein H	1.0000	6.29	2	2	4
Q$216-2	G8JLB6	G8JLB6_HUMAN	HNRNPH1	Heterogeneous nuclear ribonucleoprotein H	1.0000	5.72	2	2	4
Q$216-3	P31943	HNRH1_HUMAN	HNRNPH1	Heterogeneous nuclear ribonucleoprotein H	1.0000	6.01	2	2	4
Q$216-4	H0YB39	H0YB39_HUMAN	HNRNPH1	Heterogeneous nuclear ribonucleoprotein H (Fragment)	1.0000	9.75	2	2	4
Q$216-5	P55795	HNRH2_HUMAN	HNRNPH2	Heterogeneous nuclear ribonucleoprotein H2	1.0000	6.01	2	2	4
						STGEAFVQFASQEIAEK	2	0.9992	2
						VHIEIGPDGR	2	0.9986	2
Q$217-1	E9PEW8	E9PEW8_HUMAN	HBD	Hemoglobin subunit delta (Fragment)	1.0000	14.42	1	1	1
						SELHCDKLHVDPENF	2	0.8135	1
Q$218-1	F8VTZ0	F8VTZ0_HUMAN	PCBP2	Poly(rC)-binding protein 2 (Fragment)	0.9995	17.19	1	1	2
Q$218-2	P57721-2	PCBP3_HUMAN	PCBP3	Isoform 2 of Poly(rC)-binding protein 3	0.9995	3.19	1	1	2
Q$218-3	P57721-3	PCBP3_HUMAN	PCBP3	Isoform 3 of Poly(rC)-binding protein 3	0.9995	3.18	1	1	2
Q$218-4	P57721-4	PCBP3_HUMAN	PCBP3	Isoform 4 of Poly(rC)-binding protein 3	0.9995	2.97	1	1	2
Q$218-5	P57721-5	PCBP3_HUMAN	PCBP3	Isoform 5 of Poly(rC)-binding protein 3	0.9995	3.13	1	1	2
Q$218-6	E9PFP8	E9PFP8_HUMAN	PCBP3	Poly(rC)-binding protein 3	0.9995	3.05	1	1	2
Q$218-7	P57721	PCBP3_HUMAN	PCBP3	Poly(rC)-binding protein 3	0.9995	2.96	1	1	2
Q$218-8	J3QT27	J3QT27_HUMAN	PCBP3	Poly(rC)-binding protein 3 (Fragment)	0.9995	3.42	1	1	2

						INISEGNCPER	2	0.9946	2
Q$219-1	E9PFZ2	E9PFZ2_HUMAN	CP	Ceruloplasmin	1.0000	9.94	9	9	13
Q$219-2	P00450	CERU_HUMAN	CP	Ceruloplasmin	1.0000	8.83	9	9	13
						ALYLQYTDETFR	2	0.9993	2
						DIASGLIGPLIICK	2	0.9676	1
						EVGPTNADPVCLAK	2	0.9281	1
						EYTDASFTNR	2	0.8086	1
						EYTDASFTNRK	2	0.9964	2
						GAYPLSIEPIGVR	2	0.9988	2
						QSEDSTFYLGER	2	0.9816	2
						RQSEDSTFYLGER	2	0.8999	1
						TTIEKPVWLGFLGPIIK	3	0.8493	1
Q$220-1	E9PG40	E9PG40_HUMAN	APP	Gamma-secretase C-terminal fragment 59	1.0000	20.45	11	11	22
						CAPFFYGGCGGNR	2	0.9997	2
						CLVGEFVSDALLVPDKCK	3	0.9913	1
						EVCSEQAETGPCR	2	0.9997	3
						ISYGNDALMPSLTETK	2	0.9989	2
						LALENYITALQAVPPRPR	3	0.9754	1
						QQLVETHMAR	2	0.9832	1
						SQVMTHLR	2	0.9979	2
						STNLHDYGMLLPCGIDKFR	3	0.9475	1
						THPHFVIPYR	3	0.9994	3
						VESLEQEAANER	2	0.9975	2
						WYFDVTEGK	2	0.9997	4
Q$221-1	E9PGP2	E9PGP2_HUMAN	F11	Coagulation factor XI	1.0000	22.16	9	9	15
Q$221-2	P03951	FA11_HUMAN	F11	Coagulation factor XI	1.0000	20.32	9	9	15
Q$221-3	P03951-2	FA11_HUMAN	F11	Isoform 2 of Coagulation factor XI	1.0000	22.24	9	9	15
						CLLFTFTAESPSEDPTR	2	0.9997	3
						DTCFEGGDITTVFTPSAK	2	0.9997	2
						ERPGVYTNVVEYVDWILEK	2	0.9952	3
						GGISGYTLR	2	0.9935	1
						HNEVWHLVGITSWGEGCAQR	3	0.9979	2
						NVIYTDCWVTGWGYR	2	0.997	1
						SCALSNLACIR	2	0.9262	1
						WFTCVLK	2	0.974	1
						YCQVVCTYHPR	2	0.9994	1
Q$222-1	E9PGX9	E9PGX9_HUMAN	SSB	Lupus La protein (Fragment)	1.0000	11.67	1	1	3
						LTTDFNVIVEALSK	2	0.9996	3
Q$223-1	E9PHH3	E9PHH3_HUMAN	SDC1	Syndecan-1	1.0000	10.06	3	4	8
Q$223-2	P18827	SDC1_HUMAN	SDC1	Syndecan-1	1.0000	5.48	3	4	8
Q$223-3	H7C1K4	H7C1K4_HUMAN	SDC1	Syndecan-1 (Fragment)	1.0000	7.91	3	4	8
						EGEAVVLPEVEPGLTAR	2	0.9997	5
						EGEAVVLPEVEPGLTAR	3	0.9862	1
						PEVEPGLTAR	2	0.9948	1
						VLPEVEPGLTAR	2	0.8542	1
Q$224-1	E9PHK0	E9PHK0_HUMAN	CLEC3B	Tetranectin	1.0000	7.5	1	2	3
Q$224-2	P05452	TETN_HUMAN	CLEC3B	Tetranectin	1.0000	5.94	1	2	3
						TFHEASEDCISR	2	0.9993	2
						TFHEASEDCISR	3	0.956	1
Q$225-1	E9PHT9	E9PHT9_HUMAN	ANXA5	Annexin	0.9765	9.82	1	1	1
Q$225-2	P08758	ANXA5_HUMAN	ANXA5	Annexin A5	0.9765	5	1	1	1
						GLGTDEESILTLLTSR	2	0.9765	1
Q$226-1	E9PLE5	E9PLE5_HUMAN	RIC8A	Synembryn-A	1.0000	36.54	1	1	3
Q$226-2	E9PI04	E9PI04_HUMAN	RIC8A	Synembryn-A (Fragment)	1.0000	12.84	1	1	3
						AVAEAVETGEEDVIMEALR	2	0.9997	3
Q$227-1	E9PIF4	E9PIF4_HUMAN	NEU1	Sialidase-1	0.9998	7.12	2	2	2
Q$227-2	Q99519	NEUR1_HUMAN	NEU1	Sialidase-1	0.9998	4.58	2	2	2
						GTLLAFAEAR	2	0.9936	1
						IPLITATPR	2	0.9684	1
Q$228-1	E9PPV6	E9PPV6_HUMAN	SERPINH1	Serpin H1	0.9929	8.95	2	2	2
Q$228-2	P50454	SERPH_HUMAN	SERPINH1	Serpin H1	0.9929	6.94	2	2	2
Q$228-3	E9PIG2	E9PIG2_HUMAN	SERPINH1	Serpin H1 (Fragment)	0.9929	17.58	2	2	2
Q$228-4	E9PJH8	E9PJH8_HUMAN	SERPINH1	Serpin H1 (Fragment)	0.9929	19.33	2	2	2
Q$228-5	E9PK86	E9PK86_HUMAN	SERPINH1	Serpin H1 (Fragment)	0.9929	11.74	2	2	2
Q$228-6	E9PMI5	E9PMI5_HUMAN	SERPINH1	Serpin H1 (Fragment)	0.9929	11.65	2	2	2
Q$228-7	E9PNX1	E9PNX1_HUMAN	SERPINH1	Serpin H1 (Fragment)	0.9929	14.29	2	2	2
Q$228-8	E9PR70	E9PR70_HUMAN	SERPINH1	Serpin H1 (Fragment)	0.9929	9.83	2	2	2
Q$228-9	E9PRS3	E9PRS3_HUMAN	SERPINH1	Serpin H1 (Fragment)	0.9929	17.47	2	2	2
						LYGPSSVSFADDFVR	2	0.9127	1
						SAGLAFSLYQAMAK	2	0.9188	1
Q$229-1	E9PIT3	E9PIT3_HUMAN	F2	Thrombin light chain	1.0000	34.65	29	32	161
						ACLEGNCAEGLGTNYR	2	0.9983	3
						ANTFLEEVR	2	0.9055	1
						CYTTDPTVR	2	0.9121	1
						DKLAACLEGNCAEGLGTNYR	3	0.9995	16
						ELLESYIDGR	2	0.9997	8
						ETWTANVGK	2	0.995	1
						HQDFNSAVQLVENFCR	3	0.9997	4
						KSPQELLCGASLISDR	2	0.9997	6
						KSPQELLCGASLISDR	3	0.9997	5
						LAACLEGNCAEGLGTNYR	2	0.9997	12
						LAVTTHGLPCLAWASAQAK	2	0.9997	4
						LAVTTHGLPCLAWASAQAK	3	0.9997	7
						LEGNCAEGLGTNYR	2	0.9988	4
						NPDSSTTGPWCYTTD	2	0.9983	3
						NPDSSTTGPWCYTTDPTVR	3	0.9997	5
						RQECSIPVCGQD	2	0.9889	2
						RQECSIPVCGQDQ	2	0.9989	7
						RQECSIPVCGQDQVT	2	0.9988	5
						RQECSIPVCGQDQVTV	2	0.9981	5
						RQECSIPVCGQDQVTVA	2	0.998	6
						RQECSIPVCGQDQVTVAMTPR	3	0.9997	16
						SGIECQLWR	2	0.9997	9
						SPQELLCGASLISDR	2	0.9998	7
						SSTTGPWCYTTDPTVR	2	0.9989	3
						STTGPWCYTTDPTVR	2	0.9986	3
						STTHPGADLQENFCR	2	0.9989	5
						TFGSGEADCGLRPL	2	0.9925	3
						TFGSGEADCGLRPLF	2	0.9865	4
						VTGWGNLK	2	0.9932	2
						WVLTAAH	2	0.8499	1
						YGFYTHVFR	2	0.9995	2
						YGFYTHVFR	3	0.9991	1
Q$230-1	E9PJF4	E9PJF4_HUMAN	CLNS1A	Methylosome subunit pICln	1.0000	21.23	2	2	3
Q$230-2	E9PMI6	E9PMI6_HUMAN	CLNS1A	Methylosome subunit pICln	1.0000	22.75	2	2	3
Q$230-3	J3KN38	J3KN38_HUMAN	CLNS1A	Methylosome subunit pICln	1.0000	18.72	2	2	3
Q$230-4	P54105	ICLN_HUMAN	CLNS1A	Methylosome subunit pICln	1.0000	16.03	2	2	3
						GLGTGTLYIAESR	2	0.9994	1
						LSWLDGSGLGFSLEYPTISLHALSR	3	0.9524	2
Q$231-1	E9PJH4	E9PJH4_HUMAN	RPS3	40S ribosomal protein S3	1.0000	26.96	3	3	8
Q$231-2	E9PL09	E9PL09_HUMAN	RPS3	40S ribosomal protein S3	1.0000	13.42	3	3	8
Q$231-3	E9PPU1	E9PPU1_HUMAN	RPS3	40S ribosomal protein S3	1.0000	19.62	3	3	8
Q$231-4	E9PQ96	E9PQ96_HUMAN	RPS3	40S ribosomal protein S3	1.0000	34.07	3	3	8
Q$231-5	F2Z2S8	F2Z2S8_HUMAN	RPS3	40S ribosomal protein S3	1.0000	26.5	3	3	8
Q$231-6	P23396	RS3_HUMAN	RPS3	40S ribosomal protein S3	1.0000	12.76	3	3	8
Q$231-7	E9PK82	E9PK82_HUMAN	RPS3	40S ribosomal protein S3 (Fragment)	1.0000	24.22	3	3	8
Q$231-8	H0YCJ7	H0YCJ7_HUMAN	RPS3	40S ribosomal protein S3 (Fragment)	1.0000	22.96	3	3	8
Q$231-9	H0YEU2	H0YEU2_HUMAN	RPS3	40S ribosomal protein S3 (Fragment)	1.0000	18.13	3	3	8
Q$231-10	H0YF32	H0YF32_HUMAN	RPS3	40S ribosomal protein S3 (Fragment)	1.0000	25.2	3	3	8
Q$231-11	P23396-2	RS3_HUMAN	RPS3	Isoform 2 of 40S ribosomal protein S3	1.0000	11.97	3	3	8
						AELNEFLTR	2	0.9991	2
						ELAEDGYSGVEVR	2	0.9997	5
						TEIIILATR	2	0.9984	1

Q$232-1	F8VRJ2	F8VRJ2_HUMAN	NAP1L1	Nucleosome assembly protein 1-like 1 (Fragment)	1.0000	8.77	1	1	2
Q$232-2	F8VVB5	F8VVB5_HUMAN	NAP1L1	Nucleosome assembly protein 1-like 1 (Fragment)	1.0000	6.21	1	1	2
Q$232-3	E9PJJ2	E9PJJ2_HUMAN	NAP1L4	Nucleosome assembly protein 1-like 4 (Fragment)	1.0000	7.81	1	1	2
Q$232-4	E9PKT8	E9PKT8_HUMAN	NAP1L4	Nucleosome assembly protein 1-like 4 (Fragment)	1.0000	5.92	1	1	2
Q$232-5	E9PNJ7	E9PNJ7_HUMAN	NAP1L4	Nucleosome assembly protein 1-like 4 (Fragment)	1.0000	7.14	1	1	2
Q$232-6	E9PNW0	E9PNW0_HUMAN	NAP1L4	Nucleosome assembly protein 1-like 4 (Fragment)	1.0000	9.35	1	1	2
Q$232-7	E9PS34	E9PS34_HUMAN	NAP1L4	Nucleosome assembly protein 1-like 4 (Fragment)	1.0000	7.52	1	1	2
						FYEEVHDLER	2	0.9994	2
Q$233-1	E9PRA8	E9PRA8_HUMAN	CRYAB	Alpha-crystallin B chain	1.0000	18.71	3	3	3
Q$233-2	P02511	CRYAB_HUMAN	CRYAB	Alpha-crystallin B chain	1.0000	16.57	3	3	3
Q$233-3	E9PJL7	E9PJL7_HUMAN	CRYAB	Alpha-crystallin B chain (Fragment)	1.0000	22.48	3	3	3
Q$233-4	E9PR44	E9PR44_HUMAN	CRYAB	Alpha-crystallin B chain (Fragment)	1.0000	16.67	3	3	3
						APSWFDTGLSEMR	2	0.9995	1
						FSVNLDVK	2	0.9658	1
						HFSPEELK	2	0.9879	1
Q$234-1	E9PRY8	E9PRY8_HUMAN	EEF1D	Elongation factor 1-delta	1.0000	11.33	6	7	13
Q$234-2	P29692	EF1D_HUMAN	EEF1D	Elongation factor 1-delta	1.0000	28.11	6	7	13
Q$234-3	E9PK01	E9PK01_HUMAN	EEF1D	Elongation factor 1-delta (Fragment)	1.0000	30.27	6	7	13
Q$234-4	P29692-2	EF1D_HUMAN	EEF1D	Isoform 2 of Elongation factor 1-delta	1.0000	12.21	6	7	13
						FYEQMNGPVAGASR	2	0.9997	2
						GVVQELQQAISK	2	0.9996	2
						GVVQELQQAISKLEAR	2	0.9948	1
						GVVQELQQAISKLEAR	3	0.9994	2
						IASLEVENQSLR	2	0.9997	2
						SIQLDGLVWGASK	2	0.9997	2
						SLAGSSGPGASSGTSGDHGELVVR	3	0.9804	2
Q$235-1	G3V1A4	G3V1A4_HUMAN	CFL1	Cofilin 1 (Non-muscle), isoform CRA_a	1.0000	56.38	6	7	14
Q$235-2	E9PK25	E9PK25_HUMAN	CFL1	Cofilin-1	1.0000	41.18	6	7	14
Q$235-3	P23528	COF1_HUMAN	CFL1	Cofilin-1	1.0000	50.6	6	7	14
						AVLFCLSEDKK	2	0.9642	1
						ESKKEDLVFIFWAPESAPLK	3	0.9602	2
						KEDLVFIFWAPESAPLK	2	0.9894	1
						KEDLVFIFWAPESAPLK	3	0.9983	2
						LGGSAVISLEGKPL	2	0.9979	2
						NIILEEGKEILVGDVGQTVDDPYATFVK	3	0.9989	3
						YALYDATYETK	2	0.9995	3
Q$236-1	P06737	PYGL_HUMAN	PYGL	Glycogen phosphorylase, liver form	0.9994	1.65	1	1	2
Q$236-2	P06737-2	PYGL_HUMAN	PYGL	Isoform 2 of Glycogen phosphorylase, liver form	0.9994	1.72	1	1	2
Q$236-3	E9PK47	E9PK47_HUMAN	PYGL	Phosphorylase	0.9994	1.71	1	1	2
						DFNVGDYIQAVLDR	2	0.9893	2
Q$237-1	E9PKD5	E9PKD5_HUMAN	PSMC3	26S protease regulatory subunit 6A (Fragment)	1.0000	5.47	2	2	3
						VDILDPALLR	2	0.999	1
						VIAATNRVDILDPALLR	3	0.9925	2
Q$238-1	P54652	HSP72_HUMAN	HSPA2	Heat shock-related 70 kDa protein 2	1.0000	12.21	6	6	12
Q$238-2	E9PKE3	E9PKE3_HUMAN	HSPA8	Heat shock cognate 71 kDa protein	1.0000	12.44	6	6	12
Q$238-3	P11142	HSP7C_HUMAN	HSPA8	Heat shock cognate 71 kDa protein	1.0000	12.07	6	6	12
Q$238-4	P11142-2	HSP7C_HUMAN	HSPA8	Isoform 2 of Heat shock cognate 71 kDa protein	1.0000	15.82	6	6	12
						ARFEELNADLFR	3	0.9959	2
						IINEPTAAAIAYGLDK	2	0.9947	2
						STAGDTHLGGEDFDNR	2	0.9994	2
						TTPSYVAFTDTER	2	0.9997	3
						VEIIANDQGNR	2	0.9988	2
						VQVEYKGETK	2	0.9793	1
Q$239-1	E9PP60	E9PP60_HUMAN	TSTA3	GDP-L-fucose synthase	0.9500	10.43	1	1	2
Q$239-2	Q13630	FCL_HUMAN	TSTA3	GDP-L-fucose synthase	0.9500	3.74	1	1	2
Q$239-3	E9PKL9	E9PKL9_HUMAN	TSTA3	GDP-L-fucose synthase (Fragment)	0.9500	4.48	1	1	2
Q$239-4	E9PLH9	E9PLH9_HUMAN	TSTA3	GDP-L-fucose synthase (Fragment)	0.9500	10.17	1	1	2
Q$239-5	E9PP14	E9PP14_HUMAN	TSTA3	GDP-L-fucose synthase (Fragment)	0.9500	6.06	1	1	2
						ILVTGGSGLVGK	2	0.95	2
Q$240-1	E9PKN4	E9PKN4_HUMAN	CSDE1	Cold shock domain-containing protein E1 (Fragment)	1.0000	28.26	1	1	2
Q$240-2	E9PLD4	E9PLD4_HUMAN	CSDE1	Cold shock domain-containing protein E1 (Fragment)	1.0000	13.83	1	1	2
						LLTSYGFIQCSER	2	0.9984	2
Q$241-1	Q14697-2	GANAB_HUMAN	GANAB	Isoform 2 of Neutral alpha-glucosidase AB	1.0000	12.94	7	10	18
Q$241-2	E9PKU7	E9PKU7_HUMAN	GANAB	Neutral alpha-glucosidase AB	1.0000	14.67	7	10	18
Q$241-3	F5H6X6	F5H6X6_HUMAN	GANAB	Neutral alpha-glucosidase AB	1.0000	14.76	7	10	18
Q$241-4	Q14697	GANAB_HUMAN	GANAB	Neutral alpha-glucosidase AB	1.0000	13.24	7	10	18
						DLGIFWLNAAETWVDISSNTAGK	2	0.9793	1
						FSFSGNTLVSSSADPEGHFETPIWIER	3	0.9996	3
						LKVTEGGEPYR	2	0.9496	2
						LKVTEGGEPYR	3	0.8765	1
						LSFQHDPETSVLVLR	2	0.9991	2
						LSFQHDPETSVLVLR	3	0.999	2
						LYNLDVFQYELYNPMALYGSVPVLLAHNPHR	4	0.9968	1
						RFSFSGNTLVSSSADPEGHFETPIWIER	3	0.9046	1
						VVIIGAGKPAAVVLQTK	2	0.9995	2
						VVIIGAGKPAAVVLQTK	3	0.9996	3
Q$242-1	Q5TB19	Q5TB19_HUMAN	ANP32E	Acidic (Leucine-rich) nuclear phosphoprotein 32 family, mem	1.0000	12.75	1	1	2
Q$242-2	E9PLC4	E9PLC4_HUMAN	ANP32E	Acidic leucine-rich nuclear phosphoprotein 32 family membe	1.0000	11.52	1	1	2
						KLELSDNIISGGLEVLAEK	3	0.9965	2
Q$243-1	E9PLJ3	E9PLJ3_HUMAN	CFL1	Cofilin-1 (Fragment)	1.0000	13.92	1	1	3
						YALYDATYETK	2	0.9995	3
Q$244-1	E9PLT0	E9PLT0_HUMAN	CSDE1	Cold shock domain-containing protein E1	1.0000	4.04	2	2	3
						ATNIEVLSNTFQFTNEAR	2	0.9994	2
						LLGYVATLK	2	0.9753	1
Q$245-1	E9PM54	E9PM54_HUMAN	ILK	Integrin-linked protein kinase	0.9196	4.78	1	1	1
Q$245-2	E9PNR4	E9PNR4_HUMAN	ILK	Integrin-linked protein kinase	0.9196	4.57	1	1	1
Q$245-3	Q13418	ILK_HUMAN	ILK	Integrin-linked protein kinase	0.9196	2.21	1	1	1
Q$245-4	Q13418-2	ILK_HUMAN	ILK	Isoform 2 of Integrin-linked protein kinase	0.9196	2.56	1	1	1
						SAVVEMLIMR	2	0.9196	1
Q$246-1	E9PM69	E9PM69_HUMAN	PSMC3	26S protease regulatory subunit 6A	1.0000	9.32	3	3	4
Q$246-2	P17980	PRS6A_HUMAN	PSMC3	26S protease regulatory subunit 6A	1.0000	8.43	3	3	4
Q$246-3	R4GNH3	R4GNH3_HUMAN	PSMC3	26S protease regulatory subunit 6A	1.0000	8.75	3	3	4
						GATELTHEDYMEGILEVQAK	3	0.9627	1
						VDILDPALLR	2	0.999	1
						VIAATNRVDILDPALLR	3	0.9925	2
Q$247-1	E9PPT0	E9PPT0_HUMAN	RPS2	40S ribosomal protein S2	0.9856	6.6	1	1	2
Q$247-2	E9PQD7	E9PQD7_HUMAN	RPS2	40S ribosomal protein S2	0.9856	5.53	1	1	2
Q$247-3	P15880	RS2_HUMAN	RPS2	40S ribosomal protein S2	0.9856	4.44	1	1	2
Q$247-4	E9PMM9	E9PMM9_HUMAN	RPS2	40S ribosomal protein S2 (Fragment)	0.9856	5.96	1	1	2
Q$247-5	H0YE27	H0YE27_HUMAN	RPS2	40S ribosomal protein S2 (Fragment)	0.9856	20.63	1	1	2
Q$247-6	H0YEN5	H0YEN5_HUMAN	RPS2	40S ribosomal protein S2 (Fragment)	0.9856	6.67	1	1	2
Q$247-7	H3BNG3	H3BNG3_HUMAN	RPS2	40S ribosomal protein S2 (Fragment)	0.9856	29.55	1	1	2
						SLEEIYLFSLPIK	2	0.9856	2
Q$248-1	E9PN89	E9PN89_HUMAN	HSPA8	Heat shock cognate 71 kDa protein (Fragment)	1.0000	5.13	1	1	2
						IINEPTAAAIAYGLDK	2	0.9947	2
Q$249-1	E9PNE6	E9PNE6_HUMAN	HSPA8	Heat shock cognate 71 kDa protein	1.0000	9.4	3	3	7
						ARFEELNADLFR	3	0.9959	2
						GPAVGIDLGTTYSCVGVFQHGK	3	0.996	2
						TTPSYVAFTDTER	2	0.9997	3
Q$250-1	E9PNW4	E9PNW4_HUMAN	CD59	CD59 glycoprotein	1.0000	34.26	4	5	8
Q$250-2	E9PR17	E9PR17_HUMAN	CD59	CD59 glycoprotein	1.0000	28.46	4	5	8
Q$250-3	P13987	CD59_HUMAN	CD59	CD59 glycoprotein	1.0000	28.91	4	5	8
						AGLQVYNK	2	0.9997	1
						CWKFEHCNFNDVTTR	3	0.9084	1
						FEHCNFNDVTTR	2	0.9995	2
						FEHCNFNDVTTR	3	0.9941	1
						LQCYNCPNPTADCK	2	0.9983	3
Q$251-1	E9PP50	E9PP50_HUMAN	CFL1	Cofilin-1 (Fragment)	1.0000	17.61	2	2	5
Q$251-2	E9PQB7	E9PQB7_HUMAN	CFL1	Cofilin-1 (Fragment)	1.0000	22.95	2	2	5
Q$251-3	Q9Y281	COF2_HUMAN	CFL2	Cofilin-2	1.0000	16.87	2	2	5
Q$251-4	Q9Y281-3	COF2_HUMAN	CFL2	Isoform 3 of Cofilin-2	1.0000	18.79	2	2	5
						KEDLVFIFWAPESAPLK	3	0.9983	2
						YALYDATYETK	2	0.9995	3

Q$252-1	E9PQH6	E9PQH6_HUMAN	RHOC	Rho-related GTP-binding protein RhoC (Fragment)	1.0000	28.4	5	5	9
						HFCPNVPIILVGNKK	3	0.9995	2
						ISAFGYLECSAK	2	0.9886	1
						KLVIVGDGACGK	2	0.9964	1
						LVIVGDGACGK	2	0.984	2
						TCLLIVFSK	2	0.9996	3
Q$253-1	E9PRM1	E9PRM1_HUMAN	CAPN1	Calpain-1 catalytic subunit	1.0000	14.43	2	2	4
						LVFVHSAEGNEFWSALLEK	3	0.9978	3
						YLGQDYEQLR	2	0.9992	1
Q$254-1	Q9NPQ8-3	RIC8A_HUMAN	RIC8A	Isoform 3 of Synembryn-A	1.0000	6.89	2	3	7
Q$254-2	Q9NPQ8-4	RIC8A_HUMAN	RIC8A	Isoform 4 of Synembryn-A	1.0000	6.98	2	3	7
Q$254-3	Q9NPQ8	RIC8A_HUMAN	RIC8A	Synembryn-A	1.0000	6.97	2	3	7
Q$254-4	E9PSI0	E9PSI0_HUMAN	RIC8A	Synembryn-A (Fragment)	1.0000	20.22	2	3	7
						AVAEAVETGEEDVIMEALR	2	0.9997	3
						AVAEAVETGEEDVIMEALR	3	0.9397	1
						LAELLVSVLEQGLPPSHR	3	0.9982	3
Q$255-1	Q7Z406-2	MYH14_HUMAN	MYH14	Isoform 2 of Myosin-14	1.0000	16.31	26	31	51
Q$255-2	Q7Z406-6	MYH14_HUMAN	MYH14	Isoform 6 of Myosin-14	1.0000	16.58	26	31	51
Q$255-3	F2Z2U8	F2Z2U8_HUMAN	MYH14	Myosin-14	1.0000	16.37	26	31	51
Q$255-4	G8JLL9	G8JLL9_HUMAN	MYH14	Myosin-14	1.0000	16.31	26	31	51
Q$255-5	Q7Z406	MYH14_HUMAN	MYH14	Myosin-14	1.0000	16.64	26	31	51
						AEAELCAEAEETR	2	0.9995	2
						AELSSLQTAR	2	0.9766	1
						AMEAEAAGLREQLEEEAAAR	3	0.936	1
						AQAELENVSGALNEAESK	2	0.9997	3
						AQVTELEDELTAAEDAK	2	0.9997	3
						DLGEELEALRGELEDTLDSTNAQQELR	3	0.9986	3
						EAQAALAEAQEDLESER	2	0.9997	1
						ELFQETLESLR	2	0.9705	1
						ELQTAQAQLSEWR	2	0.9996	3
						ELSSTEAQLHDAQELLQEETR	3	0.9977	2
						FDQLLAEEK	2	0.9825	1
						HGQALGELAEQLEQAR	3	0.9993	3
						IHEAAVQELR	2	0.9994	1
						IHEAAVQELR	3	0.9921	1
						ILFQEFR	2	0.9921	1
						KEEELQAALAR	2	0.9991	2
						KEEELQAALAR	3	0.996	1
						KFDQLLAEEK	2	0.9778	1
						KQELELVVSELEAR	2	0.9993	2
						KQELELVVSELEAR	3	0.9995	2
						LAEFSSQAAEEEEK	2	0.9634	1
						LAEFSSQAAEEEEKVK	2	0.9974	1
						LALEAEVSELR	2	0.9997	1
						LALEAEVSELRAELSSLQTAR	3	0.9994	3
						LAQAEEQLEQETR	2	0.9996	2
						LQQHIQELEAHLEAEEGAR	3	0.998	1
						RLELQLQEVQGR	2	0.9963	1
						RLELQLQEVQGR	3	0.9888	2
						RQEEEAGALEAGEEAR	2	0.9992	1
						RQEEEAGALEAGEEAR	3	0.9185	1
						VAEQAANDLR	2	0.9997	2
Q$256-1	F2Z2Y4	F2Z2Y4_HUMAN	PDXK	Pyridoxal kinase	1.0000	12.13	2	2	3
Q$256-2	O00764	PDXK_HUMAN	PDXK	Pyridoxal kinase	1.0000	10.58	2	2	3
						DKSFLAMVVDIVQELK	3	0.979	2
						GQVLNSDELQELYEGLR	2	0.9993	1
Q$257-1	Q14914-2	PTGR1_HUMAN	PTGR1	Isoform 2 of Prostaglandin reductase 1	0.9947	6.64	2	2	2
Q$257-2	Q5JVP3	Q5JVP3_HUMAN	PTGR1	Leukotriene B4 12-hydroxydehydrogenase, isoform CRA_d	0.9947	11.3	2	2	2
Q$257-3	Q14914	PTGR1_HUMAN	PTGR1	Prostaglandin reductase 1	0.9947	6.08	2	2	2
Q$257-4	F2Z3J9	F2Z3J9_HUMAN	PTGR1	Prostaglandin reductase 1 (Fragment)	0.9947	16.95	2	2	2
						IAICGAISTYNR	2	0.9518	1
						MEAFVVYR	2	0.8996	1
Q$258-1	F2Z3K5	F2Z3K5_HUMAN	RPN2	Dolichyl-diphosphooligosaccharide--protein glycosyltransfera	1.0000	7.41	1	1	3
Q$258-2	Q5JYR4	Q5JYR4_HUMAN	RPN2	Dolichyl-diphosphooligosaccharide--protein glycosyltransfera	1.0000	7.23	1	1	3
Q$258-3	Q5JYR7	Q5JYR7_HUMAN	RPN2	Dolichyl-diphosphooligosaccharide--protein glycosyltransfera	1.0000	3.49	1	1	3
						SIVEEIEDLVAR	2	0.9997	3
Q$259-1	F5GWF6	F5GWF6_HUMAN	CCT2	T-complex protein 1 subunit beta	1.0000	5.09	2	2	6
Q$259-2	F8VQ14	F8VQ14_HUMAN	CCT2	T-complex protein 1 subunit beta	1.0000	6.49	2	2	6
						GATQQILDEAER	2	0.9992	3
						LTSFIGAIAIGDLVK	2	0.9997	3
Q$260-1	F5GWY2	F5GWY2_HUMAN	ATIC	Phosphoribosylaminoimidazolecarboxamide formyltransferas	1.0000	15.76	6	7	22
						DLIVATIAVK	2	0.9994	2
						EVSDGIIAPGYEEEALTILSK	2	0.9994	6
						EVSDGIIAPGYEEEALTILSK	3	0.9968	2
						LDFNLIR	2	0.9996	2
						TLFGLHLSQK	2	0.9671	1
						TVASPGVTVEEAVEQIDIGGVTLLR	3	0.9996	6
						VVACNLYPFVK	2	0.9995	3
Q$261-1	F5GX14	F5GX14_HUMAN	MLEC	Malectin	0.9999	16.13	2	2	4
Q$261-2	Q14165	MLEC_HUMAN	MLEC	Malectin	0.9999	11.99	2	2	4
Q$261-3	F5H1S8	F5H1S8_HUMAN	MLEC	Malectin (Fragment)	0.9999	23.97	2	2	4
						FAEVYFAQSQQK	2	0.9992	3
						YNEETFGYEVPIKEEGDYVLVLK	3	0.8515	1
Q$262-1	F5H5J4	F5H5J4_HUMAN	LDHA	L-lactate dehydrogenase A chain	1.0000	16.85	1	1	3
Q$262-2	F5H6W8	F5H6W8_HUMAN	LDHA	L-lactate dehydrogenase A chain	1.0000	15.96	1	1	3
Q$262-3	F5GXH2	F5GXH2_HUMAN	LDHA	L-lactate dehydrogenase A chain (Fragment)	1.0000	13.16	1	1	3
Q$262-4	F5GXY2	F5GXY2_HUMAN	LDHA	L-lactate dehydrogenase A chain (Fragment)	1.0000	9.62	1	1	3
Q$262-5	F5GYU2	F5GYU2_HUMAN	LDHA	L-lactate dehydrogenase A chain (Fragment)	1.0000	10.42	1	1	3
Q$262-6	F5GZQ4	F5GZQ4_HUMAN	LDHA	L-lactate dehydrogenase A chain (Fragment)	1.0000	20.83	1	1	3
						DLADELALVDVIEDK	2	0.9992	3
Q$263-1	F5GXR3	F5GXR3_HUMAN	PTMS	Parathymosin	0.9544	10.58	1	1	1
Q$263-2	P20962	PTMS_HUMAN	PTMS	Parathymosin	0.9544	10.78	1	1	1
Q$263-3	F5H7R9	F5H7R9_HUMAN	PTMS	Parathymosin (Fragment)	0.9544	19.3	1	1	1
						SVEAAAELSAK	2	0.9544	1
Q$264-1	F5GXS2	F5GXS2_HUMAN	ACTN4	Alpha-actinin-4	1.0000	7.68	3	3	7
Q$264-2	H7C144	H7C144_HUMAN	ACTN4	Alpha-actinin-4 (Fragment)	1.0000	11.7	3	3	7
Q$264-3	O43707-3	ACTN4_HUMAN	ACTN4	Isoform 3 of Alpha-actinin-4	1.0000	7.68	3	3	7
						ETTDTDTADQVIASFK	2	0.9991	2
						TINEVENQILTR	2	0.999	2
						VGWEQLLTTIAR	2	0.9996	3
Q$265-1	F5GY79	F5GY79_HUMAN	PTPN6	Tyrosine-protein phosphatase non-receptor type 6 (Fragmen	0.9999	6.25	1	1	1
Q$265-2	F5H0N8	F5H0N8_HUMAN	PTPN6	Tyrosine-protein phosphatase non-receptor type 6 (Fragmen	0.9999	5.45	1	1	1
Q$265-3	F5H1Z8	F5H1Z8_HUMAN	PTPN6	Tyrosine-protein phosphatase non-receptor type 6 (Fragmen	0.9999	6	1	1	1
Q$265-4	F5H5H9	F5H5H9_HUMAN	PTPN6	Tyrosine-protein phosphatase non-receptor type 6 (Fragmen	0.9999	6.92	1	1	1
						VGDQVTHIR	2	0.9916	1
Q$266-1	F5GY80	F5GY80_HUMAN	C8B	Complement component C8 beta chain	1.0000	22.68	12	14	32
Q$266-2	F5H7G1	F5H7G1_HUMAN	C8B	Complement component C8 beta chain	1.0000	22.26	12	14	32
Q$266-3	P07358	CO8B_HUMAN	C8B	Complement component C8 beta chain	1.0000	20.3	12	14	32
						DTMVEDLVVLVR	2	0.9995	3
						ELVTATDFAYSSTVR	2	0.995	3
						GDYTLNNVHACAK	2	0.9997	3
						GGASEHITTLAYQELPTADLMQEWGDAVQYNPAIIK	3	0.9906	1
						GILNEIKDR	2	0.943	1
						LPLEYSYGEYR	2	0.9994	5
						RLPLEYSYGEYR	2	0.979	1
						SDLEVAHYK	2	0.9943	2
						SVFLHAR	2	0.9625	1
						TATDFAYSSTVR	2	0.8146	1
						VEPLYELVTATDFAYSSTVR	2	0.9997	2
						VEPLYELVTATDFAYSSTVR	3	0.994	2
						VKVEPLYELVTATDFAYSSTVR	2	0.9985	1
						VKVEPLYELVTATDFAYSSTVR	3	0.9987	6
Q$267-1	F5GYI5	F5GYI5_HUMAN	KRT85	Keratin, type II cuticular Hb5	1.0000	7.8	2	2	4

Q$267-2	P78386	KRT85_HUMAN	KRT85	Keratin, type II cuticular Hb5	1.0000	4.54	2	2	4
						DLNMDCIIAEIK	2	0.9989	2
						LGLDIEIATYR	2	0.9976	2
Q$268-1	F5GYJ8	F5GYJ8_HUMAN	OTUB1	Ubiquitin thioesterase OTUB1	1.0000	25.52	7	7	9
Q$268-2	F5GYN4	F5GYN4_HUMAN	OTUB1	Ubiquitin thioesterase OTUB1	1.0000	30.29	7	7	9
Q$268-3	J3KR44	J3KR44_HUMAN	OTUB1	Ubiquitin thioesterase OTUB1	1.0000	23.7	7	7	9
Q$268-4	Q96FW1	OTUB1_HUMAN	OTUB1	Ubiquitin thioesterase OTUB1	1.0000	26.94	7	7	9
						AFGFSHLEALLDDSK	3	0.9681	1
						AFGFSHLEALLDDSKELQR	3	0.9746	2
						EYAEDDNIYQQK	2	0.9989	1
						FFEHFIEGGR	3	0.9858	2
						IQQEIAVQNPLVSER	2	0.9997	1
						LELSVLYK	2	0.9969	1
						LLTSGYLQR	2	0.9615	1
Q$269-1	F5GYR8	F5GYR8_HUMAN	USO1	General vesicular transport factor p115	1.0000	2.78	2	2	4
Q$269-2	F5H4X1	F5H4X1_HUMAN	USO1	General vesicular transport factor p115	1.0000	2.85	2	2	4
Q$269-3	O60763	USO1_HUMAN	USO1	General vesicular transport factor p115	1.0000	2.81	2	2	4
Q$269-4	H0Y7C0	H0Y7C0_HUMAN	USO1	General vesicular transport factor p115 (Fragment)	1.0000	4.23	2	2	4
Q$269-5	O60763-2	USO1_HUMAN	USO1	Isoform 2 of General vesicular transport factor p115	1.0000	2.77	2	2	4
						SQLNSQSVEITK	2	0.9995	2
						SVEVQGETETIIATK	2	0.9997	2
Q$270-1	M0R1V7	M0R1V7_HUMAN	UBA52	Ubiquitin-60S ribosomal protein L40 (Fragment)	1.0000	25.4	1	1	3
Q$270-2	J3QSA3	J3QSA3_HUMAN	UBB	Ubiquitin (Fragment)	1.0000	37.21	1	1	3
Q$270-3	F5GZ39	F5GZ39_HUMAN	UBC	Polyubiquitin-C (Fragment)	1.0000	26.23	1	1	3
						TITLEVEPSDTIENVK	2	0.9996	3
Q$271-1	Q9Y493-2	ZAN_HUMAN	ZAN	Isoform 1 of Zonadhesin	0.9719	0.33	1	1	1
Q$271-2	Q9Y493-3	ZAN_HUMAN	ZAN	Isoform 2 of Zonadhesin	0.9719	0.33	1	1	1
Q$271-3	Q9Y493-4	ZAN_HUMAN	ZAN	Isoform 4 of Zonadhesin	0.9719	0.34	1	1	1
Q$271-4	Q9Y493-5	ZAN_HUMAN	ZAN	Isoform 5 of Zonadhesin	0.9719	0.35	1	1	1
Q$271-5	Q9Y493-6	ZAN_HUMAN	ZAN	Isoform 6 of Zonadhesin	0.9719	0.33	1	1	1
Q$271-6	Q9Y493-7	ZAN_HUMAN	ZAN	Isoform 7 of Zonadhesin	0.9719	0.31	1	1	1
Q$271-7	F5GZI5	F5GZI5_HUMAN	ZAN	Zonadhesin	0.9719	0.33	1	1	1
Q$271-8	F5H0T8	F5H0T8_HUMAN	ZAN	Zonadhesin	0.9719	0.33	1	1	1
Q$271-9	F5H4B5	F5H4B5_HUMAN	ZAN	Zonadhesin	0.9719	0.34	1	1	1
Q$271-10	Q9Y493	ZAN_HUMAN	ZAN	Zonadhesin	0.9719	0.32	1	1	1
						GVFLGASGR	2	0.9719	1
Q$272-1	P63096-2	GNAI1_HUMAN	GNAI1	Isoform 2 of Guanine nucleotide-binding protein G(i) subunit	1.0000	9.27	2	2	3
Q$272-2	F5GZL8	F5GZL8_HUMAN	GNAI2	Guanine nucleotide-binding protein G(i) subunit alpha-2	1.0000	10.22	2	2	3
						EIYTHFTCATDTK	2	0.9958	2
						NVQFVFDAVTDVIIK	2	0.9937	1
Q$273-1	F5GZQ3	F5GZQ3_HUMAN	HADHB	3-ketoacyl-CoA thiolase	0.9998	5.45	2	2	2
Q$273-2	P55084-2	ECHB_HUMAN	HADHB	Isoform 2 of Trifunctional enzyme subunit beta, mitochondri	0.9998	5.53	2	2	2
Q$273-3	P55084	ECHB_HUMAN	HADHB	Trifunctional enzyme subunit beta, mitochondrial	0.9998	5.27	2	2	2
						EVVDYIIFGTVIQEVK	2	0.9932	1
						LEQDEYALR	2	0.9675	1
Q$274-1	F5H1V0	F5H1V0_HUMAN	C1R	Complement C1r subcomponent	1.0000	13.89	1	1	3
Q$274-2	F5GZR1	F5GZR1_HUMAN	C1R	Complement C1r subcomponent (Fragment)	1.0000	5.56	1	1	3
Q$274-3	F5H2D0	F5H2D0_HUMAN	C1R	Complement C1r subcomponent (Fragment)	1.0000	6.41	1	1	3
Q$274-4	F5H3N3	F5H3N3_HUMAN	C1R	Complement C1r subcomponent (Fragment)	1.0000	12.82	1	1	3
Q$274-5	F5H6Y3	F5H6Y3_HUMAN	C1R	Complement C1r subcomponent (Fragment)	1.0000	12.82	1	1	3
Q$274-6	F6T9X8	F6T9X8_HUMAN	C1R	Complement C1r subcomponent (Fragment)	1.0000	5.78	1	1	3
						LVFQQFDLEPSEGCFYDYVK	2	0.9993	3
Q$275-1	P52597	HNRPF_HUMAN	HNRNPF	Heterogeneous nuclear ribonucleoprotein F	1.0000	2.41	1	1	2
Q$275-2	F5GZT4	F5GZT4_HUMAN	HNRNPH1	Heterogeneous nuclear ribonucleoprotein H	1.0000	6.29	1	1	2
Q$275-3	H0YBD7	H0YBD7_HUMAN	HNRNPH1	Heterogeneous nuclear ribonucleoprotein H (Fragment)	1.0000	5.15	1	1	2
Q$275-4	H0YBG7	H0YBG7_HUMAN	HNRNPH1	Heterogeneous nuclear ribonucleoprotein H (Fragment)	1.0000	5.41	1	1	2
						VHIEIGPDGR	2	0.9986	2
Q$276-1	F5H0C7	F5H0C7_HUMAN	ARF3	ADP-ribosylation factor 3 (Fragment)	1.0000	34.31	2	2	4
						LGEIVTTIPTIGFNVETVEYK	2	0.9984	2
						NISFTVWDVGGQDK	2	0.9894	2
Q$277-1	Q86UX7	URP2_HUMAN	FERMT3	Fermitin family homolog 3	0.9985	2.4	1	1	2
Q$277-2	F5H1C6	F5H1C6_HUMAN	FERMT3	Fermitin family homolog 3 (Fragment)	0.9985	5.59	1	1	2
Q$277-3	Q86UX7-2	URP2_HUMAN	FERMT3	Isoform 2 of Fermitin family homolog 3	0.9985	2.41	1	1	2
						VFVGEEDPEAESVTLR	2	0.9978	2
Q$278-1	F5H283	F5H283_HUMAN	SPOCK2	Testican-2	1.0000	12.97	5	5	9
Q$278-2	Q92563	TICN2_HUMAN	SPOCK2	Testican-2	1.0000	12.97	5	5	9
						DSIGWMFSK	2	0.9982	2
						IQIQEAAK	2	0.9826	1
						LEQQACLSSK	2	0.9997	3
						SWEDNQQGDEALDTTK	3	0.9214	1
						VSTAEWCFCFWR	2	0.9997	2
Q$279-1	F5H2F4	F5H2F4_HUMAN	MTHFD1	C-1-tetrahydrofolate synthase, cytoplasmic	1.0000	2.25	2	2	2
Q$279-2	P11586	C1TC_HUMAN	MTHFD1	C-1-tetrahydrofolate synthase, cytoplasmic	1.0000	2.46	2	2	2
Q$279-3	V9GYY3	V9GYY3_HUMAN	MTHFD1	C-1-tetrahydrofolate synthase, cytoplasmic (Fragment)	1.0000	21.5	2	2	2
						GVPTGFILPIR	2	0.9748	1
						THLSLSHNPEQK	2	0.9897	1
Q$280-1	P52566	GDIR2_HUMAN	ARHGDIB	Rho GDP-dissociation inhibitor 2	0.9599	3.98	1	1	2
Q$280-2	F5H2R5	F5H2R5_HUMAN	ARHGDIB	Rho GDP-dissociation inhibitor 2 (Fragment)	0.9599	9.09	1	1	2
Q$280-3	F5H3P3	F5H3P3_HUMAN	ARHGDIB	Rho GDP-dissociation inhibitor 2 (Fragment)	0.9599	5.06	1	1	2
Q$280-4	F5H6Q0	F5H6Q0_HUMAN	ARHGDIB	Rho GDP-dissociation inhibitor 2 (Fragment)	0.9599	7.14	1	1	2
Q$280-5	H0YGX7	H0YGX7_HUMAN	ARHGDIB	Rho GDP-dissociation inhibitor 2 (Fragment)	0.9599	4.1	1	1	2
						APNVVVTR	2	0.9599	2
Q$281-1	F5H335	F5H335_HUMAN	EIF3A	Eukaryotic translation initiation factor 3 subunit A	1.0000	4.9	5	5	10
Q$281-2	Q14152	EIF3A_HUMAN	EIF3A	Eukaryotic translation initiation factor 3 subunit A	1.0000	4.78	5	5	10
						FNVLQYVVPEVK	2	0.9996	3
						LTSLVPFVDAFQLER	2	0.9979	2
						NQLTAMSSVLAK	2	0.9976	2
						TLSFGSDLNYATR	2	0.9995	2
						VLLATLSIPITPER	2	0.9902	1
Q$282-1	F5H3T8	F5H3T8_HUMAN	RARS	Arginine--tRNA ligase, cytoplasmic	1.0000	4.63	2	2	5
Q$282-2	P54136-2	SYRC_HUMAN	RARS	Isoform Monomeric of Arginine--tRNA ligase, cytoplasmic	1.0000	3.57	2	2	5
						LFEFAGYDVLR	2	0.9996	3
						STIIGESISR	2	0.9856	2
Q$283-1	F5H423	F5H423_HUMAN		Uncharacterized protein	1.0000	26.67	4	4	7
Q$283-2	P84077	ARF1_HUMAN	ARF1	ADP-ribosylation factor 1	1.0000	30.94	4	4	7
Q$283-3	P61204	ARF3_HUMAN	ARF3	ADP-ribosylation factor 3	1.0000	30.94	4	4	7
						DAVLLVFANK	2	0.9531	1
						ILMVGLDAAGK	2	0.9979	2
						LGEIVTTIPTIGFNVETVEYK	2	0.9984	2
						NISFTVWDVGGQDK	2	0.9894	2
Q$284-1	F5H4D6	F5H4D6_HUMAN	G3BP1	Ras GTPase-activating protein-binding protein 1	0.9064	4.58	1	1	1
Q$284-2	Q13283	G3BP1_HUMAN	G3BP1	Ras GTPase-activating protein-binding protein 1	0.9064	2.79	1	1	1
						DFFQSYGNVVELR	2	0.9064	1
Q$285-1	F5H4R9	F5H4R9_HUMAN	DNM2	Dynamin-2	1.0000	14.14	9	9	13
Q$285-2	P50570	DYN2_HUMAN	DNM2	Dynamin-2	1.0000	14.14	9	9	13
Q$285-3	P50570-2	DYN2_HUMAN	DNM2	Isoform 2 of Dynamin-2	1.0000	14.2	9	9	13
Q$285-4	P50570-3	DYN2_HUMAN	DNM2	Isoform 3 of Dynamin-2	1.0000	14.2	9	9	13
Q$285-5	P50570-4	DYN2_HUMAN	DNM2	Isoform 4 of Dynamin-2	1.0000	14.14	9	9	13
Q$285-6	P50570-5	DYN2_HUMAN	DNM2	Isoform 5 of Dynamin-2	1.0000	14.15	9	9	13
						DMILQFISR	2	0.9996	2
						EVDPQGLR	2	0.9416	1
						GISPVPINLR	2	0.9985	2
						HVFAIFNTEQR	2	0.9825	1
						LQDAFSSIGQSCHLDLPQIAVVGGQSAGK	3	0.9909	2
						NLVDSYVAIINK	2	0.9991	2
						SSVLENFVGR	2	0.9953	1
						VPVGDQPPDIEYQIK	2	0.9996	1
						VYSPHVLNLTLIDLPGITK	3	0.9971	1
Q$286-1	F5H5D3	F5H5D3_HUMAN	TUBA1C	Tubulin alpha-1C chain	1.0000	32.76	13	15	36
Q$286-2	Q9BQE3	TBA1C_HUMAN	TUBA1C	Tubulin alpha-1C chain	1.0000	37.86	13	15	36
						AVCMLSNTTAVAEAWAR	2	0.9997	3
						AVFVDLEPTVIDEVR	2	0.9997	4
						AVFVDLEPTVIDEVR	3	0.9996	2

						AYHEQLTVAEITNACFEPANQMVK	3	0.992	2
						DVNAAIATIK	2	0.999	2
						ECISIHVGQAGVQIGNACWEL	2	0.9109	1
						ECISIHVGQAGVQIGNACWELY	2	0.9069	1
						EIIDLVLDR	2	0.9997	4
						GHYTIGKEIIDLVLDR	3	0.9994	2
						KLADQCTGLQGF	2	0.9089	1
						LADQCTGLQGFLVFHSFGGGTGSGFTSLLMER	3	0.9909	1
						TIGGGDDSFNTFFSETGAGK	2	0.9997	8
						TIGGGDDSFNTFFSETGAGK	3	0.9851	1
						TIQFVDWCPTGFK	2	0.9995	3
						VDLEPTVIDEVR	2	0.8948	1
Q$287-1	F5H5Y3	F5H5Y3_HUMAN	SRP68	Signal recognition particle subunit SRP68	1.0000	6.27	2	2	3
						FETFCLDPSLVTK	2	0.9983	2
						SGGTEGLLAEKLEALITQTR	3	0.9664	1
Q$288-1	O00159-2	MYO1C_HUMAN	MYO1C	Isoform 2 of Unconventional myosin-Ic	1.0000	2.63	2	2	3
Q$288-2	O00159-3	MYO1C_HUMAN	MYO1C	Isoform 3 of Unconventional myosin-Ic	1.0000	2.59	2	2	3
Q$288-3	F5H6E2	F5H6E2_HUMAN	MYO1C	Unconventional myosin-Ic	1.0000	2.6	2	2	3
Q$288-4	O00159	MYO1C_HUMAN	MYO1C	Unconventional myosin-Ic	1.0000	2.54	2	2	3
						LLQFYAETCPAPER	2	0.9986	2
						MSLLQLVEILQSK	2	0.9997	1
Q$289-1	P61769	B2MG_HUMAN	B2M	Beta-2-microglobulin	1.0000	48.74	5	6	11
Q$289-2	F5H6I0	F5H6I0_HUMAN	B2M	Beta-2-microglobulin form pI 5.3	1.0000	47.54	5	6	11
						DWSFYLLYYTEFTPTEKDEYACR	3	0.8132	1
						IEKVEHSDLSFSK	2	0.9993	3
						SNFLNCYVSGFHPSDIEV	2	0.9079	1
						SNFLNCYVSGFHPSDIEVDLLK	2	0.9376	1
						SNFLNCYVSGFHPSDIEVDLLK	3	0.9996	4
						VEHSDLSFSK	3	0.9338	1
Q$290-1	F5H6T1	F5H6T1_HUMAN	ACTR2	Actin-related protein 2	1.0000	8.85	2	2	2
Q$290-2	P61160-2	ARP2_HUMAN	ACTR2	Isoform 2 of Actin-related protein 2	1.0000	6.77	2	2	2
						GYAFNHSADFETVR	2	0.999	1
						LCYVGYNIEQEQK	2	0.9829	1
Q$291-1	P19827	ITIH1_HUMAN	ITIH1	Inter-alpha-trypsin inhibitor heavy chain H1	1.0000	23.27	21	23	54
Q$291-2	F5H7E1	F5H7E1_HUMAN	ITIH1	Uncharacterized protein	1.0000	32.87	21	23	54
						AAISGENAGLVR	2	0.9991	2
						ADVQAHGEGQEFSITCLVDEEEMK	3	0.9815	2
						AHYVVTSQVVNTANEAR	3	0.905	1
						DYFDLVLFGTR	2	0.9563	2
						EVAFDLEIPK	2	0.9996	3
						FAHYVVTSQVVNTANEAR	2	0.9997	3
						GFSLDEATNLNGGLLR	2	0.9995	4
						GSLVQASEANLQAAQDFVR	2	0.9997	3
						GSLVQASEANLQAAQDFVR	3	0.9923	2
						KAAISGENAGLVR	2	0.9724	1
						LDAQASFLPK	2	0.9951	5
						LWAYLTIQELLAK	2	0.9997	5
						LWAYLTIQELLAK	3	0.9963	2
						LWAYLTIQELLAKR	3	0.9194	1
						MSLDYGFVTPLTSMSIR	2	0.9337	2
						QAVDTAVDGVFIR	2	0.9989	4
						QYYEGSEIVVAGR	2	0.9997	2
						TAFISDFAVTADGNAFIGDIK	2	0.9994	2
						VTFQLTYEEVLK	2	0.9995	2
						VTFQLTYEEVLKR	2	0.8553	1
						VTSQVVNTANEAR	2	0.9807	1
						VVTSQVVNTANEAR	2	0.9944	2
						YFDLVLFGTR	2	0.983	2
Q$292-1	F5H7T4	F5H7T4_HUMAN	C1S	Complement C1s subcomponent (Fragment)	1.0000	17.83	2	2	4
						GFQVVVTLR	2	0.9993	3
						QFGPYCGHGFPGPLNIETK	3	0.9533	1
Q$293-1	P08709	FA7_HUMAN	F7	Coagulation factor VII	1.0000	11.8	4	5	12
Q$293-2	F5H8B0	F5H8B0_HUMAN	F7	Factor VII heavy chain	1.0000	13.85	4	5	12
Q$293-3	P08709-2	FA7_HUMAN	F7	Isoform B of Coagulation factor VII	1.0000	12.39	4	5	12
						DQLQSYICFCLPAFEGR	2	0.9997	5
						DQLQSYICFCLPAFEGR	3	0.9714	2
						FSLVSGWGQLLDR	2	0.9996	2
						GATALELMVLNVPR	2	0.9943	2
						LMTQDCLQQSR	2	0.9438	1
Q$294-1	H7BZ94	H7BZ94_HUMAN	P4HB	Protein disulfide-isomerase	1.0000	13.15	5	6	10
Q$294-2	F5H8J2	F5H8J2_HUMAN	P4HB	Uncharacterized protein	1.0000	13.53	5	6	10
						DAPEEEDHVLVLR	2	0.9576	2
						NFEDVAFDEKK	2	0.9838	1
						THILLFLPK	2	0.9987	2
						VDATEESDLAQQYGVR	2	0.9997	2
						YKPESEELTAER	2	0.9994	1
						YKPESEELTAER	3	0.9985	2
Q$295-1	F6RFD5	F6RFD5_HUMAN	DSTN	Destrin	1.0000	40.74	4	4	10
Q$295-2	P60981	DEST_HUMAN	DSTN	Destrin	1.0000	33.33	4	4	10
Q$295-3	P60981-2	DEST_HUMAN	DSTN	Isoform 2 of Destrin	1.0000	37.16	4	4	10
						AVIFCLSADKK	2	0.9463	1
						EILVGDVGVTITDPFK	2	0.9989	1
						KEELMFFLWAPELAPLK	3	0.9993	5
						YALYDASFETK	2	0.9993	3
Q$296-1	F6U211	F6U211_HUMAN	RPS10	40S ribosomal protein S10	0.9771	5.23	1	1	2
Q$296-2	P46783	RS10_HUMAN	RPS10	40S ribosomal protein S10	0.9771	5.45	1	1	2
Q$296-3	S4R435	S4R435_HUMAN	RPS10-NUDT3	Protein RPS10-NUDT3 (Fragment)	0.9771	3.15	1	1	2
						IAIYELLFK	2	0.9771	2
Q$297-1	F6UT28	F6UT28_HUMAN	GNB1	Guanine nucleotide-binding protein G(I)/G(S)/G(T) subunit be	1.0000	7.88	1	1	1
Q$297-2	F6X3N5	F6X3N5_HUMAN	GNB1	Guanine nucleotide-binding protein G(I)/G(S)/G(T) subunit be	1.0000	7.98	1	1	1
						ELAGHTGYLSCCR	2	0.9973	1
Q$298-1	P50502	F10A1_HUMAN	ST13	Hsc70-interacting protein	1.0000	10.03	3	3	7
Q$298-2	F6VDH7	F6VDH7_HUMAN	ST13	Hsc70-interacting protein (Fragment)	1.0000	22.84	3	3	7
Q$298-3	H7C3I1	H7C3I1_HUMAN	ST13	Hsc70-interacting protein (Fragment)	1.0000	25.34	3	3	7
Q$298-4	Q8IZP2	ST134_HUMAN	ST13P4	Putative protein FAM10A4	1.0000	15.42	3	3	7
						AIDLFTDAIK	2	0.9861	2
						AIEINPDSAQPYK	2	0.9995	3
						VAAIEALNDGELQK	2	0.9996	2
Q$299-1	F8VWS0	F8VWS0_HUMAN	RPLP0	60S acidic ribosomal protein P0	1.0000	4.27	1	1	3
Q$299-2	G3V210	G3V210_HUMAN	RPLP0	60S acidic ribosomal protein P0	1.0000	7.23	1	1	3
Q$299-3	Q3B7A4	Q3B7A4_HUMAN	RPLP0	60S acidic ribosomal protein P0	1.0000	4.71	1	1	3
Q$299-4	F8VPE8	F8VPE8_HUMAN	RPLP0	60S acidic ribosomal protein P0 (Fragment)	1.0000	7.84	1	1	3
Q$299-5	F8VU65	F8VU65_HUMAN	RPLP0	60S acidic ribosomal protein P0 (Fragment)	1.0000	4.86	1	1	3
Q$299-6	F8VW21	F8VW21_HUMAN	RPLP0	60S acidic ribosomal protein P0 (Fragment)	1.0000	4.92	1	1	3
Q$299-7	Q8NHW5	RLA0L_HUMAN	RPLP0P6	60S acidic ribosomal protein P0-like	1.0000	3.79	1	1	3
						TSFFQALGITTK	2	0.9997	3
Q$300-1	F8VPF3	F8VPF3_HUMAN	MYL6	Myosin light polypeptide 6 (Fragment)	1.0000	20	2	2	4
						ALGQNPTNAEVLK	2	0.9994	2
						DQGTYEDYVEGLR	2	0.9909	2
Q$301-1	Q9UHB6-4	LIMA1_HUMAN	LIMA1	Isoform 4 of LIM domain and actin-binding protein 1	0.9996	2.76	2	2	2
Q$301-2	F8VRN8	F8VRN8_HUMAN	LIMA1	LIM domain and actin-binding protein 1	0.9996	5.41	2	2	2
Q$301-3	Q9UHB6	LIMA1_HUMAN	LIMA1	LIM domain and actin-binding protein 1	0.9996	2.77	2	2	2
Q$301-4	F8VTU2	F8VTU2_HUMAN	LIMA1	LIM domain and actin-binding protein 1 (Fragment)	0.9996	16.15	2	2	2
Q$301-5	F8VVQ7	F8VVQ7_HUMAN	LIMA1	LIM domain and actin-binding protein 1 (Fragment)	0.9996	18.92	2	2	2
						SNTENLSQHFR	2	0.9903	1
						SSAIVEIFSK	2	0.9817	1
Q$302-1	F8VTQ5	F8VTQ5_HUMAN	HNRNPA1	Heterogeneous nuclear ribonucleoprotein A1 (Fragment)	1.0000	26.21	4	4	11
						IEVIEIMTDR	2	0.9984	2
						KLFIGGLSFETTDESLR	2	0.9997	2
						LFIGGLSFETTDESLR	2	0.9997	3
						SESPKEPEQLR	2	0.9968	4
Q$303-1	G3V203	G3V203_HUMAN	RPL18	60S ribosomal protein L18	1.0000	7.93	1	1	2
Q$303-2	Q07020	RL18_HUMAN	RPL18	60S ribosomal protein L18	1.0000	6.91	1	1	2

Q$303-3	F8VUA6	F8VUA6_HUMAN	RPL18	60S ribosomal protein L18 (Fragment)	1.0000	10	1	1	2
Q$303-4	H0YHA7	H0YHA7_HUMAN	RPL18	60S ribosomal protein L18 (Fragment)	1.0000	7.78	1	1	2
Q$303-5	J3QQ67	J3QQ67_HUMAN	RPL18	60S ribosomal protein L18 (Fragment)	1.0000	6.84	1	1	2
Q$303-6	Q07020-2	RL18_HUMAN	RPL18	Isoform 2 of 60S ribosomal protein L18	1.0000	8.18	1	1	2
						ILTFDQLALDSPK	2	0.9992	2
Q$304-1	F8VV32	F8VV32_HUMAN	LYZ	Lysozyme C	1.0000	29.81	3	3	6
						GISLANWMCLAK	2	0.9984	1
						LGMDGYR	2	0.9993	1
						STDYGIFQINSR	2	0.9996	4
Q$305-1	F8VZ49	F8VZ49_HUMAN	HNRNPA1	Heterogeneous nuclear ribonucleoprotein A1 (Fragment)	1.0000	6.93	1	1	3
Q$305-2	Q32P51	RA1L2_HUMAN	HNRNPA1L2	Heterogeneous nuclear ribonucleoprotein A1-like 2	1.0000	5	1	1	3
						LFIGGLSFETTDESLR	2	0.9997	3
Q$306-1	F8VZY9	F8VZY9_HUMAN	KRT18	Keratin, type I cytoskeletal 18	1.0000	48.08	18	21	29
Q$306-2	P05783	K1C18_HUMAN	KRT18	Keratin, type I cytoskeletal 18	1.0000	43.72	18	21	29
						AQIFANTVDNAR	2	0.9996	2
						AQYDELARK	2	0.9957	1
						ASLENSLR	2	0.818	1
						DWSHYFK	2	0.998	1
						GLQAQIASSGLTVEVDAPK	2	0.9997	2
						GLQAQIASSGLTVEVDAPK	3	0.9987	1
						IVLQIDNAR	2	0.9996	2
						KVIDDTNITR	2	0.9983	2
						LEAEIATYR	2	0.9935	1
						LQLETEIEALKEELLFMK	2	0.9991	1
						LQLETEIEALKEELLFMKK	4	0.8827	1
						QAQEYEALLNIK	2	0.9988	3
						QSVENDIHGLR	2	0.9845	1
						QSVENDIHGLR	3	0.9375	1
						SAEVGAAETTLTELR	2	0.9467	1
						TVQSLEIDLDSMR	2	0.9996	2
						VIDDTNITR	2	0.9987	1
						VKLEAEIATYR	2	0.904	1
						VKYETELAMR	2	0.9632	1
						VKYETELAMR	3	0.9919	1
						YALQMEQLNGILLHLESELAQTR	3	0.9993	2
Q$307-1	F8W543	F8W543_HUMAN	NAP1L1	Nucleosome assembly protein 1-like 1	1.0000	6.4	2	2	4
Q$307-2	H0YH88	H0YH88_HUMAN	NAP1L1	Nucleosome assembly protein 1-like 1 (Fragment)	1.0000	11.86	2	2	4
						FYEEVHDLER	2	0.9994	2
						GIPEFWLTVFK	2	0.9985	2
Q$308-1	F8W646	F8W646_HUMAN	HNRNPA1	Heterogeneous nuclear ribonucleoprotein A1 (Fragment)	1.0000	40.38	5	5	11
						EDSQRPGAHLTVK	2	0.9891	1
						KLFIGGLSFETTDESLR	2	0.9997	2
						LFIGGLSFETTDESLR	2	0.9997	3
						SESPKEPEQLR	2	0.9968	4
						SHFEQWGTLTDCVVMRDPNTKR	4	0.9856	1
Q$309-1	F8W6I7	F8W6I7_HUMAN	HNRNPA1	Heterogeneous nuclear ribonucleoprotein A1	1.0000	23.78	6	6	13
Q$309-2	P09651	ROA1_HUMAN	HNRNPA1	Heterogeneous nuclear ribonucleoprotein A1	1.0000	19.62	6	6	13
Q$309-3	P09651-3	ROA1_HUMAN	HNRNPA1	Isoform 2 of Heterogeneous nuclear ribonucleoprotein A1	1.0000	27.34	6	6	13
Q$309-4	P09651-2	ROA1_HUMAN	HNRNPA1	Isoform A1-A of Heterogeneous nuclear ribonucleoprotein A1	1.0000	22.81	6	6	13
						EDSQRPGAHLTVK	2	0.9891	1
						IEVIEIMTDR	2	0.9984	2
						KLFIGGLSFETTDESLR	2	0.9997	2
						LFIGGLSFETTDESLR	2	0.9997	3
						SESPKEPEQLR	2	0.9968	4
						SHFEQWGTLTDCVVMRDPNTKR	4	0.9856	1
Q$310-1	P35580-2	MYH10_HUMAN	MYH10	Isoform 2 of Myosin-10	1.0000	5.82	8	10	18
Q$310-2	P35580-3	MYH10_HUMAN	MYH10	Isoform 3 of Myosin-10	1.0000	5.81	8	10	18
Q$310-3	P35580-4	MYH10_HUMAN	MYH10	Isoform 4 of Myosin-10	1.0000	5.78	8	10	18
Q$310-4	P35580-5	MYH10_HUMAN	MYH10	Isoform 5 of Myosin-10	1.0000	5.84	8	10	18
Q$310-5	F8W6L6	F8W6L6_HUMAN	MYH10	Myosin-10	1.0000	5.82	8	10	18
Q$310-6	P35580	MYH10_HUMAN	MYH10	Myosin-10	1.0000	5.87	8	10	18
						ADEWLMK	2	0.9937	1
						AGVLAHLEEER	2	0.9993	1
						DKADFCIIHYAGK	3	0.8934	2
						LDPHLVLDQLR	2	0.9489	1
						LDPHLVLDQLR	3	0.9995	2
						LQQLFNHTMFILEQEEYQR	3	0.9995	3
						SLEAEILQLQEELASSER	2	0.9997	1
						SLEAEILQLQEELASSER	3	0.955	2
						TQLEELEDELQATEDAK	2	0.9997	3
						VEDMAELTCLNEASVLHNLK	3	0.9921	2
Q$311-1	P02751	FINC_HUMAN	FN1	Fibronectin	1.0000	5.74	10	10	18
Q$311-2	P02751-10	FINC_HUMAN	FN1	Isoform 10 of Fibronectin	1.0000	6.3	10	10	18
Q$311-3	P02751-11	FINC_HUMAN	FN1	Isoform 11 of Fibronectin	1.0000	5.74	10	10	18
Q$311-4	P02751-12	FINC_HUMAN	FN1	Isoform 12 of Fibronectin	1.0000	6.82	10	10	18
Q$311-5	P02751-13	FINC_HUMAN	FN1	Isoform 13 of Fibronectin	1.0000	6.04	10	10	18
Q$311-6	P02751-14	FINC_HUMAN	FN1	Isoform 14 of Fibronectin	1.0000	6.05	10	10	18
Q$311-7	P02751-15	FINC_HUMAN	FN1	Isoform 15 of Fibronectin	1.0000	5.53	10	10	18
Q$311-8	P02751-17	FINC_HUMAN	FN1	Isoform 17 of Fibronectin	1.0000	5.88	10	10	18
Q$311-9	P02751-3	FINC_HUMAN	FN1	Isoform 3 of Fibronectin	1.0000	5.82	10	10	18
Q$311-10	P02751-5	FINC_HUMAN	FN1	Isoform 5 of Fibronectin	1.0000	6.2	10	10	18
Q$311-11	P02751-7	FINC_HUMAN	FN1	Isoform 7 of Fibronectin	1.0000	5.6	10	10	18
Q$311-12	P02751-8	FINC_HUMAN	FN1	Isoform 8 of Fibronectin	1.0000	5.97	10	10	18
Q$311-13	P02751-9	FINC_HUMAN	FN1	Isoform 9 of Fibronectin	1.0000	6.12	10	10	18
Q$311-14	F8W7G7	F8W7G7_HUMAN	FN1	Ugl-Y3	1.0000	6.2	10	10	18
						DLQFVEVTDVK	2	0.9906	2
						EYLGAICSCTCFGGQR	2	0.9963	1
						SSPVVIDASTAIDAPSNLR	2	0.9995	2
						STTPDITGYR	2	0.8916	1
						SYTITGLQPGTDYK	2	0.9993	3
						TFYSCTTEGR	2	0.9808	1
						TYLGNALVCTCYGGSR	2	0.9967	2
						VPGTSTSATLTGLTR	2	0.9996	4
						VTDATETTITISWR	2	0.9847	1
						WSRPQAPITGYR	3	0.8168	1
Q$312-1	F8W7I2	F8W7I2_HUMAN	PTGDS	Prostaglandin-H2 D-isomerase	1.0000	14.73	2	2	5
						AQGFTEDTIVFLPQTDK	2	0.9996	3
						TMLLQPAGSLGSYSYR	2	0.9973	2
Q$313-1	F8W7I9	F8W7I9_HUMAN	RANGAP1	Ran GTPase-activating protein 1	1.0000	4.51	2	2	2
Q$313-2	H0Y4Q3	H0Y4Q3_HUMAN	RANGAP1	Ran GTPase-activating protein 1 (Fragment)	1.0000	9.49	2	2	2
						QVEVINFGDCLVR	2	0.9871	1
						SSACFTLQELK	2	0.9947	1
Q$314-1	F8W7L3	F8W7L3_HUMAN	A2M	Alpha-2-macroglobulin (Fragment)	1.0000	13.1	1	1	2
						SLFTDLEAENDVLHCVAFAVPK	3	0.999	2
Q$315-1	F8W810	F8W810_HUMAN		Uncharacterized protein	0.9995	2.58	1	1	1
Q$315-2	P41091	IF2G_HUMAN	EIF2S3	Eukaryotic translation initiation factor 2 subunit 3	0.9995	2.54	1	1	1
Q$315-3	Q2VIR3-2	IF2GL_HUMAN	EIF2S3L	Isoform 2 of Putative eukaryotic translation initiation factor 2	0.9995	2.58	1	1	1
Q$315-4	Q2VIR3	IF2GL_HUMAN	EIF2S3L	Putative eukaryotic translation initiation factor 2 subunit 3-li	0.9995	2.54	1	1	1
						YNIEVVCEYIVK	2	0.9915	1
Q$316-1	G3XAM7	G3XAM7_HUMAN	CTNNA1	Catenin (Cadherin-associated protein), alpha 1, 102kDa, isofo	1.0000	7.02	3	3	3
Q$316-2	F8W845	F8W845_HUMAN	CTNNA1	Catenin alpha-1	1.0000	7.35	3	3	3
Q$316-3	P35221	CTNA1_HUMAN	CTNNA1	Catenin alpha-1	1.0000	6.51	3	3	3
Q$316-4	P35221-2	CTNA1_HUMAN	CTNNA1	Isoform 2 of Catenin alpha-1	1.0000	6.34	3	3	3
						LIEVANLACSISNNEEGVK	2	0.9792	1
						MSASQLEALCPQVINAALALAAKPQSK	3	0.9866	1
						QIIVDPLSFSEER	2	0.9465	1
Q$317-1	F8W9U3	F8W9U3_HUMAN	ABHD14B	Alpha/beta hydrolase domain-containing protein 14B	1.0000	16.35	4	5	10
						EALPGSGQAR	2	0.9926	1
						EGTIQVQGQALFFR	2	0.9994	4
						FSVLLLHGIR	2	0.9996	2
						FSVLLLHGIR	3	0.9984	2
						SVLLLHGIR	2	0.888	1
Q$318-1	F8WDD7	F8WDD7_HUMAN	ARPC4	Actin-related protein 2/3 complex subunit 4	1.0000	16.81	2	2	4

Q$318-2	P59998	ARPC4_HUMAN	ARPC4	Actin-related protein 2/3 complex subunit 4	1.0000	11.31	2	2	4
Q$318-3	P59998-2	ARPC4_HUMAN	ARPC4	Isoform 2 of Actin-related protein 2/3 complex subunit 4	1.0000	3.04	2	2	4
Q$318-4	P59998-3	ARPC4_HUMAN	ARPC4	Isoform 3 of Actin-related protein 2/3 complex subunit 4	1.0000	10.16	2	2	4
Q$318-5	F8WCF6	F8WCF6_HUMAN	ARPC4-TTLL3	Protein ARPC4-TTLL3	1.0000	10.5	2	2	4
						AENFFILR	2	0.9995	3
						VLIEGSINSVR	2	0.9947	1
Q$319-1	F8WCZ6	F8WCZ6_HUMAN	C1S	Complement C1s subcomponent	1.0000	13.05	5	5	11
						DVVQITCLDGFEVVEGR	2	0.9995	4
						GFQVVVTLR	2	0.9993	3
						LPVAPLR	2	0.972	1
						QFGPYCGHGFPGPLNIETK	3	0.9533	1
						SNALDIIFQTDLTGQK	2	0.9978	2
Q$320-1	F8WE04	F8WE04_HUMAN	HSPB1	Heat shock protein beta-1	1.0000	13.98	2	2	11
						LFDQAFGLPR	2	0.9998	6
						VSLDVNHFAPDELTVK	2	0.9997	5
Q$321-1	G3V1V0	G3V1V0_HUMAN	MYL6	Myosin light polypeptide 6	1.0000	61.49	8	10	18
						ALGQNPTNAEVLK	2	0.9994	2
						DQGTYEDYVEGLR	2	0.9909	2
						EAFQLFDR	2	0.9981	2
						HVLVTLGEK	2	0.9993	1
						ILYSQCGDVMR	2	0.9992	1
						MTEEEVEMLVAGHEDSNGCINYEGKRR	3	0.8952	1
						NKDQGTYEDYVEGLR	2	0.9995	2
						NKDQGTYEDYVEGLR	3	0.9892	2
						VLDFEHFLPMLQTVAK	2	0.9996	3
						VLDFEHFLPMLQTVAK	3	0.9996	2
Q$322-1	G3V2W1	G3V2W1_HUMAN	SERPINA10	Protein Z-dependent protease inhibitor	0.9598	2.07	1	1	2
Q$322-2	Q9UK55	ZPI_HUMAN	SERPINA10	Protein Z-dependent protease inhibitor	0.9598	2.25	1	1	2
						LILVDYILFK	2	0.9598	2
Q$323-1	P42226-2	STAT6_HUMAN	STAT6	Isoform 2 of Signal transducer and activator of transcription (	0.9255	2.38	1	1	1
Q$323-2	P42226-3	STAT6_HUMAN	STAT6	Isoform 3 of Signal transducer and activator of transcription (	0.9255	2.17	1	1	1
Q$323-3	P42226	STAT6_HUMAN	STAT6	Signal transducer and activator of transcription 6	0.9255	1.89	1	1	1
Q$323-4	G3V2X7	G3V2X7_HUMAN	STAT6	Signal transducer and activator of transcription 6 (Fragment)	0.9255	10.39	1	1	1
						FSDSEIGGITIAHVIR	3	0.9255	1
Q$324-1	Q86XR7-2	TCAM2_HUMAN	TICAM2	Isoform 2 of TIR domain-containing adapter molecule 2	0.9577	2.97	1	1	2
Q$324-2	Q9Y3B3-2	TMED7_HUMAN	TMED7	Isoform 2 of Transmembrane emp24 domain-containing prot	0.9577	6.38	1	1	2
Q$324-3	Q9Y3B3	TMED7_HUMAN	TMED7	Transmembrane emp24 domain-containing protein 7	0.9577	5.36	1	1	2
Q$324-4	G3V2Y2	G3V2Y2_HUMAN	TMED7	Transmembrane emp24 domain-containing protein 7 (Fragm	0.9577	14.81	1	1	2
						QCFYEDIAQGTK	2	0.9577	2
Q$325-1	G3V3A0	G3V3A0_HUMAN	SERPINA3	Alpha-1-antichymotrypsin	1.0000	30.24	6	6	16
						ADLSGITGAR	2	0.9993	3
						EIGELYLPK	2	0.9982	2
						FNRPFLMIIVPTDTQNIFFMSK	3	0.9991	5
						ITLLSALVETR	2	0.9997	3
						NLAVSQVVHK	2	0.9969	2
						TLLSALVETR	2	0.8869	1
Q$326-1	G3V3E1	G3V3E1_HUMAN	SKOR1	SKI family transcriptional corepressor 1	0.9976	6.8	2	2	2
Q$326-2	G3V5H7	G3V5H7_HUMAN	SKOR1	SKI family transcriptional corepressor 1	0.9976	6.72	2	2	2
Q$326-3	P84550	SKOR1_HUMAN	SKOR1	SKI family transcriptional corepressor 1	0.9976	6.53	2	2	2
						GEDGLTLDVTGTHLVEK	3	0.8056	1
						TFSLQGGGGGGANGGSGGQGKGGAGGGGGGGPGCGAEMAPGPPPHK	3	0.8299	1
Q$327-1	G3V453	G3V453_HUMAN	SAV1	Protein salvador homolog 1 (Fragment)	0.9418	11.69	1	1	1
						CASCLQTLR	2	0.9418	1
Q$328-1	G3V5I3	G3V5I3_HUMAN	SERPINA3	Alpha-1-antichymotrypsin (Fragment)	1.0000	51.11	2	2	5
						AAAATGTIFTFR	2	0.9997	3
						AVVEVDESGTR	2	0.9997	2
Q$329-1	Q6H9L7-3	ISM2_HUMAN	ISM2	Isoform 3 of Isthmin-2	0.9669	2.86	1	1	2
Q$329-2	Q6H9L7-5	ISM2_HUMAN	ISM2	Isoform 5 of Isthmin-2	0.9669	2.86	1	1	2
Q$329-3	G3XAI3	G3XAI3_HUMAN	ISM2	Isthmin-2	0.9669	2.9	1	1	2
Q$329-4	Q6H9L7	ISM2_HUMAN	ISM2	Isthmin-2	0.9669	2.45	1	1	2
						TRPCGYGCTATETR	2	0.9669	2
Q$330-1	P07225	PROS_HUMAN	PROS1	Vitamin K-dependent protein S	1.0000	29.17	17	21	46
						AHSCPSVWK	2	0.9907	1
						AHSCPSVWKK	2	0.9996	3
						DVDECSLKPSICGTAVCK	2	0.9997	2
						DVDECSLKPSICGTAVCK	3	0.9937	1
						FSAEFDFR	2	0.9947	1
						HCLVTVEK	2	0.9996	1
						IQALSLCSDQQSH	2	0.975	2
						IQALSLCSDQQSHLEF	2	0.986	2
						IQALSLCSDQQSHLEFR	2	0.9997	3
						IQALSLCSDQQSHLEFR	3	0.999	2
						ITTGGDVINNGLWNMVSVEELEHSISIK	3	0.9861	1
						KVESELIKPINPR	2	0.9997	2
						KVESELIKPINPR	3	0.9985	2
						NGFVMLSNKK	2	0.9966	2
						NNLELSTPLK	2	0.9975	2
						NNLELSTPLKIETISHEDLQR	3	0.9994	3
						SLCSDQQSHLEFR	2	0.9987	2
						SQDILLSVENTVIYR	2	0.9997	7
						SQDILLSVENTVIYR	3	0.9996	2
						VNRNNLELSTPLKIETISHEDLQR	3	0.9976	3
						VYFAGFPR	2	0.9985	2
Q$331-1	G5E9G0	G5E9G0_HUMAN	RPL3	60S ribosomal protein L3	0.9999	8.83	2	2	3
						NNASTDYDLSDK	2	0.8779	1
						SINPLGGFVHYGEVTNDFV	2	0.978	2
Q$332-1	G5E9J1	G5E9J1_HUMAN	SH3GLB2	Endophilin-B2	0.9978	45.83	1	1	1
						LASDAGIFFTR	2	0.9884	1
Q$333-1	G5EA52	G5EA52_HUMAN	PDIA3	Protein disulfide-isomerase	1.0000	20	6	6	9
						EATNPPVIQEEKPK	2	0.9995	1
						ELSDFISYLQR	2	0.9994	2
						FISDKDASIVGFFDDSFSEAHSEFLK	3	0.9975	2
						LAPEYEAAATR	2	0.9985	1
						LSKDPNIVIAK	2	0.9879	2
						TFSHELSDFGLESTAGEIPVVAIR	3	0.991	1
Q$334-1	G8JL88	G8JL88_HUMAN	APOL1	Apolipoprotein L1	0.9727	5.42	1	1	2
Q$334-2	O14791	APOL1_HUMAN	APOL1	Apolipoprotein L1	0.9727	3.77	1	1	2
Q$334-3	O14791-2	APOL1_HUMAN	APOL1	Isoform 2 of Apolipoprotein L1	0.9727	3.62	1	1	2
Q$334-4	O14791-3	APOL1_HUMAN	APOL1	Isoform 3 of Apolipoprotein L1	0.9727	3.95	1	1	2
						VTEPISAESGEQVER	2	0.9727	2
Q$335-1	P60660-2	MYL6_HUMAN	MYL6	Isoform Smooth muscle of Myosin light polypeptide 6	1.0000	65.56	8	10	20
Q$335-2	G8JLA2	G8JLA2_HUMAN	MYL6	Myosin light polypeptide 6	1.0000	65.13	8	10	20
						ALGQNPTNAEVLK	2	0.9994	2
						DQGTYEDYVEGLR	2	0.9909	2
						EAFQLFDR	2	0.9981	2
						HVLVTLGEK	2	0.9993	1
						ILYSQCGDVMR	2	0.9992	1
						MTEEEVEMLVAGHEDSNGCINYEELVR	3	0.995	3
						NKDQGTYEDYVEGLR	2	0.9995	2
						NKDQGTYEDYVEGLR	3	0.9892	2
						VLDFEHFLPMLQTVAK	2	0.9996	3
						VLDFEHFLPMLQTVAK	3	0.9996	2
Q$336-1	G8JLD5	G8JLD5_HUMAN	DNM1L	Dynamin-1-like protein	0.9996	3.51	2	2	2
Q$336-2	O00429	DNM1L_HUMAN	DNM1L	Dynamin-1-like protein	0.9996	3.4	2	2	2
Q$336-3	O00429-3	DNM1L_HUMAN	DNM1L	Isoform 2 of Dynamin-1-like protein	0.9996	3.52	2	2	2
Q$336-4	O00429-4	DNM1L_HUMAN	DNM1L	Isoform 3 of Dynamin-1-like protein	0.9996	3.58	2	2	2
Q$336-5	O00429-2	DNM1L_HUMAN	DNM1L	Isoform 4 of Dynamin-1-like protein	0.9996	3.45	2	2	2
Q$336-6	O00429-5	DNM1L_HUMAN	DNM1L	Isoform 5 of Dynamin-1-like protein	0.9996	3.52	2	2	2
Q$336-7	O00429-6	DNM1L_HUMAN	DNM1L	Isoform 6 of Dynamin-1-like protein	0.9996	3.34	2	2	2
Q$336-8	O00429-8	DNM1L_HUMAN	DNM1L	Isoform 8 of Dynamin-1-like protein	0.9996	3.39	2	2	2
						ALQGASQIIAEIR	2	0.9843	1
						YIETSELCGGAR	2	0.9722	1
Q$337-1	H0Y300	H0Y300_HUMAN	HP	Haptoglobin	1.0000	15.16	4	4	9

Q$337-2	J3QR68	J3QR68_HUMAN	HP	Haptoglobin (Fragment)	1.0000	16.58	4	4	9
						IAPTLTLYVGK	2	0.909	1
						SCAVAEYGVYVK	2	0.9997	4
						VTSIQDWVQK	2	0.9993	2
						YQEDTCYGDAGSAFAVHDLEEDTWYATGILSFDK	3	0.9973	2
Q$338-1	H0Y3H6	H0Y3H6_HUMAN	C2	Complement C2b fragment (Fragment)	1.0000	8.37	3	3	7
Q$338-2	P06681-2	CO2_HUMAN	C2	Isoform 2 of Complement C2	1.0000	8.18	3	3	7
Q$338-3	P06681-3	CO2_HUMAN	C2	Isoform 3 of Complement C2	1.0000	7.1	3	3	7
						CSSNLVLTGSSER	2	0.9988	3
						EVVTDQFLCSGTQEDESPCKGESGGAVFLER	3	0.9981	3
						GESGGAVFLER	2	0.8935	1
Q$339-1	H0Y482	H0Y482_HUMAN	EPB41L1	Band 4.1-like protein 1 (Fragment)	1.0000	16.81	1	1	1
Q$339-2	Q9H4G0-3	E41L1_HUMAN	EPB41L1	Isoform 3 of Band 4.1-like protein 1	1.0000	2.59	1	1	1
						IIITGDEDVDQDQALALAIK	2	0.9979	1
Q$340-1	H0Y494	H0Y494_HUMAN	SIK3	Serine/threonine-protein kinase SIK3	0.9576	1.98	1	1	1
Q$340-2	J3KPC8	J3KPC8_HUMAN	SIK3	Serine/threonine-protein kinase SIK3	0.9576	1.89	1	1	1
						MAAAAASGAGGAAGAGTGGAGPAGR	3	0.8051	1
Q$341-1	P12268	IMDH2_HUMAN	IMPDH2	Inosine-5'-monophosphate dehydrogenase 2	1.0000	12.84	5	6	8
Q$341-2	H0Y4R1	H0Y4R1_HUMAN	IMPDH2	Inosine-5'-monophosphate dehydrogenase 2 (Fragment)	1.0000	14.04	5	6	8
						DIDFLKEEEHDCFLEEIMTK	3	0.9155	1
						HGFCGIPITDTGR	2	0.9992	1
						LVGIISSR	2	0.996	1
						NLIDAGVDALR	2	0.9869	1
						REDLVVAPAGITLK	2	0.9996	2
						REDLVVAPAGITLK	3	0.998	2
Q$342-1	P41222	PTGDS_HUMAN	PTGDS	Prostaglandin-H2 D-isomerase	1.0000	32.63	3	3	7
Q$342-2	H0Y5A1	H0Y5A1_HUMAN	PTGDS	Prostaglandin-H2 D-isomerase (Fragment)	1.0000	29.11	3	3	7
						AQGFTEDTIVFLPQTDK	2	0.9996	3
						SPHWGSTYSVSVVETDYDQYALLYSQGSK	3	0.9717	2
						TMLLQPAGSLGSYSYR	2	0.9973	2
Q$343-1	H0Y5D1	H0Y5D1_HUMAN	C1S	Complement C1s subcomponent (Fragment)	1.0000	16.9	4	4	9
						GFQVVVTLR	2	0.9993	3
						QFGPYCGHGFPGPLNIETK	3	0.9533	1
						SNALDIIFQTDLTGQK	2	0.9978	2
						SWDIEVPEGYGIHLYF	2	0.9818	3
Q$344-1	H0Y626	H0Y626_HUMAN		Uncharacterized protein	1.0000	2.62	2	2	3
Q$344-2	K7EL43	K7EL43_HUMAN	TRIM16	Tripartite motif-containing protein 16 (Fragment)	1.0000	13.59	2	2	3
						AVAEMQFGELLAAVR	2	0.9994	1
						SVLVSVSEVK	2	0.9846	2
Q$345-1	H0Y630	H0Y630_HUMAN	STK24	Serine/threonine-protein kinase 24 12 kDa subunit (Fragmen	1.0000	20.05	5	6	12
						AANVLLSEHGEVK	2	0.9963	1
						ADIWSLGITAIELAR	2	0.9997	3
						ADIWSLGITAIELAR	3	0.9996	2
						GAIYLAEEACPGISDTMVAQLVQR	3	0.9952	2
						SQACGGNLGSIEELR	2	0.9997	3
						VLFLIPK	2	0.9889	1
Q$346-1	Q6ZNJ1-2	NBEL2_HUMAN	NBEAL2	Isoform 2 of Neurobeachin-like protein 2	0.9885	0.74	1	1	1
Q$346-2	Q6ZNJ1-3	NBEL2_HUMAN	NBEAL2	Isoform 3 of Neurobeachin-like protein 2	0.9885	0.7	1	1	1
Q$346-3	Q6ZNJ1	NBEL2_HUMAN	NBEAL2	Neurobeachin-like protein 2	0.9885	0.69	1	1	1
Q$346-4	H0Y764	H0Y764_HUMAN	NBEAL2	Neurobeachin-like protein 2 (Fragment)	0.9885	0.93	1	1	1
						CPSLSEPFSSCCIGSAGYR	2	0.9802	1
Q$347-1	H0Y7N1	H0Y7N1_HUMAN	DDAH2	N(G),N(G)-dimethylarginine dimethylaminohydrolase 2 (Frag	1.0000	22.87	4	5	8
Q$347-2	Q5SRR8	Q5SRR8_HUMAN	DDAH2	N(G),N(G)-dimethylarginine dimethylaminohydrolase 2 (Frag	1.0000	18.22	4	5	8
Q$347-3	Q5SSV3	Q5SSV3_HUMAN	DDAH2	N(G),N(G)-dimethylarginine dimethylaminohydrolase 2 (Frag	1.0000	19.03	4	5	8
						DFAVSTVPVSGPSHLR	2	0.9996	3
						DFAVSTVPVSGPSHLR	3	0.9982	1
						EFFVGLSK	2	0.9909	1
						GAEIVADTFR	2	0.9985	2
						KALQDLGLR	2	0.9989	1
Q$348-1	H0Y8W2	H0Y8W2_HUMAN	GNB2L1	Guanine nucleotide-binding protein subunit beta-2-like 1 (Fra	1.0000	12.77	3	3	3
						DVLSVAFSSDNR	2	0.983	1
						LWDLTTGTTTR	2	0.938	1
						YWLCAATGPSIK	2	0.9997	1
Q$349-1	H0Y9Q1	H0Y9Q1_HUMAN	LAP3	Cytosol aminopeptidase (Fragment)	1.0000	5.77	1	1	2
						GVLFASGQNLAR	2	0.9994	2
Q$350-1	P03952	KLKB1_HUMAN	KLKB1	Plasma kallikrein	1.0000	5.64	3	3	7
Q$350-2	H0YAC1	H0YAC1_HUMAN	KLKB1	Plasma kallikrein heavy chain (Fragment)	1.0000	5.25	3	3	7
						EIIIHQNYK	2	0.8833	1
						IAYGTQGSSGYSLR	2	0.9994	2
						LVGITSWGEGCAR	2	0.9977	4
Q$351-1	J3KPE3	J3KPE3_HUMAN	GNB2L1	Guanine nucleotide-binding protein subunit beta-2-like 1	1.0000	16.12	4	4	5
Q$351-2	H0YAF8	H0YAF8_HUMAN	GNB2L1	Guanine nucleotide-binding protein subunit beta-2-like 1 (Fra	1.0000	22.22	4	4	5
						DVLSVAFSSDNR	2	0.983	1
						LWDLTTGTTTR	2	0.938	1
						VWQVTIGTR	2	0.9992	2
						YWLCAATGPSIK	2	0.9997	1
Q$352-1	P60228	EIF3E_HUMAN	EIF3E	Eukaryotic translation initiation factor 3 subunit E	1.0000	9.44	3	3	5
Q$352-2	H0YBR5	H0YBR5_HUMAN	EIF3E	Eukaryotic translation initiation factor 3 subunit E (Fragment	1.0000	24.56	3	3	5
						HLVFPLLEFLSVK	3	0.9994	3
						LDLLSDTNMVDFAMDVYK	2	0.9754	1
						QLQAETEPIVK	2	0.9295	1
Q$353-1	P04233	HG2A_HUMAN	CD74	HLA class II histocompatibility antigen gamma chain	0.9765	6.42	1	1	3
Q$353-2	H0YBZ2	H0YBZ2_HUMAN	CD74	HLA class II histocompatibility antigen gamma chain (Fragme	0.9765	6.01	1	1	3
						CQEEVSHIPAVHPGSFRPK	3	0.9765	3
Q$354-1	H0YE82	H0YE82_HUMAN	NAGK	N-acetyl-D-glucosamine kinase (Fragment)	1.0000	19.05	1	1	2
Q$354-2	H0YEB7	H0YEB7_HUMAN	NAGK	N-acetyl-D-glucosamine kinase (Fragment)	1.0000	8.79	1	1	2
						SLGLSLSGGDQEDAGR	2	0.9996	2
Q$355-1	H7C286	H7C286_HUMAN	NAGK	N-acetyl-D-glucosamine kinase	0.9997	5.61	1	1	1
Q$355-2	H0YF44	H0YF44_HUMAN	NAGK	N-acetyl-D-glucosamine kinase (Fragment)	0.9997	10.09	1	1	1
						EGFLLALTQGR	2	0.8813	1
Q$356-1	H0YFH3	H0YFH3_HUMAN	C1R	Complement C1r subcomponent	1.0000	7.5	3	3	8
Q$356-2	P00736	C1R_HUMAN	C1R	Complement C1r subcomponent	1.0000	6.95	3	3	8
						LPVANPQACENWLR	2	0.9983	3
						LVFQQFDLEPSEGCFYDYVK	2	0.9993	3
						WVATGIVSWGIGCSR	2	0.9985	2
Q$357-1	H0YFX9	H0YFX9_HUMAN	H2AFJ	Histone H2A (Fragment)	1.0000	20.65	2	2	5
						AVLEYLTAEILELAGNAAR	3	0.9888	3
						VLEYLTAEILELAGNAAR	3	0.9791	2
Q$358-1	H0YG07	H0YG07_HUMAN	MLEC	Malectin (Fragment)	0.9999	6.94	1	1	3
						FAEVYFAQSQQK	2	0.9992	3
Q$359-1	Q8NE71	ABCF1_HUMAN	ABCF1	ATP-binding cassette sub-family F member 1	1.0000	4.14	3	3	3
Q$359-2	H0YGW7	H0YGW7_HUMAN	ABCF1	ATP-binding cassette sub-family F member 1 (Fragment)	1.0000	5.87	3	3	3
Q$359-3	Q8NE71-2	ABCF1_HUMAN	ABCF1	Isoform 2 of ATP-binding cassette sub-family F member 1	1.0000	4.34	3	3	3
						FGLESHAHTIQICK	3	0.9329	1
						GAVIVVSHDAR	2	0.9994	1
						STLLLLLTGK	2	0.999	1
Q$360-1	H0YH81	H0YH81_HUMAN	ATP5B	ATP synthase subunit beta (Fragment)	1.0000	7.46	2	2	6
						FTQAGSEVSALLGR	2	0.9997	3
						VALTGLTVAEYFR	2	0.9991	3
Q$361-1	H0YHR8	H0YHR8_HUMAN	NT5C2	Cytosolic purine 5'-nucleotidase (Fragment)	1.0000	8.28	1	1	3
Q$361-2	Q5JUV3	Q5JUV3_HUMAN	NT5C2	Cytosolic purine 5'-nucleotidase (Fragment)	1.0000	7.47	1	1	3
						GLVFDTLYGNLLK	2	0.9997	3
Q$362-1	H0YI43	H0YI43_HUMAN	MYL6	Myosin light polypeptide 6 (Fragment)	1.0000	37.35	2	2	3
						NKDQGTYEDYVEGLR	2	0.9995	2
						VFDKEGNGTVMGAEIR	3	0.84	1
Q$363-1	H0YJ11	H0YJ11_HUMAN	ACTN1	Alpha-actinin-1 (Fragment)	1.0000	11.59	2	2	5
Q$363-2	H0YJW3	H0YJW3_HUMAN	ACTN1	Alpha-actinin-1 (Fragment)	1.0000	9.56	2	2	5
Q$363-3	H7C5W8	H7C5W8_HUMAN	ACTN1	Alpha-actinin-1 (Fragment)	1.0000	8.08	2	2	5
						TINEVENQILTR	2	0.999	2
						VGWEQLLTTIAR	2	0.9996	3
Q$364-1	H0YJ21	H0YJ21_HUMAN	DYNC1H1	Cytoplasmic dynein 1 heavy chain 1 (Fragment)	1.0000	5.56	1	1	2
						VTFVNFTVTR	2	0.9992	2
Q$365-1	H0YMU9	H0YMU9_HUMAN	ANXA2	Annexin	1.0000	17.39	3	4	6

Q$365-2	H0YKS4	H0YKS4_HUMAN	ANXA2	Annexin (Fragment)	1.0000	22.73	3	4	6
Q$365-3	H0YMD0	H0YMD0_HUMAN	ANXA2	Annexin (Fragment)	1.0000	17.62	3	4	6
Q$365-4	H0YMM1	H0YMM1_HUMAN	ANXA2	Annexin (Fragment)	1.0000	26.85	3	4	6
Q$365-5	H0YN42	H0YN42_HUMAN	ANXA2	Annexin (Fragment)	1.0000	15.63	3	4	6
Q$365-6	P07355	ANXA2_HUMAN	ANXA2	Annexin A2	1.0000	11.8	3	4	6
Q$365-7	P07355-2	ANXA2_HUMAN	ANXA2	Isoform 2 of Annexin A2	1.0000	11.2	3	4	6
						GLGTDEDSLIEIICSR	2	0.9937	1
						GVDEVTIVNILTNR	2	0.9994	2
						GVDEVTIVNILTNR	3	0.9926	1
						TNQELQEINR	2	0.9962	2
Q$366-1	H0YLE8	H0YLE8_HUMAN	IQGAP1	Ras GTPase-activating-like protein IQGAP1	1.0000	3.41	3	3	6
						EEIQSSISGVTAAYNR	2	0.9997	2
						EQLWLANEGLITR	2	0.9995	3
						RLIVDVIR	2	0.996	1
Q$367-1	H0YLF3	H0YLF3_HUMAN	B2M	Beta-2-microglobulin form pI 5.3 (Fragment)	1.0000	18.31	1	1	3
						IEKVEHSDLSFSK	2	0.9993	3
Q$368-1	H0YN26	H0YN26_HUMAN	ANP32A	Acidic leucine-rich nuclear phosphoprotein 32 family membe	1.0000	53.67	7	9	15
Q$368-2	P39687	AN32A_HUMAN	ANP32A	Acidic leucine-rich nuclear phosphoprotein 32 family membe	1.0000	38.15	7	9	15
						CPNLTHLNLSGNK	2	0.9996	1
						CPNLTHLNLSGNK	3	0.9195	1
						ELVLDNSR	2	0.99	1
						KLELSDNR	2	0.993	1
						SLDLFNCEVTNLNDYR	2	0.9997	4
						SLDLFNCEVTNLNDYR	3	0.9929	2
						SLDLFNCEVTNLNDYRENVFK	3	0.9812	1
						SNEGKLEGLTDEFEELEFLSTINVGLTSIANLPK	3	0.9988	1
						VSGGLEVLAEKCPNLTHLNLSGNK	3	0.9939	3
Q$369-1	H3BNA2	H3BNA2_HUMAN	CORO1A	Coronin (Fragment)	1.0000	10.81	1	1	3
Q$369-2	H3BRJ0	H3BRJ0_HUMAN	CORO1A	Coronin (Fragment)	1.0000	6.43	1	1	3
Q$369-3	H3BSL1	H3BSL1_HUMAN	CORO1A	Coronin (Fragment)	1.0000	7.34	1	1	3
Q$369-4	H3BTU6	H3BTU6_HUMAN	CORO1A	Coronin (Fragment)	1.0000	6.5	1	1	3
Q$369-5	H3BU76	H3BU76_HUMAN	CORO1A	Coronin (Fragment)	1.0000	9.7	1	1	3
						VSQTTWDSGFCAVNPK	2	0.9995	3
Q$370-1	P15170	ERF3A_HUMAN	GSPT1	Eukaryotic peptide chain release factor GTP-binding subunit	1.0000	9.02	4	4	4
Q$370-2	H3BR35	H3BR35_HUMAN	GSPT1	Eukaryotic peptide chain release factor GTP-binding subunit	1.0000	9.47	4	4	4
Q$370-3	P15170-2	ERF3A_HUMAN	GSPT1	Isoform 2 of Eukaryotic peptide chain release factor GTP-bind	1.0000	7.08	4	4	4
Q$370-4	P15170-3	ERF3A_HUMAN	GSPT1	Isoform 3 of Eukaryotic peptide chain release factor GTP-bind	1.0000	7.06	4	4	4
						AYFETEKK	2	0.9461	1
						ETWYLSWALDTNQEER	2	0.9474	1
						HFTILDAPGHK	3	0.998	1
						KGEFETGFEK	2	0.9855	1
Q$371-1	H3BRG4	H3BRG4_HUMAN	UQCRC2	Cytochrome b-c1 complex subunit 2, mitochondrial	0.9844	3.88	1	1	2
Q$371-2	P22695	QCR2_HUMAN	UQCRC2	Cytochrome b-c1 complex subunit 2, mitochondrial	0.9844	3.53	1	1	2
						TIAQGNLSNTDVQAAK	2	0.9844	2
Q$372-1	H3BRY3	H3BRY3_HUMAN	CORO1A	Coronin	1.0000	12.34	4	4	9
Q$372-2	P31146	COR1A_HUMAN	CORO1A	Coronin-1A	1.0000	10.41	4	4	9
						DGGLICTSCR	2	0.9895	2
						ILTTGFSR	2	0.9611	1
						VSQTTWDSGFCAVNPK	2	0.9995	3
						YFEITSEAPFLHYL	2	0.9917	3
Q$373-1	H3BSV8	H3BSV8_HUMAN	GSPT1	Eukaryotic peptide chain release factor GTP-binding subunit	1.0000	5.53	1	1	1
						HFTILDAPGHK	3	0.998	1
Q$374-1	H3BVG3	H3BVG3_HUMAN	JMJD8	JmjC domain-containing protein 8	0.9577	12.79	1	1	1
Q$374-2	Q96S16	JMJD8_HUMAN	JMJD8	JmjC domain-containing protein 8	0.9577	8.38	1	1	1
						ADLTYAEFVQQYAFVRPVILQGLTDNSR	3	0.9269	1
Q$375-1	H7BXV5	H7BXV5_HUMAN	COL18A1	Endostatin (Fragment)	1.0000	5.39	2	2	4
Q$375-2	H7C457	H7C457_HUMAN	COL18A1	Endostatin (Fragment)	1.0000	13.21	2	2	4
						LTESYCETWR	2	0.993	1
						QAMLGQVHEVPEGWLIFVAEQEELYVR	3	0.9986	3
Q$376-1	H7BY16	H7BY16_HUMAN	NCL	Nucleolin (Fragment)	1.0000	6.08	2	2	3
						EVFEDAAEIR	2	0.9996	2
						GIAYIEFK	2	0.9995	1
Q$377-1	H7C0L5	H7C0L5_HUMAN	ITIH4	35 kDa inter-alpha-trypsin inhibitor heavy chain H4 (Fragmer	1.0000	4.31	2	2	9
						AGFSWIEVTFK	2	0.9994	4
						RLDYQEGPPGVEISCWSVEL	2	0.9995	5
Q$378-1	P47897	SYQ_HUMAN	QARS	Glutamine--tRNA ligase	1.0000	7.48	5	5	7
Q$378-2	H7C0R3	H7C0R3_HUMAN	QARS	Glutamine--tRNA ligase (Fragment)	1.0000	22.92	5	5	7
Q$378-3	P47897-2	SYQ_HUMAN	QARS	Isoform 2 of Glutamine--tRNA ligase	1.0000	7.59	5	5	7
						AMAVLESLR	2	0.9991	2
						GFHQVPFAPIVFIER	3	0.999	2
						GPSGCVESLEVTCR	2	0.9994	1
						LAWGQPVGLR	2	0.9993	1
						VIITNFPAAK	2	0.9755	1
Q$379-1	H7C1L7	H7C1L7_HUMAN	NAGK	N-acetyl-D-glucosamine kinase	1.0000	16.98	2	2	3
						SEVLLVSEDGK	2	0.9586	1
						SLGLSLSGGDQEDAGR	2	0.9996	2
Q$380-1	Q99460	PSMD1_HUMAN	PSMD1	26S proteasome non-ATPase regulatory subunit 1	0.9872	1.26	1	1	3
Q$380-2	H7C378	H7C378_HUMAN	PSMD1	26S proteasome non-ATPase regulatory subunit 1 (Fragment)	0.9872	6.25	1	1	3
Q$380-3	Q99460-2	PSMD1_HUMAN	PSMD1	Isoform 2 of 26S proteasome non-ATPase regulatory subunit	0.9872	1.3	1	1	3
						AAVESLGFILFR	2	0.9872	3
Q$381-1	H7C3G9	H7C3G9_HUMAN	NAGK	N-acetyl-D-glucosamine kinase	1.0000	6.47	2	2	2
Q$381-2	Q9UJ70	NAGK_HUMAN	NAGK	N-acetyl-D-glucosamine kinase	1.0000	6.4	2	2	2
						EGFLLALTQGR	2	0.8813	1
						SEVLLVSEDGK	2	0.9586	1
Q$382-1	O15083	ERC2_HUMAN	ERC2	ERC protein 2	0.9855	1.15	1	1	1
Q$382-2	H7C4G9	H7C4G9_HUMAN	ERC2	ERC protein 2 (Fragment)	0.9855	2.08	1	1	1
						KESELLALQTK	2	0.8485	1
Q$383-1	Q9NQR4	NIT2_HUMAN	NIT2	Omega-amidase NIT2	0.9939	8.33	2	2	2
Q$383-2	H7C579	H7C579_HUMAN	NIT2	Omega-amidase NIT2 (Fragment)	0.9939	8.68	2	2	2
						FAELAQIYAQR	2	0.9573	1
						LALIQLQISSIK	2	0.8575	1
Q$384-1	Q7KZF4	SND1_HUMAN	SND1	Staphylococcal nuclease domain-containing protein 1	0.9482	1.43	1	1	1
Q$384-2	H7C597	H7C597_HUMAN	SND1	Staphylococcal nuclease domain-containing protein 1 (Fragm	0.9482	5.63	1	1	1
						SEAVVEYVFSGSR	2	0.9482	1
Q$385-1	H7C5R1	H7C5R1_HUMAN	CP	Ceruloplasmin (Fragment)	1.0000	4.46	3	3	5
						EVGPTNADPVCLAK	2	0.9281	1
						EYTDASFTNRK	2	0.9964	2
						GAYPLSIEPIGVR	2	0.9988	2
Q$386-1	P16615-2	AT2A2_HUMAN	ATP2A2	Isoform 2 of Sarcoplasmic/endoplasmic reticulum calcium AT	1.0000	2.81	2	2	3
Q$386-2	P16615-3	AT2A2_HUMAN	ATP2A2	Isoform 3 of Sarcoplasmic/endoplasmic reticulum calcium AT	1.0000	2.8	2	2	3
Q$386-3	P16615-4	AT2A2_HUMAN	ATP2A2	Isoform 4 of Sarcoplasmic/endoplasmic reticulum calcium AT	1.0000	2.76	2	2	3
Q$386-4	P16615-5	AT2A2_HUMAN	ATP2A2	Isoform 5 of Sarcoplasmic/endoplasmic reticulum calcium AT	1.0000	2.81	2	2	3
Q$386-5	P16615	AT2A2_HUMAN	ATP2A2	Sarcoplasmic/endoplasmic reticulum calcium ATPase 2	1.0000	2.69	2	2	3
Q$386-6	H7C5W9	H7C5W9_HUMAN	ATP2A2	Sarcoplasmic/endoplasmic reticulum calcium ATPase 2 (Frag	1.0000	3	2	2	3
						IGIFGQDEDVTSK	2	0.9946	1
						VGEATETALTCLVEK	2	0.9992	2
Q$387-1	H9KV48	H9KV48_HUMAN	SERPING1	Plasma protease C1 inhibitor	1.0000	20.73	11	12	28
						DFTCVHQALK	2	0.9995	1
						FQPTLLTLPR	2	0.9458	1
						GVTSVSQIFHSPDLAIR	2	0.9997	3
						GVTSVSQIFHSPDLAIR	3	0.995	1
						LLDSLPSDTR	2	0.9988	4
						LVLLNAIYLSAK	2	0.9996	3
						TLLVFEVQQPF	2	0.9254	1
						TLLVFEVQQPFL	2	0.9916	2
						TLLVFEVQQPFLF	2	0.9859	3
						TNLESILSYPK	2	0.9995	4
						TNLESILSYPKDFTCVHQALK	3	0.9887	2
						VTTSQDMLSIMEK	2	0.9994	3
Q$388-1	H9KV70	H9KV70_HUMAN	LCN2	Neutrophil gelatinase-associated lipocalin	1.0000	33	4	4	9
Q$388-2	P80188	NGAL_HUMAN	LCN2	Neutrophil gelatinase-associated lipocalin	1.0000	33.33	4	4	9
Q$388-3	X6R8F3	X6R8F3_HUMAN	LCN2	Neutrophil gelatinase-associated lipocalin	1.0000	33	4	4	9
						SLGLPENHIVFPVPIDQCIDG	2	0.9871	1

						TFVPGCQPGEFTLGNIK	2	0.9962	2
						VPLQQNFQDNQFQGK	2	0.9996	2
						WYVVGLAGNAILR	2	0.9994	4
Q$389-1	H9KV75	H9KV75_HUMAN	ACTN1	Alpha-actinin-1	1.0000	8.39	6	6	12
Q$389-2	P12814	ACTN1_HUMAN	ACTN1	Alpha-actinin-1	1.0000	7.74	6	6	12
Q$389-3	P12814-2	ACTN1_HUMAN	ACTN1	Isoform 2 of Alpha-actinin-1	1.0000	7.78	6	6	12
Q$389-4	P12814-3	ACTN1_HUMAN	ACTN1	Isoform 3 of Alpha-actinin-1	1.0000	7.55	6	6	12
Q$389-5	P12814-4	ACTN1_HUMAN	ACTN1	Isoform 4 of Alpha-actinin-1	1.0000	7.42	6	6	12
						EGLLLWCQR	2	0.9195	1
						FAIQDISVEETSAK	2	0.9893	1
						LAILGIHNEVSK	2	0.9787	2
						LASDLLEWIR	2	0.9974	3
						TINEVENQILTR	2	0.999	2
						VGWEQLLTTIAR	2	0.9996	3
Q$390-1	H9KVB3	H9KVB3_HUMAN	OTOG	Otogelin	0.9980	0.74	2	2	2
Q$390-2	Q6ZRI0	OTOG_HUMAN	OTOG	Otogelin	0.9980	0.72	2	2	2
						DLAACCG	2	0.9532	1
						VGGSVVPSLEGCCR	3	0.9581	1
Q$391-1	I3L168	I3L168_HUMAN	MYO1C	Unconventional myosin-Ic (Fragment)	1.0000	10.45	1	1	2
Q$391-2	I3L204	I3L204_HUMAN	MYO1C	Unconventional myosin-Ic (Fragment)	1.0000	5.24	1	1	2
Q$391-3	I3L3F5	I3L3F5_HUMAN	MYO1C	Unconventional myosin-Ic (Fragment)	1.0000	10.22	1	1	2
Q$391-4	I3L3Y6	I3L3Y6_HUMAN	MYO1C	Unconventional myosin-Ic (Fragment)	1.0000	8.24	1	1	2
Q$391-5	I3L4D4	I3L4D4_HUMAN	MYO1C	Unconventional myosin-Ic (Fragment)	1.0000	8.54	1	1	2
						LLQFYAETCPAPER	2	0.9986	2
Q$392-1	I3L3Y0	I3L3Y0_HUMAN	KIAA0753	Uncharacterized protein KIAA0753	0.9610	2.82	1	1	1
Q$392-2	Q2KHM9	K0753_HUMAN	KIAA0753	Uncharacterized protein KIAA0753	0.9610	0.72	1	1	1
Q$392-3	I3L2A7	I3L2A7_HUMAN	KIAA0753	Uncharacterized protein KIAA0753 (Fragment)	0.9610	10.14	1	1	1
						SLLEVQR	2	0.961	1
Q$393-1	I3L2G1	I3L2G1_HUMAN	NSF	Vesicle-fusing ATPase (Fragment)	0.9961	12.5	2	2	2
Q$393-2	K7EQD6	K7EQD6_HUMAN	NSF	Vesicle-fusing ATPase (Fragment)	0.9961	9.44	2	2	2
						KAPPQGR	2	0.961	1
						LLDYVPIGPR	2	0.8999	1
Q$394-1	Q96JN8-2	NEUL4_HUMAN	NEURL4	Isoform 2 of Neuralized-like protein 4	0.9994	3.91	3	4	4
Q$394-2	I3L2W2	I3L2W2_HUMAN	NEURL4	Neuralized-like protein 4	0.9994	3.97	3	4	4
Q$394-3	Q96JN8	NEUL4_HUMAN	NEURL4	Neuralized-like protein 4	0.9994	3.91	3	4	4
						AAGSGGSGGSGGGPGPGPGGGGGPSGSGSGPGSNGGLGSGGELHPR	3	0.9851	1
						AAGSGGSGGSGGGPGPGPGGGGGPSGSGSGPGSNGGLGSGGELHPR	4	0.9046	1
						AAGSGGSGGSGGGPGPGPGGGGGPSGSGSGPGSNGGLGSGGELHPRTGR	3	0.9258	1
						VGVERTVAGELR	3	0.9128	1
Q$395-1	I3L312	I3L312_HUMAN	P4HB	Protein disulfide-isomerase (Fragment)	1.0000	34.13	5	6	10
						DAPEEEDHVLVLR	2	0.9576	2
						KSNFAEALAAHK	3	0.9852	1
						QFLQAAEAIDDIPFGITSNSDVFSK	3	0.9986	1
						THILLFLPK	2	0.9987	2
						THILLFLPK	3	0.9996	2
						YQLDKDGVVLFK	3	0.9983	2
Q$396-1	I3L3B0	I3L3B0_HUMAN	C1QBP	Complement component 1 Q subcomponent-binding protein,	0.9927	16.85	1	1	4
Q$396-2	Q07021	C1QBP_HUMAN	C1QBP	Complement component 1 Q subcomponent-binding protein,	0.9927	10.64	1	1	4
Q$396-3	I3L3Q7	I3L3Q7_HUMAN	C1QBP	Complement component 1 Q subcomponent-binding protein,	0.9927	16.95	1	1	4
						GVDNTFADELVELSTALEHQEYITFLEDLK	3	0.9927	4
Q$397-1	I3L3P5	I3L3P5_HUMAN	P4HB	Protein disulfide-isomerase (Fragment)	1.0000	10.26	1	1	2
						VDATEESDLAQQYGVR	2	0.9997	2
Q$398-1	I3L501	I3L501_HUMAN	MYO1C	Unconventional myosin-Ic (Fragment)	1.0000	14.29	2	2	3
						LLQFYAETCPAPER	2	0.9986	2
						VTMESALTAR	2	0.9769	1
Q$399-1	J3KNB4	J3KNB4_HUMAN	CAMP	Cathelicidin antimicrobial peptide	0.9952	5.2	1	1	3
Q$399-2	P49913	CAMP_HUMAN	CAMP	Cathelicidin antimicrobial peptide	0.9952	5.29	1	1	3
						FALLGDFFR	2	0.993	3
Q$400-1	J3KNC0	J3KNC0_HUMAN	GTF2A1	Transcription initiation factor IIA beta chain	0.9692	18.64	1	1	2
Q$400-2	P52655	TF2AA_HUMAN	GTF2A1	Transcription initiation factor IIA subunit 1	0.9692	2.93	1	1	2
						SVIEDVINDVR	2	0.9692	2
Q$401-1	P68402	PA1B2_HUMAN	PAFAH1B2	Platelet-activating factor acetylhydrolase IB subunit beta	0.9488	3.93	1	1	1
Q$401-2	J3KNE3	J3KNE3_HUMAN	PAFAH1B2	Platelet-activating factor acetylhydrolase IB subunit beta (Fra	0.9488	5.14	1	1	1
						IIVLGLLPR	2	0.9488	1
Q$402-1	J3KP07	J3KP07_HUMAN	GAS6	Growth arrest-specific protein 6	1.0000	25.52	14	15	27
Q$402-2	Q14393	GAS6_HUMAN	GAS6	Growth arrest-specific protein 6	1.0000	25.52	14	15	27
Q$402-3	Q14393-2	GAS6_HUMAN	GAS6	Isoform 2 of Growth arrest-specific protein 6	1.0000	27.14	14	15	27
Q$402-4	Q14393-3	GAS6_HUMAN	GAS6	Isoform 3 of Growth arrest-specific protein 6	1.0000	29.49	14	15	27
						AGGLPDVPVTSAPVTAFYR	2	0.9212	1
						DGEATLEVDGTR	2	0.9992	3
						DLVQPINPR	2	0.9996	1
						DVDECLQGR	2	0.9996	1
						GQSEVSAAQLQER	2	0.9997	1
						GSFYPGSGFAFYSLDYMR	2	0.9997	3
						HSDITAHSCPPVEPAAA	2	0.9852	1
						IAVAGDLFQPER	2	0.9997	2
						LLDLDEAAYK	2	0.996	2
						MQCFSVTER	2	0.9756	1
						QLVVLAVEHTALALMEIK	3	0.9991	2
						SPVLTFAGGLPDVPVTSAPVTAFYR	2	0.999	2
						SPVLTFAGGLPDVPVTSAPVTAFYR	3	0.9978	2
						TCQDIDECADSEACGEAR	2	0.9997	3
						VCDGQEHVVTVSLR	2	0.9898	2
Q$403-1	J3KPC0	J3KPC0_HUMAN	ZFAND4	AN1-type zinc finger protein 4	0.9880	4.44	2	2	2
Q$403-2	Q5VVY6	Q5VVY6_HUMAN	ZFAND4	AN1-type zinc finger protein 4	0.9880	4.76	2	2	2
Q$403-3	Q86XD8	ZFAN4_HUMAN	ZFAND4	AN1-type zinc finger protein 4	0.9880	3.99	2	2	2
						DITEMTNK	2	0.9002	1
						LQPSSSQSLDVQNITDSSFSR	2	0.8796	1
Q$404-1	J3KQ32	J3KQ32_HUMAN	OLA1	Obg-like ATPase 1	1.0000	6.73	2	3	5
Q$404-2	Q9NTK5	OLA1_HUMAN	OLA1	Obg-like ATPase 1	1.0000	7.07	2	3	5
						IPAFLNVVDIAGLVK	2	0.9997	3
						IPAFLNVVDIAGLVK	3	0.9488	1
						NYIVEDGDIIFFK	2	0.9898	1
Q$405-1	J3KR24	J3KR24_HUMAN	IARS	Isoleucine--tRNA ligase, cytoplasmic	1.0000	1.56	2	2	2
						VCMDFNIIR	2	0.992	1
						YIIEELNVR	2	0.9564	1
Q$406-1	J3QSE0	J3QSE0_HUMAN	PSMC5	26S protease regulatory subunit 8	1.0000	18.93	2	2	4
Q$406-2	J3KRP2	J3KRP2_HUMAN	PSMC5	26S protease regulatory subunit 8 (Fragment)	1.0000	13.28	2	2	4
Q$406-3	J3QLH6	J3QLH6_HUMAN	PSMC5	26S protease regulatory subunit 8 (Fragment)	1.0000	14.88	2	2	4
Q$406-4	J3QQM1	J3QQM1_HUMAN	PSMC5	26S protease regulatory subunit 8 (Fragment)	1.0000	12.17	2	2	4
Q$406-5	J3QSA9	J3QSA9_HUMAN	PSMC5	26S protease regulatory subunit 8 (Fragment)	1.0000	12.45	2	2	4
						IEELQLIVNDK	2	0.9964	2
						LLREELQLLQEQGSYVGEVVR	3	0.9983	2
Q$407-1	Q7Z4W1	DCXR_HUMAN	DCXR	L-xylulose reductase	0.9705	4.92	1	1	1
Q$407-2	J3KS22	J3KS22_HUMAN	DCXR	L-xylulose reductase (Fragment)	0.9705	5.38	1	1	1
Q$407-3	J3QS36	J3QS36_HUMAN	DCXR	L-xylulose reductase (Fragment)	0.9705	5.33	1	1	1
						GTVQALHATGAR	2	0.9705	1
Q$408-1	J3QQU6	J3QQU6_HUMAN	SECTM1	Secreted and transmembrane protein 1	1.0000	35.57	4	7	16
Q$408-2	Q8WVN6	SCTM1_HUMAN	SECTM1	Secreted and transmembrane protein 1	1.0000	21.37	4	7	16
Q$408-3	J3KSN0	J3KSN0_HUMAN	SECTM1	Secreted and transmembrane protein 1 (Fragment)	1.0000	36.3	4	7	16
Q$408-4	J3KTR4	J3KTR4_HUMAN	SECTM1	Secreted and transmembrane protein 1 (Fragment)	1.0000	29.78	4	7	16
Q$408-5	J3QKK2	J3QKK2_HUMAN	SECTM1	Secreted and transmembrane protein 1 (Fragment)	1.0000	31.18	4	7	16
						AHGQESAIFNEVAPGYFSR	2	0.9997	2
						AHGQESAIFNEVAPGYFSR	3	0.9996	3
						DGWQLQVQGGVAQLVIK	2	0.9994	4
						DGWQLQVQGGVAQLVIK	3	0.9992	3
						DSHAGLYMWHLVGHQR	2	0.9996	1
						DSHAGLYMWHLVGHQR	3	0.9978	2
						RAHGQESAIFNEVAPGYFSR	3	0.8699	1
Q$409-1	J3KSR8	J3KSR8_HUMAN	SRSF1	Serine/arginine-rich-splicing factor 1 (Fragment)	1.0000	15.38	2	2	4
						EAGDVCYADVYR	2	0.9997	2
						SHEGETAYIR	2	0.9996	2
Q$410-1	Q07955	SRSF1_HUMAN	SRSF1	Serine/arginine-rich splicing factor 1	1.0000	37.5	10	10	24

Q$410-2	J3KTL2	J3KTL2_HUMAN	SRSF1	Serine/arginine-rich-splicing factor 1	1.0000	36.76	10	10	24
						DGTGVVEFVR	2	0.9724	1
						EAGDVCYADVYR	2	0.9997	2
						GGPPFAFVEFEDPR	2	0.9995	5
						GGPPFAFVEFEDPRDAEDAVYGR	3	0.9792	1
						IYVGNLPPDIR	2	0.9993	9
						KEDMTYAVR	2	0.9498	1
						LRVEFPR	2	0.8397	1
						RGGPPFAFVEFEDPR	3	0.9958	1
						SHEGETAYIR	2	0.9996	2
						TKDIEDVFYK	2	0.9627	1
Q$411-1	J3QKT2	J3QKT2_HUMAN	CNDP2	Cytosolic non-specific dipeptidase (Fragment)	1.0000	4.02	1	1	1
Q$411-2	J3QRH4	J3QRH4_HUMAN	CNDP2	Cytosolic non-specific dipeptidase (Fragment)	1.0000	4.9	1	1	1
						TGQEIPVNVR	2	0.9962	1
Q$412-1	P00738	HPT_HUMAN	HP	Haptoglobin	1.0000	19.7	5	5	10
Q$412-2	J3QLC9	J3QLC9_HUMAN	HP	Haptoglobin (Fragment)	1.0000	21.92	5	5	10
Q$412-3	P00738-2	HPT_HUMAN	HP	Isoform 2 of Haptoglobin	1.0000	23.05	5	5	10
						IAPTLTLYVGK	2	0.909	1
						SCAVAEYGVYVK	2	0.9997	4
						TEGDGVYTLNNEK	2	0.8743	1
						VTSIQDWVQK	2	0.9993	2
						YQEDTCYGDAGSAFAVHDLEEDTWYATGILSFDK	3	0.9973	2
Q$413-1	J3QLP3	J3QLP3_HUMAN	VWA1	von Willebrand factor A domain-containing protein 1 (Fragme	1.0000	31.14	3	3	3
						EQLFAEASGARPGVPK	2	0.9951	1
						GNFLELSAAASAPAEK	2	0.9977	1
						VREFVGQLVAPLPLGTGALR	3	0.9076	1
Q$414-1	Q14974	IMB1_HUMAN	KPNB1	Importin subunit beta-1	0.9013	1.71	1	1	2
Q$414-2	J3QR48	J3QR48_HUMAN	KPNB1	Importin subunit beta-1 (Fragment)	0.9013	10.14	1	1	2
						AAVENLPTFLVELSR	2	0.9013	2
Q$415-1	J3QRR0	J3QRR0_HUMAN	VWA1	von Willebrand factor A domain-containing protein 1 (Fragme	0.9995	13.51	2	2	2
						EFVGQLVAPLPLGTGALR	2	0.9926	1
						VREFVGQLVAPLPLGTGALR	3	0.9076	1
Q$416-1	J3QRS3	J3QRS3_HUMAN	MYL12A	Myosin regulatory light chain 12A	1.0000	16.95	2	2	3
Q$416-2	P19105	ML12A_HUMAN	MYL12A	Myosin regulatory light chain 12A	1.0000	17.54	2	2	3
Q$416-3	O14950	ML12B_HUMAN	MYL12B	Myosin regulatory light chain 12B	1.0000	17.44	2	2	3
						GNFNYIEFTR	2	0.9944	2
						NAFACFDEEATGTIQEDYLR	2	0.993	1
Q$417-1	O00231	PSD11_HUMAN	PSMD11	26S proteasome non-ATPase regulatory subunit 11	0.9054	3.08	1	1	2
Q$417-2	J3QRY4	J3QRY4_HUMAN	PSMD11	26S proteasome non-ATPase regulatory subunit 11 (Fragmen	0.9054	6.95	1	1	2
Q$417-3	O00231-2	PSD11_HUMAN	PSMD11	Isoform 2 of 26S proteasome non-ATPase regulatory subunit	0.9054	3.07	1	1	2
						EQSILELGSLLAK	2	0.9054	2
Q$418-1	J3QST3	J3QST3_HUMAN	KRT3	Keratin, type II cytoskeletal 3	1.0000	2.38	1	1	3
Q$418-2	P12035	K2C3_HUMAN	KRT3	Keratin, type II cytoskeletal 3	1.0000	2.39	1	1	3
						DVDSAYMNKVELQAK	2	0.9835	3
Q$419-1	Q05707	COEA1_HUMAN	COL14A1	Collagen alpha-1(XIV) chain	0.9788	1.39	2	2	2
Q$419-2	J3QT83	J3QT83_HUMAN	COL14A1	Collagen alpha-1(XIV) chain (Fragment)	0.9788	2.97	2	2	2
Q$419-3	Q05707-2	COEA1_HUMAN	COL14A1	Isoform 2 of Collagen alpha-1(XIV) chain	0.9788	1.4	2	2	2
Q$419-4	Q05707-3	COEA1_HUMAN	COL14A1	Isoform 3 of Collagen alpha-1(XIV) chain	0.9788	1.47	2	2	2
						NLVVGDETTSSLRV	2	0.8638	1
						QESTVGGGTTR	2	0.8441	1
Q$420-1	K7EIV0	K7EIV0_HUMAN	CAPNS1	Calpain small subunit 1 (Fragment)	1.0000	15.82	2	2	3
						ILGGVISAISEAAAQYNPEPPPPR	3	0.9975	2
						LGFEEFK	2	0.9897	1
Q$421-1	K7EJ44	K7EJ44_HUMAN	PFN1	Profilin 1, isoform CRA_b	1.0000	13.46	1	1	5
						DSLLQDGEFSMDLR	2	0.9995	5
Q$422-1	K7EJ74	K7EJ74_HUMAN	EFTUD2	116 kDa U5 small nuclear ribonucleoprotein component (Fra	1.0000	6.72	1	1	1
						YDWDLLAAR	2	0.9995	1
Q$423-1	K7EJD3	K7EJD3_HUMAN	LGALS3BP	Galectin-3-binding protein	1.0000	10.24	1	2	6
Q$423-2	K7EN99	K7EN99_HUMAN	LGALS3BP	Galectin-3-binding protein	1.0000	18.58	1	2	6
Q$423-3	K7ERZ6	K7ERZ6_HUMAN	LGALS3BP	Galectin-3-binding protein	1.0000	16.03	1	2	6
Q$423-4	K7ES75	K7ES75_HUMAN	LGALS3BP	Galectin-3-binding protein (Fragment)	1.0000	23.86	1	2	6
						GQWGTVCDNLWDLTDASVVCR	2	0.9997	4
						GQWGTVCDNLWDLTDASVVCR	3	0.9969	2
Q$424-1	K7EJP1	K7EJP1_HUMAN	ATP5A1	ATP synthase subunit alpha, mitochondrial (Fragment)	1.0000	21.53	2	2	5
Q$424-2	K7EK77	K7EK77_HUMAN	ATP5A1	ATP synthase subunit alpha, mitochondrial (Fragment)	1.0000	14.98	2	2	5
Q$424-3	K7EQH4	K7EQH4_HUMAN	ATP5A1	ATP synthase subunit alpha, mitochondrial (Fragment)	1.0000	27.93	2	2	5
Q$424-4	K7ERX7	K7ERX7_HUMAN	ATP5A1	ATP synthase subunit alpha, mitochondrial (Fragment)	1.0000	15.12	2	2	5
						ILGADTSVDLEETGR	2	0.9997	2
						TGAIVDVPVGEELLGR	2	0.9996	3
Q$425-1	K7EKS7	K7EKS7_HUMAN	RPL22	60S ribosomal protein L22	0.9608	24.53	1	1	1
Q$425-2	K7ERI7	K7ERI7_HUMAN	RPL22	60S ribosomal protein L22	0.9608	13.68	1	1	1
Q$425-3	P35268	RL22_HUMAN	RPL22	60S ribosomal protein L22	0.9608	10.16	1	1	1
Q$425-4	K7EJT5	K7EJT5_HUMAN	RPL22	60S ribosomal protein L22 (Fragment)	0.9608	27.66	1	1	1
Q$425-5	K7ELC4	K7ELC4_HUMAN	RPL22	60S ribosomal protein L22 (Fragment)	0.9608	16.46	1	1	1
Q$425-6	K7EMH1	K7EMH1_HUMAN	RPL22	60S ribosomal protein L22 (Fragment)	0.9608	14.61	1	1	1
Q$425-7	K7EP65	K7EP65_HUMAN	RPL22	60S ribosomal protein L22 (Fragment)	0.9608	27.08	1	1	1
						AGNLGGGVVTIER	2	0.9608	1
Q$426-1	K7EKD8	K7EKD8_HUMAN	CAPNS1	Calpain small subunit 1	0.9889	21.43	1	1	1
Q$426-2	K7ES78	K7ES78_HUMAN	CAPNS1	Calpain small subunit 1 (Fragment)	0.9889	70.83	1	1	1
						GGGGGGGGGGGLGGGLGNVLGGLISGAGGGGGGGGGGGGGGGGGGGTAMR	4	0.9478	1
Q$427-1	Q92692-2	PVRL2_HUMAN	PVRL2	Isoform Alpha of Nectin-2	0.9607	2.51	1	1	2
Q$427-2	Q92692	PVRL2_HUMAN	PVRL2	Nectin-2	0.9607	2.23	1	1	2
Q$427-3	K7EKE8	K7EKE8_HUMAN	PVRL2	Nectin-2 (Fragment)	0.9607	5.71	1	1	2
						ISWLSSLDWEAK	2	0.9607	2
Q$428-1	K7EKF6	K7EKF6_HUMAN	ANGPTL6	Angiopoietin-related protein 6	1.0000	6.05	2	2	2
Q$428-2	Q8NI99	ANGL6_HUMAN	ANGPTL6	Angiopoietin-related protein 6	1.0000	5.53	2	2	2
						GDHELLVLLEDWGGR	3	0.9691	1
						YQDGVYWAEFR	2	0.9353	1
Q$429-1	K7EKI8	K7EKI8_HUMAN	PPL	Periplakin	1.0000	2.05	3	3	4
Q$429-2	O60437	PEPL_HUMAN	PPL	Periplakin	1.0000	2.05	3	3	4
						LQNLEFALNLLR	2	0.9996	2
						NQLLQEELEALQLQLR	3	0.9966	1
						YQIELLLR	2	0.9946	1
Q$430-1	P26599-2	PTBP1_HUMAN	PTBP1	Isoform 2 of Polypyrimidine tract-binding protein 1	0.9995	5.09	2	2	3
Q$430-2	P26599-3	PTBP1_HUMAN	PTBP1	Isoform 3 of Polypyrimidine tract-binding protein 1	0.9995	5.03	2	2	3
Q$430-3	P26599	PTBP1_HUMAN	PTBP1	Polypyrimidine tract-binding protein 1	0.9995	5.27	2	2	3
Q$430-4	K7EKJ7	K7EKJ7_HUMAN	PTBP1	Polypyrimidine tract-binding protein 1 (Fragment)	0.9995	11.86	2	2	3
Q$430-5	K7ELW5	K7ELW5_HUMAN	PTBP1	Polypyrimidine tract-binding protein 1 (Fragment)	0.9995	12.02	2	2	3
Q$430-6	K7ES59	K7ES59_HUMAN	PTBP1	Polypyrimidine tract-binding protein 1 (Fragment)	0.9995	11.07	2	2	3
						KLPIDVTEGEVISLGLPFGK	3	0.9876	2
						VTNILMLK	2	0.9582	1
Q$431-1	K7EKQ5	K7EKQ5_HUMAN	LGALS3BP	Galectin-3-binding protein	1.0000	16.67	2	3	8
Q$431-2	K7EP36	K7EP36_HUMAN	LGALS3BP	Galectin-3-binding protein (Fragment)	1.0000	13.51	2	3	8
Q$431-3	K7ESM3	K7ESM3_HUMAN	LGALS3BP	Galectin-3-binding protein (Fragment)	1.0000	16.75	2	3	8
						ELSEALGQIFDSQR	2	0.999	2
						GQWGTVCDNLWDLTDASVVCR	2	0.9997	4
						GQWGTVCDNLWDLTDASVVCR	3	0.9969	2
Q$432-1	K7EL21	K7EL21_HUMAN	CAPS	Calcyphosin	1.0000	26.18	7	7	11
Q$432-2	Q13938	CAYP1_HUMAN	CAPS	Calcyphosin	1.0000	38.1	7	7	11
						ALRPPMSQAR	2	0.9782	1
						EAVIAAAFAK	2	0.9987	1
						LGLVLDQAEAEGVCR	2	0.9997	2
						NGSGTLDLEEFLR	2	0.9993	3
						SGDGVVTVDDLR	2	0.9981	1
						SGEWTEDEVLR	2	0.9977	2
						SGEWTEDEVLRR	2	0.9021	1
Q$433-1	K7ELJ7	K7ELJ7_HUMAN	CAPNS1	Calpain small subunit 1	1.0000	29.5	3	3	4
Q$433-2	P04632	CPNS1_HUMAN	CAPNS1	Calpain small subunit 1	1.0000	30.6	3	3	4
						GGGGGGGGGGGLGGGLGNVLGGLISGAGGGGGGGGGGGGGGGGGGGTAMR	4	0.9478	1
						ILGGVISAISEAAAQYNPEPPPPR	3	0.9975	2
						LGFEEFK	2	0.9897	1
Q$434-1	K7ELL7	K7ELL7_HUMAN	PRKCSH	Glucosidase 2 subunit beta	1.0000	4.11	2	2	2
Q$434-2	P14314	GLU2B_HUMAN	PRKCSH	Glucosidase 2 subunit beta	1.0000	4.17	2	2	2

Q$434-3	P14314-2	GLU2B_HUMAN	PRKCSH	Isoform 2 of Glucosidase 2 subunit beta	1.0000	4.19	2	2	2
						ETMVTSTTEPSR	2	0.9994	1
						SLKDMEESIR	2	0.9091	1
Q$435-1	K7EM20	K7EM20_HUMAN	YWHAE	14-3-3 protein epsilon (Fragment)	1.0000	11.3	1	1	2
						SVFYYEILNSPDR	2	0.9476	2
Q$436-1	K7EM49	K7EM49_HUMAN	PGD	6-phosphogluconate dehydrogenase, decarboxylating (Fragm	1.0000	8.29	1	1	2
Q$436-2	K7EMN2	K7EMN2_HUMAN	PGD	6-phosphogluconate dehydrogenase, decarboxylating (Fragm	1.0000	10.63	1	1	2
Q$436-3	K7EPF6	K7EPF6_HUMAN	PGD	6-phosphogluconate dehydrogenase, decarboxylating (Fragm	1.0000	6.64	1	1	2
						GILFVGSGVSGGEEGAR	2	0.9993	2
Q$437-1	K7EM73	K7EM73_HUMAN	CAPNS1	Calpain small subunit 1 (Fragment)	1.0000	46.01	2	2	3
						GGGGGGGGGGGLGGGLGNVLGGLISGAGGGGGGGGGGGGGGGGGGTAMR	4	0.9478	1
						ILGGVISAISEAAAQYNPEPPPPR	3	0.9975	2
Q$438-1	P26368-2	U2AF2_HUMAN	U2AF2	Isoform 2 of Splicing factor U2AF 65 kDa subunit	1.0000	15.71	5	5	6
Q$438-2	K7ENG2	K7ENG2_HUMAN	U2AF2	Splicing factor U2AF 65 kDa subunit	1.0000	24.1	5	5	6
Q$438-3	P26368	U2AF2_HUMAN	U2AF2	Splicing factor U2AF 65 kDa subunit	1.0000	15.58	5	5	6
						ELLTSFGPLK	2	0.9874	1
						IFVEFTSVFDCQK	2	0.9195	1
						LGGLTQAPGNPVLAVQINQDKNFAFLEFR	3	0.9908	2
						NFAFLEFR	2	0.9898	1
						SVDETTQAMAFDGIIFQGQSLK	2	0.9988	1
Q$439-1	Q15029	U5S1_HUMAN	EFTUD2	116 kDa U5 small nuclear ribonucleoprotein component	1.0000	2.67	2	2	2
Q$439-2	K7EP67	K7EP67_HUMAN	EFTUD2	116 kDa U5 small nuclear ribonucleoprotein component (Fra	1.0000	11.66	2	2	2
Q$439-3	Q15029-2	U5S1_HUMAN	EFTUD2	Isoform 2 of 116 kDa U5 small nuclear ribonucleoprotein cor	1.0000	2.77	2	2	2
Q$439-4	Q15029-3	U5S1_HUMAN	EFTUD2	Isoform 3 of 116 kDa U5 small nuclear ribonucleoprotein cor	1.0000	2.7	2	2	2
						GLAEDIENEVVQITWNR	2	0.9986	1
						YDWDLLAAR	2	0.9995	1
Q$440-1	K7EQ48	K7EQ48_HUMAN	GPI	Glucose-6-phosphate isomerase	1.0000	15.61	6	8	16
Q$440-2	P06744	G6PI_HUMAN	GPI	Glucose-6-phosphate isomerase	1.0000	13.26	6	8	16
Q$440-3	P06744-2	G6PI_HUMAN	GPI	Isoform 2 of Glucose-6-phosphate isomerase	1.0000	13.01	6	8	16
						HFVALSTNTTK	2	0.9986	2
						NLVTEDVMR	2	0.9912	1
						TFTTQETITNAETAK	2	0.9997	2
						TFTTQETITNAETAKEWFLQAAK	3	0.8798	1
						TLAQLNPESSLFIIASK	2	0.9997	3
						TLAQLNPESSLFIIASK	3	0.9991	3
						VWYVSNIDGTHIAK	2	0.9985	2
						VWYVSNIDGTHIAK	3	0.9942	2
Q$441-1	K7EQ71	K7EQ71_HUMAN	PPL	Periplakin (Fragment)	1.0000	2.51	2	2	3
						LQNLEFALNLLR	2	0.9996	2
						NQLLQEELEALQLQLR	3	0.9966	1
Q$442-1	P13646-3	K1C13_HUMAN	KRT13	Isoform 3 of Keratin, type I cytoskeletal 13	1.0000	47.14	16	18	32
Q$442-2	K7ERE3	K7ERE3_HUMAN	KRT13	Keratin, type I cytoskeletal 13	1.0000	47.71	16	18	32
Q$442-3	P13646	K1C13_HUMAN	KRT13	Keratin, type I cytoskeletal 13	1.0000	43.23	16	18	32
						AGLENTVAETECR	2	0.9991	2
						ALEEANADLEVK	2	0.9994	2
						EVSTNTAMIQTSK	2	0.9956	1
						LASYLEK	2	0.974	1
						LKYENELALR	2	0.9829	1
						LKYENELALR	3	0.9904	1
						LQSSSASYGGGFGGGSCQLGGGR	2	0.9997	2
						LTMQNLNDR	2	0.98	1
						QSVEADINGLRR	2	0.9455	1
						SEMECQNQEYK	2	0.9989	2
						SLLEGQDAK	2	0.9793	1
						TDLEMQIESLNEELAYMK	2	0.9988	1
						TIEELRDK	2	0.9766	1
						TLQGLEIELQSQLSMK	2	0.9997	2
						TLQGLEIELQSQLSMK	3	0.9996	4
						VILEIDNAR	2	0.998	4
						VLDELTLSK	2	0.9989	2
						YALQLQQIQGLISSIEAQLSELR	3	0.9996	3
Q$443-1	P55083-2	MFAP4_HUMAN	MFAP4	Isoform 2 of Microfibril-associated glycoprotein 4	1.0000	15.77	2	2	7
Q$443-2	K7ES70	K7ES70_HUMAN	MFAP4	Microfibril-associated glycoprotein 4	1.0000	15.71	2	2	7
Q$443-3	P55083	MFAP4_HUMAN	MFAP4	Microfibril-associated glycoprotein 4	1.0000	17.25	2	2	7
						ADGEYWLGLQNMHLLTLK	3	0.9994	5
						FSTFDRDQDLFVQNCAALSSGAFWFR	3	0.9974	2
Q$444-1	K7ESA0	K7ESA0_HUMAN	ATP5A1	ATP synthase subunit alpha, mitochondrial	1.0000	19.48	1	1	2
Q$444-2	P25705-3	ATPA_HUMAN	ATP5A1	Isoform 3 of ATP synthase subunit alpha, mitochondrial	1.0000	2.82	1	1	2
						ILGADTSVDLEETGR	2	0.9997	2
Q$445-1	M0QX47	M0QX47_HUMAN	GMFG	Glia maturation factor gamma	0.9610	17.81	1	1	4
Q$445-2	M0QYG8	M0QYG8_HUMAN	GMFG	Glia maturation factor gamma	0.9610	13.98	1	1	4
Q$445-3	M0R0C1	M0R0C1_HUMAN	GMFG	Glia maturation factor gamma	0.9610	11.93	1	1	4
Q$445-4	M0R1D2	M0R1D2_HUMAN	GMFG	Glia maturation factor gamma	0.9610	12.87	1	1	4
Q$445-5	O60234	GMFG_HUMAN	GMFG	Glia maturation factor gamma	0.9610	9.15	1	1	4
						TTDDLTEAWLQEK	2	0.961	4
Q$446-1	M0QYT0	M0QYT0_HUMAN		Uncharacterized protein (Fragment)	0.9794	4.05	1	1	3
Q$446-2	M0R076	M0R076_HUMAN		Uncharacterized protein (Fragment)	0.9794	11.4	1	1	3
Q$446-3	P14866	HNRPL_HUMAN	HNRNPL	Heterogeneous nuclear ribonucleoprotein L	0.9794	2.21	1	1	3
Q$446-4	M0QXS5	M0QXS5_HUMAN	HNRNPL	Heterogeneous nuclear ribonucleoprotein L (Fragment)	0.9794	2.45	1	1	3
Q$446-5	P14866-2	HNRPL_HUMAN	HNRNPL	Isoform 2 of Heterogeneous nuclear ribonucleoprotein L	0.9794	2.85	1	1	3
						VFNVFCLYGNVEK	2	0.9794	3
Q$447-1	Q13985	Q13985_HUMAN	CEA	Carcinoembryonic antigen	1.0000	19.4	1	1	2
						EVLLLAHNLPQNR	2	0.9993	2
Q$448-1	M0QZL1	M0QZL1_HUMAN	BLVRB	Flavin reductase (NADPH)	1.0000	9.74	1	1	3
Q$448-2	M0R192	M0R192_HUMAN	BLVRB	Flavin reductase (NADPH)	1.0000	9.2	1	1	3
						TVAGQDAVIVLLGTR	2	0.9996	3
Q$449-1	M0QZN2	M0QZN2_HUMAN	RPS5	40S ribosomal protein S5	0.9719	11.19	1	1	3
Q$449-2	M0R0R2	M0R0R2_HUMAN	RPS5	40S ribosomal protein S5	0.9719	6.67	1	1	3
Q$449-3	P46782	RS5_HUMAN	RPS5	40S ribosomal protein S5	0.9719	7.35	1	1	3
Q$449-4	M0R0F0	M0R0F0_HUMAN	RPS5	40S ribosomal protein S5 (Fragment)	0.9719	7.5	1	1	3
						TIAECLADELINAAK	2	0.9719	3
Q$450-1	P52272	HNRPM_HUMAN	HNRNPM	Heterogeneous nuclear ribonucleoprotein M	0.9575	2.19	1	1	1
Q$450-2	M0R019	M0R019_HUMAN	HNRNPM	Heterogeneous nuclear ribonucleoprotein M (Fragment)	0.9575	4.55	1	1	1
Q$450-3	M0R0N3	M0R0N3_HUMAN	HNRNPM	Heterogeneous nuclear ribonucleoprotein M (Fragment)	0.9575	5.82	1	1	1
Q$450-4	M0R2T0	M0R2T0_HUMAN	HNRNPM	Heterogeneous nuclear ribonucleoprotein M (Fragment)	0.9575	4.48	1	1	1
Q$450-5	P52272-2	HNRPM_HUMAN	HNRNPM	Isoform 2 of Heterogeneous nuclear ribonucleoprotein M	0.9575	2.32	1	1	1
						VGEVTYVELLMDAEGK	2	0.9575	1
Q$451-1	M0R0U3	M0R0U3_HUMAN	PGLS	6-phosphogluconolactonase	1.0000	18.99	3	3	8
						AACCLAGAR	2	0.999	1
						ELPAAVAPAGPASLAR	2	0.9994	4
						WTLGFCDER	2	0.9992	3
Q$452-1	M0R0Y2	M0R0Y2_HUMAN	NAPA	Alpha-soluble NSF attachment protein	1.0000	10.94	2	2	4
Q$452-2	P54920	SNAA_HUMAN	NAPA	Alpha-soluble NSF attachment protein	1.0000	9.49	2	2	4
Q$452-3	M0R2M1	M0R2M1_HUMAN	NAPA	Alpha-soluble NSF attachment protein (Fragment)	1.0000	10.45	2	2	4
						NSQSFFSGLFGGSSK	2	0.9996	2
						VAGYAALLEQYQK	2	0.9996	2
Q$453-1	O95336	6PGL_HUMAN	PGLS	6-phosphogluconolactonase	1.0000	22.09	5	5	11
Q$453-2	M0R261	M0R261_HUMAN	PGLS	6-phosphogluconolactonase (Fragment)	1.0000	26.39	5	5	11
						AACCLAGAR	2	0.999	1
						ELPAAVAPAGPASLAR	2	0.9994	4
						TVIFVATGEGK	2	0.9857	1
						VTLTLPVLNAAR	2	0.9937	2
						WTLGFCDER	2	0.9992	3
Q$454-1	Q15102	PA1B3_HUMAN	PAFAH1B3	Platelet-activating factor acetylhydrolase IB subunit gamma	0.9436	3.9	1	1	1
Q$454-2	M0R389	M0R389_HUMAN	PAFAH1B3	Platelet-activating factor acetylhydrolase IB subunit gamma (	0.9436	5.49	1	1	1
						VVVLGLLPR	2	0.9436	1
Q$455-1	Q8IV08	PLD3_HUMAN	PLD3	Phospholipase D3	0.9669	1.84	1	1	1
Q$455-2	M0R3G9	M0R3G9_HUMAN	PLD3	Phospholipase D3 (Fragment)	0.9669	12.68	1	1	1
						SQLEAIFLR	2	0.9669	1
Q$456-1	O00187	MASP2_HUMAN	MASP2	Mannan-binding lectin serine protease 2	1.0000	16.33	48	58	235
						APGKDTFYSLGSSLDITF	2	0.9283	1
						APGKDTFYSLGSSLDITFR	2	0.9997	8
						APGKDTFYSLGSSLDITFR	3	0.9995	41

						ATLCGQESTDTER	2	0.9978	7
						ATLCGQESTDTERAPGK	2	0.9712	2
						ATLCGQESTDTERAPGKDTFYSLGSSLDITFR	3	0.8687	1
						DLELSHLCEYDFVK	2	0.9959	3
						DTFYSLGSSLDITFR	2	0.9997	37
						DTFYSLGSSLDITFR	3	0.9994	4
						FYSLGSSLDITFR	2	0.9726	2
						GKDTFYSLGSSLDITFR	2	0.9888	4
						GKDTFYSLGSSLDITFR	3	0.9618	2
						KDTFYSLGSSLDITFR	3	0.8956	1
						LASPGFPGEYANDQER	2	0.9997	7
						LASPGFPGEYANDQERR	2	0.999	6
						LASPGFPGEYANDQERR	3	0.9826	1
						LATLCGQESTDTER	2	0.9907	3
						LGSSLDITFR	2	0.9945	2
						LSHLCEYDFVK	2	0.8737	1
						LYFTHFDLELSHL	2	0.8293	1
						LYFTHFDLELSHL	3	0.8909	1
						LYFTHFDLELSHLCE	2	0.9704	2
						LYFTHFDLELSHLCEY	2	0.8552	1
						LYFTHFDLELSHLCEYDF	2	0.8348	1
						LYFTHFDLELSHLCEYDFV	2	0.9796	1
						LYFTHFDLELSHLCEYDFVK	2	0.9997	1
						LYFTHFDLELSHLCEYDFVK	3	0.9997	12
						LYFTHFDLELSHLCEYDFVK	4	0.9989	7
						RLYFTHFDLELSHLCEYDFVK	3	0.9001	2
						RLYFTHFDLELSHLCEYDFVK	4	0.9247	1
						RWTLTAPPGYR	3	0.9445	1
						SDYSNEKPF	2	0.9809	1
						SDYSNEKPFT	2	0.9584	1
						SDYSNEKPFTG	2	0.9984	2
						SDYSNEKPFTGF	2	0.9982	3
						SDYSNEKPFTGFE	2	0.9957	3
						SDYSNEKPFTGFEA	2	0.9978	3
						SDYSNEKPFTGFEAF	2	0.9985	4
						SDYSNEKPFTGFEAFY	2	0.9927	3
						SDYSNEKPFTGFEAFYA	2	0.936	1
						SDYSNEKPFTGFEAFYAAEDID	2	0.9466	1
						SLGSSLDITFR	2	0.9986	2
						TFYSLGSSLDITFR	2	0.9988	8
						THFDLELSHLCEYDFVK	2	0.9847	1
						THFDLELSHLCEYDFVK	3	0.9049	1
						TLCGQESTDTER	2	0.9928	5
						TLCGQESTDTERAPGK	2	0.8024	1
						VLATLCGQESTDTER	2	0.9997	7
						VLATLCGQESTDTERA	2	0.9769	1
						VLATLCGQESTDTERAPG	2	0.9852	4
						VLATLCGQESTDTERAPGK	2	0.9996	2
						VLATLCGQESTDTERAPGK	3	0.9991	3
						VLATLCGQESTDTERAPGKD	2	0.9807	1
						VLATLCGQESTDTERAPGKDT	2	0.9779	1
						VLATLCGQESTDTERAPGKDTFYSLGSSLDITFR	3	0.9993	4
						WPEPVFGR	2	0.9995	3
						WTLTAPPGYR	2	0.9997	4
						YSLGSSLDITFR	2	0.9925	1
Q$457-1	O00187-2	MASP2_HUMAN	MASP2	Isoform 2 of Mannan-binding lectin serine protease 2	1.0000	60.54	48	57	234
						APGKDTFYSLGSSLDITF	2	0.9283	1
						APGKDTFYSLGSSLDITFR	2	0.9997	8
						APGKDTFYSLGSSLDITFR	3	0.9995	41
						ATLCGQESTDTER	2	0.9978	7
						ATLCGQESTDTERAPGK	2	0.9712	2
						ATLCGQESTDTERAPGKDTFYSLGSSLDITFR	3	0.8687	1
						DLELSHLCEYDFVK	2	0.9959	3
						DTFYSLGSSLDITFR	2	0.9997	37
						DTFYSLGSSLDITFR	3	0.9994	4
						FYSLGSSLDITFR	2	0.9726	2
						GKDTFYSLGSSLDITFR	2	0.9888	4
						GKDTFYSLGSSLDITFR	3	0.9618	2
						KDTFYSLGSSLDITFR	3	0.8956	1
						LASPGFPGEYANDQER	2	0.9997	7
						LASPGFPGEYANDQERR	2	0.999	6
						LASPGFPGEYANDQERR	3	0.9826	1
						LATLCGQESTDTER	2	0.9907	3
						LGSSLDITFR	2	0.9945	2
						LSHLCEYDFVK	2	0.8737	1
						LYFTHFDLELSHL	2	0.8293	1
						LYFTHFDLELSHL	3	0.8909	1
						LYFTHFDLELSHLCE	2	0.9704	2
						LYFTHFDLELSHLCEY	2	0.8552	1
						LYFTHFDLELSHLCEYDF	2	0.8348	1
						LYFTHFDLELSHLCEYDFV	2	0.9796	1
						LYFTHFDLELSHLCEYDFVK	2	0.9997	1
						LYFTHFDLELSHLCEYDFVK	3	0.9997	12
						LYFTHFDLELSHLCEYDFVK	4	0.9989	7
						RLYFTHFDLELSHLCEYDFVK	3	0.9001	2
						RLYFTHFDLELSHLCEYDFVK	4	0.9247	1
						RWTLTAPPGYR	3	0.9445	1
						SDYSNEKPF	2	0.9809	1
						SDYSNEKPFT	2	0.9584	1
						SDYSNEKPFTG	2	0.9984	2
						SDYSNEKPFTGF	2	0.9982	3
						SDYSNEKPFTGFE	2	0.9957	3
						SDYSNEKPFTGFEA	2	0.9978	3
						SDYSNEKPFTGFEAF	2	0.9985	4
						SDYSNEKPFTGFEAFY	2	0.9927	3
						SDYSNEKPFTGFEAFYA	2	0.936	1
						SDYSNEKPFTGFEAFYAAEDID	2	0.9466	1
						SLGSSLDITFR	2	0.9986	2
						TFYSLGSSLDITFR	2	0.9988	8
						THFDLELSHLCEYDFVK	2	0.9847	1
						TLCGQESTDTER	2	0.9928	5
						TLCGQESTDTERAPGK	2	0.8024	1
						VLATLCGQESTDTER	2	0.9997	7
						VLATLCGQESTDTERA	2	0.9769	1
						VLATLCGQESTDTERAPG	2	0.9852	4
						VLATLCGQESTDTERAPGK	2	0.9996	2
						VLATLCGQESTDTERAPGK	3	0.9991	3
						VLATLCGQESTDTERAPGKD	2	0.9807	1
						VLATLCGQESTDTERAPGKDT	2	0.9779	1
						VLATLCGQESTDTERAPGKDTFYSLGSSLDITFR	3	0.9993	4
						WPEPVFGR	2	0.9995	3
						WTLTAPPGYR	2	0.9997	4
						YSLGSSLDITFR	2	0.9925	1
Q$458-1	O00203	AP3B1_HUMAN	AP3B1	AP-3 complex subunit beta-1	1.0000	2.01	2	2	3
Q$458-2	O00203-3	AP3B1_HUMAN	AP3B1	Isoform 2 of AP-3 complex subunit beta-1	1.0000	2.11	2	2	3
						EGSTAQLIINTEK	2	0.9988	2
						TVIGSVLLR	2	0.9964	1
Q$459-1	O00232	PSD12_HUMAN	PSMD12	26S proteasome non-ATPase regulatory subunit 12	1.0000	7.02	3	3	6
						ILVAVVK	2	0.9521	1
						LQEVIETLLSLEK	2	0.9996	3
						MEVDYSATVDQR	2	0.9996	2
Q$460-1	O00299	CLIC1_HUMAN	CLIC1	Chloride intracellular channel protein 1	1.0000	7.05	2	2	3
						GFTIPEAFR	2	0.9935	1

						LFMVLWLK	2	0.9929	2
Q$461-1	O00429-7	DNM1L_HUMAN	DNM1L	Isoform 7 of Dynamin-1-like protein	0.9996	6.19	3	3	3
						ALQGASQIIAEIR	2	0.9843	1
						KMTLLHGK	2	0.976	1
						YIETSELCGGAR	2	0.9722	1
Q$462-1	O00468-2	AGRIN_HUMAN	AGRN	Isoform 2 of Agrin	1.0000	1.02	2	2	3
						FGALCEAETGR	2	0.9995	2
						SFLAFPTLR	2	0.9986	1
Q$463-1	O00468	AGRIN_HUMAN	AGRN	Agrin	1.0000	26.8	38	41	77
Q$463-2	O00468-3	AGRIN_HUMAN	AGRN	Isoform 3 of Agrin	1.0000	27.04	38	41	77
Q$463-3	O00468-4	AGRIN_HUMAN	AGRN	Isoform 4 of Agrin	1.0000	26.91	38	41	77
Q$463-4	O00468-5	AGRIN_HUMAN	AGRN	Isoform 5 of Agrin	1.0000	26.93	38	41	77
Q$463-5	O00468-6	AGRIN_HUMAN	AGRN	Isoform 6 of Agrin	1.0000	27.09	38	41	77
Q$463-6	O00468-7	AGRIN_HUMAN	AGRN	Isoform 7 of Agrin	1.0000	26.85	38	41	77
						AAAVSSGFDGAIQLVSLGGR	2	0.9926	1
						ALGPAGCEADASAPATCAEMR	2	0.9997	4
						ALQSNHFELSLR	2	0.9962	1
						ASCYNSALGCCSDGKTPSLDAEGSNCPATK	3	0.9908	1
						AYGTGFVGCLR	2	0.998	1
						CDRCEPGFWNFR	3	0.999	2
						CEHPPPGPVCGSDGVTYGSACELR	3	0.9997	4
						CEPGFWNFR	2	0.9542	1
						EAACLQQTQIEEAR	2	0.9997	1
						EEEANVVLTGTVEEILNVDPVQHTYSCK	3	0.9643	1
						ESLLDGGNKVVISGFGDPLICDNQVSTGDTR	3	0.999	3
						FDTGSGPAVLTSAVPVEPGQWHR	3	0.9873	2
						FGALCEAETGR	2	0.9995	2
						FHCQCPPGR	2	0.9805	2
						FNAVCLSR	2	0.998	1
						GAPEGTVCGSDGADYPGECQLLR	2	0.9997	3
						GAPEGTVCGSDGADYPGECQLLR	3	0.9939	2
						GKDFLALALLDGR	3	0.9928	1
						GLHTFAR	2	0.9872	1
						GMLCGFGAVCEPNAEGPGR	2	0.9997	3
						HQGPCDQAPSPCLGVQCAFGATCAVK	3	0.9479	1
						IFFVNPAPPYLWPAHK	3	0.9974	3
						KFDGPCDPCQGALPDPSR	2	0.9996	2
						KFDGPCDPCQGALPDPSR	3	0.9828	2
						KSPCPSVVAPVCGSDASTYSNECELQR	3	0.9996	3
						LELGIGPGAATR	2	0.9993	1
						LLDVNNQR	2	0.9981	1
						MALEVVFLAR	2	0.9997	2
						QAPVCGDDGVTYENDCVMGR	2	0.9997	2
						REEEANVVLTGTVEEILNVDPVQHTYSCK	3	0.997	1
						REEEANVVLTGTVEEILNVDPVQHTYSCK	4	0.9334	1
						SADGLTASCLCPATCR	2	0.9996	2
						SFLAFPTLR	2	0.9986	1
						SIESTLDDLFR	2	0.9997	2
						SPCPSVVAPVCGSDASTYSNECELQR	3	0.9991	3
						SPCQPNPCHGAAPCR	2	0.9996	1
						TFVGAGLR	2	0.9946	1
						VCGSDGVTYGNECQLK	2	0.9997	3
						VGPTCADEKSPCQPNPCHGAAPCR	3	0.9993	4
						VTCDGAYRPVCAQDGR	2	0.9974	2
						VVISGFGDPLICDNQVSTGDTR	2	0.9997	2
Q$464-1	O00526	UPK2_HUMAN	UPK2	Uroplakin-2	0.9596	7.61	1	1	2
						LSAYQVTNLVPGTK	2	0.9596	2
Q$465-1	O00571	DDX3X_HUMAN	DDX3X	ATP-dependent RNA helicase DDX3X	1.0000	13.44	8	8	11
Q$465-2	O00571-2	DDX3X_HUMAN	DDX3X	Isoform 2 of ATP-dependent RNA helicase DDX3X	1.0000	13.78	8	8	11
						DLLDLLVEAK	2	0.9988	1
						DLMACAQTGSGK	2	0.9479	1
						ELAVQIYEEAR	2	0.9988	2
						GCHLLVATPGR	2	0.999	1
						HAIPIIK	2	0.9983	1
						HVINFDLPSDIEEYVHR	3	0.8247	1
						SFLLDLLNATGK	2	0.9996	3
						YLVLDEADR	2	0.965	1
Q$466-1	O14556	G3PT_HUMAN	GAPDHS	Glyceraldehyde-3-phosphate dehydrogenase, testis-specific	1.0000	4.41	2	2	3
						AGIALNDNFVK	2	0.9968	2
						LTGMAFR	2	0.9971	1
Q$467-1	O14773-2	TPP1_HUMAN	TPP1	Isoform 2 of Tripeptidyl-peptidase 1	1.0000	58.75	19	22	51
Q$467-2	O14773	TPP1_HUMAN	TPP1	Tripeptidyl-peptidase 1	1.0000	33.39	19	22	51
						ASGDSGAGCWSVSGR	2	0.9659	1
						AYPDVAALSDGYWVVSNR	2	0.9997	6
						AYPDVAALSDGYWVVSNR	3	0.9991	2
						AYPDVAALSDGYWVVSNRVPIPWVSGTSASTPVFGGILSLINEHR	4	0.9741	1
						FASGDSGAGCWSVSGR	2	0.9942	2
						FGGILSLINEHR	3	0.9551	2
						FGGNFAHQASVAR	2	0.8323	1
						GDDEDSLSSAYIQR	2	0.8765	1
						GLTLLFASGDSGAGCWSVSGR	2	0.9997	7
						GLTLLFASGDSGAGCWSVSGR	3	0.9252	1
						HQFRPTFPA	2	0.8441	1
						HQFRPTFPASSPY	2	0.8794	1
						HQFRPTFPASSPYVT	2	0.8469	1
						HQFRPTFPASSPYVTTVGGTSFQEPFLITNEIV	3	0.9398	1
						ILSGRPPLGFLNPR	2	0.9992	5
						ILSGRPPLGFLNPR	3	0.9994	3
						LFGGNFAHQASVAR	2	0.9997	3
						LITNEIVDYISGGGFSNVFPRPSYQEEAVTK	3	0.8827	2
						LYQQHGAGLFDVTR	3	0.9996	3
						SYGDDEDSLSSAYIQR	2	0.9871	1
						VNTELMK	2	0.9827	1
						VPIPWVSGTSASTPVFGGILSLINEHR	3	0.9997	5
Q$468-1	O14980	XPO1_HUMAN	XPO1	Exportin-1	0.9990	3.45	2	2	2
						EFAGEDTSDLFLEER	2	0.9904	1
						LDINLLDNVVNCLYHGEGAQQR	3	0.8917	1
Q$469-1	O15269	SPTC1_HUMAN	SPTLC1	Serine palmitoyltransferase 1	0.9765	2.96	1	1	2
						VVVTVEQTEEELER	2	0.9765	2
Q$470-1	O43143	DHX15_HUMAN	DHX15	Putative pre-mRNA-splicing factor ATP-dependent RNA helica	1.0000	3.02	2	2	2
						TLATDILMGVLK	2	0.9986	1
						YGVIILDEAHER	2	0.9893	1
Q$471-1	O43175	SERA_HUMAN	PHGDH	D-3-phosphoglycerate dehydrogenase	1.0000	10.88	4	4	6
						AGTGVDNVDLEAATR	2	0.9994	2
						GGIVDEGALLR	2	0.999	1
						QNLSKEELIAELQDCEGLIVR	3	0.9443	1
						VTADVINAAEK	2	0.9972	2
Q$472-1	O43653	PSCA_HUMAN	PSCA	Prostate stem cell antigen	1.0000	27.64	3	3	5
						AVGLLTVISK	2	0.9998	3
						GCSLNCVDDSQDYYVGK	2	0.9995	1
						LLCYSCK	2	0.9548	1
Q$473-1	O43707	ACTN4_HUMAN	ACTN4	Alpha-actinin-4	1.0000	5.49	4	4	10
Q$473-2	O43707-2	ACTN4_HUMAN	ACTN4	Isoform ACTN4ISO of Alpha-actinin-4	1.0000	7.23	4	4	10
						ETTDTDTADQVIASFK	2	0.9991	2
						LASDLLEWIR	2	0.9974	3
						TINEVENQILTR	2	0.999	2
						VGWEQLLTTIAR	2	0.9996	3
Q$474-1	Q14533	KRT81_HUMAN	KRT81	Keratin, type II cuticular Hb1	1.0000	2.18	1	1	2
Q$474-2	P78385	KRT83_HUMAN	KRT83	Keratin, type II cuticular Hb3	1.0000	2.23	1	1	2
Q$474-3	O43790	KRT86_HUMAN	KRT86	Keratin, type II cuticular Hb6	1.0000	2.26	1	1	2
						LGLDIEIATYR	2	0.9976	2
Q$475-1	O43865-2	SAHH2_HUMAN	AHCYL1	Isoform 2 of Putative adenosylhomocysteinase 2	0.9999	5.8	3	3	3
Q$475-2	O43865	SAHH2_HUMAN	AHCYL1	Putative adenosylhomocysteinase 2	0.9999	5.28	3	3	3

						QVVVCGYGEVGK	2	0.913	1
						TPELTWER	2	0.9921	1
						VVLLAEGR	2	0.9833	1
Q$476-1	O60841	IF2P_HUMAN	EIF5B	Eukaryotic translation initiation factor 5B	1.0000	6.48	5	5	6
						HFEATDILVSK	2	0.9753	1
						IPGMLIIDTPGHESFSNLR	3	0.9899	2
						LAHCEELR	2	0.9516	1
						LKEGDTIIVPGVEGPIVTQIR	3	0.9933	1
						SRINSSGESGDESDEFLQSR	2	0.8462	1
Q$477-1	O60884	DNJA2_HUMAN	DNAJA2	DnaJ homolog subfamily A member 2	0.9781	3.64	1	1	1
						IGLVEALCGFQFTFK	2	0.9781	1
Q$478-1	O75083	WDR1_HUMAN	WDR1	WD repeat-containing protein 1	1.0000	44.06	19	20	40
						CFSIDNPGYEPEVVAVHPGGDTVAIGGVDGNVR	3	0.9987	2
						CVAVGPGGYAVVVCIGQIVLLKDQR	3	0.9974	4
						DIAWTEDSKR	2	0.9981	2
						DIYTEHAHQVVVAK	2	0.9728	1
						FATASADGQIYIYDGK	2	0.9997	3
						FGAVFLWDSGSSVGEITGHNK	3	0.9967	3
						FTIGDHSR	2	0.9984	1
						GPVTDVAYSHDGAFLAVCDASK	3	0.9995	5
						IAVVGEGR	2	0.9996	2
						IKDIAWTEDSKR	2	0.9987	2
						IKDIAWTEDSKR	3	0.9854	1
						LATGSDDNCAAFFEGPPFK	2	0.999	2
						LYSILGTTLKDEGK	2	0.9984	2
						NIDNPALADIYTEHAHQVVVAK	3	0.9916	2
						NNPSKPLHVIK	2	0.9942	1
						TGSDDNCAAFFEGPPFK	2	0.9332	1
						VINSVDIK	2	0.9258	1
						VVTVFSVADGYSENNVFYGHHAK	3	0.9373	1
						YAPSGFYIASGDVSGK	2	0.9997	3
						YEYQPFAGK	2	0.9981	1
Q$479-1	O75112	LDB3_HUMAN	LDB3	LIM domain-binding protein 3	0.9768	14.03	2	2	2
						GSLVAPSPSPEARASPGTPGTPELRPTFSPAFSRPSAFSSLAEASDPGPPRASLR	5	0.8527	1
						SSTPIEHAPVCTSQATTPLLPASAQPPAAASPSAASPPLATAAAHTA	4	0.8427	1
Q$480-1	O75351	VPS4B_HUMAN	VPS4B	Vacuolar protein sorting-associated protein 4B	0.9560	3.6	1	1	1
						SLSNTKPTVNEHDLLK	3	0.8754	1
Q$481-1	O75494-2	SRS10_HUMAN	SRSF10	Isoform 2 of Serine/arginine-rich splicing factor 10	1.0000	10.34	2	2	6
Q$481-2	O75494-3	SRS10_HUMAN	SRSF10	Isoform 3 of Serine/arginine-rich splicing factor 10	1.0000	14.75	2	2	6
Q$481-3	O75494-4	SRS10_HUMAN	SRSF10	Isoform 4 of Serine/arginine-rich splicing factor 10	1.0000	15.61	2	2	6
Q$481-4	O75494-5	SRS10_HUMAN	SRSF10	Isoform 5 of Serine/arginine-rich splicing factor 10	1.0000	16.36	2	2	6
Q$481-5	O75494-6	SRS10_HUMAN	SRSF10	Isoform 6 of Serine/arginine-rich splicing factor 10	1.0000	14.84	2	2	6
Q$481-6	O75494	SRS10_HUMAN	SRSF10	Serine/arginine-rich splicing factor 10	1.0000	10.31	2	2	6
Q$481-7	Q5JRI1	Q5JRI1_HUMAN	SRSF10	Serine/arginine-rich-splicing factor 10	1.0000	15.7	2	2	6
						GFAYVQFEDVR	2	0.9993	2
						YGPIVDVYVPLDFYTR	2	0.9996	4
Q$482-1	O75531	BAF_HUMAN	BANF1	Barrier-to-autointegration factor	0.9789	13.48	1	1	3
						AYVVLGQFLVLK	2	0.9789	3
Q$483-1	O75534	CSDE1_HUMAN	CSDE1	Cold shock domain-containing protein E1	1.0000	5.01	3	3	5
Q$483-2	O75534-2	CSDE1_HUMAN	CSDE1	Isoform 2 of Cold shock domain-containing protein E1	1.0000	5.22	3	3	5
Q$483-3	O75534-3	CSDE1_HUMAN	CSDE1	Isoform 3 of Cold shock domain-containing protein E1	1.0000	4.92	3	3	5
Q$483-4	O75534-4	CSDE1_HUMAN	CSDE1	Isoform 4 of Cold shock domain-containing protein E1	1.0000	4.74	3	3	5
						ATNIEVLSNTFQFTNEAR	2	0.9994	2
						LLGYVATLK	2	0.9753	1
						LLTSYGFIQCSER	2	0.9984	2
Q$484-1	O75594	PGRP1_HUMAN	PGLYRP1	Peptidoglycan recognition protein 1	1.0000	19.9	2	2	5
						AAQGLLACGVAQGALR	2	0.9993	3
						YVVVSHTAGSSCNTPASCQQQAR	3	0.9904	2
Q$485-1	O75631	UPK3A_HUMAN	UPK3A	Uroplakin-3a	1.0000	31.01	4	4	7
						ASQILNAYLVR	2	0.9992	1
						AVAFDLIPCSDLPSLDAIGDVSK	2	0.9996	3
						EALTGTHEVYLYVLVDSAISR	3	0.9919	2
						VNLQPQLASVTFATNNPTLTTVALEKPLCMFDSK	3	0.8699	1
Q$486-1	O75636	FCN3_HUMAN	FCN3	Ficolin-3	1.0000	11.04	3	4	7
Q$486-2	Q5SSB9	Q5SSB9_HUMAN	FCN3	Ficolin-3	1.0000	33.33	3	4	7
Q$486-3	O75636-2	FCN3_HUMAN	FCN3	Isoform 2 of Ficolin-3	1.0000	11.46	3	4	7
						LLGEVDHYQLALGK	2	0.9839	1
						RQDGSVDFFR	2	0.9842	3
						TFAHYATFR	2	0.9936	2
						TFAHYATFR	3	0.9946	1
Q$487-1	O75891-2	AL1L1_HUMAN	ALDH1L1	Isoform 2 of Cytosolic 10-formyltetrahydrofolate dehydroger	1.0000	13.98	8	8	17
						ANATEFGLASGVFTR	2	0.9997	2
						DLGEAALNEYLR	2	0.9996	2
						FADGDLDAVLSR	2	0.9995	4
						GAASSVLELTEAELVTAEAVR	3	0.9967	4
						IAVIGQSLFGQEVYCHLRK	4	0.8671	1
						MILASNFFK	2	0.9601	1
						SPLIIFADCDLNK	2	0.9994	2
						TDVAAPFGGFK	2	0.9467	1
Q$488-1	O75891	AL1L1_HUMAN	ALDH1L1	Cytosolic 10-formyltetrahydrofolate dehydrogenase	1.0000	14.63	10	10	19
Q$488-2	O75891-3	AL1L1_HUMAN	ALDH1L1	Isoform 3 of Cytosolic 10-formyltetrahydrofolate dehydroger	1.0000	14.47	10	10	19
						ANATEFGLASGVFTR	2	0.9997	2
						DLGEAALNEYLR	2	0.9996	2
						EESFGPVMIISR	2	0.942	1
						FADGDLDAVLSR	2	0.9995	4
						FLFPEGIK	2	0.9765	1
						GAASSVLELTEAELVTAEAVR	3	0.9967	4
						IAVIGQSLFGQEVYCHLRK	4	0.8671	1
						MILASNFFK	2	0.9601	1
						SPLIIFADCDLNK	2	0.9994	2
						TDVAAPFGGFK	2	0.9467	1
Q$489-1	O76027	ANXA9_HUMAN	ANXA9	Annexin A9	1.0000	8.41	2	3	7
						ASDSAVDVAIEILATR	2	0.9993	3
						ASDSAVDVAIEILATR	3	0.999	2
						TPPQLQECLAVYK	2	0.9938	2
Q$490-1	O95343	SIX3_HUMAN	SIX3	Homeobox protein SIX3	0.9848	13.86	1	1	1
						SILLASSGGGNGAGGGGGAGGGSGGGNGAGGGGAGGAGGGGGGGSR	3	0.9495	1
Q$491-1	O95445	APOM_HUMAN	APOM	Apolipoprotein M	0.9998	9.04	2	2	2
Q$491-2	O95445-2	APOM_HUMAN	APOM	Isoform 2 of Apolipoprotein M	0.9998	14.66	2	2	2
						AFLLTPR	2	0.9854	1
						NQEACELSNN	2	0.9919	1
Q$492-1	O95865	DDAH2_HUMAN	DDAH2	N(G),N(G)-dimethylarginine dimethylaminohydrolase 2	1.0000	47.37	12	14	27
						AGAGLSSLCLVLSTRPH	3	0.8454	1
						AGAGLSSLCLVLSTRPHS	2	0.9975	1
						AGAGLSSLCLVLSTRPHS	3	0.9941	1
						ALQDLGLR	2	0.9348	1
						DFAVSTVPVSGPSHLR	2	0.9996	3
						DFAVSTVPVSGPSHLR	3	0.9982	1
						EFFVGLSK	2	0.9909	1
						GAEIVADTFR	2	0.9985	2
						GAEIVADTFRDFAVSTVPVSGPSHLR	3	0.9985	3
						GGGDLPNSQEALQK	2	0.9996	2
						GVPESLASGEGAGAGLPALDLAK	2	0.9996	2
						IVEIGDENATLDGTDVLFTGR	2	0.9997	5
						KALQDLGLR	2	0.9989	1
						LSDVTLVPVSCSELEK	2	0.9992	3
Q$493-1	O95954-2	FTCD_HUMAN	FTCD	Isoform C of Formimidoyltransferase-cyclodeaminase	1.0000	21.85	6	7	9
						DQPPAGSQDPGCTGAGLLGDPAGVTVR	3	0.8943	1
						GVSVDECVLCAQAFGQR	2	0.9985	2
						LAEELDVPVYLYGEAAR	2	0.9967	1
						LGLDSLCPFSPK	2	0.9797	1
						NQEVIDAISGAITQTPGCVLLDVDAGPSTNR	3	0.9664	1
						TVYTFVGPPECVVEGALNAAR	2	0.9984	2

						TVYTFVGPPECVVEGALNAAR	3	0.9894	1
Q$494-1	O95954-4	FTCD_HUMAN	FTCD	Isoform E of Formimidoyltransferase-cyclodeaminase	1.0000	24.05	2	3	5
						GVSVDECVLCAQAFGQR	2	0.9985	2
						TVYTFVGPPECVVEGALNAAR	2	0.9984	2
						TVYTFVGPPECVVEGALNAAR	3	0.9894	1
Q$495-1	Q4JM47	Q4JM47_HUMAN	AGR2	AGR2	1.0000	32.82	6	8	13
Q$495-2	O95994	AGR2_HUMAN	AGR2	Anterior gradient protein 2 homolog	1.0000	36.57	6	8	13
						GWGDQLIWTQTYEEALYK	2	0.9997	3
						HLSPDGQYVPR	2	0.9917	1
						LAEQFVLLNLVYETTDK	2	0.9997	3
						LAEQFVLLNLVYETTDK	3	0.9995	2
						LAEQFVLLNLVYETTDKHLSPDGQYVPR	3	0.9737	1
						LAEQFVLLNLVYETTDKHLSPDGQYVPR	4	0.987	1
						LYAYEPADTALLLDNMK	2	0.9969	1
						LYAYEPADTALLLDNMKK	3	0.9192	1
Q$496-1	O96009	NAPSA_HUMAN	NAPSA	Napsin-A	1.0000	12.14	4	4	7
						FAIQYGTGR	2	0.9995	1
						GCAAILDTGTSLITGPTEEIR	2	0.9997	4
						KPIFVPLSNYR	2	0.9135	1
						VGPGLTLCAK	2	0.9875	1
Q$497-1	P00167	CYB5_HUMAN	CYB5A	Cytochrome b5	1.0000	32.09	3	3	6
Q$497-2	P00167-2	CYB5_HUMAN	CYB5A	Isoform 2 of Cytochrome b5	1.0000	43.88	3	3	6
Q$497-3	P00167-3	CYB5_HUMAN	CYB5A	Isoform 3 of Cytochrome b5	1.0000	34.68	3	3	6
						EQAGGDATENFEDVGHSTDAR	3	0.9629	1
						FLEEHPGGEEVLR	2	0.9995	3
						STWLILHHK	2	0.9987	2
Q$498-1	P00338-2	LDHA_HUMAN	LDHA	Isoform 2 of L-lactate dehydrogenase A chain	1.0000	8.76	2	2	4
Q$498-2	P00338-5	LDHA_HUMAN	LDHA	Isoform 5 of L-lactate dehydrogenase A chain	1.0000	9.96	2	2	4
						DLADELALVDVIEDK	2	0.9992	3
						LVIITAGAR	2	0.9899	1
Q$499-1	P00338-4	LDHA_HUMAN	LDHA	Isoform 4 of L-lactate dehydrogenase A chain	1.0000	9.12	2	2	4
						DLADELALVDVIEDK	2	0.9992	3
						VTLTSEEEAR	2	0.9974	1
Q$500-1	P00338-3	LDHA_HUMAN	LDHA	Isoform 3 of L-lactate dehydrogenase A chain	1.0000	9.42	3	3	5
Q$500-2	P00338	LDHA_HUMAN	LDHA	L-lactate dehydrogenase A chain	1.0000	10.24	3	3	5
						DLADELALVDVIEDK	2	0.9992	3
						LVIITAGAR	2	0.9899	1
						VTLTSEEEAR	2	0.9974	1
Q$501-1	P00390	GSHR_HUMAN	GSR	Glutathione reductase, mitochondrial	1.0000	4.41	2	2	2
Q$501-2	P00390-3	GSHR_HUMAN	GSR	Isoform 2 of Glutathione reductase, mitochondrial	1.0000	4.67	2	2	2
Q$501-3	P00390-4	GSHR_HUMAN	GSR	Isoform 3 of Glutathione reductase, mitochondrial	1.0000	4.9	2	2	2
Q$501-4	P00390-5	GSHR_HUMAN	GSR	Isoform 4 of Glutathione reductase, mitochondrial	1.0000	5.23	2	2	2
Q$501-5	P00390-2	GSHR_HUMAN	GSR	Isoform Cytoplasmic of Glutathione reductase, mitochondria	1.0000	4.8	2	2	2
						ALLTPVAIAAGR	2	0.9989	1
						GIYAVGDVCGK	2	0.9721	1
Q$502-1	P00441	SODC_HUMAN	SOD1	Superoxide dismutase [Cu-Zn]	1.0000	71.43	5	5	9
						AVCVLKGDGPVQGIINFEQK	3	0.978	1
						GDGPVQGIINFEQK	2	0.9741	3
						GLTEGLHGFHVHEFGDNTAGCTSAGPHFNPLSR	4	0.8768	1
						HVGDLGNVTADKDGVADVSIEDSVISLSGDHCIIGR	4	0.992	2
						TLVVHEKADDLGKGGNEESTK	3	0.9952	2
Q$503-1	P00488	F13A_HUMAN	F13A1	Coagulation factor XIII A chain	1.0000	3.42	2	2	5
						HVYGELDVQIQR	2	0.9948	3
						MYVAVWTPYGVLR	2	0.9981	2
Q$504-1	P00491	PNPH_HUMAN	PNP	Purine nucleoside phosphorylase	0.9687	3.46	1	1	2
						VFGFSLITNK	2	0.9687	2
Q$505-1	P00734	THRB_HUMAN	F2	Prothrombin	1.0000	62.7	116	141	426
						AACLEGNCAEGLGTNYR	2	0.9984	5
						ACLEGNCAEGLGTNYR	2	0.9983	3
						ADLQENFCR	2	0.9919	3
						ADLQENFCRNPDSSTTGPWCYTTDPTVR	3	0.9663	7
						AEGLGTNYR	2	0.8349	1
						ANTFLEEVR	2	0.9055	1
						ATDVFWAK	2	0.985	1
						CAEGLGTNYR	2	0.9799	1
						CLEGNCAEGLGTNYR	2	0.9982	2
						CRNPDSSTTGPWCYTTDPTVR	3	0.9412	1
						CYTTDPTVR	2	0.9121	1
						DKLAACLEGN	2	0.8743	1
						DKLAACLEGNCAE	2	0.9463	1
						DKLAACLEGNCAEG	2	0.9838	1
						DKLAACLEGNCAEGL	2	0.9784	3
						DKLAACLEGNCAEGLG	2	0.8735	1
						DKLAACLEGNCAEGLGT	2	0.884	1
						DKLAACLEGNCAEGLGTN	2	0.9863	2
						DKLAACLEGNCAEGLGTNY	2	0.9888	3
						DKLAACLEGNCAEGLGTNYR	2	0.9996	12
						DKLAACLEGNCAEGLGTNYR	3	0.9995	16
						DLQENFCR	2	0.9867	1
						DSSTTGPWCYTTDPTVR	2	0.9989	7
						EGNCAEGLGTNYR	2	0.9867	3
						ELLESYIDGR	2	0.9997	8
						ENFCRNPDSSTTGPWCYTTDPTVR	3	0.9587	3
						ENLDRDIALMK	2	0.9973	1
						ETAASLLQAGYK	2	0.9997	1
						ETWTANVGK	2	0.995	1
						FCRNPDSSTTGPWCYTTDPTVR	2	0.9566	2
						FCRNPDSSTTGPWCYTTDPTVR	3	0.9785	4
						GADLQENFCRNPDSSTTGPWCYTTDPTVR	3	0.9484	2
						GDACEGDSGGPFVMK	2	0.9997	5
						GIECQLWR	2	0.9794	1
						GNCAEGLGTNYR	2	0.9141	1
						GQDQVTVAMTPR	2	0.9788	2
						GQPSVLQVVNL	2	0.9144	1
						GQPSVLQVVNLPIVER	2	0.9859	3
						GQPSVLQVVNLPIVERPV	2	0.9963	1
						GQPSVLQVVNLPIVERPVCK	2	0.9997	2
						GQPSVLQVVNLPIVERPVCK	3	0.9992	1
						GQPSVLQVVNLPIVERPVCKDSTR	3	0.9883	2
						HQDFNSAVQLVENFCR	2	0.9997	4
						HQDFNSAVQLVENFCR	3	0.9997	4
						IECQLWR	2	0.9399	1
						ITDNMFCAGYKPDEGK	2	0.9997	3
						ITDNMFCAGYKPDEGKR	2	0.9997	3
						ITDNMFCAGYKPDEGKR	3	0.9177	1
						IVEGSDAEIGMSPWQVMLFR	3	0.9997	3
						KLAACLEGNCAEGLGTNYR	2	0.9982	3
						KPVAFSDYIHPVCLPDRETAASLLQAGYK	3	0.9333	1
						KPVAFSDYIHPVCLPDRETAASLLQAGYK	4	0.9893	1
						KSPQELLCGASLI	2	0.8909	1
						KSPQELLCGASLISDR	2	0.9997	6
						KSPQELLCGASLISDR	3	0.9997	5
						LAACLEGNCAEGLGTN	2	0.8584	1
						LAACLEGNCAEGLGTNY	2	0.9738	2
						LAACLEGNCAEGLGTNYR	2	0.9997	12
						LAACLEGNCAEGLGTNYR	3	0.9947	2
						LAVTTHGLPCL	2	0.8756	1
						LAVTTHGLPCLAWASA	2	0.8434	1
						LAVTTHGLPCLAWASAQ	2	0.8619	1
						LAVTTHGLPCLAWASAQA	2	0.9815	3
						LAVTTHGLPCLAWASAQAK	2	0.9997	4
						LAVTTHGLPCLAWASAQAK	3	0.9997	7
						LEGNCAEGLGTNYR	2	0.9988	4

						LKKPVAFSDYIHPV	3	0.8441	1
						LKKPVAFSDYIHPVCLPDRETAASLLQAGYK	3	0.9938	2
						LKKPVAFSDYIHPVCLPDRETAASLLQAGYK	4	0.9705	1
						LQENFCR	2	0.9506	1
						LQENFCRNPDSSTTGPWCYTTDPTVR	3	0.9757	5
						LSPPLEQCVPDR	2	0.9732	1
						NCAEGLGTNYR	2	0.9881	1
						NFCRNPDSSTTGPWCYTTDPTVR	3	0.9668	3
						NLPIVERPVCK	2	0.9863	1
						NPDGDEEGVWCYVAGKPGD	2	0.984	1
						NPDSSTTGPWCYTTD	2	0.9983	3
						NPDSSTTGPWCYTTDPTVR	2	0.9998	16
						NPDSSTTGPWCYTTDPTVR	3	0.9997	5
						NPDSSTTGPWCYTTDPTVRR	2	0.9982	1
						NPDSSTTGPWCYTTDPTVRR	3	0.985	1
						PGADLQENFCR	2	0.9817	1
						PGADLQENFCRNPDSSTTGPWCYTTDPTVR	3	0.965	5
						PWCYTTDPTVR	2	0.9731	2
						QECSIPVCGQDQVTVAMTPR	2	0.9997	6
						QECSIPVCGQDQVTVAMTPR	3	0.9996	2
						RGDACEGDSGGPFVMK	2	0.9985	1
						RGDACEGDSGGPFVMK	3	0.9785	1
						RNPDSSTTGPWCYTTDPTVR	2	0.9965	3
						RNPDSSTTGPWCYTTDPTVR	3	0.9923	2
						RQECSIPVCG	2	0.8759	1
						RQECSIPVCGQ	2	0.9119	1
						RQECSIPVCGQD	2	0.9889	2
						RQECSIPVCGQDQ	2	0.9989	7
						RQECSIPVCGQDQVT	2	0.9988	5
						RQECSIPVCGQDQVTV	2	0.9981	5
						RQECSIPVCGQDQVTVA	2	0.998	6
						RQECSIPVCGQDQVTVAM	2	0.8554	1
						RQECSIPVCGQDQVTVAMTP	2	0.9519	1
						RQECSIPVCGQDQVTVAMTPR	2	0.9997	9
						RQECSIPVCGQDQVTVAMTPR	3	0.9997	16
						SEGSSVNLSPPLEQCVPDR	2	0.9997	7
						SEGSSVNLSPPLEQCVPDR	3	0.9985	1
						SEGSSVNLSPPLEQCVPDRG	2	0.9736	2
						SEGSSVNLSPPLEQCVPDRGQQY	2	0.9171	1
						SEGSSVNLSPPLEQCVPDRGQQYQGR	3	0.9963	4
						SGIECQLW	2	0.9776	1
						SGIECQLWR	2	0.9997	9
						SGIECQLWR	3	0.9843	1
						SIPVCGQDQVTVAMTPR	2	0.9989	5
						SIPVCGQDQVTVAMTPR	3	0.9642	1
						SLEDKTERELLESYIDGR	3	0.9994	6
						SPPLEQCVPDR	2	0.9557	3
						SPQELLCGASLISDR	2	0.9998	7
						SPQELLCGASLISDR	3	0.9949	1
						SRYPHKPEINSTTHPGADLQENFCR	3	0.9262	1
						SRYPHKPEINSTTHPGADLQENFCR	4	0.9682	1
						SSTTGPWCYTTDPTVR	2	0.9989	3
						STATDVFWAK	2	0.9585	1
						STTGPWCYTTDPTVR	2	0.9986	3
						STTHPGADLQENFCR	2	0.9989	5
						STTHPGADLQENFCR	3	0.9776	3
						STTHPGADLQENFCRNPDSSTTGPWCYTTDPTVR	3	0.9617	3
						TATDVFWAK	2	0.9794	1
						TFGSGEADCGLRPL	2	0.9925	3
						TFGSGEADCGLRPLF	2	0.9865	4
						TFGSGEADCGLRPLFE	2	0.9784	1
						TFGSGEADCGLRPLFEK	2	0.9997	5
						TFGSGEADCGLRPLFEK	3	0.9994	1
						TFGSGEADCGLRPLFEKK	2	0.9997	4
						TFGSGEADCGLRPLFEKK	4	0.9974	3
						TGPWCYTTDPTVR	2	0.9978	3
						TPRDKLAACLEGNCAEGLGTNYR	3	0.9992	3
						TTGPWCYTTDPTVR	2	0.9979	3
						TTHPGADLQENFCR	3	0.9712	1
						VNLPIVERPVCK	2	0.9462	2
						VTGWGNLK	2	0.9932	2
						VTGWGNLKETWTANVGK	2	0.9995	3
						VTGWGNLKETWTANVGK	3	0.9988	2
						WVLTAAH	2	0.8499	1
						YTTDPTVR	2	0.9549	2
Q$506-1	P00739	HPTR_HUMAN	HPR	Haptoglobin-related protein	1.0000	26.15	8	8	12
Q$506-2	P00739-2	HPTR_HUMAN	HPR	Isoform 2 of Haptoglobin-related protein	1.0000	23.64	8	8	12
						AVGDKLPECEAVCGKPK	2	0.997	2
						GSFPWQAK	2	0.9876	1
						ILGGHLDAK	2	0.9991	1
						LRTEGDGVYTLNDKK	3	0.9803	1
						SCAVAEYGVYVK	2	0.9997	4
						SPVGVQPILNEHTFCVGMSK	3	0.9365	1
						TEGDGVYTLNDKK	2	0.9996	1
						VTSIQHWVQK	2	0.9648	1
Q$507-1	P00740	FA9_HUMAN	F9	Coagulation factor IX	1.0000	23.64	9	11	22
						FGSGYVSGWGR	2	0.9992	2
						ITVVAGEHNIEETEHTEQK	3	0.9931	2
						NCELDVTCNIK	2	0.9993	1
						NSADNKVVCSCTEGYR	2	0.9985	2
						NSADNKVVCSCTEGYR	3	0.8358	1
						SCEPAVPFPCGR	2	0.9997	3
						SYECWCPFGFEGK	2	0.9957	3
						VDAFCGGSIVNEK	2	0.9996	3
						VVCSCTEGYR	2	0.99	1
						WIVTAAHCVETGVK	2	0.9996	3
						WIVTAAHCVETGVK	3	0.9349	1
Q$508-1	P00740-2	FA9_HUMAN	F9	Isoform 2 of Coagulation factor IX	1.0000	8.75	3	3	8
						FGSGYVSGWGR	2	0.9992	2
						SCEPAVPFPCGR	2	0.9997	3
						WIVTAAHCVETGVK	2	0.9996	3
Q$509-1	P00742	FA10_HUMAN	F10	Coagulation factor X	1.0000	24.18	11	14	27
						ACIPTGPYPCGK	2	0.9997	2
						ACIPTGPYPCGKQ	2	0.9304	2
						ETYDFDIAVLR	2	0.9997	3
						ETYDFDIAVLR	3	0.9426	1
						FKDTYFVTGIVSWGEGCAR	3	0.9988	2
						FTKETYDFDIAVLR	2	0.9996	2
						FTKETYDFDIAVLR	3	0.9993	2
						GYTLADNGK	2	0.9992	1
						LSSSFIITQNMFCAGYDTK	2	0.9996	4
						MLEVPYVDR	2	0.9993	1
						NCELFTR	2	0.9992	2
						NTEQEEGGEAVHEVEVVIK	2	0.9997	2
						NTEQEEGGEAVHEVEVVIK	3	0.9995	2
						TGIVSGFGR	2	0.9996	1
Q$510-1	P00747	PLMN_HUMAN	PLG	Plasminogen	1.0000	59.51	58	68	149
						ATTVTGTPCQDWAAQEPHR	2	0.9998	3
						ATTVTGTPCQDWAAQEPHR	3	0.9996	1
						CEEDEEFTCR	2	0.9986	3
						CQSWSSMTPHR	2	0.9982	2
						CQSWSSMTPHR	3	0.9558	1
						CTTPPPSSGPTYQCLK	2	0.9993	3

						DVVLFEKK	2	0.9304	1
						EAQLPVIENK	2	0.9995	2
						ELRPWCFTTDPNK	2	0.8957	1
						ELRPWCFTTDPNKR	2	0.964	1
						ELRPWCFTTDPNKR	3	0.999	2
						EQQCVIMAENR	2	0.9992	1
						EQQCVIMAENRK	2	0.9985	2
						FSPATHPSEGLEENYCR	2	0.9997	2
						FSPATHPSEGLEENYCR	3	0.9621	1
						FVTWIEGVMR	2	0.9996	4
						HSIFTPETNPR	2	0.9997	4
						HSIFTPETNPR	3	0.9989	1
						KLYDYCDVPQCA	2	0.9956	1
						KLYDYCDVPQCAA	2	0.8058	1
						KLYDYCDVPQCAAPSFDCGKPQVEP	3	0.8157	1
						KLYDYCDVPQCAAPSFDCGKPQVEPK	3	0.9997	6
						KQLGAGSIEECAAK	2	0.9995	2
						KSSIIIR	2	0.991	2
						KVYLSECK	2	0.9951	1
						LFLEPTR	2	0.9982	1
						LGAGSIEECAAK	2	0.9018	1
						LSSPAVITDK	2	0.9992	1
						LSSPAVITDKVIPACLPSPNYVVADRTECFITGWGETQGTFGAGLLK	4	0.8369	1
						LYDYCDVPQCAAPSFDCGKPQVEPK	3	0.9973	1
						MRDVVLFEK	3	0.9709	2
						NLDENYCR	2	0.9993	1
						NLDENYCRNPDGKR	2	0.9915	1
						NLEPHVQEIEVSR	2	0.899	1
						NPDADKGPWCFTTDPSVR	2	0.9996	4
						NPDADKGPWCFTTDPSVR	3	0.9992	4
						NPDGDVGGPWCYTTNPR	2	0.9998	6
						NPDNDPQGPWCYTTDPEKR	3	0.9993	3
						NYCRNPDGDVGGPWCYTTNPR	2	0.9996	5
						NYCRNPDGDVGGPWCYTTNPR	3	0.9995	9
						PDVETPSEEDCMFGNGK	2	0.9985	3
						QLGAGSIEECAAK	2	0.9994	2
						RAPWCHTTNSQVR	2	0.9981	2
						RAPWCHTTNSQVR	3	0.9874	1
						RATTVTGTPCQDWAAQEPHR	3	0.9996	3
						RWELCDIPR	2	0.9957	2
						SIFTPETNPR	2	0.9361	1
						TECFITGWGETQGTFGAGLLK	2	0.9997	4
						TECFITGWGETQGTFGAGLLK	3	0.9965	2
						TPENFPCK	2	0.9996	1
						TPENYPNAGLTMNYCR	2	0.9997	3
						VCNRYEFLNGR	2	0.9464	2
						VCNRYEFLNGR	3	0.9787	1
						VETPSEEDCMFGNGK	2	0.9906	2
						VGGPWCYTTNPR	2	0.9266	1
						VILGAHQEVNLEPHVQEIEVSR	3	0.9997	4
						VIPACLPSPNYVVADR	2	0.9997	6
						VIPACLPSPNYVVADRTECFITGWGETQGTFGAGLLK	3	0.8951	1
						VQDCYHGDGQSYR	2	0.9974	2
						VQSTELCAGHLAG	2	0.9476	1
						VQSTELCAGHLAGG	2	0.9587	1
						VQSTELCAGHLAGGT	2	0.9694	1
						VQSTELCAGHLAGGTD	2	0.9894	2
						VQSTELCAGHLAGGTDS	2	0.9525	1
						VQSTELCAGHLAGGTDSCQGDSGGPLVCFEK	3	0.9995	3
						VYLSECK	2	0.994	1
						WELCDIPR	2	0.9997	3
						YILQGVTSWGLGCARPNKPGVYVR	3	0.9964	3
Q$511-1	P00748	FA12_HUMAN	F12	Coagulation factor XII	1.0000	17.89	10	11	24
						CFEPQLLR	2	0.9867	1
						CLEVEGHR	2	0.9993	1
						DVAYYLAWIR	2	0.9916	2
						LHEAFSPVSYQHDLALLR	3	0.9995	2
						LTLQGIISWGSGCGDR	2	0.9995	4
						NKPGVYTDVAYYLAWIR	2	0.9996	3
						NKPGVYTDVAYYLAWIR	3	0.9995	4
						NPDNDIRPWCFVLNR	3	0.9482	1
						RLTLQGIISWGSGCGDR	3	0.9959	1
						TTLSGAPCQPWASEATYR	2	0.9997	3
						VVGGLVALR	2	0.9993	2
Q$512-1	P00918	CAH2_HUMAN	CA2	Carbonic anhydrase 2	0.9999	9.62	2	2	2
						AVQQPDGLAVLGIFLK	2	0.9148	1
						VVDVLDSIK	2	0.9382	1
Q$513-1	P00966	ASSY_HUMAN	ASS1	Argininosuccinate synthase	1.0000	74.76	29	33	58
						AELVYTGFWHSPECEFVR	2	0.9084	1
						APNTPDILEIEFK	2	0.9996	3
						EFVEEFIWPAIQSSALYEDR	3	0.9997	6
						EQGYDVIAYLANIGQK	2	0.9996	1
						EQGYDVIAYLANIGQKEDFEEAR	3	0.9702	2
						ESPLSLYNEELVSMNVQGDYEPTDATGFININSLR	3	0.9994	3
						FAELVYTGFWHSPECEFVR	2	0.9997	2
						FAELVYTGFWHSPECEFVR	3	0.9362	2
						FELSCYSLAPQIK	2	0.9997	3
						FELSCYSLAPQIK	3	0.8958	1
						FELSCYSLAPQIKV	2	0.977	2
						GIYETPAGTILYH	2	0.9658	2
						GIYETPAGTILYHAHLDIEAFTMDR	3	0.8278	1
						GNDQVRFELSCYSLAPQIK	3	0.8655	1
						GQVYILGR	2	0.9986	2
						GRNDLMEYAK	2	0.9436	2
						IDIVENR	2	0.9922	1
						KVFIEDVSR	2	0.9952	2
						KVFIEDVSR	3	0.9988	1
						MPEFYNR	2	0.9976	1
						NDLMEYAK	2	0.9963	1
						NPWSMDENLMHISYEAGILENPK	3	0.999	4
						NQAPPGLYTK	2	0.9967	1
						QHGIPIPVTPK	2	0.9801	1
						SGGLDTSCILVWLK	2	0.9741	1
						SYEAGILENPK	2	0.9797	1
						TQDPAKAPNTPDILEIEFK	3	0.9983	3
						TQDPAKAPNTPDILEIEFKK	3	0.9237	1
						VQVSVLK	2	0.9935	1
						VTNVKDGTTHQTSLELFMYLNEVAGK	3	0.9926	1
						YLLGTSLAR	2	0.8694	1
						YLLGTSLARPCIAR	2	0.9971	2
						YLLGTSLARPCIAR	3	0.902	1
Q$514-1	P01008	ANT3_HUMAN	SERPINC1	Antithrombin-III	1.0000	60.13	27	35	70
						ADGESCSASMMYQEGK	2	0.9996	2
						AFLEVNEEGSEAAASTAVVIAGR	2	0.9997	3
						AFLEVNEEGSEAAASTAVVIAGR	3	0.9994	4
						ANRPFLVFIR	2	0.9993	2
						ANRPFLVFIR	3	0.9996	3
						ATEDEGSEQKIPEATNR	2	0.9996	2
						ATEDEGSEQKIPEATNR	3	0.9519	1
						DDLYVSDAFHK	2	0.9996	1
						DIPMNPMCIYR	2	0.9976	1
						EQLQDMGLVDLFSPEK	2	0.9997	5
						EQLQDMGLVDLFSPEK	3	0.9705	1

						EVPLNTIIFMGR	2	0.9996	3
						FATTFYQHLADSK	2	0.999	3
						FATTFYQHLADSKNDNDNIFLSPLSISTAFAMTK	3	0.9979	2
						FRIEDGFSLK	2	0.9469	1
						FRIEDGFSLK	3	0.999	3
						FRIEDGFSLKEQLQDMGLVDLFSPEK	3	0.9966	2
						GSPVDICTAKPR	2	0.8451	1
						IEDGFSLKEQLQDMGLVDLFSPEK	3	0.8954	2
						ITDVIPSEAINELTVLVLVNTIYFK	2	0.9997	3
						ITDVIPSEAINELTVLVLVNTIYFK	3	0.9995	3
						LQPLDFKENAEQSR	2	0.9983	2
						NDNDNIFLSPLSISTAFAMTK	2	0.9996	2
						RVAEGTQVLELPFKGDDITMVLILPKPEK	4	0.9328	1
						RVWELSK	2	0.9933	1
						SKLPGIVAEGR	2	0.9922	1
						SKLPGIVAEGR	3	0.8738	1
						SKLPGIVAEGRDDLYVSDAFHK	4	0.9619	2
						SLNPNRVTFK	2	0.9378	1
						SPVDICTAKPR	2	0.9549	1
						TSDQIHFFFAK	2	0.9996	3
						TSDQIHFFFAK	3	0.9711	1
						VAEGTQVLELPFK	2	0.9995	3
						VAEGTQVLELPFKG	2	0.9691	2
						VAEGTQVLELPFKGDDITMVLILPKPEK	3	0.9969	1
Q$515-1	P01009	A1AT_HUMAN	SERPINA1	Alpha-1-antitrypsin	1.0000	69.86	47	58	120
						ALVNYIFFK	2	0.8542	1
						AVLTIDEK	2	0.9995	2
						DTEEEDFHVD	2	0.9957	3
						DTEEEDFHVDQV	2	0.8215	1
						DTEEEDFHVDQVTTVK	2	0.9997	2
						DTVFALVNYIFFK	2	0.9996	3
						ELDRDTVFALVNY	2	0.9545	1
						ELDRDTVFALVNYIFFK	2	0.9979	2
						ELDRDTVFALVNYIFFK	3	0.9993	3
						FFLPDEGK	2	0.9838	2
						FLENEDR	2	0.976	1
						FNKPFVF	2	0.9763	1
						FNKPFVFL	2	0.9853	3
						FNKPFVFLMIEQNTK	2	0.9996	3
						FNKPFVFLMIEQNTK	3	0.9995	3
						GKWERPFEVK	2	0.9023	1
						GTEAAGAMFLEAIPMSIPPEVK	3	0.9862	2
						ITPNLAEFAF	2	0.8964	1
						ITPNLAEFAFSLYR	2	0.9998	6
						ITPNLAEFAFSLYR	3	0.9997	5
						KLSSWVLLMK	2	0.9862	3
						KLSSWVLLMK	3	0.9975	2
						KQINDYVEK	2	0.9943	2
						LAEFAFSLYR	2	0.9971	3
						LGMFNIQH	2	0.9786	2
						LGMFNIQHCK	2	0.9911	2
						LQHLENELTH	2	0.971	1
						LQHLENELTHDIITK	2	0.9997	3
						LQHLENELTHDIITK	3	0.9992	1
						LQHLENELTHDIITK	4	0.9593	1
						LSGVTEEAPLK	2	0.954	1
						LSITGTYDLK	2	0.9997	6
						LSSWVLLMK	2	0.9996	3
						LVDKFLEDVK	2	0.9729	1
						LVDKFLEDVK	3	0.9812	1
						LVDKFLEDVKK	2	0.9978	2
						LVDKFLEDVKK	3	0.9984	1
						LYHSEAFTVNFGDTEEAK	3	0.9436	1
						LYHSEAFTVNFGDTEEAKK	3	0.9991	3
						LYHSEAFTVNFGDTEEAKK	4	0.9749	1
						NLAEFAFSLYR	2	0.9367	2
						QINDYVEK	2	0.9602	1
						QINDYVEKGTQGK	2	0.9963	2
						SASLHLPK	2	0.9995	3
						SASLHLPKL	2	0.9646	3
						SPLFMGK	2	0.9995	1
						SPVSIATAFAMLSLGTK	3	0.9295	1
						SVLGQLGITK	2	0.9997	3
						SVLGQLGITKV	2	0.9849	1
						TAFAMLSLGTK	2	0.8751	1
						TLNQPDSQLQLTTGNGLFLSEGLK	2	0.9985	1
						TLNQPDSQLQLTTGNGLFLSEGLK	3	0.9963	1
						VFSNGADLSGVTEEAPLK	2	0.9997	5
						VFSNGADLSGVTEEAPLKL	2	0.9694	2
						VFSNGADLSGVTEEAPLKLSK	2	0.9974	2
						VFSNGADLSGVTEEAPLKLSK	3	0.9966	3
						WERPFEV	2	0.8973	1
						WERPFEVK	2	0.8846	1
Q$516-1	P01011	AACT_HUMAN	SERPINA3	Alpha-1-antichymotrypsin	1.0000	38.06	15	16	36
						ADLSGITGAR	2	0.9993	3
						AVLDVFEEGTEASAATAVK	2	0.9997	4
						AVLDVFEEGTEASAATAVK	3	0.9946	1
						DEELSCTVVELK	2	0.9992	3
						DVFEEGTEASAATAVK	2	0.9969	2
						DYNLNDILLQLGIEEAFTSK	2	0.9958	1
						EIGELYLPK	2	0.9982	2
						EIGELYLPKF	2	0.9863	1
						EQLSLLDR	2	0.9332	1
						FNRPFLMIIVPTDTQNIFFMSK	3	0.9991	5
						GTHVDLGLASANVDFAFSLYK	3	0.9966	2
						ITLLSALVETR	2	0.9997	3
						LYGSEAFATDFQDSAAAK	2	0.9995	3
						NLAVSQVVHK	2	0.9969	2
						TLLSALVETR	2	0.8869	1
						VFEEGTEASAATAVK	2	0.9874	2
Q$517-1	P01023	A2MG_HUMAN	A2M	Alpha-2-macroglobulin	1.0000	29.04	39	41	77
						AAQVTIQSSGTFSSK	2	0.9993	2
						AFQPFFVELTMPYSVIR	2	0.9995	3
						AGAFCLSEDAGLGISSTASLR	2	0.9985	2
						AIGYLNTGYQR	2	0.9986	1
						ALFCLESAWK	2	0.966	2
						ALLEIPLTVTHPVVR	2	0.9821	3
						AQIQEEGTVVELTGR	2	0.9966	3
						AYIFIDEAHITQ	2	0.9588	2
						AYIFIDEAHITQA	2	0.9796	2
						AYIFIDEAHITQALIWLSQR	3	0.9994	5
						DLKPAIVK	2	0.9863	1
						FEVQVTVPK	2	0.9878	1
						FSGQLNSHGCFYQQVK	3	0.9635	1
						GEAFTLK	2	0.9821	1
						GEAFTLKA	2	0.9521	1
						GGVEDEVTLSAYITIALLEIPLTVTHPVVR	3	0.9959	1
						IAQWQSFQLEGGLK	2	0.9995	2
						ITIALLEIPLTVTHPVVR	3	0.8933	1
						LHTEAQIQEEGTVVELTGR	3	0.999	2
						LPPNVVEESAR	2	0.9705	1
						LVHVEEPHTETVR	2	0.9994	2
						NALFCLESAWK	2	0.9988	3

						NEDSLVFVQTDK	2	0.9992	2
						NQGNTWLTAFVLK	2	0.9994	3
						QFSFPLSSEPFQGSYK	2	0.998	2
						QGIPFFGQVR	2	0.9935	2
						RVTAAPQSVCALR	2	0.9882	2
						SDIAPVAR	2	0.9369	1
						SLFTDLEAENDVLH	2	0.9888	3
						SLFTDLEAENDVLHCVAFAVPK	2	0.9905	1
						SLFTDLEAENDVLHCVAFAVPK	3	0.999	2
						SSGSLLNNAIK	2	0.9936	1
						TEHPFTVEEFVLPK	2	0.9914	2
						TEHPFTVEEFVLPK	3	0.9346	1
						TGTHGLLVK	2	0.9992	2
						VGFYESDVMGR	2	0.9985	2
						VSVQLEASPAFLAVPVEK	2	0.9991	3
						VTAAPQSVCALR	2	0.9988	1
						VTGEGCVYLQTSLK	2	0.9992	3
						VVSMDENFHPLNELIPLVYIQDPK	3	0.997	1
						YDVENCLANK	2	0.9588	1
Q$518-1	P01024	CO3_HUMAN	C3	Complement C3	1.0000	64.4	147	175	397
						AAVYHHFISDGVR	2	0.9997	3
						AAVYHHFISDGVR	3	0.9995	3
						AAVYHHFISDGVRK	2	0.9961	1
						AAVYHHFISDGVRK	3	0.9991	3
						ACEPGVDYVYK	2	0.9991	1
						ACEPGVDYVYKT	2	0.8916	1
						ADIGCTPGSGK	2	0.9839	1
						AEDLVGK	2	0.9984	1
						AFVIFGIQDGEQR	2	0.919	1
						AFVLISLQEAK	2	0.8951	1
						AGDFLEANYMNLQR	2	0.9997	4
						AKDQLTCNKFDLK	2	0.9993	3
						ALLALLQLK	2	0.9582	1
						ALLQLKDFDFVPPVVR	2	0.9134	2
						APSTWLTAYVVK	2	0.9992	2
						ASHLGLAR	2	0.998	1
						AVHYLDETEQWEK	2	0.9639	2
						AVYHHFISDGVR	2	0.9948	3
						AVYHHFISDGVR	3	0.9631	1
						AYYENSPQQVFSTEFEVK	2	0.9997	6
						AYYENSPQQVFSTEFEVK	3	0.999	3
						AYYNLEESCTR	2	0.9915	1
						AYYTLIGASGQR	2	0.9886	2
						CAEENCFIQK	2	0.9997	1
						DAPDHQELNLDVSLQLPSR	3	0.9985	2
						DEDIIAEENIVSR	2	0.9562	1
						DFDFVPPVVR	2	0.9996	2
						DICEEQVNSLPGSITK	2	0.9997	4
						DKVSHSEDDCLAFK	2	0.9568	3
						DQLTCNKFDLK	2	0.9693	1
						DSCVGSLVVK	2	0.9994	2
						DSITTWEILAVSMSDKK	2	0.9993	1
						DTWVEHWPEEDECQDEENQK	3	0.9995	3
						DVSLQLPSR	2	0.9861	2
						DYAGVFSDAGLTFTSSSGQQTAQR	3	0.98	2
						EALKLEEK	2	0.9763	1
						ENEGFTVTAEGK	2	0.9704	2
						EPGQDLVVLPLSITTDFIPSFR	2	0.9997	5
						EPGQDLVVLPLSITTDFIPSFR	3	0.9842	2
						EVVADSVWVDVKD	2	0.9959	2
						EYVLPSFEVIVEPTEK	2	0.9997	4
						EYVLPSFEVIVEPTEK	3	0.9581	1
						EYVLPSFEVIVEPTEKF	2	0.929	1
						FDEYIMAIEQTIK	2	0.9483	2
						FVTVQATFGTQVVEK	2	0.9989	2
						FYYIYNEK	2	0.9994	2
						GICVADPFEVTVMQDFFIDLR	2	0.9997	2
						GLEVTITAR	2	0.9997	3
						GQGTLSVVTMYHAK	2	0.9997	2
						GQGTLSVVTMYHAK	3	0.9629	1
						GVFVLNKK	2	0.9412	1
						GYTQQLAFR	2	0.9913	1
						HFISDGVR	2	0.8323	1
						HYLDETEQWEK	2	0.9919	2
						IHWESASLLR	2	0.9995	2
						IHWESASLLR	3	0.9995	4
						IIAEENIVSR	2	0.8286	1
						ILLQGTPVAQMTEDAVDAER	2	0.9986	1
						ILLQGTPVAQMTEDAVDAER	3	0.9889	1
						ILLQGTPVAQMTEDAVDAERLK	3	0.9136	1
						IPIEDGSGEVVLSR	2	0.9997	8
						ISLPESLKR	2	0.9794	1
						ISLQEAK	2	0.8934	1
						IWDVVEK	2	0.9736	1
						KACEPGVDYVYK	2	0.8292	1
						KGYTQQLAFR	2	0.9982	1
						KQELSEAEQATR	2	0.9995	5
						KQELSEAEQATR	3	0.9183	1
						KVEGTAFVIFGIQDGEQR	3	0.9993	8
						KVLLDGVQNPR	2	0.9994	3
						KVLLDGVQNPR	3	0.9954	2
						LDKACEPGVDYVYK	2	0.9989	3
						LDKACEPGVDYVYK	3	0.8931	1
						LESEETMVLEAH	2	0.8048	1
						LESEETMVLEAHD	2	0.9932	2
						LESEETMVLEAHDAQG	2	0.928	1
						LESEETMVLEAHDAQGDVPVTV	2	0.8127	1
						LESEETMVLEAHDAQGDVPVTVTVHDFPGK	3	0.9996	3
						LESEETMVLEAHDAQGDVPVTVTVHDFPGK	4	0.9971	1
						LKGPLLNK	2	0.8267	1
						LPYSVVR	2	0.9989	2
						LPYSVVRNEQVEIR	3	0.9935	1
						LSINTHPSQKPLSITVR	2	0.9956	1
						LSINTHPSQKPLSITVR	3	0.998	2
						LVAYYTLIGASGQR	2	0.9997	7
						LVAYYTLIGASGQR	3	0.9996	4
						NLIAIDSQVLCGAVK	2	0.99	4
						NNNEKDMALTAFVLISLQEAK	3	0.9826	2
						NTLIIYLDK	2	0.9954	2
						NTLIIYLDKVSHSEDDCLAFK	3	0.991	2
						NTMILEICTR	2	0.9997	3
						PLSITTDFIPSFR	2	0.9773	1
						QCQDLGAFTESMVVFGCPN	2	0.9995	2
						QELSEAEQATR	2	0.9985	2
						QGALELIK	2	0.9965	1
						QVREPGQDLVVLPLSITTDFIPSFR	3	0.9978	6
						RAPSTWLTAYVVK	2	0.9484	1
						RAPSTWLTAYVVK	3	0.8601	1
						RIFTVNHK	2	0.9326	1
						RIHWESASLLR	3	0.9687	1
						RIPIEDGSGEVVLSR	2	0.9993	5
						RIPIEDGSGEVVLSR	3	0.9987	2
						RLVAYYTLIGASGQR	2	0.9888	3

						RLVAYYTLIGASGQR	3	0.8417	1
						SDAGLTFTSSSGQQTAQR	2	0.9935	2
						SDDKVTLEER	2	0.9993	10
						SEETKENEGFTVTAEGK	2	0.9996	3
						SEETKENEGFTVTAEGK	3	0.9994	2
						SGIPIVTSPYQIHF	2	0.9867	2
						SGIPIVTSPYQIHFTK	2	0.9997	3
						SGQSEDRQPVPGQQMTLK	2	0.9993	3
						SGSDEVQVGQQR	2	0.9996	4
						SITTDFIPSFR	2	0.9822	2
						SLQSGYLFIQTDK	2	0.9923	3
						SLYVSATVILHSGSDMVQAER	3	0.9473	1
						SNLDEDIIAEENIVSR	2	0.9997	7
						SNLDEDIIAEENIVSR	3	0.9967	3
						SPQQVFSTEFEVK	2	0.9646	2
						SSLSVPYVIVPLK	2	0.9995	3
						SYALLALQLK	2	0.9476	2
						SYTVAIAGYALAQMGR	2	0.9995	3
						SYTVAIAGYALAQMGR	3	0.9937	2
						TELRPGETL	2	0.89	1
						TELRPGETLNV	2	0.9835	2
						TELRPGETLNVN	2	0.9872	1
						TELRPGETLNVNF	2	0.9969	3
						TELRPGETLNVNFLLR	2	0.9993	4
						TELRPGETLNVNFLLR	3	0.9997	9
						TFISPIK	2	0.9968	1
						TFISPIKC	2	0.8338	1
						TGLQEVEVK	2	0.9997	2
						TGLQEVEVKA	2	0.9928	1
						TIYTPGSTVLY	2	0.9899	1
						TIYTPGSTVLYR	2	0.9986	2
						TKKQELSEAEQATR	2	0.9988	3
						TVMVNIENPEGIPVK	2	0.9358	1
						VAYYTLIGASGQR	2	0.8481	1
						VEGTAFVIFGIQDGEQR	2	0.9994	6
						VEGTAFVIFGIQDGEQR	3	0.8989	1
						VELLHNPAFCSL	2	0.8501	1
						VELLHNPAFCSLATTK	2	0.9996	2
						VELLHNPAFCSLATTK	3	0.9956	3
						VFLDCCNYITELR	2	0.9994	2
						VHQYFNVELIQPGAVK	2	0.9997	3
						VHQYFNVELIQPGAVK	3	0.9995	4
						VIFGIQDGEQR	2	0.8066	1
						VLISLQEAK	2	0.9639	2
						VLLDGVQNPR	2	0.9995	4
						VLPLSITTDFIPSFR	2	0.9563	1
						VLPSFEVIVEPTEK	2	0.8929	1
						VQLSNDFDEYIMAIEQTIK	2	0.9997	8
						VQLSNDFDEYIMAIEQTIK	3	0.9996	4
						VSHSEDDCLAFK	2	0.9986	2
						VSHSEDDCLAFK	3	0.9972	1
						VTIKPAPETEK	2	0.9948	1
						VTIKPAPETEK	3	0.9933	1
						VTIKPAPETEKRPQDAK	3	0.8802	2
						VVLVAVDK	2	0.9995	2
						VVLVAVDKGVFVLNK	3	0.9938	2
						VVLVAVDKGVFVLNKK	3	0.9989	2
						VYAYYNLEESCTR	2	0.9997	5
						VYAYYNLEESCTRF	2	0.8516	1
						VYHHFISDGVR	2	0.9538	2
						YAYYNLEESCTR	2	0.9903	3
						YENSPQQVFSTEFEVK	2	0.9757	1
						YISKYELDK	2	0.9784	1
						YISKYELDKAFSDR	3	0.9911	2
						YNLEESCTR	2	0.9332	2
						YSIITPNILR	2	0.9689	1
						YTLIGASGQR	2	0.9938	1
						YYGGGYGSTQATF	2	0.938	1
						YYGGGYGSTQATFMVFQALAQYQK	3	0.9996	4
						YYNLEESCTR	2	0.9785	3
						YYTLIGASGQR	2	0.9279	1
						YYTYLIMNK	2	0.9995	2
Q$519-1	P01031	CO5_HUMAN	C5	Complement C5	1.0000	13.84	19	20	32
						AFTECCVVASQLR	2	0.9846	1
						ALLVGEHLNIIVTPK	3	0.9978	2
						ATLLDIYK	2	0.9678	1
						DINYVNPVIK	2	0.9804	1
						ELSYYSLEDLNNK	2	0.9985	3
						ENSLYLTAFTVIGIR	2	0.9995	3
						ENSLYLTAFTVIGIR	3	0.9958	1
						ENSLYLTAFTVIGIRK	2	0.8992	1
						GGSASTWLTAFALR	2	0.9959	2
						IDTALIK	2	0.9007	1
						IVACASYKPSR	2	0.9986	1
						KAFDICPLVK	2	0.9925	2
						LLVYYIVTGEQTAELVSDSVWLNIEEK	3	0.9984	2
						LPYSVVR	2	0.9989	2
						LQGTLPVEAR	2	0.9877	1
						SYFPESWLWEVHLVPR	3	0.9423	1
						TDAPDLPEENQAR	2	0.9983	2
						TSTSEEVCSFYLK	2	0.9995	3
						VSITSITVENVFVK	2	0.9616	1
						YIYPLDSLTWIEYWPR	2	0.9031	1
Q$520-1	P01034	CYTC_HUMAN	CST3	Cystatin-C	0.9864	16.44	2	2	3
						AGVNYFLDVELGR	2	0.821	1
						ALDFAVGEYNK	2	0.9829	2
Q$521-1	P01042-2	KNG1_HUMAN	KNG1	Isoform LMW of Kininogen-1	1.0000	45.9	27	31	70
						DFVQPPTK	2	0.9592	1
						DIPTNSPELEETLTHTITK	2	0.9997	4
						EGDCPVQSGKTWQDCEYKDAAK	3	0.9822	2
						ENFLFLTPDCK	2	0.9996	2
						ESNEELTESCETK	2	0.9997	2
						ESNEELTESCETKK	2	0.9992	3
						ETTCSKESNEELTESCETK	2	0.9995	3
						ETTCSKESNEELTESCETK	3	0.9921	1
						ETTCSKESNEELTESCETKK	3	0.9836	2
						IASFSQNCDIYPGK	2	0.9995	2
						IASFSQNCDIYPGKD	2	0.8871	1
						IASFSQNCDIYPGKDFVQPPTK	3	0.999	7
						IGEIKEETTSHLR	2	0.9995	2
						IGEIKEETTSHLR	3	0.9988	1
						KLGQSLDCNAEVYVVPWEK	2	0.9997	2
						KLGQSLDCNAEVYVVPWEKK	3	0.986	2
						KYFIDFVAR	2	0.9993	3
						KYFIDFVAR	3	0.9991	1
						KYNSQNQSNNQFVLYR	2	0.9997	2
						LGQSLDCNAEVYVVPWEK	2	0.9997	4
						LGQSLDCNAEVYVVPWEKK	3	0.9992	2
						QVVAGLNFR	2	0.9996	1
						SLWNGDTGECTDNAYIDIQLR	2	0.9997	3
						TVGSDTFYSFK	2	0.9996	1
						TVGSDTFYSFKYEIK	2	0.9992	3
						TVGSDTFYSFKYEIK	3	0.8514	1

						TWQDCEYK	2	0.9985	1
						TWQDCEYKDAAK	2	0.9992	4
						YEIKEGDCPVQSGK	2	0.9913	1
						YFIDFVAR	2	0.9994	2
						YNSQNQSNNQFVLYR	2	0.9997	4
Q$522-1	P01042-3	KNG1_HUMAN	KNG1	Isoform 3 of Kininogen-1	1.0000	15.35	6	7	13
						ESNEELTESCETK	2	0.9997	2
						IASFSQNCDIYPGK	2	0.9995	2
						IGEIKEETTSHLR	2	0.9995	2
						IGEIKEETTSHLR	3	0.9988	1
						KYFIDFVAR	2	0.9993	3
						TVGSDTFYSFK	2	0.9996	1
						YFIDFVAR	2	0.9994	2
Q$523-1	P01133-2	EGF_HUMAN	EGF	Isoform 2 of Pro-epidermal growth factor	1.0000	11.07	11	12	38
						ALLETSEK	2	0.9909	1
						FIFWSSEVAGSLYR	2	0.9997	10
						FIFWSSEVAGSLYR	3	0.9995	4
						IDTEGTNYEQLVVDAGVSVIMDFHYNEKR	4	0.9962	2
						IESSSLQGLGR	2	0.9996	3
						ITAVSLDVLDKR	2	0.9996	3
						IYWVDLER	2	0.9997	3
						LFWIQYNR	2	0.9997	3
						LFWTDTGINPR	2	0.9997	3
						LGTAWCSCR	2	0.9988	1
						LIEEGVDVPEGLAVDWIGR	2	0.9998	4
						SLDVLDKR	2	0.9426	1
Q$524-1	P01133-3	EGF_HUMAN	EGF	Isoform 3 of Pro-epidermal growth factor	1.0000	34.22	42	52	121
Q$524-2	P01133	EGF_HUMAN	EGF	Pro-epidermal growth factor	1.0000	33.06	42	52	121
						ADLDGVGVK	2	0.9995	1
						AEDDTWEPEQK	2	0.9994	4
						ALLETSEK	2	0.9909	1
						ALLETSEKITAVSLDVLDKR	3	0.9995	2
						ERLIEEGVDVPEGLAVDWIGR	3	0.9992	3
						ERLIEEGVDVPEGLAVDWIGRR	3	0.996	2
						ERLIEEGVDVPEGLAVDWIGRR	4	0.8502	1
						FIFWSSEVAGSLYR	2	0.9997	10
						FIFWSSEVAGSLYR	3	0.9995	4
						GIAVHPMAK	2	0.9975	1
						HMHFDGTDYGTLLSQQMGMVYALDHDPVENK	3	0.98	1
						IDTEGTNYEQLVVDAGVSVIMDFHYNEK	3	0.9997	3
						IDTEGTNYEQLVVDAGVSVIMDFHYNEKR	3	0.9994	2
						IDTEGTNYEQLVVDAGVSVIMDFHYNEKR	4	0.9962	2
						IEEGVDVPEGLAVDWIGR	2	0.9817	2
						IESSSLQGLGR	2	0.9996	3
						IITKENISQPR	2	0.9945	1
						INLHSSFVPLGELK	2	0.9995	3
						INLHSSFVPLGELK	3	0.9644	1
						ITAVSLDVLDK	2	0.9996	1
						ITAVSLDVLDKR	2	0.9996	3
						ITAVSLDVLDKR	3	0.998	1
						IYFAHTALK	2	0.9997	2
						IYFAHTALK	3	0.9951	2
						IYWVDLER	2	0.9997	3
						LCSDIDECEMGVPVCPPASSK	2	0.9997	3
						LFWIQYNR	2	0.9997	3
						LFWTDTGINPR	2	0.9997	3
						LGTAWCSCR	2	0.9988	1
						LIEEGVDVPEGLAVDWIGR	2	0.9998	4
						LIEEGVDVPEGLAVDWIGR	3	0.9996	5
						LIEEGVDVPEGLAVDWIGRR	2	0.9894	2
						LIEEGVDVPEGLAVDWIGRR	3	0.998	2
						LLFANSQDIR	2	0.9597	2
						LVIASSDLIWPSGITI	2	0.8935	1
						LVIASSDLIWPSGITIDFLTDK	3	0.9991	2
						LVIASSDLIWPSGITIDFLTDKLYWCDAK	3	0.9992	3
						NQVTPLDILSK	2	0.9891	2
						QSVIEMANLDGSKR	2	0.9657	1
						RIYWVDLER	2	0.987	1
						RIYWVDLER	3	0.9895	1
						RLFWTDTGINPR	2	0.9967	3
						RLIEEGVDVPEGLAVDWIGR	3	0.9763	1
						SCAASGPQPFLLFANSQDIR	2	0.9997	5
						SCAASGPQPFLLFANSQDIR	3	0.9996	3
						SLDVLDKR	2	0.9426	1
						SSDLIWPSGITIDFLTDK	2	0.8924	1
						SWECDCFPGYDLQLDEK	2	0.9986	2
						TCLALDGHQLLAGGEVDLK	3	0.9986	4
						VVHPLAQPK	2	0.9994	1
						YCEDVNECAFWNHGCTLGCK	3	0.9868	3
						YPANVAVDPVER	2	0.9994	2
Q$525-1	P01229	LSHB_HUMAN	LHB	Lutropin subunit beta	0.9842	10.64	1	1	3
						GVDPVVSFPVALSCR	2	0.9842	3
Q$526-1	P01593	KV101_HUMAN		Ig kappa chain V-I region AG	0.9541	16.67	1	1	1
Q$526-2	P01594	KV102_HUMAN		Ig kappa chain V-I region AU	0.9541	16.67	1	1	1
Q$526-3	P01599	KV107_HUMAN		Ig kappa chain V-I region Gal	0.9541	16.67	1	1	1
Q$526-4	P01600	KV108_HUMAN		Ig kappa chain V-I region Hau	0.9541	16.67	1	1	1
Q$526-5	P01607	KV115_HUMAN		Ig kappa chain V-I region Rei	0.9541	16.67	1	1	1
Q$526-6	P01608	KV116_HUMAN		Ig kappa chain V-I region Roy	0.9541	16.67	1	1	1
Q$526-7	P01609	KV117_HUMAN		Ig kappa chain V-I region Scw	0.9541	16.67	1	1	1
Q$526-8	P80362	KV125_HUMAN		Ig kappa chain V-I region WAT	0.9541	16.67	1	1	1
Q$526-9	P01610	KV118_HUMAN		Ig kappa chain V-I region WEA	0.9541	16.67	1	1	1
						DIQMTQSPSSLSASVGDR	2	0.9541	1
Q$527-1	P01605	KV113_HUMAN		Ig kappa chain V-I region Lay	0.9454	8.33	1	1	1
Q$527-2	P04207	KV308_HUMAN		Ig kappa chain V-III region CLL	0.9454	6.98	1	1	1
Q$527-3	P01624	KV306_HUMAN		Ig kappa chain V-III region POM	0.9454	8.26	1	1	1
Q$527-4	P04434	KV310_HUMAN		Ig kappa chain V-III region VH (Fragment)	0.9454	7.76	1	1	1
						LLIYGASTR	2	0.9454	1
Q$528-1	P01614	KV201_HUMAN		Ig kappa chain V-II region Cum	1.0000	24.35	2	2	4
Q$528-2	P06309	KV205_HUMAN		Ig kappa chain V-II region GM607 (Fragment)	1.0000	23.93	2	2	4
Q$528-3	P01617	KV204_HUMAN		Ig kappa chain V-II region TEW	1.0000	24.78	2	2	4
						FSGSGSGTDFTLK	2	0.9993	2
						SLPVTPGEPASISCR	2	0.9804	2
Q$529-1	P01616	KV203_HUMAN		Ig kappa chain V-II region MIL	1.0000	13.39	2	2	3
						PVTPGEPASISCR	2	0.8984	1
						SLPVTPGEPASISCR	2	0.9804	2
Q$530-1	P18135	KV312_HUMAN		Ig kappa chain V-III region HAH	1.0000	37.98	4	4	8
Q$530-2	P18136	KV313_HUMAN		Ig kappa chain V-III region HIC	1.0000	37.98	4	4	8
Q$530-3	P01620	KV302_HUMAN		Ig kappa chain V-III region SIE	1.0000	44.95	4	4	8
Q$530-4	P01623	KV305_HUMAN		Ig kappa chain V-III region WOL	1.0000	44.95	4	4	8
						ATGIPDRFSGSGSGTDFTLTISR	3	0.9639	1
						EIVLTQSPGTLSLSPGER	2	0.999	1
						FSGSGSGTDFTLTISR	2	0.9996	5
						LIYGASSR	2	0.9531	1
Q$531-1	P04206	KV307_HUMAN		Ig kappa chain V-III region GOL	1.0000	31.19	2	2	6
Q$531-2	P01622	KV304_HUMAN		Ig kappa chain V-III region Ti	1.0000	31.19	2	2	6
						EIVLTQSPGTLSLSPGER	2	0.999	1
						FSGSGSGTDFTLTISR	2	0.9996	5
Q$532-1	P06314	KV404_HUMAN		Ig kappa chain V-IV region B17	1.0000	6.72	2	2	4
Q$532-2	P06313	KV403_HUMAN		Ig kappa chain V-IV region JI	1.0000	6.77	2	2	4
Q$532-3	P01625	KV402_HUMAN		Ig kappa chain V-IV region Len	1.0000	7.89	2	2	4
Q$532-4	P06312	KV401_HUMAN	IGKV4-1	Ig kappa chain V-IV region (Fragment)	1.0000	7.44	2	2	4
						LIYWASTR	2	0.9399	1
						LLIYWASTR	2	0.9982	3

Q$533-1	P01743	HV102_HUMAN		Ig heavy chain V-I region HG3	0.9333	9.4	1	1	1
						SEDTAVYYCAR	2	0.9333	1
Q$534-1	P01766	HV305_HUMAN		Ig heavy chain V-III region BRO	0.9989	9.17	1	1	2
Q$534-2	P01767	HV306_HUMAN		Ig heavy chain V-III region BUT	0.9989	9.57	1	1	2
Q$534-3	P01763	HV302_HUMAN		Ig heavy chain V-III region WEA	0.9989	9.65	1	1	2
						AEDTAVYYCAR	2	0.9982	2
Q$535-1	P06331	HV209_HUMAN		Ig heavy chain V-II region ARH-77	0.9666	10.96	1	1	2
Q$535-2	P01825	HV207_HUMAN		Ig heavy chain V-II region NEWM	0.9666	13.68	1	1	2
						LSSVTAADTAVYYCAR	2	0.9666	2
Q$536-1	P01834	IGKC_HUMAN	IGKC	Ig kappa chain C region	1.0000	83.96	21	25	104
						AAPSVFIFPPSDEQLK	2	0.9465	3
						ACEVTHQGLSSPVTK	2	0.9949	3
						CEVTHQGLSSPVTK	2	0.9972	3
						DSTYSLSSTLTLSK	2	0.9997	5
						EVTHQGLSSPVTK	2	0.9962	2
						HKVYACEVTHQGLSSPVTK	3	0.9993	4
						KVDNALQSGNSQESVTEQDSK	3	0.9436	1
						SGTASVVCLLNNFYPR	2	0.9997	10
						SGTASVVCLLNNFYPR	3	0.9996	4
						SGTASVVCLLNNFYPRE	2	0.9872	4
						SLSSTLTLSK	2	0.9662	1
						STYSLSSTLTLSK	2	0.947	2
						TVAAPSVFIFPPSDEQLK	2	0.9997	20
						TVAAPSVFIFPPSDEQLK	3	0.9992	15
						VCLLNNFYPR	2	0.8451	2
						VDNALQSGNSQESVTE	2	0.9882	2
						VDNALQSGNSQESVTEQDSK	2	0.9997	4
						VDNALQSGNSQESVTEQDSK	3	0.9989	2
						VDNALQSGNSQESVTEQDSKDSTYSLSSTLTLSK	3	0.9992	5
						VFIFPPSDEQLK	2	0.9189	4
						VYACEVTHQGL	2	0.9902	1
						VYACEVTHQGLSSPV	2	0.9455	1
						VYACEVTHQGLSSPVT	2	0.9881	2
						VYACEVTHQGLSSPVTK	2	0.9997	3
						VYACEVTHQGLSSPVTK	3	0.9996	1
Q$537-1	P01857	IGHG1_HUMAN	IGHG1	Ig gamma-1 chain C region	1.0000	38.18	15	17	35
						DYFPEPV	2	0.9023	1
						DYFPEPVTV	2	0.938	1
						DYFPEPVTVSWNSGALTSGVHTFP	2	0.9804	1
						FNWYVDGVEVHNAK	2	0.9997	2
						FNWYVDGVEVHNAK	3	0.9971	1
						GPSVFPLAPSSK	2	0.9996	3
						NQVSLTCLVK	2	0.9995	3
						NWYVDGVEVHNAK	2	0.9912	3
						STSGGTAALGCLVK	2	0.9996	2
						TPEVTCVVVDVSH	2	0.9846	3
						TPEVTCVVVDVSHE	2	0.8276	2
						TPEVTCVVVDVSHEDPEVK	2	0.9996	3
						TPEVTCVVVDVSHEDPEVK	3	0.9997	5
						TTPPVLDSDGSFFLY	2	0.9788	1
						TTPPVLDSDGSFFLYSK	3	0.998	2
						VVSVLTVLHQDWLNGK	3	0.9558	1
						WYVDGVEVHNAK	2	0.9784	1
Q$538-1	P01859	IGHG2_HUMAN	IGHG2	Ig gamma-2 chain C region	1.0000	37.12	14	15	31
						CCVECPPCPAPPVAGPS	2	0.8399	1
						DYFPEPV	2	0.9023	1
						DYFPEPVTV	2	0.938	1
						DYFPEPVTVSWNSGALTSGVHTFP	2	0.9804	1
						FNWYVDGVEVHNAK	2	0.9997	2
						GPSVFPLAPCSR	2	0.9996	3
						NQVSLTCLVK	2	0.9995	3
						NWYVDGVEVHNAK	2	0.9912	3
						STSESTAALGCLVK	2	0.9996	5
						STSESTAALGCLVK	3	0.9764	2
						TPEVTCVVVDVSH	2	0.9846	3
						TPEVTCVVVDVSHE	2	0.8276	2
						TPEVTCVVVDVSHEDPEVQF	2	0.975	2
						VVSVLTVVHQD	2	0.8156	1
						WYVDGVEVHNAK	2	0.9784	1
Q$539-1	P01860	IGHG3_HUMAN	IGHG3	Ig gamma-3 chain C region	1.0000	12.2	5	5	12
						GPSVFPLAPCSR	2	0.9996	3
						STSGGTAALGCLVK	2	0.9996	2
						TPEVTCVVVDVSH	2	0.9846	3
						TPEVTCVVVDVSHE	2	0.8276	2
						TPEVTCVVVDVSHEDPEVQF	2	0.975	2
Q$540-1	P01861	IGHG4_HUMAN	IGHG4	Ig gamma-4 chain C region	1.0000	24.77	6	6	15
						FNWYVDGVEVHNAK	2	0.9997	2
						GPSVFPLAPCSR	2	0.9996	3
						NQVSLTCLVK	2	0.9995	3
						STSESTAALGCLVK	2	0.9996	5
						TTPPVLDSDGSFFLY	2	0.9788	1
						VVSVLTVLHQDWLNGK	3	0.9558	1
Q$541-1	P01876	IGHA1_HUMAN	IGHA1	Ig alpha-1 chain C region	1.0000	17.85	5	5	9
						DASGVTFTWTPSSGK	2	0.9997	4
						QEPSQGTTTFAVTSILR	2	0.9972	1
						TFTCTAAYPESK	2	0.9991	2
						TPLTATLSK	2	0.9982	1
						WLQGSQELPR	2	0.9443	1
Q$542-1	P02042	HBD_HUMAN	HBD	Hemoglobin subunit delta	1.0000	92.52	60	78	235
						AFSDGLAHLDNLK	2	0.9924	5
						AGVANALAHKYH	2	0.9134	1
						DKLHVDPENFR	2	0.9673	3
						DKLHVDPENFR	3	0.9414	2
						EFTPQMQAAYQK	2	0.9963	2
						FFESFGDLS	2	0.939	1
						FFESFGDLSSPDAVMGNPK	2	0.9997	3
						FSDGLAHLDNLK	2	0.9345	3
						FSDGLAHLDNLK	3	0.8507	1
						GAFSDGLAHLDNLK	2	0.9951	6
						GLAHLDNLK	2	0.9422	1
						GNVLVCVLAR	2	0.9268	2
						GTFSQLSELHCDK	2	0.9894	1
						GTFSQLSELHCDKLHVDPENFR	3	0.9938	2
						HCDKLHVDPENFR	2	0.9801	2
						HCDKLHVDPENFR	3	0.8269	1
						HLTPEEK	2	0.9103	2
						HVDPENFR	2	0.9515	1
						KLHVDPENFR	2	0.9703	1
						KLHVDPENFR	3	0.8815	1
						KVLGAFSD	2	0.8856	1
						KVLGAFSDGLAHLD	2	0.961	2
						KVLGAFSDGLAHLDN	2	0.9751	3
						KVLGAFSDGLAHLDNL	2	0.9889	2
						KVLGAFSDGLAHLDNLK	2	0.9997	6
						KVLGAFSDGLAHLDNLK	3	0.9997	12
						KVLGAFSDGLAHLDNLK	4	0.9375	1
						LAHLDNLK	2	0.8465	1
						LHCDKLHVDPENFR	2	0.9735	2
						LHCDKLHVDPENFR	3	0.8133	1
						LHVDPENFR	2	0.9997	7
						LHVDPENFR	3	0.9996	4
						LLGNVLVC	2	0.9772	1
						LLGNVLVCV	2	0.9694	2

						LLGNVLVCVL	2	0.9978	2
						LLGNVLVCVLA	2	0.9959	2
						LLGNVLVCVLAR	2	0.9997	7
						LLGNVLVCVLAR	3	0.9996	2
						LLVVYPW	2	0.9962	2
						LLVVYPWT	2	0.9952	3
						LLVVYPWTQ	2	0.9963	2
						LLVVYPWTQR	3	0.9994	5
						LSELHCDKLHVDPENFR	2	0.9902	3
						LSELHCDKLHVDPENFR	3	0.9641	1
						LVVYPWTQR	2	0.996	3
						RLLVVYPWTQR	2	0.9957	4
						RLLVVYPWTQR	3	0.9801	2
						SDGLAHLDNLK	2	0.9895	5
						SDGLAHLDNLK	3	0.8253	1
						SELHCDKLHVDPENFR	2	0.9888	3
						SELHCDKLHVDPENFR	3	0.8897	1
						SELHCDKLHVDPENFR	4	0.9011	1
						SQLSELHCDKLHVDPENFR	3	0.9564	1
						TAVNALWGK	2	0.9997	2
						VAGVANALAHK	2	0.9934	2
						VAGVANALAHKYH	2	0.9256	1
						VDPENFR	2	0.94	1
						VGGEALGR	2	0.9882	2
						VHLTPEEK	2	0.9997	11
						VLGAFSDGLAH	2	0.8917	1
						VLGAFSDGLAHL	2	0.9865	6
						VLGAFSDGLAHLD	2	0.992	4
						VLGAFSDGLAHLDN	2	0.9941	4
						VLGAFSDGLAHLDNL	2	0.9966	8
						VLGAFSDGLAHLDNLK	2	0.9998	18
						VLGAFSDGLAHLDNLK	3	0.9997	13
						VLGAFSDGLAHLDNLKGT	2	0.9329	2
						VNVDAVGGEALGR	2	0.9997	6
						VNVDAVGGEALGRL	2	0.9716	2
						VVAGVANALA	2	0.8581	1
						VVAGVANALAH	2	0.9932	2
						VVAGVANALAHK	2	0.9997	3
						VVAGVANALAHK	3	0.9993	1
						VVAGVANALAHKY	2	0.9757	2
						VVAGVANALAHKYH	2	0.9996	3
						VVAGVANALAHKYH	3	0.9919	1
						VVAGVANALAHKYH	4	0.9959	1
						VVYPWTQR	2	0.9788	2
Q$543-1	P02100	HBE_HUMAN	HBE1	Hemoglobin subunit epsilon	1.0000	10.2	1	1	1
						LSELHCDKLHVDPEN	2	0.9348	1
Q$544-1	P02533	K1C14_HUMAN	KRT14	Keratin, type I cytoskeletal 14	1.0000	54.24	22	27	49
						ADLEMQIESLKEELAYLK	3	0.9948	3
						ADLEMQIESLKEELAYLKK	3	0.9862	2
						ALEEANADLEVK	2	0.9994	2
						APSTYGGGLSVSSSR	2	0.9995	2
						ASLENSLEETK	2	0.9996	1
						ASLENSLEETKGR	2	0.9933	1
						CEMEQQNQEYK	2	0.9997	2
						DAEEWFFTK	2	0.9989	2
						DAEEWFFTKTEELNR	2	0.9987	3
						DAEEWFFTKTEELNR	3	0.9614	1
						EVATNSELVQSGK	2	0.9997	2
						GSCGIGGGIGGGSSR	2	0.9993	1
						ILNEMRDQYEK	2	0.9902	1
						ILTATVDNANVLLQIDNAR	2	0.9995	2
						ILTATVDNANVLLQIDNAR	3	0.9973	3
						ISSVLAGGSCR	2	0.9994	1
						LLEGEDAHLSSSQFSSGSQSSR	3	0.9995	3
						MSVEADINGLR	2	0.9994	1
						NHEEEMNALR	2	0.9996	2
						RLLEGEDAHLSSSQFSSGSQSSR	3	0.9985	3
						TKYETELNLR	2	0.9934	1
						TKYETELNLR	3	0.821	1
						TMQNLEIELQSQLSMK	2	0.9986	3
						TMQNLEIELQSQLSMK	3	0.9023	2
						VTMQNLNDR	2	0.9989	1
						YCMQLAQIQEMIGSVEEQLAQLR	2	0.9986	1
						YCMQLAQIQEMIGSVEEQLAQLR	3	0.9981	2
Q$545-1	P02538	K2C6A_HUMAN	KRT6A	Keratin, type II cytoskeletal 6A	1.0000	37.77	17	17	33
						ADTLTDEINFLR	2	0.9995	3
						AIGGGLSSVGGGSSTIK	2	0.9995	2
						ALYDAELSQMQTHISDTSVVLSMDNNR	3	0.9992	3
						DVDAAYMNKVELQAK	2	0.9988	2
						EYQELMNVK	2	0.9948	1
						GSGGLGGACGGAGFGSR	2	0.9984	2
						ISIGGGSCAISGGYGSR	2	0.9994	2
						LLKEYQELMNVK	2	0.9981	3
						NKLEGLEDALQK	3	0.9118	1
						QCANLQAAIADAEQR	2	0.9973	2
						QNLEPLFEQYINNLR	2	0.9977	3
						RQLDSIVGER	2	0.9846	1
						SRAEAESWYQTK	2	0.9945	2
						SRAEAESWYQTKYEELQVTAGR	3	0.9983	2
						TAAENEFVTLKK	2	0.9973	1
						WTLLQEQGTK	2	0.9985	1
						YEELQVTAGR	2	0.9991	2
Q$546-1	P02545-6	LMNA_HUMAN	LMNA	Isoform 6 of Prelamin-A/C	1.0000	15.15	9	9	10
Q$546-2	P02545-2	LMNA_HUMAN	LMNA	Isoform C of Prelamin-A/C	1.0000	16.26	9	9	10
						AAYEAELGDAR	2	0.9995	1
						EAALSTALSEKR	2	0.9838	1
						EGDLIAAQAR	2	0.9861	1
						ITESEEVVSR	2	0.9994	1
						LADALQELR	2	0.9986	1
						LAVYIDR	2	0.9977	1
						SGAQASSTPLSPTR	2	0.991	1
						SLETENAGLR	2	0.9971	1
						TLEGELHDLR	2	0.9897	2
Q$547-1	P02545-3	LMNA_HUMAN	LMNA	Isoform ADelta10 of Prelamin-A/C	1.0000	18.93	10	10	11
Q$547-2	P02545	LMNA_HUMAN	LMNA	Prelamin-A/C	1.0000	18.07	10	10	11
						AAYEAELGDAR	2	0.9995	1
						ASASGSGAQVGGPISSGSSASSVTVTR	2	0.9159	1
						EAALSTALSEKR	2	0.9838	1
						EGDLIAAQAR	2	0.9861	1
						ITESEEVVSR	2	0.9994	1
						LADALQELR	2	0.9986	1
						LAVYIDR	2	0.9977	1
						SGAQASSTPLSPTR	2	0.991	1
						SLETENAGLR	2	0.9971	1
						TLEGELHDLR	2	0.9897	2
Q$548-1	P02647	APOA1_HUMAN	APOA1	Apolipoprotein A-I	1.0000	68.91	27	32	85
						AKPALEDLR	2	0.9982	1
						ATEHLSTLSEK	2	0.9997	3
						ATEHLSTLSEK	3	0.9958	1
						DLATVYVDVLK	2	0.9997	3
						DLATVYVDVLKDSGR	2	0.9995	6
						DLATVYVDVLKDSGRDYVSQFEGSALGK	3	0.999	6
						DSGRDYVSQFEGSALGK	2	0.9994	2

						DSGRDYVSQFEGSALGK	3	0.9977	2
						DYVSQFEGSALGK	2	0.9997	3
						EQLGPVTQEFWDNLEK	2	0.9997	5
						EQLGPVTQEFWDNLEKETEGLR	3	0.9256	1
						EQLGPVTQEFWDNLEKETEGLRQEMSK	3	0.9019	1
						LEALKENGGAR	2	0.9992	2
						LLDNWDSVTSTFSK	2	0.9997	8
						LREQLGPVTQEFWDNLEK	3	0.9989	4
						LREQLGPVTQEFWDNLEKETEGLR	3	0.9976	2
						LREQLGPVTQEFWDNLEKETEGLR	4	0.9899	1
						LSPLGEEMR	2	0.9921	1
						QGLLPVLESF	2	0.9692	2
						QGLLPVLESFK	2	0.9997	3
						QGLLPVLESFKVSFLSALEEYTK	3	0.983	2
						QKVEPLRAELQEGAR	2	0.9343	3
						SFLSALEEYTK	2	0.9578	1
						THLAPYSDELR	2	0.9997	4
						THLAPYSDELR	3	0.9996	1
						THLAPYSDELRQ	2	0.983	2
						VKDLATVYVDVLK	2	0.9989	4
						VQPYLDDFQKK	2	0.9969	1
						VSFLSALEEYTK	2	0.9997	4
						VSFLSALEEYTKK	2	0.9993	2
						VSFLSALEEYTKK	3	0.9847	2
						WQEEMELYR	2	0.9946	2
Q$549-1	P02649	APOE_HUMAN	APOE	Apolipoprotein E	1.0000	65.93	20	22	42
						AATVGSLAGQPLQER	2	0.9996	3
						AKLEEQAQQIR	2	0.9993	4
						AYKSELEEQLTPVAEETR	2	0.999	1
						AYKSELEEQLTPVAEETR	3	0.9653	2
						DRLDEVKEQVAEVR	2	0.9971	2
						DRLDEVKEQVAEVR	3	0.9956	2
						GEVQAMLGQSTEELR	2	0.9974	2
						KVEQAVETEPEPELR	2	0.9929	2
						LAVYQAGAR	2	0.9993	1
						LDEVKEQVAEVR	2	0.9986	1
						LEEQAQQIR	2	0.9924	2
						LGADMEDVCGR	2	0.9991	1
						LGPLVEQGR	2	0.9995	1
						LKSWFEPLVEDMQR	3	0.9981	2
						LQAEAFQAR	2	0.9993	1
						LSKELQAAQAR	2	0.9607	1
						QQTEWQSGQR	2	0.9897	1
						QWAGLVEK	2	0.9229	1
						SELEEQLTPVAEETR	2	0.9997	2
						SWFEPLVEDMQR	2	0.9997	2
						VQAAVGTSAAPVPSDNH	2	0.9997	4
						WVQTLSEQVQEELLSSQVTQELR	3	0.9995	4
Q$550-1	P02652	APOA2_HUMAN	APOA2	Apolipoprotein A-II	1.0000	69	7	9	22
Q$550-2	V9GYM3	V9GYM3_HUMAN	APOA2	Apolipoprotein A-II	1.0000	51.88	7	9	22
						AGTELVNFLSYFVELGTQPATQ	3	0.9994	3
						EPCVESLVSQYFQTVTDYGK	2	0.9997	5
						EPCVESLVSQYFQTVTDYGK	3	0.9981	3
						EPCVESLVSQYFQTVTDYGKDLMEK	3	0.9769	2
						SKEQLTPLIK	2	0.9958	1
						SKEQLTPLIKK	2	0.9656	1
						SPELQAEAK	2	0.9997	1
						VKSPELQAEAK	2	0.9994	4
						VKSPELQAEAK	3	0.9979	2
Q$551-1	P02671	FIBA_HUMAN	FGA	Fibrinogen alpha chain	1.0000	42.84	38	48	153
						ADSGEGDFLAEGGGVR	2	0.9928	2
						AQLVDMK	2	0.9993	1
						DCDDVLQTHPSG	2	0.9915	1
						DCDDVLQTHPSGTQ	2	0.9097	2
						DCDDVLQTHPSGTQSGIFNIK	2	0.9997	2
						DCDDVLQTHPSGTQSGIFNIK	3	0.9991	4
						DCDDVLQTHPSGTQSGIFNIKLPGSSK	3	0.9993	3
						DQETSLGGWLLIQQR	2	0.9374	1
						DSDWPFCSDEDWNYK	2	0.9997	4
						DSHSLTTNIMEILR	3	0.9706	1
						EVDLKDYEDQQK	2	0.9915	2
						EVVTSEDGSDCPEAMDLGTLSGIGTLDGFR	3	0.9699	1
						GADYSLR	2	0.9939	1
						GFGSLNDEGEGEFWLGNDYLHLLTQR	3	0.9997	16
						GGSTSYGTGSETESPR	2	0.9997	4
						GLIDEVNQDFTNR	2	0.9998	10
						GLIDEVNQDFTNR	3	0.9997	3
						GLIDEVNQDFTNRINK	2	0.9984	2
						GSESGIFTNTK	2	0.9992	1
						GSVLRVELEDWAGNEAYAEYHFR	3	0.9993	4
						GSVLRVELEDWAGNEAYAEYHFR	4	0.8614	1
						HQSACKDSDWPFCSDEDWNYK	3	0.9994	3
						HRHPDEAAFFDTASTGK	3	0.9956	4
						IFSVYCDQETSLGGWLLIQQR	2	0.9997	5
						IFSVYCDQETSLGGWLLIQQR	3	0.9996	12
						IRPLVTQ	2	0.9997	2
						LEVDIDIK	2	0.9994	4
						MELERPGGNEITR	2	0.9974	2
						MKGLIDEVNQDF	2	0.9055	2
						MKGLIDEVNQDFTNR	2	0.9994	2
						MKGLIDEVNQDFTNR	3	0.9992	2
						NNSPYEIENGVVWVSFR	2	0.9998	11
						NNSPYEIENGVVWVSFR	3	0.9993	4
						NSLFEYQK	2	0.9943	2
						PFCSDEDWNYK	2	0.9608	2
						QLEQVIAK	2	0.9107	1
						RGFGSLNDEGEGEFWLGNDYLHLLTQR	3	0.9996	4
						RGFGSLNDEGEGEFWLGNDYLHLLTQR	4	0.9987	3
						RLEVDIDIK	2	0.9986	1
						RLEVDIDIK	3	0.9963	1
						SPYEIENGVVWVSFR	2	0.8368	1
						TFPGFFSPMLGEFVSETESR	2	0.9982	3
						VELEDWAGNEAYAEYHFR	2	0.9997	3
						VELEDWAGNEAYAEYHFR	3	0.9997	7
						VGSEAEGYALQVS	2	0.9711	1
						VQHIQLLQK	2	0.995	1
						VQHIQLLQK	3	0.9148	1
						WPFCSDEDWNYK	2	0.9931	3
Q$552-1	P02675	FIBB_HUMAN	FGB	Fibrinogen beta chain	1.0000	68.43	56	64	149
						AHYGGFTVQNEANK	2	0.9997	3
						AHYGGFTVQNEANKY	2	0.9869	1
						AHYGGFTVQNEANKYQISVNK	2	0.9995	2
						AHYGGFTVQNEANKYQISVNK	3	0.9993	2
						APDAGGCLHAD	2	0.8727	1
						APDAGGCLHADPDLGVLCPTGCQLQEALLQQERP	3	0.9924	3
						APDAGGCLHADPDLGVLCPTGCQLQEALLQQERPI	3	0.9556	2
						APDAGGCLHADPDLGVLCPTGCQLQEALLQQERPIR	4	0.999	4
						DETVNSNIPTNLR	2	0.8911	1
						DMNTENGGWTVIQNR	2	0.9944	2
						DNENVVNEYSSELEK	2	0.9997	3
						ECEEIIR	2	0.9964	1
						ECEEIIRK	2	0.9899	1
						EDGGGWWYNR	2	0.9992	4

						EEAPSLRPAPPPISGGGYR	3	0.99	1
						GGETSEMYLIQPDSSVKPYR	3	0.9926	2
						HGTDDGVVWMNWK	2	0.9993	4
						HGTDDGVVWMNWK	3	0.9751	3
						HQLYIDETVNSNIPTNLR	2	0.9997	1
						HQLYIDETVNSNIPTNLR	3	0.9992	2
						IDETVNSNIPTNLR	2	0.9815	3
						IQKLESDVSAQMEYCR	2	0.9996	2
						IQKLESDVSAQMEYCR	3	0.985	1
						IRPFFPQ	2	0.9798	2
						IRPFFPQQ	2	0.9994	3
						KAPDAGGCLHADPD	2	0.9223	1
						KAPDAGGCLHADPDLGVLCPTGCQLQE	3	0.9861	4
						KAPDAGGCLHADPDLGVLCPTGCQLQEA	3	0.8183	2
						KAPDAGGCLHADPDLGVLCPTGCQLQEAL	3	0.9828	3
						KAPDAGGCLHADPDLGVLCPTGCQLQEALLQQE	3	0.8677	1
						KAPDAGGCLHADPDLGVLCPTGCQLQEALLQQERP	3	0.9916	3
						KAPDAGGCLHADPDLGVLCPTGCQLQEALLQQERPI	3	0.9532	1
						KAPDAGGCLHADPDLGVLCPTGCQLQEALLQQERPIR	4	0.9993	7
						KGGETSEMYLIQPDSSVKPYR	3	0.9996	5
						KGGETSEMYLIQPDSSVKPYR	4	0.9977	1
						KREEAPSLRPAPPPISGGGYR	3	0.988	1
						KWDPYKQGFGNVATNTDGK	3	0.972	1
						LESDVSAQMEYCR	2	0.9997	3
						MGPTELLIEMEDWK	2	0.9997	6
						MGPTELLIEMEDWK	3	0.9994	2
						NSVDELNNNVEA	2	0.9283	1
						NSVDELNNNVEAVSQTS	2	0.9765	2
						NSVDELNNNVEAVSQTSSSS	2	0.9929	1
						NSVDELNNNVEAVSQTSSSSF	2	0.9809	1
						NSVDELNNNVEAVSQTSSSSFQYMYLLK	3	0.9996	4
						NYCGLPGEYWLGNDK	2	0.9996	4
						NYCGLPGEYWLGNDKISQLTR	2	0.9979	1
						NYCGLPGEYWLGNDKISQLTR	3	0.9971	1
						QDGSVDFGR	2	0.9056	1
						QGFGNVATNTDGK	2	0.9997	3
						QLYIDETVNSNIPTNLR	2	0.9851	1
						QVKDNENVVNEYSSELEK	3	0.8346	1
						REEAPSLRPAPPPISGGGYR	3	0.9993	3
						SILENLR	2	0.9991	2
						SKIQKLESDVSAQMEYCR	3	0.9992	3
						TPCTVSCNIPVVSGK	2	0.9997	7
						TPCTVSCNIPVVSGKECEEIIR	3	0.9993	3
						TPCTVSCNIPVVSGKECEEIIRK	3	0.8241	1
						TVSCNIPVVSGK	2	0.9488	1
						VYCDMNTENGGWTVIQNR	2	0.9997	5
						VYCDMNTENGGWTVIQNR	3	0.9925	2
						YIDETVNSNIPTNLR	2	0.8772	1
						YQISVNK	2	0.9975	1
						YYWGGQYTWDMAK	2	0.9997	4
Q$553-1	P02679	FIBG_HUMAN	FGG	Fibrinogen gamma chain	1.0000	54.08	47	53	120
Q$553-2	P02679-2	FIBG_HUMAN	FGG	Isoform Gamma-A of Fibrinogen gamma chain	1.0000	56.06	47	53	120
						AIQLTYNPDESSKP	2	0.936	1
						AIQLTYNPDESSKPNMIDAATLK	3	0.9968	2
						ALRVELEDWNGR	3	0.9007	1
						ASILTHDSSIR	2	0.9082	1
						ASTPNGYDNGIIWATWK	2	0.9971	4
						CHAGHLNGVYYQGGTYSK	3	0.9979	3
						DNCCILDER	2	0.9997	5
						DTVQIHDITGK	2	0.999	1
						EASILTHDSSIR	2	0.843	1
						EGFGHLSPTGTTEFWLGNEK	2	0.9997	4
						EGFGHLSPTGTTEFWLGNEK	3	0.9996	4
						FGSYCPTTCGIADFL	2	0.9461	2
						FGSYCPTTCGIADFLSTY	2	0.9962	2
						FGSYCPTTCGIADFLSTYQ	2	0.9834	2
						FGSYCPTTCGIADFLSTYQT	2	0.9978	2
						FGSYCPTTCGIADFLSTYQTK	2	0.9998	7
						FGSYCPTTCGIADFLSTYQTK	3	0.9996	4
						FGSYCPTTCGIADFLSTYQTKV	2	0.9937	2
						GIADFLSTYQTK	2	0.9958	2
						IHLISTQ	2	0.976	1
						IHLISTQSAIPYALR	2	0.9997	4
						IHLISTQSAIPYALR	3	0.9995	2
						IHLISTQSAIPYALRV	2	0.9677	1
						IHLISTQSAIPYALRVELEDWNGR	3	0.997	5
						KEGFGHLSPTGTTEFWLGNEK	3	0.9592	2
						KMLEEIMKYEASILTHDSSIR	3	0.9918	2
						LDGSVDFK	2	0.9988	4
						LDGSVDFKK	2	0.9991	2
						LQEIYNSNNQK	2	0.9914	1
						LSPTGTTEFWLGNEK	2	0.9199	1
						LTIGEGQQHHLGGAK	2	0.9994	2
						MLEEIMKYEASILTHDSSIR	3	0.9988	3
						QSGLYFIKPLK	2	0.9997	2
						QSGLYFIKPLK	3	0.9664	1
						RLDGSVDFK	2	0.9992	2
						RVELEDWNGR	2	0.9422	1
						RYLQEIYNSNNQK	2	0.8573	1
						SILTHDSSIR	2	0.9949	4
						SPTGTTEFWLGNEK	2	0.9861	3
						STQSAIPYALR	2	0.9526	1
						TGTTEFWLGNEK	2	0.9087	1
						TSTADYAMFK	2	0.9992	1
						VAQLEAQCQEPCK	2	0.9997	2
						VAQLEAQCQEPCKD	2	0.9969	2
						VAQLEAQCQEPCKDT	2	0.9829	2
						VAQLEAQCQEPCKDTV	2	0.9777	2
						VAQLEAQCQEPCKDTVQIHDITGK	3	0.9987	3
						VAQLEAQCQEPCKDTVQIHDITGKDCQDIANK	3	0.9982	1
						VAQLEAQCQEPCKDTVQIHDITGKDCQDIANK	4	0.8362	1
						VELEDWNGR	2	0.9996	2
						YEASILTHDSSIR	2	0.9997	4
						YEASILTHDSSIR	3	0.9956	1
						YLQEIYNSNNQK	2	0.9997	3
Q$554-1	P02730	B3AT_HUMAN	SLC4A1	Band 3 anion transport protein	1.0000	6.48	5	6	14
						AAATLMSER	2	0.9638	1
						ADFLEQPVLGFVR	2	0.9996	2
						FLFVLLGPEAPHIDYTQLGR	3	0.9996	3
						LQEAAELEAVELPVPIR	2	0.9997	4
						LQEAAELEAVELPVPIR	3	0.9914	1
						LQEAAELEAVELPVPIRF	2	0.9938	3
Q$555-1	P02743	SAMP_HUMAN	APCS	Serum amyloid P-component	1.0000	40.36	9	9	16
						AYSLFSYNTQGR	2	0.9996	3
						DNELLVYK	2	0.9988	1
						DNELLVYKER	2	0.964	1
						GYVIIKPLVWV	2	0.9997	4
						IVLGQEQDSYGGKFDR	2	0.9994	2
						NILDWQALNYEIR	2	0.8979	1
						QGYFVEAQPK	2	0.9965	1
						RAYSDLSR	2	0.9379	1
						VGEYSLYIGR	2	0.9997	2
Q$556-1	P02745	C1QA_HUMAN	C1QA	Complement C1q subcomponent subunit A	0.9997	13.47	3	3	4

						GHIYQGSEADSVFSGFLIFPSA	2	0.8959	2
						KGHIYQGSEADSVFSGFLIFPSA	3	0.9476	1
						SLGFCDTTNK	2	0.997	1
Q$557-1	P02747	C1QC_HUMAN	C1QC	Complement C1q subcomponent subunit C	0.9239	4.08	1	1	1
						VVTFCGHTSK	2	0.9239	1
Q$558-1	P02748	CO9_HUMAN	C9	Complement component C9	1.0000	33.63	16	19	38
						AIEDYINEFSVR	2	0.9997	3
						CLCACPFKFEGIACEISK	2	0.9775	1
						CLCACPFKFEGIACEISK	3	0.9898	1
						DRDGNTLTYYR	2	0.985	1
						DRVVEESELAR	2	0.9904	2
						DVVLTTTFVDDIK	2	0.9994	4
						GEYFAFLETYGTHY	2	0.963	2
						GTVIDVTDFVNWASSINDAPVLISQK	3	0.9697	1
						ISEGLPALEFPNE	2	0.8931	1
						KYAFELK	2	0.9856	1
						LSPIYNLVPVK	2	0.9882	2
						NFRTEHYEEQIEAFK	3	0.9893	2
						RPWNVASLIYETK	2	0.9994	4
						RPWNVASLIYETK	3	0.9994	3
						SENLIDDVVSLIR	2	0.9656	2
						TEHYEEQIEAFK	2	0.9997	3
						TEHYEEQIEAFK	3	0.9868	2
						TSNFNAAISLK	2	0.9997	2
						VVEESELAR	2	0.9894	1
Q$559-1	Q5VY30	Q5VY30_HUMAN	RBP4	Retinol binding protein 4, plasma, isoform CRA_b	1.0000	20.1	4	4	9
Q$559-2	P02753	RET4_HUMAN	RBP4	Retinol-binding protein 4	1.0000	19.9	4	4	9
						LIVHNGYCDGR	2	0.9885	1
						LLNLDGTCADSYSFVFSR	2	0.9996	5
						LLNLDGTCADSYSFVFSRD	2	0.9916	1
						YWGVASFLQK	2	0.9983	2
Q$560-1	P02760	AMBP_HUMAN	AMBP	Protein AMBP	1.0000	35.23	13	15	34
						AFIQLWAFDAVK	2	0.9996	3
						ETLLQDFR	2	0.9997	2
						GECVPGEQEPEPILIPR	2	0.9997	2
						GVCEETSGAYEK	2	0.9997	1
						GVCEETSGAYEKTDTDGK	2	0.9997	3
						KGVCEETSGAYEK	2	0.9997	4
						KGVCEETSGAYEKTDTDGK	2	0.999	1
						KGVCEETSGAYEKTDTDGK	3	0.9973	3
						MTVSTLVLGEGATEAEISMTSTR	2	0.9997	3
						TVAACNLPIVR	2	0.9917	2
						VLGEGATEAEISMTSTR	2	0.9938	1
						VVAQGVGIPEDSIFTMADRGECVPGEQEPEPILIPR	3	0.9871	2
						WYNLAIGSTCPWLK	2	0.9997	4
						WYNLAIGSTCPWLK	3	0.9278	1
						WYNLAIGSTCPWLKK	2	0.996	2
Q$561-1	P02763	A1AG1_HUMAN	ORM1	Alpha-1-acid glycoprotein 1	1.0000	19.4	4	4	8
						EQLGEFYEALDCLR	2	0.9995	4
						SDVVYTDWK	2	0.9954	2
						SDVVYTDWKK	2	0.9671	1
						YVGGQEHFAHLLILR	2	0.9987	1
Q$562-1	P02766	TTHY_HUMAN	TTR	Transthyretin	1.0000	53.74	5	5	9
						AADDTWEPFASGK	2	0.9995	1
						GSPAINVAVHVFR	2	0.9821	2
						KAADDTWEPFASGK	2	0.964	1
						RYTIAALLSPYSYSTTAVVTNPKE	3	0.9981	2
						TSESGELHGLTTEEEFVEGIYKVEIDTK	3	0.9978	3
Q$563-1	P02768	ALBU_HUMAN	ALB	Serum albumin	1.0000	70.28	61	74	149
						AACLLPK	2	0.9888	1
						AAFTECCQAADK	2	0.9996	1
						ADDKETCFAEEGK	2	0.9987	3
						ADDKETCFAEEGKK	2	0.9995	6
						ADDKETCFAEEGKK	3	0.9983	2
						AFAQYLQQCPFEDHVK	3	0.9005	1
						ALVLIAFAQYLQQCPFEDHVK	2	0.9997	5
						ALVLIAFAQYLQQCPFEDHVK	3	0.9997	11
						ALVLIAFAQYLQQCPFEDHVK	4	0.999	2
						AQYLQQCPFEDHVK	2	0.9911	4
						AQYLQQCPFEDHVK	3	0.9074	1
						AVMDDFAAFVEK	2	0.9998	3
						AVMDDFAAFVEK	3	0.9938	1
						CAAADPHECYAK	2	0.9868	2
						CCAAADPHECYA	2	0.8159	1
						CCAAADPHECYAK	3	0.9913	1
						CCTESLVNR	2	0.9973	1
						CTESLVNR	2	0.8874	1
						DLGEENFK	2	0.9792	1
						DVCKNYAEAK	2	0.8516	1
						DVFLGMFLYEYAR	2	0.9997	5
						ECCEKPLLEK	2	0.9258	1
						EFNAETFTFHADICTLSEK	2	0.9993	1
						EFNAETFTFHADICTLSEKER	3	0.9753	1
						FKDLGEENFK	2	0.988	1
						FKDLGEENFK	3	0.9581	1
						FQNALLVR	2	0.9986	1
						FSALEVDETYVPK	2	0.8615	1
						FYAPELLFFAK	2	0.9695	2
						HPYFYAPELLFFAK	2	0.9785	2
						HPYFYAPELLFFAK	3	0.9988	4
						KLVAASQAALGL	2	0.992	1
						KQTALVELVK	2	0.956	1
						KQTALVELVK	3	0.9953	1
						KVPQVSTPTLVEV	2	0.9522	1
						KVPQVSTPTLVEVSR	3	0.9997	2
						KYLYEIAR	2	0.8126	1
						LCTVATLR	2	0.9964	1
						LDELRDEGK	2	0.9476	1
						LKECCEKPLLEK	3	0.9097	1
						LVAASQAALGL	2	0.9991	3
						LVNEVTEFAK	2	0.9997	3
						LVRPEVDVMCTAFHDNEETFLKK	3	0.8761	1
						LVTDLTK	2	0.9647	1
						MPCAEDYLSVVLNQLCVLHEK	3	0.9986	2
						QNCELFEQLGEYK	2	0.9997	3
						QNCELFEQLGEYKFQNALLVR	3	0.9976	2
						QTALVELVK	2	0.9727	1
						QYLQQCPFEDHVK	2	0.9814	1
						RHPDYSVVLLLR	2	0.9993	4
						RHPDYSVVLLLR	3	0.9996	5
						RHPYFYAPELLFFAK	2	0.9984	2
						RHPYFYAPELLFFAK	4	0.9961	2
						RMPCAEDYLSVVLNQLCVLHEK	3	0.9858	2
						RPCFSAL	2	0.9674	1
						RPCFSALEVDETYVPK	2	0.9984	3
						RPCFSALEVDETYVPK	3	0.9996	2
						SHCIAEVENDEMPADLPSLAADFVESK	3	0.9993	3
						SLHTLFGDK	2	0.999	1
						SLHTLFGDKLCTVAT	2	0.8361	1
						SLHTLFGDKLCTVATLR	2	0.9995	3
						SLHTLFGDKLCTVATLR	3	0.9994	1
						TCVADESAENCDK	2	0.9997	3
						TCVADESAENCDKSLHTLFGDK	3	0.9489	1

						VFDEFKPL	2	0.8356	1
						VFDEFKPLVEEPQ	2	0.8081	1
						VFDEFKPLVEEPQN	2	0.8802	1
						VFDEFKPLVEEPQNL	2	0.9592	2
						VFDEFKPLVEEPQNLIK	2	0.9997	4
						VFDEFKPLVEEPQNLIK	3	0.9997	3
						VHTECCHGDLLECADDR	3	0.9991	2
						VHTECCHGDLLECADDRADLAK	3	0.9047	1
						VPQVSTPTLVEVSR	2	0.9993	3
						YLYEIAR	2	0.9981	1
Q$564-1	P02787	TRFE_HUMAN	TF	Serotransferrin	1.0000	31.23	23	26	58
						CDEWSVNSVGK	2	0.9952	1
						CLVEKGDVAFVK	2	0.9692	2
						CSTSSLLEACTFR	2	0.9996	3
						DGAGDVAFVK	2	0.9686	1
						DLLFRDDTVCLAK	2	0.9735	1
						EGTCPEAPTDECKPV	2	0.9602	3
						EGTCPEAPTDECKPVK	2	0.9993	2
						EGYYGYTGAFR	2	0.9993	2
						FDEFFSEGCAPGSK	2	0.9996	4
						GLLYCDLPEPR	2	0.9883	3
						HSTIFENLANK	2	0.9328	1
						IECVSAETTEDCIAK	2	0.9996	4
						KCSTSSLLEACTFR	2	0.9993	4
						KPVEEYANCHLAR	2	0.9996	3
						KPVEEYANCHLAR	3	0.9959	1
						KPVEEYANCHLAR	4	0.9582	1
						LKCDEWSVNSVGK	2	0.9656	1
						MYLGYEYVTAIR	2	0.9995	3
						SAGWNIPIGLLYCDLPEPR	2	0.9996	4
						SAGWNIPIGLLYCDLPEPR	3	0.9205	2
						SASDLTWDNLK	2	0.9989	3
						SVIPSDGPSVACVK	2	0.9739	1
						WCALSHHER	2	0.9996	4
						WCAVSEHEATK	2	0.9988	1
						YCDLPEPR	2	0.9152	1
						YYGYTGAFR	2	0.9983	2
Q$565-1	P02788-2	TRFL_HUMAN	LTF	Isoform DeltaLf of Lactotransferrin	1.0000	7.51	4	4	14
						CLAENAGDVAFVK	2	0.9995	3
						LADFALLCLDGK	2	0.9995	3
						NLLFNDNTECLAR	2	0.9994	4
						VVWCAVGEQELR	2	0.9995	4
Q$566-1	P02790	HEMO_HUMAN	HPX	Hemopexin	1.0000	17.97	10	12	22
						CSPHLVL	2	0.8998	1
						DSVDAAFICPGSSR	2	0.9756	2
						EVGTPHGIILDSVDAAFICPGSSR	3	0.9957	2
						GGYTLVSGYPK	2	0.9996	2
						LHIMAGR	2	0.9845	1
						LWWLDLK	2	0.9974	1
						LYLVQGTQVYVFLTK	2	0.9996	4
						LYLVQGTQVYVFLTK	3	0.9994	3
						RLWWLDLK	2	0.9869	1
						RLWWLDLK	3	0.9952	2
						SVDAAFICPGSSR	2	0.944	1
						YYCFQGNQFLR	2	0.9995	2
Q$567-1	P04003	C4BPA_HUMAN	C4BPA	C4b-binding protein alpha chain	1.0000	44.89	39	51	125
						CEWETPEGCEQVLTGK	2	0.9997	4
						CEWETPEGCEQVLTGKR	2	0.9997	3
						CEWETPEGCEQVLTGKR	3	0.9973	2
						CHPGYKPTTDEPTTV	2	0.9934	2
						CHPGYKPTTDEPTTVICQK	2	0.9997	2
						CHPGYKPTTDEPTTVICQK	3	0.9995	3
						EDVYVVGTVLR	2	0.9997	2
						FSAICQGDGTWSPR	2	0.9998	5
						FSAICQGDGTWSPR	3	0.9192	1
						FSLLGHASI	2	0.9739	2
						GDEISFSCHETSR	2	0.8778	1
						GVGWSHPLPQCEIVK	2	0.9996	2
						GVGWSHPLPQCEIVK	3	0.9982	1
						GYILVGQAK	2	0.9997	1
						HSGEENFYAYGFSV	2	0.9147	1
						HSGEENFYAYGFSVTY	2	0.9878	3
						HSGEENFYAYGFSVTYSCDPR	3	0.9996	3
						KEDVYVVGTVLR	2	0.808	1
						KEEIIYECDKGYILVGQAK	3	0.9116	2
						KPDVSHGEMVSGFGPIYNYK	3	0.9996	3
						KPDVSHGEMVSGFGPIYNYKDTIVFK	3	0.9974	1
						KPDVSHGEMVSGFGPIYNYKDTIVFK	4	0.9838	3
						LMQCLPNPEDVK	2	0.9997	5
						LSCSYSHWSAPAPQCK	2	0.9997	3
						LSCSYSHWSAPAPQCK	3	0.9962	1
						LSLEIEQLELQR	2	0.9997	7
						LSLEIEQLELQR	3	0.9997	3
						LSLEIEQLELQRD	2	0.9955	3
						LSLEIEQLELQRD	3	0.9017	1
						LSLEIEQLELQRDS	2	0.9873	2
						LSLEIEQLELQRDSA	2	0.9569	2
						LSLEIEQLELQRDSAR	2	0.9583	1
						LSLEIEQLELQRDSAR	3	0.9994	2
						MALEVYK	2	0.9996	2
						MALEVYKL	2	0.8233	1
						MALEVYKLSLEIEQLELQR	2	0.9961	1
						MALEVYKLSLEIEQLELQR	3	0.9993	4
						QSSSYSFFKEEIIYECDKGYILVGQAK	3	0.9957	5
						RLMQCLPNPEDVK	2	0.9893	3
						SAICQGDGTWSPR	2	0.9802	2
						SGFGPIYNYK	2	0.984	1
						SLEIEQLELQR	2	0.991	2
						SRPANHCVYFYGDEISFSCHETSR	3	0.9986	1
						SYSHWSAPAPQCK	2	0.9922	3
						TWYPEVPK	2	0.9997	2
						TWYPEVPKCEWETPEGCEQVLTGK	3	0.9995	5
						TWYPEVPKCEWETPEGCEQVLTGKR	3	0.9994	6
						TWYPEVPKCEWETPEGCEQVLTGKR	4	0.9744	1
						WTPYQGCEALCCPEPK	2	0.9997	4
						WTPYQGCEALCCPEPK	3	0.9988	2
						YTCLPGYVR	2	0.9997	2
Q$568-1	P04004	VTNC_HUMAN	VTN	Vitronectin	1.0000	49.79	34	38	104
						AQYWLGCPAPGHL	2	0.971	2
						CQCDELCSYYQSC	2	0.9731	2
						CQCDELCSYYQSCCTDYTAECKPQ	3	0.9796	1
						CQCDELCSYYQSCCTDYTAECKPQVTR	3	0.9992	3
						CTEGFNVDKK	2	0.9997	6
						CTEGFNVDKK	3	0.9989	1
						DGVLDPDYPR	2	0.9833	1
						DSWEDIFELLFWGR	3	0.9994	3
						DVWGIEGPIDAAFTR	2	0.9998	17
						DVWGIEGPIDAAFTR	3	0.9997	3
						DVWGIEGPIDAAFTRI	2	0.9513	2
						DWHGVPGQVDAAMAGR	2	0.9997	4
						DWHGVPGQVDAAMAGR	3	0.9997	3
						ERVYFFK	2	0.9592	1
						FEDGVLDPDYPR	2	0.9997	10

						FFFSGDKYYR	2	0.9131	2
						GNPEQTPVLKPEEEAPAPEVGASKPEG	3	0.9825	2
						GQYCYELDEK	2	0.9997	4
						GSQYWRFEDGVLDPDYPR	3	0.9969	3
						IEGPIDAAFTR	2	0.9117	1
						MDWLVPATCEPIQSVFFFSGDK	2	0.9996	2
						MDWLVPATCEPIQSVFFFSGDKYYR	3	0.9874	1
						QYWEYQFQHQPSQEECEGS	2	0.9967	1
						QYWEYQFQHQPSQEECEGSS	2	0.953	1
						QYWEYQFQHQPSQEECEGSSL	2	0.9004	1
						QYWEYQFQHQPSQEECEGSSLSAVFEHF	3	0.9353	1
						RDVWGIEGPIDAAFTR	3	0.9594	2
						RVDTVDPPYPR	2	0.9997	3
						RVDTVDPPYPR	3	0.9996	3
						SIAQYWLGCPAPG	2	0.9985	1
						SIAQYWLGCPAPGH	2	0.9981	3
						SIAQYWLGCPAPGHL	3	0.9996	4
						TCEPIQSVFFFSGDK	2	0.9713	2
						TCEPIQSVFFFSGDKYYR	2	0.9664	1
						TSAGTRQPQFISR	2	0.9559	2
						VDTVDPPYPR	2	0.9984	2
						VWGIEGPIDAAFTR	2	0.9674	1
						WGIEGPIDAAFTR	2	0.8911	2
Q$569-1	P04040	CATA_HUMAN	CAT	Catalase	1.0000	12.9	4	4	8
						ADVLTTGAGNPVGDKLNVITVGPR	3	0.9956	3
						AFYVNVLNEEQR	2	0.9981	2
						FNTANDDNVTQVR	2	0.9995	1
						GPLLVQDVVFTDEMAHFDR	3	0.9994	2
Q$570-1	P04080	CYTB_HUMAN	CSTB	Cystatin-B	0.9986	24.49	2	2	2
						SQVVAGTNYFIK	2	0.961	1
						VHVGDEDFVHLR	3	0.9842	1
Q$571-1	P04114	APOB_HUMAN	APOB	Apolipoprotein B-100	1.0000	27.96	96	106	196
						AAIQALR	2	0.9919	1
						AALTELSLGSAYQAMILGVDSK	2	0.9852	1
						AASGTTGTYQEWK	2	0.9975	2
						ADSVVDLLSYNVQGSGETTYDHK	3	0.9707	1
						AEPLAFTFSHDYK	2	0.9478	1
						AHLDIAGSLEGHLR	3	0.9991	1
						ALVEQGFTVPEIK	2	0.9952	1
						ATFQTPDFIVPLTDLR	2	0.9985	3
						ATGVLYDYVNK	2	0.9992	2
						AVSMPSFSILGSDVR	2	0.997	2
						DAVEKPQEFTIVAFVK	3	0.9989	2
						DFSAEYEEDGKYEGLQEWEGK	3	0.9813	1
						DKDQEVLLQTFLDDASPGDKR	3	0.9957	3
						DKIGVELTGR	2	0.9861	1
						DKIGVELTGR	3	0.9825	1
						DLKVEDIPLAR	2	0.9361	1
						EFNLQNMGLPDFHIPENLFLK	3	0.9962	3
						EFQVPTFTIPK	2	0.9932	1
						ENFAGEATLQR	2	0.9995	1
						EVYGFNPEGK	2	0.9995	2
						EYSGTIASEANTYLNSK	2	0.9997	2
						FDHTNSLNIAGLSLDFSSK	3	0.9675	3
						FSVPAGIVIPSFQALTAR	2	0.9997	4
						FSVPAGIVIPSFQALTAR	3	0.9904	1
						FVTQAEGAK	2	0.9922	1
						GAYQNNEIK	2	0.96	1
						GFEPTLEALFGK	2	0.9997	3
						GIISALLVPPETEEAK	2	0.9969	2
						GIISALLVPPETEEAK	3	0.9849	1
						HIYAISSAALSASYK	2	0.9021	1
						IADFELPTIIVPEQTIEIPSIK	2	0.9997	4
						IADFELPTIIVPEQTIEIPSIK	3	0.999	3
						IAELSATAQEIIK	2	0.9987	1
						IDDIWNLEVK	2	0.9996	5
						IEGNLIFDPNNYLPK	2	0.9997	5
						IEIPLPFGGK	2	0.9987	1
						IGQDGISTSATTNLK	2	0.9995	2
						IGVELTGR	2	0.999	1
						IHSGSFQSQVELSNDQEK	3	0.9972	2
						ILGEELGFASLHDLQLLGK	3	0.9752	1
						INCKVELEVPQLCSFILK	3	0.9832	3
						ITENDIQIALDDAK	2	0.9996	2
						IYSLWEHSTK	2	0.9989	2
						KGNVATEISTER	2	0.9991	1
						KSISAALEHK	2	0.9935	2
						KYTYNYEAESSSGVPGTADSR	2	0.9997	2
						LAAYLMLMR	2	0.9995	1
						LAPGELTIIL	2	0.9612	1
						LDNIYSSDKFYK	2	0.9978	2
						LELELRPTGEIEQYSVSATYELQR	3	0.9993	3
						LELELRPTGEIEQYSVSATYELQREDR	4	0.9872	1
						LNTDIAGLASAIDMSTNYNSDSLHFSNVFR	3	0.9994	4
						LPYTIITTPPLKDFSLWEK	3	0.9412	2
						LSLESLTSYFSIESSTK	2	0.9997	2
						LSLESLTSYFSIESSTK	3	0.9754	1
						LSLPDFK	2	0.9983	1
						LTIFKTELR	2	0.9947	2
						LTLDIQNK	2	0.9992	1
						LTLDIQNKK	2	0.9966	1
						NFVASHIANILNSEELDIQDLK	3	0.9982	2
						NFVASHIANILNSEELDIQDLKK	3	0.9989	3
						NHLQLEGLFFTNGEHTSK	3	0.9798	1
						NLQNNAEWVYQGAIR	2	0.9997	2
						NLQNNAEWVYQGAIR	3	0.8835	1
						NMEVSVATTTK	2	0.9417	1
						NNALDFVTK	2	0.9941	1
						NSEEFAAAMSR	2	0.9991	2
						NSLKIEIPLPFGGK	3	0.9978	2
						NTASLKYENYELTLK	2	0.9778	1
						QSMTLSSEVQIPDFDVDLGTILR	2	0.9978	1
						QSMTLSSEVQIPDFDVDLGTILR	3	0.9806	2
						QTIIVVLENVQR	2	0.9986	2
						QTVNLQLQPYSLVTTLNSDLK	2	0.9979	3
						QVFLYPEKDEPTYILNIK	3	0.9865	1
						QVFLYPEKDEPTYILNIKR	3	0.9936	2
						SEILAHWSPAK	3	0.9477	1
						SEYQADYESLR	2	0.9997	2
						SISAALEHK	2	0.9969	1
						SKPTVSSSMEFK	2	0.99	2
						SPAFTDLHLR	2	0.9987	2
						SPAFTDLHLR	3	0.9996	1
						SVSDGIAALDLNAVANK	2	0.9997	5
						SVSLPSLDPASAK	2	0.9585	1
						TEHGSEMLFFGNAIEGK	3	0.9977	1
						TEVIPPLIENR	2	0.9965	2
						TGISPLALIK	2	0.9995	2
						TLADLTLLDSPIK	2	0.9995	1
						TQFNNNEYSQDLDAYNTK	2	0.9997	2
						TSSFALNLPTLPEVKFPEVDVLTK	3	0.9978	3
						TTLTAFGFASADLIEIGLEGK	2	0.9997	4
						VEKPQEFTIVAFVK	3	0.9829	2

						VELEVPQLCSFILK	2	0.9992	2
						VLADKFIIPGLK	3	0.994	2
						VLLDQLGTTISFER	2	0.9953	1
						VNWEEEAASGLLTSLK	2	0.9995	1
						VNWEEEAASGLLTSLK	3	0.9977	1
						VNWEEEAASGLLTSLKDNVPK	3	0.9981	4
						VPSYTLILPSLELPVLHVPR	3	0.9995	4
						VQGVEFSHR	2	0.9984	1
						VQGVEFSHR	3	0.9905	1
						VSALLTPAEQTGTWK	2	0.9991	2
						YDKNQDVHSINLPFFETLQEYFER	3	0.9719	1
						YEDGTLSLTSTSDLQSGIIK	2	0.9997	4
						YHWEHTGLTLR	3	0.9781	1
						YNALDLTNNGK	2	0.9782	2
						YTYNYEAESSSGVPGTADSR	2	0.9969	1
Q$572-1	P04196	HRG_HUMAN	HRG	Histidine-rich glycoprotein	1.0000	30.86	23	28	52
						ADLFYDVEALDLESPK	2	0.9998	4
						ADLFYDVEALDLESPK	3	0.9997	1
						ALDLINK	2	0.9977	1
						ALDLINKR	2	0.9993	1
						DGYLFQLLR	2	0.9997	5
						DSPVLIDFFEDTER	2	0.9998	7
						DSPVLIDFFEDTERYR	2	0.9612	1
						DVQESDCSVLSR	2	0.9711	2
						HPNVFGFCR	2	0.9963	1
						HPNVFGFCR	3	0.9983	1
						KYWNDCEPPDSR	2	0.9997	1
						KYWNDCEPPDSR	3	0.9578	1
						LVLDVQESDCSVLSR	2	0.9451	1
						NLVINCEVFDPQEH	2	0.9591	1
						NLVINCEVFDPQEHEN	2	0.9666	2
						NLVINCEVFDPQEHENI	2	0.9725	1
						RDGYLFQLLR	2	0.9612	1
						RDGYLFQLLR	3	0.9996	4
						RPSEIVIGQCK	2	0.9997	2
						RPSEIVIGQCK	3	0.9941	1
						RRDGYLFQLLR	3	0.9983	2
						SGFPQVSMFFTHTFPK	2	0.999	2
						SPVLIDFFEDTER	2	0.9857	2
						VRGGEGTGYFVDFSVR	2	0.9962	2
						YDVEALDLESPK	2	0.9847	2
						YKEENDDFASFR	3	0.9914	1
						YKEENDDFASFRVDR	2	0.9378	1
						YWNDCEPPDSR	2	0.9985	1
Q$573-1	P04217	A1BG_HUMAN	A1BG	Alpha-1B-glycoprotein	1.0000	19.39	8	8	19
						ALGDGGHYTCR	2	0.9754	1
						ATWSGAVLAGR	2	0.9996	3
						AVALGDGGHYTCR	2	0.9321	1
						CEGPIPDVTFELLR	2	0.9996	4
						GVTFLLR	2	0.9723	1
						SGLSTGWTQLSK	2	0.9824	2
						TPGAAANLELIFVGPQHAGNYR	3	0.9996	4
						VTLTCVAPLSGVDFQLR	2	0.9991	3
Q$574-1	P04217-2	A1BG_HUMAN	A1BG	Isoform 2 of Alpha-1B-glycoprotein	1.0000	19.03	5	5	15
						ATWSGAVLAGR	2	0.9996	3
						CEGPIPDVTFELLR	2	0.9996	4
						GVTFLLR	2	0.9723	1
						TPGAAANLELIFVGPQHAGNYR	3	0.9996	4
						VTLTCVAPLSGVDFQLR	2	0.9991	3
Q$575-1	P04259	K2C6B_HUMAN	KRT6B	Keratin, type II cytoskeletal 6B	1.0000	27.3	10	10	23
						ADTLTDEINFLR	2	0.9995	3
						ALYDAELSQMQTHISDTSVVLSMDNNR	3	0.9992	3
						DVDAAYMNKVELQAK	2	0.9988	2
						GSGGLGGACGGAGFGSR	2	0.9984	2
						ISIGGGSCAISGGYGSR	2	0.9994	2
						LLKEYQELMNVK	2	0.9981	3
						NKLEGLEDALQK	3	0.9118	1
						QCANLQAAIADAEQR	2	0.9973	2
						QNLEPLFEQYINNLR	2	0.9977	3
						SRAEAESWYQTK	2	0.9945	2
Q$576-1	P04264	K2C1_HUMAN	KRT1	Keratin, type II cytoskeletal 1	1.0000	69.1	41	54	116
						AEAESLYQSK	2	0.9989	1
						AEAESLYQSKYEELQITAGR	3	0.9575	1
						AQYEDIAQK	2	0.9995	1
						DVDGAYMTK	2	0.999	2
						DYQELMNTK	2	0.9988	1
						ELLQQVDTSTR	2	0.9816	1
						FLEQQNQVLQTK	2	0.9997	3
						FLEQQNQVLQTKWELLQQVDTSTR	3	0.9987	3
						GGGGGGYGSGGSSYGSGGGSYGSGGGGGGGR	2	0.9997	3
						GGGGGGYGSGGSSYGSGGGSYGSGGGGGGGR	3	0.9996	3
						GGGGGSFGAGGGFGSR	2	0.8341	1
						GGSGGGGGGSSGGRGSGGGSSGGSIGGRGSSSGGVK	3	0.9527	1
						GSYGSGGSSYGSGGGSYGSGGGGGGHGSYGSGSSSGGYR	3	0.9994	2
						KQISNLQQSISDAEQR	3	0.8333	1
						LDNLQQEIDFLTALYQAELSQMQTQISETNVILSMDNNR	4	0.9928	3
						LDSELKNMQDMVEDYR	3	0.8465	1
						LEQQNQVLQTK	2	0.9924	1
						LNDLEDALQQAK	2	0.9996	2
						LNDLEDALQQAKEDLAR	2	0.9826	1
						LNDLEDALQQAKEDLAR	3	0.8112	1
						MSGECAPNVSVSVSTSHTTISGGGSR	3	0.9996	3
						NKLNDLEDALQQAK	2	0.9986	2
						NKLNDLEDALQQAK	3	0.9959	1
						NKLNDLEDALQQAKEDLAR	2	0.9985	2
						NKLNDLEDALQQAKEDLAR	3	0.9991	3
						NMQDMVEDYR	2	0.9995	2
						NSKIEISELNR	2	0.9811	2
						NSKIEISELNR	3	0.9979	1
						QISNLQQSISDAEQR	2	0.9997	3
						QISNLQQSISDAEQR	3	0.9994	1
						SDQSRLDSELK	2	0.9963	1
						SGGGFSSGSAGIINYQR	2	0.9995	3
						SISISVAR	2	0.9747	1
						SKAEAESLYQSKYEELQITAGR	3	0.9993	4
						SLDLDSIIAEVK	2	0.9997	3
						SLDLDSIIAEVK	3	0.9954	2
						SLLQPLNVEIDPEIQK	2	0.9945	2
						SLLQPLNVEIDPEIQK	3	0.8668	2
						SLNNQFASFIDK	2	0.9996	1
						SLNNQFASFIDKVR	2	0.9989	3
						SLNNQFASFIDKVR	3	0.9881	2
						SLVNLGGSKSISISVAR	3	0.9376	2
						THNLEPYFESFINNLR	2	0.9995	6
						THNLEPYFESFINNLR	3	0.9997	5
						THNLEPYFESFINNLRR	2	0.8671	1
						THNLEPYFESFINNLRR	3	0.999	3
						THNLEPYFESFINNLRR	4	0.9962	4
						TINQSLLQPLNVEIDPEIQK	2	0.9925	3
						TLLEGEESR	2	0.999	2
						TNAENEFVTIK	2	0.9996	2
						TNAENEFVTIKK	2	0.9994	5
						TNAENEFVTIKK	3	0.9948	1

						WELLQQVDTSTR	2	0.9997	3
						YEELQITAGR	2	0.9997	2
Q$577-1	P04350	TBB4A_HUMAN	TUBB4A	Tubulin beta-4A chain	1.0000	7.66	3	3	8
						ALTVPELTQQMFDAK	2	0.9994	4
						GHYTEGAELVDAVLDVVR	3	0.9989	3
						GHYTEGAELVDAVLDVVRK	3	0.9891	1
Q$578-1	P04406	G3P_HUMAN	GAPDH	Glyceraldehyde-3-phosphate dehydrogenase	1.0000	65.67	24	29	84
						AGAEYVVESTGVFTTMEK	2	0.8445	1
						GALQNIIPASTGAAK	2	0.9997	3
						GILGYTEHQVVSSD	2	0.8054	1
						GILGYTEHQVVSSDFNSDTHSSTFDAGAGIALNDHFVK	4	0.9881	3
						IISNASCTTNCLAPLAK	2	0.9997	4
						IISNASCTTNCLAPLAK	3	0.8098	1
						ISWYDNEFGYSNR	2	0.9884	2
						LEKPAKYDDIK	2	0.9917	3
						LEKPAKYDDIKK	2	0.999	4
						LISWYDNEFGYSNR	2	0.9998	9
						LISWYDNEFGYSNR	3	0.9993	3
						LTGMAFR	2	0.9971	1
						LTGMAFRVPTANVSVVDLTCR	3	0.9994	3
						LVINGNPITIFQER	2	0.9996	2
						LVINGNPITIFQERD	2	0.9461	1
						LVINGNPITIFQERDPSK	2	0.995	1
						SWYDNEFGYSNR	2	0.9964	3
						VDIVAINDPFIDLNY	2	0.9381	1
						VDIVAINDPFIDLNYMVYMFQYDSTHGK	3	0.9949	3
						VIHDNFGIVEGLMTTV	2	0.9446	1
						VIHDNFGIVEGLMTTVHAI	3	0.9769	1
						VIHDNFGIVEGLMTTVHAITATQK	3	0.9996	11
						VIHDNFGIVEGLMTTVHAITATQK	4	0.9981	4
						VPTANVSVVDLTCR	2	0.9998	5
						VSVVDLTCR	2	0.8801	1
						VVDLMAHMASKE	2	0.9997	2
						VVDLMAHMASKE	3	0.9841	2
						WGDAGAEYVVESTGVFTTMEK	2	0.9997	5
						WGDAGAEYVVESTGVFTTMEK	3	0.9996	3
Q$579-1	P04406-2	G3P_HUMAN	GAPDH	Isoform 2 of Glyceraldehyde-3-phosphate dehydrogenase	1.0000	65.53	20	25	78
						AGAEYVVESTGVFTTMEK	2	0.8445	1
						GALQNIIPASTGAAK	2	0.9997	3
						GILGYTEHQVVSSDFNSDTHSSTFDAGAGIALNDHFVK	4	0.9881	3
						IISNASCTTNCLAPLAK	2	0.9997	4
						IISNASCTTNCLAPLAK	3	0.8098	1
						ISWYDNEFGYSNR	2	0.9884	2
						LEKPAKYDDIK	2	0.9917	3
						LEKPAKYDDIKK	2	0.999	4
						LISWYDNEFGYSNR	2	0.9998	9
						LISWYDNEFGYSNR	3	0.9993	3
						LTGMAFR	2	0.9971	1
						LTGMAFRVPTANVSVVDLTCR	3	0.9994	3
						LVINGNPITIFQER	2	0.9996	2
						LVINGNPITIFQERD	2	0.9461	1
						LVINGNPITIFQERDPSK	2	0.995	1
						SWYDNEFGYSNR	2	0.9964	3
						VIHDNFGIVEGLMTTV	2	0.9446	1
						VIHDNFGIVEGLMTTVHAI	3	0.9769	1
						VIHDNFGIVEGLMTTVHAITATQK	3	0.9996	11
						VIHDNFGIVEGLMTTVHAITATQK	4	0.9981	4
						VPTANVSVVDLTCR	2	0.9998	5
						VVDLMAHMASKE	2	0.9997	2
						VVDLMAHMASKE	3	0.9841	2
						WGDAGAEYVVESTGVFTTMEK	2	0.9997	5
						WGDAGAEYVVESTGVFTTMEK	3	0.9996	3
Q$580-1	P04792	HSPB1_HUMAN	HSPB1	Heat shock protein beta-1	1.0000	67.32	18	25	100
						AQLGGPEAAK	2	0.9996	1
						AQLGGPEAAKSDETAA	2	0.8581	1
						AQLGGPEAAKSDETAAK	2	0.9996	7
						DGVVEITGK	2	0.9995	2
						DGVVEITGKHEER	2	0.9995	2
						DGVVEITGKHEER	3	0.8002	1
						GPSWDPFR	2	0.997	2
						GPSWDPFRDWYPHSR	2	0.9863	2
						GPSWDPFRDWYPHSR	3	0.9979	2
						GPSWDPFRDWYPHSR	4	0.9547	1
						KYTLPPGVDPTQVSSSLSPEGTLTVEAPMPK	3	0.9997	12
						KYTLPPGVDPTQVSSSLSPEGTLTVEAPMPK	4	0.9954	3
						KYTLPPGVDPTQVSSSLSPEGTLTVEAPMPKL	3	0.9916	6
						LATQSNEITIPVTFESR	2	0.9997	6
						QDEHGYISR	2	0.9926	1
						QLSSGVSEIR	2	0.9994	1
						RVPFSLLR	2	0.9929	1
						SLDVNHFAPDELTVK	2	0.9512	2
						TKDGVVEITGK	2	0.9996	4
						TKDGVVEITGK	3	0.9994	3
						TKDGVVEITGKHEER	2	0.9996	19
						TKDGVVEITGKHEER	3	0.9994	7
						VSLDVNHFAPDELTVK	2	0.9997	5
						VSLDVNHFAPDELTVK	3	0.9996	4
						YTLPPGVDPTQVSSSLSPEGTLTVEAPMPK	3	0.9994	5
Q$581-1	P04844	RPN2_HUMAN	RPN2	Dolichyl-diphosphooligosaccharide--protein glycosyltransfera	1.0000	10.94	4	4	6
Q$581-2	P04844-2	RPN2_HUMAN	RPN2	Isoform 2 of Dolichyl-diphosphooligosaccharide--protein glyc	1.0000	11.22	4	4	6
						FSSGYYDFLVEVEGDNR	2	0.979	1
						NFESLSEAFSVASAAAVLSHNR	3	0.9972	1
						SIVEEIEDLVAR	2	0.9997	3
						TSFTPVGDVFELNFMNVK	2	0.9498	1
Q$582-1	P04899	GNAI2_HUMAN	GNAI2	Guanine nucleotide-binding protein G(i) subunit alpha-2	1.0000	14.65	4	5	7
						EIYTHFTCATDTK	2	0.9958	2
						IIHEDGYSEEECR	2	0.9977	1
						LLLLGAGESGK	2	0.958	1
						NVQFVFDAVTDVIIK	2	0.9937	1
						NVQFVFDAVTDVIIK	3	0.9912	2
Q$583-1	P04899-2	GNAI2_HUMAN	GNAI2	Isoform 2 of Guanine nucleotide-binding protein G(i) subunit	1.0000	12.09	3	4	6
Q$583-2	P04899-3	GNAI2_HUMAN	GNAI2	Isoform 3 of Guanine nucleotide-binding protein G(i) subunit	1.0000	12.89	3	4	6
Q$583-3	P04899-5	GNAI2_HUMAN	GNAI2	Isoform 5 of Guanine nucleotide-binding protein G(i) subunit	1.0000	12.09	3	4	6
Q$583-4	P04899-6	GNAI2_HUMAN	GNAI2	Isoform 6 of Guanine nucleotide-binding protein G(i) subunit	1.0000	13.53	3	4	6
						EIYTHFTCATDTK	2	0.9958	2
						IIHEDGYSEEECR	2	0.9977	1
						NVQFVFDAVTDVIIK	2	0.9937	1
						NVQFVFDAVTDVIIK	3	0.9912	2
Q$584-1	P04899-4	GNAI2_HUMAN	GNAI2	Isoform sGi2 of Guanine nucleotide-binding protein G(i) subu	1.0000	10.11	3	3	4
						EIYTHFTCATDTK	2	0.9958	2
						IIHEDGYSEEECR	2	0.9977	1
						LLLLGAGESGK	2	0.958	1
Q$585-1	Q9BTM1	H2AJ_HUMAN	H2AFJ	Histone H2A.J	1.0000	21.71	4	5	8
Q$585-2	Q9BTM1-2	H2AJ_HUMAN	H2AFJ	Isoform 2 of Histone H2A.J	1.0000	18.54	4	5	8
Q$585-3	P16104	H2AX_HUMAN	H2AFX	Histone H2AX	1.0000	19.58	4	5	8
Q$585-4	P04908	H2A1B_HUMAN	HIST1H2AB	Histone H2A type 1-B/E	1.0000	21.54	4	5	8
Q$585-5	Q93077	H2A1C_HUMAN	HIST1H2AC	Histone H2A type 1-C	1.0000	21.54	4	5	8
Q$585-6	P20671	H2A1D_HUMAN	HIST1H2AD	Histone H2A type 1-D	1.0000	21.54	4	5	8
Q$585-7	P0C0S8	H2A1_HUMAN	HIST1H2AG	Histone H2A type 1	1.0000	21.54	4	5	8
Q$585-8	Q96KK5	H2A1H_HUMAN	HIST1H2AH	Histone H2A type 1-H	1.0000	21.88	4	5	8
Q$585-9	Q99878	H2A1J_HUMAN	HIST1H2AJ	Histone H2A type 1-J	1.0000	21.88	4	5	8
Q$585-10	Q8IUE6	H2A2B_HUMAN	HIST2H2AB	Histone H2A type 2-B	1.0000	21.54	4	5	8
Q$585-11	Q7L7L0	H2A3_HUMAN	HIST3H2A	Histone H2A type 3	1.0000	21.54	4	5	8

						AGLQFPVGR	2	0.9832	1
						AVLEYLTAEILELAGNAAR	2	0.9332	1
						AVLEYLTAEILELAGNAAR	3	0.9888	3
						EILELAGNAAR	2	0.8606	1
						VLEYLTAEILELAGNAAR	3	0.9791	2
Q$586-1	P05023-3	AT1A1_HUMAN	ATP1A1	Isoform 3 of Sodium/potassium-transporting ATPase subunit	1.0000	5.14	4	4	5
Q$586-2	P05023-4	AT1A1_HUMAN	ATP1A1	Isoform 4 of Sodium/potassium-transporting ATPase subunit	1.0000	4.99	4	4	5
Q$586-3	P05023	AT1A1_HUMAN	ATP1A1	Sodium/potassium-transporting ATPase subunit alpha-1	1.0000	4.99	4	4	5
						GIVVYTGDR	2	0.9906	1
						GVGIISEGNETVEDIAAR	2	0.9996	2
						LIIVEGCQR	2	0.9945	1
						NIAFFSTNCVEGTAR	2	0.9991	1
Q$587-1	P05060	SCG1_HUMAN	CHGB	Secretogranin-1	0.9635	1.77	1	1	2
						CIIEVLSNALSK	2	0.9635	2
Q$588-1	P05062	ALDOB_HUMAN	ALDOB	Fructose-bisphosphate aldolase B	1.0000	10.99	3	3	6
						IADQCPSSLAIQENANALAR	2	0.9996	3
						LDQGGAPLAGTNK	2	0.9992	2
						LSFSYGR	2	0.9834	1
Q$589-1	P05067	A4_HUMAN	APP	Amyloid beta A4 protein	1.0000	20.39	12	13	24
Q$589-2	P05067-11	A4_HUMAN	APP	Isoform 11 of Amyloid beta A4 protein	1.0000	21.05	12	13	24
Q$589-3	P05067-8	A4_HUMAN	APP	Isoform APP751 of Amyloid beta A4 protein	1.0000	20.91	12	13	24
Q$589-4	P05067-7	A4_HUMAN	APP	Isoform L-APP733 of Amyloid beta A4 protein	1.0000	21.42	12	13	24
Q$589-5	P05067-9	A4_HUMAN	APP	Isoform L-APP752 of Amyloid beta A4 protein	1.0000	20.88	12	13	24
						CAPFFYGGCGGNR	2	0.9997	2
						CLVGEFVSDALLVPDKCK	2	0.9974	1
						CLVGEFVSDALLVPDKCK	3	0.9913	1
						EVCSEQAETGPCR	2	0.9997	3
						ISYGNDALMPSLTETK	2	0.9989	2
						LALENYITALQAVPPRPR	3	0.9754	1
						LNMHMNVQNGK	2	0.8631	1
						QQLVETHMAR	2	0.9832	1
						SQVMTHLR	2	0.9979	2
						STNLHDYGMLLPCGIDKFR	3	0.9475	1
						THPHFVIPYR	3	0.9994	3
						VESLEQEAANER	2	0.9975	2
						WYFDVTEGK	2	0.9997	4
Q$590-1	P05090	APOD_HUMAN	APOD	Apolipoprotein D	1.0000	47.09	18	23	59
						CPNPPVQENFDVNK	2	0.9997	5
						CPNPPVQENFDVNK	3	0.9993	2
						CPNPPVQENFDVNKYLGR	2	0.9995	5
						CPNPPVQENFDVNKYLGR	3	0.9994	3
						DSLKNILTSNNIDVK	2	0.9402	1
						HVDFAWILAR	2	0.8847	1
						IKVLNQELR	2	0.9804	1
						IKVLNQELR	3	0.8852	1
						IPTTFENGR	2	0.9724	1
						KMTVTDQVNCPK	2	0.9997	2
						KMTVTDQVNCPK	3	0.9994	1
						MTVTDQVNCPK	2	0.9996	2
						MTVTDQVNCPKL	2	0.9718	1
						NILTSNNIDVK	2	0.9997	4
						NILTSNNIDVKK	2	0.9997	1
						NPNLPPETVDSLK	2	0.9997	11
						NPNLPPETVDSLKNILTSNNIDVK	3	0.986	2
						NPPVQENFDVNK	2	0.9905	2
						VLNQELR	2	0.9994	2
						WYEIEKIPTTF	2	0.9158	2
						WYEIEKIPTTFE	2	0.963	2
						WYEIEKIPTTFENGR	2	0.9992	6
						WYEIEKIPTTFENGR	3	0.9986	1
Q$591-1	P05109	S10A8_HUMAN	S100A8	Protein S100-A8	1.0000	75.27	19	23	65
						ALNSIIDVYH	2	0.9799	1
						ALNSIIDVYHK	2	0.9996	3
						ALNSIIDVYHK	3	0.9993	3
						ALNSIIDVYHKY	2	0.9946	3
						ALNSIIDVYHKY	3	0.9515	1
						ALNSIIDVYHKYSLIK	2	0.9927	2
						ELDINTDGAVNFQE	2	0.8167	1
						ELDINTDGAVNFQEFLILVIK	3	0.9996	18
						GADVWFK	2	0.9996	2
						GADVWFKELDINTDGAVNFQEFLILVIK	3	0.9952	2
						GNFHAVYR	2	0.9996	3
						GNFHAVYRDDLK	2	0.9989	3
						GNFHAVYRDDLK	3	0.9709	1
						GNFHAVYRDDLKK	2	0.9956	4
						KGADVWFK	2	0.9955	1
						KGNFHAVYR	2	0.9816	2
						KLLETECPQYIR	2	0.9996	4
						KLLETECPQYIR	3	0.9973	2
						LETECPQYIR	2	0.9893	1
						LLETECPQYIR	2	0.9996	3
						LLETECPQYIRK	2	0.9993	2
						NFQEFLILVIK	2	0.9842	2
						QEFLILVIK	2	0.9756	1
Q$592-1	P05154	IPSP_HUMAN	SERPINA5	Plasma serine protease inhibitor	1.0000	28.57	11	12	24
						AAAATGTIFTFR	2	0.9997	3
						AVVEVDESGTR	2	0.9997	2
						DFTFDLYR	2	0.9995	2
						EDQYHYLLDR	2	0.8973	1
						FSIEGSYQLEK	2	0.9965	2
						GFQQLLQELNQPR	2	0.9993	3
						GFQQLLQELNQPR	3	0.9996	2
						GTQEQDFYVTSETVVR	2	0.9997	3
						MQILEGLGLNLQK	2	0.9994	2
						QLELYLPK	2	0.9636	1
						RDFTFDLYR	2	0.9422	1
						TLYLADTFPTNFR	2	0.9997	2
Q$593-1	P05164-2	PERM_HUMAN	MPO	Isoform H14 of Myeloperoxidase	1.0000	19.23	10	10	23
Q$593-2	P05164-3	PERM_HUMAN	MPO	Isoform H7 of Myeloperoxidase	1.0000	16.09	10	10	23
Q$593-3	P05164	PERM_HUMAN	MPO	Myeloperoxidase	1.0000	16.78	10	10	23
						AVSNEIVRFPTDQLTPDQER	3	0.9811	2
						FPTDQLTPDQER	2	0.9992	6
						IANVFTNAFR	2	0.9982	1
						IICDNTGITTVSK	2	0.9822	2
						IICDNTGITTVSKN	2	0.9772	2
						IPCFLAGDTR	2	0.9975	2
						SLMFMQWGQLLDHDLDFTPEPAAR	3	0.9829	1
						VGPLLACIIGTQFR	2	0.9993	3
						VVLEGGIDPILR	2	0.9985	3
						WLPAEYEDGFSLPYGWTPGVK	2	0.9963	1
Q$594-1	P05386	RLA1_HUMAN	RPLP1	60S acidic ribosomal protein P1	1.0000	14.04	1	2	5
						AAGVNVEPFWPGLFAK	2	0.9997	3
						AAGVNVEPFWPGLFAK	3	0.9956	2
Q$595-1	P05387	RLA2_HUMAN	RPLP2	60S acidic ribosomal protein P2	1.0000	48.7	5	5	8
						ILDSVGIEADDDRLNK	2	0.9992	3
						KILDSVGIEADDDRLNK	3	0.9795	1
						NIEDVIAQGIGK	2	0.9993	2
						VISELNGKNIEDVIAQGIGK	3	0.9102	1
						YVASYLLAALGGNSSPSAK	2	0.999	1
Q$596-1	P05388	RLA0_HUMAN	RPLP0	60S acidic ribosomal protein P0	1.0000	35.65	9	10	18
						AGAIAPCEVTVPAQNTGLGPEK	2	0.9996	2
						AGAIAPCEVTVPAQNTGLGPEK	3	0.8427	1
						CFIVGADNVGSK	2	0.992	1

						GHLENNPALEK	2	0.9986	3
						GNVGFVFTK	2	0.9943	1
						GNVGFVFTKEDLTEIR	3	0.9979	1
						GTIEILSDVQLIK	2	0.9994	1
						IIQLLDDYPK	2	0.9994	2
						TSFFQALGITTK	2	0.9997	3
						VLALSVETDYTFPLAEK	2	0.9997	3
Q$597-1	P05534	1A24_HUMAN	HLA-A	HLA class I histocompatibility antigen, A-24 alpha chain	1.0000	20.82	5	5	10
						APWIEQEGPEYWDEETGKVK	3	0.9961	3
						AYLEGTCVDGLR	2	0.9973	1
						CWALGFYPAEITLTWQR	2	0.9996	2
						FIAVGYVDDTQFVR	2	0.9996	2
						WAAVVVPSGEEQR	2	0.9989	2
Q$598-1	P05543	THBG_HUMAN	SERPINA7	Thyroxine-binding globulin	1.0000	4.58	2	2	4
						NALALFVLPK	2	0.9991	2
						SFMLLILER	2	0.9996	2
Q$599-1	P05546	HEP2_HUMAN	SERPIND1	Heparin cofactor 2	1.0000	38.48	26	30	69
						DALENIDPATQMMILNCIYFK	2	0.9907	1
						EYYFAEAQIADFSD	2	0.9738	2
						EYYFAEAQIADFSDPAFISK	2	0.9997	5
						FAFNLYR	2	0.9994	2
						FTVDRPFLF	2	0.9958	8
						FTVDRPFLFLIYEHR	2	0.9992	3
						FTVDRPFLFLIYEHR	3	0.9996	6
						FTVDRPFLFLIYEHR	4	0.9994	4
						GPLDQLEK	2	0.9837	1
						HQGTITVNEEGTQ	2	0.9953	2
						HQGTITVNEEGTQA	2	0.9635	2
						HQGTITVNEEGTQAT	2	0.9865	2
						HQGTITVNEEGTQATT	2	0.9899	3
						HQGTITVNEEGTQATTV	2	0.9937	2
						HQGTITVNEEGTQATTVTTV	2	0.994	2
						IAIDLFK	2	0.9966	1
						LIVVPHK	2	0.8948	1
						LNILNAK	2	0.99	1
						MLFDKNGNMAGISDQR	2	0.9966	3
						MLFDKNGNMAGISDQR	3	0.9988	2
						NFGYTLR	2	0.9974	1
						NGNMAGISDQR	2	0.9985	1
						NYNLVESLK	2	0.9991	2
						QFPILLDFK	2	0.9989	2
						RNFGYTLR	2	0.9964	1
						SVNDLYIQK	2	0.9898	1
						TLEAQLTPR	2	0.9988	2
						TSCLLFMGR	2	0.9996	2
						YEITTIHNLFR	2	0.9996	2
						YEITTIHNLFR	3	0.9979	2
Q$600-1	P05787-2	K2C8_HUMAN	KRT8	Isoform 2 of Keratin, type II cytoskeletal 8	1.0000	41.68	23	27	54
Q$600-2	P05787	K2C8_HUMAN	KRT8	Keratin, type II cytoskeletal 8	1.0000	44.1	23	27	54
						AQYEDIANR	2	0.9966	2
						ASLEAAIADAEQR	2	0.9992	2
						ASLEAAIADAEQR	3	0.9992	2
						ASLEAAIADAEQRGELAIK	3	0.9643	1
						ASLEAAIADAEQRGELAIKDANAK	3	0.9894	2
						DVDEAYMNKVELESR	2	0.9996	2
						DVDEAYMNKVELESR	3	0.999	1
						DVDEAYMNKVELESRLEGLTDEINFLR	3	0.8179	1
						ELQSQISDTSVVLSMDNSR	2	0.9997	2
						EYQELMNVK	2	0.9948	1
						KLLEGEESR	2	0.9986	2
						LALDIEIATYRK	2	0.9972	1
						LEAELGNMQGLVEDFK	2	0.9997	3
						LEGLTDEINFLR	2	0.9997	5
						LEGLTDEINFLR	3	0.9926	2
						LKLEAELGNMQGLVEDFK	2	0.9995	3
						LKLEAELGNMQGLVEDFK	3	0.999	3
						LSELEAALQR	2	0.9996	2
						QLETLGQEK	2	0.9979	1
						QLYEEEIRELQSQISDTSVVLSMDNSR	3	0.9963	3
						RQLETLGQEK	2	0.9916	1
						SLDMDSIIAEVK	2	0.9997	3
						SNMDNMFESYINNLR	2	0.9997	2
						TEMENEFVLIK	2	0.9958	3
						TEMENEFVLIKK	2	0.9995	1
						TKTEISEMNR	2	0.9862	2
						YEELQSLAGK	2	0.9954	1
Q$601-1	P06310	KV206_HUMAN		Ig kappa chain V-II region RPMI 6410	1.0000	9.77	1	1	2
						FSGSGSGTDFTLK	2	0.9993	2
Q$602-1	P06396	GELS_HUMAN	GSN	Gelsolin	1.0000	25.96	16	16	34
Q$602-2	P06396-2	GELS_HUMAN	GSN	Isoform 2 of Gelsolin	1.0000	27.77	16	16	34
Q$602-3	P06396-3	GELS_HUMAN	GSN	Isoform 3 of Gelsolin	1.0000	27.36	16	16	34
Q$602-4	P06396-4	GELS_HUMAN	GSN	Isoform 4 of Gelsolin	1.0000	27.47	16	16	34
						AGALNSNDAFVLK	2	0.9995	3
						AGKEPGLQIWR	3	0.9852	1
						AQPVQVAEGSEPDGFWEALGGK	2	0.9996	1
						DFFTGDAYVILK	2	0.9495	2
						DSQEEEKTEALTSAK	2	0.9916	2
						EVQGFESATFLGYFK	2	0.9997	6
						HVVPNEVVVQR	2	0.9982	1
						IEGSNKVPVDPATYGQFYGGDSYIILYNYR	3	0.9995	4
						NLYGDFFTGDAYVILK	2	0.8426	1
						QTQVSVLPEGGETPLFK	2	0.997	2
						TGAQELLR	2	0.99	1
						TPITVVK	2	0.9952	1
						TPSAAYLWVGTGASEAEK	2	0.9997	2
						TPSAAYLWVGTGASEAEKTGAQELLR	3	0.9975	2
						VEKFDLVPVPTNLYGDFFTGDAYVILK	3	0.9951	3
						YIETDPANR	2	0.9943	2
Q$603-1	P06576	ATPB_HUMAN	ATP5B	ATP synthase subunit beta, mitochondrial	1.0000	32.51	12	15	29
						AHGGYSVFAGVGER	2	0.9988	2
						AHGGYSVFAGVGER	3	0.9452	1
						AIAELGIYPAVDPLDSTSR	2	0.9987	2
						DQEGQDVLLFIDNIFR	2	0.9997	2
						FTQAGSEVSALLGR	2	0.9997	3
						LVLEVAQHLGESTVR	2	0.9997	3
						LVLEVAQHLGESTVR	3	0.9431	1
						SLQDIIAILGMDELSEEDKLTVSR	3	0.8566	1
						TVLIMELINNVAK	2	0.9997	2
						VALTGLTVAEYFR	2	0.9991	3
						VALTGLTVAEYFRDQEGQDVLLFIDNIFR	3	0.9994	3
						VALTGLTVAEYFRDQEGQDVLLFIDNIFR	4	0.813	1
						VALVYGQMNEPPGAR	2	0.965	1
						VLDSGAPIKIPVGPETLGR	3	0.9981	3
						VVDLLAPYAK	2	0.9968	1
Q$604-1	P06681	CO2_HUMAN	C2	Complement C2	1.0000	7.05	4	4	10
						CSSNLVLTGSSER	2	0.9988	3
						EVVTDQFLCSGTQEDESPCKGESGGAVFLER	3	0.9981	3
						GESGGAVFLER	2	0.8935	1
						HAFILQDTK	2	0.9986	3
Q$605-1	P06702	S10A9_HUMAN	S100A9	Protein S100-A9	1.0000	67.54	21	26	66
						DLQNFLK	2	0.9995	1
						DLQNFLKK	2	0.9976	2
						IETIINTFHQYSVK	2	0.8736	1

						INTFHQYSVK	2	0.9945	2
						KDLQNFLK	2	0.9976	2
						KDLQNFLKK	2	0.9883	2
						KLGHPDTLNQGEFK	2	0.9951	3
						KLGHPDTLNQGEFK	3	0.9691	1
						LGHPDTLNQGEFK	2	0.9997	6
						LGHPDTLNQGEFK	3	0.9996	4
						LGHPDTLNQGEFKE	2	0.9948	2
						LGHPDTLNQGEFKELVRK	3	0.9052	1
						LTWASHEK	2	0.9993	3
						NIETIINTF	2	0.9666	1
						NIETIINTFH	2	0.878	1
						NIETIINTFHQ	2	0.8537	1
						NIETIINTFHQY	2	0.9974	6
						NIETIINTFHQY	3	0.9805	1
						NIETIINTFHQYS	2	0.9015	1
						NIETIINTFHQYSV	2	0.9962	3
						NIETIINTFHQYSVK	2	0.9997	10
						NIETIINTFHQYSVK	3	0.9995	3
						QLSFEEFIMLMAR	3	0.9992	2
						VIEHIMEDLDTNADK	3	0.9995	3
						VIEHIMEDLDTNADKQLSFEEFIMLMAR	3	0.9987	3
						VIEHIMEDLDTNADKQLSFEEFIMLMAR	4	0.9439	1
Q$606-1	P06703	S10A6_HUMAN	S100A6	Protein S100-A6	0.9991	31.11	2	2	2
						DQEVNFQEYVTFLGALALIYNEALKG	3	0.9528	1
						NKDQEVNFQEYVTFLGALALIYNEALKG	3	0.9198	1
Q$607-1	P06727	APOA4_HUMAN	APOA4	Apolipoprotein A-IV	1.0000	54.55	22	28	44
						AKIDQNVEELKGR	2	0.9919	2
						AKIDQNVEELKGR	3	0.8572	1
						ALVQQMEQLR	2	0.9996	1
						DKVNSFFSTFK	2	0.9745	1
						ENADSLQASLRPHADELK	3	0.9163	1
						IDQNVEELK	2	0.9986	2
						IDQNVEELKGR	2	0.9939	2
						IDQNVEELKGR	3	0.9184	1
						ISASAEELR	2	0.9997	1
						ISASAEELRQ	2	0.8468	1
						KLVPFATELHER	2	0.9945	1
						KLVPFATELHER	3	0.998	2
						LAPLAEDVR	2	0.9206	1
						LGPHAGDVEGHLSFLEK	4	0.9884	1
						LKEEIGKELEELR	2	0.9924	2
						LKEEIGKELEELR	3	0.9951	2
						LLPHANEVSQK	2	0.999	1
						LNHQLEGLTFQMK	2	0.998	2
						LNHQLEGLTFQMK	3	0.9915	2
						LVPFATELHER	2	0.8977	1
						RVEPYGENFNK	2	0.9964	1
						SELTQQLNALFQDK	2	0.9997	2
						SELTQQLNALFQDK	3	0.8906	1
						SELTQQLNALFQDKLGEVNTYAGDLQK	3	0.9959	3
						SLAELGGHLDQQVEEFR	2	0.9997	2
						SLAPYAQDTQEK	2	0.9982	4
						TQVNTQAEQLR	2	0.9991	2
						TQVNTQAEQLRR	2	0.9405	1
Q$608-1	P06733	ENOA_HUMAN	ENO1	Alpha-enolase	1.0000	55.3	19	21	52
						AAVPSGASTGIYEALELR	2	0.9997	4
						AAVPSGASTGIYEALELR	3	0.9476	1
						AGYTDKVVIGMDVAASEFFR	3	0.9957	2
						DATNVGDEGGFAPNILENK	2	0.9997	3
						EIFDSRGNPTVEVDLFTSK	3	0.8496	1
						FGANAILGVSLAVCK	2	0.9996	2
						FTASAGIQVVGDDLTVTNPK	2	0.9997	3
						FTASAGIQVVGDDLTVTNPK	3	0.9961	2
						FTASAGIQVVGDDLTVTNPKR	3	0.9955	1
						GNPTVEVDLFTSK	2	0.9997	3
						IGAEVYHNLK	2	0.9996	2
						LAMQEFMILPVGAANFR	3	0.9678	1
						SCNCLLLK	2	0.9861	1
						SFIKDYPVVSIEDPFDQDDWGAWQK	3	0.9992	6
						SGETEDTFIADLVVGLCTGQIK	3	0.9996	5
						SGKYDLDFK	2	0.995	1
						SGKYDLDFKSPDDPSR	3	0.9918	2
						VNQIGSVTESLQACK	2	0.9985	3
						VVIGMDVAASEFFR	2	0.9997	4
						YGKDATNVGDEGGFAPNILENK	3	0.9877	1
						YISPDQLADLYK	2	0.9996	4
Q$609-1	P06748-2	NPM_HUMAN	NPM1	Isoform 2 of Nucleophosmin	1.0000	38.49	7	10	23
Q$609-2	P06748	NPM_HUMAN	NPM1	Nucleophosmin	1.0000	34.69	7	10	23
						GGSLPKVEAK	2	0.9322	1
						GPSSVEDIK	2	0.999	1
						MSVQPTVSLGGFEITPPVVLR	2	0.9997	4
						MSVQPTVSLGGFEITPPVVLR	3	0.9973	3
						MTDQEAIQDLWQWR	2	0.9994	3
						MTDQEAIQDLWQWR	3	0.9962	2
						TVSLGAGAKDELHIVEAEAMNYEGSPIK	3	0.9995	3
						TVSLGAGAKDELHIVEAEAMNYEGSPIK	4	0.9784	3
						VDNDENEHQLSLR	2	0.9997	2
						VTLATLK	2	0.9621	1
Q$610-1	P06753-2	TPM3_HUMAN	TPM3	Isoform 2 of Tropomyosin alpha-3 chain	0.9999	10.08	2	2	3
Q$610-2	P06753-5	TPM3_HUMAN	TPM3	Isoform 5 of Tropomyosin alpha-3 chain	0.9999	10.08	2	2	3
Q$610-3	Q5VU61	Q5VU61_HUMAN	TPM3	Tropomyosin alpha-3 chain	0.9999	11.21	2	2	3
Q$610-4	Q5VU59	Q5VU59_HUMAN	TPM3	Uncharacterized protein	0.9999	10.78	2	2	3
						KIQVLQQQADDAEER	3	0.9977	1
						TIDDLEDKLK	2	0.9476	2
Q$611-1	P33778	H2B1B_HUMAN	HIST1H2BB	Histone H2B type 1-B	1.0000	19.05	5	5	9
Q$611-2	P06899	H2B1J_HUMAN	HIST1H2BJ	Histone H2B type 1-J	1.0000	19.05	5	5	9
Q$611-3	P23527	H2B1O_HUMAN	HIST1H2BO	Histone H2B type 1-O	1.0000	19.05	5	5	9
Q$611-4	Q16778	H2B2E_HUMAN	HIST2H2BE	Histone H2B type 2-E	1.0000	19.05	5	5	9
Q$611-5	Q8N257	H2B3B_HUMAN	HIST3H2BB	Histone H2B type 3-B	1.0000	19.05	5	5	9
						AMGIMNSFVNDIFER	2	0.9995	4
						LLLPGELAK	2	0.9986	2
						MNSFVNDIFER	2	0.8689	1
						NSFVNDIFER	2	0.814	1
						SFVNDIFER	2	0.9578	1
Q$612-1	P07195	LDHB_HUMAN	LDHB	L-lactate dehydrogenase B chain	1.0000	21.26	5	5	7
						ITVVGVGQVGMACAISILGK	3	0.9215	1
						IVVVTAGVR	2	0.9019	1
						LIAPVAEEEATVPNNK	2	0.9996	2
						MVVESAYEVIK	2	0.9964	2
						SLADELALVDVLEDK	2	0.9993	1
Q$613-1	P07203	GPX1_HUMAN	GPX1	Glutathione peroxidase 1	1.0000	34.98	5	5	5
						FQTIDIEPDIEALLSQGPSCA	2	0.9796	1
						LAAAAAAAQSVYAFSARPLAGGEPVSLGSLR	3	0.9381	1
						LITWSPVCR	2	0.9882	1
						NDVAWNFEK	2	0.9964	1
						RFQTIDIEPDIEALLSQGPSCA	2	0.9993	1
Q$614-1	P07225	PROS_HUMAN	PROS1	Vitamin K-dependent protein S	1.0000	31.66	24	31	58
						AHSCPSVWK	2	0.9907	1
						AHSCPSVWKK	2	0.9996	3
						AHSCPSVWKK	3	0.9991	1
						ASFTCTCKPGWQGEK	2	0.9996	1
						DCKDVDECSLKPSICGTAVCK	3	0.9495	1

						DVDECSLKPSICGTAVCK	2	0.9997	2
						DVDECSLKPSICGTAVCK	3	0.9937	1
						FSAEFDFR	2	0.9947	1
						HCLVTVEK	2	0.9996	1
						IQALSLCSDQQSH	2	0.975	2
						IQALSLCSDQQSHL	2	0.9691	1
						IQALSLCSDQQSHLEF	2	0.986	2
						IQALSLCSDQQSHLEFR	2	0.9997	3
						IQALSLCSDQQSHLEFR	3	0.999	2
						ITTGGDVINNGLWNMVSVEELEHSISIK	3	0.9861	1
						KVESELIKPINPR	2	0.9997	2
						KVESELIKPINPR	3	0.9985	2
						NGFVMLSNKK	2	0.9966	2
						NNLELSTPLK	2	0.9975	2
						NNLELSTPLKIETISHEDLQR	2	0.9963	1
						NNLELSTPLKIETISHEDLQR	3	0.9994	3
						QSTNAYPDLR	2	0.968	1
						SCEVVSVCLPLNLDTK	2	0.9896	2
						SFQTGLFTAAR	2	0.9981	1
						SLCSDQQSHLEFR	2	0.9987	2
						SQDILLSVENTVIYR	2	0.9997	7
						SQDILLSVENTVIYR	3	0.9996	2
						SQDILLSVENTVIYRI	2	0.8994	1
						VNRNNLELSTPLKIETISHEDLQR	3	0.9976	3
						VNRNNLELSTPLKIETISHEDLQR	4	0.9874	2
						VYFAGFPR	2	0.9985	2
QS615-1	P07237	PDIA1_HUMAN	P4HB	Protein disulfide-isomerase	1.0000	31.3	14	16	22
						DAPEEEDHVLVLR	2	0.9576	2
						EADDIVNWLK	2	0.9972	1
						ENLLDFIK	2	0.979	1
						ILEFFGLK	2	0.9977	1
						ITEFCHR	2	0.9989	1
						LAKVDATEESDLAQQYGVR	3	0.8804	1
						MDSTANEVEAVK	2	0.9976	1
						NFEDVAFDEKK	2	0.9838	1
						TGPAATTLPDGAAAESLVESSEVAVIGFFK	3	0.9405	1
						TGPAATTLPDGAAAESLVESSEVAVIGFFKDVESDSAK	3	0.9198	1
						THILLFLPK	2	0.9987	2
						THILLFLPK	3	0.9996	2
						VDATEESDLAQQYGVR	2	0.9997	2
						YKPESEELTAER	2	0.9994	1
						YKPESEELTAER	3	0.9985	2
						YQLDKDGVVLFK	3	0.9983	2
QS616-1	P07339	CATD_HUMAN	CTSD	Cathepsin D	1.0000	40.05	12	15	31
						AIGAVPLIQGEYMIPCEK	2	0.9996	3
						AYWQVHLDQVEVASGLTLCK	3	0.9734	2
						EGCEAIVDTGTSLMVGPVDEVR	2	0.9997	3
						FDGILGMAYPR	2	0.9997	2
						GPIPEVLK	2	0.9479	1
						ISVNNVLPVFDNLMQQK	2	0.9993	3
						ISVNNVLPVFDNLMQQK	3	0.9994	2
						KAYWQVHLDQVEVASGLTLCK	3	0.9944	1
						LLDIACWIHHK	2	0.9978	2
						LVDQNIFSFYLSR	2	0.9997	3
						LVDQNIFSFYLSR	3	0.999	1
						LVDQNIFSFYLSRDPDAQPGGELMLGGTDSK	3	0.9994	4
						QVFGEATKQPGITFIAAK	2	0.9605	2
						QVFGEATKQPGITFIAAK	3	0.8206	1
						VGFAEAAR	2	0.9997	1
QS617-1	P07384	CAN1_HUMAN	CAPN1	Calpain-1 catalytic subunit	1.0000	3.78	3	3	3
						CLQSGTLFR	2	0.9553	1
						SEQFINLR	2	0.9942	1
						YLGQDYEQLR	2	0.9992	1
QS618-1	P07437	TBB5_HUMAN	TUBB	Tubulin beta chain	1.0000	41.89	15	20	43
						ALTVPELTQQVFDAK	2	0.9995	6
						ALTVPELTQQVFDAK	3	0.996	2
						EIVHIQAGQCGNQIGAK	2	0.9987	2
						EIVHLQAGQCGNQIGAK	2	0.9967	1
						EIVHLQAGQCGNQIGAK	3	0.8644	1
						FWEVISDEHGID	2	0.9626	1
						FWEVISDEHGIDPTGTYHGDSDLQLDR	3	0.9997	7
						FWEVISDEHGIDPTGTYHGDSDLQLDR	4	0.9977	2
						GHYTEGAELVDSVLDVVR	2	0.9967	1
						GHYTEGAELVDSVLDVVRK	3	0.9794	2
						ISVYYNEATGGK	2	0.9997	4
						ISVYYNEATGGKYVPR	2	0.9996	2
						ISVYYNEATGGKYVPR	3	0.9911	1
						LHFFMPGFAPLTSR	2	0.9886	1
						LHFFMPGFAPLTSR	3	0.9695	2
						LTTPTYGDLNHLVSATMSGVTTCLR	3	0.9852	1
						MAVTFIGNSTAIQELFKR	3	0.8833	1
						NSSYFVEWIPNNVK	2	0.8393	1
						TAVCDIPPR	2	0.9915	1
						YLTVAAVFR	2	0.9997	4
QS619-1	P07711	CATL1_HUMAN	CTSL	Cathepsin L1	1.0000	15.32	3	4	5
						AVATVGPISVAIDAGHESFLFYK	2	0.9976	1
						AVATVGPISVAIDAGHESFLFYK	3	0.9845	1
						NHCGIASAASYPTV	2	0.9993	2
						NSWGEEWGMGGYVK	2	0.9991	1
QS620-1	P07737	PROF1_HUMAN	PFN1	Profilin-1	1.0000	53.57	8	8	19
						CYEMASHLR	2	0.9873	1
						DSLLQDGEFSMDLR	2	0.9995	5
						DSPSVWAAVPGK	2	0.9876	2
						SLLQDGEFSMDLR	2	0.9904	3
						STGGAPTFNVTVTK	2	0.9989	1
						TFVNITPAEVGVLVGK	2	0.9996	5
						TKSTGGAPTFNVTVTK	2	0.9936	1
						TLVLLMGK	2	0.9462	1
QS621-1	P07814	SYEP_HUMAN	EPRS	Bifunctional glutamate/proline--tRNA ligase	1.0000	5.75	6	6	10
						DQDLEPGAPSMGAK	2	0.9829	1
						FAGGDYTTTIEAFISASGR	2	0.9175	1
						GFFICDQPYEPVSPYSCK	2	0.9996	3
						INEAVECLLSLK	2	0.9984	1
						KGDIIQLQR	2	0.9975	2
						LQAILEDIQVTLFTR	2	0.9997	2
QS622-1	P07858	CATB_HUMAN	CTSB	Cathepsin B	1.0000	50.15	13	18	40
						DQGSCGSCWAFGAVEAISDR	2	0.9998	4
						DQGSCGSCWAFGAVEAISDR	3	0.9997	2
						EIRDQGSCGSCWAFGAVEAISDR	3	0.9825	1
						EQWPQCPTIK	2	0.9997	3
						EQWPQCPTIK	3	0.995	1
						GLVSGGLYESHVGCRPY	2	0.9969	2
						GQDHCGIESEVVAGIPR	2	0.9998	2
						GQDHCGIESEVVAGIPR	3	0.9996	4
						HYGYNSYSVSNSEK	2	0.9995	1
						HYGYNSYSVSNSEKDIMAEIYK	3	0.9618	3
						ICEPGYSPTYKQDK	2	0.9968	2
						ILGWGVENGTPYWLVANSWNTDWGDNGFFK	3	0.9932	1
						KGLVSGGLYESHVGCRPY	3	0.8831	1
						NGPVEGAFSVYSDFLLYK	2	0.9997	8
						NGPVEGAFSVYSDFLLYK	3	0.9995	2
						SGVYQHVTGEMMGGHAIR	2	0.9997	1
						SGVYQHVTGEMMGGHAIR	3	0.9994	1

						WLVANSWNTDWGDNGFFK	2	0.9898	1
Q$623-1	P07900	HS90A_HUMAN	HSP90AA1	Heat shock protein HSP 90-alpha	1.0000	47.68	36	44	102
Q$623-2	P07900-2	HS90A_HUMAN	HSP90AA1	Isoform 2 of Heat shock protein HSP 90-alpha	1.0000	40.87	36	44	102
						ADLINNLGTIAK	2	0.9997	4
						CIVTSTYGWTANMER	2	0.9975	3
						DLINNLGTIAK	2	0.9414	1
						DLVILLYETALLSSGFSLEDPQTHANR	3	0.9996	3
						DNSTMGYMAAK	2	0.9952	1
						EGLELPEDEEEK	2	0.9954	1
						EGLELPEDEEEKK	2	0.9869	2
						EGLELPEDEEEKKK	2	0.9921	2
						ELHINLIPNKQDR	2	0.9986	2
						ELISNSSDALDK	2	0.9989	3
						ELISNSSDALDKIR	2	0.9995	2
						GVGFYSAYLVAEK	2	0.9454	1
						GVVDSEDLPLNISR	2	0.9995	5
						HFSVEGQLEFR	2	0.9997	4
						HFSVEGQLEFR	3	0.9992	2
						HGLEVIYMIEPIDEYCVQQL	2	0.9598	1
						HGLEVIYMIEPIDEYCVQQLK	3	0.9996	3
						HGLEVIYMIEPIDEYCVQQLKEFEGK	3	0.9981	4
						HIYYITGETK	2	0.9993	3
						HIYYITGETK	3	0.9411	1
						HIYYITGETKDQVANSAFVER	3	0.9994	4
						HLEINPDHSIIETLR	2	0.9996	2
						HLEINPDHSIIETLR	3	0.9995	4
						HNDDEQYAWESSAGGSF	2	0.9972	2
						HNDDEQYAWESSAGGSFTVR	2	0.9997	3
						HNDDEQYAWESSAGGSFTVR	3	0.996	3
						IEPIDEYCVQQLK	2	0.9809	2
						KHGLEVIYMIEPIDEYCVQQLK	3	0.9995	2
						KHGLEVIYMIEPIDEYCVQQLK	4	0.9973	2
						KTKFENLCK	2	0.9838	1
						LVTSPCCIVTSTYGWTANMER	2	0.9997	2
						LVTSPCCIVTSTYGWTANMER	3	0.9984	5
						NPDDITNEEYGEFYK	2	0.9997	4
						SLTNDWEDHLAVK	2	0.9997	3
						SLTNDWEDHLAVK	3	0.9332	1
						TLTLVDTGIGMTK	2	0.9995	4
						TLVSVTK	2	0.9267	1
						VFIMDNCEELIPEYLNFIR	3	0.9689	1
						VILHLKEDQTEYLEER	3	0.9919	3
						VILHLKEDQTEYLEERR	3	0.9877	1
						VILHLKEDQTEYLEERR	4	0.882	1
						YIDQEELNK	2	0.9678	1
						YMIEPIDEYCVQQLK	2	0.9588	1
						YYTSASGDEMVSLK	2	0.9996	1
Q$624-1	P07911-3	UROM_HUMAN	UMOD	Isoform 3 of Uromodulin	1.0000	15.38	10	10	29
						CPHTRDSTIQVVENGESSQGR	3	0.8523	1
						DSTIQVVENGESSQGR	2	0.9997	4
						FSVQMFR	2	0.9994	2
						INFACSYPLDMK	2	0.9968	1
						SLGFDKVFMYLSDSR	3	0.9956	2
						STEYGEGYACDTDLR	2	0.9997	10
						TLDEYWR	2	0.9993	1
						VFMYLSDSR	2	0.9995	4
						VGGTGMFTVR	2	0.9994	2
						YFIIQDR	2	0.9988	2
Q$625-1	P07911-4	UROM_HUMAN	UMOD	Isoform 4 of Uromodulin	1.0000	28.64	20	20	50
Q$625-2	P07911-5	UROM_HUMAN	UMOD	Isoform 5 of Uromodulin	1.0000	26.3	20	20	50
Q$625-3	P07911	UROM_HUMAN	UMOD	Uromodulin	1.0000	27.66	20	20	50
Q$625-4	X6RBG4	X6RBG4_HUMAN	UMOD	Uromodulin	1.0000	25.69	20	20	50
						ALVCADPCQAHR	2	0.8378	1
						CPHTRDSTIQVVENGESSQGR	3	0.8523	1
						DGPCGTVLTR	2	0.9929	1
						DSTIQVVENGESSQGR	2	0.9997	4
						DWVSVVTPAR	2	0.9923	4
						FAGNYDLVYLH	2	0.9642	2
						FAGNYDLVYLHCE	2	0.994	3
						FSVQMFR	2	0.9994	2
						INFACSYPLDMK	2	0.9968	1
						LADEIIIR	2	0.9512	1
						MAETCVPVLR	2	0.9997	1
						SLGFDKVFMYLSDSR	3	0.9956	2
						STEYGEGYACDTDLR	2	0.9997	10
						STEYGEGYACDTDLRGWYR	3	0.9955	3
						TALQPMVSALNIR	2	0.9959	3
						TLDEYWR	2	0.9993	1
						TLYLADEIIIR	2	0.9752	2
						VFMYLSDSR	2	0.9995	4
						VGGTGMFTVR	2	0.9994	2
						YFIIQDR	2	0.9988	2
Q$626-1	P07996	TSP1_HUMAN	THBS1	Thrombospondin-1	1.0000	12.82	11	12	22
						CTSYPDGSWK	2	0.9962	2
						DCVGDVTENQICNK	2	0.9996	1
						DLQAICGISCDELSSMVLELR	2	0.9989	1
						FTGSQPFGQGVEHATANK	2	0.9994	2
						FVFGTTPEDILR	2	0.9988	3
						GGVNDNFQGVLQNVR	2	0.9994	2
						IEDANLIPPVPDDKFQDLVDAVR	3	0.9351	2
						KVTEENKELANELR	2	0.9994	2
						KVTEENKELANELR	3	0.9954	1
						LCNNPTPQFGGK	2	0.9995	1
						LCNNPTPQFGGKDCVGDVTENQICNK	3	0.9985	4
						TIVTTLQDSIR	2	0.9994	1
Q$627-1	P08134	RHOC_HUMAN	RHOC	Rho-related GTP-binding protein RhoC	1.0000	29.02	6	6	10
Q$627-2	Q5JR08	Q5JR08_HUMAN	RHOC	Rho-related GTP-binding protein RhoC (Fragment)	1.0000	29.79	6	6	10
						EVFEMATR	2	0.8642	1
						HFCPNVPIILVGNKK	3	0.9995	2
						ISAFGYLECSAK	2	0.9886	1
						KLVIVGDGACGK	2	0.9964	1
						LVIVGDGACGK	2	0.984	2
						TCLLIVFSK	2	0.9996	3
Q$628-1	P08185	CBG_HUMAN	SERPINA6	Corticosteroid-binding globulin	1.0000	8.4	3	3	4
						EENFYVDETTVVK	2	0.9657	2
						GTWTQPFDLASTR	2	0.9868	1
						HLVALSPK	2	0.9127	1
Q$629-1	P08237	PFKAM_HUMAN	PFKM	ATP-dependent 6-phosphofructokinase, muscle type	1.0000	2.31	2	2	3
Q$629-2	P08237-3	PFKAM_HUMAN	PFKM	Isoform 3 of ATP-dependent 6-phosphofructokinase, muscle	1.0000	2.12	2	2	3
						TFVLEVMGR	2	0.9965	2
						VTVLGHVQR	2	0.9913	1
Q$630-1	P08238	HS90B_HUMAN	HSP90AB1	Heat shock protein HSP 90-beta	1.0000	48.76	35	43	100
						ADLINNLGTIAK	2	0.9997	4
						AKFENLCK	2	0.996	1
						ALLFIPR	2	0.9986	2
						CIVTSTYGWTANMER	2	0.9975	3
						DLVVLLFETALLSSGFSLEDPQTH	3	0.9771	1
						DLVVLLFETALLSSGFSLEDPQTHSNR	4	0.9975	2
						EDQTEYLEER	2	0.9547	2
						EGLELPEDEEEK	2	0.9954	1
						EGLELPEDEEEKK	2	0.9869	2
						EGLELPEDEEEKKK	2	0.9921	2
						EQVANSAFVER	2	0.9997	2

						FYEAFSK	2	0.9722	1
						GFEVVYMTEPIDEYCVQQLK	2	0.9997	5
						GFEVVYMTEPIDEYCVQQLK	3	0.9909	2
						GVGFYSAYLVAEK	2	0.9454	1
						GVVDSEDIPLNLSR	2	0.9922	7
						GVVDSEDLPLNISR	2	0.9995	5
						HFSVEGQLEFR	2	0.9997	4
						HFSVEGQLEFR	3	0.9992	2
						HLEINPDHPIVETLR	2	0.9996	2
						HLEINPDHPIVETLR	3	0.9996	3
						HNDDEQYAWESSAGGSF	2	0.9972	2
						HNDDEQYAWESSAGGSFTVR	2	0.9997	3
						HNDDEQYAWESSAGGSFTVR	3	0.996	3
						IDIIPNPQER	2	0.9982	1
						KHLEINPDHPIVETLR	4	0.9722	1
						LGIHEDSTNR	2	0.9997	1
						LVSSPCCIVTSTYGWTANMER	2	0.9997	2
						LVSSPCCIVTSTYGWTANMER	3	0.9978	3
						NPDDITQEEYGEFYK	2	0.9997	4
						PEEVHHGEEEVETFAFQAEIAQLMSLIINTFYSNK	4	0.9936	2
						RGFEVVYMTEPIDEYCVQQLK	3	0.8946	1
						RLSELLR	2	0.9842	1
						SIYYITGESK	2	0.9997	1
						SIYYITGESKEQVANSAFVER	3	0.9937	2
						SLTNDWEDHLAVK	2	0.9997	3
						SLTNDWEDHLAVK	3	0.9332	1
						TLTLVDTGIGMTK	2	0.9995	4
						VILHLKEDQTEYLEER	3	0.9919	3
						VILHLKEDQTEYLEERR	3	0.9877	1
						VILHLKEDQTEYLEERR	4	0.882	1
						YHTSQSGDEMTSLSEYVSR	2	0.9997	3
						YHTSQSGDEMTSLSEYVSR	3	0.9995	3
Q$631-1	P08246	ELNE_HUMAN	ELANE	Neutrophil elastase	1.0000	44.94	11	13	29
						AQFVNWIDSIIQR	2	0.9693	2
						GGCASGLYPDAFAPVAQFVNWIDSIIQR	2	0.9991	1
						GGCASGLYPDAFAPVAQFVNWIDSIIQR	3	0.9992	3
						GGHFCGATLIAPNFVMSAAHCVANVNVR	3	0.9643	1
						GLIHGIASFVR	3	0.8604	1
						LGNGVQCLAMGWGLLGR	2	0.999	5
						NWIDSIIQR	2	0.891	1
						QAGVCFGDSGSPLVCNGLIHGIASFVR	3	0.9993	9
						RLGNGVQCLAMGWGLLGR	3	0.8277	1
						RSNVCTLVR	2	0.9631	1
						SNVCTLVR	2	0.9616	1
						VVLGAHNLSR	2	0.999	2
						VVLGAHNLSR	3	0.9691	1
Q$632-1	P08263	GSTA1_HUMAN	GSTA1	Glutathione S-transferase A1	1.0000	31.08	5	5	14
Q$632-2	P09210	GSTA2_HUMAN	GSTA2	Glutathione S-transferase A2	1.0000	31.08	5	5	14
						ADIHLVELLYYVEELDSSLISSFPLLK	3	0.9991	3
						AILNYIASK	2	0.9966	1
						SAEDLDKLR	2	0.9879	1
						SHGQDYLVGNK	2	0.9955	1
						WLLAAAGVEFEEK	2	0.9997	8
Q$633-1	P08294	SODE_HUMAN	SOD3	Extracellular superoxide dismutase [Cu-Zn]	1.0000	61.67	16	21	43
						AGLAASLAGPHSIVGR	2	0.9997	3
						AGLAASLAGPHSIVGR	3	0.9994	2
						AIHVHQFGDLSQGCESTGPHYNPLAVPHPQHPGDFGNFAVR	4	0.9785	1
						AVVVHAGEDDLGR	2	0.9997	4
						AVVVHAGEDDLGR	3	0.9994	1
						AVVVHAGEDDLGRGGNQASVENGNAGR	3	0.964	1
						AVVVHAGEDDLGRGGNQASVENGNAGRR	3	0.8852	1
						CVVGVCGPGLWER	2	0.9856	2
						DDDGALHAACQVQPSATLDAAQPR	3	0.9994	4
						LACCVVGVCGPGLWER	2	0.9997	3
						LACCVVGVCGPGLWER	3	0.9994	2
						LACCVVGVCGPGLWERQ	2	0.823	1
						RDDDGALHAACQVQPSATLDAAQPR	3	0.9997	5
						RDDDGALHAACQVQPSATLDAAQPR	4	0.8466	1
						RLACCVVGVCGPGLWER	3	0.9991	2
						VTEIWQEVMQR	2	0.9997	3
						VTEIWQEVMQR	3	0.9984	2
						VTGVVLFR	2	0.9997	2
						VVHAGEDDLGR	2	0.9224	1
						VVVHAGEDDLGR	2	0.9469	1
						YRAGLAASLAGPHSIVGR	3	0.9994	1
Q$634-1	P08311	CATG_HUMAN	CTSG	Cathepsin G	1.0000	34.9	11	11	21
						AQEGLRPGTLCTV	2	0.9649	4
						AQEGLRPGTLCTVAGWGR	3	0.997	1
						AYLQIQSPAGQSR	2	0.9941	3
						CGGFLVR	2	0.9903	1
						IFGSYDPR	2	0.9993	1
						LQIQSPAGQSR	2	0.8015	1
						NNVAHGIVSYGK	2	0.9529	2
						NVAHGIVSYGK	2	0.954	1
						NVNPVALPR	2	0.9346	1
						TIQNDIMLLQLSR	2	0.9981	3
						VSSFLPWIR	2	0.9992	3
Q$635-1	P08519	APOA_HUMAN	LPA	Apolipoprotein(a)	1.0000	1.65	6	6	11
						CQSWSSMTPHR	2	0.9982	2
						GTYSTTVTGR	2	0.9997	2
						NPDAVAAPYCYTR	2	0.9995	3
						TPENYPNAGLTENYCR	2	0.9995	1
						TPENYPNAGLTR	2	0.9589	1
						VQDCYHGDGQSYR	2	0.9974	2
Q$636-1	P08582	TRFM_HUMAN	MFI2	Melanotransferrin	1.0000	13.69	9	9	15
						ADTDGGLIFR	2	0.9533	1
						CLAEGAGDVAFVK	2	0.9978	2
						CLVENAGDVAFVR	2	0.998	2
						LSVMGCDVLK	2	0.9882	1
						SSHVTIDTLK	2	0.9975	2
						TVGWNVPVGYLVESGR	2	0.9994	3
						VPAHAVVVR	2	0.9955	1
						WCVLSTPEIQK	2	0.9348	1
						YYDYSGAFR	2	0.9982	2
Q$637-1	P08582-2	TRFM_HUMAN	MFI2	Isoform 2 of Melanotransferrin	1.0000	15.89	4	4	9
						CLAEGAGDVAFVK	2	0.9978	2
						SSHVTIDTLK	2	0.9975	2
						TVGWNVPVGYLVESGR	2	0.9994	3
						YYDYSGAFR	2	0.9982	2
Q$638-1	P08603	CFAH_HUMAN	CFH	Complement factor H	0.9999	1.95	2	2	2
						CFEGFGIDGPAIAK	2	0.9973	1
						LSYTCEGGFR	2	0.9621	1
Q$639-1	P08697	A2AP_HUMAN	SERPINF2	Alpha-2-antiplasmin	1.0000	36.05	16	16	33
Q$639-2	P08697-2	A2AP_HUMAN	SERPINF2	Isoform 2 of Alpha-2-antiplasmin	1.0000	41.45	16	16	33
						DTTGLPLFVGSVR	2	0.8528	1
						FIFEDTTGLPLFVGSVR	2	0.9724	1
						GISEQSLVVSGVQHQSTLELSEVGVEAAAATSIAMSR	3	0.9996	4
						HQMDLVATLSQLGLQEL	2	0.9831	2
						IFEDTTGLPLFVGSVR	2	0.9863	3
						IQEFLSGLPEDTVLLLLNAIHFQGFWR	3	0.9848	2
						LCQDLGPGAFR	2	0.9994	3
						LCQDLGPGAFRL	2	0.9681	1
						LQQVLHAGSGPCLPHLLSR	3	0.9985	4

						MSLSSFSVNRPF	2	0.8588	1
						MSLSSFSVNRPFLF	2	0.8699	1
						MSLSSFSVNRPFLFF	2	0.9439	1
						MSLSSFSVNRPFLFFIFEDTTGLPLFVGSVR	3	0.9873	1
						NKFDPSLTQR	2	0.8812	1
						SQLGLQELFQAPDLR	2	0.9917	2
						WFLLEQPEIQVAHFPFK	3	0.9984	5
Q$640-1	P08727	K1C19_HUMAN	KRT19	Keratin, type I cytoskeletal 19	1.0000	61.25	23	28	56
						AALEDTLAETEAR	2	0.9997	4
						ALEAANGELEVK	2	0.9991	2
						DAEAWFTSR	2	0.9997	2
						DKILGATIENSR	2	0.9994	2
						DYSHYYTTIQDLR	2	0.9997	3
						DYSHYYTTIQDLR	3	0.9987	1
						EVAGHTEQLQMSR	2	0.9996	2
						FGAQLAHIQALISGIEAQLGDVR	3	0.9997	3
						FGPGVAFR	2	0.9986	1
						GQVGGQVSVEVDSAPGTDLAK	2	0.9997	3
						ILGATIENSR	2	0.999	2
						IVLQIDNAR	2	0.9996	2
						KDAEAWFTSR	2	0.9817	1
						KNHEEEISTLR	2	0.9708	1
						NHEEEISTLR	2	0.9996	1
						QSSATSSFGGLGGGSVR	2	0.9997	2
						SLLEGQEDHYNNLSASK	2	0.9481	1
						SQYEVMAEQNR	2	0.9997	1
						SQYEVMAEQNRK	2	0.9995	2
						SRLEQEIATYR	2	0.9986	2
						TDLEMQIEGLKEELAYLK	2	0.9988	3
						TDLEMQIEGLKEELAYLK	3	0.9986	3
						TDLEMQIEGLKEELAYLKK	3	0.9991	2
						TDLEMQIEGLKEELAYLKK	4	0.9867	1
						TKFETEQALR	2	0.9992	2
						TKFETEQALR	3	0.9955	1
						TLQGLEIELQSQLSMK	2	0.9997	2
						TLQGLEIELQSQLSMK	3	0.9996	4
Q$641-1	P08729	K2C7_HUMAN	KRT7	Keratin, type II cytoskeletal 7	1.0000	56.08	30	37	79
						AEAEAWYQTK	2	0.9988	3
						AEAEAWYQTKFETLQAQAGK	3	0.9807	2
						AKLEAAIAEAEER	2	0.9987	2
						AKLEAAIAEAEER	3	0.9967	1
						AKLEAAIAEAEERGELALK	3	0.9923	2
						AKQEELEAALQR	2	0.9981	2
						AKQEELEAALQR	3	0.9927	1
						AQYEEMAK	2	0.9037	1
						DVDAAYMSKVELEAK	2	0.9994	2
						ELQSQISDTSVVLSMDNSR	2	0.9997	2
						EVTINQSLLAPLR	2	0.998	2
						EVTINQSLLAPLRLDADPSLQR	3	0.9241	1
						EYQELMSVK	2	0.9703	1
						FETLQAQAGK	2	0.9937	1
						GQLEALQVDGGR	2	0.9997	3
						GQLEALQVDGGRLEAELR	2	0.9985	3
						GQLEALQVDGGRLEAELR	3	0.9995	4
						KLLEGEESR	2	0.9986	2
						LALDIEIATYRK	2	0.9972	1
						LEAAIAEAEER	2	0.9828	2
						LPDIFEAQIAGLR	2	0.9998	5
						LPDIFEAQIAGLR	3	0.9996	4
						LQAEIDNIK	2	0.9978	1
						LQAEIDNIKNQR	2	0.9989	2
						NTRNEISEMNR	2	0.9857	1
						SLDLDGIIAEVK	2	0.9997	5
						SMQDVVEDFK	2	0.9986	1
						SSRLPDIFEAQIAGLR	2	0.999	2
						SSRLPDIFEAQIAGLR	3	0.9995	3
						TAAENEFVVLK	2	0.9638	2
						TAAENEFVVLKK	2	0.9973	3
						TAAENEFVVLKK	3	0.9724	1
						TLNETELTELQSQISDTSVVLSMDNSR	3	0.999	2
						VDALNDEINFLR	2	0.9997	5
						VDALNDEINFLR	3	0.9905	1
						VELEAKVDALNDEINFLR	3	0.9716	2
						WTLLQEQK	2	0.9882	1
Q$642-1	P63096	GNAI1_HUMAN	GNAI1	Guanine nucleotide-binding protein G(i) subunit alpha-1	1.0000	11.02	3	4	6
Q$642-2	P08754	GNAI3_HUMAN	GNAI3	Guanine nucleotide-binding protein G(k) subunit alpha	1.0000	11.02	3	4	6
						EIYTHFTCATDTK	2	0.9958	2
						LLLLGAGESGK	2	0.958	1
						NVQFVFDAVTDVIIK	2	0.9937	1
						NVQFVFDAVTDVIIK	3	0.9912	2
Q$643-1	P08779	K1C16_HUMAN	KRT16	Keratin, type I cytoskeletal 16	1.0000	59.62	25	31	59
						APSTYGGGLSVSSR	2	0.9995	3
						ASLENSLEETK	2	0.9996	1
						ASLENSLEETKGR	2	0.9933	1
						CEMEQQSQEYQILLDVK	2	0.9996	2
						CEMEQQSQEYQILLDVK	3	0.997	3
						DAETWFLSK	2	0.9982	2
						DAETWFLSKTEELNKEVASNSELVQSSR	3	0.9953	2
						EVASNSELVQSSR	2	0.9996	1
						EVFTSSSSSSSR	2	0.9983	2
						GQTGGDVNVEMDAAPGVDLSR	2	0.9997	2
						GSCGIGGGIGGGSSR	2	0.9993	1
						IIAATIENAQPILQIDNAR	2	0.9997	2
						IIAATIENAQPILQIDNAR	3	0.9996	3
						ILNEMRDQYEQMAEK	2	0.9972	2
						ISSVLAGGSCR	2	0.9994	1
						LLEGEDAHLSSQQASGQS	2	0.9948	2
						LLEGEDAHLSSQQASGQSYSSR	3	0.9996	3
						NHEEEMLALR	2	0.9571	2
						NKIIAATIENAQPILQIDNAR	3	0.9982	2
						QRPSEIKDYSPYFK	3	0.814	1
						RLLEGEDAHLSSQQASGQSYSSR	2	0.8349	1
						RLLEGEDAHLSSQQASGQSYSSR	3	0.9992	2
						TDLEMQIEGLKEELAYLR	2	0.9982	2
						TDLEMQIEGLKEELAYLR	3	0.9994	3
						TDLEMQIEGLKEELAYLRK	3	0.9976	2
						TEELNKEVASNSELVQSSR	3	0.9973	1
						TKYEHELALR	2	0.9809	2
						TKYEHELALR	3	0.8301	1
						VLQGLEIELQSQLSMK	2	0.9801	1
						VLQGLEIELQSQLSMK	3	0.9935	3
						YCMQLSQIQGLIGSVEEQLAQLR	3	0.9994	3
Q$644-1	P09211	GSTP1_HUMAN	GSTP1	Glutathione S-transferase P	1.0000	46.19	8	8	19
						ALPGQLKPFETLLSQNQGGK	3	0.9972	2
						ASCLYGQLPK	2	0.9996	3
						FQDGDLTLYQSNTILR	2	0.9997	6
						MLLADQGQSWKEEVVTVETWQEGSLK	3	0.9921	2
						PPYTVVYFPVR	2	0.9984	3
						SCLYGQLPK	2	0.983	1
						YISLIYTNYEAGK	2	0.9416	1
						YISLIYTNYEAGKD	2	0.8939	1
Q$645-1	P09429	HMGB1_HUMAN	HMGB1	High mobility group protein B1	1.0000	14.88	5	6	13
Q$645-2	Q5T7C4	Q5T7C4_HUMAN	HMGB1	High mobility group protein B1	1.0000	20.25	5	6	13

Q$645-3	Q5T7C6	Q5T7C6_HUMAN	HMGB1	High mobility group protein B1 (Fragment)	1.0000	19.75	5	6	13
						GEHPGLSIGDVAK	2	0.9879	2
						IKGEHPGLSIGDVAK	2	0.9992	3
						IKGEHPGLSIGDVAK	3	0.9984	2
						IKGEHPGLSIGDVAKK	3	0.9578	1
						RPPSAFFLF	2	0.8304	1
						RPPSAFFLFCSEYRPK	3	0.9987	4
Q$646-1	P09525	ANXA4_HUMAN	ANXA4	Annexin A4	1.0000	13.48	3	4	11
						GAGTDEGCLIEILASR	2	0.9997	2
						GLGTDEDAIISVLAYR	2	0.9997	5
						GLGTDEDAIISVLAYR	3	0.9986	3
						ISQTYQQQYGR	2	0.9993	1
Q$647-1	P09758	TACD2_HUMAN	TACSTD2	Tumor-associated calcium signal transducer 2	1.0000	13.62	3	3	6
						AAGDVDIGDAAYYFER	2	0.9997	2
						ALGSGMAVDCSTLTSK	2	0.9995	3
						MTVCSPDGPGGR	2	0.9908	1
Q$648-1	P09871	C1S_HUMAN	C1S	Complement C1s subcomponent	1.0000	16.28	8	8	17
						CVPVCGVPR	2	0.8785	1
						DVVQITCLDGFEVVEGR	2	0.9995	4
						GFQVVVTLR	2	0.9993	3
						LPVAPLR	2	0.972	1
						QFGPYCGHGFPGPLNIETK	3	0.9533	1
						SNALDIIFQTDLTGQK	2	0.9978	2
						SSNNPHSPIVEEFQVPYNK	3	0.8922	2
						SWDIEVPEGYGIHLYF	2	0.9818	3
Q$649-1	P09960-2	LKHA4_HUMAN	LTA4H	Isoform 2 of Leukotriene A-4 hydrolase	0.9995	3.57	2	2	3
Q$649-2	P09960	LKHA4_HUMAN	LTA4H	Leukotriene A-4 hydrolase	0.9995	3.11	2	2	3
						CSVDFTR	2	0.9572	1
						LIALVVGALESR	2	0.9807	2
Q$650-1	P0C0L4	CO4A_HUMAN	C4A	Complement C4-A	1.0000	32.45	56	62	150
						AACAQLNDFLQEYGTQGCQV	2	0.9997	4
						ADGSYAAWLSR	2	0.992	2
						AEFQDALEK	2	0.972	1
						AEMADQASAWLTR	2	0.9985	3
						ALDALSAYWIASHTTEER	2	0.9905	2
						ALSAYWIASHTTEER	2	0.9519	2
						ASAGLLGAHAAAITAYALTLTK	2	0.9997	2
						ASAGLLGAHAAAITAYALTLTK	3	0.9995	3
						AVGSGATFSHYYY	2	0.9477	1
						AYWIASHTTEER	2	0.9874	3
						CSVFYGAPSK	2	0.9985	2
						DALSAYWIASHTTEER	2	0.9929	4
						DHAVDLIQK	2	0.9992	1
						DSSTWLTAFVLK	2	0.9994	5
						ECVGFEAVQEVPVGLVQPASATLYDYYNPER	3	0.9734	1
						ECVGFEAVQEVPVGLVQPASATLYDYYNPERR	3	0.9997	3
						EFHLHLR	2	0.899	1
						EGAIHREELVYELNPLDHR	3	0.9984	4
						FACYYPR	2	0.9663	1
						FGLLDEDGKK	2	0.9982	2
						GLCVATPVQLR	2	0.9591	1
						GLEEELQF	2	0.8724	1
						GLEEELQFSLGSK	2	0.9997	5
						GLQDEDGYR	2	0.9763	2
						GSFEFPVGDAVSK	2	0.9995	7
						HLVPGAPFLLQ	2	0.8824	1
						HLVPGAPFLLQALVR	2	0.9751	1
						HLVPGAPFLLQALVR	3	0.9997	5
						KADGSYAAWLSR	2	0.9994	4
						KADGSYAAWLSR	3	0.9991	4
						KYVLPNFEVK	2	0.8029	1
						KYVLPNFEVK	3	0.9942	1
						LGQYASPTAK	2	0.9992	1
						LHLETDSLALVALGALDTALYAAGSK	3	0.9994	3
						LLATLCSAEVCQCAEGK	2	0.9992	3
						LLLHEGK	2	0.8616	1
						LNMGITDLQGLR	2	0.9901	1
						PVAFSVVPTAAAAVSLK	2	0.9861	2
						QGSFQGGFR	2	0.9945	1
						SAYWIASHTTEER	2	0.9807	2
						SFFPENWLWR	2	0.9994	5
						SHALQLNNR	2	0.9995	4
						STQDTVIALDALSAYWIASH	2	0.8476	1
						STQDTVIALDALSAYWIASHTTEER	3	0.9995	5
						TAYALTLTK	2	0.952	1
						TTNIQGINLLFSSR	2	0.9995	3
						TYNVLDMK	2	0.8476	1
						VDFTLSSER	2	0.9983	1
						VEYGFQVK	2	0.998	1
						VGDTLNLNLR	2	0.9988	3
						VGLSGMAIADVTLLSGFHALR	3	0.9996	4
						VHYTVCIWR	2	0.9994	2
						VHYTVCIWR	3	0.9969	2
						VLSLAQEQVGGSPEK	2	0.9997	2
						VLSLAQEQVGGSPEKL	2	0.9811	2
						VTASDPLDTLGSEGALSPGGVASLLR	2	0.9997	4
						VTASDPLDTLGSEGALSPGGVASLLR	3	0.9989	4
						WIASHTTEER	2	0.9957	1
						YIYGKPVQGVAY	2	0.8997	1
						YIYGKPVQGVAYVR	3	0.9987	2
						YVSHFETEGPHVLLYFDSVPTSR	3	0.9996	5
						YWIASHTTEER	2	0.949	2
Q$651-1	P0C0L5	CO4B_HUMAN	C4B	Complement C4-B	1.0000	31.48	55	60	145
						AACAQLNDFLQEYGTQGCQV	2	0.9997	4
						ADGSYAAWLSR	2	0.992	2
						AEFQDALEK	2	0.972	1
						AEMADQAAAWLTR	2	0.9994	2
						ALDALSAYWIASHTTEER	2	0.9905	2
						ALSAYWIASHTTEER	2	0.9519	2
						ASAGLLGAHAAAITAYALTLTK	2	0.9997	2
						ASAGLLGAHAAAITAYALTLTK	3	0.9995	3
						AVGSGATFSHYYY	2	0.9477	1
						AYWIASHTTEER	2	0.9874	3
						CSVFYGAPSK	2	0.9985	2
						DALSAYWIASHTTEER	2	0.9929	4
						DHAVDLIQK	2	0.9992	1
						ECVGFEAVQEVPVGLVQPASATLYDYYNPER	3	0.9734	1
						ECVGFEAVQEVPVGLVQPASATLYDYYNPERR	3	0.9997	3
						EFHLHLR	2	0.899	1
						EGAIHREELVYELNPLDHR	3	0.9984	4
						FACYYPR	2	0.9663	1
						FGLLDEDGKK	2	0.9982	2
						GLCVATPVQLR	2	0.9591	1
						GLEEELQF	2	0.8724	1
						GLEEELQFSLGSK	2	0.9997	5
						GLQDEDGYR	2	0.9763	2
						GSFEFPVGDAVSK	2	0.9995	7
						GSSTWLTAFVLK	2	0.9995	4
						HLVPGAPFLLQ	2	0.8824	1
						HLVPGAPFLLQALVR	2	0.9751	1
						HLVPGAPFLLQALVR	3	0.9997	5
						KADGSYAAWLSR	2	0.9994	4
						KADGSYAAWLSR	3	0.9991	4

						KYVLPNFEVK	3	0.9942	1
						LGQYASPTAK	2	0.9992	1
						LHLETDSLALVALGALDTALYAAGSK	3	0.9994	3
						LLATLCSAEVCQCAEGK	2	0.9992	3
						LLLHEGK	2	0.8616	1
						LNMGITDLQGLR	2	0.9901	1
						QGSFQGGFR	2	0.9945	1
						SAYWIASHTTEER	2	0.9807	2
						SFFPENWLWR	2	0.9994	5
						SHALQLNNR	2	0.9995	4
						STQDTVIALDALSAYWIASH	2	0.8476	1
						STQDTVIALDALSAYWIASHTTEER	3	0.9995	5
						TAYALTLTK	2	0.952	1
						TTNIQGINLLFSSR	2	0.9995	3
						TYNVLDMK	2	0.8476	1
						VDFTLSSER	2	0.9983	1
						VEYGFQVK	2	0.998	1
						VGDTLNLNLR	2	0.9988	3
						VGLSGMAIADVTLLSGFHALR	3	0.9996	4
						VHYTVCIWR	2	0.9994	2
						VHYTVCIWR	3	0.9969	2
						VLSLAQEQVGGSPEK	2	0.9997	2
						VLSLAQEQVGGSPEKL	2	0.9811	2
						VTASDPLDTLGSEGALSPGGVASLLR	2	0.9997	4
						VTASDPLDTLGSEGALSPGGVASLLR	3	0.9989	4
						WIASHTTEER	2	0.9957	1
						YIYGKPVQGVAY	2	0.8997	1
						YIYGKPVQGVAYVR	3	0.9987	2
						YVSHFETEGPHVLLYFDSVPTSR	3	0.9996	5
						YWIASHTTEER	2	0.949	2
Q$652-1	P0CG04	LAC1_HUMAN	IGLC1	Ig lambda-1 chain C regions	1.0000	47.17	4	4	13
						ATLVCLISDFYPGAVTVAWK	3	0.9997	5
						SYSCQVTHEGSTVE	2	0.9852	2
						SYSCQVTHEGSTVEK	2	0.9997	2
						YAASSYLSLTPEQWK	2	0.9997	4
Q$653-1	P0CG05	LAC2_HUMAN	IGLC2	Ig lambda-2 chain C regions	1.0000	65.09	8	10	30
Q$653-2	P0CG06	LAC3_HUMAN	IGLC3	Ig lambda-3 chain C regions	1.0000	65.09	8	10	30
						AAPSVTLFPPSSEELQANK	2	0.9996	8
						ATLVCLISDFYPGAVTV	2	0.9435	1
						ATLVCLISDFYPGAVTVAWK	2	0.9997	4
						ATLVCLISDFYPGAVTVAWK	3	0.9997	5
						SYLSLTPEQWK	2	0.896	1
						SYSCQVTHEGSTV	2	0.9899	2
						SYSCQVTHEGSTVE	2	0.9852	2
						SYSCQVTHEGSTVEK	2	0.9997	2
						YAASSYLSLTPEQWK	2	0.9997	4
						YAASSYLSLTPEQWK	3	0.9964	1
Q$654-1	P0DME0	SETLP_HUMAN	SETSIP	Protein SETSIP	1.0000	16.56	5	5	14
						EFHLNESGDPSSK	2	0.9994	2
						IDFYFDENPYFENK	2	0.9997	3
						LRQPFFQKR	3	0.9708	1
						SGYRIDFYFDENPYFENK	3	0.9971	4
						VEVTEFEDIK	2	0.9997	4
Q$655-1	P10153	RNAS2_HUMAN	RNASE2	Non-secretory ribonuclease	1.0000	29.81	6	10	23
						DPPQYPVVPVHLDR	2	0.9958	3
						DPPQYPVVPVHLDR	3	0.9995	2
						DPPQYPVVPVHLDRII	3	0.9334	1
						NQNTFLLTTFANVV	2	0.8368	1
						RDPPQYPVVPVHLDR	2	0.9996	3
						RDPPQYPVVPVHLDR	3	0.9997	4
						RDPPQYPVVPVHLDR	4	0.999	1
						RDPPQYPVVPVHLDRII	3	0.9993	2
						YAQTPANMFYIVACDNR	2	0.9997	4
						YAQTPANMFYIVACDNR	3	0.9996	2
Q$656-1	P10253	LYAG_HUMAN	GAA	Lysosomal alpha-glucosidase	0.9954	2	2	2	2
						FDCAPDK	2	0.9468	1
						VTSEGAGLQLQK	2	0.9141	1
Q$657-1	P10451-2	OSTP_HUMAN	SPP1	Isoform B of Osteopontin	1.0000	12.67	4	4	30
						AIPVAQDLNAPSDWDSR	2	0.9997	22
						GDSVVYGLR	2	0.9997	3
						LNAPSDWDSR	2	0.9849	3
						QNLLAPETLPSK	2	0.9989	2
Q$658-1	P10451-5	OSTP_HUMAN	SPP1	Isoform 5 of Osteopontin	1.0000	38.67	22	22	68
						AIPVAQDLNAPSD	2	0.9702	1
						AIPVAQDLNAPSDWDSR	2	0.9997	22
						AVATWLNPDPSQK	2	0.9969	3
						DLNAPSDWDSR	2	0.8173	1
						EHSDVIDSQELSK	2	0.8529	1
						FRISHELDSASSEVN	2	0.935	2
						GDSVVYGLR	2	0.9997	3
						GKDSYETSQLDDQSAETH	2	0.9872	2
						GKDSYETSQLDDQSAETHSHK	2	0.9996	3
						GKDSYETSQLDDQSAETHSHKQ	3	0.9135	1
						IPVAQDLNAPSDWDSR	2	0.9971	2
						ISHELDSASSEVN	2	0.9996	4
						KANDESNEHSDVIDSQELSK	2	0.9997	2
						LNAPSDWDSR	2	0.9849	3
						PDAVATWLNPDPSQK	2	0.9943	3
						QLYNKYPDAVATWLN	2	0.8152	1
						QLYNKYPDAVATWLNPDPS	2	0.8754	1
						QLYNKYPDAVATWLNPDPSQ	2	0.8621	1
						QNLLAPQTLPSK	2	0.9997	2
						VAQDLNAPSDWDSR	2	0.9936	2
						WLNPDPSQK	2	0.9731	1
						YPDAVATWLNPDPSQK	2	0.9995	7
Q$659-1	P10451-4	OSTP_HUMAN	SPP1	Isoform D of Osteopontin	1.0000	17.81	6	6	31
Q$659-2	P10451	OSTP_HUMAN	SPP1	Osteopontin	1.0000	16.56	6	6	31
						AIPVAQDLNAPSDWDSR	2	0.9997	22
						AVSSEETNDFKQETLPSK	2	0.9481	1
						GDSVVYGLR	2	0.9997	3
						LNAPSDWDSR	2	0.9849	3
						QNLLAPQNAVSSEETNDFK	2	0.9924	1
						QNLLAPQNAVSSEETNDFKQETLPSK	3	0.9823	1
Q$660-1	P10643	CO7_HUMAN	C7	Complement component C7	0.9601	2.37	1	1	2
						LIDQYGTHYLQSGSLGGEYR	3	0.9601	2
Q$661-1	P10809	CH60_HUMAN	HSPD1	60 kDa heat shock protein, mitochondrial	1.0000	11.52	6	6	7
						AAVEEGIVLGGGCALLR	2	0.9989	2
						CEFQDAYVLLSEK	2	0.9991	1
						IGIEIIKR	2	0.9735	1
						NAGVEGSLIVEK	2	0.9777	1
						RGVMLAVDAVIAELK	3	0.9982	1
						RGVMLAVDAVIAELKK	3	0.9335	1
Q$662-1	P10909-2	CLUS_HUMAN	CLU	Isoform 2 of Clusterin	1.0000	22.75	18	23	47
						ASSIIDELFQDR	2	0.9996	5
						ASSIIDELFQDR	3	0.9865	1
						ASSIIDELFQDRF	2	0.9828	2
						CREILSVDCSTNNPSQ	2	0.9379	2
						CREILSVDCSTNNPSQAK	3	0.996	1
						DQTVSDNELQEMSNQGSK	2	0.9979	2
						EILSVDCSTNNPSQ	2	0.9832	4
						EILSVDCSTNNPSQA	2	0.9688	1
						EILSVDCSTNNPSQAK	2	0.9997	4
						ELDESLQVAER	2	0.9995	3

						KTLLSNLEEAK	2	0.9968	1
						KTLLSNLEEAK	3	0.9756	1
						KTLLSNLEEAKK	2	0.9974	1
						KTLLSNLEEAKK	3	0.9774	1
						LFDSDPITVTVPVEVSR	2	0.9997	3
						RELDESLQVAER	2	0.9995	3
						RELDESLQVAER	3	0.9956	1
						RVTTVASHTSDSDVPSGVTEVVVK	3	0.8416	1
						VPSGVTEVVVK	2	0.9664	1
						VTTVASHTSDSDVPSG	2	0.9935	2
						VTTVASHTSDSDVPSGVTE	2	0.8882	2
						VTTVASHTSDSDVPSGVTEVVVK	2	0.9996	2
						VTTVASHTSDSDVPSGVTEVVVK	3	0.9991	3
Q$663-1	P10909	CLUS_HUMAN	CLU	Clusterin	1.0000	18.04	10	11	30
Q$663-2	P10909-3	CLUS_HUMAN	CLU	Isoform 3 of Clusterin	1.0000	29.56	10	11	30
Q$663-3	P10909-4	CLUS_HUMAN	CLU	Isoform 4 of Clusterin	1.0000	19.47	10	11	30
Q$663-4	P10909-5	CLUS_HUMAN	CLU	Isoform 5 of Clusterin	1.0000	17.61	10	11	30
						ASSIIDELFQDR	2	0.9996	5
						EILSVDCSTNNPSQ	2	0.9832	4
						EILSVDCSTNNPSQAK	2	0.9997	4
						ELDESLQVAER	2	0.9995	3
						LFDSDPITVTVPVEVSR	2	0.9997	3
						RELDESLQVAER	2	0.9995	3
						RELDESLQVAER	3	0.9956	1
						RVTTVASHTSDSDVPSGVTEVVVK	3	0.8416	1
						VPSGVTEVVVK	2	0.9664	1
						VTTVASHTSDSDVPSG	2	0.9935	2
						VTTVASHTSDSDVPSGVTEVVVK	3	0.9991	3
Q$664-1	P11021	GRP78_HUMAN	HSPA5	78 kDa glucose-regulated protein	1.0000	14.98	8	9	18
						AKFEELNMDLFR	2	0.9763	2
						AKFEELNMDLFR	3	0.9925	2
						IEIESFYEGEDFSETLTR	2	0.9997	3
						IINEPTAAAIAYGLDK	2	0.9947	2
						IINEPTAAAIAYGLDKR	3	0.9632	1
						ITPSYVAFTPEGER	2	0.9991	2
						TFAPEEISAMVLTK	2	0.9988	2
						TWNDPSVQQDIK	2	0.9994	2
						VEIIANDQGNR	2	0.9988	2
Q$665-1	P11047	LAMC1_HUMAN	LAMC1	Laminin subunit gamma-1	1.0000	3.73	4	4	4
						EAQQALGSAAADATEAK	2	0.9984	1
						NTIEETGNLAEQAR	2	0.9535	1
						SQECYFDPELYR	2	0.9854	1
						TFAEVTDLDNEVNNMLK	2	0.9923	1
Q$666-1	P11215	ITAM_HUMAN	ITGAM	Integrin alpha-M	0.9982	0.87	1	1	2
Q$666-2	P11215-2	ITAM_HUMAN	ITGAM	Isoform 2 of Integrin alpha-M	0.9982	0.87	1	1	2
						YVIGVGDAFR	2	0.9708	2
Q$667-1	P11216	PYGB_HUMAN	PYGB	Glycogen phosphorylase, brain form	0.9712	1.9	1	1	1
						LQDFNVGDYIEAVLDR	2	0.9712	1
Q$668-1	P11678	PERE_HUMAN	EPX	Eosinophil peroxidase	1.0000	10.63	7	7	11
						DFLPLVLGK	2	0.9929	1
						FCGLSQPR	2	0.9789	1
						IICDNTGITTVSR	2	0.9986	2
						IPCFLAGDTR	2	0.9975	2
						IVYEGGIDPILR	2	0.9978	2
						VANVFTLAFR	2	0.9994	2
						VGPLLACLFENQFR	2	0.9935	1
Q$669-1	P12109	CO6A1_HUMAN	COL6A1	Collagen alpha-1(VI) chain	1.0000	2.33	2	2	2
						TAEYDVAYGESHLFR	3	0.99	1
						VPSYQALLR	2	0.9801	1
Q$670-1	P12259	FA5_HUMAN	F5	Coagulation factor V	0.9845	0.45	1	1	2
						AEVDDVIQVR	2	0.9721	2
Q$671-1	P12273	PIP_HUMAN	PIP	Prolactin-inducible protein	0.9999	15.07	2	2	3
						FYTIEILKVE	2	0.9654	1
						TVQIAAVVDVIR	2	0.9967	2
Q$672-1	P12277	KCRB_HUMAN	CKB	Creatine kinase B-type	1.0000	17.06	5	5	7
						FCTGLTQIETLFK	2	0.9996	2
						LAVEALSSLDGDLAGR	2	0.9559	1
						LGFSEVELVQMVVDGVK	2	0.9994	2
						LLIEMEQR	2	0.9478	1
						VLTPELYAELR	2	0.9896	1
Q$673-1	P12724	ECP_HUMAN	RNASE3	Eosinophil cationic protein	1.0000	36.88	13	15	40
						ANVVNVCGNQSIR	2	0.9884	1
						AQWFAIQHI	2	0.9967	2
						AQWFAIQHISLNPPR	2	0.9996	6
						AQWFAIQHISLNPPR	3	0.9995	5
						DSPRYPVVPVHLDTTI	2	0.9969	2
						FANVVNVCGNQSIR	2	0.9459	2
						FYVVACDNR	2	0.9996	1
						FYVVACDNRD	2	0.9521	3
						FYVVACDNRDPR	3	0.9952	2
						NVVNVCGNQSIR	2	0.9695	2
						TTFANVVNVCGNQSIR	2	0.9996	4
						TTFANVVNVCGNQSIR	3	0.9983	2
						YPVVPVHL	2	0.9381	1
						YPVVPVHLD	2	0.9846	1
						YPVVPVHLDTTI	2	0.9996	6
Q$674-1	Q9UII8	Q9UII8_HUMAN	CDH1	Cadherin 1, type 1, E-cadherin (Epithelial), isoform CRA_c	0.9993	4.63	2	2	2
Q$674-2	P12830	CADH1_HUMAN	CDH1	Cadherin-1	0.9993	4.31	2	2	2
						DTANWLEINPDTGAISTR	2	0.9919	1
						YLPRPANPDEIGNFIDENLK	3	0.9191	1
Q$675-1	P12838	DEF4_HUMAN	DEFA4	Neutrophil defensin 4	1.0000	17.53	2	2	7
						VGNCLIGGVSFTY	2	0.8337	1
						VGNCLIGGVSFTYCCTR	2	0.9995	6
Q$676-1	P12931-2	SRC_HUMAN	SRC	Isoform 2 of Proto-oncogene tyrosine-protein kinase Src	0.9325	3.69	1	1	1
Q$676-2	P12931	SRC_HUMAN	SRC	Proto-oncogene tyrosine-protein kinase Src	0.9325	3.73	1	1	1
						AGPLAGGVTTFVALYDYESR	2	0.9325	1
Q$677-1	P12955-2	PEPD_HUMAN	PEPD	Isoform 2 of Xaa-Pro dipeptidase	0.9750	4.42	1	1	1
Q$677-2	P12955-3	PEPD_HUMAN	PEPD	Isoform 3 of Xaa-Pro dipeptidase	0.9750	4.66	1	1	1
Q$677-3	P12955	PEPD_HUMAN	PEPD	Xaa-Pro dipeptidase	0.9750	4.06	1	1	1
						IEEDVVVTDSGIELLTCVPR	2	0.975	1
Q$678-1	P12956	XRCC6_HUMAN	XRCC6	X-ray repair cross-complementing protein 6	1.0000	6.57	3	3	4
						DIISIAEDEDLR	2	0.983	1
						NIYVLQELDNPGAK	2	0.9997	2
						SDSFENPVLQQHFR	2	0.9987	1
Q$679-1	P13639	EF2_HUMAN	EEF2	Elongation factor 2	1.0000	30.65	18	22	43
						AGIIASAR	2	0.9828	1
						ALLELQLEPEELYQTFQR	2	0.9997	3
						ALLELQLEPEELYQTFQR	3	0.9996	3
						AYLPVNESFGFTADLR	2	0.9997	2
						AYLPVNESFGFTADLR	3	0.9631	1
						CLYASVLTAQPR	2	0.9993	2
						EGIPALDNFLDKL	2	0.999	2
						ETVSEESNVLCLSK	2	0.9997	3
						FSVSPVVR	2	0.9615	1
						GEGQLGPAER	2	0.9725	1
						GGGQIIPTAR	2	0.9979	1
						GHVFEESQVAGTPMFVVK	3	0.8772	1
						GVQYLNEIKDSVVAGFQWATK	3	0.9813	2
						KIWCFGPDGTGPNILTDITK	3	0.9989	3
						STAISLFYELSENDLNFIK	2	0.9996	3
						STAISLFYELSENDLNFIK	3	0.9988	2
						TFCQLILDPIFK	2	0.9993	3
						TGTITTFEHAHNMR	2	0.9994	1

						VFSGLVSTGLK	2	0.8715	1
						YLAEKYEWDVAEAR	2	0.9995	3
						YLAEKYEWDVAEAR	3	0.9981	1
						YVEPIEDVPCGNIVGLVGVDQFLVK	3	0.9996	3
Q$680-1	P13645	K1C10_HUMAN	KRT10	Keratin, type I cytoskeletal 10	1.0000	71.06	30	40	92
						ADLEMQIESLTEELAYLKK	2	0.9995	2
						ADLEMQIESLTEELAYLKK	3	0.9996	3
						AETECQNTEYQQLLDIK	2	0.9997	3
						AETECQNTEYQQLLDIK	3	0.9982	2
						ALEESNYELEGK	2	0.9997	4
						DAEAWFNEK	2	0.9995	2
						ELTTEIDNNIEQISSYK	2	0.9997	4
						ELTTEIDNNIEQISSYKSEITELRR	3	0.9109	1
						GGGFSSGGFSGGSFSR	2	0.8942	1
						GGGSFGGGYGGGSSGGGSSGGGHGGGHGGSSGGGYGGGSSGGGSSGGGYGGGSSSGGHGGSSSGGYGGGSSGG	4	0.8584	1
						GSLGGGFSSGGFSGGSFSR	2	0.9997	2
						GSSGGGCFGGSSGGYGGLGGFGGGSFR	2	0.9997	3
						GSSGGGCFGGSSGGYGGLGGFGGGSFR	3	0.9995	3
						IKEWYEK	2	0.9881	1
						IRLENEIQTYR	2	0.999	1
						IRLENEIQTYR	3	0.9237	1
						LENEIQTYR	2	0.9996	2
						LKYENEVALR	2	0.9899	1
						LKYENEVALR	3	0.9657	1
						NHEEEMKDLR	2	0.9987	2
						NQILNLTTDNANILLQIDNAR	2	0.9997	4
						NQILNLTTDNANILLQIDNAR	3	0.9996	4
						NVQALEIELQSQLALK	2	0.9997	4
						NVQALEIELQSQLALK	3	0.9987	3
						NVQALEIELQSQLALKQSLEASLAETEGR	3	0.9967	2
						NVSTGDVNVEMNAAPGVDLTQLLNNMR	3	0.9996	6
						QILNLTTDNANILLQIDNAR	3	0.9302	2
						QSLEASLAETEGR	2	0.9996	1
						QSVEADINGLRR	2	0.9455	1
						SKELTTEIDNNIEQISSYK	2	0.9991	1
						SKELTTEIDNNIEQISSYK	3	0.9977	3
						SLLEGEGSSGGGGR	2	0.9996	1
						SQYEQLAEQNR	2	0.9995	2
						SSSSGSVGESSSK	2	0.8269	1
						TIDDLKNQILNLTTDNANILLQIDNAR	3	0.9994	6
						TIDDLKNQILNLTTDNANILLQIDNAR	4	0.9666	3
						VLDELTLTK	2	0.9976	1
						VTMQNLNDR	2	0.9989	1
						YCVQLSQIQAQISALEEQLQQIR	2	0.9994	2
						YCVQLSQIQAQISALEEQLQQIR	3	0.9995	4
Q$681-1	P13647	K2C5_HUMAN	KRT5	Keratin, type II cytoskeletal 5	1.0000	35.08	19	20	32
						AQYEEIANR	2	0.9993	2
						DVDAAYMNKVELEAK	2	0.9986	2
						FLEQQNKVLDTK	2	0.9015	1
						GLGVGFGSGGGSSSSVK	2	0.9988	2
						ISISTSGGSFR	2	0.8509	1
						LAELEEALQK	2	0.9991	1
						LRAEIDNVKK	2	0.9501	1
						NKLAELEEALQK	2	0.8098	1
						NMQDLVEDFK	2	0.9712	2
						QCANLQNAIADAEQR	2	0.9987	2
						QNLEPLFEQYINNLR	2	0.9977	3
						QNLEPLFEQYINNLR	3	0.9825	1
						RQLDSIVGER	2	0.9846	1
						SRTEAESWYQTK	2	0.9845	2
						SRTEAESWYQTKYEELQQTAGR	3	0.995	3
						TTAENEFVMLKK	2	0.9889	1
						VDALMDEINFMK	2	0.9996	3
						VSLAGACGVGGYGSR	2	0.9991	1
						WTLLQEQGTK	2	0.9985	1
						YEELQQTAGR	2	0.9994	1
Q$682-1	P13667	PDIA4_HUMAN	PDIA4	Protein disulfide-isomerase A4	1.0000	8.06	4	4	7
						EVSQPDWTPPPEVTLVLTK	2	0.9993	3
						FDVSGYPTIK	2	0.9944	2
						IDATSASVLASR	2	0.9996	1
						VDATAETDLAK	2	0.9943	1
Q$683-1	P13671	CO6_HUMAN	C6	Complement component C6	0.9535	1.18	1	1	1
						SEYGAALAWEK	2	0.9366	1
Q$684-1	P13727	PRG2_HUMAN	PRG2	Bone marrow proteoglycan	1.0000	34.68	8	11	25
						FQWVDGSR	2	0.9766	1
						GGHCVALCTR	2	0.9995	4
						GNLVSIHNFNINYR	2	0.9993	3
						GNLVSIHNFNINYR	3	0.9992	1
						IQCSVSALNQGQVWIGGR	2	0.9995	1
						SALNQGQVWIGGR	2	0.8745	2
						SLQTFSQAWFTCR	2	0.9996	4
						SLQTFSQAWFTCR	3	0.9968	3
						SVSALNQGQVWIGGR	2	0.9862	2
						WNFAYWAAHQPWSR	2	0.9929	2
						WNFAYWAAHQPWSR	3	0.9738	2
Q$685-1	P14136	GFAP_HUMAN	GFAP	Glial fibrillary acidic protein	1.0000	2.08	1	1	1
Q$685-2	P14136-2	GFAP_HUMAN	GFAP	Isoform 2 of Glial fibrillary acidic protein	1.0000	2.05	1	1	1
Q$685-3	P14136-3	GFAP_HUMAN	GFAP	Isoform 3 of Glial fibrillary acidic protein	1.0000	2.09	1	1	1
						LEEEGQSLK	2	0.8442	1
Q$686-1	P14174	MIF_HUMAN	MIF	Macrophage migration inhibitory factor	0.9608	7.83	1	1	1
						LLCGLLAER	2	0.9608	1
Q$687-1	P14543-2	NID1_HUMAN	NID1	Isoform 2 of Nidogen-1	1.0000	16.7	10	11	41
Q$687-2	P14543	NID1_HUMAN	NID1	Nidogen-1	1.0000	14.92	10	11	41
						AFSQGSVGFLWK	2	0.8278	1
						EDLSPSITQR	2	0.9995	1
						ENNQVPAVVAFSQGSVGFLWK	3	0.9327	1
						ESHPGLFPPTFGAVAPFLADLDTTDGLGK	3	0.9997	19
						GFPEISFQPSSAVVVTWESVAPYQGPSR	3	0.9929	1
						GFPEISFQPSSAVVVTWESVAPYQGPSRDPDQK	3	0.9986	5
						NTFQAVLASSDSSSYAIFLYPEDGLQFHTTFSKK	4	0.9757	1
						QELFPFGPGQGDLELEDGDDFVSPALELSGALR	3	0.9995	8
						SDIDAVYVTTNGIIATSEPPAK	2	0.9994	1
						VVYREDLSPSITQR	2	0.9987	2
						VVYREDLSPSITQR	3	0.9132	1
Q$688-1	P14550	AK1A1_HUMAN	AKR1A1	Alcohol dehydrogenase [NADP(+)]	1.0000	32.31	8	8	12
						ALEALVAK	2	0.9823	1
						AWRDPDEPVLLEEPVVLALAEK	3	0.9907	2
						GLEVTAYSPLGSSDR	2	0.9997	3
						GLVQALGLSNFNSR	2	0.9996	1
						HIDCAAIYGNEPEIGEAL	2	0.9781	1
						HIDCAAIYGNEPEIGEALKEDVGPGK	3	0.9966	1
						MPILGLGTWK	2	0.9869	1
						MPLIGLGTWK	2	0.9893	2
Q$689-1	P14618	KPYM_HUMAN	PKM	Pyruvate kinase PKM	1.0000	25.24	11	11	23
						APIIAVTR	2	0.9583	1
						CCSGAIIVLTK	2	0.9901	2
						CDENILWLDYK	2	0.9995	3
						EAEAAIYHLQLFEELR	3	0.9197	1
						FGVEQDVDMVFASFIR	2	0.9996	1
						GDLGIEIPAEK	2	0.9883	2
						GSGTAEVELKK	2	0.9897	1
						IYVDDGLISLQVK	2	0.9997	3
						LDIDSPPITAR	2	0.9985	3

						NTGIICTIGPASR	2	0.9996	3
						PVQEAWAEDVDLR	2	0.9952	3
Q$690-1	P14625	ENPL_HUMAN	HSP90B1	Endoplasmin	1.0000	19.05	12	12	15
						DISTNYYASQKK	2	0.9665	2
						EAESSPFVER	2	0.9977	1
						FAFQAEVNR	2	0.9993	1
						FQSSHHPTDITSLDQYVER	3	0.9711	1
						GYEVIYLTEPVDEYCIQALPEFDGKR	3	0.9965	1
						KEAESSPFVER	2	0.9834	1
						LGVIEDHSNR	2	0.9979	2
						LIINSLYK	2	0.928	1
						LISLTDENALSGNEELTVK	2	0.9981	1
						NLLHVTDTGVGMTREELVK	3	0.9345	1
						SGTSEFLNK	2	0.9917	1
						SILFVPTSAPR	2	0.9986	2
Q$691-1	P14780	MMP9_HUMAN	MMP9	Matrix metalloproteinase-9	0.9968	4.1	2	2	4
						AFALWSAVTPLTFTR	2	0.9755	2
						QVWVYTGASVLGPR	2	0.9778	2
Q$692-1	P14868	SYDC_HUMAN	DARS	Aspartate--tRNA ligase, cytoplasmic	1.0000	10.98	5	5	6
Q$692-2	Q68CR9	Q68CR9_HUMAN	DKFZp781B11202	Aspartate--tRNA ligase, cytoplasmic	1.0000	13.72	5	5	6
						ESIVDVEGVVR	2	0.9923	1
						FQTEIQTVNK	2	0.9517	1
						IGSCTQQDVELHVQK	3	0.9871	1
						IYVISLAEPR	2	0.9987	2
						VFSIGPVFR	2	0.9542	1
Q$693-1	P14923	PLAK_HUMAN	JUP	Junction plakoglobin	1.0000	7.38	4	4	5
						LNTIPLFVQLLYSSVENIQR	3	0.9877	1
						LVQNCLWTLR	2	0.8793	1
						NEGTATYAAAVLFR	2	0.9994	2
						VSVELTNSLFK	2	0.9635	1
Q$694-1	P15121	ALDR_HUMAN	AKR1B1	Aldose reductase	1.0000	42.41	10	10	19
						HIDCAHVYQNENEVGVAIQEK	3	0.9995	3
						LDYLDLYLIHWPTGFKPGK	3	0.9963	3
						LIQYCQSK	2	0.9678	1
						MPILGLGTWK	2	0.9869	1
						MPLIGLGTWK	2	0.9893	2
						REELFIVSK	2	0.9744	1
						SPPGQVTEAVK	2	0.9987	2
						VAIDVGYR	2	0.9996	1
						VCALLSCTSHKDYPFHEEF	3	0.9639	2
						VFDFELSSQDMTTLLSYNR	2	0.9996	3
Q$695-1	P15153	RAC2_HUMAN	RAC2	Ras-related C3 botulinum toxin substrate 2	1.0000	29.69	5	5	8
						CVVVGDGAVGK	2	0.9993	4
						LRPLSYPQTDVFLICFSLVSPASYENVR	3	0.8744	1
						SLVSPASYENVR	2	0.9155	1
						TVFDEAIR	2	0.8878	1
						YLECSALTQR	2	0.9867	1
Q$696-1	P18669	PGAM1_HUMAN	PGAM1	Phosphoglycerate mutase 1	1.0000	9.84	2	2	3
Q$696-2	P15259	PGAM2_HUMAN	PGAM2	Phosphoglycerate mutase 2	1.0000	9.88	2	2	3
						ALPFWNEEIVPQIK	2	0.9971	2
						VLIAAHGNSLR	2	0.9987	1
Q$697-1	P15374	UCHL3_HUMAN	UCHL3	Ubiquitin carboxyl-terminal hydrolase isozyme L3	0.9993	10.87	2	2	2
						WLPLEANPEVTNQFLK	2	0.993	1
						YLENYDAIR	2	0.9006	1
Q$698-1	P15428	PGDH_HUMAN	HPGD	15-hydroxyprostaglandin dehydrogenase [NAD(+)]	1.0000	45.49	10	11	25
						AALDEQFEPQK	2	0.9997	4
						AFAEALLLK	2	0.9996	2
						GIHFQDYDTTPFQAK	2	0.9997	4
						HGIVGFTR	2	0.9965	2
						LDILVNNAGVNNEK	2	0.9997	2
						SAALAANLMNSGVR	2	0.9995	2
						TLFIQCDVADQQQLR	2	0.9997	3
						TLFIQCDVADQQQLR	3	0.9979	2
						VALVDWNLEAGVQCK	2	0.9997	2
						VALVTGAAQGIGR	2	0.9997	1
						VVDHFGR	2	0.9946	1
Q$699-1	P15428-2	PGDH_HUMAN	HPGD	Isoform 2 of 15-hydroxyprostaglandin dehydrogenase [NAD(	1.0000	43.26	7	7	14
						AALDEQFEPQK	2	0.9997	4
						AFAEALLLK	2	0.9996	2
						HGIVGFTR	2	0.9965	2
						LDILVNNAGVNNEK	2	0.9997	2
						VALVDWNLEAGVQCK	2	0.9997	2
						VALVTGAAQGIGR	2	0.9997	1
						VVDHFGR	2	0.9946	1
Q$700-1	P15428-5	PGDH_HUMAN	HPGD	Isoform 5 of 15-hydroxyprostaglandin dehydrogenase [NAD(	1.0000	50.51	8	9	22
						AALDEQFEPQK	2	0.9997	4
						AFAEALLLK	2	0.9996	2
						GIHFQDYDTTPFQAK	2	0.9997	4
						HGIVGFTR	2	0.9965	2
						SAALAANLMNSGVR	2	0.9995	2
						TLFIQCDVADQQQLR	2	0.9997	3
						TLFIQCDVADQQQLR	3	0.9979	2
						VALVDWNLEAGVQCK	2	0.9997	2
						VALVTGAAQGIGR	2	0.9997	1
Q$701-1	P16152	CBR1_HUMAN	CBR1	Carbonyl reductase [NADPH] 1	1.0000	51.62	12	13	22
						DVCTELLPLIKPQGR	2	0.9995	3
						DVCTELLPLIKPQGR	3	0.9973	2
						EYGGLDVLVNNAGIAFK	2	0.9992	2
						FHQLDIDDLQSIR	3	0.9993	2
						FRSETITEEELVGLMNK	3	0.949	1
						GIGLAIVR	2	0.9939	1
						GQAAVQQLQAEGLSPR	2	0.9995	2
						IGVTVLSR	2	0.9933	1
						ILLNACCPGWVR	2	0.9995	3
						LFSGDVVLTAR	2	0.9908	1
						LGVTVLSR	2	0.9874	1
						TNFFGTR	2	0.9992	1
						VVNVSSIMSVR	2	0.9983	2
Q$702-1	P16444	DPEP1_HUMAN	DPEP1	Dipeptidase 1	0.9478	4.87	1	1	1
						VPEGLEDVSKYPDLIAELLR	3	0.9478	1
Q$703-1	P17858	PFKAL_HUMAN	PFKL	ATP-dependent 6-phosphofructokinase, liver type	1.0000	11.67	8	9	12
Q$703-2	P17858-2	PFKAL_HUMAN	PFKL	Isoform 2 of ATP-dependent 6-phosphofructokinase, liver typ	1.0000	11	8	9	12
						GQLESIVENIR	2	0.9602	1
						IMEVIDAITTTAQSHQR	3	0.8135	1
						LNIIIIAEGAIDR	2	0.9801	2
						MGIYVGAK	2	0.8421	1
						SEWGSLLEELVAEGK	2	0.9992	2
						SEWGSLLEELVAEGK	3	0.9891	1
						TFVLEVMGR	2	0.9965	2
						VNVEHMTEK	2	0.9536	1
						VTVLGHVQR	2	0.9913	1
Q$704-1	P17900	SAP3_HUMAN	GM2A	Ganglioside GM2 activator	1.0000	13.99	2	2	3
						SEFVVPDLELPSWLTTGNYR	2	0.9996	2
						VDLVLEK	2	0.9751	1
Q$705-1	P17987	TCPA_HUMAN	TCP1	T-complex protein 1 subunit alpha	1.0000	17.81	9	9	13
						EQLAIAEFAR	2	0.9985	2
						FATEAAITILR	2	0.9996	2
						IACLDFSLQK	2	0.9871	1
						ICDDELILIK	2	0.9962	2
						LLEVEHPAAK	2	0.9991	1
						SQNVMAAASIANIVK	2	0.9914	1
						SSLGPVGLDK	2	0.9919	1
						TSASIILR	2	0.9936	1

						YINENLIVNTDELGR	2	0.9995	2
Q$706-1	P18085	ARF4_HUMAN	ARF4	ADP-ribosylation factor 4	1.0000	57.22	7	7	11
						DAVLLLFANK	2	0.9979	1
						ILMVGLDAAGK	2	0.9979	2
						IQEVADELQK	2	0.9944	1
						LGEIVTTIPTIGFNVETVEYK	2	0.9984	2
						MLLVDELR	2	0.9955	1
						NICFTVWDVGGQDR	2	0.9784	2
						TWYVQATCATQGTGLYEGLDWLSNELSKR	3	0.999	2
Q$707-1	P18206-2	VINC_HUMAN	VCL	Isoform 1 of Vinculin	0.9715	1.78	1	1	1
Q$707-2	P18206-3	VINC_HUMAN	VCL	Isoform 3 of Vinculin	0.9715	8.56	1	1	1
Q$707-3	P18206	VINC_HUMAN	VCL	Vinculin	0.9715	1.68	1	1	1
						GILSGTSDLLLTFDEAEVR	2	0.9715	1
Q$708-1	P19012	K1C15_HUMAN	KRT15	Keratin, type I cytoskeletal 15	1.0000	5.04	1	1	1
						GGSLLAGGGGFGGGSLSGGGGSR	3	0.9291	1
Q$709-1	P19021-2	AMD_HUMAN	PAM	Isoform 2 of Peptidyl-glycine alpha-amidating monooxygenas	0.9984	2.66	2	2	2
Q$709-2	P19021-3	AMD_HUMAN	PAM	Isoform 3 of Peptidyl-glycine alpha-amidating monooxygenas	0.9984	2.54	2	2	2
Q$709-3	P19021-4	AMD_HUMAN	PAM	Isoform 4 of Peptidyl-glycine alpha-amidating monooxygenas	0.9984	2.59	2	2	2
Q$709-4	P19021-5	AMD_HUMAN	PAM	Isoform 5 of Peptidyl-glycine alpha-amidating monooxygenas	0.9984	2.36	2	2	2
Q$709-5	P19021-6	AMD_HUMAN	PAM	Isoform 6 of Peptidyl-glycine alpha-amidating monooxygenas	0.9984	2.41	2	2	2
Q$709-6	P19021	AMD_HUMAN	PAM	Peptidyl-glycine alpha-amidating monooxygenase	0.9984	2.36	2	2	2
						IPVDEEAFVIDFKPR	3	0.9844	1
						NNLVIFHR	2	0.9	1
Q$710-1	P19224-3	UD16_HUMAN	UGT1A6	Isoform 3 of UDP-glucuronosyltransferase 1-6	1.0000	7.22	2	2	3
Q$710-2	P19224	UD16_HUMAN	UGT1A6	UDP-glucuronosyltransferase 1-6	1.0000	6.02	2	2	3
						GAGVTLNVLEMTSEDLENALK	2	0.9993	2
						SFLTAPQTEYR	2	0.9802	1
Q$711-1	P19338	NUCL_HUMAN	NCL	Nucleolin	1.0000	27.46	21	23	46
						ALELTGLK	2	0.9984	1
						EVFEDAAEIR	2	0.9996	2
						FGYVDFESAEDLEK	2	0.9997	2
						GFGFVDFNSEEDAK	2	0.9997	2
						GIAYIEFK	2	0.9995	1
						GIAYIEFKTEADAEKTFEEK	3	0.9948	3
						GLSEDTTEETLKESFDGSVR	2	0.9987	1
						GYAFIEFASFEDAK	2	0.9997	5
						GYAFIEFASFEDAKEALNSCNKR	3	0.9884	3
						KFGYVDFESAEDLEK	2	0.9997	3
						KFGYVDFESAEDLEK	3	0.9987	1
						KFGYVDFESAEDLEKALELTGLK	3	0.9916	2
						LELQGPR	2	0.9983	1
						NDLAVVDVR	2	0.9994	1
						NLPYKVTQDELK	2	0.9969	2
						NLPYKVTQDELKEVFEDAAEIR	3	0.9976	3
						SISLYYTGEK	2	0.9955	1
						SISLYYTGEKGQNQDYR	2	0.9944	1
						SKGYAFIEFASFEDAK	2	0.9334	1
						TGISDVFAK	2	0.9981	2
						TLVLSNLSYSATEETLQEVFEK	2	0.9997	4
						TLVLSNLSYSATEETLQEVFEK	3	0.9997	3
						VFGNEIK	2	0.9534	1
Q$712-1	P19652	A1AG2_HUMAN	ORM2	Alpha-1-acid glycoprotein 2	1.0000	17.91	3	3	5
						EHVAHLLFLR	3	0.96	1
						EQLGEFYEALDCLCIPR	2	0.9941	2
						WFYIASAFR	2	0.9996	2
Q$713-1	P19823	ITIH2_HUMAN	ITIH2	Inter-alpha-trypsin inhibitor heavy chain H2	1.0000	19.13	15	17	35
Q$713-2	Q5T985	Q5T985_HUMAN	ITIH2	Inter-alpha-trypsin inhibitor heavy chain H2	1.0000	19.36	15	17	35
						AEDHFSVIDFNQNIR	2	0.9992	2
						AGELEVFNGYFVHFFAPDNLDPIPK	3	0.9961	4
						AHVSFKPTVAQQR	2	0.8992	1
						AYLTINQLLAER	2	0.954	2
						ETAVDGELVVLYDVK	2	0.9994	4
						FLHVPDT	2	0.8882	1
						FYNQVSTPLLR	2	0.9695	2
						HLEVDVWVIEPQGLR	2	0.9996	2
						HLEVDVWVIEPQGLR	3	0.9942	2
						IQPSGGTNINEALLR	2	0.9993	3
						KLWAYLTINQLLAER	3	0.9958	2
						SSALDMENFR	2	0.997	2
						TEVNVLPGAK	2	0.9993	2
						VQFELHYQEVK	2	0.9974	2
						VVNNSPQPQNVVF	2	0.8102	1
						VVNNSPQPQNVVFDVQIPK	2	0.9996	2
						VVNNSPQPQNVVFDVQIPK	3	0.9932	1
Q$714-1	P20160	CAP7_HUMAN	AZU1	Azurocidin	1.0000	36.25	8	9	17
						CQVAGWGSQR	2	0.9973	1
						GPDFFTR	2	0.9993	2
						GPDFFTRV	2	0.9806	2
						HFCGGALIHA	2	0.8749	1
						HFCGGALIHAR	2	0.9965	1
						HFCGGALIHAR	3	0.9963	1
						QFPFLASIQNQGR	2	0.9986	4
						RGGICNGDGGTPLVCEGLAHGVASFSLGPCGR	3	0.9987	3
						VALFRDWIDGVLNNPGPG	2	0.9507	2
Q$715-1	P20851	C4BPB_HUMAN	C4BPB	C4b-binding protein beta chain	1.0000	42.06	9	11	26
Q$715-2	P20851-2	C4BPB_HUMAN	C4BPB	Isoform 2 of C4b-binding protein beta chain	1.0000	42.23	9	11	26
						ALLAFQESK	2	0.9997	2
						ESGMTMEELK	2	0.9981	1
						ESGMTMEELKYSLELK	2	0.9969	2
						EVEGQILGTYVCIK	2	0.9997	2
						LIQEAPKPECEK	2	0.9997	2
						LIQEAPKPECEKA	2	0.9833	2
						NLCEAMENFMQQLK	2	0.9997	3
						NLCEAMENFMQQLK	3	0.9993	3
						SQCLEDHTWAPPFPICK	2	0.9995	4
						SQCLEDHTWAPPFPICK	3	0.993	4
						YYLVGVQEQQCVDGEWSSALPVCK	3	0.8682	1
Q$716-1	P20908	CO5A1_HUMAN	COL5A1	Collagen alpha-1(V) chain	0.9999	1.58	2	2	2
						GDPGPAGLPGK	2	0.9685	1
						GNEGPPGPPGPAGSPGER	2	0.9957	1
Q$717-1	P21266	GSTM3_HUMAN	GSTM3	Glutathione S-transferase Mu 3	1.0000	18.22	3	3	5
						LLLEFTDTSYEEK	2	0.9994	2
						LTFVDFLTYDILDQNR	2	0.9992	1
						VDIIENQVMDFR	2	0.9946	2
Q$718-1	P21281	VATB2_HUMAN	ATP6V1B2	V-type proton ATPase subunit B, brain isoform	1.0000	11.94	4	5	9
						AVVGEEALTSDDLLYLEFLQK	2	0.8793	1
						LALTTAEFLAYQCEK	2	0.9911	2
						NFIAQGPYENR	2	0.9197	2
						TVFETLDIGWQLLR	2	0.9997	3
						TVFETLDIGWQLLR	3	0.9616	1
Q$719-1	P21796	VDAC1_HUMAN	VDAC1	Voltage-dependent anion-selective channel protein 1	1.0000	36.75	8	8	14
						GYGFGLIK	2	0.994	1
						LTLSALIDGK	2	0.9668	1
						LTLSALLDGK	2	0.9881	2
						TDEFQLHTNVNDGTEFGGSIYQK	3	0.9966	2
						VTQSNFAVGYK	2	0.9314	1
						WNTDNTLGTEITVEDQLAR	2	0.9997	3
						WTEYGLTFTEK	2	0.998	2
						YQIDPDACFSAK	2	0.9994	2
Q$720-1	P21810	PGS1_HUMAN	BGN	Biglycan	1.0000	13.86	5	5	8
						IQAIELEDLLR	2	0.9996	2
						LAIQFGNY	2	0.9171	1
						LGLGHNQIR	2	0.9995	1

						VGVNDFCPMGFGVK	2	0.9996	2
						VPSGLPDLK	2	0.9995	2
Q$721-1	P21964	COMT_HUMAN	COMT	Catechol O-methyltransferase	1.0000	41.33	8	12	37
Q$721-2	P21964-2	COMT_HUMAN	COMT	Isoform Soluble of Catechol O-methyltransferase	1.0000	50.68	8	12	37
						AIYKGPGSEAGP	2	0.9926	2
						DRYLPDTLLLEECGLLR	3	0.9938	3
						GSSCFECTHYQSFLEYR	2	0.9997	2
						GSSCFECTHYQSFLEYR	3	0.9984	2
						GTVLLADNVICPGAPDFLAHVR	3	0.9994	4
						IVDAVIQEHQPSVLLELGAYCGYSAVR	3	0.9981	3
						KGTVLLADNVICPGAPDFLAHVR	3	0.9993	2
						KGTVLLADNVICPGAPDFLAHVR	4	0.9249	1
						LITIEINPDCAAITQR	2	0.9997	7
						LITIEINPDCAAITQR	3	0.9994	3
						YLPDTLLLEECGLLR	2	0.9997	5
						YLPDTLLLEECGLLR	3	0.9995	3
Q$722-1	P22314	UBA1_HUMAN	UBA1	Ubiquitin-like modifier-activating enzyme 1	1.0000	18.05	12	13	22
						AAVATFLQSVQVPEFTPK	2	0.9935	2
						DNPGVVTCLDEAR	2	0.9996	2
						GGIVSQVK	2	0.9707	1
						KLAYVAAGDLAPINAFIGGLAAQEVMK	3	0.9298	2
						KPLLESGTLGTK	2	0.9982	1
						LAGTQPLEVLEAVQR	2	0.9991	3
						LQTSSVLVSGLR	2	0.9989	2
						NEEDAAELVALAQAVNAR	2	0.9963	1
						NEEDAAELVALAQAVNAR	3	0.9978	1
						SLVASLAEPDFVVTDFAK	2	0.9967	1
						VLGPYTFSICDTSNFSDYIR	2	0.9996	2
						YDGQVAVFGSDLQEK	2	0.9975	1
						YFLVGAGAIGCELLK	2	0.9997	3
Q$723-1	P22626	ROA2_HUMAN	HNRNPA2B1	Heterogeneous nuclear ribonucleoproteins A2/B1	1.0000	9.92	4	5	8
Q$723-2	P22626-2	ROA2_HUMAN	HNRNPA2B1	Isoform A2 of Heterogeneous nuclear ribonucleoproteins A2/	1.0000	10.26	4	5	8
						IDTIEIITDR	2	0.9991	1
						KLFIGGLSFETTEESLR	2	0.9992	1
						KLFIGGLSFETTEESLR	3	0.9971	1
						LFIGGLSFETTEESLR	2	0.9997	4
						NYYEQWGK	2	0.9654	1
Q$724-1	P22891-2	PROZ_HUMAN	PROZ	Isoform 2 of Vitamin K-dependent protein Z	1.0000	46.21	53	63	164
Q$724-2	P22891	PROZ_HUMAN	PROZ	Vitamin K-dependent protein Z	1.0000	48.75	53	63	164
						AGLPVCTPEKDFAEHLLIPR	3	0.9512	2
						APDLQDLPWQ	2	0.8409	1
						APDLQDLPWQV	2	0.8962	1
						APDLQDLPWQVK	2	0.9997	14
						APDLQDLPWQVK	3	0.8262	1
						APDLQDLPWQVKL	2	0.9882	3
						CLPGQESYTCSCAQGYR	2	0.9874	1
						DFAEHLLIPR	2	0.9997	3
						DFCGGVIIR	2	0.9997	6
						DFCGGVIIRE	2	0.9134	1
						DFCGGVIIREN	2	0.8656	2
						DLQDLPWQVK	2	0.95	1
						DQCACGVLTSEKR	2	0.8811	1
						ENFVLTTAK	2	0.9997	4
						FAEHLLIPR	3	0.9754	1
						FCGGVIIR	2	0.9478	1
						GLLSGWAR	2	0.9997	4
						GSWFLTGVLG	2	0.9723	1
						GSWFLTGVLGSQPV	2	0.9915	2
						GSWFLTGVLGSQPVGGQ	2	0.8255	1
						GSWFLTGVLGSQPVGGQA	2	0.9075	1
						GSWFLTGVLGSQPVGGQAH	2	0.9976	3
						GSWFLTGVLGSQPVGGQAHMVL	2	0.9747	3
						GSWFLTGVLGSQPVGGQAHMVLVTK	2	0.9997	1
						GSWFLTGVLGSQPVGGQAHMVLVTK	3	0.9997	12
						GYTCTCSPGYEGSNCELAK	2	0.9899	1
						KLTNSEGKDFCGGVIIR	3	0.9228	1
						LGEDHKQCVPHDQCACG	2	0.9665	1
						LGEDHKQCVPHDQCACGVLTSEK	3	0.9991	3
						LGEDHKQCVPHDQCACGVLTSEK	4	0.9906	1
						LGEDHKQCVPHDQCACGVLTSEKR	3	0.9994	4
						LGEDHKQCVPHDQCACGVLTSEKR	4	0.9564	2
						LPGQESYTCSCAQGYR	2	0.9967	2
						LPVCTPEKDFAEHLLIPR	3	0.9571	3
						LQDLPWQVK	2	0.8792	1
						LTNSEGKDFCGGVIIR	2	0.9995	4
						LTNSEGKDFCGGVIIR	3	0.9995	6
						LTNSEGKDFCGGVIIRE	2	0.8778	1
						LTNSEGKDFCGGVIIRE	3	0.8527	1
						LTNSEGKDFCGGVIIREN	2	0.8768	1
						LTNSEGKDFCGGVIIRENFVLTTAK	3	0.8477	1
						NSEGKDFCGGVIIR	2	0.8803	1
						PDLQDLPWQVK	2	0.9865	2
						QCVPHDQCACGVLT	2	0.824	1
						QCVPHDQCACGVLTSEK	2	0.9997	2
						QCVPHDQCACGVLTSEK	3	0.9762	1
						QCVPHDQCACGVLTSEKR	2	0.9997	3
						QCVPHDQCACGVLTSEKR	3	0.999	4
						RAPDLQDLPWQ	2	0.986	4
						RAPDLQDLPWQV	2	0.9926	2
						RAPDLQDLPWQVK	2	0.9997	4
						RAPDLQDLPWQVK	3	0.9997	5
						SEGKDFCGGVIIR	2	0.9747	1
						SPGYEGSNCELAK	2	0.9967	1
						SSVAAMHWMDGSVVTR	2	0.9997	5
						SSVAAMHWMDGSVVTR	3	0.9997	5
						TCSPGYEGSNCELAK	2	0.9786	2
						TCTCSPGYEGSNCELAK	2	0.9954	2
						TDGCQHFCLPGQES	2	0.946	1
						TDGCQHFCLPGQESYTCSCAQGYR	3	0.9997	8
						TSQDPLMIK	2	0.9997	4
						TSQDPLMIKI	2	0.8467	1
						YDADAGENDLSL	2	0.8814	1
Q$725-1	P23284	PPIB_HUMAN	PPIB	Peptidyl-prolyl cis-trans isomerase B	1.0000	9.72	2	2	2
						IEVEKPFAIAKE	2	0.9991	1
						VLEGMEVVR	2	0.9971	1
Q$726-1	P23526	SAHH_HUMAN	AHCY	Adenosylhomocysteinase	1.0000	5.32	2	2	3
						VADIGLAAWGR	2	0.999	1
						VAVVAGYGDVGK	2	0.9974	2
Q$727-1	P23526-2	SAHH_HUMAN	AHCY	Isoform 2 of Adenosylhomocysteinase	1.0000	2.97	1	1	2
						VAVVAGYGDVGK	2	0.9974	2
Q$728-1	P24158	PRTN3_HUMAN	PRTN3	Myeloblastin	1.0000	27.73	6	8	20
Q$728-2	U3KPS2	U3KPS2_HUMAN	PRTN3	Myeloblastin	1.0000	33.02	6	8	20
						GNPGSHFCGGTLIHPSFVLTAAHCLR	3	0.9986	4
						GNPGSHFCGGTLIHPSFVLTAAHCLR	4	0.9944	2
						LVNVVLGAHNVR	2	0.9996	2
						LVNVVLGAHNVR	3	0.9853	1
						NVVLGAHNVR	2	0.8585	2
						TQEPTQQHFSVAQVFLNNYDAENK	3	0.9996	4
						VALYVDWIR	2	0.9996	4
						VVLGAHNVR	2	0.8982	1
Q$729-1	P24534	EF1B_HUMAN	EEF1B2	Elongation factor 1-beta	1.0000	12.44	2	2	4
						SIQADGLVWGSSK	2	0.9996	3
						SPAGLQVLNDYLADK	2	0.9987	1

Q$730-1	P25325	THTM_HUMAN	MPST	3-mercaptopyruvate sulfurtransferase	1.0000	13.47	3	4	9
Q$730-2	P25325-2	THTM_HUMAN	MPST	Isoform 2 of 3-mercaptopyruvate sulfurtransferase	1.0000	12.62	3	4	9
						AGQPLQLLDASWYLPK	2	0.9996	4
						ALVSAQWVAEALR	2	0.9997	2
						ALVSAQWVAEALR	3	0.9857	1
						ARPEDVISEGR	2	0.9995	2
Q$731-1	P25705	ATPA_HUMAN	ATP5A1	ATP synthase subunit alpha, mitochondrial	1.0000	32.55	12	14	25
						EAYPGDVFYLHSR	2	0.9903	1
						EIVTNFLAGFEA	2	0.9821	1
						EVAAFAQFGSDLDAATQQLLSR	2	0.9997	3
						EVAAFAQFGSDLDAATQQLLSR	3	0.998	2
						FENAFLSHVVSQHQALLGTIR	3	0.9618	1
						FENAFLSHVVSQHQALLGTIR	4	0.9442	1
						GMSLNLEPDNVGVVVFGNDK	2	0.9994	2
						HALIIYDDLSK	2	0.997	1
						ILGADTSVDLEETGR	2	0.9997	2
						NVQAEEMVEFSSGLK	2	0.9997	3
						TGAIVDVPVGEELLGR	2	0.9996	3
						TGTAEMSSILEER	2	0.9995	2
						TSIAIDTIINQK	2	0.9946	2
						VLSIGDGIAR	2	0.9989	1
Q$732-1	P25705-2	ATPA_HUMAN	ATP5A1	Isoform 2 of ATP synthase subunit alpha, mitochondrial	1.0000	8.75	3	3	6
						EAYPGDVFYLHSR	2	0.9903	1
						ILGADTSVDLEETGR	2	0.9997	2
						TGAIVDVPVGEELLGR	2	0.9996	3
Q$733-1	P25788-2	PSA3_HUMAN	PSMA3	Isoform 2 of Proteasome subunit alpha type-3	0.9931	4.84	1	1	2
Q$733-2	P25788	PSA3_HUMAN	PSMA3	Proteasome subunit alpha type-3	0.9931	4.71	1	1	2
						AVENSSTAIGIR	2	0.9931	2
Q$734-1	P25815	S100P_HUMAN	S100P	Protein S100-P	1.0000	86.32	5	7	12
						DKDAVDKLLK	2	0.9801	1
						DLDANGDAQVDFSEFIVFVAAITSACHK	3	0.9997	4
						ELPGFLQSGKDKDAVDK	2	0.9981	2
						MTELETAMGMIIDVFSR	2	0.8558	1
						MTELETAMGMIIDVFSR	3	0.9827	1
						YSGSEGSTQTLTKGELK	2	0.9995	2
						YSGSEGSTQTLTKGELK	3	0.912	1
Q$735-1	P26038	MOES_HUMAN	MSN	Moesin	1.0000	12.13	6	6	10
						APDFVFYAPR	2	0.9896	1
						EVWFFGLQYQDTK	2	0.9981	3
						GMLREDAVLEYLK	3	0.9109	1
						IAQDLEMYGVNYFSIK	2	0.9845	1
						IGFPWSEIR	2	0.9994	2
						IQVWHEEHR	2	0.9991	2
Q$736-1	P26640	SYVC_HUMAN	VARS	Valine--tRNA ligase	1.0000	10.52	10	10	13
						ADFPAGIPECGTDALR	2	0.8794	1
						CGEMAQAASAAVTR	2	0.9919	2
						EAFLQEVWK	2	0.9472	1
						GALINVPPPFLGLPR	2	0.9932	2
						IETMLGDVAVAVHPK	3	0.9738	1
						KAVLVALK	2	0.9942	1
						LHEEGIIYR	2	0.993	1
						LSAAVTEAFVR	2	0.9994	1
						SLPIVFDEFVDMDFGTGAVK	2	0.9996	2
						VQGSDSDEEVVVATTR	2	0.9993	1
Q$737-1	P27169	PON1_HUMAN	PON1	Serum paraoxonase/arylesterase 1	1.0000	61.41	23	28	89
						AEGFDFANGINISPDGK	2	0.9841	2
						DLNEEDPTVLELGITGSK	2	0.9567	1
						EVQPVELPNCNLVK	2	0.9997	4
						FDVSSFNPHGISTF	2	0.9883	2
						FDVSSFNPHGISTFTDEDNAMYLLVVNHPDAK	3	0.999	5
						FDVSSFNPHGISTFTDEDNAMYLLVVNHPDAK	4	0.9925	1
						GFDFANGINISPDGK	2	0.8638	1
						GIETGSEDLEILPNGLAFISSGLK	2	0.9997	5
						GIETGSEDLEILPNGLAFISSGLK	3	0.9996	3
						GIETGSEDLEILPNGLAFISSGLKYPGIK	3	0.9993	9
						IFFYDSENPPASEVLR	2	0.9997	9
						IFFYDSENPPASEVLR	3	0.8542	1
						ILLMDLNEEDPTVLELGITGSK	2	0.9997	4
						ILLMDLNEEDPTVLELGITGSK	3	0.9994	3
						IQNILTEEPK	2	0.9997	3
						LLIGTVFHK	2	0.9992	2
						LLVVNHPDAK	2	0.8787	1
						NEEDPTVLELGITGSK	2	0.9006	1
						REVQPVELPNCNLVK	2	0.9564	1
						SFNPNSPGK	2	0.985	1
						STVELFK	2	0.994	1
						STVELFKFQEEEK	2	0.9828	3
						TLLGMGLALFR	2	0.9894	2
						VTQVYAENGTVLQGSTVASVYK	2	0.9995	2
						VVAEGFDFANGINISPDGK	2	0.9997	13
						YVYIAELLAH	2	0.9321	2
						YVYIAELLAHK	2	0.9996	3
						YVYIAELLAHK	3	0.9995	4
Q$738-1	P27348	1433T_HUMAN	YWHAQ	14-3-3 protein theta	1.0000	9.8	2	2	4
						AVTEQGAELSNEER	2	0.9994	3
						DSTLIMQLLR	2	0.997	1
Q$739-1	P27797	CALR_HUMAN	CALR	Calreticulin	1.0000	27.34	9	12	22
						EQFLDGDGWTSR	2	0.975	2
						FYALSASFEPFSNK	2	0.9997	3
						FYALSASFEPFSNK	3	0.998	2
						FYGDEEKDKGLQTSQDAR	2	0.9996	1
						FYGDEEKDKGLQTSQDAR	3	0.9933	1
						GQTLVVQFTVK	2	0.9997	3
						HEQNIDCGGGYVK	2	0.9997	2
						KVHVIFNYK	2	0.9916	2
						KVHVIFNYK	3	0.9337	1
						QIDNPDYK	2	0.9312	1
						SGTIFDNFLITNDEAYAEEFGNETWGVTK	3	0.9995	3
						VHVIFNYK	2	0.9992	1
Q$740-1	P28066	PSA5_HUMAN	PSMA5	Proteasome subunit alpha type-5	0.9859	4.98	1	1	2
						LFQVEYAIEAIK	2	0.9859	2
Q$741-1	P28074-3	PSB5_HUMAN	PSMB5	Isoform 3 of Proteasome subunit beta type-5	0.9813	10	1	1	2
Q$741-2	P28074	PSB5_HUMAN	PSMB5	Proteasome subunit beta type-5	0.9813	6.08	1	1	2
						GYSYDLEVEQAYDLAR	2	0.9813	2
Q$742-1	P28799-3	GRN_HUMAN	GRN	Isoform 3 of Granulins	0.9998	8.23	1	1	2
						LQSGAWGCCPFTQAVCCEDHIHCCPAGFTCDTQK	4	0.9808	2
Q$743-1	P28799	GRN_HUMAN	GRN	Granulins	0.9998	8.43	2	2	3
Q$743-2	P28799-2	GRN_HUMAN	GRN	Isoform 2 of Granulins	0.9998	11.42	2	2	3
						LQSGAWGCCPFTQAVCCEDHIHCCPAGFTCDTQK	4	0.9808	2
						VHCCPHGAFCDLVHTR	4	0.8923	1
Q$744-1	P28838	AMPL_HUMAN	LAP3	Cytosol aminopeptidase	1.0000	9.63	4	4	6
Q$744-2	P28838-2	AMPL_HUMAN	LAP3	Isoform 2 of Cytosol aminopeptidase	1.0000	10.25	4	4	6
						ADMGGAATICSAIVSAAK	2	0.9107	1
						GITFDSGGISIK	2	0.9813	2
						GVLFASGQNLAR	2	0.9994	2
						TLIEFLLR	2	0.9882	1
Q$745-1	P29034	S10A2_HUMAN	S100A2	Protein S100-A2	0.9998	26.53	2	2	2
						ELPSFVGEKVDEEGLK	2	0.967	1
						YSCQEGDKFK	2	0.9934	1
Q$746-1	P29350-3	PTN6_HUMAN	PTPN6	Isoform 2 of Tyrosine-protein phosphatase non-receptor type	0.9999	2.85	2	2	2
Q$746-2	P29350-4	PTN6_HUMAN	PTPN6	Isoform 4 of Tyrosine-protein phosphatase non-receptor type	0.9999	2.72	2	2	2
Q$746-3	P29350	PTN6_HUMAN	PTPN6	Tyrosine-protein phosphatase non-receptor type 6	0.9999	2.86	2	2	2
						LEVLQSQK	2	0.9537	1

						VGDQVTHIR	2	0.9916	1
Q$747-1	P29622	KAIN_HUMAN	SERPINA4	Kallistatin	1.0000	12.41	5	5	11
						FYYLIASETPGK	2	0.9992	3
						IAPANADFAFR	2	0.909	1
						IVDLVSELK	2	0.9774	2
						LGFTDLFSK	2	0.9978	3
						VGSALFLSHNLK	2	0.9976	2
Q$748-1	P29692-3	EF1D_HUMAN	EEF1D	Isoform 3 of Elongation factor 1-delta	1.0000	21.4	5	6	11
						FYEQMNGPVAGASR	2	0.9997	2
						GVVQELQQAISK	2	0.9996	2
						GVVQELQQAISKLEAR	2	0.9948	1
						GVVQELQQAISKLEAR	3	0.9994	2
						IASLEVENQSLR	2	0.9997	2
						SIQLDGLVWGASK	2	0.9997	2
Q$749-1	P30041	PRDX6_HUMAN	PRDX6	Peroxiredoxin-6	1.0000	20.98	4	5	8
						DFTPVCTTELGR	2	0.9968	2
						FHDFLGDSWGILFSHPR	3	0.9986	3
						FHDFLGDSWGILFSHPR	4	0.9315	1
						LPFPIIDDR	2	0.9349	1
						VVFVFGPDK	2	0.9796	1
Q$750-1	P30043	BLVRB_HUMAN	BLVRB	Flavin reductase (NADPH)	1.0000	18.45	3	4	6
						HDLGHFMLR	2	0.9178	1
						HDLGHFMLR	3	0.9986	1
						NDLSPTTVMSEGAR	2	0.9991	1
						TVAGQDAVIVLLGTR	2	0.9996	3
Q$751-1	P30048-2	PRDX3_HUMAN	PRDX3	Isoform 2 of Thioredoxin-dependent peroxide reductase, mito	0.9369	5.88	1	1	2
Q$751-2	P30048	PRDX3_HUMAN	PRDX3	Thioredoxin-dependent peroxide reductase, mitochondrial	0.9369	5.47	1	1	2
						DYGVLLEGSGLALR	2	0.9369	2
Q$752-1	P30084	ECHM_HUMAN	ECHS1	Enoyl-CoA hydratase, mitochondrial	0.9840	9.66	2	2	2
						AQFAQPEILIGTIPGAGGTQR	2	0.8255	1
						IVVAMAK	2	0.9084	1
Q$753-1	P30101	PDIA3_HUMAN	PDIA3	Protein disulfide-isomerase A3	1.0000	33.66	14	15	20
						EATNPPVIQEEKPK	2	0.9995	1
						ELSDFISYLQR	2	0.9994	2
						FISDKDASIVGFFDDSFSEAHSEFLK	3	0.9975	2
						FLQDYFDGNLKR	3	0.9934	1
						FVMQEEFSR	2	0.9988	1
						IFRDGEEAGAYDGPR	3	0.9136	1
						KTFSHELSDFGLESTAGEIPVVAIR	3	0.9396	1
						LAPEYEAAATR	2	0.9985	1
						LNFAVASR	2	0.9748	1
						LSKDPNIVIAK	2	0.9879	2
						LSKDPNIVIAK	3	0.9808	1
						RLAPEYEAAATR	2	0.9578	1
						SDVLELTDDNFESR	2	0.991	2
						SEPIPESNDGPVK	2	0.9916	2
						TFSHELSDFGLESTAGEIPVVAIR	3	0.991	1
Q$754-1	P30153	2AAA_HUMAN	PPP2R1A	Serine/threonine-protein phosphatase 2A 65 kDa regulatory	1.0000	22.58	11	12	21
						ELVSDANQHVK	2	0.9947	1
						FSVCYPR	2	0.8942	1
						IGPILDNSTLQSEVKPILEK	3	0.9897	1
						LAGGDWFTSR	2	0.993	4
						LNIISNLDCVNEVIGIR	2	0.9995	2
						LNIISNLDCVNEVIGIR	3	0.8721	1
						LSTIALALGVER	2	0.9994	3
						LTQDQDVDVK	2	0.9989	3
						SEIIPMFSNLASDEQDSVR	2	0.9786	1
						TSACGLFSVCYPR	2	0.9992	2
						VKEFCENLSADCR	2	0.9033	1
						VLELDNVK	2	0.9318	1
Q$755-1	P30447	1A23_HUMAN	HLA-A	HLA class I histocompatibility antigen, A-23 alpha chain	1.0000	24.93	7	7	12
						APWIEQEGPEYWDEETGKVK	3	0.9961	3
						AYLEGTCVDGLR	2	0.9973	1
						AYLEGTCVDGLRR	2	0.8332	1
						CWALGFYPAEITLTWQR	2	0.9996	2
						FIAVGYVDDTQFVR	2	0.9996	2
						SWTAADMAAQITQR	2	0.9693	1
						WAAVVVPSGEEQR	2	0.9989	2
Q$756-1	P30520	PURA2_HUMAN	ADSS	Adenylosuccinate synthetase isozyme 2	0.9545	3.07	1	1	2
						VVDLLAQDADIVCR	2	0.9545	2
Q$757-1	P30740	ILEU_HUMAN	SERPINB1	Leukocyte elastase inhibitor	1.0000	38.52	12	14	29
						ADLSGMSGAR	2	0.9167	1
						FAYGYIEDLK	2	0.9984	3
						FKLEESYTLNSDLAR	2	0.996	2
						FQSLNADINKR	2	0.9988	3
						HNSSGSILFLGR	2	0.9871	3
						HNSSGSILFLGR	3	0.977	2
						KFAYGYIEDLK	3	0.9544	1
						KIEEQLTLEK	2	0.99	1
						LGVQDLFNSSK	2	0.9954	2
						LVLVNAIYFK	2	0.9991	1
						TFHFNTVEEVHSR	2	0.9994	4
						TFHFNTVEEVHSR	3	0.9598	1
						TYNFLPEFLVSTQK	2	0.9991	4
						VLELPYQGEELSMVILLPDDIEDESTGLK	3	0.8917	1
Q$758-1	P31431-2	SDC4_HUMAN	SDC4	Isoform 2 of Syndecan-4	1.0000	32.68	4	4	8
Q$758-2	P31431	SDC4_HUMAN	SDC4	Syndecan-4	1.0000	25.25	4	4	8
						AGSGSQVPTEPK	2	0.9939	2
						ETEVIDPQDLLEGR	2	0.9997	3
						KLEENEVIPK	2	0.9868	1
						RISPVEESEDVSNK	2	0.9996	2
Q$759-1	P31939	PUR9_HUMAN	ATIC	Bifunctional purine biosynthesis protein PURH	1.0000	37.5	16	19	35
Q$759-2	P31939-2	PUR9_HUMAN	ATIC	Isoform 2 of Bifunctional purine biosynthesis protein PURH	1.0000	37.56	16	19	35
						AFTHTAQYDEAISDYFR	3	0.9571	1
						DLIVATIAVK	2	0.9994	2
						DVSELTGFPEMLGGR	2	0.8681	1
						EVSDGIIAPGYEEEALTILSK	2	0.9994	6
						EVSDGIIAPGYEEEALTILSK	3	0.9968	2
						LDFNLIR	2	0.9996	2
						LTEVSISSDAFFPFR	2	0.9969	1
						LTEVSISSDAFFPFRDNVDR	3	0.9178	1
						MSSFGDFVALSDVCDVPTAK	2	0.9987	1
						NGQVIGIGAGQQSR	2	0.9964	1
						TLFGLHLSQK	2	0.9671	1
						TLHPAVHAGILAR	2	0.9886	1
						TLHPAVHAGILAR	3	0.9203	1
						TVASPGVTVEEAVEQIDIGGVTLLR	2	0.9996	1
						TVASPGVTVEEAVEQIDIGGVTLLR	3	0.9996	6
						VCMVYDLYK	2	0.9951	2
						VVACNLYPFVK	2	0.9995	3
						VVIEACDELGIILAHTNLR	3	0.9442	1
						YTQSNSVCYAK	2	0.9759	1
Q$760-1	P31946	1433B_HUMAN	YWHAB	14-3-3 protein beta/alpha	1.0000	22.36	4	4	7
Q$760-2	P31946-2	1433B_HUMAN	YWHAB	Isoform Short of 14-3-3 protein beta/alpha	1.0000	22.54	4	4	7
						SVFYYEILNSPEK	2	0.9529	3
						TAFDEAIAELDTLNEESYK	2	0.9864	1
						YLIPNATQPESK	2	0.9948	1
						YLSEVASGDNK	2	0.9965	2
Q$761-1	P31947	1433S_HUMAN	SFN	14-3-3 protein sigma	1.0000	10.89	2	2	4
Q$761-2	P31947-2	1433S_HUMAN	SFN	Isoform 2 of 14-3-3 protein sigma	1.0000	12.5	2	2	4
						GAVEKGEELSCEER	2	0.9987	2
						YLAEVATGDDKKR	2	0.9955	2
Q$762-1	P31949	S10AB_HUMAN	S100A11	Protein S100-A11	1.0000	25.71	2	2	6

						CIESLIAVFQK	2	0.9996	3
						TEFLSFMNTELAAFTK	2	0.9997	3
Q$763-1	P31997	CEAM8_HUMAN	CEACAM8	Carcinoembryonic antigen-related cell adhesion molecule 8	1.0000	12.32	3	3	3
						EVLLLVHNLPQDPR	3	0.9517	1
						IIGYVISNQQITPGPAYSNR	3	0.9967	1
						TLTLLSVTR	2	0.9942	1
Q$764-1	P32119	PRDX2_HUMAN	PRDX2	Peroxiredoxin-2	1.0000	27.78	7	7	11
						EGGLGPLNIPLLADVTR	2	0.9993	2
						GLFIIDGK	2	0.9969	1
						KEGGLGPLNIPLLADVTR	3	0.9981	2
						QITVNDLPVGR	2	0.9982	2
						RLSEDYGVLK	2	0.9369	1
						RLSEDYGVLKTDEGIAYR	3	0.9782	2
						TDEGIAYR	2	0.9902	1
Q$765-1	P33176	KINH_HUMAN	KIF5B	Kinesin-1 heavy chain	0.9981	3.63	3	3	3
						FRPLNESEVNR	2	0.9335	1
						NTIQWLENELNR	2	0.864	1
						TGAEGAVLDEAK	2	0.9862	1
Q$766-1	P33908	MA1A1_HUMAN	MAN1A1	Mannosyl-oligosaccharide 1,2-alpha-mannosidase IA	1.0000	27.41	17	21	44
						AYYLSGEEIFR	2	0.9765	3
						DILLEKK	2	0.9439	1
						DVYLLHESYDDVQQSFFLAETLK	3	0.9993	3
						FDGGVEAIATR	2	0.9997	6
						FVGGLLSAYYLSGEEIFR	2	0.9997	5
						FVGGLLSAYYLSGEEIFR	3	0.9994	4
						GATIVDALDTLFIMEMK	2	0.9996	2
						GGHSSSLFGNIK	2	0.9984	1
						GGHSSSLFGNIK	3	0.9824	1
						GLPPVDFVPPIGVESR	2	0.8729	1
						HAWNNYK	2	0.9602	1
						KMYFDAVQAIETHLIR	3	0.9996	2
						KMYFDAVQAIETHLIR	4	0.9431	1
						KSSSGLTYIAEWK	2	0.9996	2
						KWAWEAVEALENHCR	3	0.985	1
						LGPEAFR	2	0.9927	1
						MYFDAVQAIETHLIR	3	0.9995	1
						SSSGLTYIAEWK	2	0.9993	3
						WAWEAVEALENHCR	2	0.9925	2
						WAWEAVEALENHCR	3	0.9242	1
						YYILRPEVMETYMYMWR	3	0.9983	2
Q$767-1	P34932	HSP74_HUMAN	HSPA4	Heat shock 70 kDa protein 4	1.0000	3.33	2	2	4
						AGGIETIANEYSDR	2	0.9664	2
						GCALQCAILSPAFK	2	0.9991	2
Q$768-1	P35232	PHB_HUMAN	PHB	Prohibitin	0.9999	8.82	2	2	3
						FDAGELITQR	2	0.9931	1
						KLEAAEDIAYQLSR	3	0.9934	2
Q$769-1	P35442	TSP2_HUMAN	THBS2	Thrombospondin-2	1.0000	1.71	1	1	1
						DGQGDACDPDDDNDGVPDDR	3	0.9658	1
Q$770-1	P35527	K1C9_HUMAN	KRT9	Keratin, type I cytoskeletal 9	1.0000	83.47	37	48	112
						DIENQYETQITQIEHEVSSSGQEVQSSAK	3	0.9996	8
						DIENQYETQITQIEHEVSSSGQEVQSSAK	4	0.9817	2
						EIETYHNLLEGGQEDFESSGAGK	2	0.9997	3
						EIETYHNLLEGGQEDFESSGAGK	3	0.9996	4
						FSSSSGYGGGSSR	2	0.9997	4
						GFGGASGGGYSSSGGFGGGFGGGSGGGFGGGYGSGFGGFGGFGGGAGGGDGGILTANEK	4	0.9977	2
						GGGGGGGLGSGGSIR	2	0.8788	1
						GGGGSFGYSYGGGSGGGFSASSLGGGFGGGSR	3	0.9995	4
						GGSGGSHGGGSGFGGESGGSYGGGEEASGSGGGYGGGSGK	3	0.9996	2
						GGSGGSHGGGSGFGGESGGSYGGGEEASGSGGGYGGGSGKS	3	0.9262	1
						GGSGGSYGGGGSGGGYGGGSGSR	2	0.9997	4
						GGSGGSYGGGSGSGGGSGGGYGGGSGGGHSGGSGGGHSGGSGGNYGGGSGSG	3	0.8009	1
						HGVQELEIELQSQLSK	2	0.9997	3
						HGVQELEIELQSQLSKK	2	0.9996	2
						HGVQELEIELQSQLSKK	3	0.9997	3
						IKFEMEQNLR	2	0.9571	1
						IKFEMEQNLR	3	0.9963	1
						IQDWYDKK	2	0.993	1
						IQDWYDKKGPAAIQK	2	0.9985	1
						IQDWYDKKGPAAIQK	3	0.9893	1
						LASYLDKVQALEEANNDLENK	2	0.9996	3
						LASYLDKVQALEEANNDLENK	3	0.9992	3
						LEKEIETYHNLLEGGQEDFESSGAGK	3	0.9994	3
						MRLEKEIETYHNLLEGGQEDFESSGAGK	3	0.9171	1
						MTLDDFR	2	0.9993	2
						NHKEEMSQLTGQNSGDVNVEINVAPGK	3	0.9825	1
						NHKEEMSQLTGQNSGDVNVEINVAPGKDLTK	3	0.9942	1
						NHKEEMSQLTGQNSGDVNVEINVAPGKDLTK	4	0.9921	1
						NLLEGGQEDFESSGAGK	2	0.9885	2
						NYSPYYNTIDDLKDQIVDLTVGNNK	3	0.9995	4
						NYSPYYNTIDDLKDQIVDLTVGNNK	4	0.8793	1
						NYSPYYNTIDDLKDQIVDLTVGNNKTLLDIDNTR	3	0.9946	1
						QEIECQNQEYSLLLSIK	2	0.9996	4
						QEIECQNQEYSLLLSIK	3	0.9954	2
						QEYEQLIAK	2	0.9865	1
						QVLDNLTMEK	2	0.9977	1
						QVLDNLTMEKSDLEMQYETLQEELMALK	3	0.9981	4
						QVLDNLTMEKSDLEMQYETLQEELMALKK	3	0.9759	2
						SDLEMQYETLQEELMALK	2	0.9997	3
						SDLEMQYETLQEELMALK	3	0.9889	2
						SDLEMQYETLQEELMALKK	2	0.9993	2
						SDLEMQYETLQEELMALKK	3	0.9944	3
						STMQELNSR	2	0.9996	6
						TLLDIDNTR	2	0.9995	2
						TLNDMRQEYEQLIAK	2	0.9869	2
						VQALEEANNDLENK	2	0.9997	2
						YCGQLQMIQEQISNLEAQITDVR	3	0.9996	3
						YCGQLQMIQEQISNLEAQITDVRQEIECQNQEYSLLLSIK	4	0.9524	1
Q$771-1	P35555	FBN1_HUMAN	FBN1	Fibrillin-1	0.9838	0.45	1	1	2
						YEDEECTLPIAGR	2	0.9838	2
Q$772-1	P35579	MYH9_HUMAN	MYH9	Myosin-9	1.0000	46.89	93	117	233
						ADEWLMK	2	0.9937	1
						ADFCIIHYAGK	2	0.9922	1
						ADFCIIHYAGK	3	0.9175	1
						AGVLAHLEEER	2	0.9993	1
						AKQTLENERGELANEVK	2	0.9964	1
						ALEEAMEQKAELER	2	0.9842	1
						ALELDSNLYR	2	0.9997	2
						ALEQQVEEMK	2	0.9995	1
						ALEQQVEEMKTQLEELEDELQATEDAK	3	0.9993	4
						ANLQIDQINTDLNLER	2	0.9997	2
						ANLQIDQINTDLNLER	3	0.9955	2
						ASITALEAK	2	0.9992	1
						ASREEILAQAK	2	0.9108	1
						ASREEILAQAK	3	0.8482	1
						ASREEILAQAKENEK	2	0.9972	1
						ASREEILAQAKENEK	3	0.9966	2
						DELADEIANSSGK	2	0.9995	1
						DFSALESQLQDTQELLQEENR	2	0.9998	2
						DFSALESQLQDTQELLQEENR	3	0.9997	2
						DFSALESQLQDTQELLQEENRQK	3	0.9981	3
						DKADFCIIHYAGK	3	0.8934	2
						DLEAHIDSANK	2	0.9997	2
						DLEGLSQR	2	0.9866	1

						DLEGLSQRHEEK	2	0.9914	2
						EEILAQAKENEK	2	0.9962	1
						ELEDATETADAMNR	2	0.9995	1
						ELESQISELQEDLESER	2	0.9997	4
						ELESQISELQEDLESER	3	0.9987	2
						EMEAELEDERK	2	0.9996	2
						EQLEEEEEAKHNLEK	2	0.9995	2
						FDQLLAEEK	2	0.9825	1
						FVSELWK	2	0.9358	1
						GALALEEK	2	0.9927	1
						GALALEEKR	2	0.9992	1
						HSQAVEELAEQLEQTK	2	0.9991	1
						HSQAVEELAEQLEQTK	3	0.9853	2
						HSQAVEELAEQLEQTKR	3	0.9995	3
						IAEFTTNLTEEEEK	2	0.9996	2
						IAEFTTNLTEEEEKSK	2	0.9995	3
						IAQLEEELEEEQGNTELINDR	2	0.9997	3
						IAQLEEELEEEQGNTELINDR	3	0.9991	3
						IAQLEEELEEEQGNTELINDRLK	3	0.9971	3
						IAQLEEQLDNETK	2	0.9997	1
						IAQLEEQLDNETKER	3	0.9228	1
						IIGLDQVAGMSETALPGAFK	2	0.9992	2
						IMGIPEEEQMGLLR	2	0.9737	2
						IRELESQISELQEDLESER	2	0.9986	2
						IRELESQISELQEDLESER	3	0.9995	4
						ITDVIIGFQACCR	2	0.9957	2
						KANLQIDQINTDLNLER	2	0.9996	1
						KANLQIDQINTDLNLER	3	0.9951	2
						KEEELQAALAR	2	0.9991	2
						KEEELQAALAR	3	0.996	1
						KEEELQAALARVEEEAAQK	3	0.8121	1
						KFDQLLAEEK	2	0.9778	1
						KKVEAQLQELQVK	2	0.9991	2
						KKVEAQLQELQVK	3	0.8102	1
						KLEEEQIILEDQNCK	2	0.9997	3
						KLEEEQIILEDQNCK	3	0.9768	1
						KLEGDSTDLSDQIAELQAQIAELK	3	0.9996	8
						KMEDSVGCLETAEEVKR	3	0.9961	2
						KQELEEICHDLEAR	3	0.9993	2
						KVEAQLQELQVK	2	0.9997	2
						KVEAQLQELQVK	3	0.9982	2
						KVIQYLAYVASSHK	3	0.9994	3
						LDPHLVLDQLR	2	0.9489	1
						LDPHLVLDQLR	3	0.9995	2
						LEGDSTDLSDQIAELQAQIAELK	3	0.8805	1
						LEVNLQAMK	2	0.9988	1
						LQLQEQLQAETELCAEAEELR	3	0.9986	3
						LQQELDDLLVDLDHQR	2	0.9997	4
						LQQELDDLLVDLDHQR	3	0.9994	3
						LQQLFNHTMFILEQEEYQR	3	0.9995	3
						LQVELDNVTGLLSQSDSK	2	0.9997	6
						LQVELDNVTGLLSQSDSK	3	0.9991	2
						LRLEVNLQAMK	3	0.9338	1
						LTEMETLQSQLMAEK	2	0.9996	3
						LTKDFSALESQLQDTQELLQEENR	3	0.9942	4
						MEDSVGCLETAEEVKR	3	0.9218	1
						NFINNPLAQADWAAK	2	0.9996	2
						NKHEAMITDLEER	3	0.9936	1
						NLPIYSEEIVEMYK	2	0.9989	2
						QAQQERDELADEIANSSGK	2	0.9992	3
						QAQQERDELADEIANSSGK	3	0.9965	1
						QIATLHAQVADMK	2	0.9994	2
						QIATLHAQVADMKK	2	0.9015	1
						QLEEAEEEAQR	2	0.9992	2
						QLLQANPILEAFGNAK	2	0.9978	3
						QTLENERGELANEVK	2	0.9888	3
						RKLEGDSTDLSDQIAELQAQIAELK	3	0.9957	2
						RKLEGDSTDLSDQIAELQAQIAELK	4	0.9901	1
						SGFEPASLKEEVGEEAIVELVENGKK	3	0.9769	2
						SMEAEMIQLQEELAAAER	2	0.9997	5
						TDLLLEPYNK	2	0.9993	2
						TFHIFYYLLSGAGEHLK	3	0.9996	3
						TFHIFYYLLSGAGEHLK	4	0.998	4
						THEAQIQEMR	2	0.9996	2
						TLEEEAKTHEAQIQEMR	3	0.9873	1
						TQLEELEDELQATEDAK	2	0.9997	3
						TRLQQELDDLLVDLDHQR	3	0.9993	4
						TVGQLYKEQLAK	2	0.9748	1
						VAAYDKLEK	2	0.988	1
						VEAQLQELQVK	2	0.9995	1
						VEDMAELTCLNEASVLHNLK	3	0.9921	2
						VIQYLAYVASSHK	2	0.9997	3
						VIQYLAYVASSHK	3	0.9996	2
						VISGVLQLGNIVFK	2	0.9997	3
						VISGVLQLGNIVFKK	2	0.9984	1
						VISGVLQLGNIVFKK	3	0.9837	1
						VRTELADKVTK	2	0.9789	1
						VRTELADKVTK	3	0.9992	1
						VSHLLGINVTDFTR	2	0.9996	3
						VSHLLGINVTDFTR	3	0.9987	1
						VVFQEFR	2	0.9994	2
						YEILTPNSIPK	2	0.9978	2
						YKASITALEAK	2	0.9948	1
						YLYVDKNFINNPLAQADWAAK	3	0.9941	1
Q$773-1	P35579-2	MYH9_HUMAN	MYH9	Isoform 2 of Myosin-9	1.0000	2.03	3	3	5
						AGVLAHLEEER	2	0.9993	1
						ALELDSNLYR	2	0.9997	2
						VVFQEFR	2	0.9994	2
Q$774-1	P35716	SOX11_HUMAN	SOX11	Transcription factor SOX-11	0.9321	4.08	1	1	1
						SAAGGGGGSAGGGAGGAK	2	0.915	1
Q$775-1	P35858	ALS_HUMAN	IGFALS	Insulin-like growth factor-binding protein complex acid labile	1.0000	8.6	3	3	3
Q$775-2	P35858-2	ALS_HUMAN	IGFALS	Isoform 2 of Insulin-like growth factor-binding protein compl	1.0000	8.09	3	3	3
						LFQGLGKLEYLLLSR	3	0.9015	1
						LWLEGNPWDCGCPLK	2	0.9982	1
						SFEGLGQLEVLTLDHNQLQEVK	3	0.8752	1
Q$776-1	P35908	K22E_HUMAN	KRT2	Keratin, type II cytoskeletal 2 epidermal	1.0000	46.17	26	28	57
						AQYEEIAQR	2	0.9986	1
						DVDNAYMIK	2	0.9739	2
						DYQELMNVK	2	0.9871	2
						FLEQQNQVLQTK	2	0.9997	3
						GFSSGSAVVSGGSR	2	0.9993	2
						GGGFGGGSSFGGGSGFSGGGFGGGGFGGGR	3	0.9351	1
						HGGGGGGFGGGGFGSR	2	0.9989	3
						HGGGGGGFGGGGFGSR	3	0.9984	2
						LALDVEIATYR	2	0.9976	1
						LEQQNQVLQTK	2	0.9924	1
						LNDLEEALQQAK	2	0.999	2
						LQGEIAHVK	2	0.9954	2
						NKLNDLEEALQQAK	2	0.9901	3
						NKLNDLEEALQQAK	3	0.9029	2
						NKLNDLEEALQQAKEDLAR	3	0.9979	3
						NLDLDSIIAEVK	2	0.9996	2
						NVQDAIADAEQR	2	0.9995	3

						SISISVAGGGGGFGAAGGFGGR	2	0.9981	1
						SISVAGGGGGFGAAGGFGGR	2	0.9912	2
						SKEEAEALYHSKYEELQVTVGR	3	0.9989	2
						SVAGGGGGFGAAGGFGGR	2	0.9821	1
						TAAENDFVTLK	2	0.9158	1
						TAAENDFVTLKK	2	0.9961	2
						VDLLNQEIEFLK	2	0.9997	4
						VLYDAEISQIHQSVTDTNVILSMDNSR	3	0.999	3
						WELLQQMNVGTRPINLEPIFQGYIDSLKR	4	0.9963	3
						YEELQVTVGR	2	0.9985	2
						YLDGLTAER	2	0.9949	1
Q$777-1	P36955	PEDF_HUMAN	SERPINF1	Pigment epithelium-derived factor	1.0000	29.67	11	13	24
						ALYYDLISSPDIHGTYK	2	0.9989	1
						ALYYDLISSPDIHGTYK	3	0.9759	1
						DTDTGALLFIGK	2	0.9996	3
						ELLDTVTAPQK	2	0.9482	1
						KTSLEDFYLDEER	2	0.9995	2
						KTSLEDFYLDEER	3	0.9981	2
						LAAAVSNFGYDLYR	2	0.9996	3
						LQSLFDSPDFSK	2	0.9977	2
						LSYEGEVTK	2	0.9877	1
						SSFVAPLEK	2	0.9844	1
						TESIIHR	2	0.9985	1
						TVQAVLTVPK	2	0.9978	2
						YGLDSDLSCK	2	0.9984	4
Q$778-1	P39023	RL3_HUMAN	RPL3	60S ribosomal protein L3	0.9999	9.68	3	3	4
						AGMTHIVR	2	0.997	1
						NNASTDYDLSDK	2	0.8779	1
						SINPLGGFVHYGEVTNDFV	2	0.978	2
Q$779-1	P39059	COFA1_HUMAN	COL15A1	Collagen alpha-1(XV) chain	1.0000	8.14	10	11	20
						DFAISVVVKPSSTR	2	0.9994	3
						DFAISVVVKPSSTR	3	0.9384	1
						FTGSLQQLTVHPD	2	0.9931	3
						FTGSLQQLTVHPDPR	2	0.9974	3
						GGVLFAITDAFQK	2	0.9995	3
						IILYYTEPGSHVS	2	0.9586	2
						IILYYTEPGSHVSQEAAAF	2	0.9065	1
						SSQALAFESSAGIFMGNAGATGLER	3	0.9568	1
						TLIPSTFFR	2	0.9979	1
						TLLVNCEEHSR	2	0.9892	1
						VIYLGLR	2	0.9952	1
Q$780-1	P39060	COIA1_HUMAN	COL18A1	Collagen alpha-1(XVIII) chain	1.0000	4.39	5	6	9
Q$780-2	P39060-1	COIA1_HUMAN	COL18A1	Isoform 2 of Collagen alpha-1(XVIII) chain	1.0000	5.07	5	6	9
Q$780-3	P39060-2	COIA1_HUMAN	COL18A1	Isoform 3 of Collagen alpha-1(XVIII) chain	1.0000	5.75	5	6	9
						ELLREETGAALKPR	3	0.9795	2
						LTESYCETWR	2	0.993	1
						QAMLGQVHEVPEGWLIFVAEQEELYVR	3	0.9986	3
						TEAPSATGQASSLLGGR	2	0.9996	1
						YHFPSLFFR	2	0.9039	1
						YHFPSLFFR	3	0.9887	1
Q$781-1	P39656	OST48_HUMAN	DDOST	Dolichyl-diphosphooligosaccharide--protein glycosyltransfera	1.0000	3.95	2	2	3
Q$781-2	U3KQ84	U3KQ84_HUMAN	DDOST	Dolichyl-diphosphooligosaccharide--protein glycosyltransfera	1.0000	11.69	2	2	3
						GFELTFK	2	0.9969	1
						TLVLLDNLNVR	2	0.9994	2
Q$782-1	P40199	CEAM6_HUMAN	CEACAM6	Carcinoembryonic antigen-related cell adhesion molecule 6	1.0000	17.44	3	3	4
						EVLLLAHNLPQNR	2	0.9993	2
						SDPVTLNVLYGPDGPTISPSK	2	0.9995	1
						VDGNSLIVGYVIGTQQATPGPAYSGR	3	0.9522	1
Q$783-1	P40227	TCPZ_HUMAN	CCT6A	T-complex protein 1 subunit zeta	1.0000	33.52	11	11	18
						AQAALAVNISAAR	2	0.9994	2
						AQLGVQAFADALLIIPK	2	0.9996	3
						GIDPFSLDALSK	2	0.9945	1
						HKSETDTSLIR	2	0.9768	1
						IITEGFEAAKEK	2	0.9965	2
						KQDEPIDLFMIEIMEMK	3	0.9991	2
						QADLYISEGLHPR	3	0.9927	1
						QLLHSCTVIATNILLVDEIMR	3	0.9724	1
						VATAQDDITGDGTTSNVLIIGELLK	3	0.9081	1
						VHAELADVLTEAVVDSILAIK	3	0.9967	1
						VLAQNSGFDLQETLVK	2	0.9997	3
Q$784-1	P40227-2	TCPZ_HUMAN	CCT6A	Isoform 2 of T-complex protein 1 subunit zeta	1.0000	26.54	8	8	15
						AQAALAVNISAAR	2	0.9994	2
						AQLGVQAFADALLIIPK	2	0.9996	3
						GIDPFSLDALSK	2	0.9945	1
						IITEGFEAAKEK	2	0.9965	2
						KQDEPIDLFMIEIMEMK	3	0.9991	2
						QLLHSCTVIATNILLVDEIMR	3	0.9724	1
						VHAELADVLTEAVVDSILAIK	3	0.9967	1
						VLAQNSGFDLQETLVK	2	0.9997	3
Q$785-1	P40939	ECHA_HUMAN	HADHA	Trifunctional enzyme subunit alpha, mitochondrial	0.9402	1.97	1	1	1
						ADMVIEAVFEDLSLK	2	0.9402	1
Q$786-1	P41218	MNDA_HUMAN	MNDA	Myeloid cell nuclear differentiation antigen	0.9999	5.65	2	2	4
						SLLAYDLGLTTK	2	0.9988	2
						VITISDYSECK	2	0.9636	2
Q$787-1	P41226	UBA7_HUMAN	UBA7	Ubiquitin-like modifier-activating enzyme 7	0.9598	1.48	1	1	1
						VLVSGLQGLGAEVAK	2	0.9598	1
Q$788-1	P41252	SYIC_HUMAN	IARS	Isoleucine--tRNA ligase, cytoplasmic	1.0000	2.77	3	3	4
						GSELEITLTR	2	0.9818	1
						ILEFWTEFNCFQECLK	2	0.9978	2
						YIIEELNVR	2	0.9564	1
Q$789-1	P42224	STAT1_HUMAN	STAT1	Signal transducer and activator of transcription 1-alpha/beta	1.0000	4.8	3	3	4
						EGAITFTWVER	2	0.9973	1
						NLSFFLTPPCAR	2	0.9208	1
						TELISVSEVHPSR	2	0.9995	2
Q$790-1	P42330	AK1C3_HUMAN	AKR1C3	Aldo-keto reductase family 1 member C3	1.0000	33.13	8	10	22
Q$790-2	S4R3Z2	S4R3Z2_HUMAN	AKR1C3	Aldo-keto reductase family 1 member C3	1.0000	35.67	8	10	22
						HIDSAHLYNNEEQVGLAIR	2	0.9997	3
						HIDSAHLYNNEEQVGLAIR	3	0.9996	5
						HIDSAHLYNNEEQVGLAIR	4	0.9975	1
						LAIEAGFR	2	0.9995	2
						NLHYFNSDSFASHPNYPYSDEY	3	0.829	1
						QNVQVFEFQLTAEDMK	2	0.9996	3
						SKDIVLVAYSALGSQR	3	0.9801	2
						TPALIALR	2	0.9995	2
						TPALIALRY	2	0.9522	1
						VIFDIVDLCTTWEAMEK	2	0.9996	2
Q$791-1	P43490	NAMPT_HUMAN	NAMPT	Nicotinamide phosphoribosyltransferase	1.0000	10.79	4	4	7
Q$791-2	Q5SYT8	Q5SYT8_HUMAN	NAMPTL	Protein NAMPTL (Fragment)	1.0000	11.23	4	4	7
						NAQLNIELEAAHH	2	0.9995	2
						STQAPLIIRPDSGNPLDTVLK	3	0.9422	2
						SYSFDEIRK	2	0.9969	2
						YDGHLPIEIK	3	0.9116	1
Q$792-1	Q13185	CBX3_HUMAN	CBX3	Chromobox protein homolog 3	0.9759	6.56	1	1	3
Q$792-2	P45973	CBX5_HUMAN	CBX5	Chromobox protein homolog 5	0.9759	6.28	1	1	3
						CPQIVIAFYEER	2	0.9759	3
Q$793-1	P45974-2	UBP5_HUMAN	USP5	Isoform Short of Ubiquitin carboxyl-terminal hydrolase 5	0.9384	3.11	1	1	1
Q$793-2	P45974	UBP5_HUMAN	USP5	Ubiquitin carboxyl-terminal hydrolase 5	0.9384	3.03	1	1	1
						IGEWELIQESGVPLKPLFGPGYTGIR	3	0.9384	1
Q$794-1	P46060	RAGP1_HUMAN	RANGAP1	Ran GTPase-activating protein 1	1.0000	8.69	4	4	4
						NRLENDGATALAEAFR	3	0.8539	1
						QVEVINFGDCLVR	2	0.9871	1
						SSACFTLQELK	2	0.9947	1
						TQVAGGQLSFK	2	0.9013	1

Q$795-1	P46734	MP2K3_HUMAN	MAP2K3	Dual specificity mitogen-activated protein kinase kinase 3	0.9996	8.93	2	2	2
Q$795-2	P46734-2	MP2K3_HUMAN	MAP2K3	Isoform 1 of Dual specificity mitogen-activated protein kinase	0.9996	9.75	2	2	2
Q$795-3	P46734-3	MP2K3_HUMAN	MAP2K3	Isoform 2 of Dual specificity mitogen-activated protein kinase	0.9996	8.81	2	2	2
						FSPEFVDFTAQCLR	2	0.9872	1
						NFEVEADDLVTISELGR	2	0.9661	1
Q$796-1	P46777	RL5_HUMAN	RPL5	60S ribosomal protein L5	0.9912	3.37	1	1	2
Q$796-2	Q5T7N0	Q5T7N0_HUMAN	RPL5	60S ribosomal protein L5 (Fragment)	0.9912	7.69	1	1	2
						DIICQIAYAR	2	0.9912	2
Q$797-1	P46940	IQGA1_HUMAN	IQGAP1	Ras GTPase-activating-like protein IQGAP1	1.0000	17.86	20	22	34
						ALESGDVNTVWK	2	0.9991	2
						ALQSPALGLR	2	0.999	2
						ATFYGEQVDYYK	2	0.9219	1
						EEIQSSISGVTAAYNR	2	0.9997	2
						EQLWLANEGLITR	2	0.9995	3
						FALGIFAINEAVESGDVGK	2	0.9997	3
						FALGIFAINEAVESGDVGK	3	0.9635	1
						FQPGETLTEILETPATSEQEAEHQR	3	0.9748	1
						GLQQQNSDWYLK	2	0.9966	1
						GVLLEIEDLQVNQFK	2	0.9993	1
						IGGILANELSVDEAALHAAVIAINEAIDR	3	0.9903	1
						IIGNLLYYR	2	0.8261	1
						ILAIGLINEALDEGDAQK	2	0.9988	1
						ILAIGLINEALDEGDAQK	3	0.9994	3
						LAAVALINAAIQK	2	0.9993	1
						LEGVLAEVAQHYQDTLIR	3	0.9975	2
						LGNFFSPK	2	0.9715	1
						NVIFEISPTEEVGDFEVK	2	0.9989	2
						RLAAVALINAAIQK	3	0.9993	2
						RLIVDVIR	2	0.996	1
						SNQQLENDLNLMDIK	2	0.9996	1
						TVLELMNPEAQLPQVYPFAADLYQK	3	0.8559	1
Q$798-1	P48147	PPCE_HUMAN	PREP	Prolyl endopeptidase	1.0000	3.38	2	2	3
						SASGSDWVTIK	2	0.971	1
						VLYVQDSLEGEAR	2	0.9994	2
Q$799-1	P48506	GSH1_HUMAN	GCLC	Glutamate--cysteine ligase catalytic subunit	0.9579	1.41	1	1	1
						GYVSDIDCR	2	0.9579	1
Q$800-1	P48507	GSH0_HUMAN	GCLM	Glutamate--cysteine ligase regulatory subunit	0.9789	4.74	1	1	2
						LFIVESNSSSSTR	2	0.9789	2
Q$801-1	P48668	K2C6C_HUMAN	KRT6C	Keratin, type II cytoskeletal 6C	1.0000	40.96	21	22	40
						ADTLTDEINFLR	2	0.9995	3
						AIGGGLSSVGGGSSTIK	2	0.9995	2
						ALYDAELSQMQTHISDTSVVLSMDNNR	3	0.9992	3
						AQYEEIAQR	2	0.9986	1
						DVDAAYMNKVELQAK	2	0.9988	2
						EYQELMNVK	2	0.9948	1
						FLEQQNKVLDTK	2	0.9015	1
						GSGGLGGACGGAGFGSR	2	0.9984	2
						ISIGGGSCAISGGYGSR	2	0.9994	2
						LLKEYQELMNVK	2	0.9981	3
						NKLEGLEDALQK	2	0.9622	1
						NMQDLVEDLK	2	0.9049	2
						QNLEPLFEQYINNLR	2	0.9977	3
						RQLDSIVGER	2	0.9846	1
						SRAEAESWYQTK	2	0.9945	2
						SRAEAESWYQTKYEELQVTAGR	3	0.9983	2
						SRGSGGLGGACGGAGFGSR	2	0.9981	2
						SRGSGGLGGACGGAGFGSR	3	0.8482	2
						TAAENEFVTLK	2	0.9811	1
						TAAENEFVTLKK	2	0.9973	1
						WTLLQEQGTK	2	0.9985	1
						YEELQVTAGR	2	0.9991	2
Q$802-1	P48741	HSP77_HUMAN	HSPA7	Putative heat shock 70 kDa protein 7	1.0000	14.17	4	4	7
						ARFEELCSDLFR	2	0.9883	1
						ATAGDTHLGGEDFDNR	2	0.9976	1
						TTPSYVAFTDTER	2	0.9997	3
						VEIIANDQGNR	2	0.9988	2
Q$803-1	P49257	LMAN1_HUMAN	LMAN1	Protein ERGIC-53	0.9052	2.35	1	1	1
						YVSSLTEEISKR	2	0.9052	1
Q$804-1	P49327	FAS_HUMAN	FASN	Fatty acid synthase	1.0000	32.5	58	62	112
						AALQEELQLCK	2	0.9994	3
						ACLDTAVENMPSLK	2	0.9986	1
						ALGLGVEQLPVVFEDVVLHQATILPK	3	0.994	1
						CVLLSNLSSTSHVPEVDPGSAELQK	3	0.9972	2
						DLVEAVAHILGIR	2	0.9997	1
						DNLEFFLAGIGR	2	0.9958	2
						DPSQQELPR	2	0.9992	1
						DTVTISGPQAPVFEFVEQLRK	3	0.8791	1
						EACPELDYFVVFSSVSCGR	2	0.9997	8
						EAHLPPGAMAAVGLSWEECK	3	0.9243	1
						EDGLAQQQTQLNLR	2	0.9979	1
						EGGFLLLHTLLR	3	0.8969	1
						ELNLVLSVR	2	0.9979	2
						EVWALVQAGIR	2	0.9931	1
						FDASFFGVHPK	3	0.9983	1
						GLVQALQTK	2	0.9997	1
						GVDLVLNSLAEEK	2	0.9996	2
						GYAVLGGER	2	0.9749	1
						GYDYGPHFQGILEASLEGDSGR	3	0.9995	4
						HAQPTCPGAQLCTVYYASLNFR	3	0.9977	3
						HFLLEEDKPEEPTAHAFVSTLTR	4	0.9926	1
						HGLYLPTR	2	0.9923	1
						IPGLLSPHPLL	2	0.8947	2
						IPGLLSPHPLLQL	2	0.9972	3
						LFDHPESPTPNPTEPLFLAQAEVYK	3	0.9992	5
						LGMLSPEGTCK	2	0.9774	1
						LLEQGLR	2	0.9563	1
						LLLEVTYEAIVDGGINPDSLR	3	0.9996	1
						LNSVQSSERPLFLVHPIEGSTTVFHSLASR	4	0.9945	2
						LPEDPLLSGLLDSPALK	2	0.9997	4
						LQVVDQPLPVR	2	0.9996	2
						LSFFFDFR	2	0.9997	5
						LSIPTYGLQCTR	2	0.9555	1
						MASCLEVLDLFLNQPHMVLSSFVLAEK	3	0.9933	1
						QEPLLIGSTK	2	0.967	1
						QVQPEGPYR	2	0.9983	1
						RQQEQQVPILEK	2	0.9969	3
						SDEAVKPFGLK	2	0.9971	1
						SEGVVAVLLTK	2	0.9991	2
						SEGVVAVLLTKK	2	0.9994	1
						SLLVNPEGPTLMR	2	0.9989	1
						SLYQSAGVAPESFEYIEAHGTGTK	2	0.9948	1
						SLYQSAGVAPESFEYIEAHGTGTK	3	0.9798	1
						TGGAYGEDLGADYNLSQVCDGK	2	0.9997	2
						TGTVSLEVR	2	0.9956	1
						TLLEGSGLESIISIIHSSLAEPR	2	0.9996	1
						TLLEGSGLESIISIIHSSLAEPR	3	0.9996	3
						VAAAVDLIIK	2	0.9996	3
						VFTTVGSAEKR	2	0.9987	2
						VGDPQELNGITR	2	0.9979	1
						VLEALLPLK	2	0.9995	1
						VLEALLPLKGLEER	2	0.9972	1
						VLEALLPLKGLEER	3	0.9595	1
						VLFPATGYLSIVWK	2	0.9995	2

						VLQGDLVMNVYR	2	0.9993	1
						VSVHVIEGDHR	2	0.9997	2
						VSVHVIEGDHR	3	0.9994	2
						VTVAGGVHISGLHTESAPR	3	0.985	1
						VVEVLAGHGHLYSR	2	0.9996	2
						VVVQVLAEEPEAVLK	2	0.9995	3
						WTSQDSLLGMEFSGR	2	0.8435	1
						YSGTLNLDR	2	0.9942	2
Q$805-1	P49902	5NTC_HUMAN	NT5C2	Cytosolic purine 5'-nucleotidase	1.0000	4.99	2	2	4
Q$805-2	Q5JUV4	Q5JUV4_HUMAN	NT5C2	Cytosolic purine 5'-nucleotidase (Fragment)	1.0000	10.57	2	2	4
Q$805-3	P49902-2	5NTC_HUMAN	NT5C2	Isoform 2 of Cytosolic purine 5'-nucleotidase	1.0000	5.26	2	2	4
						GLVFDTLYGNLLK	2	0.9997	3
						SPEYESLGFELTVER	2	0.9997	1
Q$806-1	P50990-2	TCPQ_HUMAN	CCT8	Isoform 2 of T-complex protein 1 subunit theta	0.9706	1.89	1	1	1
Q$806-2	P50990-3	TCPQ_HUMAN	CCT8	Isoform 3 of T-complex protein 1 subunit theta	0.9706	2.11	1	1	1
Q$806-3	P50990	TCPQ_HUMAN	CCT8	T-complex protein 1 subunit theta	0.9706	1.82	1	1	1
						FAEAFEAIPR	2	0.9706	1
Q$807-1	P50995	ANX11_HUMAN	ANXA11	Annexin A11	0.9997	3.17	1	1	3
Q$807-2	P50995-2	ANX11_HUMAN	ANXA11	Isoform 2 of Annexin A11	0.9997	3.39	1	1	3
						GVGTDEACLIEILASR	2	0.9988	3
Q$808-1	P51654	GPC3_HUMAN	GPC3	Glypican-3	1.0000	8.28	3	3	5
Q$808-2	P51654-3	GPC3_HUMAN	GPC3	Isoform 3 of Glypican-3	1.0000	7.96	3	3	5
						EYILSLEELVNGMYR	2	0.9741	1
						LNMEQLLQSASMELK	2	0.9994	2
						WVPETPVPGSDLQVCLPK	2	0.9985	2
Q$809-1	P52790	HXK3_HUMAN	HK3	Hexokinase-3	0.9132	1.63	1	1	1
						AAQLCGAGVAAVVEK	2	0.9132	1
Q$810-1	P52895	AK1C2_HUMAN	AKR1C2	Aldo-keto reductase family 1 member C2	1.0000	29.1	5	5	10
						LNDGHFMPVLGFGTYAPAEVPK	3	0.9336	1
						NLQLDYVDLYLIHFPVSVKPGEEVIPK	3	0.9978	2
						QNVQVFEFQLTSEEMK	2	0.9997	3
						SIGVSNFNHR	2	0.9954	2
						YLTLDIFAGPPNYPFSDEY	2	0.9953	2
Q$811-1	P53597	SUCA_HUMAN	SUCLG1	Succinyl-CoA ligase [ADP/GDP-forming] subunit alpha, mitocl	0.9813	7.51	1	1	1
						EKISALQSAGVVVSMSPAQLGTTIYK	3	0.9127	1
Q$812-1	P53621	COPA_HUMAN	COPA	Coatomer subunit alpha	1.0000	6.62	6	6	8
Q$812-2	P53621-2	COPA_HUMAN	COPA	Isoform 2 of Coatomer subunit alpha	1.0000	6.57	6	6	8
						ASNLENSTYDLYTIPK	2	0.982	1
						CPLSGACYSPEFK	2	0.9995	2
						GFFEGTIASK	2	0.9979	1
						GITGVDLFGTTDAVVK	2	0.8849	1
						SSGLTAVWVAR	2	0.9083	1
						VTTVTEIGKDVIGLR	2	0.9992	2
Q$813-1	P53634	CATC_HUMAN	CTSC	Dipeptidyl peptidase 1	1.0000	23.54	9	11	22
						DPFNPFELTNHAVLLVGYGTDSASGMDYWIVK	3	0.9996	2
						GTDECAIESIAVAATPIPK	2	0.9995	3
						GTDECAIESIAVAATPIPK	3	0.9866	1
						GTDECAIESIAVAATPIPKL	2	0.9997	4
						GTDECAIESIAVAATPIPKL	3	0.9954	2
						NSWGTGWGENGYFR	2	0.9995	3
						NVHGINFVSPVR	3	0.996	1
						NWACFTGK	2	0.9735	1
						RGTDECAIESIAVAATPIPK	3	0.9967	2
						RGTDECAIESIAVAATPIPKL	3	0.9987	2
						YYSSEYHYVGGFYGGCNEALMK	3	0.998	1
Q$814-1	P53999	TCP4_HUMAN	SUB1	Activated RNA polymerase II transcriptional coactivator p15	1.0000	18.9	3	3	5
						EQISDIDDAVR	2	0.9431	1
						GISLNPEQWSQLK	2	0.9992	2
						GISLNPEQWSQLKEQISDIDDAVR	3	0.9972	2
Q$815-1	P54136	SYRC_HUMAN	RARS	Arginine--tRNA ligase, cytoplasmic	1.0000	14.39	8	8	12
						IVFVPGCSIPLTIVK	2	0.998	2
						LFEFAGYDVLR	2	0.9996	3
						LLQQEEEIK	2	0.9943	1
						LNDYIFSFDK	2	0.9971	1
						SDGGYTYDTSDLAAIK	2	0.9326	1
						SLTAEIDRLK	2	0.9876	1
						STIIGESISR	2	0.9856	2
						VEIAGPGFINVHLR	3	0.9926	1
Q$816-1	P54868	HMCS2_HUMAN	HMGCS2	Hydroxymethylglutaryl-CoA synthase, mitochondrial	1.0000	7.68	2	3	5
Q$816-2	P54868-2	HMCS2_HUMAN	HMGCS2	Isoform 2 of Hydroxymethylglutaryl-CoA synthase, mitochon	1.0000	8.37	2	3	5
Q$816-3	P54868-3	HMCS2_HUMAN	HMGCS2	Isoform 3 of Hydroxymethylglutaryl-CoA synthase, mitochon	1.0000	8.61	2	3	5
						IGAFSYGSGLAASFFSFR	2	0.9995	3
						IGAFSYGSGLAASFFSFR	3	0.9062	1
						VNFSPPGDTNSLFPGTWYLER	2	0.9988	1
Q$817-1	P55010	IF5_HUMAN	EIF5	Eukaryotic translation initiation factor 5	1.0000	2.32	2	2	4
						VNILFDFVK	2	0.9973	2
						VNILFDFVKK	3	0.9976	2
Q$818-1	P55072	TERA_HUMAN	VCP	Transitional endoplasmic reticulum ATPase	1.0000	30.4	18	19	35
						AIANECQANFISIK	2	0.9993	2
						AIGVKPPR	2	0.9519	1
						DVDLEFLAK	2	0.9869	1
						ETVVEVPQVTWEDIGGLEDVKR	3	0.9228	1
						EVDIGIPDATGR	2	0.9904	2
						GVLFYGPPGCGK	2	0.9972	1
						IVSQLLTLMDGLK	2	0.9921	1
						LADDVDLEQVANETHGHVGADLAALCSEAALQAIR	3	0.9177	1
						LAGESESNLR	2	0.9993	2
						LDQLIYIPLPDEK	2	0.9988	2
						LEILQIHTK	3	0.9818	1
						LGDVISIQPCPDVK	2	0.9993	2
						LIVDEAINEDNSVVSLSQPK	2	0.9996	3
						LIVDEAINEDNSVVSLSQPK	3	0.9964	2
						MDELQLFR	2	0.999	1
						NAPAIIFIDELDAIAPK	2	0.9996	5
						QAAPCVLFFDELDSIAK	2	0.9997	5
						VLFFDELDSIAK	2	0.8913	1
						WALSQSNPSALR	2	0.9997	1
Q$819-1	P55291	CAD15_HUMAN	CDH15	Cadherin-15	1.0000	2.7	1	2	4
						FSILQQGSPELFSIDELTGEIR	2	0.9997	2
						FSILQQGSPELFSIDELTGEIR	3	0.9993	2
Q$820-1	P55884	EIF3B_HUMAN	EIF3B	Eukaryotic translation initiation factor 3 subunit B	1.0000	2.83	2	2	4
Q$820-2	P55884-2	EIF3B_HUMAN	EIF3B	Isoform 2 of Eukaryotic translation initiation factor 3 subunit	1.0000	2.63	2	2	4
						GTQGVVTNFEIFR	2	0.999	2
						GTYLATFHQR	2	0.983	2
Q$821-1	P58107	EPIPL_HUMAN	EPPK1	Epiplakin	1.0000	2.1	6	7	12
						GFFDPNTHENLTYLQLLER	3	0.9904	3
						GFFDPNTHENLTYVQLLR	3	0.99	1
						GSAVHQLSEELR	2	0.9186	1
						LLDAQLATGGLVCPARR	3	0.9288	1
						LSVEEAVAAGVVGGEIQEK	3	0.9908	1
						VTPGSGALQGQSVSVWELLFYR	2	0.9993	2
						VTPGSGALQGQSVSVWELLFYR	3	0.9995	3
Q$822-1	P59665	DEF1_HUMAN	DEFA1	Neutrophil defensin 1	1.0000	22.34	6	7	25
Q$822-2	P59666	DEF3_HUMAN	DEFA3	Neutrophil defensin 3	1.0000	22.34	6	7	25
						IPACIAGER	2	0.9996	6
						IPACIAGERR	2	0.9994	5
						RIPACIAGER	2	0.9538	1
						RYGTCIYQGR	2	0.9989	3
						RYGTCIYQGR	3	0.9966	2
						YGTCIYQGR	2	0.9997	7
						YGTCIYQGRL	2	0.9838	1
Q$823-1	R4GN08	R4GN08_HUMAN	ARPC4	Actin-related protein 2/3 complex subunit 4 (Fragment)	1.0000	10.39	1	1	3
Q$823-2	P59998-4	ARPC4_HUMAN	ARPC4	Isoform 4 of Actin-related protein 2/3 complex subunit 4	1.0000	10.26	1	1	3

						AENFFILR	2	0.9995	3
Q$824-1	P60022	DEFB1_HUMAN	DEFB1	Beta-defensin 1	0.9985	33.82	1	1	2
						SDHYNCVSSGGQCLYSACPIFTK	3	0.9927	2
Q$825-1	P60174-4	TPIS_HUMAN	TPI1	Isoform 4 of Triosephosphate isomerase	1.0000	40.12	5	5	14
						DCGATWVVLGHSER	2	0.9997	3
						HVFGESDELIGQK	2	0.9996	4
						IIYGGSVTGATCK	2	0.9992	3
						SNVSDAVAQSTR	2	0.9997	1
						VVLAYEPVWAIGTGK	2	0.9997	3
Q$826-1	P60174-1	TPIS_HUMAN	TPI1	Isoform 2 of Triosephosphate isomerase	1.0000	79.92	17	22	49
Q$826-2	P60174	TPIS_HUMAN	TPI1	Triosephosphate isomerase	1.0000	69.58	17	22	49
						DCGATWVVLGHSER	2	0.9997	3
						ELASQPDVDGFLVGGASLKPEFVDIINAK	3	0.9813	3
						ELASQPDVDGFLVGGASLKPEFVDIINAKQ	3	0.8996	2
						HVFGESDELIGQK	2	0.9996	4
						HVFGESDELIGQK	3	0.9967	1
						IAVAAQNCYK	2	0.9997	1
						IIYGGSVTGATCK	2	0.9992	3
						KQSLGELIGTLNAAK	3	0.8281	1
						QSLGELIGTLNAAK	2	0.9995	3
						RHVFGESDELIGQK	2	0.9959	1
						RHVFGESDELIGQK	3	0.9994	2
						SNVSDAVAQSTR	2	0.9997	1
						VAHALAEGLGVIACIGEK	2	0.9997	3
						VAHALAEGLGVIACIGEK	3	0.9992	3
						VAHALAEGLGVIACIGEKLDER	3	0.9993	3
						VAHALAEGLGVIACIGEKLDEREAGITEK	4	0.8514	1
						VIADNVKDWSK	2	0.9978	2
						VPADTEVVCAPPTAYIDFAR	2	0.9997	5
						VPADTEVVCAPPTAYIDFAR	3	0.9168	1
						VTNGAFTGEISPGMIK	2	0.9994	1
						VVLAYEPVWAIGTGK	2	0.9997	3
						VVLAYEPVWAIGTGK	3	0.9995	2
Q$827-1	P60709	ACTB_HUMAN	ACTB	Actin, cytoplasmic 1	1.0000	73.6	34	41	102
Q$827-2	P63261	ACTG_HUMAN	ACTG1	Actin, cytoplasmic 2	1.0000	73.6	34	41	102
						AGFAGDDAPR	2	0.9997	5
						AIQAVLSLYASGR	2	0.9488	2
						ALDFEQEMATAASSSSLEK	2	0.9851	2
						ALPHAILR	2	0.9389	1
						AVFPSIVGRPR	2	0.9997	2
						AVLSLYASGR	2	0.9869	2
						CPEALFQPSFLGMESCGIHETTFNSIMK	3	0.9996	5
						DESGPSIVHR	2	0.9707	2
						DGQVITIGNER	2	0.9355	1
						DLTDYLMK	2	0.986	1
						DLYANTVLSGGTTMYPGIADR	2	0.9997	3
						DSYVGDEAQSKR	2	0.9997	2
						EITALAPSTMK	2	0.9992	2
						EKLCYVALDFEQEMATAASSSSLEK	3	0.9572	2
						ETFNTPAMYVAIQAVLSLYASGR	3	0.9813	3
						FRCPEALFQPSFLGMESCGIHETTFNSIMK	3	0.9988	2
						GYSFTTTAER	2	0.9996	4
						HQGVMVGMGQK	2	0.9979	1
						IIAPPERK	2	0.9994	2
						IWHHTFY	2	0.8665	1
						IWHHTFYNELR	2	0.9994	3
						IWHHTFYNELR	3	0.9993	2
						KDLYANTVLSGGTT	2	0.9773	2
						KDLYANTVLSGGTTMYPGIADR	2	0.9996	1
						KDLYANTVLSGGTTMYPGIADR	3	0.9995	2
						LCYVALDFEQEMATAASSSSLEK	2	0.9997	3
						LCYVALDFEQEMATAASSSSLEK	3	0.9996	3
						LDLAGRDLTDYLMK	2	0.9936	3
						LSLYASGR	2	0.9821	2
						QAVLSLYASGR	2	0.9604	1
						QEYDESGPSIVHR	2	0.9994	2
						QEYDESGPSIVHR	3	0.9827	1
						QEYDESGPSIVHRK	2	0.9878	1
						SYELPDGQVITIGNER	2	0.9998	13
						SYELPDGQVITIGNER	3	0.999	4
						TTGIVMDSGDGVTHTVPIYEGYALPHAILR	3	0.9995	3
						TTGIVMDSGDGVTHTVPIYEGYALPHAILR	4	0.9012	2
						VAIQAVLSLYASGR	2	0.992	2
						VAPEEHPVLLTEAPLNPK	2	0.9997	3
						VAPEEHPVLLTEAPLNPK	3	0.9981	2
						YELPDGQVITIGNER	2	0.9535	2
Q$828-1	P63000-2	RAC1_HUMAN	RAC1	Isoform B of Ras-related C3 botulinum toxin substrate 1	1.0000	13.74	3	3	6
Q$828-2	P63000	RAC1_HUMAN	RAC1	Ras-related C3 botulinum toxin substrate 1	1.0000	15.1	3	3	6
Q$828-3	P60763	RAC3_HUMAN	RAC3	Ras-related C3 botulinum toxin substrate 3	1.0000	15.1	3	3	6
						CVVVGDGAVGK	2	0.9993	4
						TVFDEAIR	2	0.8878	1
						YLECSALTQR	2	0.9867	1
Q$829-1	P60953	CDC42_HUMAN	CDC42	Cell division control protein 42 homolog	1.0000	21.47	3	3	6
						CVVVGDGAVGK	2	0.9993	4
						NVFDEAILAALEPPEPK	2	0.8882	1
						TPFLLVGTQIDLR	2	0.9987	1
Q$830-1	Q5JYX0	Q5JYX0_HUMAN	CDC42	Cell division control protein 42 homolog (Fragment)	1.0000	17.65	2	2	5
Q$830-2	P60953-1	CDC42_HUMAN	CDC42	Isoform 1 of Cell division control protein 42 homolog	1.0000	12.57	2	2	5
						CVVVGDGAVGK	2	0.9993	4
						TPFLLVGTQIDLR	2	0.9987	1
Q$831-1	P61026	RAB10_HUMAN	RAB10	Ras-related protein Rab-10	1.0000	17	3	3	5
						AFLTLAEDILR	2	0.9994	2
						LLLIGDSGVGK	2	0.985	2
						NIDEHANEDVER	2	0.9994	1
Q$832-1	P61158	ARP3_HUMAN	ACTR3	Actin-related protein 3	1.0000	10.77	4	4	6
						DITYFIQQLLR	2	0.9994	2
						EFSIDVGYER	2	0.9812	1
						LPACVVDCGTGYTK	2	0.9988	2
						YSYVCPDLVK	2	0.9973	1
Q$833-1	P61160	ARP2_HUMAN	ACTR2	Actin-related protein 2	1.0000	18.53	5	5	6
						CGYAGSNFPEHIFPALVGRPIIR	3	0.8743	1
						DLMVGDEASELR	2	0.9955	2
						GYAFNHSADFETVR	2	0.999	1
						HLWDYTFGPEK	2	0.9836	1
						LCYVGYNIEQEQK	2	0.9829	1
Q$834-1	P62834	RAP1A_HUMAN	RAP1A	Ras-related protein Rap-1A	1.0000	12.5	2	2	3
Q$834-2	P61224-2	RAP1B_HUMAN	RAP1B	Isoform 2 of Ras-related protein Rap-1b	1.0000	16.79	2	2	3
Q$834-3	P61224-3	RAP1B_HUMAN	RAP1B	Isoform 3 of Ras-related protein Rap-1b	1.0000	13.94	2	2	3
Q$834-4	P61224-4	RAP1B_HUMAN	RAP1B	Isoform 4 of Ras-related protein Rap-1b	1.0000	16.2	2	2	3
Q$834-5	P61224	RAP1B_HUMAN	RAP1B	Ras-related protein Rap-1b	1.0000	12.5	2	2	3
						INVNEIFYDLVR	2	0.9997	2
						LVVLGSGGVGK	2	0.9992	1
Q$835-1	P61586	RHOA_HUMAN	RHOA	Transforming protein RhoA	1.0000	37.31	7	7	12
						EVFEMATR	2	0.8642	1
						HFCPNVPIILVGNKK	3	0.9995	2
						IGAFGYMECSAK	2	0.9988	2
						KLVIVGDGACGK	2	0.9964	1
						LRPLSYPDTDVILMCF	2	0.8263	1
						LVIVGDGACGK	2	0.984	2
						TCLLIVFSK	2	0.9996	3
Q$836-1	P61626	LYSC_HUMAN	LYZ	Lysozyme C	1.0000	52.03	8	8	13
						GISLANWMCLAK	2	0.9984	1
						LGMDGYR	2	0.9993	1

						RLGMDGYR	2	0.9919	1
						SALLQDNIADAVACAK	2	0.9892	2
						STDYGIFQINSR	2	0.9996	4
						TPGAVNACHLSCSALLQDNIADAVACAK	3	0.9282	2
						VVRDPQGIR	2	0.9785	1
						WESGYNTR	2	0.9903	1
Q$837-1	P61970	NTF2_HUMAN	NUTF2	Nuclear transport factor 2	1.0000	74.8	7	8	14
						ADEDPIMGFHQMFLLK	2	0.9994	1
						ADEDPIMGFHQMFLLK	3	0.9917	3
						IQHSITAQDHQPTPDSCIISMVVGQLK	3	0.8574	1
						LALHNFG	2	0.9994	1
						LSSLPFQK	2	0.9981	1
						LSSLPFQKI	2	0.8813	1
						NINDAWVCTNDMFR	2	0.9997	3
						TQLGAIYIDASCLTWEGQQFQGK	3	0.987	3
Q$838-1	P61978	HNRPK_HUMAN	HNRNPK	Heterogeneous nuclear ribonucleoprotein K	1.0000	8.86	3	4	5
Q$838-2	Q5T6W1	Q5T6W1_HUMAN	HNRNPK	Heterogeneous nuclear ribonucleoprotein K	1.0000	13.4	3	4	5
Q$838-3	Q5T6W5	Q5T6W5_HUMAN	HNRNPK	Heterogeneous nuclear ribonucleoprotein K	1.0000	9.58	3	4	5
Q$838-4	Q5T6W2	Q5T6W2_HUMAN	HNRNPK	Heterogeneous nuclear ribonucleoprotein K (Fragment)	1.0000	10.82	3	4	5
Q$838-5	P61978-2	HNRPK_HUMAN	HNRNPK	Isoform 2 of Heterogeneous nuclear ribonucleoprotein K	1.0000	8.84	3	4	5
Q$838-6	P61978-3	HNRPK_HUMAN	HNRNPK	Isoform 3 of Heterogeneous nuclear ribonucleoprotein K	1.0000	9.32	3	4	5
						GSDFDCELR	2	0.9962	1
						ILSISADIETIGEILKK	3	0.9021	1
						LLIHQSLAGGIIGVK	2	0.9917	1
						LLIHQSLAGGIIGVK	3	0.9986	2
Q$839-1	P61981	1433G_HUMAN	YWHAG	14-3-3 protein gamma	1.0000	19.03	4	4	6
						DSTLIMQLLR	2	0.997	1
						LGLALNYSVFYYEIQNAPEQACHLAK	3	0.988	2
						YLAEVATGEK	2	0.8873	1
						YLAEVATGEKR	2	0.9987	2
Q$840-1	P62081	RS7_HUMAN	RPS7	40S ribosomal protein S7	0.9973	11.86	2	2	2
						AIIIFVPVPQLK	2	0.8956	1
						DVNFEFPEFQL	2	0.9743	1
Q$841-1	P62140	PP1B_HUMAN	PPP1CB	Serine/threonine-protein phosphatase PP1-beta catalytic sub	1.0000	11.93	4	4	6
						EIFLSQPILLELEAPLK	2	0.9997	3
						FLNRHDLDLICR	3	0.8421	1
						HDLDLICR	2	0.937	1
						IVQMTEAEVR	2	0.9977	1
Q$842-1	P62195	PRS8_HUMAN	PSMC5	26S protease regulatory subunit 8	1.0000	17.98	5	5	7
Q$842-2	P62195-2	PRS8_HUMAN	PSMC5	Isoform 2 of 26S protease regulatory subunit 8	1.0000	18.34	5	5	7
						GVCTEAGMYALR	2	0.9541	1
						IDILDSALLRPGR	3	0.963	1
						IEELQLIVNDK	2	0.9964	2
						LLREELQLLQEQGSYVGEVVR	3	0.9983	2
						TMLELLNQLDGFEATK	2	0.8268	1
Q$843-1	P62241	RS8_HUMAN	RPS8	40S ribosomal protein S8	1.0000	23.56	4	5	9
						ELEFYLR	2	0.9757	1
						IIDVVYNASNNELVR	2	0.9997	2
						IIDVVYNASNNELVR	3	0.9908	2
						LDVGNFSWGSECCTR	2	0.9995	2
						NCIVLIDSTPYR	2	0.999	2
Q$844-1	P62258	1433E_HUMAN	YWHAE	14-3-3 protein epsilon	1.0000	19.61	4	4	6
Q$844-2	P62258-2	1433E_HUMAN	YWHAE	Isoform SV of 14-3-3 protein epsilon	1.0000	21.46	4	4	6
						HLIPAANTGESK	2	0.9831	1
						LICCDILDVLDK	2	0.9959	2
						SVFYYEILNSPDR	2	0.9476	2
						VAGMDVELTVEER	2	0.9994	1
Q$845-1	P62333	PRS10_HUMAN	PSMC6	26S protease regulatory subunit 10B	1.0000	12.34	4	4	8
						ALQSVGQIVGEVLK	2	0.9971	2
						HGEIDYEAIVK	2	0.9989	3
						HGEIDYEAIVKLSDGFNGADLR	3	0.8186	1
						NVCTEAGMFAIR	2	0.9988	2
Q$846-1	P62424	RL7A_HUMAN	RPL7A	60S ribosomal protein L7a	0.9999	8.65	2	2	3
Q$846-2	Q5T8U2	Q5T8U2_HUMAN	RPL7A	60S ribosomal protein L7a	0.9999	15.23	2	2	3
						AGVNTVTTLVENK	2	0.999	2
						HWGGNVLGPK	2	0.9493	1
Q$847-1	P62701	RS4X_HUMAN	RPS4X	40S ribosomal protein S4, X isoform	1.0000	20.91	5	5	9
						FDTGNLCMVTGGANLGR	2	0.9997	2
						LSNIFVIGK	2	0.999	1
						TIRYPDPLIK	3	0.9855	1
						VNDTIQIDLETGK	2	0.9991	2
						VNDTIQIDLETGKITDFIK	3	0.9958	3
Q$848-1	P62714	PP2AB_HUMAN	PPP2CB	Serine/threonine-protein phosphatase 2A catalytic subunit be	1.0000	12.3	3	3	6
						ELDQWVEQLNECK	2	0.9823	1
						NVVTIFSAPNYCYR	2	0.9996	2
						YSFLQFDPAPR	2	0.9996	3
Q$849-1	P62745	RHOB_HUMAN	RHOB	Rho-related GTP-binding protein RhoB	1.0000	4.59	1	1	3
						TCLLIVFSK	2	0.9996	3
Q$850-1	P62805	H4_HUMAN	HIST1H4A	Histone H4	1.0000	27.18	4	4	9
						DAVTYTEHAK	2	0.9995	4
						ISGLIYEETR	2	0.9996	3
						SGLIYEETR	2	0.9895	1
						VFLENVIR	2	0.9989	1
Q$851-1	P62820	RAB1A_HUMAN	RAB1A	Ras-related protein Rab-1A	1.0000	28.29	4	4	7
						EFADSLGIPFLETSAK	2	0.9674	2
						FADDTYTESYISTIGVDFK	2	0.9994	2
						LLLIGDSGVGK	2	0.985	2
						MGPGATAGGAEK	2	0.9107	1
Q$852-1	P62873	GBB1_HUMAN	GNB1	Guanine nucleotide-binding protein G(I)/G(S)/G(T) subunit be	1.0000	18.82	4	4	5
						ELAGHTGYLSCCR	2	0.9973	1
						LLLAGYDDFNCNVWDALK	2	0.9995	2
						LLVSASQDGK	2	0.9815	1
						VSCLGVTDDGMAVATGSWDSFLK	2	0.9993	1
Q$853-1	P62873-2	GBB1_HUMAN	GNB1	Isoform 2 of Guanine nucleotide-binding protein G(I)/G(S)/G(	1.0000	12.35	3	3	4
						ELAGHTGYLSCCR	2	0.9973	1
						LLLAGYDDFNCNVWDALK	2	0.9995	2
						LLVSASQDGK	2	0.9815	1
Q$854-1	P62937	PPIA_HUMAN	PPIA	Peptidyl-prolyl cis-trans isomerase A	1.0000	74.55	10	13	33
						DIAVDGEPLGR	2	0.9791	2
						EGMNIVEAMER	2	0.9942	1
						HTGPGILSMANAGPNTNGSQFFICTAK	3	0.9983	2
						IIPGFMCQGGDFTR	2	0.9992	5
						KITIADCGQLE	2	0.9997	3
						SIYGEKFEDENFILK	2	0.9993	1
						TEWLDGKHVVFGK	2	0.9973	2
						TEWLDGKHVVFGK	3	0.9032	1
						VKEGMNIVEAMER	2	0.9979	1
						VKEGMNIVEAMER	3	0.8071	1
						VNPTVFFDIAVDGEPLGR	2	0.9997	5
						VSFELFADKVPK	2	0.9991	5
						VSFELFADKVPK	3	0.9863	4
Q$855-1	P62995-3	TRA2B_HUMAN	TRA2B	Isoform 3 of Transformer-2 protein homolog beta	1.0000	15.96	2	2	3
Q$855-2	P62995	TRA2B_HUMAN	TRA2B	Transformer-2 protein homolog beta	1.0000	10.42	2	2	3
						GFAFVYFENVDDAK	2	0.999	1
						YGPIADVSIVYDQQSR	2	0.9982	2
Q$856-1	P63151-2	2ABA_HUMAN	PPP2R2A	Isoform 2 of Serine/threonine-protein phosphatase 2A 55 kD	1.0000	7.88	3	3	7
Q$856-2	P63151	2ABA_HUMAN	PPP2R2A	Serine/threonine-protein phosphatase 2A 55 kDa regulatory	1.0000	8.05	3	3	7
						INLWHLEITDR	2	0.9972	2
						SFFSEIISSISDVK	2	0.9994	2
						VVIFQQEQENK	2	0.9989	3
Q$857-1	P63241	IF5A1_HUMAN	EIF5A	Eukaryotic translation initiation factor 5A-1	1.0000	34.42	3	3	6
Q$857-2	P63241-2	IF5A1_HUMAN	EIF5A	Isoform 2 of Eukaryotic translation initiation factor 5A-1	1.0000	28.8	3	3	6
						EDLRLPEGDLGKEIEQK	3	0.8604	1

						VHLVGIDIFTGK	3	0.9992	2
						YDCGEEILITVLSAMTEEAAVAIK	3	0.9996	3
Q$858-1	P63244	GBLP_HUMAN	GNB2L1	Guanine nucleotide-binding protein subunit beta-2-like 1	1.0000	46.37	10	10	15
						DETNYGIPQR	2	0.9825	1
						DVLSVAFSSDNR	2	0.983	1
						FSPNSSNPIIVSCGWDK	2	0.9995	2
						GHNGWVTQIATTPQFPDMILSASR	3	0.9516	1
						HLYTLDGGDIINALCFSPNR	3	0.9973	3
						IIVDELKQEVISTSSK	2	0.9995	2
						LWDLTTGTTTR	2	0.938	1
						VWQVTIGTR	2	0.9992	2
						YTVQDESHSEWVSCVR	3	0.9927	1
						YWLCAATGPSIK	2	0.9997	1
Q$859-1	P67775-2	PP2AA_HUMAN	PPP2CA	Isoform 2 of Serine/threonine-protein phosphatase 2A cataly	1.0000	14.9	3	3	8
Q$859-2	P67775	PP2AA_HUMAN	PPP2CA	Serine/threonine-protein phosphatase 2A catalytic subunit al	1.0000	12.3	3	3	8
						ELDQWIEQLNECK	2	0.9995	3
						NVVTIFSAPNYCYR	2	0.9996	2
						YSFLQFDPAPR	2	0.9996	3
Q$860-1	P68104	EF1A1_HUMAN	EEF1A1	Elongation factor 1-alpha 1	1.0000	26.19	12	15	29
Q$860-2	Q5VTE0	EF1A3_HUMAN	EEF1A1P5	Putative elongation factor 1-alpha-like 3	1.0000	26.19	12	15	29
						AAGVGEFEAGISK	2	0.919	1
						EHALLAYTLGVK	2	0.9997	3
						EHALLAYTLGVK	3	0.9996	3
						EVSTYIK	2	0.9591	1
						EVSTYIKK	2	0.9833	1
						IGGIGTVPVGR	2	0.9997	2
						LPLQDVYK	2	0.9709	1
						QLIVGVNK	2	0.8689	1
						QTVAVGVIK	2	0.9995	1
						STTTGHLIYK	2	0.9997	3
						VETGVLKPGMVVTFAPVNVTTEVK	3	0.9993	3
						YEEIVKEVSTYIK	2	0.9988	3
						YEEIVKEVSTYIK	3	0.9955	1
						YYVTIIDAPGHR	2	0.9997	3
						YYVTIIDAPGHR	3	0.9993	2
Q$861-1	P68363	TBA1B_HUMAN	TUBA1B	Tubulin alpha-1B chain	1.0000	12.42	6	6	19
						AVCMLSNTTAIAEAWAR	2	0.9996	5
						DVNAAIATIK	2	0.999	2
						EIIDLVLDR	2	0.9997	4
						GHYTIGKEIIDLVLDR	3	0.9994	2
						MLSNTTAIAEAWAR	2	0.9183	2
						SIQFVDWCPTGFK	2	0.9997	4
Q$862-1	P68366-2	TBA4A_HUMAN	TUBA4A	Isoform 2 of Tubulin alpha-4A chain	1.0000	23.56	10	11	28
Q$862-2	P68366	TBA4A_HUMAN	TUBA4A	Tubulin alpha-4A chain	1.0000	22.77	10	11	28
						AVCMLSNTTAIAEAWAR	2	0.9996	5
						AVCMLSNTTAIAEAWAR	3	0.9984	2
						AVFVDLEPTVIDEIR	2	0.9993	3
						AYHEQLSVAEITNACFEPA	2	0.9601	2
						AYHEQLSVAEITNACFEPANQMVK	3	0.9994	3
						DVNAAIAAIK	2	0.9937	1
						MLSNTTAIAEAWAR	2	0.9183	2
						SIQFVDWCPTGFK	2	0.9997	4
						SIQFVDWCPTGFKV	2	0.9786	2
						SIQFVDWCPTGFKVGI	2	0.8202	1
						TIGGGDDSFTTFFCETGAGK	2	0.9997	3
Q$863-1	P68371	TBB4B_HUMAN	TUBB4B	Tubulin beta-4B chain	1.0000	25.39	9	9	34
						ALTVPELTQQMFDAK	2	0.9994	4
						AVLVDLEPGTMDSVR	2	0.9997	7
						FWEVISDEHGIDPTGTYHGDSDLQLER	3	0.9997	8
						INVYYNEATGGK	2	0.9997	4
						INVYYNEATGGKY	2	0.9722	1
						INVYYNEATGGKYVPR	2	0.9996	3
						LHFFMPGFAPLTSR	3	0.9695	2
						MSATFIGNSTAIQELFK	2	0.8786	1
						YLTVAAVFR	2	0.9997	4
Q$864-1	P68871	HBB_HUMAN	HBB	Hemoglobin subunit beta	1.0000	95.92	112	153	475
						AFSDGLAHLDNLK	2	0.9924	5
						AGVANALAHKYH	2	0.9134	1
						ALWGKVNVDEVGGEALGR	2	0.9934	3
						ALWGKVNVDEVGGEALGR	3	0.9401	2
						ATLSELHCDK	2	0.9013	1
						ATLSELHCDKLHVDPENFR	2	0.9749	3
						ATLSELHCDKLHVDPENFR	3	0.9066	1
						AVTALWGKVNVDEVGGEALGR	3	0.8119	1
						DEVGGEALGR	2	0.9943	3
						DKLHVDPENFR	2	0.9673	3
						DKLHVDPENFR	3	0.9414	2
						EFTPPVQ	2	0.9852	2
						EFTPPVQAA	2	0.9036	1
						EFTPPVQAAY	2	0.9937	2
						EFTPPVQAAYQ	2	0.9971	2
						EFTPPVQAAYQK	2	0.9997	8
						EFTPPVQAAYQK	3	0.9774	1
						EFTPPVQAAYQKV	2	0.9957	2
						EKSAVTALWGK	2	0.9214	1
						FATLSELHCDKLHVDPENFR	3	0.9151	1
						FESFGDLSTPDAVMGNPK	2	0.9978	4
						FFESFGDLS	2	0.939	1
						FFESFGDLSTP	2	0.8555	1
						FFESFGDLSTPD	2	0.9975	5
						FFESFGDLSTPDA	2	0.9935	3
						FFESFGDLSTPDAV	2	0.9986	6
						FFESFGDLSTPDAVMGNPK	2	0.9998	19
						FFESFGDLSTPDAVMGNPK	3	0.9996	3
						FFESFGDLSTPDAVMGNPKV	2	0.9267	1
						FSDGLAHLDNLK	2	0.9345	3
						FSDGLAHLDNLK	3	0.8507	1
						GAFSDGLAHLDNLK	2	0.9951	6
						GKVNVDEVGGEALGR	2	0.9861	3
						GKVNVDEVGGEALGR	3	0.9361	1
						GLAHLDNLK	2	0.9422	1
						GNVLVCVLAHHFGK	2	0.9765	2
						GNVLVCVLAHHFGK	4	0.984	1
						GTFATLSELH	2	0.9963	2
						GTFATLSELHC	2	0.9972	2
						GTFATLSELHCD	2	0.9956	3
						GTFATLSELHCDK	2	0.9997	4
						GTFATLSELHCDK	3	0.9498	1
						GTFATLSELHCDKL	2	0.9511	2
						GTFATLSELHCDKLHVD	2	0.9595	2
						GTFATLSELHCDKLHVDPEN	2	0.924	1
						GTFATLSELHCDKLHVDPENFR	2	0.9996	4
						GTFATLSELHCDKLHVDPENFR	3	0.9995	8
						GTFATLSELHCDKLHVDPENFR	4	0.9978	1
						HCDKLHVDPENFR	2	0.9801	2
						HCDKLHVDPENFR	3	0.8269	1
						HLTPEEK	2	0.9103	2
						HVDPENFR	2	0.9515	1
						KEFTPPVQAAYQK	2	0.9652	1
						KLHVDPENFR	2	0.9703	1
						KLHVDPENFR	3	0.8815	1
						KSAVTALWGK	3	0.8747	1
						KVLGAFSD	2	0.8856	1

						KVLGAFSDGLAHLD	2	0.961	2
						KVLGAFSDGLAHLDN	2	0.9751	3
						KVLGAFSDGLAHLDNL	2	0.9889	2
						KVLGAFSDGLAHLDNLK	2	0.9997	6
						KVLGAFSDGLAHLDNLK	3	0.9997	12
						KVLGAFSDGLAHLDNLK	4	0.9375	1
						KVNVDEVGGEALGR	2	0.9974	2
						KVNVDEVGGEALGR	3	0.9823	1
						LAHHFGK	2	0.9225	1
						LAHLDNLK	2	0.8465	1
						LGNVLVCVLAHHFGK	4	0.8854	1
						LHCDKLHVDPENFR	2	0.9735	2
						LHCDKLHVDPENFR	3	0.8133	1
						LHVDPENFR	2	0.9997	7
						LHVDPENFR	3	0.9996	4
						LLGNVLVC	2	0.9772	1
						LLGNVLVCV	2	0.9694	2
						LLGNVLVCVL	2	0.9978	2
						LLGNVLVCVLA	2	0.9959	2
						LLGNVLVCVLAH	2	0.998	4
						LLGNVLVCVLAHH	2	0.9971	2
						LLGNVLVCVLAHH	3	0.987	2
						LLGNVLVCVLAHHF	2	0.9974	5
						LLGNVLVCVLAHHF	3	0.9875	2
						LLGNVLVCVLAHHFG	2	0.9952	4
						LLGNVLVCVLAHHFG	3	0.9826	1
						LLGNVLVCVLAHHFGK	2	0.9997	5
						LLGNVLVCVLAHHFGK	3	0.9997	6
						LLGNVLVCVLAHHFGK	4	0.9995	9
						LLGNVLVCVLAHHFGKE	2	0.9975	4
						LLGNVLVCVLAHHFGKE	3	0.9892	3
						LLGNVLVCVLAHHFGKE	4	0.9959	3
						LLGNVLVCVLAHHFGKEFTPPVQ	3	0.8397	1
						LLVVYPW	2	0.9962	2
						LLVVYPWT	2	0.9952	3
						LLVVYPWTQ	2	0.9963	2
						LLVVYPWTQR	3	0.9994	5
						LSELHCDKLHVDPENFR	2	0.9902	3
						LSELHCDKLHVDPENFR	3	0.9641	1
						LVVYPWTQR	2	0.996	3
						LWGKVNVDEVGGEALGR	3	0.9507	1
						NVDEVGGEALGR	2	0.871	1
						NVLVCVLAHHFGK	2	0.9959	2
						NVLVCVLAHHFGK	4	0.9885	2
						RLLGNVLVCVLAHHFGK	4	0.8258	1
						RLLVVYPWTQR	2	0.9957	4
						RLLVVYPWTQR	3	0.9801	2
						SAVTALWGK	2	0.9997	10
						SAVTALWGKV	2	0.835	1
						SAVTALWGKVNVDEVGGEA	2	0.8945	2
						SAVTALWGKVNVDEVGGEALGR	2	0.9996	6
						SAVTALWGKVNVDEVGGEALGR	3	0.9996	14
						SAVTALWGKVNVDEVGGEALGR	4	0.9949	1
						SDGLAHLDNLK	2	0.9895	5
						SDGLAHLDNLK	3	0.8253	1
						SELHCDKLHVDPENFR	2	0.9888	3
						SELHCDKLHVDPENFR	3	0.8897	1
						SELHCDKLHVDPENFR	4	0.9011	1
						TALWGKVNVDEVGGEALGR	2	0.9919	2
						TALWGKVNVDEVGGEALGR	3	0.956	1
						TLSELHCDK	2	0.977	1
						TLSELHCDKLHVDPENFR	2	0.9883	3
						TLSELHCDKLHVDPENFR	3	0.9697	2
						TPPVQAAYQK	2	0.9045	1
						VAGVANALAHK	2	0.9934	2
						VAGVANALAHKYH	2	0.9256	1
						VCVLAHHFGK	2	0.9923	3
						VDEVGGEALGR	2	0.9969	4
						VDPENFR	2	0.94	1
						VGGEALGR	2	0.9882	2
						VHLTPEEK	2	0.9997	11
						VLAHHFGK	2	0.9887	2
						VLGAFSDGLAH	2	0.8917	1
						VLGAFSDGLAHL	2	0.9865	6
						VLGAFSDGLAHLD	2	0.992	4
						VLGAFSDGLAHLDN	2	0.9941	4
						VLGAFSDGLAHLDNL	2	0.9966	8
						VLGAFSDGLAHLDNLK	2	0.9998	18
						VLGAFSDGLAHLDNLK	3	0.9997	13
						VLGAFSDGLAHLDNLKGT	2	0.9329	2
						VLVCVLAHHFGK	2	0.9919	2
						VNVDEVGGEALG	2	0.9976	3
						VNVDEVGGEALGR	2	0.9998	20
						VNVDEVGGEALGR	3	0.9996	4
						VNVDEVGGEALGRL	2	0.9984	6
						VVAGVANALA	2	0.8581	1
						VVAGVANALAH	2	0.9932	2
						VVAGVANALAHK	2	0.9997	3
						VVAGVANALAHK	3	0.9993	1
						VVAGVANALAHKY	2	0.9757	2
						VVAGVANALAHKYH	2	0.9996	3
						VVAGVANALAHKYH	3	0.9919	1
						VVAGVANALAHKYH	4	0.9959	1
						VVYPWTQR	2	0.9788	2
						WGKVNVDEVGGEALGR	2	0.9888	3
						WGKVNVDEVGGEALGR	3	0.9445	1
Q$865-1	P69891	HBG1_HUMAN	HBG1	Hemoglobin subunit gamma-1	1.0000	23.81	3	3	5
Q$865-2	P69892	HBG2_HUMAN	HBG2	Hemoglobin subunit gamma-2	1.0000	23.81	3	3	5
						EFTPEVQASWQK	2	0.9978	2
						VLTSLGDAIK	2	0.9761	1
						VNVEDAGGETLGR	2	0.9997	2
Q$866-1	P69905	HBA_HUMAN	HBA1	Hemoglobin subunit alpha	1.0000	85.92	62	87	202
						AAHLPAEFTPAVHASLDK	2	0.9975	2
						AAHLPAEFTPAVHASLDK	3	0.9926	4
						AAHLPAEFTPAVHASLDK	4	0.9913	2
						AEFTPAVHASLDK	2	0.9906	2
						AGEYGAEALER	2	0.9932	2
						AHLPAEFTPAVHASLDK	2	0.996	3
						AHLPAEFTPAVHASLDK	3	0.9929	2
						AHLPAEFTPAVHASLDK	4	0.994	1
						AHVDDMPNALSALSDLHAHK	3	0.9528	1
						CLLVTLAAHLPAEFTPAVHASLDK	3	0.9735	4
						CLLVTLAAHLPAEFTPAVHASLDK	4	0.9854	2
						FLASVSTVLTSK	2	0.9997	6
						FLASVSTVLTSKY	2	0.9915	2
						GAHAGEYGAEALER	2	0.9954	3
						GEYGAEALER	2	0.9668	1
						GKVGAHAGEYGAEALER	2	0.9893	2
						GKVGAHAGEYGAEALER	3	0.8428	1
						HAGEYGAEALER	2	0.9769	1
						HLPAEFTPAVHASLDK	2	0.9909	3
						HLPAEFTPAVHASLDK	3	0.993	1
						KVADALTNAVAHVDDMPNALSALSDLHAHK	3	0.9979	1

						KVADALTNAVAHVDDMPNALSALSDLHAHK	4	0.9981	2
						KVGAHAGEYGAEALER	2	0.9875	1
						LLSHCLL	2	0.9592	1
						LLSHCLLV	2	0.9908	1
						LLSHCLLVT	2	0.9293	1
						LLSHCLLVTL	2	0.9846	2
						LLSHCLLVTLA	2	0.8432	1
						LLSHCLLVTLAAH	3	0.8717	1
						LLSHCLLVTLAAHL	2	0.9765	3
						LLSHCLLVTLAAHL	3	0.8753	2
						LLSHCLLVTLAAHLP	2	0.9385	1
						LLSHCLLVTLAAHLP	3	0.9498	2
						LLSHCLLVTLAAHLPA	2	0.9748	2
						LLSHCLLVTLAAHLPA	3	0.9931	3
						LLSHCLLVTLAAHLPAE	3	0.9925	3
						LLSHCLLVTLAAHLPAEF	2	0.8458	1
						LLSHCLLVTLAAHLPAEFT	2	0.9716	1
						LLSHCLLVTLAAHLPAEFTPA	2	0.9974	4
						LLSHCLLVTLAAHLPAEFTPA	3	0.9493	1
						LLSHCLLVTLAAHLPAEFTPAV	2	0.984	2
						LLSHCLLVTLAAHLPAEFTPAV	3	0.9893	2
						LLSHCLLVTLAAHLPAEFTPAVH	3	0.9907	6
						LLSHCLLVTLAAHLPAEFTPAVH	4	0.995	4
						LLSHCLLVTLAAHLPAEFTPAVHA	3	0.9821	5
						LLSHCLLVTLAAHLPAEFTPAVHA	4	0.9903	2
						LLSHCLLVTLAAHLPAEFTPAVHAS	3	0.9774	2
						LLSHCLLVTLAAHLPAEFTPAVHASL	3	0.9473	4
						LLSHCLLVTLAAHLPAEFTPAVHASLD	3	0.8408	2
						LLSHCLLVTLAAHLPAEFTPAVHASLD	4	0.889	1
						LLSHCLLVTLAAHLPAEFTPAVHASLDK	3	0.9991	5
						LLSHCLLVTLAAHLPAEFTPAVHASLDK	4	0.9996	6
						LLSHCLLVTLAAHLPAEFTPAVHASLDKF	4	0.9168	1
						LPAEFTPAVHASLDK	2	0.9916	2
						LRVDPVNFK	2	0.9995	2
						LRVDPVNFK	3	0.9995	4
						LSALSDLHAHK	2	0.8625	1
						LVTLAAHLPAEFTPAVHASLDK	3	0.8511	2
						MFLSFPTTK	2	0.9997	4
						MFLSFPTTKT	2	0.985	2
						PAEFTPAVHASLDK	2	0.9517	3
						PAEFTPAVHASLDK	3	0.8465	1
						PAVHASLDK	2	0.9096	1
						PHFDLSHGSAQVK	3	0.9446	1
						TLAAHLPAEFTPAVHASLDK	2	0.966	1
						TLAAHLPAEFTPAVHASLDK	3	0.993	3
						TLAAHLPAEFTPAVHASLDK	4	0.9947	2
						TPAVHASLDK	2	0.8443	1
						TYFPHFD	2	0.9842	1
						TYFPHFDL	2	0.9933	5
						TYFPHFDLSHG	2	0.9056	2
						TYFPHFDLSHGSAQ	2	0.9745	2
						TYFPHFDLSHGSAQV	2	0.9945	1
						TYFPHFDLSHGSAQVK	2	0.9997	6
						TYFPHFDLSHGSAQVK	4	0.9947	2
						VADALTNAVAH	2	0.9585	1
						VADALTNAVAHV	2	0.9751	1
						VADALTNAVAHVDDMPNAL	2	0.9362	1
						VADALTNAVAHVDDMPNALSALSDLHAHK	3	0.9995	6
						VADALTNAVAHVDDMPNALSALSDLHAHK	4	0.9858	1
						VGAHAGEYGA	2	0.8518	1
						VGAHAGEYGAEALE	2	0.988	2
						VGAHAGEYGAEALER	2	0.9997	7
						VGAHAGEYGAEALER	3	0.9996	6
						VGAHAGEYGAEALERM	2	0.9175	1
						VTLAAHLPAEFTPAVHASLDK	3	0.9861	4
						VTLAAHLPAEFTPAVHASLDK	4	0.9491	1
Q$867-1	P78371	TCPB_HUMAN	CCT2	T-complex protein 1 subunit beta	1.0000	29.53	9	13	27
						AAHSEGNTTAGLDMR	2	0.9985	1
						EGTIGDMAILGITESFQVK	2	0.9996	4
						EGTIGDMAILGITESFQVK	3	0.9957	3
						GATQQILDEAER	2	0.9992	3
						HGINCFINR	2	0.9822	1
						LTSFIGAIAIGDLVK	2	0.9997	3
						LTSFIGAIAIGDLVK	3	0.9959	2
						MLPTIIADNAGYDSADLVAQLR	3	0.961	1
						QLIYNYPEQLFGAAGVMAIEHADFAGVER	3	0.9992	3
						QVLLSAAEAAEVILR	2	0.9997	3
						QVLLSAAEAAEVILR	3	0.9352	1
						VQDDEVGDGTTSVTVLAAELLR	2	0.9996	1
						VQDDEVGDGTTSVTVLAAELLR	3	0.9103	1
Q$868-1	P78371-2	TCPB_HUMAN	CCT2	Isoform 2 of T-complex protein 1 subunit beta	1.0000	2.46	1	1	3
						GATQQILDEAER	2	0.9992	3
Q$869-1	P80188-2	NGAL_HUMAN	LCN2	Isoform 2 of Neutrophil gelatinase-associated lipocalin	1.0000	14.14	2	2	6
						VPLQQNFQDNQFQGK	2	0.9996	2
						WYVVGLAGNAILR	2	0.9994	4
Q$870-1	P80511	S10AC_HUMAN	S100A12	Protein S100-A12	1.0000	72.83	10	15	35
						AVIDEIFQGLDANQDEQVDF	2	0.9397	1
						AVIDEIFQGLDANQDEQVDFQEFISLVAIALK	3	0.9994	5
						AVIDEIFQGLDANQDEQVDFQEFISLVAIALK	4	0.999	3
						DKAVIDEIFQGLDANQDEQVDFQEFISLVAIALK	3	0.8865	1
						GHFDTLSK	2	0.9979	1
						GHFDTLSKGELK	2	0.9895	2
						KGHFDTLSKGELK	2	0.9966	3
						KGHFDTLSKGELK	3	0.9782	1
						LEEHLEGIVNIFH	2	0.9696	2
						LEEHLEGIVNIFH	3	0.9406	2
						LEEHLEGIVNIFHQY	2	0.9641	2
						LEEHLEGIVNIFHQYSVR	3	0.9995	4
						LEEHLEGIVNIFHQYSVR	4	0.9989	2
						TKLEEHLEGIVNIFHQYSVR	3	0.9991	4
						TKLEEHLEGIVNIFHQYSVR	4	0.9961	2
Q$871-1	P80748	LV302_HUMAN		Ig lambda chain V-III region LOI	0.9214	14.41	1	1	1
						FSGSNSGNTATLTISR	2	0.9214	1
Q$872-1	P81605	DCD_HUMAN	DCD	Dermcidin	1.0000	25.45	2	3	5
						GAVHDVKDVLDSVL	2	0.9375	1
						LGKDAVEDLESVGK	2	0.9991	2
						LGKDAVEDLESVGK	3	0.9984	2
Q$873-1	P81605-2	DCD_HUMAN	DCD	Isoform 2 of Dermcidin	1.0000	11.57	1	2	4
						LGKDAVEDLESVGK	2	0.9991	2
						LGKDAVEDLESVGK	3	0.9984	2
Q$874-1	P98160	PGBM_HUMAN	HSPG2	Basement membrane-specific heparan sulfate proteoglycan c	1.0000	6.01	19	19	31
						AELLQVLQSLEAVLIQTVYNTK	3	0.9996	3
						AELLVTEAPSKPITVTVEEQR	3	0.9822	1
						CAPGYYGNPSQGQPCQR	2	0.9997	1
						CEQCQPGYYGDAQR	2	0.9997	1
						CLCLPGFSGPR	2	0.9098	1
						CRPVNQEIVR	2	0.996	2
						CSATGSPAPTIHWSK	2	0.9981	2
						DFISLGLQDGHLVFR	3	0.8136	1
						EHLLMALAGIDTLLIR	3	0.9896	2
						EVSEAVVDTLESEYLK	2	0.9993	2
						LEGDTLIIPR	2	0.9994	1
						LLSGPYFWSLPSR	2	0.9984	3

						RGSIQVDGEELVSGR	2	0.9964	2
						RYQLGSGEAR	2	0.9124	1
						SIVPQGGSHSLR	2	0.9979	1
						SLPEVPETIELEVR	2	0.9994	3
						TCESLGAGGYR	2	0.9959	1
						TPSGLYLGTCER	2	0.9914	2
						YELGSGLAVLR	2	0.9695	1
Q$875-1	P98164	LRP2_HUMAN	LRP2	Low-density lipoprotein receptor-related protein 2	1.0000	0.92	3	3	5
						IFWPCGLTIDYPNR	2	0.9573	1
						NLYWTDYALETIEVSK	2	0.9995	3
						TVMSLDYDSVSDR	2	0.995	1
Q$876-1	Q00577	PURA_HUMAN	PURA	Transcriptional activator protein Pur-alpha	0.9044	2.8	1	1	2
						FFFDVGSNK	2	0.9044	2
Q$877-1	Q00610	CLH1_HUMAN	CLTC	Clathrin heavy chain 1	1.0000	13.43	15	15	25
Q$877-2	Q00610-2	CLH1_HUMAN	CLTC	Isoform 2 of Clathrin heavy chain 1	1.0000	13.73	15	15	25
						CNEPAVWSQLAK	2	0.9732	1
						ESYVETELIFALAK	2	0.9997	2
						FQEHLQLQNLGINPANIGFSTLTMESDKFICIR	4	0.8683	1
						GQCDLELINVCNENSLFK	2	0.9519	1
						HDVVFLITK	2	0.9989	2
						HSSLAGCQIINYR	2	0.9779	1
						ISGETIFVTAPHEATAGIIGVNR	3	0.9988	2
						KFDVNTSAVQVLIEHIGNLDR	3	0.9997	3
						LEKHELIEFR	3	0.8668	1
						LLLPWLEAR	2	0.9975	2
						NLQNLLILTAIK	2	0.9996	2
						NNLAGAEELFAR	2	0.9978	2
						TLQIFNIEMK	2	0.9701	2
						TSIDAYDNFDNISLAQR	2	0.999	1
						VGYTPDWIFLLR	2	0.9992	2
Q$878-1	Q01082-3	SPTB2_HUMAN	SPTBN1	Isoform 2 of Spectrin beta chain, non-erythrocytic 1	1.0000	2.51	4	4	8
Q$878-2	Q01082-2	SPTB2_HUMAN	SPTBN1	Isoform Short of Spectrin beta chain, non-erythrocytic 1	1.0000	2.49	4	4	8
Q$878-3	Q01082	SPTB2_HUMAN	SPTBN1	Spectrin beta chain, non-erythrocytic 1	1.0000	2.28	4	4	8
						DVEDEILWVGER	2	0.9983	2
						EVDDLEQWIAER	2	0.9991	2
						HQILEQAVEDYAETVHQLSK	3	0.9992	3
						LTTLELLEVR	2	0.9973	1
Q$879-1	Q01105-2	SET_HUMAN	SET	Isoform 2 of Protein SET	1.0000	29.5	6	6	17
Q$879-2	Q01105-3	SET_HUMAN	SET	Isoform 3 of Protein SET	1.0000	30.83	6	6	17
Q$879-3	Q01105-4	SET_HUMAN	SET	Isoform 4 of Protein SET	1.0000	30.48	6	6	17
Q$879-4	Q01105	SET_HUMAN	SET	Protein SET	1.0000	28.28	6	6	17
						EFHLNESGDPSSK	2	0.9994	2
						IDFYFDENPYFENK	2	0.9997	3
						IPNFWVTTFVNHPQVSALLGEEDEEALHYLTR	4	0.9974	3
						LRQPFFQKR	3	0.9708	1
						SGYRIDFYFDENPYFENK	3	0.9971	4
						VEVTEFEDIK	2	0.9997	4
Q$880-1	Q01518	CAP1_HUMAN	CAP1	Adenylyl cyclase-associated protein 1	1.0000	20.84	8	8	14
Q$880-2	Q01518-2	CAP1_HUMAN	CAP1	Isoform 2 of Adenylyl cyclase-associated protein 1	1.0000	20.89	8	8	14
						ALLVTASQCQQPAENK	2	0.9997	3
						CVNTTLQIK	2	0.9544	1
						DVVGIVEIINSK	2	0.9696	2
						LGLVFDDVVGIVEIINSK	3	0.9989	1
						LSDLLAPISEQIKEVITFR	3	0.9911	2
						NSLDCEIVSAK	2	0.9976	2
						VENQENVSNLVIEDTELK	2	0.9996	2
						VPTISINK	2	0.9823	1
Q$881-1	Q01813	PFKAP_HUMAN	PFKP	ATP-dependent 6-phosphofructokinase, platelet type	1.0000	4.34	3	3	4
Q$881-2	Q01813-2	PFKAP_HUMAN	PFKP	Isoform 2 of ATP-dependent 6-phosphofructokinase, platelet	1.0000	4.38	3	3	4
						DLQSNVEHLTEK	2	0.9994	2
						MLAIYDGFDGFAK	2	0.9941	1
						VTILGHVQR	2	0.9978	1
Q$882-1	Q01814	AT2B2_HUMAN	ATP2B2	Plasma membrane calcium-transporting ATPase 2	0.9931	3.22	2	2	2
						GLNRIQTQIR	3	0.9212	1
						KGDGLQLPAADGAAASNAADSANASLVNGK	3	0.912	1
Q$883-1	Q02487	DSC2_HUMAN	DSC2	Desmocollin-2	0.9999	3.66	2	2	3
Q$883-2	Q02487-2	DSC2_HUMAN	DSC2	Isoform 2B of Desmocollin-2	0.9999	3.9	2	2	3
						TSYVTSVEENTVDVEILR	2	0.999	2
						VTVEDKDLVNTANWR	3	0.9439	1
Q$884-1	Q02763	TIE2_HUMAN	TEK	Angiopoietin-1 receptor	0.9708	1.78	2	2	2
Q$884-2	Q02763-2	TIE2_HUMAN	TEK	Isoform 2 of Angiopoietin-1 receptor	0.9708	1.85	2	2	2
Q$884-3	Q02763-3	TIE2_HUMAN	TEK	Isoform 3 of Angiopoietin-1 receptor	0.9708	2.05	2	2	2
						CICPPGFMGR	2	0.8247	1
						DGLRMDAAIK	2	0.8334	1
Q$885-1	Q02790	FKBP4_HUMAN	FKBP4	Peptidyl-prolyl cis-trans isomerase FKBP4	1.0000	5.88	2	2	3
						AWDIAIATMK	2	0.9942	1
						FEIGEGENLDLPYGLER	2	0.9988	2
Q$886-1	Q04695	K1C17_HUMAN	KRT17	Keratin, type I cytoskeletal 17	1.0000	40.28	14	16	29
						ADLEMQIENLKEELAYLK	3	0.9467	1
						ADLEMQIENLKEELAYLKK	3	0.8421	1
						ALEEANTELEVK	2	0.9991	2
						ASLEGNLAETENR	2	0.9996	3
						CEMEQQNQEYK	2	0.9997	2
						DAEDWFFSKTEELNR	2	0.9953	2
						DYSQYYR	2	0.9026	1
						EVATNSELVQSGK	2	0.9997	2
						ILNEMRDQYEK	2	0.9902	1
						ILTATVDNANILLQIDNAR	2	0.9995	3
						ILTATVDNANILLQIDNAR	3	0.999	2
						LSVEADINGLR	2	0.9783	1
						NHEEEMNALR	2	0.9996	2
						TKFETEQALR	2	0.9992	2
						TKFETEQALR	3	0.9955	1
						YCVQLSQIQGLIGSVEEQLAQLR	3	0.9994	3
Q$887-1	Q04828	AK1C1_HUMAN	AKR1C1	Aldo-keto reductase family 1 member C1	1.0000	38.39	9	10	22
						HIDSAHLYNNEEQVGLAIR	2	0.9997	3
						HIDSAHLYNNEEQVGLAIR	3	0.9996	5
						ILFDTVDLCATWEAVEK	2	0.9983	1
						LAIEAGFR	2	0.9995	2
						NLQLDYVDLYLIHFPVSVKPGEEVIPK	3	0.9978	2
						QNVQVFEFQLTSEEMK	2	0.9997	3
						REDIFYTSK	2	0.8377	1
						TPALIALR	2	0.9995	2
						TPALIALRY	2	0.9522	1
						YLTLDIFAGPPNYPFSDEY	2	0.9953	2
Q$888-1	Q04917	1433F_HUMAN	YWHAH	14-3-3 protein eta	1.0000	4.47	1	1	1
						YLAEVASGEKK	2	0.8953	1
Q$889-1	Q05519-2	SRS11_HUMAN	SRSF11	Isoform 2 of Serine/arginine-rich splicing factor 11	1.0000	5.18	2	2	3
Q$889-2	Q05519	SRS11_HUMAN	SRSF11	Serine/arginine-rich splicing factor 11	1.0000	5.17	2	2	3
Q$889-3	Q5T760	Q5T760_HUMAN	SRSF11	Serine/arginine-rich-splicing factor 11 (Fragment)	1.0000	6.43	2	2	3
						ALIVVPYAEGVIPDEAK	2	0.9993	2
						TLFGFLGK	2	0.9783	1
Q$890-1	Q06210	GFPT1_HUMAN	GFPT1	Glutamine--fructose-6-phosphate aminotransferase [isomeri	1.0000	5.01	2	2	4
Q$890-2	Q06210-2	GFPT1_HUMAN	GFPT1	Isoform 2 of Glutamine--fructose-6-phosphate aminotransfe	1.0000	5.14	2	2	4
						AVEYYFASDASAVIEHTNR	3	0.9877	1
						VIFLEDDDVAAVVDGR	2	0.9997	3
Q$891-1	Q06481	APLP2_HUMAN	APLP2	Amyloid-like protein 2	1.0000	13.5	9	10	14
Q$891-2	Q06481-3	APLP2_HUMAN	APLP2	Isoform 3 of Amyloid-like protein 2	1.0000	13.72	9	10	14
Q$891-3	Q06481-6	APLP2_HUMAN	APLP2	Isoform 6 of Amyloid-like protein 2	1.0000	13.53	9	10	14
						CLVGEFVSDVLLVPEK	2	0.9995	3
						CLVGEFVSDVLLVPEK	3	0.9961	1
						FIYGGCGGNR	2	0.9844	1
						LNMHVNIQTGK	2	0.9994	2

						LNMHVNIQTGKWEPDPTGTK	3	0.9986	2
						NNFESEDYCMAVCK	2	0.9997	1
						VEAMLNDR	2	0.9958	1
						VPYVAQEIQEEIDELLQEQR	2	0.995	1
						VSIDNWCR	2	0.9985	1
						WYFDLSK	2	0.9974	1
Q$892-1	Q06830	PRDX1_HUMAN	PRDX1	Peroxiredoxin-1	1.0000	9.55	2	2	3
						GLFIIDDK	2	0.9957	1
						QITVNDLPVGR	2	0.9982	2
Q$893-1	Q06945	SOX4_HUMAN	SOX4	Transcription factor SOX-4	0.9999	2.32	1	1	1
						LILAGGGGGK	2	0.9785	1
Q$894-1	Q07507	DERM_HUMAN	DPT	Dermatopontin	0.9729	8.46	1	1	1
						QGFSYQCPQGQVIVAVR	2	0.9729	1
Q$895-1	Q08211	DHX9_HUMAN	DHX9	ATP-dependent RNA helicase A	0.9993	0.94	2	2	6
Q$895-2	Q08211-2	DHX9_HUMAN	DHX9	Isoform 2 of ATP-dependent RNA helicase A	0.9993	5.11	2	2	6
						GAYGTGYFGQGR	2	0.964	1
						YGTGYFGQGR	2	0.9867	5
Q$896-1	Q08380	LG3BP_HUMAN	LGALS3BP	Galectin-3-binding protein	1.0000	9.4	3	4	9
						ALMLCEGLFVADVTDFEGWK	2	0.9827	1
						ELSEALGQIFDSQR	2	0.999	2
						GQWGTVCDNLWDLTDASVVCR	2	0.9997	4
						GQWGTVCDNLWDLTDASVVCR	3	0.9969	2
Q$897-1	Q12882	DPYD_HUMAN	DPYD	Dihydropyrimidine dehydrogenase [NADP(+)]	0.9457	0.88	1	1	1
						KNDWTELAK	2	0.9103	1
Q$898-1	Q12904	AIMP1_HUMAN	AIMP1	Aminoacyl tRNA synthase complex-interacting multifunctiona	1.0000	10.26	3	3	4
Q$898-2	Q12904-2	AIMP1_HUMAN	AIMP1	Isoform 2 of Aminoacyl tRNA synthase complex-interacting m	1.0000	9.52	3	3	4
						GAEADQIIEYLK	2	0.9996	2
						IGCIITAR	2	0.9987	1
						MVILLCNLKPAK	3	0.9847	1
Q$899-1	Q12923-3	PTN13_HUMAN	PTPN13	Isoform 3 of Tyrosine-protein phosphatase non-receptor type	0.9909	1.54	2	2	2
Q$899-2	Q12923-4	PTN13_HUMAN	PTPN13	Isoform 4 of Tyrosine-protein phosphatase non-receptor type	0.9909	1.53	2	2	2
Q$899-3	Q12923	PTN13_HUMAN	PTPN13	Tyrosine-protein phosphatase non-receptor type 13	0.9909	1.53	2	2	2
						GDSDMDEATYSSSQDHQTPK	2	0.9333	1
						RLSCSELSLYQPLQNSSK	2	0.8631	1
Q$900-1	Q13103	SPP24_HUMAN	SPP2	Secreted phosphoprotein 24	1.0000	9.95	2	2	3
						DALSASVVK	2	0.9825	1
						VNSQSLSPYLFR	2	0.9997	2
Q$901-1	Q13151	ROA0_HUMAN	HNRNPA0	Heterogeneous nuclear ribonucleoprotein A0	0.9816	5.25	1	1	3
						LFIGGLNVQTSESGLR	2	0.9816	3
Q$902-1	Q13247-3	SRSF6_HUMAN	SRSF6	Isoform SRP55-3 of Serine/arginine-rich splicing factor 6	1.0000	9.55	4	4	5
Q$902-2	Q13247	SRSF6_HUMAN	SRSF6	Serine/arginine-rich splicing factor 6	1.0000	9.3	4	4	5
						CSWQDLK	2	0.993	1
						ILEVDLK	2	0.9731	1
						LIVENLSSR	2	0.9965	1
						TNEGVIEFR	2	0.9929	2
Q$903-1	Q13268	DHRS2_HUMAN	DHRS2	Dehydrogenase/reductase SDR family member 2, mitochond	1.0000	36.07	9	11	14
						AGIVCHVGK	2	0.886	1
						LQGEGLSVAGIVCHVGK	2	0.9979	1
						LQGEGLSVAGIVCHVGK	3	0.9921	1
						LSVAGIVCHVGK	2	0.9442	1
						SAGVNPLVGSTLGTSEQIWDK	2	0.9922	1
						SPALLLSQLLPYMENRR	3	0.9954	2
						TLALELAPK	2	0.9656	1
						VAVVTGSTSGIGFAIAR	2	0.9996	3
						VFHGNESLWK	2	0.9827	1
						VFHGNESLWK	3	0.8937	1
						VNCVVPGIIK	2	0.9975	1
Q$904-1	Q13347	EIF3I_HUMAN	EIF3I	Eukaryotic translation initiation factor 3 subunit I	1.0000	11.08	3	3	6
						FFHLAFEEEFGR	3	0.9995	3
						IHYFDPQYFEFEFEA	2	0.9992	2
						SGEVLVNVK	2	0.9973	1
Q$905-1	Q13509	TBB3_HUMAN	TUBB3	Tubulin beta-3 chain	1.0000	15.11	4	4	8
						ALTVPELTQQMFDAK	2	0.9994	4
						FWEVISDEHGIDPSGNYVGDSDLQLER	3	0.9989	2
						MSSTFIGNSTAIQELFK	2	0.9986	1
						YLTVATVFR	2	0.992	1
Q$906-1	Q13510-3	ASAH1_HUMAN	ASAH1	Isoform 3 of Acid ceramidase	1.0000	19.02	6	6	11
						ESLDVYELDAK	2	0.9992	1
						GQFETYLR	2	0.9932	1
						LTVYTTLIDVTK	2	0.9997	3
						TSQENISFETMYDVLSTKPVLNK	3	0.9988	3
						WKHPFFLDDR	3	0.9442	2
						WYVVQTNYDR	2	0.9938	1
Q$907-1	Q13882-2	PTK6_HUMAN	PTK6	Isoform 2 of Protein-tyrosine kinase 6	0.9993	20.15	3	3	3
Q$907-2	Q13882	PTK6_HUMAN	PTK6	Protein-tyrosine kinase 6	0.9993	5.99	3	3	3
						AGDVFHVAR	2	0.9756	1
						SRTDEELSFR	2	0.9647	1
						YVGLWDFK	2	0.9697	1
Q$908-1	Q13885	TBB2A_HUMAN	TUBB2A	Tubulin beta-2A chain	1.0000	26.07	7	7	12
						ALTVPELTQQMFDSK	2	0.9952	3
						ESESCDCLQGFQLTHSLGGGTGSGMGTLLISK	3	0.9946	1
						FWEVISDEHGIDPTGSYHGDSDLQLER	3	0.9981	3
						INVYYNEAAGNK	2	0.9996	1
						INVYYNEAAGNKYVPR	3	0.8469	1
						MSATFIGNSTAIQELFK	2	0.8786	1
						YLTVAAIFR	2	0.9989	2
Q$909-1	Q13938-3	CAYP1_HUMAN	CAPS	Isoform 2 of Calcyphosin	1.0000	37.04	5	5	8
						EAVIAAAFAK	2	0.9987	1
						GASGIQGLAR	2	0.9979	1
						LGLVLDQAEAEGVCR	2	0.9997	2
						NGSGTLDLEEFLR	2	0.9993	3
						SGDGVVTVDDLR	2	0.9981	1
Q$910-1	Q14117	DPYS_HUMAN	DPYS	Dihydropyrimidinase	0.9991	2.31	1	1	2
						GGSLIEAFETWR	2	0.9504	2
Q$911-1	Q14166	TTL12_HUMAN	TTLL12	Tubulin--tyrosine ligase-like protein 12	1.0000	4.66	2	2	3
						DLDTGEEVTR	2	0.8342	1
						FTLTQSEADADILFNFSHFK	3	0.9938	2
Q$912-1	Q14204	DYHC1_HUMAN	DYNC1H1	Cytoplasmic dynein 1 heavy chain 1	1.0000	5.32	18	18	22
						EGTEAWEAAMK	2	0.9135	1
						FGNPLLVQDVESYDPVLNPVLNR	3	0.9708	2
						FNYGFEYLGVQDK	2	0.9986	1
						GIFEALRPLETLPVEGLIR	3	0.8999	1
						IQGLTVEQAEAVVR	2	0.9997	2
						KLVPLLLEDGGEAPAALEAALEEK	3	0.9215	1
						LALESICLLLGESTTDWK	2	0.9406	1
						LLNTFLER	2	0.9648	1
						LVPLLLEDGGEAPAALEAALEEK	3	0.8355	1
						LVQTPLTDR	2	0.9879	1
						REEFVQWVELLPDTQTPSWLGLPNNAER	3	0.9425	1
						TEYLSNADER	2	0.993	1
						TTDLLTDWEK	2	0.9817	1
						VQVALEELQDLK	2	0.9976	1
						VTFVNFTVTR	2	0.9992	2
						WAIAQLNYADMLK	2	0.9853	1
						YATLATVSR	2	0.9972	2
						YKEEYAVLISEAQAIK	3	0.9457	1
Q$913-1	Q14210	LY6D_HUMAN	LY6D	Lymphocyte antigen 6D	1.0000	11.72	1	1	2
						TTNTVEPLRGNLVKK	2	0.9855	2
Q$914-1	Q14258	TRI25_HUMAN	TRIM25	E3 ubiquitin/ISG15 ligase TRIM25	0.9706	3.02	1	1	1
						TCSPASLSQASADLEATLR	2	0.9706	1
Q$915-1	Q14314	FGL2_HUMAN	FGL2	Fibroleukin	1.0000	29.16	12	14	26
						AGFGNLR	2	0.9978	1
						AKDVCPVRLESR	2	0.9792	2

						CPSQEQIQSRPVQHLIYK	3	0.9535	2
						DCSDYYAIGKR	2	0.9995	1
						DVCPVRLESR	2	0.8333	1
						ELESEVNKLSSELK	2	0.9994	2
						GKCEEAGECPYQVSLPPLTIQLPK	3	0.9986	3
						IEEVFKEVQNLK	2	0.9992	2
						IEEVFKEVQNLK	3	0.9889	2
						LNLVNMNNIENYVDSK	2	0.9997	3
						NAKEEINVLHGR	2	0.9994	3
						NAKEEINVLHGR	3	0.9994	2
						PVQHLIYK	2	0.8937	1
						VRELESEVNKLSSELK	3	0.9755	1
Q$916-1	Q14376	GALE_HUMAN	GALE	UDP-glucose 4-epimerase	1.0000	20.4	8	8	15
						DLCQADKTWNAVLLR	3	0.9921	2
						DYIHVVDLAK	2	0.9993	4
						EGDVAACYANPSLAQEELGWTAALGLDR	3	0.9947	1
						FFIEEMIR	2	0.995	1
						MCEDLWR	2	0.9734	1
						REGDVAACYANPSLAQEELGWTAALGLDR	3	0.9994	4
						SKFFIEEMIR	3	0.976	1
						YANPSLAQEELGWTAALGLDR	2	0.9181	1
Q$917-1	Q14520	HABP2_HUMAN	HABP2	Hyaluronan-binding protein 2	1.0000	7.32	3	3	5
Q$917-2	Q14520-2	HABP2_HUMAN	HABP2	Isoform 2 of Hyaluronan-binding protein 2	1.0000	7.68	3	3	5
						FCEIGSDDCYVGDGYSYR	2	0.9996	1
						GQCLITQSPPYYR	2	0.9942	3
						LIANTLCNSR	2	0.9897	1
Q$918-1	Q14624-4	ITIH4_HUMAN	ITIH4	Isoform 4 of Inter-alpha-trypsin inhibitor heavy chain H4	1.0000	11.48	7	7	22
						AGFSWIEVTFK	2	0.9994	4
						ANTVQEATFQMELPK	2	0.9996	4
						DQFNLIVFSTEATQWRPSLVPASAENVNK	3	0.9312	1
						ITFELVYEELLK	2	0.9995	3
						LGVYELLLK	2	0.9995	3
						RLDYQEGPPGVEISCWSVEL	2	0.9995	5
						RLGVYELLLK	3	0.9989	2
Q$919-1	Q14764	MVP_HUMAN	MVP	Major vault protein	1.0000	17.81	12	13	18
						AQALAIETEAELQR	2	0.9997	2
						AQQLAEVEVKK	2	0.9832	1
						DAQGLVLFDVTGQVR	2	0.9828	1
						ELELVYAR	2	0.9893	1
						ELLELEALSMAVESTGTAK	2	0.9582	1
						GPLEYVPSAK	2	0.9984	1
						HYCTVANPVSR	2	0.9798	1
						IEGEGSVLQAK	2	0.9857	2
						KEVEVVEIIQATIIR	2	0.9996	1
						KEVEVVEIIQATIIR	3	0.9917	2
						LAQDPFPLYPGEVLEK	2	0.9994	3
						QAIPLDENEGIYVQDVK	2	0.9969	1
						TAVFGFETSEAK	2	0.9879	1
Q$920-1	Q14929	ZN169_HUMAN	ZNF169	Zinc finger protein 169	0.9993	3.65	2	2	2
						EWKLLSSAQRTLYR	2	0.9865	1
						SSLFSHQK	2	0.949	1
Q$921-1	Q15075	EEA1_HUMAN	EEA1	Early endosome antigen 1	0.9995	2.27	3	3	3
						KLEADSLEVK	2	0.9449	1
						LSASETSLHR	2	0.9729	1
						LSLAQEDLISNR	2	0.9835	1
Q$922-1	Q15149	PLEC_HUMAN	PLEC	Plectin	0.9998	0.45	2	2	3
						IISLETYNLLR	2	0.9846	2
						LTAEDLFEAR	2	0.8906	1
Q$923-1	Q15149-2	PLEC_HUMAN	PLEC	Isoform 2 of Plectin	0.9998	0.24	1	1	2
Q$923-2	Q15149-3	PLEC_HUMAN	PLEC	Isoform 3 of Plectin	0.9998	0.24	1	1	2
Q$923-3	Q15149-4	PLEC_HUMAN	PLEC	Isoform 4 of Plectin	0.9998	0.24	1	1	2
Q$923-4	Q15149-5	PLEC_HUMAN	PLEC	Isoform 5 of Plectin	0.9998	0.24	1	1	2
Q$923-5	Q15149-6	PLEC_HUMAN	PLEC	Isoform 6 of Plectin	0.9998	0.24	1	1	2
Q$923-6	Q15149-7	PLEC_HUMAN	PLEC	Isoform 7 of Plectin	0.9998	0.24	1	1	2
Q$923-7	Q15149-8	PLEC_HUMAN	PLEC	Isoform 8 of Plectin	0.9998	0.24	1	1	2
Q$923-8	Q15149-9	PLEC_HUMAN	PLEC	Isoform 9 of Plectin	0.9998	0.24	1	1	2
						IISLETYNLLR	2	0.9846	2
Q$924-1	Q15185-2	TEBP_HUMAN	PTGES3	Isoform 2 of Prostaglandin E synthase 3	1.0000	28.35	3	4	17
						DYVFIEFCVEDSKDVNVNFEK	3	0.9931	4
						LTFSCLGGSDNFK	2	0.9997	5
						SKLTFSCLGGSDNFK	2	0.9995	5
						SKLTFSCLGGSDNFK	3	0.9942	3
Q$925-1	Q15185-3	TEBP_HUMAN	PTGES3	Isoform 3 of Prostaglandin E synthase 3	1.0000	27.69	4	5	21
Q$925-2	Q15185-4	TEBP_HUMAN	PTGES3	Isoform 4 of Prostaglandin E synthase 3	1.0000	25.9	4	5	21
Q$925-3	Q15185	TEBP_HUMAN	PTGES3	Prostaglandin E synthase 3	1.0000	22.5	4	5	21
						DYVFIEFCVEDSK	2	0.9993	4
						DYVFIEFCVEDSKDVNVNFEK	3	0.9931	4
						LTFSCLGGSDNFK	2	0.9997	5
						SKLTFSCLGGSDNFK	2	0.9995	5
						SKLTFSCLGGSDNFK	3	0.9942	3
Q$926-1	Q15323	K1H1_HUMAN	KRT31	Keratin, type I cuticular Ha1	1.0000	6.01	2	2	4
						DSLENTLTESEAR	2	0.9983	2
						QNQEYQVLLDVR	2	0.9907	2
Q$927-1	Q15365	PCBP1_HUMAN	PCBP1	Poly(rC)-binding protein 1	0.9995	6.18	2	2	3
						IANPVEGSSGR	2	0.8206	1
						INISEGNCPER	2	0.9946	2
Q$928-1	Q15485	FCN2_HUMAN	FCN2	Ficolin-2	1.0000	19.49	6	10	23
Q$928-2	Q15485-2	FCN2_HUMAN	FCN2	Isoform 2 of Ficolin-2	1.0000	22.18	6	10	23
						LGEFWLGNDNIHALTAQGTSELR	2	0.9997	2
						LGEFWLGNDNIHALTAQGTSELR	3	0.9997	4
						RVDGSVDFYR	2	0.9996	3
						RVDGSVDFYR	3	0.9947	1
						RVDGSVDFYRDWATYK	2	0.9881	2
						RVDGSVDFYRDWATYK	3	0.9678	1
						TCKDLLDR	2	0.9985	2
						VDGSVDFYR	2	0.9198	1
						VDLVDFEDNYQFAK	2	0.9998	6
						VDLVDFEDNYQFAK	3	0.9949	1
Q$929-1	Q16204	CCDC6_HUMAN	CCDC6	Coiled-coil domain-containing protein 6	0.9765	2.95	1	1	2
						AEQEEEFISNTLFK	2	0.9765	2
Q$930-1	Q16772	GSTA3_HUMAN	GSTA3	Glutathione S-transferase A3	1.0000	9.91	2	2	9
						AILNYIASK	2	0.9966	1
						WLLAAAGVEFEEK	2	0.9997	8
Q$931-1	Q6FI13	H2A2A_HUMAN	HIST2H2AA3	Histone H2A type 2-A	0.9960	22.31	1	1	1
Q$931-2	Q16777	H2A2C_HUMAN	HIST2H2AC	Histone H2A type 2-C	0.9960	22.48	1	1	1
						VGAGAPVYMAAVLEYLTAEILELAGNAAR	3	0.9931	1
Q$932-1	Q16787-3	LAMA3_HUMAN	LAMA3	Isoform 3 of Laminin subunit alpha-3	0.9994	0.95	2	2	2
Q$932-2	Q16787	LAMA3_HUMAN	LAMA3	Laminin subunit alpha-3	0.9994	0.93	2	2	2
						FGGSCQPCSCNSNGQLGSCHP	2	0.8996	1
						LHVEGPTCSR	2	0.9941	1
Q$933-1	Q562R1	ACTBL_HUMAN	ACTBL2	Beta-actin-like protein 2	1.0000	6.91	2	2	3
						IIAPPERK	2	0.9994	2
						VAPDEHPILLTEAPLNPK	3	0.9879	1
Q$934-1	Q58FF6	H90B4_HUMAN	HSP90AB4P	Putative heat shock protein HSP 90-beta 4	1.0000	3.96	1	1	1
						GFEVIYMSEPIDEYCVQQLK	3	0.8937	1
Q$935-1	Q5JR95	Q5JR95_HUMAN	RPS8	40S ribosomal protein S8	1.0000	22.87	4	5	8
						ADGYVLEGK	2	0.9985	1
						ELEFYLR	2	0.9757	1
						IIDVVYNASNNELVR	2	0.9997	2
						IIDVVYNASNNELVR	3	0.9908	2
						NCIVLIDSTPYR	2	0.999	2
Q$936-1	Q5JRR9	Q5JRR9_HUMAN	UBA1	Ubiquitin-like modifier-activating enzyme 1 (Fragment)	1.0000	4.44	1	1	2
Q$936-2	Q5JRS0	Q5JRS0_HUMAN	UBA1	Ubiquitin-like modifier-activating enzyme 1 (Fragment)	1.0000	4.23	1	1	2

Q$936-3	Q5JRS1	Q5JRS1_HUMAN	UBA1	Ubiquitin-like modifier-activating enzyme 1 (Fragment)	1.0000	6.94	1	1	2
Q$936-4	Q5JRS2	Q5JRS2_HUMAN	UBA1	Ubiquitin-like modifier-activating enzyme 1 (Fragment)	1.0000	5.13	1	1	2
Q$936-5	Q5JRS3	Q5JRS3_HUMAN	UBA1	Ubiquitin-like modifier-activating enzyme 1 (Fragment)	1.0000	6.15	1	1	2
						LQTSSVLVSGLR	2	0.9989	2
Q$937-1	Q5JV98	Q5JV98_HUMAN	STK24	Serine/threonine-protein kinase 24 12 kDa subunit (Fragmen	1.0000	29.19	4	5	11
						ADIWSLGITAIELAR	2	0.9997	3
						ADIWSLGITAIELAR	3	0.9996	2
						GAIYLAEEACPGISDTMVAQLVQR	3	0.9952	2
						SQACGGNLGSIEELR	2	0.9997	3
						VLFLIPK	2	0.9889	1
Q$938-1	Q5QPP4	Q5QPP4_HUMAN	GALE	UDP-glucose 4-epimerase (Fragment)	1.0000	26.78	7	7	14
						DLCQADKTWNAVLLR	3	0.9921	2
						DYIHVVDLAK	2	0.9993	4
						EGDVAACYANPSLAQEELGWTAALGLDR	3	0.9947	1
						FFIEEMIR	2	0.995	1
						REGDVAACYANPSLAQEELGWTAALGLDR	3	0.9994	4
						SKFFIEEMIR	3	0.976	1
						YANPSLAQEELGWTAALGLDR	2	0.9181	1
Q$939-1	Q5QPP9	Q5QPP9_HUMAN	GALE	UDP-glucose 4-epimerase (Fragment)	1.0000	36.11	3	3	9
						DYIHVVDLAK	2	0.9993	4
						EGDVAACYANPSLAQEELGWTAALGLDR	3	0.9947	1
						REGDVAACYANPSLAQEELGWTAALGLDR	3	0.9994	4
Q$940-1	Q5SQ12	Q5SQ12_HUMAN	PTGDS	Prostaglandin-H2 D-isomerase	1.0000	10	1	1	2
Q$940-2	Q5SQ11	Q5SQ11_HUMAN	PTGDS	Prostaglandin-H2 D-isomerase (Fragment)	1.0000	8.21	1	1	2
						TMLLQPAGSLGSYSYR	2	0.9973	2
Q$941-1	Q5STZ7	Q5STZ7_HUMAN	ABCF1	ATP-binding cassette sub-family F member 1	1.0000	10.5	2	2	2
						FGLESHAHTIQICK	3	0.9329	1
						GAVIVVSHDAR	2	0.9994	1
Q$942-1	Q5SZU1	Q5SZU1_HUMAN	PHGDH	D-3-phosphoglycerate dehydrogenase	1.0000	7.41	3	3	5
						AGTGVDNVDLEAATR	2	0.9994	2
						GGIVDEGALLR	2	0.999	1
						VTADVINAAEK	2	0.9972	2
Q$943-1	Q5T0R1	Q5T0R1_HUMAN	CAP1	Adenylyl cyclase-associated protein (Fragment)	1.0000	16.28	2	2	5
Q$943-2	Q5T0R2	Q5T0R2_HUMAN	CAP1	Adenylyl cyclase-associated protein (Fragment)	1.0000	16.83	2	2	5
Q$943-3	Q5T0R3	Q5T0R3_HUMAN	CAP1	Adenylyl cyclase-associated protein (Fragment)	1.0000	17.24	2	2	5
Q$943-4	Q5T0R4	Q5T0R4_HUMAN	CAP1	Adenylyl cyclase-associated protein (Fragment)	1.0000	17.41	2	2	5
Q$943-5	Q5T0R5	Q5T0R5_HUMAN	CAP1	Adenylyl cyclase-associated protein (Fragment)	1.0000	19.55	2	2	5
Q$943-6	Q5T0R6	Q5T0R6_HUMAN	CAP1	Adenylyl cyclase-associated protein (Fragment)	1.0000	19.89	2	2	5
Q$943-7	Q5T0R7	Q5T0R7_HUMAN	CAP1	Adenylyl cyclase-associated protein (Fragment)	1.0000	20.11	2	2	5
Q$943-8	Q5T0R9	Q5T0R9_HUMAN	CAP1	Adenylyl cyclase-associated protein (Fragment)	1.0000	13.36	2	2	5
						ALLVTASQCQQPAENK	2	0.9997	3
						LSDLLAPISEQIKEVITFR	3	0.9911	2
Q$944-1	Q5T4S7	UBR4_HUMAN	UBR4	E3 ubiquitin-protein ligase UBR4	0.9445	0.23	1	1	2
Q$944-2	Q5T4S7-2	UBR4_HUMAN	UBR4	Isoform 2 of E3 ubiquitin-protein ligase UBR4	0.9445	0.23	1	1	2
Q$944-3	Q5T4S7-3	UBR4_HUMAN	UBR4	Isoform 3 of E3 ubiquitin-protein ligase UBR4	0.9445	0.23	1	1	2
Q$944-4	Q5T4S7-4	UBR4_HUMAN	UBR4	Isoform 4 of E3 ubiquitin-protein ligase UBR4	0.9445	0.23	1	1	2
Q$944-5	Q5T4S7-5	UBR4_HUMAN	UBR4	Isoform 5 of E3 ubiquitin-protein ligase UBR4	0.9445	0.49	1	1	2
						QLLQLLTTYIVR	2	0.9445	2
Q$945-1	Q5T6L6	Q5T6L6_HUMAN	ASS1	Argininosuccinate synthase (Fragment)	1.0000	18.18	3	3	7
						FELSCYSLAPQIK	2	0.9997	3
						KVFIEDVSR	2	0.9952	2
						YLLGTSLARPCIAR	2	0.9971	2
Q$946-1	Q5T7D5	Q5T7D5_HUMAN	ALDOB	Fructose-bisphosphate aldolase	1.0000	14.8	2	2	5
						IADQCPSSLAIQENANALAR	2	0.9996	3
						LDQGGAPLAGTNK	2	0.9992	2
Q$947-1	Q5T8U3	Q5T8U3_HUMAN	RPL7A	60S ribosomal protein L7a (Fragment)	0.9999	6.81	1	1	2
						AGVNTVTTLVENK	2	0.999	2
Q$948-1	Q5T987	Q5T987_HUMAN	ITIH2	Inter-alpha-trypsin inhibitor heavy chain H2 (Fragment)	1.0000	12.24	2	2	4
						TEVNVLPGAK	2	0.9993	2
						VVNNSPQPQNVVFDVQIPK	2	0.9996	2
Q$949-1	Q5TCU6	Q5TCU6_HUMAN	TLN1	Talin-1	1.0000	3.5	6	6	10
Q$949-2	Q9Y490	TLN1_HUMAN	TLN1	Talin-1	1.0000	3.35	6	6	10
						ALEATTEHIR	2	0.999	2
						ALSTDPAAPNLK	2	0.847	1
						AVSSAIAQLLGEVAQGNENYAGIAAR	3	0.9902	2
						GLAGAVSELLR	2	0.9994	2
						LAQAAQSSVATITR	2	0.9992	2
						VLVQNAAGSQEK	2	0.9744	1
Q$950-1	Q9HCE6-2	ARGAL_HUMAN	ARHGEF10L	Isoform 2 of Rho guanine nucleotide exchange factor 10-like	1.0000	2.98	3	3	4
Q$950-2	Q9HCE6-3	ARGAL_HUMAN	ARHGEF10L	Isoform 3 of Rho guanine nucleotide exchange factor 10-like	1.0000	3	3	3	4
Q$950-3	Q9HCE6-4	ARGAL_HUMAN	ARHGEF10L	Isoform 4 of Rho guanine nucleotide exchange factor 10-like	1.0000	3.77	3	3	4
Q$950-4	Q5VXI4	Q5VXI4_HUMAN	ARHGEF10L	Rho guanine nucleotide exchange factor 10-like protein	1.0000	3.52	3	3	4
Q$950-5	Q9HCE6	ARGAL_HUMAN	ARHGEF10L	Rho guanine nucleotide exchange factor 10-like protein	1.0000	2.89	3	3	4
						EICSVAIISGGQGYR	2	0.9994	2
						NFGSALGSSGR	2	0.9838	1
						SPGLQPVLCLR	2	0.9969	1
Q$951-1	Q5VYL6	Q5VYL6_HUMAN	CFHR5	Complement factor H-related protein 5	1.0000	2.19	1	1	2
Q$951-2	Q9BXR6	FHR5_HUMAN	CFHR5	Complement factor H-related protein 5	1.0000	2.28	1	1	2
						ITCTEEGWSPTPK	2	0.9991	2
Q$952-1	Q66LE6	2ABD_HUMAN	PPP2R2D	Serine/threonine-protein phosphatase 2A 55 kDa regulatory	1.0000	2.43	1	1	2
						INLWHLEITDR	2	0.9972	2
Q$953-1	Q6IS14	IF5AL_HUMAN	EIF5AL1	Eukaryotic translation initiation factor 5A-1-like	1.0000	23.38	2	2	5
						VHLVGIDIFTGK	3	0.9992	2
						YDCGEEILITVLSAMTEEAAVAIK	3	0.9996	3
Q$954-1	Q6KB66-2	K2C80_HUMAN	KRT80	Isoform 2 of Keratin, type II cytoskeletal 80	1.0000	2.13	1	1	1
Q$954-2	Q6KB66-3	K2C80_HUMAN	KRT80	Isoform 3 of Keratin, type II cytoskeletal 80	1.0000	1.85	1	1	1
Q$954-3	Q6KB66	K2C80_HUMAN	KRT80	Keratin, type II cytoskeletal 80	1.0000	1.99	1	1	1
						KLVEGEEGR	2	0.8984	1
Q$955-1	Q6MZW2	FSTL4_HUMAN	FSTL4	Follistatin-related protein 4	1.0000	6.06	3	3	7
Q$955-2	Q6MZW2-2	FSTL4_HUMAN	FSTL4	Isoform 2 of Follistatin-related protein 4	1.0000	7.58	3	3	7
						SFTESNQYNIYAALHTEPDLLFLELSTGK	3	0.9984	3
						VLVVDIQAQK	2	0.9961	2
						YEDTGAYTCIAK	2	0.9997	2
Q$956-1	Q6P9A2	GLT18_HUMAN	GALNT18	Polypeptide N-acetylgalactosaminyltransferase 18	0.9705	1.98	1	1	3
						SPALIGCFIVDR	2	0.9705	3
Q$957-1	Q6PCB0	VWA1_HUMAN	VWA1	von Willebrand factor A domain-containing protein 1	1.0000	15.73	5	6	7
						EFVGQLVAPLPLGTGALR	2	0.9926	1
						EQLFAEASGARPGVPK	2	0.9951	1
						GNFLELSAAASAPAEK	2	0.9977	1
						HLHFVDVDDLHIIVQELR	3	0.9786	1
						HLHFVDVDDLHIIVQELR	4	0.9864	2
						VREFVGQLVAPLPLGTGALR	3	0.9076	1
Q$958-1	Q6ZNA5	FRRS1_HUMAN	FRRS1	Ferric-chelate reductase 1	0.9018	6.25	1	1	1
Q$958-2	Q6ZNA5-2	FRRS1_HUMAN	FRRS1	Isoform 2 of Ferric-chelate reductase 1	0.9018	5.91	1	1	1
						NAEDLNGPPIGSFTLIDSEVSQLLTCEDIQGSAVSHR	3	0.9018	1
Q$959-1	Q709C8-2	VP13C_HUMAN	VPS13C	Isoform 2 of Vacuolar protein sorting-associated protein 13C	0.9728	0.3	1	1	1
Q$959-2	Q709C8-3	VP13C_HUMAN	VPS13C	Isoform 3 of Vacuolar protein sorting-associated protein 13C	0.9728	0.3	1	1	1
Q$959-3	Q709C8-4	VP13C_HUMAN	VPS13C	Isoform 4 of Vacuolar protein sorting-associated protein 13C	0.9728	0.31	1	1	1
Q$959-4	Q709C8	VP13C_HUMAN	VPS13C	Vacuolar protein sorting-associated protein 13C	0.9728	0.29	1	1	1
						TLDVFNIILAR	2	0.9728	1
Q$960-1	Q70CQ4	UBP31_HUMAN	USP31	Ubiquitin carboxyl-terminal hydrolase 31	0.9002	0.74	1	1	1
						LSSPTQTAAK	2	0.8244	1
Q$961-1	Q7L5L3	GDPD3_HUMAN	GDPD3	Glycerophosphodiester phosphodiesterase domain-containin	1.0000	8.18	2	2	2
Q$961-2	Q7L5L3-1	GDPD3_HUMAN	GDPD3	Isoform 2 of Glycerophosphodiester phosphodiesterase dom	1.0000	10.16	2	2	2
						DVGSLDFEDLPLYK	2	0.9991	1
						SDLLELDCQLTR	2	0.9942	1
Q$962-1	Q7Z5L0	VMO1_HUMAN	VMO1	Vitelline membrane outer layer protein 1 homolog	1.0000	54.95	9	9	48
						CSDGEELQGPGLSWGDFGDWSDHCPK	3	0.9995	11
						GGAYLVAFSLR	2	0.9997	5
						GLGDDTALNDAR	2	0.9997	8
						GNVLGNTHVVE	2	0.8715	1
						GNVLGNTHVVESQ	2	0.9839	2
						GNVLGNTHVVESQSG	2	0.8509	1

						GNVLGNTHVVESQSGSWGEWSEPLWCR	3	0.9997	8
						VEAPTTLGDNTAANNVR	2	0.9997	4
						VEPPQGIPGDDTALNGIR	2	0.9991	8
Q$963-1	Q7Z794	K2C1B_HUMAN	KRT77	Keratin, type II cytoskeletal 1b	1.0000	4.33	1	1	1
						RVYSTSSSAGSGGGSPAVGSVCYAR	2	0.9637	1
Q$964-1	Q86UU0	BCL9L_HUMAN	BCL9L	B-cell CLL/lymphoma 9-like protein	0.9126	0.6	1	1	1
Q$964-2	Q86UU0-2	BCL9L_HUMAN	BCL9L	Isoform 2 of B-cell CLL/lymphoma 9-like protein	0.9126	0.6	1	1	1
Q$964-3	Q86UU0-3	BCL9L_HUMAN	BCL9L	Isoform 3 of B-cell CLL/lymphoma 9-like protein	0.9126	0.65	1	1	1
Q$964-4	Q86UU0-4	BCL9L_HUMAN	BCL9L	Isoform 4 of B-cell CLL/lymphoma 9-like protein	0.9126	0.62	1	1	1
						GLLSPPMGQ	2	0.8579	1
Q$965-1	Q86VP6	CAND1_HUMAN	CAND1	Cullin-associated NEDD8-dissociated protein 1	1.0000	9.51	11	12	16
						AADIDQEVK	2	0.996	1
						AADIDQEVKER	2	0.9589	1
						ADVFHAYLSLLK	2	0.9973	1
						ADVFHAYLSLLK	3	0.999	2
						FMATNDLMTELQK	2	0.9978	1
						ITSEALLVTQQLVK	2	0.9989	2
						LGTLSALDILIK	2	0.9981	1
						LTLIDPETLLPR	2	0.9917	2
						NCIGDFLK	2	0.981	1
						NGEVQNLAVK	2	0.9619	2
						SVILEAFSSPSEEVK	2	0.9986	1
						TLEDPDLNVR	2	0.9907	1
Q$966-1	Q86VQ1	GLCI1_HUMAN	GLCCI1	Glucocorticoid-induced transcript 1 protein	0.9623	6.22	1	1	1
						SAAGSPPAVAAAGSGNGAGGGGGVGCAPAAGAGR	3	0.8202	1
Q$967-1	Q8IYD1	ERF3B_HUMAN	GSPT2	Eukaryotic peptide chain release factor GTP-binding subunit E	1.0000	5.89	3	3	3
						ETWYLSWALDTNQEER	2	0.9474	1
						HFTILDAPGHK	3	0.998	1
						KGEFETGFEK	2	0.9855	1
Q$968-1	Q8N111	CEND_HUMAN	CEND1	Cell cycle exit and neuronal differentiation protein 1	0.9051	12.75	1	1	1
						GPGDGAEEDEAASGGPGGR	2	0.9051	1
Q$969-1	Q8N159	NAGS_HUMAN	NAGS	N-acetylglutamate synthase, mitochondrial	0.9973	3.18	2	2	2
						ATALMAVVLR	2	0.9579	1
						FVVSSSR	2	0.9349	1
Q$970-1	Q8N1G4	LRC47_HUMAN	LRRC47	Leucine-rich repeat-containing protein 47	1.0000	3.95	2	2	4
						ELLLTGPGLEER	2	0.9981	2
						TAATLATHELR	2	0.9931	2
Q$971-1	Q8N475	FSTL5_HUMAN	FSTL5	Follistatin-related protein 5	1.0000	1.3	1	1	1
Q$971-2	Q8N475-2	FSTL5_HUMAN	FSTL5	Isoform 2 of Follistatin-related protein 5	1.0000	1.3	1	1	1
Q$971-3	Q8N475-3	FSTL5_HUMAN	FSTL5	Isoform 3 of Follistatin-related protein 5	1.0000	1.31	1	1	1
						VIRVYPESQAR	2	0.8164	1
Q$972-1	Q8NCH7	Q8NCH7_HUMAN	ANGPTL2	Angiopoietin-like 2, isoform CRA_c	1.0000	41.88	7	9	14
						HDPGGWTVIQR	2	0.9781	1
						KVFAEYASFR	2	0.9975	1
						KVFAEYASFR	3	0.9943	2
						LDGSVNFFR	2	0.9986	2
						LLVTMEDWSGR	2	0.9996	2
						QGFGNIDGEYWLGLENIYWLTNQGNYK	3	0.9522	1
						RLDGSVNFFR	2	0.9979	2
						RLDGSVNFFR	3	0.9979	2
						YQDGVYWAEFR	2	0.9353	1
Q$973-1	Q8ND23	LR16B_HUMAN	LRRC16B	Leucine-rich repeat-containing protein 16B	0.9961	1.24	2	2	3
						LESVASEVSK	3	0.8017	1
						RAQSCDK	2	0.9801	2
Q$974-1	Q8NFC6	BD1L1_HUMAN	BOD1L1	Biorientation of chromosomes in cell division protein 1-like 1	0.9891	1.08	2	2	2
						RNENSEVDTSAGSGSAPSVLHQR	3	0.8848	1
						VKDISIDVERR	2	0.905	1
Q$975-1	Q8NFI4	F10A5_HUMAN	ST13P5	Putative protein FAM10A5	1.0000	6.23	2	2	5
						AIDLFTDAIK	2	0.9861	2
						AIEINPDSAQPYK	2	0.9995	3
Q$976-1	Q8TE77	SSH3_HUMAN	SSH3	Protein phosphatase Slingshot homolog 3	1.0000	6.37	3	3	4
						IFPHLYLGSEWNAANLEELQR	3	0.8843	1
						QLQIYQGILTASR	2	0.9967	2
						YLLVVSTR	2	0.9988	1
Q$977-1	Q8TE77-2	SSH3_HUMAN	SSH3	Isoform 2 of Protein phosphatase Slingshot homolog 3	1.0000	6.16	2	2	2
						IFPHLYLGSEWNAANLEELQR	3	0.8843	1
						YLLVVSTR	2	0.9988	1
Q$978-1	Q8WUM4-2	PDC6I_HUMAN	PDCD6IP	Isoform 2 of Programmed cell death 6-interacting protein	0.9996	1.26	1	1	3
Q$978-2	Q8WUM4	PDC6I_HUMAN	PDCD6IP	Programmed cell death 6-interacting protein	0.9996	1.27	1	1	3
						FYNELTEILVR	2	0.998	3
Q$979-1	Q8WXI4	ACO11_HUMAN	ACOT11	Acyl-coenzyme A thioesterase 11	1.0000	7.08	4	4	6
						GELSVGQLLK	2	0.9972	1
						RGETLCSGFCLWR	3	0.9927	1
						SVTLPTHR	2	0.9655	1
						WIDTTACLSAER	2	0.9993	3
Q$980-1	Q8WXI4-2	ACO11_HUMAN	ACOT11	Isoform 2 of Acyl-coenzyme A thioesterase 11	1.0000	11.11	6	6	9
						ACEQFLLDNR	2	0.9904	2
						DNWVLSSEISQVR	2	0.9986	1
						GELSVGQLLK	2	0.9972	1
						RGETLCSGFCLWR	3	0.9927	1
						SVTLPTHR	2	0.9655	1
						WIDTTACLSAER	2	0.9993	3
Q$981-1	Q8WXS5	CCG8_HUMAN	CACNG8	Voltage-dependent calcium channel gamma-8 subunit	0.9659	11.29	1	1	1
						DPSKGSVAAGLAGAGGGGGGAVGAFGGAAGGAGGGGGGGGGAGAERDR	3	0.9166	1
Q$982-1	Q8WZ42-10	TITIN_HUMAN	TTN	Isoform 10 of Titin	0.9949	0.07	2	2	2
Q$982-2	Q8WZ42-11	TITIN_HUMAN	TTN	Isoform 11 of Titin	0.9949	0.06	2	2	2
Q$982-3	Q8WZ42-12	TITIN_HUMAN	TTN	Isoform 12 of Titin	0.9949	0.06	2	2	2
Q$982-4	Q8WZ42-13	TITIN_HUMAN	TTN	Isoform 13 of Titin	0.9949	0.06	2	2	2
Q$982-5	Q8WZ42-2	TITIN_HUMAN	TTN	Isoform 2 of Titin	0.9949	0.06	2	2	2
Q$982-6	Q8WZ42-3	TITIN_HUMAN	TTN	Isoform 3 of Titin	0.9949	0.07	2	2	2
Q$982-7	Q8WZ42-4	TITIN_HUMAN	TTN	Isoform 4 of Titin	0.9949	0.06	2	2	2
Q$982-8	Q8WZ42-5	TITIN_HUMAN	TTN	Isoform 5 of Titin	0.9949	0.06	2	2	2
Q$982-9	Q8WZ42-7	TITIN_HUMAN	TTN	Isoform 7 of Titin	0.9949	0.06	2	2	2
Q$982-10	Q8WZ42-8	TITIN_HUMAN	TTN	Isoform 8 of Titin	0.9949	0.06	2	2	2
Q$982-11	Q8WZ42-9	TITIN_HUMAN	TTN	Isoform 9 of Titin	0.9949	0.07	2	2	2
Q$982-12	Q8WZ42	TITIN_HUMAN	TTN	Titin	0.9949	0.06	2	2	2
						KSAATSLEK	2	0.917	1
						LITTCECVVPN	2	0.8425	1
Q$983-1	Q8WZ75-2	ROBO4_HUMAN	ROBO4	Isoform 2 of Roundabout homolog 4	1.0000	3.64	3	3	5
Q$983-2	Q8WZ75	ROBO4_HUMAN	ROBO4	Roundabout homolog 4	1.0000	3.38	3	3	5
						LSVAVLR	2	0.9975	1
						LSVAVLREDFQIQPR	3	0.997	2
						VSIQEPQDYTEPVELLAVR	2	0.9992	2
Q$984-1	Q92499	DDX1_HUMAN	DDX1	ATP-dependent RNA helicase DDX1	0.9999	4.32	2	3	4
						ELLIIGGVAAR	2	0.9969	1
						FLVLDEADGLLSQGYSDFINR	2	0.9888	1
						FLVLDEADGLLSQGYSDFINR	3	0.9929	2
Q$985-1	Q92598-3	HS105_HUMAN	HSPH1	Isoform 3 of Heat shock protein 105 kDa	1.0000	3.43	2	2	5
						AGGIETIANEFSDR	2	0.9994	3
						GCALQCAILSPAFK	2	0.9991	2
Q$986-1	Q92598	HS105_HUMAN	HSPH1	Heat shock protein 105 kDa	1.0000	5.01	2	2	4
Q$986-2	Q92598-2	HS105_HUMAN	HSPH1	Isoform Beta of Heat shock protein 105 kDa	1.0000	5.28	2	2	4
						AGGIETIANEFSDR	2	0.9994	3
						LKETAENSLKKPVTDCVISVPSFFTDAER	3	0.851	1
Q$987-1	Q92688	AN32B_HUMAN	ANP32B	Acidic leucine-rich nuclear phosphoprotein 32 family membe	1.0000	26.69	5	6	11
Q$987-2	Q92688-2	AN32B_HUMAN	ANP32B	Isoform 2 of Acidic leucine-rich nuclear phosphoprotein 32 fa	1.0000	34.36	5	6	11
						ELVLDNCK	2	0.9745	1
						KLELSENR	2	0.99	2
						LELSENRI	2	0.9104	1
						SLDLFNCEVTNLNDYR	2	0.9997	4
						SLDLFNCEVTNLNDYR	3	0.9929	2
						SNDGKIEGLTAEFVNLEFLSLINVGLISVSNLPK	3	0.9679	1
Q$988-1	Q92820	GGH_HUMAN	GGH	Gamma-glutamyl hydrolase	1.0000	33.65	9	11	20

						KPIIGILMQK	2	0.9975	1
						KPIIGILMQK	3	0.9993	1
						LDLTEKDYEILFK	2	0.9988	2
						LDLTEKDYEILFK	3	0.9926	2
						MFQNFPTELLLSLAVEPLTANFHK	3	0.9791	1
						NLDGISHAPNAVK	2	0.9997	4
						RLDLTEKDYEILFK	3	0.8465	1
						SINGILFPGGSVDLR	2	0.9982	2
						TAFYLAEFFVNEAR	3	0.9996	3
						YLESAGAR	2	0.934	1
						YYIAASYVK	2	0.9997	2
Q$989-1	Q92882	OSTF1_HUMAN	OSTF1	Osteoclast-stimulating factor 1	1.0000	5.61	1	1	2
						GYADIVQLLLAK	2	0.9995	2
Q$990-1	Q93084-2	AT2A3_HUMAN	ATP2A3	Isoform SERCA3A of Sarcoplasmic/endoplasmic reticulum cal	1.0000	1.5	1	1	2
Q$990-2	Q93084-3	AT2A3_HUMAN	ATP2A3	Isoform SERCA3C of Sarcoplasmic/endoplasmic reticulum cal	1.0000	1.46	1	1	2
Q$990-3	Q93084-6	AT2A3_HUMAN	ATP2A3	Isoform SERCA3D of Sarcoplasmic/endoplasmic reticulum cal	1.0000	1.44	1	1	2
Q$990-4	Q93084-5	AT2A3_HUMAN	ATP2A3	Isoform SERCA3E of Sarcoplasmic/endoplasmic reticulum cal	1.0000	1.43	1	1	2
Q$990-5	Q93084-7	AT2A3_HUMAN	ATP2A3	Isoform SERCA3F of Sarcoplasmic/endoplasmic reticulum cal	1.0000	1.45	1	1	2
Q$990-6	Q93084-4	AT2A3_HUMAN	ATP2A3	Isoform SERCA3G of Sarcoplasmic/endoplasmic reticulum cal	1.0000	1.5	1	1	2
Q$990-7	Q93084	AT2A3_HUMAN	ATP2A3	Sarcoplasmic/endoplasmic reticulum calcium ATPase 3	1.0000	1.44	1	1	2
						VGEATETALTCLVEK	2	0.9992	2
Q$991-1	Q96A08	H2B1A_HUMAN	HIST1H2BA	Histone H2B type 1-A	1.0000	7.09	1	1	2
						LLLPGELAK	2	0.9986	2
Q$992-1	Q96B18	DACT3_HUMAN	DACT3	Dapper homolog 3	0.9921	5.41	2	2	3
						RGAGQPRTSPGGADGGPR	2	0.852	1
						RGPAPTLAAQAAGSCR	2	0.9465	2
Q$993-1	Q96BW5-2	PTER_HUMAN	PTER	Isoform 2 of Phosphotriesterase-related protein	0.9903	9.27	2	2	2
Q$993-2	Q96BW5	PTER_HUMAN	PTER	Phosphotriesterase-related protein	0.9903	8.02	2	2	2
						CGIIGEIGCSWPLTESER	2	0.8447	1
						LLVEEGCEDR	2	0.9373	1
Q$994-1	Q96DG6	CMBL_HUMAN	CMBL	Carboxymethylenebutenolidase homolog	1.0000	13.47	3	4	8
						AVIVIQDIFGWQLPNTR	2	0.9996	4
						AVIVIQDIFGWQLPNTR	3	0.9994	2
						EVQVEHIK	2	0.9938	1
						LEYGGLGR	2	0.9965	1
Q$995-1	Q96HJ9-2	CG055_HUMAN	C7orf55	Isoform 2 of UPF0562 protein C7orf55	1.0000	3.71	2	2	3
Q$995-2	Q9Y383-2	LC7L2_HUMAN	LUC7L2	Isoform 2 of Putative RNA-binding protein Luc7-like 2	1.0000	4.35	2	2	3
Q$995-3	Q9Y383-3	LC7L2_HUMAN	LUC7L2	Isoform 3 of Putative RNA-binding protein Luc7-like 2	1.0000	4.37	2	2	3
Q$995-4	Q9Y383	LC7L2_HUMAN	LUC7L2	Putative RNA-binding protein Luc7-like 2	1.0000	4.34	2	2	3
						VHDLALR	2	0.99	1
						VHELNEEIGK	2	0.9956	2
Q$996-1	Q96HR3-2	MED30_HUMAN	MED30	Isoform 2 of Mediator of RNA polymerase II transcription sub	0.9597	6.29	1	1	2
Q$996-2	Q96HR3	MED30_HUMAN	MED30	Mediator of RNA polymerase II transcription subunit 30	0.9597	5.06	1	1	2
						TMEIFQLLR	2	0.9597	2
Q$997-1	Q96IY4	CBPB2_HUMAN	CPB2	Carboxypeptidase B2	1.0000	7.57	3	3	5
						DTGTYGFLLPER	2	0.9835	3
						EAFAAVSK	2	0.8309	1
						NAIWIDCGIHAR	2	0.8319	1
Q$998-1	Q96KP4	CNDP2_HUMAN	CNDP2	Cytosolic non-specific dipeptidase	1.0000	36.42	11	12	19
						EGGSIPVTLTFQEATGK	2	0.9995	2
						FCLEGMEESGSEGLDELIFAR	2	0.9997	3
						FCLEGMEESGSEGLDELIFAR	3	0.9698	1
						GSTDDKGPVAGWINALEAYQK	3	0.992	2
						LYDDIDFDIEEFAK	2	0.9917	1
						MLAAYLYEVSQLKD	3	0.9906	1
						MMEVAAADVK	2	0.999	1
						QKLPDGSEIPLPPILLGR	3	0.9953	4
						TGQEIPVNVR	2	0.9962	1
						TVCIYGHLDVQPAALEDGWDSEPFTLVER	3	0.9869	1
						TVFGVEPDLTR	2	0.9976	1
						YNYIEGTK	2	0.9959	1
Q$999-1	Q96QE3	ATAD5_HUMAN	ATAD5	ATPase family AAA domain-containing protein 5	0.9965	1.46	2	2	2
						KVPPPSPKSSGPK	2	0.9004	1
						SEATDGGFTSQIRK	2	0.9649	1
Q$1000-1	Q96QK1	VPS35_HUMAN	VPS35	Vacuolar protein sorting-associated protein 35	1.0000	3.89	2	3	4
						AQLAAITLIIGTFER	2	0.9997	2
						LFDIFSQQVATVIQSR	2	0.9985	1
						LFDIFSQQVATVIQSR	3	0.9331	1
Q$1001-1	Q96TA1-2	NIBL1_HUMAN	FAM129B	Isoform 2 of Niban-like protein 1	1.0000	2.86	2	2	3
Q$1001-2	Q96TA1	NIBL1_HUMAN	FAM129B	Niban-like protein 1	1.0000	2.82	2	2	3
						FDVSSTSVFK	2	0.985	1
						FQELIFEDFAR	2	0.9996	2
Q$1002-1	Q99832-3	TCPH_HUMAN	CCT7	Isoform 3 of T-complex protein 1 subunit eta	1.0000	16.03	6	6	9
Q$1002-2	Q99832	TCPH_HUMAN	CCT7	T-complex protein 1 subunit eta	1.0000	14.73	6	6	9
						CQVFEETQIGGER	2	0.9996	2
						GGAEQFMEETER	2	0.9606	1
						IALLNVELELK	2	0.9824	1
						INALTAASEAACLIVSVDETIKNPR	3	0.9993	3
						LLDVVHPAAK	2	0.8255	1
						YNFFTGCPK	2	0.9076	1
Q$1003-1	Q99988	GDF15_HUMAN	GDF15	Growth/differentiation factor 15	1.0000	10.39	2	2	5
						ASLEDLGWADWVLSPR	2	0.9996	3
						TDTGVSLQTYDDLLAK	2	0.9996	2
Q$1004-1	Q9BR76	COR1B_HUMAN	CORO1B	Coronin-1B	1.0000	16.16	5	5	11
						AGEAGKLEEVMQELR	3	0.9974	2
						KCEPIVMTVPR	2	0.9732	2
						KSDLFQDDLYPDTAGPEAALEAEEWVSGR	3	0.9988	3
						VGIIAWHPTAR	3	0.9992	2
						VTWDSTFCAVNPK	2	0.9996	2
Q$1005-1	Q9BRK3-4	MXRA8_HUMAN	MXRA8	Isoform 4 of Matrix-remodeling-associated protein 8	1.0000	6.74	2	2	5
						HVEEAQQVVHWDR	3	0.9996	2
						VAVGADAFER	2	0.9986	3
Q$1006-1	Q9BRK3-2	MXRA8_HUMAN	MXRA8	Isoform 2 of Matrix-remodeling-associated protein 8	1.0000	28.89	13	17	30
Q$1006-2	Q9BRK3-3	MXRA8_HUMAN	MXRA8	Isoform 3 of Matrix-remodeling-associated protein 8	1.0000	30.02	13	17	30
Q$1006-3	Q9BRK3	MXRA8_HUMAN	MXRA8	Matrix-remodeling-associated protein 8	1.0000	29.41	13	17	30
						AYGPLFLR	2	0.9996	2
						DRVAVGADAFER	2	0.9602	2
						HVEEAQQVVHWDR	2	0.9997	3
						HVEEAQQVVHWDR	3	0.9996	2
						HVEEAQQVVHWDRQPPGVPHDR	3	0.832	1
						LEVTDGPPATPAYWDGEKEVLAVAR	3	0.9895	1
						LLDLYASGERR	2	0.9443	1
						LLDLYASGEQR	2	0.9997	4
						RLLDLYSAGEQR	2	0.9978	2
						RVFHLTVAEPHAEPPPR	3	0.9638	1
						RVFHLTVAEPHAEPPPR	4	0.8589	1
						SSVVSESAVSWEAGAR	2	0.8566	1
						VAVGADAFER	2	0.9986	3
						VFHLTVAEPHAEPPPR	2	0.9992	1
						VFHLTVAEPHAEPPPR	3	0.9992	3
						VFHLTVAEPHAEPPPR	4	0.8844	1
						VLHWDLR	2	0.9938	1
Q$1007-1	Q9BRL6-2	SRSF8_HUMAN	SRSF8	Isoform 2 of Serine/arginine-rich splicing factor 8	0.9991	2.91	1	1	1
Q$1007-2	Q9BRL6	SRSF8_HUMAN	SRSF8	Serine/arginine-rich splicing factor 8	0.9991	2.84	1	1	1
						VGDVYIPR	2	0.9789	1
Q$1008-1	Q9BT78-2	CSN4_HUMAN	COPS4	Isoform 2 of COP9 signalosome complex subunit 4	1.0000	9.09	2	3	4
						CQQLAAYGILEK	2	0.857	1
						LYNNITFEELGALLEIPAAK	2	0.9994	1
						LYNNITFEELGALLEIPAAK	3	0.999	2
Q$1009-1	Q9BTT0	AN32E_HUMAN	ANP32E	Acidic leucine-rich nuclear phosphoprotein 32 family membe	1.0000	13.06	2	2	3
Q$1009-2	Q9BTT0-3	AN32E_HUMAN	ANP32E	Isoform 3 of Acidic leucine-rich nuclear phosphoprotein 32 fa	1.0000	15.91	2	2	3
						KLELSDNIISGGLEVLAEK	3	0.9965	2
						SLDLFNCEITNLEDYR	2	0.9963	1

Q$1010-1	Q9BUF5	TBB6_HUMAN	TUBB6	Tubulin beta-6 chain	1.0000	18.83	6	6	12
						EIVHIQAGQCGNQIGTK	3	0.8277	1
						FWEVISDEHGIDPAGGYVGDSALQLER	3	0.999	4
						GHYTEGAELVDAVLDVVR	3	0.9989	3
						GHYTEGAELVDAVLDVVRK	3	0.9891	1
						INVYYNESSSQK	2	0.9995	2
						YLTVATVFR	2	0.992	1
Q$1011-1	Q9BUT1	BDH2_HUMAN	BDH2	3-hydroxybutyrate dehydrogenase type 2	1.0000	13.47	2	2	5
						SVAADFIQQGIR	2	0.9994	2
						VIILTAAAQGIGQAAALAFAR	3	0.9996	3
Q$1012-1	Q9BVA1	TBB2B_HUMAN	TUBB2B	Tubulin beta-2B chain	1.0000	18.65	4	4	9
						ALTVPELTQQMFDSK	2	0.9952	3
						ESESCDCLQGFQLTHSLGGGTGSGMGTLLISK	3	0.9946	1
						FWEVISDEHGIDPTGSYHGDSDLQLER	3	0.9981	3
						YLTVAAIFR	2	0.9989	2
Q$1013-1	Q9H223	EHD4_HUMAN	EHD4	EH domain-containing protein 4	1.0000	10.54	5	5	10
						AGGADAVQTVTGGLR	2	0.9996	3
						EYQISAGDFPEVK	2	0.9997	2
						FGNAFLNR	2	0.996	1
						LPEIYIQLQR	2	0.9873	1
						YLLEQDFPGMR	2	0.9995	3
Q$1014-1	Q9H3G5	CPVL_HUMAN	CPVL	Probable serine carboxypeptidase CPVL	1.0000	15.55	6	7	16
						CTEPEDQLYYVK	2	0.9991	4
						DLYSALIQFFQIFPEYK	2	0.9997	3
						DLYSALIQFFQIFPEYK	3	0.9996	3
						FLSLPEVR	2	0.9886	2
						GGGHILPYDQPLR	3	0.9969	1
						NNDFYVTGESYAGK	2	0.9997	2
						SDSEVAGYIR	2	0.9991	1
Q$1015-1	Q9H4A4	AMPB_HUMAN	RNPEP	Aminopeptidase B	1.0000	12.31	7	7	9
						AEFGPPGPGAGSR	2	0.9907	1
						AFELLHLHL	3	0.8938	1
						CLEPEGAAELR	2	0.8422	1
						GLSGTAVLDLR	2	0.9983	2
						ISTILFGAAYTCLEAATGR	3	0.9104	1
						LFGPYVWGR	2	0.9301	1
						LQVLLTYR	2	0.9681	2
Q$1016-1	Q9H4G0	E41L1_HUMAN	EPB41L1	Band 4.1-like protein 1	1.0000	6.7	3	3	5
Q$1016-2	Q9H4G0-2	E41L1_HUMAN	EPB41L1	Isoform 2 of Band 4.1-like protein 1	1.0000	7.57	3	3	5
Q$1016-3	Q9H4G0-4	E41L1_HUMAN	EPB41L1	Isoform 4 of Band 4.1-like protein 1	1.0000	8.42	3	3	5
						DVLTSTYGATAETLSTSTTTHVTK	3	0.9683	2
						IIITGDEDVDQDQALALAIK	2	0.9979	1
						VTLLDASEYECEVEK	2	0.9995	2
Q$1017-1	Q9H8L6	MMRN2_HUMAN	MMRN2	Multimerin-2	0.9631	1.37	1	1	3
						VWFELTQGSITKR	2	0.9631	3
Q$1018-1	Q9HAT2-2	SIAE_HUMAN	SIAE	Isoform 2 of Sialate O-acetylesterase	0.9999	5.94	2	2	2
Q$1018-2	Q9HAT2	SIAE_HUMAN	SIAE	Sialate O-acetylesterase	0.9999	5.54	2	2	2
						FFPFGLVQLSSDLSK	2	0.9945	1
						WHQTADFGYVPNPK	3	0.9886	1
Q$1019-1	Q9HB40	RISC_HUMAN	SCPEP1	Retinoid-inducible serine carboxypeptidase	1.0000	3.76	2	2	2
						EATELWGK	2	0.9457	1
						NLAFYWILK	2	0.9867	1
Q$1020-1	Q9HD89	RETN_HUMAN	RETN	Resistin	1.0000	24.07	3	3	7
						AISSIGLECQSVTSR	2	0.9997	2
						IQEVAGSLIFR	2	0.9996	3
						SSIGLECQSVTSR	2	0.9431	2
Q$1021-1	Q9NQT8	KI13B_HUMAN	KIF13B	Kinesin-like protein KIF13B	0.9999	1.2	2	2	4
						SVLAVENLLTLDR	2	0.9992	2
						YAGQDIVFK	2	0.9223	2
Q$1022-1	Q9NRX4	PHP14_HUMAN	PHPT1	14 kDa phosphohistidine phosphatase	1.0000	43.2	3	3	6
						AKYPDYEVTWANDGY	2	0.9983	2
						IHVYGYSMAYGPAQHAISTEK	3	0.9996	3
						WAEYHADIYDKVSGDMQK	3	0.9827	1
Q$1023-1	Q9NSB2	KRT84_HUMAN	KRT84	Keratin, type II cuticular Hb4	1.0000	1.33	1	1	1
						AQYEEVAR	2	0.982	1
Q$1024-1	Q9NZ45	CISD1_HUMAN	CISD1	CDGSH iron-sulfur domain-containing protein 1	0.9491	13.89	1	1	1
						HNEETGDNVGPLIIK	2	0.9491	1
Q$1025-1	Q9NZE6	Q9NZE6_HUMAN	EIF4A2	Eukaryotic initiation factor 4A-II	1.0000	3.21	1	1	3
						VLITTDLLAR	2	0.9995	3
Q$1026-1	Q9NZW4	DSPP_HUMAN	DSPP	Dentin sialophosphoprotein	0.9525	1.92	1	1	1
						SDSSDSSDSSDSTSDSNDESDSQSK	2	0.8143	1
Q$1027-1	Q9P258	RCC2_HUMAN	RCC2	Protein RCC2	1.0000	6.32	4	4	6
						AQRIEYDCELVPR	3	0.9224	1
						AVQDLCGWR	2	0.9971	1
						IEYDCELVPR	2	0.9978	2
						VFSWGFGGYGR	2	0.9962	2
Q$1028-1	Q9P260-2	K1468_HUMAN	KIAA1468	Isoform 2 of LisH domain and HEAT repeat-containing protei	1.0000	2.48	2	2	4
Q$1028-2	Q9P260	K1468_HUMAN	KIAA1468	LisH domain and HEAT repeat-containing protein KIAA1468	1.0000	2.55	2	2	4
						DQYLLTALELHTELLESGR	3	0.9727	2
						LSIDAIAAQLLR	2	0.9992	2
Q$1029-1	Q9UBF2	COPG2_HUMAN	COPG2	Coatomer subunit gamma-2	0.9999	1.38	1	1	2
						SIATLAITTLLK	2	0.9967	2
Q$1030-1	Q9UBQ7	GRHPR_HUMAN	GRHPR	Glyoxylate reductase/hydroxypyruvate reductase	1.0000	8.23	2	2	3
Q$1030-2	U3KQ56	U3KQ56_HUMAN	GRHPR	Glyoxylate reductase/hydroxypyruvate reductase	1.0000	7.54	2	2	3
						GDVVNQDDLYQALASGK	2	0.9994	2
						ILDAAGANLK	2	0.9845	1
Q$1031-1	Q9UBR2	CATZ_HUMAN	CTSZ	Cathepsin Z	1.0000	15.84	4	4	7
						NSWGEPWGER	2	0.9962	1
						NVDGVNYASITR	2	0.9993	2
						VGDYGSLSGR	2	0.9993	1
						YNLAIEEHCTFGDPIV	2	0.9995	3
Q$1032-1	Q9UDV7	ZN282_HUMAN	ZNF282	Zinc finger protein 282	0.9909	7	1	1	1
						GSGEAPPGGDRSTGGGGGDGGGGGGGAEAGTGAGGGCGSCCPGGLRR	3	0.9553	1
Q$1033-1	Q9UDY2-6	ZO2_HUMAN	TJP2	Isoform 6 of Tight junction protein ZO-2	1.0000	9.85	8	9	16
Q$1033-2	Q9UDY2-7	ZO2_HUMAN	TJP2	Isoform 7 of Tight junction protein ZO-2	1.0000	9.34	8	9	16
Q$1033-3	Q9UDY2-2	ZO2_HUMAN	TJP2	Isoform A2 of Tight junction protein ZO-2	1.0000	10.93	8	9	16
Q$1033-4	Q9UDY2-5	ZO2_HUMAN	TJP2	Isoform A3 of Tight junction protein ZO-2	1.0000	11.48	8	9	16
Q$1033-5	Q9UDY2-3	ZO2_HUMAN	TJP2	Isoform C1 of Tight junction protein ZO-2	1.0000	9.77	8	9	16
Q$1033-6	Q9UDY2-4	ZO2_HUMAN	TJP2	Isoform C2 of Tight junction protein ZO-2	1.0000	11.18	8	9	16
Q$1033-7	Q9UDY2	ZO2_HUMAN	TJP2	Tight junction protein ZO-2	1.0000	9.58	8	9	16
						EDAVLYLLEIPK	2	0.9996	2
						GDSFFIR	2	0.9584	1
						GLVREDAVLYLLEIPKGEMVTILAQSR	3	0.9388	1
						GLVREDAVLYLLEIPKGEMVTILAQSR	4	0.9885	3
						LAGGNDVGIFVAGIQEGTSAEQEGLQEGDQILK	3	0.9987	3
						LANELPDWFQTAK	2	0.9895	1
						LGNWLAVR	2	0.9964	2
						SHFECEKETPQSLAFTR	3	0.9958	2
						VVDTLYDGK	2	0.9764	1
Q$1034-1	Q9UEW8	STK39_HUMAN	STK39	STE20/SPS1-related proline-alanine-rich protein kinase	1.0000	9.17	3	3	7
						DAYELQEVIGSGATAVVQAALCKPR	3	0.9994	4
						LASGCDGSEIPDEVK	2	0.9986	2
						LIGFAQLSVS	2	0.9849	1
Q$1035-1	Q9UGI0	ZRAN1_HUMAN	ZRANB1	Ubiquitin thioesterase ZRANB1	0.9741	1.41	1	1	1
						GSCSSGNSQR	2	0.9549	1
Q$1036-1	Q9UGM5	FETUB_HUMAN	FETUB	Fetuin-B	0.9641	2.88	1	1	1
						IFFESVYGQCK	2	0.9641	1
Q$1037-1	Q9UGT4	SUSD2_HUMAN	SUSD2	Sushi domain-containing protein 2	0.9797	4.74	2	2	3
						CGALDGPCSCHPTCSGLGTCCLDFR	3	0.9509	2
						GEYVLLEAALTDLR	2	0.9797	1
Q$1038-1	Q9UHB9-2	SRP68_HUMAN	SRP68	Isoform 2 of Signal recognition particle subunit SRP68	1.0000	2.18	1	1	2
Q$1038-2	Q9UHB9-3	SRP68_HUMAN	SRP68	Isoform 3 of Signal recognition particle subunit SRP68	1.0000	4.51	1	1	2
						FETFCLDPSLVTK	2	0.9983	2

Q$1039-1	Q9UHB9-4	SRP68_HUMAN	SRP68	Isoform 4 of Signal recognition particle subunit SRP68	1.0000	10.19	3	3	4
Q$1039-2	Q9UHB9	SRP68_HUMAN	SRP68	Signal recognition particle subunit SRP68	1.0000	9.57	3	3	4
						FETFCLDPSLVTK	2	0.9983	2
						KQVPGGGGGGGSGGGGGSGGGGSGGGR	2	0.8463	1
						SGGTEGLLAEKLEALITQTR	3	0.9664	1
Q$1040-1	Q9UHD8-2	SEPT9_HUMAN	SEPT9	Isoform 2 of Septin-9	0.9994	3.87	1	1	2
Q$1040-2	Q9UHD8-5	SEPT9_HUMAN	SEPT9	Isoform 5 of Septin-9	0.9994	3.8	1	1	2
Q$1040-3	Q9UHD8-7	SEPT9_HUMAN	SEPT9	Isoform 7 of Septin-9	0.9994	3.88	1	1	2
Q$1040-4	Q9UHD8	SEPT9_HUMAN	SEPT9	Septin-9	0.9994	3.75	1	1	2
						TELSIDISSKQVENAGAIGPSR	2	0.9241	2
Q$1041-1	Q9UIB8-2	SLAF5_HUMAN	CD84	Isoform 2 of SLAM family member 5	1.0000	11.21	2	2	4
Q$1041-2	Q9UIB8-3	SLAF5_HUMAN	CD84	Isoform 3 of SLAM family member 5	1.0000	11.59	2	2	4
Q$1041-3	Q9UIB8-4	SLAF5_HUMAN	CD84	Isoform 4 of SLAM family member 5	1.0000	13.57	2	2	4
Q$1041-4	Q9UIB8-5	SLAF5_HUMAN	CD84	Isoform 5 of SLAM family member 5	1.0000	13.97	2	2	4
Q$1041-5	Q9UIB8-6	SLAF5_HUMAN	CD84	Isoform 6 of SLAM family member 5	1.0000	15.77	2	2	4
Q$1041-6	Q9UIB8	SLAF5_HUMAN	CD84	SLAM family member 5	1.0000	11.01	2	2	4
						IHALGPNYNLVISDLR	3	0.9991	2
						TSVAYVTPGDSETAPVVTVTHR	3	0.9941	2
Q$1042-1	Q9UJ70-2	NAGK_HUMAN	NAGK	Isoform 2 of N-acetyl-D-glucosamine kinase	1.0000	11.03	2	2	3
						DANGGTSSDGSSSMAAIYGGVEGGGTR	3	0.9273	1
						SLGLSLSGGDQEDAGR	2	0.9996	2
Q$1043-1	Q9UJ72	ANX10_HUMAN	ANXA10	Annexin A10	1.0000	7.72	2	2	5
						GVGTDENCLIEILASR	2	0.9991	3
						SEIDLLTIR	2	0.9965	2
Q$1044-1	Q9UKU9	ANGL2_HUMAN	ANGPTL2	Angiopoietin-related protein 2	1.0000	23.12	9	11	16
						DCLQALEDGHDTSSIYLVKPENTNR	3	0.9712	1
						HDPGGWTVIQR	2	0.9781	1
						KVFAEYASFR	2	0.9975	1
						KVFAEYASFR	3	0.9943	2
						LDGSVNFFR	2	0.9986	2
						LLVTMEDWSGR	2	0.9996	2
						LMQVWCDQR	2	0.9957	1
						QGFGNIDGEYWLGLENIYWLTNQGNYK	3	0.9522	1
						RLDGSVNFFR	2	0.9979	2
						RLDGSVNFFR	3	0.9979	2
						YQDGVYWAEFR	2	0.9353	1
Q$1045-1	Q9UL46	PSME2_HUMAN	PSME2	Proteasome activator complex subunit 2	0.9999	12.97	2	2	3
						AFYAELYHIISSNLEK	3	0.9687	1
						ALVHERDEAAYGELR	3	0.8949	2
Q$1046-1	Q9UN67	PCDBA_HUMAN	PCDHB10	Protocadherin beta-10	0.9472	1.63	1	1	1
Q$1046-2	Q9Y5E6	PCDB3_HUMAN	PCDHB3	Protocadherin beta-3	0.9472	1.63	1	1	1
Q$1046-3	Q9Y5E5	PCDB4_HUMAN	PCDHB4	Protocadherin beta-4	0.9472	1.64	1	1	1
Q$1046-4	Q9Y5E4	PCDB5_HUMAN	PCDHB5	Protocadherin beta-5	0.9472	1.64	1	1	1
Q$1046-5	Q9Y5E2	PCDB7_HUMAN	PCDHB7	Protocadherin beta-7	0.9472	1.64	1	1	1
Q$1046-6	Q9UN66	PCDB8_HUMAN	PCDHB8	Protocadherin beta-8	0.9472	1.62	1	1	1
						SLDYEALQAFEFR	2	0.9472	1
Q$1047-1	Q9UN70-2	PCDGK_HUMAN	PCDHGC3	Isoform 2 of Protocadherin gamma-C3	1.0000	4.52	3	3	3
Q$1047-2	Q9UN70	PCDGK_HUMAN	PCDHGC3	Protocadherin gamma-C3	1.0000	4.18	3	3	3
						GFAVGNVVANLGLDLGSLSAR	2	0.9956	1
						VLEDAPSGTR	2	0.9675	1
						YAELVLER	2	0.9956	1
Q$1048-1	Q9UNN8	EPCR_HUMAN	PROCR	Endothelial protein C receptor	0.9988	5.46	1	1	1
						EFLEDTCVQYVQK	2	0.9651	1
Q$1049-1	Q9Y287	ITM2B_HUMAN	ITM2B	Integral membrane protein 2B	0.9735	3.76	1	1	1
						NLLELLINIK	2	0.9735	1
Q$1050-1	Q9Y2S2-2	CRYL1_HUMAN	CRYL1	Isoform 2 of Lambda-crystallin homolog	1.0000	15.44	4	4	7
Q$1050-2	Q9Y2S2	CRYL1_HUMAN	CRYL1	Lambda-crystallin homolog	1.0000	14.42	4	4	7
						HVLQTFGPIPEFSR	3	0.9913	2
						IFAQLDSIIDDR	2	0.9994	2
						LFAGLVHVK	2	0.9992	1
						LQYAIISEAWR	2	0.9997	2
Q$1051-1	Q9Y2T7	YBOX2_HUMAN	YBX2	Y-box-binding protein 2	0.9957	18.13	2	2	2
						AGATAVPAATVPATAAGVVAVVVPVPAGEPQKGGGAGGGGGAASGPAAGTPSAPGSR	5	0.8305	1
						MSEVEAAAGATAVPAATVPATAAGVVAVVVPVPAGEPQKGGGAGGGGGAASGPAAGTPSAPGSRTP	5	0.9745	1
Q$1052-1	Q9Y2X9-2	ZN281_HUMAN	ZNF281	Isoform 2 of Zinc finger protein 281	0.9857	4.54	2	2	2
Q$1052-2	Q9Y2X9	ZN281_HUMAN	ZNF281	Zinc finger protein 281	0.9857	4.36	2	2	2
						MKIGSGFLSGGGGTGSSGGSGSGGGGSGGGGGGGSSGR	3	0.9219	1
						MKIGSGFLSGGGGTGSSGGSGSGGGGSGGGGGGGSSGRR	3	0.8173	1
Q$1053-1	Q9Y2Y8	PRG3_HUMAN	PRG3	Proteoglycan 3	1.0000	35.56	5	6	11
						CYGGNLVSIHDFNFNYR	2	0.9996	3
						CYGGNLVSIHDFNFNYR	3	0.9987	1
						FCWTDGSHWNFAYWSPGQPGNGQGSCVALCTK	3	0.8941	1
						IQCCTSTVNQAQVWIGGNLR	3	0.9978	3
						TFAEAQNVCSR	2	0.9996	2
						TSTVNQAQVWIGGNLR	2	0.9404	1
Q$1054-1	Q9Y5H0-2	PCDG3_HUMAN	PCDHGA3	Isoform 2 of Protocadherin gamma-A3	0.9312	1.21	1	1	1
Q$1054-2	Q9Y5H0	PCDG3_HUMAN	PCDHGA3	Protocadherin gamma-A3	0.9312	1.07	1	1	1
						LEKSIDQYYR	2	0.8911	1
Q$1055-1	Q9Y678	COPG1_HUMAN	COPG1	Coatomer subunit gamma-1	1.0000	3.43	2	2	3
						SIATLAITTLLK	2	0.9967	2
						SSPEPVALTESETEYVIR	2	0.9986	1
Q$1056-1	R4GMN6	R4GMN6_HUMAN	C1R	Complement C1r subcomponent (Fragment)	1.0000	10.32	2	2	5
						LPVANPQACENWLR	2	0.9983	3
						WVATGIVSWGIGCSR	2	0.9985	2
Q$1057-1	R4GN69	R4GN69_HUMAN	HSPH1	Heat shock protein 105 kDa	1.0000	12.17	1	1	3
						AGGIETIANEFSDR	2	0.9994	3
Q$1058-1	R4GN98	R4GN98_HUMAN	S100A6	Protein S100-A6 (Fragment)	0.9991	9.41	1	1	2
						LQDAEIAR	2	0.9901	2
Q$1059-1	V9GYE3	V9GYE3_HUMAN	APOA2	Apolipoprotein A-II	1.0000	84.62	4	5	11
						AGTELVNFLSYFVELGTQPATQ	3	0.9994	3
						SKEQLTPLIK	2	0.9958	1
						SKEQLTPLIKK	2	0.9656	1
						VKSPELQAEAK	2	0.9994	4
						VKSPELQAEAK	3	0.9979	2
Q$1060-1	V9GYZ6	V9GYZ6_HUMAN	EPRS	Bifunctional glutamate/proline--tRNA ligase (Fragment)	1.0000	2.79	2	2	5
						GFFICDQPYEPVSPYSCK	2	0.9996	3
						KGDIIQLQR	2	0.9975	2
Q$1061-1	X6R2L4	X6R2L4_HUMAN	RUVBL2	RuvB-like 2	1.0000	5.02	1	1	2
						TTEMETIYDLGTK	2	0.9976	2
Q$1062-1	X6RLJ0	X6RLJ0_HUMAN	C1QA	Complement C1q subcomponent subunit A (Fragment)	0.9997	4.55	1	1	1
						SLGFCDTTNK	2	0.997	1

**-------- Summary --------**		
**Total Peptides:**		**5,957**
**Total Proteins:**		**2,468**
**Total Groups (Q#):**		**1,062**
**Total Groups (I#):**		**1,071**
**Total Genes:**		**893**
**Total Proteins in the Database:**		**177,988**
**Total Genes in the Database:**		**25,200**
**Protein Quantification Rate (%):**		**1.387**
**Gene Quantification Rate (%):**		**3.544**
