# Supplementary material for: Label-free proteomic methodology for the analysis of human kidney stone matrix composition
Source: Proteome Sci. 2016 Feb 27;14:4. doi: 10.1186/s12953-016-0093-x (PMC4769560; doi:10.1186/s12953-016-0093-x)
Supplement: Additional file 3: — Human Kidney Stone Matrix Proteome Database – 1,039 proteins found in human kidney stone matrices from this study and 12 other proteomic papers. Includes details regarding Isoelectric Point, negatively charged residues (Asp + Glu), positively charged residues (Arg + Lys), Neg/Pos Ratio, Aliphatic index, GRAVY score, Molecular Class, Biological Process, Cellular component, and Function. (PDF 316 kb) [file 12953_2016_93_MOESM3_ESM.pdf]

SUPPLEMENTAL TABLE 3. HUMAN KIDNEY STONE MATRIX PROTEOME DATABASE

BOLD entries in columns 1-3 are common to one or more additional studies; *different isoform;
Gray shading for proteins detected **only** in other studies [1-12]

Bold Red - highly expressed in kidney
Red - moderately expressed in kidney
Black - low expression level in kidney
Bold Green - Not expressed in kidney

Gene Symbol	Protein Name	Found in other studies	Isoelectric Point	negatively charged residues (Asp + Glu)	positively charged residues (Arg + Lys)	Neg/Pos Ratio	Aliphatic index	GRAVY	Molecular Class	Biological Process	Cellular component	Function
EFTUD2	116 kDa U5 small nuclear ribonucleoprotein component		4.84	156	100	1.6	90.39	-0.245	Unclassified	Protein metabolism	Nucleus; Nucleolus; Cytoplasm; Chromosome	Component of the U5 snRNP and the U4/U6-U5 tri-snRNP complex required for pre-mRNA splicing. Binds GTP
PHPT1	14 kDa phosphohistidine phosphatase		5.66	18	14	1.3	73.23	-0.484	Enzyme: Phosphatase	Unknown	Cytoplasm	Exhibits phosphohistidine phosphatase activity.
YWHAB	14-3-3 protein beta/alpha		4.76	44	30	1.5	75.71	-0.740	Adapter molecule	Signal transduction	Cytoplasm; Mitochondrion	Adapter protein implicated in the regulation of a large spectrum of both general and specialized signaling pathways. Negative regulator of osteogenesis.
YWHAE	14-3-3 protein epsilon		4.63	51	32	1.6	85.76	-0.540	Adapter molecule	Cell communication; Signal transduction	Cytoplasm; Nucleus; Golgi apparatus; Nucleolus; Mitochondrion	Adapter protein implicated in the regulation of a large spectrum of both general and specialized signaling pathways.
YWHAH	14-3-3 protein eta		4.76	45	32	1.4	82.49	-0.629	Adapter molecule	Cell communication; Signal transduction	Cytoplasm; Nucleus	Adapter protein implicated in the regulation of a large spectrum of both general and specialized signaling pathways. Negatively regulates the kinase activity of PDPK1.
YWHAG	14-3-3 protein gamma		4.80	45	30	1.5	80.93	-0.691	Adapter molecule	Cell communication; Signal transduction	Cytoplasm; Endoplasmic reticulum; Golgi apparatus; Nucleus	Adapter protein implicated in the regulation of a large spectrum of both general and specialized signaling pathways.
SFN	14-3-3 protein sigma		4.68	48	30	1.6	77.98	-0.599	Adapter molecule	Cell communication; Signal transduction	Cytoplasm	Adapter protein implicated in the regulation of a large spectrum of both general and specialized signaling pathways. When bound to KRT17, regulates protein synthesis and epithelial cell growth by stimulating Akt/mTOR pathway.
YWHAQ	14-3-3 protein theta		4.68	44	30	1.5	83.71	-0.512	Adapter molecule	Cell communication; Signal transduction	Cytoplasm; Nucleus	Adapter protein implicated in the regulation of a large spectrum of both general and specialized signaling pathways. Negatively regulates the kinase activity of PDPK1.
HPGD	15-hydroxyprostaglandin dehydrogenase [NAD(+)]		5.56	28	23	1.2	99.77	-0.009	Enzyme: Dehydrogenase	Metabolism; Energy pathways	Cytoplasm	Prostaglandin inactivation. Contributes to the regulation of events that are under the control of prostaglandin levels. Catalyzes the NAD-dependent dehydrogenation of lipoxin A4 to form 15-oxo-lipoxin A4.
PSMC6	26S protease regulatory subunit 10B		7.09	60	60	1.0	93.98	-0.437	Ubiquitin proteasome system	Protein metabolism	Cytoplasm	The 26S protease is involved in the ATP-dependent degradation of ubiquitinated proteins. The regulatory (or ATPase) complex confers ATP dependency and substrate specificity to the 26S complex.
PSMC1	26S protease regulatory subunit 4		5.87	74	67	1.1	88.79	-0.548	Ubiquitin proteasome system	Protein metabolism	Nucleus; Cytoplasm	The 26S protease is involved in the ATP-dependent degradation of ubiquitinated proteins. The regulatory (or ATPase) complex confers ATP dependency and substrate specificity to the 26S complex.
PSMC3	26S protease regulatory subunit 6A		4.94	66	51	1.3	91.66	-0.396	Ubiquitin proteasome system	Protein metabolism	Nucleus	The 26S protease is involved in the ATP-dependent degradation of ubiquitinated proteins. The regulatory (or ATPase) complex confers ATP dependency and substrate specificity to the 26S complex.
PSMC5	26S protease regulatory subunit 8		7.27	60	60	1.0	99.14	-0.327	Ubiquitin proteasome system	Protein metabolism	Nucleus; Cytoplasm	The 26S protease is involved in the ATP-dependent degradation of ubiquitinated proteins. The regulatory (or ATPase) complex confers ATP dependency and substrate specificity to the 26S complex.
PSMD1	26S proteasome non-ATPase regulatory subunit 1		5.24	133	101	1.3	91.41	-0.229	Ubiquitin proteasome system	Protein metabolism; Proteolysis and peptidolysis	Nucleus; Cytoplasm	Acts as a regulatory subunit of the 26 proteasome which is involved in the ATP-dependent degradation of ubiquitinated proteins.
PSMD11	26S proteasome non-ATPase regulatory subunit 11		6.09	60	56	1.1	105.30	-0.187	Ubiquitin proteasome system	Protein metabolism; Proteolysis & peptidolysis	Nucleus; Cytoplasm	Component of the lid subcomplex of the 26S proteasome, a multiprotein complex involved in the ATP-dependent degradation of ubiquitinated proteins. In the complex, PSMD11 is required for proteasome assembly.
PSMD12	26S proteasome non-ATPase regulatory subunit 12		7.68	68	69	1.0	97.69	-0.389	Ubiquitin proteasome system	Protein metabolism	Cytoplasm	Acts as a regulatory subunit of the 26 proteasome which is involved in the ATP-dependent degradation of ubiquitinated proteins.
**ITIH4**	**35 kDa inter-alpha-trypsin inhibitor heavy chain H4**	**[1-2]**	6.51	101	97	1.0	84.49	-0.315	Protease inhibitor	Protein metabolism	Extracellular	Type II acute-phase protein (APP) involved in inflammatory responses to trauma. May also play a role in liver development or regeneration.
**MRPL12**	**39S ribosomal protein L12, mitochondrial**	**[1]**	5.33	22	19	1.2	118.63	0.008	Ribosomal subunit	Regulation of nucleobase, nucleoside, nucleotide and nucleic acid metabolism	Mitochondrion; Nucleolus	Positive regulation of transcription
BDH2	3-hydroxybutyrate dehydrogenase type 2		7.56	27	28	1.0	93.96	-0.022	**Enzyme: Oxidoreductase**	**Metabolism; Energy pathways**	**Unknown**	**Dehydrogenase that mediates the formation of 2,5-dihydroxybenzoic acid (2,5-DHBA), a siderophore that shares structural similarities with bacterial enterobactin and associates with LCN2, thereby playing a key role in iron homeostasis and transport.**
MPST	3-mercaptopyruvate sulfurtransferase		6.14	39	34	1.1	75.54	-0.433	Enzyme: Sulphotransferase	Metabolism; Energy pathways	Mitochondrion	Transfer of a sulfur ion to cyanide or to other thiol compounds. Also has weak rhodanese activity. Detoxifies cyanide and is required for thiosulfate biosynthesis. Acts as an antioxidant.
**RPS10**	**40S ribosomal protein S10**	**[1]**	9.92	19	31	0.6	72.62	-0.662	Ribosomal subunit	Protein metabolism	Ribosome; Nucleolus	Component of the 40S ribosomal subunit.
RPS2	40S ribosomal protein S2		10.87	15	37	0.4	75.28	-0.352	Ribosomal subunit	Protein metabolism	Ribosome; Nucleolus; Mitochondrion; Cytoplasm	Component of the 40S ribosomal subunit.
RPS23	40S ribosomal protein S23		10.23	11	27	0.4	75.67	-0.516	Ribosomal subunit	Protein metabolism	Ribosome; Nucleolus	Component of the 40S ribosomal subunit.; poly(A) RNA binding
RPS3	40S ribosomal protein S3		9.13	15	18	0.8	85.57	-0.324	Ribosomal subunit	Protein metabolism	Cytoplasm; Nucleus; Ribosome	Component of the 40S ribosomal subunit.
RPS3A	40S ribosomal protein S3a		9.46	29	40	0.7	78.06	-0.554	Ribosomal subunit	Protein metabolism	Cytoplasm; Nucleus; Nucleolus; Cytosol; Ribosome	May play a role during erythropoiesis through regulation of transcription factor DDIT3
RPS4X	40S ribosomal protein S4, X isoform		10.16	25	49	0.5	90.42	-0.338	Ribosomal subunit	Protein metabolism	Ribosome; Nucleolus; Cytosol	Component of the 40S ribosomal subunit.; poly(A) RNA binding
RPS5	40S ribosomal protein S5		8.66	17	19	0.9	93.28	-0.358	Ribosomal subunit	Protein metabolism	Ribosome; Nucleolus	Component of the 40S ribosomal subunit.; poly(A) RNA binding
RPS6	40S ribosomal protein S6		11.13	21	61	0.3	78.26	-0.976	Ribosomal subunit	Protein metabolism	Ribosome; Nucleolus	Component of the 40S ribosomal subunit.; poly(A) RNA binding
RPS7	40S ribosomal protein S7		10.09	23	38	0.6	94.90	-0.459	Ribosomal subunit	Protein metabolism	Ribosome; Nucleolus	Component of the 40S ribosomal subunit.; poly(A) RNA binding
RPS8	40S ribosomal protein S8		10.32	20	54	0.4	72.56	-1.034	Ribosomal subunit	Protein metabolism	Ribosome; Nucleolus	Component of the 40S ribosomal subunit.; poly(A) RNA binding
RPSA	40S ribosomal protein SA		4.79	40	26	1.5	79.08	-0.317	Cell surface receptor, Ribosomal subunit	Cell communication; Signal transduction; Cell adhesion	Cytoplasm; Ribosome; Nucleolus; Cytosol	Required for the assembly and/or stability of the 40S ribosomal subunit. Also functions as a cell surface receptor for laminin. Plays a role in cell adhesion to the basement membrane and in the consequent activation of signaling transduction pathways.
ALDH9A1	4-trimethylaminobutyraldehyde dehydrogenase		5.69	56	51	1.1	82.88	-0.048	**Enzyme: Dehydrogenase**	**Metabolism; Energy pathways**	**Cytoplasm**	**Converts gamma-trimethylaminobutyraldehyde into gamma-butyrobetaine. Catalyzes the irreversible oxidation of a broad range of aldehydes to the corresponding acids in an NAD-dependent reaction.**
**ALAS1**	**5-aminolevulinate synthase, nonspecific, mitochondrial**	**[1]**	8.16	64	67	1.0	77.96	-0.326	**Enzyme: Synthase**	**Amino acid and derivative metabolism**	**Mitochondrial matrix; Cytoplasm**	**Mitochondrial enzyme which is catalyzes the rate-limiting step in heme (iron-protoporphyrin) biosynthesis. The enzyme encoded by this gene is the housekeeping enzyme; a separate gene encodes a form of the enzyme that is specific for erythroid tissue.**
**PRKAB2**	**5'-AMP-activated protein kinase subunit beta-2**	**[3]**	5.99	35	29	1.2	76.29	-0.494	**Regulatory/other subunit**	**Cell communication; Signal transduction**	**Cytoplasm**	**Non-catalytic subunit of AMP-activated protein kinase (AMPK), an energy sensor protein kinase that plays a key role in regulating cellular energy metabolism.**
HSPD1	60 kDa heat shock protein, mitochondrial		5.24	80	79	1.0	100.71	-0.081	Heat shock protein	Protein metabolism; Protein folding; Apoptosis; Regulation of immune response; Signal transduction	Mitochondrion; Cytoplasm; Plasma membrane; Mitochondrial matrix; Endoplasmic reticulum; Peroxisomal matrix; Secretory granule; Extracellular; Golgi apparatus; Nucleus; Cytosol	Implicated in mitochondrial protein import and macromolecular assembly. May facilitate the correct folding of imported proteins. May also prevent misfolding and promote the refolding and proper assembly of unfolded polypeptides generated under stress conditions in the mitochondrial matrix.
RPLP0	60S acidic ribosomal protein P0		5.70	36	32	1.1	95.14	0.040	Ribosomal subunit	Protein metabolism	Nucleus; Ribosome; Nucleolus; Cytoplasm; Cytosol; Mitochondrion	Component of the 60S ribosomal subunit.; poly(A) RNA binding
RPLP1	60S acidic ribosomal protein P1		4.21	18	7	2.6	88.32	0.094	Ribosomal subunit	Protein metabolism	Ribosome; Cytoplasm; Mitochondrion; Nucleus	Plays an important role in the elongation step of protein synthesis.
RPLP2	60S acidic ribosomal protein P2		4.38	21	12	1.8	85.91	-0.237	Ribosomal subunit	Protein metabolism	Cytoplasm; Nucleolus	Plays an important role in the elongation step of protein synthesis.
RPL14	60S ribosomal protein L14		10.21	12	25	0.5	67.66	-0.544	Ribosomal subunit	Protein metabolism	Ribosome; Nucleolus	Component of the 60S ribosomal subunit.; poly(A) RNA binding
RPL15	60S ribosomal protein L15		11.62	11	50	0.2	67.89	-0.930	Ribosomal subunit	Protein metabolism	Ribosome; Nucleolus	Component of the 60S ribosomal subunit.; poly(A) RNA binding
RPL18	60S ribosomal protein L18		11.59	11	39	0.3	87.87	-0.546	Ribosomal subunit	Protein metabolism	Ribosome; Nucleolus	Component of the 60S ribosomal subunit.; poly(A) RNA binding
RPL22	60S ribosomal protein L22		10.16	5	9	0.6	79.06	-0.377	Ribosomal subunit	Protein metabolism	Ribosome; Nucleolus	Component of the 60S ribosomal subunit.; poly(A) RNA binding
RPL23A	60S ribosomal protein L23a		10.49	14	43	0.3	97.06	-0.401	Ribosomal subunit	Protein metabolism	Ribosome; Nucleolus	Component of the 60S ribosomal subunit.; poly(A) RNA binding
**RPL29**	**60S ribosomal protein L29**	**[1]**	11.66	7	47	0.1	60.82	-1.108	Ribosomal subunit	Protein metabolism	Plasma membrane; Nucleus; Cytoplasm; Nucleolus;	The protein belongs to the L29E family of ribosomal proteins. The protein is also a peripheral membrane protein expressed on the cell surface that directly binds heparin.
RPL3	60S ribosomal protein L3		10.19	39	86	0.5	74.13	-0.653	Ribosomal subunit	Protein metabolism	Ribosome; Nucleolus	Component of the 60S ribosomal subunit.; poly(A) RNA binding
RPL32	60S ribosomal protein L32		10.60	12	35	0.3	81.57	-0.680	Ribosomal subunit	Protein metabolism	Ribosome; Nucleolus	Component of the 60S ribosomal subunit.; poly(A) RNA binding
RPL5	60S ribosomal protein L5		9.73	35	59	0.6	66.93	-0.752	Ribosomal subunit	Protein metabolism	Ribosome; Nucleolus	Component of the 60S ribosomal subunit.; poly(A) RNA binding
RPL7A	60S ribosomal protein L7a		10.61	21	62	0.3	91.28	-0.556	Ribosomal subunit	Protein metabolism	Ribosome; Nucleolus	Component of the 60S ribosomal subunit.; poly(A) RNA binding
**RPL8**	**60S ribosomal protein L8**	**[1]**	11.04	17	54	0.3	76.95	-0.537	Ribosomal subunit	Protein metabolism	Nucleolus; Mitochondrion	Component of the 60S ribosomal subunit.; poly(A) RNA binding
PGD	6-phosphogluconate dehydrogenase, decarboxylating		6.88	56	55	1.0	86.06	-0.155	Enzyme: Dehydrogenase	Metabolism; Energy pathways	Cytoplasm	Catalyzes the oxidative decarboxylation of 6-phosphogluconate to ribulose 5-phosphate and CO2, with concomitant reduction of NADP to NADPH.
**PGLS**	**6-phosphogluconolactonase**	**[2]**	5.71	27	22	1.2	104.51	0.152	Enzyme: Hydrolase	Metabolism; Energy pathways	Cytoplasm; Extracellular vesicular exosome	Hydrolysis of 6-phosphogluconolactone to 6-phosphogluconate.
HSPA5	78 kDa glucose-regulated protein		5.01	111	87	1.3	83.21	-0.547	Chaperone	Protein metabolism	Endoplasmic reticulum; Plasma membrane; Cytoplasm; Nucleolus; Endoplasmic reticulum lumen; Mitochondrion; Cell surface	Facilitates the assembly of multimeric protein complexes inside the endoplasmic reticulum. Involved in the correct folding of proteins and degradation of misfolded proteins via its interaction with DNAJC10
ABI1	Abl interactor 1		6.61	49	47	1.0	69.47	-0.627	Adapter molecule	Regulation of cell growth; Signal transduction	Cytosol	Involved in cytoskeletal reorganization and EGFR signaling. Together with EPS8 participates in transduction of signals from Ras to Rac.Recruits WASF1 to lamellipodia and there seems to regulate WASF1 protein level.
**ASPM**	**Abnormal spindle-like microcephaly-associated protein**	**[1]**	10.45	235	684	0.3	89.29	-0.482	**Unclassified**	**Unknown**	**Nucleus; Microtubule**	**Probable role in mitotic spindle regulation and coordination of mitotic processes. May have a preferential role in regulating neurogenesis.**
**ASAH1**	**Acid ceramidase**	**[1, 3]**	5.63	13	11	1.2	90.99	-0.099	Enzyme: Hydrolase	Metabolism; Energy pathways	Lysosome	Hydrolyzes the sphingolipid ceramide into sphingosine and free fatty acid.
**ANP32A**	**Acidic leucine-rich nuclear phosphoprotein 32 family member A**	**[3, 4]**	4.47	40	22	1.8	94.69	-0.711	MHC complex protein	Immune response	Cytoplasm; Nucleus; Plasma membrane	Implicated in a number of cellular processes, including proliferation, differentiation, caspase-dependent and caspase-independent apoptosis, suppression of transformation (tumor suppressor), inhibition of protein phosphatase 2A, regulation of mRNA trafficking and stability in association with ELAVL1, and inhibition of acetyltransferases as part of the INHAT (inhibitor of histone acetyltransferases) complex. Plays a role in E4F1-mediated transcriptional repression.

**ANP32B**	**Acidic leucine-rich nuclear phosphoprotein 32 family member B**	**[1, 4]**	3.93	94	27	3.5	75.70	-1.164	Chaperone	Negative regulation of cell differentiation	Nucleus; Nucleolus; Cytoplasm	Multifunctional protein working as a cell cycle progression factor as well as a cell survival factor. Required for the progression from the G1 to the S phase. Anti-apoptotic protein which functions as a caspase-3 inhibitor. Has no phosphatase 2A (PP2A) inhibitor activity By similarity. Exhibits histone chaperone properties, stimulating core histones to assemble into a nucleosome
ANP32E	Acidic leucine-rich nuclear phosphoprotein 32 family member E		3.76	102	22	4.6	74.22	-1.176	Chaperone	Histone exchange	Nucleus; Nucleolus; Cytoplasm; cytoplasmic membrane-bounded vesicle	Histone chaperone that specifically mediates the genome-wide removal of histone H2A.Z/H2AFZ from the nucleosome: removes H2A.Z/H2AFZ from its normal sites of deposition, especially from enhancer and insulator regions.
**ACPP**	**ACPP protein**	**[2]**	9.24	29	36	0.8	90.38	-0.327	**Enzyme: Acid phosphatase**	**Metabolism; Energy pathways**	**Extracellular; Cytoplasm; Lysosome; Nucleus**	**A non-specific tyrosine phosphatase that dephosphorylates a diverse number of substrates under acidic conditions (pH 4-6) including alkyl, aryl, and acyl orthophosphate monoesters and phosphorylated proteins. Has lipid phosphatase activity and inactivates lysophosphatidic acid in seminal plasma.**
**ACTA1**	**Actin, alpha**	**[3, 4]**	5.23	50	37	1.4	82.21	-0.245	**Structural protein**	**Cell growth and/or maintenance**	**Cytoplasm; Kinetochore; Extracellular**	**Actins are highly conserved proteins that are involved in various types of cell motility and are ubiquitously expressed in all eukaryotic cells.**
**ACTB**	**Actin, cytoplasmic 1**	**[1, 4-6, 11]**	5.29	49	37	1.3	82.17	-0.205	Cytoskeletal protein	Cell growth and/or maintenance	Cytoplasm; Golgi apparatus; Plasma membrane; Nucleolus; Cytosol; Extracellular; Cytoskeleton; Mitochondrion	Actins are highly conserved proteins that are involved in various types of cell motility and are ubiquitously expressed in all eukaryotic cells.
ACTR2	Actin-related protein 2		6.29	53	50	1.1	96.14	-0.185	Cytoskeletal protein	Cell motility; Cell growth and/or maintenance	Cytoplasm; Actin cytoskeleton	Functions as ATP-binding component of the Arp2/3 complex which is involved in regulation of actin polymerization and together with an activating nucleation-promoting factor (NPF) mediates the formation of branched actin networks. Seems to contact the pointed end of the daughter actin filament.
ARPC4	Actin-related protein 2/3 complex subunit 4		6.83	12	12	1.0	84.81	-0.175	Cytoskeletal associated protein	Cell growth and/or maintenance	Cytoskeleton; Nucleolus	Functions as actin-binding component of the Arp2/3 complex which is involved in regulation of actin polymerization and together with an activating nucleation-promoting factor (NPF) mediates the formation of branched actin networks. Seems to contact the mother actin filament.
ACTR3	Actin-related protein 3		5.61	57	48	1.2	83.88	-0.271	Cytoskeletal protein	Cell growth and/or maintenance	Cytoskeleton; Nucleolus	Functions as ATP-binding component of the Arp2/3 complex which is involved in regulation of actin polymerization and together with an activating nucleation-promoting factor (NPF) mediates the formation of branched actin networks. Seems to contact the pointed end of the daughter actin filament. Plays a role in ciliogenesis
ACTR3B	Actin-related protein 3B		5.61	57	48	1.2	87.36	-0.195	Cytoskeletal protein	Cell growth and/or maintenance	Cytoskeleton	Plays a role in the organization of the actin cytoskeleton. May function as ATP-binding component of the Arp2/3 complex which is involved in regulation of actin polymerization and together with an activating nucleation-promoting factor (NPF) mediates the formation of branched actin networks. May decrease the metastatic
**SUB1**	**Activated RNA polymerase II transcriptional coactivator p15**	**[3]**	9.60	20	27	0.7	61.03	-1.117	Transcription factor	Regulation of nucleobase, nucleoside, nucleotide and nucleic acid metabolism	Nucleus; Cytoplasm; Nucleolus; Mitochondrion	General coactivator that functions cooperatively with TAFs and mediates functional interactions between upstream activators and the general transcriptional machinery. May be involved in stabilizing the multiprotein transcription complex. Binds single-stranded DNA. Also binds, in vitro, non-specifically to double-stranded DNA (ds DNA).
ACOT11	Acyl-coenzyme A thioesterase 11		8.66	71	79	0.9	82.22	-0.385	Enzyme: Esterase	Metabolism; Energy pathways	Cytoplasm	Has acyl-CoA thioesterase activity towards medium (C12) and long-chain (C18) fatty acyl-CoA substrates.
**AHCY**	**Adenosylhomocysteinase**	**[3, 5]**	5.92	53	46	1.2	93.25	-0.107	**Enzyme: Hydrolase**	**Metabolism; Energy pathways**	**Cytoplasm**	**Competitive inhibitor of S-adenosyl-L-methionine-dependent methyl transferase reactions; therefore adenosylhomocysteinase may play a key role in the control of methylations via regulation of the intracellular concentration of adenosylhomocysteine.**
ADSS	Adenylosuccinate synthetase isozyme 2		6.13	54	50	1.1	90.61	-0.178	Enzyme: Synthase	Metabolism; Energy pathways	Cytoplasm	Plays an important role in the de novo pathway and in the salvage pathway of purine nucleotide biosynthesis. Catalyzes the first committed step in the biosynthesis of AMP from IMP.
**CAP1**	**Adenylyl cyclase-associated protein 1**	**[1]**	8.26	53	56	0.9	82.68	-0.357	Unclassified	Cell growth and/or maintenance	Cortical actin cytoskeleton; extracellular vesicular exosome; plasma membrane	Directly regulates filament dynamics and has been implicated in a number of complex developmental and morphological processes, including mRNA localization and the establishment of cell polarity.
ARF1	ADP-ribosylation factor 1		6.36	23	22	1.0	95.89	-0.274	**GTPase**	**Signal transduction; Endosome transport**	**Cytoplasm; Golgi apparatus; Plasma membrane; Endosome; Golgi membrane**	**Involved in protein trafficking among different compartments. Modulates vesicle budding and uncoating within the Golgi complex. Deactivation induces the redistribution of the entire Golgi complex to the endoplasmic reticulum, suggesting a crucial role in protein trafficking.**
ARF3	ADP-ribosylation factor 3		7.04	23	23	1.0	100.22	-0.258	**G protein**	**Cell communication; Signal transduction**	**Plasma membrane; Cytoplasm; Golgi apparatus**	**GTP-binding protein that functions as an allosteric activator of the cholera toxin catalytic subunit, an ADP-ribosyltransferase. Involved in protein trafficking; may modulate vesicle budding and uncoating within the Golgi apparatus.**
ARF4	ADP-ribosylation factor 4		6.81	21	21	1.0	102.91	-0.104	Transport/cargo protein	Transport	Plasma membrane; Cytoplasm; Golgi apparatus	GTP-binding protein that functions as an allosteric activator of the cholera toxin catalytic subunit, an ADP-ribosyltransferase. Involved in protein trafficking; may modulate vesicle budding and uncoating within the Golgi apparatus.
ARF5	ADP-ribosylation factor 5		6.35	22	21	1.0	95.25	-0.216	G protein	Cell communication; Signal transduction	Plasma membrane; Cytoplasm; Golgi apparatus	GTP-binding protein that functions as an allosteric activator of the cholera toxin catalytic subunit, an ADP-ribosyltransferase. Involved in protein trafficking; may modulate vesicle budding and uncoating within the Golgi apparatus.
AGRN	Agrin		5.93	219	193	1.1	67.80	-0.274	Extracellular matrix protein	Cell growth and/or maintenance	Extracellular	Heparan sulfate basal lamina glycoprotein that plays a central role in the formation and the maintenance of the neuromuscular junction (NMJ) and directs key events in postsynaptic differentiation.
AKR1A1	Alcohol dehydrogenase [NADP(+)]		6.35	39	36	1.1	93.02	-0.277	**Enzyme: Oxidoreductase**	**Metabolism**	**Cytoplasm; Plasma membrane; Mitochondrion**	**Catalyzes the NADPH-dependent reduction of a variety of aromatic and aliphatic aldehydes to their corresponding alcohols. Catalyzes the reduction of mevaldate to mevalonic acid and of glyceraldehyde to glycerol.**
AKR1C1	Aldo-keto reductase family 1 member C1		8.02	41	43	1.0	92.04	-0.331	Enzyme: Reductase	Metabolism; Energy pathways	Cytoplasm	Converts progesterone to its inactive form, 20-alpha-dihydroxyprogesterone (20-alpha-OHP). In the liver and intestine, may have a role in the transport of bile.
AKR1C2	Aldo-keto reductase family 1 member C2		7.13	41	41	1.0	92.94	-0.301	Enzyme: Dehydrogenase	Metabolism; Transport	Cytosol	Works in concert with the 5-alpha/5-beta-steroid reductases to convert steroid hormones into the 3-alpha/5-alpha and 3-alpha/5-beta-tetrahydrosteroids.
AKR1C3	Aldo-keto reductase family 1 member C3		8.06	41	43	1.0	86.63	-0.415	Enzyme: Reductase	Metabolism; Energy pathways	Cytoplasm	Catalyzes the conversion of aldehydes and ketones to alcohols. Catalyzes the reduction of prostaglandin (PG) D2, PGH2 and phenanthrenequinone (PQ) and the oxidation of 9-alpha,11-beta-PGF2 to PGD2.
AKR1B1	Aldose reductase		6.55	38	36	1.1	93.43	-0.260	Enzyme: Oxidoreductase	Metabolism; Energy pathways	Cytoplasm; Extracellular	Catalyzes the NADPH-dependent reduction of a wide variety of carbonyl-containing compounds to their corresponding alcohols with a broad range of catalytic efficiencies
ABHD14B	Alpha/beta hydrolase domain-containing protein 14B		7.72	12	13	0.9	96.06	0.192	**Enzyme: Hydrolase**	**Positive regulation of transcription from RNA polymerase II promoter**	**Cytoplasm; Nucleus**	**Has hydrolase activity towards p-nitrophenyl butyrate (in vitro). May activate transcription.**
**ORM1**	**Alpha-1-acid glycoprotein 1**	**[1, 4, 6-8]**	5.00	30	22	1.4	70.33	-0.764	Secreted polypeptide	Immune response	Extracellular	Functions as transport protein in the blood stream. Binds various ligands in the interior of its beta-barrel domain. Also binds synthetic drugs and influences their distribution and availability in the body. Appears to function in modulating the activity of the immune system during the acute-phase reaction.
**ORM2**	**Alpha-1-acid glycoprotein 2**	**[7, 8]**	5.12	30	23	1.3	62.35	-0.816	Secreted polypeptide	Immune response	Extracellular	Functions as transport protein in the blood stream. Binds various ligands in the interior of its beta-barrel domain. Also binds synthetic drugs and influences their distribution and availability in the body. Appears to function in modulating the activity of the immune system during the acute-phase reaction.
**SERPINA3**	**Alpha-1-antichymotrypsin**	**[1, 2]**	5.32	53	41	1.3	94.38	-0.163	Protease inhibitor	Protein metabolism	Extracellular; Cytoplasm; Nucleus	Although its physiological function is unclear, it can inhibit neutrophil cathepsin G and mast cell chymase, both of which can convert angiotensin-1 to the active angiotensin-2.
**SERPINA1**	**Alpha-1-antitrypsin**	**[1-2, 4, 5, 7, 8]**	5.37	56	41	1.4	87.11	-0.302	Protease inhibitor	Protein metabolism	Extracellular; Endoplasmic reticulum; Lysosome; Cytoplasm	Inhibitor of serine proteases. Its primary target is elastase, but it also has a moderate affinity for plasmin and thrombin. Irreversibly inhibits trypsin, chymotrypsin and plasminogen activator.
**A1BG**	**Alpha-1B-glycoprotein**	**[1, 2, 5, 8, 9]**	5.63	58	44	1.3	83.33	-0.291	Secreted polypeptide	Unknown	Extracellular	Unknown
**SERPINF2**	**Alpha-2-antiplasmin**	**[1]**	5.87	46	38	1.2	89.96	-0.196	Protease inhibitor	Protein metabolism	Extracellular	Serine protease inhibitor. The major targets of this inhibitor are plasmin and trypsin, but it also inactivates matriptase-3/TMPRSS7 and chymotrypsin.
**AHSG**	**Alpha-2-HS-glycoprotein**	**[1, 2-4, 10]**	4.53	39	19	2.1	80.60	-0.284	Secreted polypeptide	Cell communication; Signal transduction	Extracellular	Synthesized by hepatocytes, promotes endocytosis, possesses opsonic properties and influences the mineral phase of bone. Shows affinity for calcium and barium ions.
**A2M**	**Alpha-2-macroglobulin**	**[1, 6]**	5.98	156	133	1.2	85.06	-0.214	Protease inhibitor	Protein metabolism	Extracellular; Cytoplasm	Is able to inhibit all four classes of proteinases by a unique 'trapping' mechanism.
**ACTN1**	**Alpha-actinin-1**	**[8]**	5.47	129	102	1.3	84.20	-0.536	Cytoskeletal associated protein; Structural protein	Cell growth and/or maintenance	Cytoplasm; Mitochondrial membrane; Cytosol; Cytoskeleton; Mitochondrion; Nucleus	F-actin cross-linking protein which is thought to anchor actin to a variety of intracellular structures. This is a bundling protein.
**ACTN2**	**Alpha-actinin-2**	**[8]**	5.31	146	113	1.3	84.53	-0.597	Cytoskeletal associated protein	Cell growth and/or maintenance	Cytoplasm; Mitochondrial membrane; Actin filament	F-actin cross-linking protein which is thought to anchor actin to a variety of intracellular structures. This is a bundling protein.
ACTN4	Alpha-actinin-4		5.27	150	109	1.4	81.88	-0.637	**Cytoskeletal protein**	**Cell growth and/or maintenance**	**Cytoplasm; Nucleus; Plasma membrane; Mitochondrion**	**F-actin cross-linking protein which is thought to anchor actin to a variety of intracellular structures. This is a bundling protein. Probably involved in vesicular trafficking via its association with the CART complex. Involved in tight junction assembly in epithelial cells probably through interaction with**
CRYAB	Alpha-crystallin B chain		6.18	18	14	1.3	94.19	-0.022	Heat shock protein	Protein metabolism	Nucleus	May contribute to the transparency and refractive index of the lens. Has chaperone-like activity, preventing aggregation of various proteins under a wide range of stress conditions.
**ENO1**	**Alpha-enolase**	**[10]**	6.99	55	55	1.0	88.78	-0.226	Enzyme: Hydratase	Metabolism; Energy pathways	Cytoplasm; Plasma membrane; Nucleus; Extracellular	Multifunctional enzyme that, as well as its role in glycolysis, plays a part in various processes such as growth control, hypoxia tolerance and allergic responses. May also function in the intravascular and pericellular fibrinolytic system due to its ability to serve as a receptor and activator of plasminogen on the cell surface of several cell-types such as leukocytes and neurons. Stimulates immunoglobulin production.
NAPA	Alpha-soluble NSF attachment protein		5.08	42	31	1.4	80.98	-0.361	Adapter molecule	Transport	Golgi apparatus; Cytoplasm; Lysosome	Required for vesicular transport between the endoplasmic reticulum and the Golgi apparatus.
AIMP1	Aminoacyl tRNA synthase complex-interacting multifunctional protein 1		8.62	44	48	0.9	88.71	-0.524	Cytokine	Immune response	Nucleus; Cytoplasm; Extracellular; Microtubule; Ribosome	Stimulates the catalytic activity of cytoplasmic arginyl-tRNA synthase. Binds tRNA. Possesses inflammatory cytokine activity. Negatively regulates TGF-beta signaling through stabilization of SMURF2 by binding to SMURF2 and inhibiting its SMAD7-mediated degradation. Involved in glucose homeostasis through induction of glucagon secretion at low glucose levels.
ACY1	Aminoacylase-1		6.56	53	49	1.1	70.70	-0.307	**Enzyme: Hydrolase**	**Metabolism; Energy pathways**	**Cytoplasm**	**Involved in the hydrolysis of N-acylated or N-acetylated amino acids (except L-aspartate).**
RNPEP	Aminopeptidase B		5.51	77	61	1.3	80.62	-0.210	Aminopeptidase	Protein metabolism	Golgi apparatus; Plasma membrane; Extracellular; Nucleus	Exopeptidase which selectively removes arginine and/or lysine residues from the N-terminus of several peptide substrates including Arg(0)-Leu-enkephalin, Arg(0)-Met-enkephalin and Arg(-1)-Lys(0)-somatostatin-14. Can hydrolyze leukotriene A4 (LTA-4) into leukotriene B4 (LTB-4).
**ANPEP**	**Aminopeptidase N**	**[2]**	5.31	109	85	1.3	84.60	-0.319	**Metallo protease**	**Protein metabolism**	**Plasma membrane; Extracellular**	**May be involved in the metabolism of regulatory peptides of diverse cell types, responsible for the processing of peptide hormones, such as angiotensin III and IV, neuropeptides, and chemokines. Found to cleave antigen peptides bound to major histocompatibility complex class II molecules of presenting cells and to degrade neurotransmitters at synaptic junctions.**
**APP**	**Amyloid beta A4 protein**	**[3, 4]**	4.72	142	77	1.8	71.06	-0.631	Cell surface receptor	Cell communication; Signal transduction	Nucleus; Integral to membrane; Cell surface; Vesicle; Endoplasmic reticulum; Golgi apparatus; Endosome; Cytoplasm; Extracellular; Plasma membrane	Functions as a cell surface receptor and performs physiological functions on the surface of neurons relevant to neurite growth, neuronal adhesion and axonogenesis. Involved in cell mobility and transcription regulation through protein-protein interactions.
**APLP2**	**Amyloid-like protein 2**	**[3]**	4.71	144	79	1.8	71.37	-0.673	Integral membrane protein	Cell communication; Signal transduction	Plasma membrane	May play a role in the regulation of hemostasis. The soluble form may have inhibitory properties towards coagulation factors. May interact with cellular G-protein signaling pathways.Inhibits trypsin, chymotrypsin, plasmin, factor XIA and plasma and glandular kallikrein. Modulates the Cu/Zn nitric oxide-catalyzed autodegradation of GPC1 heparan sulfate side chains in fibroblasts.
ZFAND4	AN1-type zinc finger protein 4		8.80	67	78	0.9	72.86	-0.581	Unclassified	Protein metabolism	Unknown	Zinc ion binding.
**AMOT**	**Angiomotin**	**[4]**	7.27	116	115	1.0	69.10	-0.712	**Unclassified**	**Unknown**	**Plasma membrane**	**Plays a central role in tight junction maintenance via the complex formed with ARHGAP17, which acts by regulating the uptake of polarity proteins at tight junctions. Appears to regulate endothelial cell migration and tube formation. May also play a role in the assembly of endothelial cell-cell junctions.**
TEK	Angiopoietin-1 receptor		6.61	125	119	1.1	84.31	-0.308	Receptor tyrosine kinase	Cell communication; Signal transduction	Plasma membrane	Tyrosine-protein kinase that acts as cell-surface receptor for ANGPT1, ANGPT2 and ANGPT4 and regulates angiogenesis, endothelial cell survival, proliferation, migration, adhesion and cell spreading, reorganization of the actin cytoskeleton, but also maintenance of vascular quiescence. Has anti-inflammatory effects by preventing the leakage of proinflammatory plasma proteins and leukocytes from blood vessels.
ANGPTL2	Angiopoietin-related protein 2		7.02	56	55	1.0	73.25	-0.783	Secreted polypeptide	Cell development	Extracellular	Induces sprouting in endothelial cells through an autocrine and paracrine action.
ANGPTL6	Angiopoietin-related protein 6		8.70	44	48	0.9	76.84	-0.468	Secreted polypeptide	Cell communication; Signal transduction; Blood vessel development; Wound healing	Extracellular	May play a role in the wound healing process. May promote epidermal proliferation, remodeling and regeneration. May promote the chemotactic activity of endothelial cells and induce neovascularization. May counteract high-fat diet-induced obesity and related insulin resistance through increased energy expenditure.

**AGT**	**Angiotensinogen**	**[1, 2]**	5.60	46	34	1.4	99.47	0.033	Peptide hormone	Cell communication; Signal transduction	Extracellular	Essential component of the renin-angiotensin system (RAS), a potent regulator of blood pressure, body fluid and electrolyte homeostasis.
ANKS1A	Ankyrin repeat and SAM domain-containing protein 1A		5.93	148	122	1.2	77.09	-0.570	Adapter molecule	Cell communication; Signal transduction	Cytoplasm	Regulator of different signaling pathways. Regulates EPHA8 receptor tyrosine kinase signaling to control cell migration and neurite retraction.
**ASB17**	**Ankyrin repeat and SOCS box protein 17**	**[3]**	9.05	27	37	0.7	110.31	0.049	**Unclassified**	**Cell communication; Signal transduction**	**Cytosol; Mitochondrion**	**May be a substrate-recognition component of a SCF-like ECS (Elongin-Cullin-SOCS-box protein) E3 ubiquitin-protein ligase complex which mediates the ubiquitination and subsequent proteasomal degradation of target proteins.**
**ANKRD30A**	**Ankyrin repeat domain-containing protein 30A**	**[3]**	6.08	232	210	1.1	73.76	-0.827	**Transcription regulatory protein**	**Regulation of nucleobase, nucleoside, nucleotide and nucleic acid metabolism**	**Nucleus**	**?**
**ANXA1**	**Annexin A1**	**[1, 11]**	6.64	52	51	1.0	87.71	-0.426	Calcium binding protein	Cell communication; Signal transduction	Cytoplasm; Nucleus; Plasma membrane; Mitochondrion	Calcium/phospholipid-binding protein which promotes membrane fusion and is involved in exocytosis. This protein regulates phospholipase A2 activity. It seems to bind from two to four calcium ions with high affinity.
ANXA10	Annexin A10		5.13	43	30	1.4	80.77	-0.265	Calcium binding protein	Cell communication; Signal transduction	Mitochondrion	Calcium-dependent phospholipid binding.
ANXA11	Annexin A11		7.53	50	51	1.0	64.97	-0.521	Calcium binding protein	Cell communication; Signal transduction	Cytoplasm; Nucleus	Binds specifically to calcyclin in a calcium-dependent manner By similarity. Required for midbody formation and completion of the terminal phase of cytokinesis.
**ANXA2**	**Annexin A2**	**[1-2, 6, 10, 11]**	5.68	36	32	1.1	91.22	-0.445	Calcium binding protein	Cell communication; Signal transduction	Cytoplasm; Nucleus; Plasma membrane; Endosome; Extracellular; Nucleolus; Apical membrane; Cytoskeleton	Calcium-regulated membrane-binding protein whose affinity for calcium is greatly enhanced by anionic phospholipids. It binds two calcium ions with high affinity. May be involved in heat-stress response.
**ANXA3**	**Annexin A3**	**[6]**	5.63	46	40	1.2	92.43	-0.420	Calcium binding protein	Cell communication; Signal transduction	Cytoplasm	Inhibitor of phospholipase A2, also possesses anti-coagulant properties. Also cleaves the cyclic bond of inositol 1,2-cyclic phosphate to form inositol 1-phosphate.
ANXA4	Annexin A4		5.84	49	46	1.1	84.06	-0.447	Calcium binding protein	Cell communication; Signal transduction	Nucleus; Cytoplasm; Golgi apparatus	Calcium/phospholipid-binding protein which promotes membrane fusion and is involved in exocytosis.
**ANXA5**	**Annexin A5**	**[6]**	4.93	54	41	1.3	91.16	-0.337	Calcium binding protein	Cell communication; Signal transduction	Extracellular; Cytoplasm; Nucleus; Plasma membrane; Endoplasmic reticulum	This protein is an anticoagulant protein that acts as an indirect inhibitor of the thromboplastin-specific complex, which is involved in the blood coagulation cascade.
ANXA9	Annexin A9		5.53	43	37	1.2	98.17	-0.243	Calcium binding protein	Cell communication; Signal transduction	Cytoplasm; Nucleoplasm	Low affinity receptor for acetylcholine known to be targeted by disease-causing pemphigus vulgaris antibodies in keratinocytes.
**AGR2**	**Anterior gradient protein 2 homolog**	**[5]**	9.06	20	24	0.8	87.48	-0.610	Dystroglycan binding	Lung goblet cell differentiation	Extracellular; Endoplasmic reticulum	Required for MUC2 post-transcriptional synthesis and secretion. May play a role in the production of mucus by intestinal cells By similarity. Proto-oncogene that may play a role in cell migration, cell differentiation and cell growth.
**SLPI**	**Antileukoproteinase**	**[4]**	9.11	8	20	0.4	38.22	-0.649	Protease inhibitor	Protein metabolism	Extracellular	Acid-stable proteinase inhibitor with strong affinities for trypsin, chymotrypsin, elastase, and cathepsin G. May prevent elastase-mediated damage to oral and possibly other mucosal tissues.
**SERPINC1**	**Antithrombin-III**	**[1, 5]**	5.95	61	57	1.1	81.94	-0.359	Protease inhibitor	Protein metabolism	Extracellular	Most important serine protease inhibitor in plasma that regulates the blood coagulation cascade. AT-III inhibits thrombin, matriptase-3/TMPRSS7, as well as factors IXa, Xa and XIa. Its inhibitory activity is greatly enhanced in the presence of heparin.
**AP3B1**	**AP-3 complex subunit beta-1**	**[3]**	5.75	151	135	1.1	89.47	-0.383	Adapter molecule	Protein metabolism	Plasma membrane; Cytoplasm; Nucleus	Subunit of non-clathrin- and clathrin-associated adaptor protein complex 3 (AP-3) that plays a role in protein sorting in the late-Golgi/trans-Golgi network (TGN) and/or endosomes. The AP complexes mediate both the recruitment of clathrin to membranes and the recognition of sorting signals within the cytosolic tails of transmembrane cargo molecules. AP-3 appears to be involved in the sorting of a subset of transmembrane proteins targeted to lysosomes and lysosome-related organelles.
AP3D1	AP-3 complex subunit delta		8.69	158	172	0.9	88.54	-0.494	Transport/cargo protein	Transport	Cytoplasm; Golgi apparatus	Part of the AP-3 complex, an adaptor-related complex which is not clathrin-associated. The complex is associated with the Golgi region as well as more peripheral structures. It facilitates the budding of vesicles from the Golgi membrane and may be directly involved in trafficking to lysosomes.
**APOA1**	**Apolipoprotein A-I**	**[1, 3, 4, 8, 9, 12]**	5.27	46	37	1.2	82.72	-0.840	Transport/cargo protein	Transport	Extracellular; Cytoplasm; Golgi apparatus	Participates in the reverse transport of cholesterol from tissues to the liver for excretion by promoting cholesterol efflux from tissues and by acting as a cofactor for the lecithin cholesterol acyltransferase (LCAT).
**APOA2**	**Apolipoprotein A-II**	**[1]**	5.05	11	9	1.2	74.68	-0.503	Transport/cargo protein	Transport	Extracellular	May stabilize HDL (high density lipoprotein) structure by its association with lipids, and affect the HDL metabolism.
**APOA4**	**Apolipoprotein A-IV**	**[1, 3]**	5.18	68	52	1.3	81.76	-0.939	Transport/cargo protein	Transport	Extracellular	May have a role in chylomicrons and VLDL secretion and catabolism. Required for efficient activation of lipoprotein lipase by ApoC-II; potent activator of LCAT. Apoa-IV is a major component of HDL and chylomicrons.
**APOB**	**Apolipoprotein B-100**	**[1, 3, 6]**	6.57	530	504	1.1	91.73	-0.306	Transport/cargo protein	Transport	Extracellular; Cytoplasm; Golgi apparatus	Apolipoprotein B is a major protein constituent of chylomicrons (apo B-48), LDL (apo B-100) and VLDL (apo B-100). Apo B-100 functions as a recognition signal for the cellular binding and internalization of LDL particles by the apoB/E receptor.
**APOC1**	**Apolipoprotein C-I**	**[1]**	7.93	11	12	0.9	77.02	-0.839	Transport/cargo protein	Transport	Extracellular	Inhibitor of lipoprotein binding to the low density lipoprotein (LDL) receptor, LDL receptor-related protein, and very low density lipoprotein (VLDL) receptor. Associates with high density lipoproteins (HDL) and the triacylglycerol-rich lipoproteins in the plasma and makes up about 10% of the protein of the VLDL and 2% of that of HDL. Appears to interfere directly with fatty acid uptake and is also the major plasma inhibitor of cholesteryl ester transfer protein (CETP). Binds free fatty acids and reduces their intracellular esterification.
**APOC2**	**Apolipoprotein C-II**	**[1]**	4.58	11	7	1.6	66.71	-0.641	Transport/cargo protein	Metabolism; Energy pathways	Extracellular	Component of chylomicrons, very low-density lipoproteins (VLDL), low-density lipoproteins (LDL), and high-density lipoproteins (HDL) in plasma. Plays an important role in lipoprotein metabolism as an activator of lipoprotein lipase. Both proapolipoprotein C-II and apolipoprotein C-II can activate lipoprotein lipase.
**APOC3**	**Apolipoprotein C-III**	**[3]**	4.72	12	8	1.5	59.37	-0.505	Transport/cargo protein	Transport	Extracellular	Inhibits lipoprotein lipase and hepatic lipase and decreases the uptake of lymph chylomicrons by hepatic cells. This suggests that it delays the catabolism of triglyceride-rich particles.
**APOD**	**Apolipoprotein D**	**[1-5, 8, 12]**	5.20	19	15	1.3	86.51	-0.291	Transport/cargo protein	Transport	Extracellular	APOD occurs in the macromolecular complex with lecithin-cholesterol acyltransferase. It is probably involved in the transport and binding of bilin. Appears to be able to transport a variety of ligands in a number of different contexts.
**APOE**	**Apolipoprotein E**	**[12]**	5.52	51	46	1.1	83.91	-0.727	Transport/cargo protein	Transport	Cytoplasm; Extracellular	Mediates the binding, internalization, and catabolism of lipoprotein particles. It can serve as a ligand for the LDL (apo B/E) receptor and for the specific apo-E receptor (chylomicron remnant) of hepatic tissues.
**APOL1**	**Apolipoprotein L1**	**[1]**	5.49	52	44	1.2	93.61	-0.365	Transport/cargo protein	Transport; Lipid metabolism	Extracellular	May play a role in lipid exchange and transport throughout the body. May participate in reverse cholesterol transport from peripheral cells to the liver.
APOM	Apolipoprotein M		5.66	21	16	1.3	77.87	-0.259	Transport/cargo protein	Transport; Lipid metabolism	Extracellular; Plasma membrane; Endoplasmic reticulum	Probably involved in lipid transport. Can bind sphingosine-1-phosphate, myristic acid, palmitic acid and stearic acid, retinol, all-trans-retinoic acid and 9-cis-retinoic acid.
LPA	Apolipoprotein(a)		5.57	408	297	1.4	42.73	-0.732	Transport/cargo protein	Transport	Extracellular	Apo(a) is the main constituent of lipoprotein(a) (Lp(a)). It has serine proteinase activity and is able of autoproteolysis. Inhibits tissue-type plasminogen activator 1. Lp(a) may be a ligand for megalin/Gp 330.
ARCN1	Archain 1 (Coatomer subunit delta)		5.63	78	68	1.1	83.17	-0.378	Transport/cargo protein	Transport	Cytoplasm; Golgi apparatus; Cytosol	The coatomer is a cytosolic protein complex that binds to dilysine motifs and reversibly associates with Golgi non-clathrin-coated vesicles, which further mediate biosynthetic protein transport from the ER, via the Golgi up to the trans Golgi network. Coatomer complex is required for budding from Golgi membranes, and is essential for the retrograde Golgi-to-ER transport of dilysine-tagged proteins.
**ARFIP1**	**Arfaptin-1**	**[1]**	6.25	49	45	1.1	87.61	-0.533	Unclassified	Signal transduction	Cytoplasm; Golgi apparatus	Putative target protein of ADP-ribosylation factor.
RARS	Arginine--tRNA ligase, cytoplasmic		6.26	95	90	1.1	94.11	-0.280	Enzyme: Ligase	Protein metabolism	Cytoplasm; Cytosol	Forms part of a macromolecular complex that catalyzes the attachment of specific amino acids to cognate tRNAs during protein synthesis. Modulates the secretion of AIMP1 and may be involved in generation of the inflammatory cytokine EMAP2 from AIMP1.
**ASL**	**Argininosuccinate lyase**	**[3]**	6.05	56	50	1.1	91.68	-0.194	Enzyme: Lyase	Metabolism; Energy pathways	Cytosol; Mitochondrion; Nucleus	Forms a cytosolic homotetramer and primarily catalyzes the reversible hydrolytic cleavage of argininosuccinate into arginine and fumarate, an essential step in the liver in detoxifying ammonia via the urea cycle.
**ASS1**	**Argininosuccinate synthase**	**[1, 3-4]**	8.08	51	53	1.0	85.66	-0.367	**Enzyme: Ligase**	**Metabolism; Energy pathways**	**Cytosol; Nucleus; Microsome**	**Is indirectly involved in the control of blood pressure.**
DARS	Aspartate--tRNA ligase, cytoplasmic		6.11	70	63	1.1	82.89	-0.416	ATPase	Metabolism; Energy pathways	Cytoplasm; Cytosol	Catalyzes the specific attachment of an amino acid to its cognate tRNA in a 2 step reaction: the amino acid (AA) is first activated by ATP to form AA-AMP and then transferred to the acceptor end of the tRNA.
**AKNA**	**AT-hook-containing transcription factor**	**[3]**	5.91	180	152	1.2	59.31	-0.789	**Transcription factor**	**Regulation of nucleobase, nucleoside, nucleotide and nucleic acid metabolism**	**Nucleus**	**Transcription factor that specifically activates the expression of the CD40 receptor and its ligand CD40L/CD154, two cell surface molecules on lymphocytes that are critical for antigen-dependent-B-cell development. Binds to A/T-rich promoters.**
**ATP5A1**	**ATP synthase subunit alpha, mitochondrial**	**[3]**	8.28	60	62	1.0	98.69	-0.106	Transport/cargo protein	Metabolism; Energy pathways	Mitochondrion; Extracellular; Zymogen granule	Mitochondrial membrane ATP synthase (F1F0 ATP synthase or Complex V) produces ATP from ADP in the presence of a proton gradient across the membrane which is generated by electron transport complexes of the respiratory chain.Subunit alpha does not bear the catalytic high-affinity ATP-binding sites By similarity.
**ATP5B**	**ATP synthase subunit beta, mitochondrial**	**[1]**	5.00	63	44	1.4	98.13	-0.020	Transport/cargo protein	Metabolism; Energy pathways	Mitochondrion; Plasma membrane; Nucleolus; Extracellular; Zymogen granule	Mitochondrial membrane ATP synthase (F1F0 ATP synthase or Complex V) produces ATP from ADP in the presence of a proton gradient across the membrane which is generated by electron transport complexes of the respiratory chain.Subunits alpha and beta form the catalytic core in F1. Rotation of the central stalk against the surrounding alpha3beta3 subunits leads to hydrolysis of ATP in three separate catalytic sites on the beta subunits.
ATAD5	ATPase family AAA domain-containing protein 5		9.27	244	309	0.8	72.33	-0.754	**ATP binding**	**DNA repair**	**Nucleus**	**Involved in DNA damage response. Involved in a RAD9A-related damage checkpoint, a pathway that is important in determining whether DNA damage is compatible with cell survival or whether it requires cell elimination by apoptosis.**
**ABCD4**	**ATP-binding cassette sub-family D member 4**	**[1]**	6.12	62	57	1.1	102.77	0.051	Transport/cargo protein	Transport	Peroxisomal membrane	May be involved in intracellular processing of vitamin B12 (cobalamin). Could play a role in the lysosomal release of vitamin B12 into the cytoplasm.
ABCF1	ATP-binding cassette sub-family F member 1		6.40	151	146	1.0	71.72	-0.954	Translation regulatory protein	Protein metabolism	Ribosome; Nucleus; Cytoplasm	Isoform 2 is required for efficient Cap- and IRES-mediated mRNA translation initiation. Isoform 2 is not involved in the ribosome biogenesis.
PFKL	ATP-dependent 6-phosphofructokinase, liver type		7.35	84	84	1.0	88.88	-0.083	Enzyme: Phosphotransferase	Metabolism; Energy pathways	Cytoplasm	Catalyzes the phosphorylation of D-fructose 6-phosphate to fructose 1,6-bisphosphate by ATP, the first committing step of glycolysis.
PFKM	ATP-dependent 6-phosphofructokinase, muscle type		8.23	89	93	1.0	85.49	-0.174	Enzyme: Phosphotransferase	Metabolism; Energy pathways	Cytoplasm	Catalyzes the phosphorylation of D-fructose 6-phosphate to fructose 1,6-bisphosphate by ATP, the first committing step of glycolysis.
PFKP	ATP-dependent 6-phosphofructokinase, platelet type		7.50	89	90	1.0	87.97	-0.146	Enzyme: Phosphotransferase	Metabolism; Energy pathways	Cytoplasm	Catalyzes the phosphorylation of D-fructose 6-phosphate to fructose 1,6-bisphosphate by ATP, the first committing step of glycolysis.
DHX9	ATP-dependent RNA helicase A		6.41	147	139	1.1	81.39	-0.339	Transcription factor	Regulation of nucleobase, nucleoside, nucleotide and nucleic acid metabolism	Cytoplasm; Nucleus; Nucleolus; Cytoplasmic vesicle; Integral to membrane	Unwinds double-stranded DNA and RNA in a 3' to 5' direction.Functions as a transcriptional activator. Component of the CRD-mediated complex that promotes MYC mRNA stability.
DDX1	ATP-dependent RNA helicase DDX1		6.80	95	92	1.0	81.00	-0.396	RNA binding protein	Regulation of nucleobase, nucleoside, nucleotide and nucleic acid metabolism	Nucleus; Nucleolus	Acts as an ATP-dependent RNA helicase, able to unwind both RNA-RNA and RNA-DNA duplexes. Possesses 5' single-stranded RNA overhang nuclease activity. Possesses ATPase activity on various RNA, but not DNA polynucleotides.
DDX3X	ATP-dependent RNA helicase DDX3X		6.73	89	87	1.0	65.22	-0.638	RNA helicase	Regulation of nucleobase, nucleoside, nucleotide and nucleic acid metabolism	Nucleus; Cytoplasm; Nucleolus	Multifunctional ATP-dependent RNA helicase.Found associated with the E-cadherin promoter and can down-regulate transcription from the promoter. Involved in regulation of translation initiation. Proposed to be involved in stress response and stress granule assembly.
DDX3Y	ATP-dependent RNA helicase DDX3Y		7.23	88	88	1.0	66.59	-0.639	**RNA helicase**	**Regulation of nucleobase, nucleoside, nucleotide and nucleic acid metabolism**	**Nucleus; Cytoplasm**	**Probable ATP-dependent RNA helicase. May play a role in spermatogenesis.**
**AZU1**	**Azurocidin**	**[1, 4, 6, 8]**	9.53	15	23	0.7	75.95	-0.249	Secreted polypeptide	Immune response	Cytoplasmic vesicle; Extracellular; Plasma membrane; Nucleus; Mitochondrion	This is a neutrophil granule-derived antibacterial and monocyte- and fibroblast-specific chemotactic glycoprotein. Binds heparin. The cytotoxic action is limited to many species of Gram-negative bacteria.
SLC4A1	Band 3 anion transport protein		5.08	106	74	1.4	109.03	0.213	Membrane transport protein	Transport	Plasma membrane; Nucleus; Golgi apparatus; Mitochondrion	Functions both as a transporter that mediates electroneutral anion exchange across the cell membrane and as a structural protein. Mediates chloride-bicarbonate exchange in the kidney, and is required for normal acidification of the urine.
**EPB41L1**	**Band 4.1-like protein 1**	**[3]**	5.43	148	120	1.2	66.03	-0.796	Cytoskeletal associated protein	Cell growth and/or maintenance	Plasma membrane; Nucleus; Cytoplasm; Cytoskeleton	May function to confer stability and plasticity to neuronal membrane via multiple interactions, including the spectrin-actin-based cytoskeleton, integral membrane channels and membrane-associated guanylate kinases.
BANF1	Barrier-to-autointegration factor		5.81	15	14	1.1	74.49	-0.366	DNA binding protein	DNA integration	Nucleus; Nucleolus	Plays fundamental roles in nuclear assembly, chromatin organization, gene expression and gonad development. May potently compress chromatin structure and be involved in membrane recruitment and chromatin decondensation during nuclear assembly. C

**HSPG2**	**Basement membrane-specific heparan sulfate proteoglycan core protein**	[2, 11]	6.03	411	326	1.3	72.81	-0.302	Extracellular matrix protein	Cell growth and/or maintenance	Extracellular	Integral component of basement membranes. Component of the glomerular basement membrane (GBM), responsible for the fixed negative electrostatic membrane charge, and which provides a barrier which is both size- and charge-selective. It serves as an attachment substrate for cells. Plays essential roles in vascularization. Blocks endothelial cell adhesion to fibronectin and type I collagen.
**BCL6B**	**B-cell CLL/lymphoma 6 member B protein**	[1]	9.23	35	53	0.7	65.64	-0.470	Transcription regulatory protein	Cell communication; Signal transduction	Nucleus	Acts as a sequence-specific transcriptional repressor in association with BCL6. May function in a narrow stage or be related to some events in the early B-cell development.
BCL9L	B-cell CLL/lymphoma 9-like protein		8.79	99	106	0.9	52.41	-0.689	Beta catenin-binding	Regulation of cell morphogenesis	Nucleus; Cytoplasm	Transcriptional regulator that acts as an activator. Promotes beta-catenin transcriptional activity. Plays a role in tumorigenesis. Enhances the neoplastic transforming activity of CTNNB1 By similarity.
**APOH**	**Beta-2-glycoprotein 1**	[1]	8.37	34	40	0.9	57.12	-0.411	Transport/cargo protein	Transport	Extracellular; Plasma membrane; Cytoplasm	Binds to various kinds of negatively charged substances such as heparin, phospholipids, and dextran sulfate. May prevent activation of the intrinsic blood coagulation cascade by binding to phospholipids on the surface of damaged cells.
**B2M**	**Beta-2-microglobulin**	[1]	6.07	15	13	1.2	69.80	-0.742	MHC complex protein	Immune response	Immune response	Component of the class I major histocompatibility complex (MHC). Involved in the presentation of peptide antigens to the immune system.
ACTBL2	Beta-actin-like protein 2		5.39	48	38	1.3	83.78	-0.190	Cytoskeletal protein; ATP binding	Cell growth and/or maintenance	Cytoplasm, Cytoskeleton	Actins are highly conserved proteins that are involved in various types of cell motility and are ubiquitously expressed in all eukaryotic cells.
DEFB1	Beta-defensin 1		8.87	1	5	0.2	46.11	-0.272	Defensin	Immune response	Cytoplasm; Extracellular	Has bactericidal activity.
EPRS	Bifunctional glutamate/proline--tRNA ligase		7.02	209	207	1.0	78.87	-0.517	Enzyme: Ligase	Protein metabolism	Cytoplasm; Nucleus	Catalyzes the attachment of the cognate amino acid to the corresponding tRNA in a two-step reaction: the amino acid is first activated by ATP to form a covalent intermediate with AMP and is then transferred to the acceptor end of the cognate tRNA.
ATIC	Bifunctional purine biosynthesis protein PURH		6.27	70	65	1.1	93.94	-0.108	Enzyme: Hydrolase	Metabolism; Energy pathways	Cytosol; Membrane; Extracellular vesicular exosome	Bifunctional enzyme that catalyzes 2 steps in purine biosynthesis.
**BGN**	**Biglycan**	[8]	8.13	37	39	0.9	98.91	-0.298	Extracellular matrix protein	Cell growth and/or maintenance	Extracellular	May be involved in collagen fiber assembly.
**CEL**	**Bile salt-activated lipase**	[4]	5.08	74	57	1.3	68.16	-0.334	Enzyme: Lipase	Metabolism; Energy pathways	Endoplasmic reticulum; Extracellular; Golgi apparatus	Catalyzes fat and vitamin absorption. Acts in concert with pancreatic lipase and colipase for the complete digestion of dietary triglycerides.
BOD1L1	Biorientation of chromosomes in cell division protein 1-like 1		5.00	577	415	1.4	59.77	-0.990	Unclassified	Unknown	Nucleus; Cytoplasm	Dna binding
**PRG2**	**Bone marrow proteoglycan**	[4]	6.11	27	24	1.1	63.40	-0.585	Unclassified	Immune response	Extracellular	Cytotoxin and helminthotoxin. Also induces non-cytolytic histamine release from human basophils. Involved in antiparasitic defense mechanisms and immune hypersensitivity reactions. The proform acts as a proteinase inhibitor, reducing the activity of PAPPA.
BAI3	Brain-specific angiogenesis inhibitor 3		6.58	159	151	1.1	77.04	-0.307	Integral membrane protein	Cell communication; Signal transduction	Plasma membrane	May regulate the number of excitatory synapses that are formed on hippocampus neurons. Has no effect on inhibitory synapses. Might be involved in angiogenesis inhibition and suppression of glioblastoma.
**BRCA1**	**Breast cancer type 1 susceptibility protein**	[1]	5.29	283	213	1.3	69.01	-0.785	Transcription regulatory protein	Regulation of gene expression, epigenetic	Nucleus; Cytoplasm; Centrosome; Perinuclear region; Mitochondrion	E3 ubiquitin-protein ligase that specifically mediates the formation of 'Lys-6'-linked polyubiquitin chains and plays a central role in DNA repair by facilitating cellular responses to DNA damage.
MTHFD1	C-1-tetrahydrofolate synthase, cytoplasmic		8.66	108	117	0.9	99.30	-0.055	Enzyme: Dehydrogenase	Metabolism; Energy pathways	Cytoplasm; Cytosol; Mitochondrion	5,10-methylenetetrahydrofolate + NADP+ = 5,10-methenyltetrahydrofolate + NADPH. 5,10-methenyltetrahydrofolate + H2O = 10-formyltetrahydrofolate. ATP + formate + tetrahydrofolate = ADP + phosphate + 10-formyltetrahydrofolate.
**C4BPA**	**C4b-binding protein alpha chain**	[1, 4]	6.24	63	57	1.1	61.38	-0.532	Complement protein	Immune response	Extracellular	Controls the classical pathway of complement activation. It binds as a cofactor to C3b/C4b inactivator (C3bINA), which then hydrolyzes the complement fragment C4b. It also accelerates the degradation of the C4bC2a complex (C3 convertase) by dissociating the complement fragment C2a. Alpha chain binds C4b. It interacts also with anticoagulant protein S and with serum amyloid P component.
C4BPB	C4b-binding protein beta chain		4.97	34	22	1.5	71.74	-0.413	Regulatory/other subunit	Immune response; Regulation of coagulation	Extracellular	Controls the classical pathway of complement activation. It binds as a cofactor to C3b/C4b inactivator (C3bINA), which then hydrolyzes the complement fragment C4b. It also accelerates the degradation of the C4bC2a complex (C3 convertase) by dissociating the complement fragment C2a. It also interacts with anticoagulant protein S and with serum amyloid P component. The beta chain binds protein S.
CDH1	Cadherin 1, type 1, E-cadherin (Epithelial)		4.23	117	53	2.2	87.03	-0.318	Adhesion molecule	Cell communication; Signal transduction	Plasma membrane; Cytoplasm; Extracellular; Cell junction	Cadherins are calcium-dependent cell adhesion proteins. They preferentially interact with themselves in a homophilic manner in connecting cells; cadherins may thus contribute to the sorting of heterogeneous cell types. CDH1 is involved in mechanisms regulating cell-cell adhesions, mobility and proliferation of epithelial cells. Has a potent invasive suppressor role. It is a ligand for integrin alpha-E/beta-7.
**CDH11**	**Cadherin-11**	[2]	4.53	120	67	1.8	83.06	-0.397	Cell junction protein	Cell growth and/or maintenance	Extracellular; Plasma membrane; Cytoplasm	Cadherins are calcium-dependent cell adhesion proteins. They preferentially interact with themselves in a homophilic manner in connecting cells; cadherins may thus contribute to the sorting of heterogeneous cell types.
CDH15	Cadherin-15		4.64	112	65	1.7	89.92	-0.354	Adhesion molecule	Cell growth and/or maintenance	Plasma membrane	Cadherins are calcium-dependent cell adhesion proteins. They preferentially interact with themselves in a homophilic manner in connecting cells; cadherins may thus contribute to the sorting of heterogeneous cell types. M-cadherin is part of the myogenic program and may provide a trigger for terminal muscle differentiation.
CAMK2D	Calcium/calmodulin-dependent protein kinase (CaM kinase) II delta		6.81	63	61	1.0	81.31	-0.405	Serine/threonine kinase	Serine/threonine kinase	Cytoplasm; Nucleus	Calcium/calmodulin-dependent protein kinase involved in the regulation of Ca2+ homeostatis and excitation-contraction coupling (ECC) in heart by targeting ion channels, transporters and accessory proteins involved in Ca2+ influx into the myocyte, Ca2+ release from the sarcoplasmic reticulum (SR), SR Ca2+ uptake and Na+ and K+ channel transport.
**KCNMB2**	**Calcium-activated potassium channel subunit beta-2**	[3]	8.71	20	26	0.8	87.49	-0.086	Transport/cargo protein	Transport	Endoplasmic reticulum; Plasma membrane	Regulatory subunit of the calcium activated potassium KCNMA1 (maxiK) channel. Modulates the calcium sensitivity and gating kinetics of KCNMA1, thereby contributing to KCNMA1 channel diversity. Acts as a negative regulator that confers rapid and complete inactivation of KCNMA1 channel complex.
CAPS	Calcyphosin		4.74	33	24	1.4	74.39	-0.448	Calcium binding protein	Cell communication; Signal transduction	Cytoplasm	Calcium-binding protein. May play a role in cellular signaling events.
**CALM1**	**Calmodulin**	[1, 10]	4.09	38	14	2.7	65.95	-0.671	Calcium binding protein	Cell communication; Signal transduction	Cytoplasm; Nucleus; Plasma membrane	Calmodulin mediates the control of a large number of enzymes, ion channels, aquaporins and other proteins by Ca2+. Among the enzymes to be stimulated by the calmodulin-Ca2+ complex are a number of protein kinases and phosphatases. Together with CCP110 and centrin, is involved in a genetic pathway that regulates the centrosome cycle and progression through cytokinesis.
**CANX**	**Calnexin**	[4]	4.46	135	74	1.8	63.25	-0.954	Chaperone	Protein folding	Endoplasmic reticulum; Nuclear membrane; Endoplasmic reticulum membrane; Plasma membrane; Golgi apparatus; Lysosome; Mitochondrion	Calcium-binding protein that interacts with newly synthesized glycoproteins in the endoplasmic reticulum. It may act in assisting protein assembly and/or in the retention within the ER of unassembled protein subunits. It seems to play a major role in the quality control apparatus of the ER by the retention of incorrectly folded proteins.
CAPNS1	Calpain small subunit 1		5.15	30	22	1.4	73.71	-0.216	Regulatory/other subunit	Protein folding	Cytoplasm; Plasma membrane; Nucleus	Regulatory subunit of the calcium-regulated non-lysosomal thiol-protease which catalyzes limited proteolysis of substrates involved in cytoskeletal remodeling and signal transduction.
CAPN1	Calpain-1 catalytic subunit		5.49	102	89	1.1	81.61	-0.360	Cysteine protease	Protein metabolism	Cytoplasm; Plasma membrane; Nucleus	Calcium-regulated non-lysosomal thiol-protease which catalyze limited proteolysis of substrates involved in cytoskeletal remodeling and signal transduction.
**CALR**	**Calreticulin**	[9]	4.29	109	50	2.2	51.90	-1.254	Chaperone	Protein metabolism	Endoplasmic reticulum; Nucleus; Extracellular; Cytoplasm; Plasma membrane; Sarcoplasmic reticulum; Mitochondrion	Calcium-binding chaperone that promotes folding, oligomeric assembly and quality control in the endoplasmic reticulum (ER) via the calreticulin/calnexin cycle. This lectin interacts transiently with almost all of the monoglucosylated glycoproteins that are synthesized in the ER
**CASQ2**	**Calsequestrin-2**	[4]	4.14	102	40	2.6	85.66	-0.567	Calcium binding protein	Cell communication; Signal transduction	Sarcoplasmic reticulum; Plasma membrane	Calsequestrin is a high-capacity, moderate affinity, calcium-binding protein and thus acts as an internal calcium store in muscle. Calcium ions are bound by clusters of acidic residues at the protein surface, especially at the interface between subunits. Can bind around 60 Ca2+ ions. Regulates the release of lumenal Ca2+ via the calcium release channel RYR2; this plays an important role in triggering muscle contraction.
**PRKAR2A**	**cAMP-dependent protein kinase type II-alpha regulatory subunit**	[3]	4.96	69	51	1.4	84.14	-0.502	Serine/threonine kinase	Cell communication; Signal transduction	Cytoplasm; Nucleus	Regulatory subunit of the cAMP-dependent protein kinases involved in cAMP signaling in cells. Type II regulatory chains mediate membrane association by binding to anchoring proteins, including the MAP2 kinase.
**CLIP2**	**CAP-Gly domain-containing linker protein 2**	[3]	6.29	168	157	1.1	82.10	-0.728	Cytoskeletal protein	Cell communication; Signal transduction	Cytoplasm	Seems to link microtubules to dendritic lamellar body (DLB), a membranous organelle predominantly present in bulbous dendritic appendages of neurons linked by dendrodendritic gap junctions. May operate in the control of brain-specific organelle translocations (By similarity).
**CA1**	**Carbonic anhydrase 1**	[1, 5, 8]	6.63	27	25	1.1	71.27	-0.546	Enzyme: Carbonic anhydrase	Metabolism; Energy pathways	Cytoplasm	Reversible hydration of carbon dioxide. Can hydrate cyanamide to urea.
CA2	Carbonic anhydrase 2		6.86	32	31	1.0	76.76	-0.588	Enzyme: Carbonic anhydrase	Metabolism; Energy pathways	Cytoplasm; Plasma membrane; Golgi apparatus; Nucleus; Extracellular	Essential for bone resorption and osteoclast differentiation By similarity. Reversible hydration of carbon dioxide. Can hydrate cyanamide to urea. Involved in the regulation of fluid secretion into the anterior chamber of the eye. Contributes to intracellular pH regulation in the duodenal upper villous epithelium during proton-coupled peptide absorption. Stimulates the chloride-bicarbonate exchange activity of SLC26A6.
CBR1	Carbonyl reductase [NADPH] 1		8.55	32	35	0.9	91.09	-0.219	Enzyme: Oxidoreductase	Metabolism; Energy pathways	Cytoplasm	NADPH-dependent reductase with broad substrate specificity. Catalyzes the reduction of a wide variety of carbonyl compounds including quinones, prostaglandins, menadione, plus various xenobiotics. Can convert prostaglandin E2 to prostaglandin F2-alpha. Can bind glutathione, which explains its higher affinity for glutathione-conjugated substrates. Catalyzes the reduction of S-nitrosoglutathione.
CMBL	Carboxymethylenebutenolidase homolog		6.78	30	29	1.0	84.67	-0.328	Enzyme: Hydrolase	Metabolism	Cytoplasm; Extracellular vesicular exosome	Cysteine hydrolase. Can convert the prodrug olmesartan medoxomil into its pharmacologically active metabolite olmerstatan, an angiotensin receptor blocker, in liver and intestine. May also activate beta-lactam antibiotics faropenem medoxomil and lenampicillin.
CPB2	Carboxypeptidase B2		8.11	32	34	0.9	77.67	-0.358	Carboxypeptidase	Protein metabolism	Extracellular	Cleaves C-terminal arginine or lysine residues from biologically active peptides such as kinins or anaphylatoxins in the circulation thereby regulating their activities. Down-regulates fibrinolysis by removing C-terminal lysine residues from fibrin that has already been partially degraded by plasmin.
**CPE**	**Carboxypeptidase E**	[2]	4.85	65	40	1.6	76.84	-0.506	Carboxypeptidase	Protein metabolism	Plasma membrane; Cytoplasmic vesicle; Endoplasmic reticulum	Removes residual C-terminal Arg or Lys remaining after initial endoprotease cleavage during prohormone processing. Processes proinsulin.
CPQ	Carboxypeptidase Q		5.52	48	37	1.3	94.53	-0.071	Carboxypeptidase	Protein metabolism	Extracellular; Secretory granule	Carboxypeptidase that may play an important role in the hydrolysis of circulating peptides. Catalyzes the hydrolysis of dipeptides with unsubstituted terminals into amino acids. May play a role in the liberation of thyroxine hormone from its thyroglobulin (Tg) precursor.
**CEA**	**Carcinoembryonic antigen**	[5]	5.20	10	7	1.4	91.96	-0.277	-	-	-	Carcinoembryonic antigen (CEA) describes a set of highly related glycoproteins involved in cell adhesion. CEA is normally produced in gastrointestinal tissue during fetal development, but the production stops before birth. Therefore CEA is usually present only at very low levels in the blood of healthy
**CEACAM5**	**Carcinoembryonic antigen-related cell adhesion molecule 5**	[1]	5.44	52	40	1.3	80.25	-0.411	Adhesion molecule	Immune response	Plasma membrane	Cell surface glycoprotein that plays a role in cell adhesion and in intracellular signaling. Receptor for E.coli Dr adhesins.
CEACAM6	Carcinoembryonic antigen-related cell adhesion molecule 6		5.09	23	16	1.4	78.04	-0.400	Adhesion molecule	Cell communication; Signal transduction	Plasma membrane; Cytoplasm	Carcinoembryonic antigen (CEA; MIM 114890) is one of the most widely used tumor markers in serum immunoassay determinations of carcinoma. An apparent lack of absolute cancer specificity for CEA probably results in part from the presence in normal and neoplastic tissues of antigens that share antigenic determinants with the 180-kD form of CEA.
**CEACAM8**	**Carcinoembryonic antigen-related cell adhesion molecule 8**	[1]	5.74	23	19	1.2	74.62	-0.508	Unclassified	Immune response	Plasma membrane	Carcinoembryonic antigen (CEA; MIM 114890) is one of the most widely used tumor markers in serum immunoassay determinations of carcinoma. An apparent lack of absolute cancer specificity for CEA probably results in part from the presence in normal and neoplastic tissues of antigens that share antigenic determinants with the 180-kD form of CEA.
COMP	Cartilage oligomeric matrix protein		4.37	131	67	2.0	54.60	-0.792	Extracellular matrix protein	Cell growth and/or maintenance	Extracellular	May play a role in the structural integrity of cartilage via its interaction with other extracellular matrix proteins such as the collagens and fibronectin.
CAT	Catalase		6.95	61	59	1.0	68.42	-0.590	Enzyme: Oxidoreductase	Metabolism; Energy pathways	Cytoplasm; Mitochondrion; Peroxisome	Occurs in almost all aerobically respiring organisms and serves to protect cells from the toxic effects of hydrogen peroxide. Promotes growth of cells including T-cells, B-cells, myeloid leukemia cells, melanoma cells, mastocytoma cells and normal and transformed fibroblast cells.
COMT	Catechol O-methyltransferase		5.26	34	24	1.4	114.39	0.160	Enzyme: Acyltransferase	Metabolism; Energy pathways	Endoplasmic reticulum; Cytoplasm	Catalyzes the O-methylation, and thereby the inactivation, of catecholamine neurotransmitters and catechol hormones. Also shortens the biological half-lives of certain neuroactive drugs, like L-DOPA, alpha-methyl DOPA and isoproterenol.
CTNNA1	Catenin alpha-1		5.95	132	123	1.1	91.14	-0.370	Cytoskeletal protein	Cell growth and/or maintenance	Cytoplasm; Plasma membrane; Cell junction	Associates with the cytoplasmic domain of a variety of cadherins. The association of catenins to cadherins produces a complex which is linked to the actin filament network, and which seems to be of primary importance for cadherins cell-adhesion properties. Can associate with both E- and N-cadherins. May play a crucial role in

CTNND1	Catenin delta-1		5.86	144	127	1.1	76.16	-0.728	Adhesion molecule	Cell communication; Signal transduction	Cytoplasm; Nucleus	Binds to and inhibits the transcriptional repressor ZBTB33, which may lead to activation of target genes of the Wnt signaling pathway By similarity. Associates with and regulates the cell adhesion properties of both C-, E- and N-cadherins, being critical for their surface stability.
CAMP	Cathelicidin antimicrobial peptide		9.48	22	30	0.7	85.61	-0.429	Secreted polypeptide	Immune response	Secretory granule; Extracellular	Binds to bacterial lipopolysaccharides (LPS), has antibacterial activity.
CTSB	Cathepsin B		5.22	30	19	1.6	60.63	-0.406	Cysteine protease	Protein metabolism; Apoptosis	Cytoplasm; Plasma membrane; Extracellular; Perinuclear region; Mitochondrion; Lysosome; Nuclear membrane; Endoplasmic reticulum; Zymogen granule	Thiol protease which is believed to participate in intracellular degradation and turnover of proteins. Has also been implicated in tumor invasion and metastasis.
**CTSD**	**Cathepsin D**	[1, 10]	5.60	34	29	1.2	87.36	-0.049	Aspartic protease	Protein metabolism	Lysosome; Extracellular; Golgi apparatus; Endoplasmic reticulum; Mitochondrion	Thiol protease which is believed to participate in intracellular degradation and turnover of proteins. Has also been implicated in tumor invasion and metastasis.
**CTSG**	**Cathepsin G**	[4-8]	11.37	16	38	0.4	78.38	-0.562	Serine protease	Protein metabolism	Plasma membrane; Cytoplasm; Lysosome; Golgi apparatus; Extracellular	Serine protease with trypsin- and chymotrypsin-like specificity. Cleaves complement C3. Has antibacterial activity against the Gram-negative bacterium P.aeruginosa, antibacterial activity is inhibited by LPS from P.aeruginosa, Z-Gly-Leu-Phe-CH2Cl and phenylmethylsulfonyl fluoride.
CTSL	Cathepsin L1		4.43	26	13	2.0	59.60	-0.443	Cysteine protease	Protein metabolism	Endosome; Lysosome	Important for the overall degradation of proteins in lysosomes.
CTSZ	Cathepsin Z		5.48	28	21	1.3	63.72	-0.545	Cysteine protease	Protein metabolism	Lysosome	Exhibits carboxy-monopeptidase as well as carboxy-dipeptidase activity.
CD44	CD44 antigen		5.10	95	53	1.8	52.98	-0.805	Cell surface receptor	Cell communication; Signal transduction	Plasma membrane; Cytoplasm; Nucleus	Receptor for hyaluronic acid (HA). Mediates cell-cell and cell-matrix interactions through its affinity for HA, and possibly also through its affinity for other ligands such as osteopontin, collagens, and matrix metalloproteinases (MMPs). Adhesion with HA plays an important role in cell migration, tumor growth and progression.
CD55	CD55 antigen, decay accelerating factor for complement (Cromer blood group)		5.99	28	25	1.1	72.52	-0.259	Complement protein	Immune response	Plasma membrane; Extracellular	This protein recognizes C4b and C3b fragments that condense with cell-surface hydroxyl or amino groups when nascent C4b and C3b are locally generated during C4 and c3 activation. Interaction of daf with cell-associated C4b and C3b polypeptides interferes with their ability to catalyze the conversion of C2 and factor B to enzymatically active C2a and Bb and thereby prevents the formation of C4b2a and C3bBb, the amplification convertases of the complement cascade.
**CD59**	**CD59 glycoprotein**	[1]	5.46	11	8	1.4	72.22	-0.146	Cell surface receptor	Immune response	Extracellular; Plasma membrane; Cytoplasm	Potent inhibitor of the complement membrane attack complex (MAC) action. Acts by binding to the C8 and/or C9 complements of the assembling MAC, thereby preventing incorporation of the multiple copies of C9 required for complete formation of the osmolytic pore. This inhibitor appears to be species-specific. Involved in signal transduction for T-cell activation complexed to a protein tyrosine kinase. The soluble form from urine retains its specific complement binding activity, but exhibits greatly reduced ability to inhibit MAC assembly on cell membranes.
**CD63**	**CD63 antigen**	[1]	8.17	16	19	0.8	112.28	0.762	Integral membrane protein	Cell communication; Signal transduction	Plasma membrane; Lysosome; Endosome; Cytoplasm; Cell surface	Functions as cell surface receptor for TIMP1 and plays a role in the activation of cellular signaling cascades. Plays a role in the activation of ITGB1 and integrin signaling, leading to the activation of AKT, FAK/PTK2 and MAP kinases. Promotes cell survival, reorganization of the actin cytoskeleton, cell adhesion, spreading and migration, via its role in the activation of AKT and FAK/PTK2. Plays a role in VEGFA signaling via its role in regulating the internalization of KDR/VEGFR2. Plays a role in intracellular vesicular transport processes.
**CD82**	**CD82 antigen**	[1]	5.14	25	19	1.3	101.80	0.378	Integral membrane proteinCell surface receptor	Cell communication; Signal transduction	Plasma membrane	Associates with CD4 or CD8 and delivers costimulatory signals for the TCR/CD3 pathway.
CISD1	CDGSH iron-sulfur domain-containing protein 1		9.20	12	17	0.7	76.64	-0.419	1. 2 iron, 2 sulfur cluster binding	Regulation of cellular respiration	Mitochondrial outer membrane; Single-pass type III membrane protein	Plays a key role in regulating maximal capacity for electron transport and oxidative phosphorylation By similarity. May be involved in Fe-S cluster shuttling and/or in redox reactions.
CEND1	Cell cycle exit and neuronal differentiation protein 1		9.24	15	19	0.8	63.09	-0.548	Integral membrane protein	Cell growth and/or maintenance	Mitochondrion	Involved in neuroblastoma cell differentiation.
CDC42	Cell division control protein 42 homolog		6.16	24	23	1.0	85.48	-0.222	GTPase	Cell communication; Signal transduction	Cell junction; Cytoplasm; Plasma membrane; Extracellular; Microtubule cytoskeleton; Apical membrane; Cytosol; Cell projection	Probably involved in the organization of the actin cytoskeleton by acting downstream of CDC42, inducing actin filament assembly. Alters CDC42-induced cell shape changes. In activated T-cells, may play a role in CDC42-mediated F-actin accumulation at the immunological synapse. May play a role in early contractile events in phagocytosis in macrophages.
**CEP192**	**Centrosomal protein of 192 kDa**	[1]	6.09	228	205	1.1	82.95	-0.369	Cytoskeletal protein	Cell growth and/or maintenance	Centrosome	Required for mitotic centrosome and spindle assembly. Appears to be a major regulator of pericentriolar material (PCM) recruitment, centrosome maturation, and centriole duplication.
**CP**	**Ceruloplasmin**	[1-2, 6, 8]	5.49	135	100	1.4	72.02	-0.550	Enzyme: Oxidoreductase	Metabolism; Energy pathways	Endoplasmic reticulum; Golgi apparatus; Extracellular; Plasma membrane	Ceruloplasmin is a blue, copper-binding (6-7 atoms per molecule) glycoprotein. It has ferroxidase activity oxidizing Fe2+ to Fe3+ without releasing radical oxygen species. It is involved in iron transport across the cell membrane. Provides Cu2+ ions for the ascorbate-mediated deaminase degradation of the heparan sulfate chains of GPC1.
CCT7	Chaperonin containing TCP1, subunit 7 (Eta)		7.55	67	68	1.0	95.43	-0.098	Chaperone	Protein folding	Cytoplasm; Microtubule; Mitochondrion	Molecular chaperone; assists the folding of proteins upon ATP hydrolysis. Known to play a role, in vitro, in the folding of actin and tubulin By similarity.
CLIC1	Chloride intracellular channel protein 1		5.09	35	27	1.3	89.00	-0.293	Intracellular ligand gated channel	Transport	Cytoplasm; Nucleus; Plasma membrane	Can insert into membranes and form chloride ion channels. Channel activity depends on the pH. Membrane insertion seems to be redox-regulated and may occur only under oxydizing conditions. Involved in regulation of the cell cycle.
**CSPG4**	**Chondroitin sulfate proteoglycan 4**	[2]	5.24	257	173	1.5	89.79	-0.167	Integral membrane protein	Cell communication; Signal transduction	Plasma membrane	Proteoglycan playing a role in cell proliferation and migration which stimulates endothelial cells motility during microvascular morphogenesis. May also inhibit neurite outgrowth and growth cone collapse during axon regeneration. Cell surface receptor for collagen alpha 2(VI) which may confer cells ability to migrate on that substrate. Binds through its extracellular N-terminus growth factors, extracellular matrix proteases modulating their activity.
CBX3	Chromobox protein homolog 3		5.23	38	33	1.2	60.60	-0.982	DNA binding protein	Regulation of gene expression, epigenetic	Nucleus; Nucleolus; Cytoplasm	Seems to be involved in transcriptional silencing in heterochromatin-like complexes. Recognizes and binds histone H3 tails methylated at 'Lys-9', leading to epigenetic repression. May contribute to the association of the heterochromatin with the inner nuclear membrane through its interaction with lamin B receptor (LBR). Involved in the formation of functional kinetochore through interaction with MIS12 complex proteins.
CLTC	Clathrin heavy chain 1		5.48	219	178	1.2	93.86	-0.244	Structural protein	Cell growth and/or maintenance	Cytoplasm; Plasma membrane; Golgi apparatus; Endosome; Microtubule; Mitochondrion	Clathrin is the major protein of the polyhedral coat of coated pits and vesicles. Two different adapter protein complexes link the clathrin lattice either to the plasma membrane or to the trans-Golgi network.
**CSTF3**	**Cleavage stimulation factor subunit 3**	[1]	8.26	103	107	1.0	84.73	-0.484	RNA binding protein	RNA metabolism	Nucleus; Cytoplasm	One of the multiple factors required for polyadenylation and 3'-end cleavage of mammalian pre-mRNAs.
**CLU**	**Clusterin**	[1, 3-5, 8]	5.89	66	57	1.2	68.22	-0.761	Complement protein	Immune response	Extracellular; Nucleus; Cytoplasm; Zymogen granule	Isoform 1 functions as extracellular chaperone that prevents aggregation of nonnative proteins. Prevents stress-induced aggregation of blood plasma proteins. Inhibits formation of amyloid fibrils by APP, APOC2, B2M, CALCA, CSN3, SNCA and aggregation-prone LYZ variants (in vitro). Does not require ATP. Maintains partially unfolded proteins in a state appropriate for subsequent refolding by other chaperones, such as HSPA8/HSC70.
**F9**	**Coagulation factor IX**	[3]	5.20	58	43	1.3	68.51	-0.437	Coagulation factor	Protein metabolism	Extracellular; Endoplasmic reticulum; Golgi apparatus	Factor IX is a vitamin K-dependent plasma protein that participates in the intrinsic pathway of blood coagulation by converting factor X to its active form in the presence of Ca2+ ions, phospholipids, and factor VIIIa.
F5	Coagulation factor V		5.68	275	218	1.3	72.91	-0.613	Coagulation factor	Protein metabolism	Extracellular; Plasma membrane; Endoplasmic reticulum; Golgi apparatus	Central regulator of hemostasis. It serves as a critical cofactor for the prothrombinase activity of factor Xa that results in the activation of prothrombin to thrombin.
**F7**	**Coagulation factor VII**	[2, 11]	6.92	51	50	1.0	79.27	-0.288	Coagulation factor	Protein metabolism	Extracellular	Initiates the extrinsic pathway of blood coagulation. Serine protease that circulates in the blood in a zymogen form. Factor VII is converted to factor VIIa by factor Xa, factor XIIa, factor IXa, or thrombin by minor proteolysis. In the presence of tissue factor and calcium ions, factor VIIa then converts factor X to factor Xa by limited proteolysis. Factor VIIa will also convert factor IX to factor IXa in the presence of tissue factor and calcium.
**F10**	**Coagulation factor X**	[1, 3, 4]	5.38	69	57	1.2	57.48	-0.681	Coagulation factor	Protein metabolism	Extracellular	Factor Xa is a vitamin K-dependent glycoprotein that converts prothrombin to thrombin in the presence of factor Va, calcium and phospholipid during blood clotting.
F11	Coagulation factor XI		8.47	59	70	0.8	74.85	-0.263	Coagulation factor	Protein metabolism	Extracellular; Plasma membrane	Factor XI triggers the middle phase of the intrinsic pathway of blood coagulation by activating factor IX.
F12	Coagulation factor XII		8.04	54	59	0.9	67.63	-0.411	Coagulation factor	Protein metabolism	Extracellular	Factor XII is a serum glycoprotein that participates in the initiation of blood coagulation, fibrinolysis, and the generation of bradykinin and angiotensin. Prekallikrein is cleaved by factor XII to form kallikrein, which then cleaves factor XII first to alpha-factor XIIa and then trypsin cleaves it to beta-factor XIIa. Alpha-factor XIIa activates factor XI to factor XIa.
F13A1	Coagulation factor XIII A chain		5.81	90	79	1.1	79.96	-0.376	Coagulation factor	Metabolism; Energy pathways	Extracellular; Nucleus; Cytoplasm	Factor XIII is activated by thrombin and calcium ion to a transglutaminase that catalyzes the formation of gamma-glutamyl-epsilon-lysine cross-links between fibrin chains, thus stabilizing the fibrin clot. Also cross-link alpha-2-plasmin inhibitor, or fibronectin, to the alpha chains of fibrin.
COPB2	Coatomer protein complex, subunit beta 2 (Beta prime)		5.14	131	94	1.4	88.56	-0.308	Transport/cargo protein	Transport	Cytoplasm; Golgi apparatus; Cytoplasmic vesicle; Mitochondrion; Nucleus; Cytosol	The coatomer is a cytosolic protein complex that binds to dilysine motifs and reversibly associates with Golgi non-clathrin-coated vesicles, which further mediate biosynthetic protein transport from the ER, via the Golgi up to the trans Golgi network. Coatomer complex is required for budding from Golgi membranes, and is essential for the retrograde Golgi-to-ER transport of dilysine-tagged proteins. In mammals, the coatomer can only be recruited by membranes associated to ADP-ribosylation factors (ARFs), which are small GTP-binding proteins; the complex also influences the Golgi structural integrity, as well as the processing, activity, and
COPA	Coatomer subunit alpha		7.70	148	150	1.0	89.45	-0.286	Transport/cargo protein	Transport	Cytoplasm; Golgi apparatus; Endoplasmic reticulum; Mitochondrion	The coatomer is a cytosolic protein complex that binds to dilysine motifs and reversibly associates with Golgi non-clathrin-coated vesicles, which further mediate biosynthetic protein transport from the ER, via the Golgi up to the trans Golgi network. Coatomer complex is required for budding from Golgi membranes, and is essential for the retrograde Golgi-to-ER transport of dilysine-tagged proteins. In mammals, the coatomer can only be recruited by membranes associated to ADP-ribosylation factors (ARFs), which are small GTP-binding proteins; the complex also influences the Golgi structural integrity, as well as the processing, activity, and
COPG1	Coatomer subunit gamma-1		5.32	120	93	1.3	90.92	-0.145	Transport/cargo protein	Transport	Golgi apparatus	Required for limiting lipid storage in lipid droplets. Involved in lipid homeostasis by regulating the presence of perilipin family members PLIN2 and PLIN3 at the lipid droplet surface and promoting the association of adipocyte triglyceride lipase (PNPLA2) with the lipid droplet surface to mediate lipolysis By similarity.
COPG2	Coatomer subunit gamma-2		5.56	113	93	1.2	92.25	-0.113	Transport/cargo protein	Transport	Cytoplasm; Golgi apparatus	The coatomer is a cytosolic protein complex that binds to dilysine motifs and reversibly associates with Golgi non-clathrin-coated vesicles, which further mediate biosynthetic protein transport from the ER, via the Golgi up to the trans Golgi network. Coatomer complex is required for budding from Golgi membranes, and is essential for the retrograde Golgi-to-ER transport of dilysine-tagged proteins. In mammals, the coatomer can only be recruited by membranes associated to ADP-ribosylation factors (ARFs), which are small GTP-binding proteins; the complex also influences the Golgi structural integrity, as well as the processing, activity, and
CFL1	Cofilin-1		8.26	27	29	0.9	86.85	-0.373	Cytoskeletal associated protein	Cell growth and/or maintenance	Cytoplasm; Nucleus; Plasma membrane; Extracellular; Microsome; Cytosol; Mitochondrion	Binds to F-actin and exhibits pH-sensitive F-actin depolymerizing activity. Regulates actin cytoskeleton dynamics. Important for normal progress through mitosis and normal cytokinesis. Plays a role in the regulation of cell morphology and cytoskeletal organization. Required for the up-regulation of atypical chemokine receptor ACKR2 from endosomal compartment to cell membrane, increasing its efficiency in chemokine uptake and degradation.
CCDC6	Coiled-coil domain-containing protein 6		6.95	66	65	1.0	63.76	-0.981	Cell cycle control protein	Apoptosis	Cytoplasm; Nucleus	This gene encodes a coiled-coil domain-containing protein. The encoded protein is ubiquitously expressed and may function as a tumor suppressor.
**CCDC78**	**Coiled-coil domain-containing protein 78**	[1]	8.27	51	53	1.0	76.74	-0.684	Unclassified	Unkown	Deuterosome	Component of the deuterosome, a structure that promotes de novo centriole amplification in multiciliated cells that can generate more than 100 centrioles. Deuterosome-mediated centriole amplification occurs in terminally differentiated multiciliated cells (G1/0) and not in S phase. Essential for centriole amplification and is required for CEP152 localization to the deuterosome.
CSDE1	Cold shock domain-containing protein E1		5.88	115	102	1.1	78.23	-0.443	RNA binding protein	Regulation of gene expression, epigenetic	Cytoplasm	RNA-binding protein. Required for internal initiation of translation of human rhinovirus RNA. May be involved in translationally coupled mRNA turnover. Implicated with other RNA-binding proteins in the cytoplasmic deadenylation/translational and decay interplay of the FOS mRNA mediated by the major coding-region determinant of instability (mCRD) domain.
**COL1A1**	**Collagen alpha-1(I) chain**	[5, 8]	9.29	83	91	0.9	27.45	-0.881	Extracellular matrix protein	Cell growth and/or maintenance	Extracellular	Type I collagen is a member of group I collagen (fibrillar forming collagen).
**COL3A1**	**Collagen alpha-1(III) chain**	[12]	9.36	77	88	0.9	27.03	-0.916	Extracellular matrix protein	Cell growth and/or maintenance	Extracellular	Collagen type III occurs in most soft connective tissues along with type I collagen. Involved in regulation of cortical development. Is the major ligand of GPR56 in the developing brain and binding to GPR56 inhibits neuronal migration and activates the RhoA pathway by coupling GPR56 to GNA13 and possibly GNA12.
COL5A1	Collagen alpha-1(V) chain		4.86	193	140	1.4	41.22	-0.957	Extracellular matrix protein	Cell growth and/or maintenance	Extracellular	Type V collagen is a member of group I collagen (fibrillar forming collagen). It is a minor connective tissue component of nearly ubiquitous distribution. Type V collagen binds to DNA, heparan sulfate, thrombospondin, heparin, and insulin.
**COL6A1**	**Collagen alpha-1(VI) chain**	[1-2, 5, 8, 11, 12]	5.20	139	112	1.2	67.46	-0.553	Extracellular matrix protein	Cell growth and/or maintenance	Extracellular	Collagen VI acts as a cell-binding protein.

COL14A1	Collagen alpha-1(XIV) chain		5.10	211	156	1.4	77.19	-0.342	Extracellular matrix protein	Cell growth and/or maintenance	Extracellular	Plays an adhesive role by integrating collagen bundles. It is probably associated with the surface of interstitial collagen fibrils via COL1. The COL2 domain may then serve as a rigid arm which sticks out from the fibril and protrudes the large N-terminal globular domain into the extracellular space, where it might interact with other matrix molecules or cell surface receptors By similarity.
**COL15A1**	**Collagen alpha-1(XV) chain**	[1, 2, 11]	4.86	155	97	1.6	67.35	-0.393	Extracellular matrix protein	Cell growth and/or maintenance	Extracellular	Structural protein that stabilizes microvessels and muscle cells, both in heart and in skeletal muscle.
COL18A1	Collagen alpha-1(XVIII) chain		5.62	164	132	1.2	60.90	-0.493	**Extracellular matrix protein**	**Cell growth and/or maintenance**	**Extracellular**	**COLA18A probably plays a major role in determining the retinal structure as well as in the closure of the neural tube. Endostatin potently inhibits endothelial cell proliferation and angiogenesis. May inhibit angiogenesis by binding to the heparan sulfate proteoglycans involved in growth factor**
**COL1A2**	**Collagen alpha-2(I) chain**	[5, 8]	10.05	66	85	0.8	41.34	-0.680	Extracellular matrix protein	Cell growth and/or maintenance	Extracellular	Type I collagen is a member of group I collagen (fibrillar forming collagen).
**COL4A3**	**Collagen alpha-3(IV) chain**	[2, 12]	9.24	120	152	0.8	45.88	-0.656	**Extracellular matrix protein**	**Cell growth and/or maintenance**	**Extracellular**	**Type IV collagen is the major structural component of glomerular basement membranes (GBM), forming a 'chicken-wire' meshwork together with laminins, proteoglycans and entactin/nidogen.**
C1QA	Complement C1q subcomponent subunit A		9.34	19	26	0.7	63.32	-0.547	Complement protein	Immune response	Extracellular	C1q associates with the proenzymes C1r and C1s to yield C1, the first component of the serum complement system. The collagen-like regions of C1q interact with the Ca2+-dependent C1r2C1s2 proenzyme complex, and efficient activation of C1 takes place on interaction of the globular heads of C1q with the Fc regions of IgG or IgM antibody present in immune complexes.
**C1QB**	**Complement C1q subcomponent subunit B**	[1]	8.85	19	23	0.8	60.00	-0.468	Complement protein	Immune response	Extracellular	C1q associates with the proenzymes C1r and C1s to yield C1, the first component of the serum complement system. The collagen-like regions of C1q interact with the Ca2+-dependent C1r2C1s2 proenzyme complex, and efficient activation of C1 takes place on interaction of the globular heads of C1q with the Fc regions of IgG or IgM antibody present in immune complexes.
**C1QC**	**Complement C1q subcomponent subunit C**	[1]	8.33	15	17	0.9	65.48	-0.336	Complement protein	Immune response	Extracellular	C1q associates with the proenzymes C1r and C1s to yield C1, the first component of the serum complement system. The collagen-like regions of C1q interact with the Ca2+-dependent C1r2C1s2 proenzyme complex, and efficient activation of C1 takes place on interaction of the globular heads of C1q with the Fc regions of IgG or IgM antibody present in immune complexes.
**C1R**	**Complement C1r subcomponent**	[1]	5.76	84	70	1.2	66.44	-0.559	Complement protein	Immune response	Extracellular	C1r B chain is a serine protease that combines with C1q and C1s to form C1, the first component of the classical pathway of the complement system.
**C1S**	**Complement C1s subcomponent**	[1]	4.85	94	63	1.5	67.01	-0.425	Complement protein	Immune response	Extracellular	C1s B chain is a serine protease that combines with C1q and C1r to form C1, the first component of the classical pathway of the complement system. C1r activates C1s so that it can, in turn, activate C2 and C4.
**C2**	**Complement C2**	[1]	7.57	76	77	1.0	77.10	-0.349	Complement protein	Immune response	Extracellular	Component C2 which is part of the classical pathway of the complement system is cleaved by activated factor C1 into two fragments: C2b and C2a. C2a, a serine protease, then combines with complement factor C4b to generate the C3 or C5 convertase.
**C3**	**Complement C3**	[2-8]	6.00	213	195	1.1	86.85	-0.340	Complement protein	Immune response	Extracellular; Endosome	C3 plays a central role in the activation of the complement system. Its processing by C3 convertase is the central reaction in both classical and alternative complement pathways. After activation C3b can bind covalently, via its reactive thioester, to cell surface carbohydrates or immune aggregates.
**C4A**	**Complement C4-A**	[1-3]	6.66	192	185	1.0	87.00	-0.252	Complement protein	Immune response	Extracellular	Non-enzymatic component of C3 and C5 convertases and thus essential for the propagation of the classical complement pathway. Covalently binds to immunoglobulins and immune complexes and enhances the solubilization of immune aggregates and the clearance of IC through CR1 on erythrocytes. C4A isotype is responsible for effective binding to form amide bonds with immune aggregates or protein antigens, while C4B isotype catalyzes the transacylation of the thioester carbonyl group to form ester bonds with carbohydrate antigens.
**C4B**	**Complement C4-B**	[2]	8.69	62	67	0.9	89.86	-0.167	Complement protein	Immune response	Extracellular	Non-enzymatic component of the C3 and C5 convertases and thus essential for the propagation of the classical complement pathway. Covalently binds to immunoglobulins and immune complexes and enhances the solubilization of immune aggregates and the clearance of IC through CR1 on erythrocytes. C4A isotype is responsible for effective binding to form amide bonds with immune aggregates or protein antigens, while C4B isotype catalyzes the transacylation of the thioester carbonyl group to form ester bonds with carbohydrate antigens.
**C5**	**Complement C5**	[1]	6.11	192	175	1.1	91.04	-0.176	Complement protein	Immune response	Extracellular; Endosome	Activation of C5 by a C5 convertase initiates the spontaneous assembly of the late complement components, C5-C9, into the membrane attack complex. C5b has a transient binding site for C6. The C5b-C6 complex is the foundation upon which the lytic complex is assembled.
C1QBP	Complement component 1 Q subcomponent-binding protein, mitochondrial		4.10	41	13	3.2	76.07	-0.617	Complement receptor	Immune response	Mitochondrion; Endoplasmic reticulum; Golgi apparatus; Cytoplasm; Plasma membrane; Nucleus	Is believed to be a multifunctional and multicompartmental protein involved in inflammation and infection processes, ribosome biogenesis, regulation of apoptosis, transcriptional regulation and pre-mRNA splicing. At the cell surface is thought to act as an endothelial receptor for plasma proteins of the complement and kallikrein-kinin cascades. Putative receptor for C1q; specifically binds to the globular "heads" of C1q thus inhibiting C1; may perform the receptor function through a complex with C1qR/CD93.
C6	Complement component C6		6.17	119	110	1.1	60.65	-0.622	Complement protein	Immune response	Extracellular	Constituent of the membrane attack complex (MAC) that plays a key role in the innate and adaptive immune response by forming pores in the plasma membrane of target cells.
C7	Complement component C7		6.09	98	89	1.1	59.81	-0.535	Complement protein	Immune response	Extracellular	Constituent of the membrane attack complex (MAC) that plays a key role in the innate and adaptive immune response by forming pores in the plasma membrane of target cells. C7 serves as a membrane anchor.
**C8B**	**Complement component C8 beta chain**	[1]	7.85	64	67	1.0	64.95	-0.574	Complement protein	Immune response	Extracellular	Constituent of the membrane attack complex (MAC) that plays a key role in the innate and adaptive immune response by forming pores in the plasma membrane of target cells.
**C9**	**Complement component C9**	[1, 4-6]	5.42	82	69	1.2	69.22	-0.534	**Complement protein**	**Immune response**	**Extracellular**	**Constituent of the membrane attack complex (MAC) that plays a key role in the innate and adaptive immune response by forming pores in the plasma membrane of target cells. C9 is the pore-forming subunit of the MAC.**
**CFB**	**Complement factor B**	[1]	6.82	147	143	1.0	74.51	-0.464	Complement protein	Immune response	Extracellular	Factor B which is part of the alternate pathway of the complement system is cleaved by factor D into 2 fragments: Ba and Bb. Bb, a serine protease, then combines with complement factor 3b to generate the C3 or C5 convertase. It has also been implicated in proliferation and differentiation of preactivated B-lymphocytes, rapid spreading of peripheral blood monocytes, stimulation of lymphocyte blastogenesis and lysis of erythrocytes.
**CFH**	**Complement factor H**	[6, 8]	6.12	147	133	1.1	57.42	-0.643	Regulatory/other subunit	Immune response	Extracellular; Endoplasmic reticulum	Factor H functions as a cofactor in the inactivation of C3b by factor I and also increases the rate of dissociation of the C3bBb complex (C3 convertase) and the (C3b)NBB complex (C5 convertase) in the alternative complement pathway.
CFHR1	Complement factor H-related protein 1		7.10	35	35	1.0	53.08	-0.706	Complement protein	Immune response	Extracellular	Involved in complement regulation. The dimerized forms have avidity for tissue-bound complement fragments and efficiently compete with the physiological complement inhibitor CFH. Can associate with lipoproteins and may play a role in lipid metabolism.
CFHR5	Complement factor H-related protein 5		6.87	60	59	1.0	63.05	-0.531	Secreted polypeptide	Immune response; Complement activation	Extracellular	Involved in complement regulation. The dimerized forms have avidity for tissue-bound complement fragments and efficiently compete with the physiological complement inhibitor CFH.
GPS1	COP9 signalosome complex subunit 1		6.32	63	59	1.1	89.84	-0.322	Unclassified	Cell communication; Signal transduction	Nucleus; Cytoplasm; Golgi apparatus	Essential component of the COP9 signalosome complex (CSN), a complex involved in various cellular and developmental processes. The CSN complex is an essential regulator of the ubiquitin (Ubl) conjugation pathway by mediating the deneddylation of the cullin subunits of SCF-type E3 ligase complexes, leading to decrease the Ubl ligase activity of SCF-type complexes such as SCF, CSA or DDB2.
COPS4	COP9 signalosome complex subunit 4		5.57	54	43	1.3	96.72	-0.316	Unclassified	Protein metabolism	Cytoplasm; Nucleus; Cytoplasmic vesicle	Component of the COP9 signalosome complex (CSN), a complex involved in various cellular and developmental processes. The CSN complex is an essential regulator of the ubiquitin (Ubl) conjugation pathway by mediating the deneddylation of the cullin subunits of SCF-type E3 ligase complexes, leading to decrease the Ubl ligase activity of SCF-type complexes such as SCF, CSA or DDB2.
CORO1A	Coronin 1A		6.25	61	57	1.1	81.13	-0.324	Cytoskeletal associated protein	Cell growth and/or maintenance	Cytoplasm; Mitochondrion	May be a crucial component of the cytoskeleton of highly motile cells, functioning both in the invagination of large pieces of plasma membrane, as well as in forming protrusions of the plasma membrane involved in cell locomotion. In mycobacteria-infected cells, its retention on the phagosomal membrane prevents fusion between phagosomes and lysosomes.
CORO1B	Coronin1B		5.60	69	57	1.2	82.35	-0.315	Cytoskeletal associated protein	Cell motility	Cytoplasm; Cytoskeleton	Regulates leading edge dynamics and cell motility in fibroblasts. May be involved in cytokinesis and signal transduction By similarity.
SERPINA6	Corticosteroid-binding globulin		5.64	36	27	1.3	95.20	0.056	Transport/cargo protein	Transport	Extracellular	Major transport protein for glucocorticoids and progestins in the blood of almost all vertebrate species.
CKB	Creatine kinase B-type		5.35	57	42	1.4	82.34	-0.427	Enzyme: Phosphotransferase	Metabolism; Energy pathways	Cytoplasm	Reversibly catalyzes the transfer of phosphate between ATP and various phosphogens (e.g. creatine phosphate). Creatine kinase isoenzymes play a central role in energy transduction in tissues with large, fluctuating energy demands, such as skeletal muscle, heart, brain and spermatozoa.
**CKMT2**	**Creatine kinase S-type, mitochondrial**	[1]	7.28	55	55	1.0	83.08	-0.521	Enzyme: Phosphotransferase	Metabolism; Energy pathways	Mitochondrion; Extracellular	Reversibly catalyzes the transfer of phosphate between ATP and various phosphogens (e.g. creatine phosphate). Creatine kinase isoenzymes play a central role in energy transduction in tissues with large, fluctuating energy demands, such as skeletal muscle, heart, brain and spermatozoa.
**CUBN**	**Cubilin**	[2]	5.14	350	218	1.6	67.85	-0.300	**Transport/cargo protein**	**Transport**	**Plasma membrane**	**Cotransporter which plays a role in lipoprotein, vitamin and iron metabolism, by facilitating their uptake. Binds to ALB, MB, Kappa and lambda-light chains, TF, hemoglobin, GC, SCGB1A1, APOA1, high density lipoprotein, and the GIF-cobalamin complex. Serves as important transporter in several absorptive epithelia, including intestine, renal proximal tubules and embryonic yolk sac. Interaction with LRP2 mediates its trafficking throughout vesicles and facilitates the uptake of specific ligands like GC, hemoglobin, ALB, TF and SCGB1A1.**
**CAND1**	**Cullin-associated NEDD8-dissociated protein 1**	[3]	5.52	156	129	1.2	105.50	-0.021	Transcription regulatory protein	Regulation of nucleobase, nucleoside, nucleotide and nucleic acid metabolism	Nucleus; Mitochondrion	Key assembly factor of SCF (SKP1-CUL1-F-box protein) E3 ubiquitin ligase complexes that promotes the exchange of the substrate-recognition F-box subunit in SCF complexes, thereby playing a key role in the cellular repertoire of SCF complexes. Acts as a F-box protein exchange factor.
**CSTA**	**Cystatin-A**	[9-12]	5.39	15	13	1.2	80.31	-0.704	Protease inhibitor	Protein metabolism	Cytoplasm; Extracellular	This is an intracellular thiol proteinase inhibitor. Has an important role in desmosome-mediated cell-cell adhesion in the lower levels of the epidermis.
**CSTB**	**Cystatin-B**	[12]	6.96	11	11	1.0	66.63	-0.546	Protease inhibitor	Protein metabolism	Nucleus; Cytoplasm	This is an intracellular thiol proteinase inhibitor. Tightly binding reversible inhibitor of cathepsins L, H and B.
CST3	Cystatin-C		8.75	12	15	0.8	65.00	-0.510	Protease inhibitor	Protein metabolism	Extracellular	As an inhibitor of cysteine proteinases, this protein is thought to serve an important physiological role as a local regulator of this enzyme activity.
CYB5A	Cytochrome b5		4.86	24	12	2.0	78.50	-0.586	Enzyme: Oxidoreductase	Metabolism; Energy pathways	Mitochondrion	Cytochrome b5 is a membrane bound hemoprotein which function as an electron carrier for several membrane bound oxygenases.
UQCRC2	Cytochrome b-c1 complex subunit 2, mitochondrial		7.74	37	38	1.0	88.52	-0.074	Enzyme: Reductase	Metabolism; Energy pathways	Mitochondrion	This is a component of the ubiquinol-cytochrome c reductase complex (complex III or cytochrome b-c1 complex), which is part of the mitochondrial respiratory chain. The core protein 2 is required for the assembly of the complex.
**CYP4X1**	**Cytochrome P450 4X1**	[3]	8.74	52	60	0.9	88.55	-0.210	**Enzyme: Oxygenase**	**Metabolism; Energy pathways**	**Endoplasmic reticulum**	**The cytochrome P450 proteins are monooxygenases which catalyze many reactions involved in drug metabolism and synthesis of cholesterol, steroids and other lipids. RH + reduced flavoprotein + O2 = ROH + oxidized flavoprotein + H2O.**
DYNC1H1	Cytoplasmic dynein 1 heavy chain 1		6.01	639	582	1.1	92.75	-0.341	ATPase	Metabolism; Energy pathways	Cytoplasm; Golgi apparatus; Endosome; Lysosome; Mitochondrion; Plasma membrane; Nucleus; Nucleolus; Microtubule	Cytoplasmic dynein 1 acts as a motor for the intracellular retrograde motility of vesicles and organelles along microtubules. Dynein has ATPase activity; the force-producing power stroke is thought to occur on release of ADP.
**DYNC1LI2**	**Cytoplasmic dynein 1 light intermediate chain 2**	[1]	5.97	69	61	1.1	78.46	-0.496	Unclassified	Unknown	Cytoplasm; Microtubule	Acts as one of several non-catalytic accessory components of the cytoplasmic dynein 1 complex that are thought to be involved in linking dynein to cargos and to adapter proteins that regulate dynein function. Cytoplasmic dynein 1 acts as a motor for the intracellular retrograde motility of vesicles and organelles along microtubules. May play a role in binding dynein to membranous organelles or chromosomes.
CYFIP1	Cytoplasmic FMR1-interacting protein 1		6.46	155	145	1.1	91.33	-0.264	Cytoskeletal associated protein	Lamellipodium assembly	Cytoplasm; Nucleus	Component of the CYFIP1-EIF4E-FMR1 complex which binds to the mRNA cap and mediates translational repression. In the CYFIP1-EIF4E-FMR1 complex this subunit is an adapter between EIF4E and FMR1. Promotes the translation repression activity of FMR1 in brain probably by mediating its association with EIF4E and mRNA By similarity. Regulates formation of membrane ruffles and lamellipodia.
LAP3	Cytosol aminopeptidase		8.03	58	60	1.0	85.97	-0.172	**Aminopeptidase**	**Protein metabolism**	**Cytoplasm**	**Presumably involved in the processing and regular turnover of intracellular proteins. Catalyzes the removal of unsubstituted N-terminal amino acids from various peptides.**
ALDH1L1	Cytosolic 10-formyltetrahydrofolate dehydrogenase		5.63	117	98	1.2	89.71	-0.113	Enzyme: Dehydrogenase	Metabolism; Energy pathways	Cytoplasm	Formyltetrahydrofolate dehydrogenase activity; 10-formyltetrahydrofolate + NADP+ + H2O = tetrahydrofolate + CO2 + NADPH.
CNDP2	Cytosolic non-specific dipeptidase		5.66	67	56	1.2	87.59	-0.281	**Metallo protease**	**Protein metabolism**	**Cytoplasm**	**Hydrolyzes a variety of dipeptides including L-carnosine but has a strong preference for Cys-Gly. Isoform 2 may be play a role as tumor suppressor in hepatocellular carcinoma (HCC) cells.**
NT5C2	Cytosolic purine 5'-nucleotidase		5.75	81	67	1.2	77.84	-0.428	Enzyme: Hydrolase	Metabolism; Energy pathways	Cytoplasm	May have a critical role in the maintenance of a constant composition of intracellular purine/pyrimidine nucleotides in cooperation with other nucleotidases. Preferentially hydrolyzes inosine 5'-monophosphate (IMP) and other purine nucleotides.
PHGDH	D-3-phosphoglycerate dehydrogenase		6.31	51	48	1.1	99.38	0.090	Enzyme: Dehydrogenase	Metabolism; Energy pathways	Extracellular; Cytosol	3-phospho-D-glycerate + NAD+ = 3-phosphonooxypyruvate + NADH; 2-hydroxyglutarate + NAD+ = 2-oxoglutarate + NADH.
**DACT3**	**Dapper homolog 3**	[1]	10.38	64	89	0.7	54.39	-0.711	Protein kinase binding	Negative regulation of Wnt signaling pathway	Cytoplasm; Nucleus	May be involved in regulation of intracellular signaling pathways during development. Specifically thought to play a role in canonical and/or non-canonical Wnt signaling pathways through interaction with DSH (Dishevelled) family proteins.

**DHRS2**	**Dehydrogenase/reductase SDR family member 2, mitochondrial**	**[1]**	8.90	22	27	0.8	107.32	0.165	Enzyme: Oxidoreductase	Metabolism; Energy pathways	Nucleus; Cytoplasm	Displays NADPH-dependent dicarbonyl reductase activity in vitro with 3,4-Hexanedione, 2,3-Heptanedione and 1-Phenyl-1,2-propanedione as substrates. No reductase activity is displayed in vitro with steroids, retinoids and sugars as substrates.
**DENND5A**	**DENN domain-containing protein 5A**	**[3]**	6.21	163	147	1.1	87.03	-0.352	Unclassified	Unknown	Cytoplasm	Guanine nucleotide exchange factor (GEF) which may activate RAB6A and RAB39A and/or RAB39B. Promotes the exchange of GDP to GTP, converting inactive GDP-bound Rab proteins into their active GTP-bound form.
DSPP	Dentin sialophosphoprotein		3.57	323	56	5.8	15.92	-1.663	Extracellular matrix protein	Cell growth and/or maintenance	Extracellular	DSP may be an important factor in dentinogenesis. DPP may bind high amount of calcium and facilitate initial mineralization of dentin matrix collagen as well as regulate the size and shape of the crystals.
DNASE2	Deoxyribonuclease-2-alpha		8.35	24	27	0.9	66.75	-0.389	Deoxyribonuclease	Regulation of nucleobase, nucleoside, nucleotide and nucleic acid metabolism	Lysosome; Nucleus	Hydrolyzes DNA under acidic conditions with a preference for double-stranded DNA. Plays a major role in the degradation of nuclear DNA in cellular apoptosis during development. Necessary for proper fetal development and for definitive erythropoiesis in fetal liver, where it degrades nuclear DNA expelled from erythroid precursor cells.
DPT	Dermatopontin		4.78	24	16	1.5	39.95	-0.813	Extracellular matrix protein	Cell growth and/or maintenance	Extracellular	Seems to mediate adhesion by cell surface integrin binding. May serve as a communication link between the dermal fibroblast cell surface and its extracellular matrix environment. Enhances TGFB1 activity. Inhibits cell proliferation. Accelerates collagen fibril formation, and stabilizes collagen fibrils against low-temperature dissociation By similarity.
**DCD**	**Dermcidin**	**[4, 9-11]**	5.64	15	13	1.2	75.16	-0.667	Secreted polypeptide	Immune response	Extracellular; Golgi apparatus	DCD-1 displays antimicrobial activity thereby limiting skin infection by potential pathogens in the first few hours after bacterial colonization. Highly effective against E.coli, E.faecalis, S.aureus and C.albicans. Optimal pH and salt concentration resemble the conditions in sweat. Also exhibits proteolytic activity.
**DSC1**	**Desmocollin-1a**	**[10]**	4.89	109	72	1.5	76.38	-0.485	Adhesion molecule	Cell growth and/or maintenance	Plasma membrane; Cytoplasmic vesicle; Extracellular	Component of intercellular desmosome junctions. Involved in the interaction of plaque proteins and intermediate filaments mediating cell-cell adhesion. May contribute to epidermal cell positioning (stratification) by mediating differential adhesiveness between cells that express different isoforms. Linked to the keratinization of epithelial
DSC2	Desmocollin-2		4.80	103	67	1.5	77.06	-0.400	Adhesion molecule	Cell growth and/or maintenance	Plasma membrane	Component of intercellular desmosome junctions. Involved in the interaction of plaque proteins and intermediate filaments mediating cell-cell adhesion. May contribute to epidermal cell positioning (stratification) by mediating differential adhesiveness between cells that express different isoforms.
**DSG1**	**Desmoglein-1**	**[10-11]**	4.77	121	74	1.6	79.42	-0.285	Cell junction protein	Cell growth and/or maintenance	Plasma membrane; Extracellular	Component of intercellular desmosome junctions. Involved in the interaction of plaque proteins and intermediate filaments mediating cell-cell adhesion.
DSTN	Destrin		8.12	25	27	0.9	84.45	-0.167	Cytoskeletal associated protein	Cell growth and/or maintenance	Nucleus	Actin-depolymerizing protein. Severs actin filaments (F-actin) and binds to actin monomers (G-actin). Acts in a pH-independent manner.
DPYS	Dihydropyrimidinase		6.81	62	60	1.0	81.39	-0.198	Enzyme: Hydrolase	Regulation of nucleobase, nucleoside, nucleotide and nucleic acid metabolism	Cytoplasm	Catalyzes the second step of the reductive pyrimidine degradation, the reversible hydrolytic ring opening of dihydropyrimidines. Can catalyze the ring opening of 5,6-dihydrouracil to N-carbamyl-alanine and of 5,6-dihydrothymine to N-carbamyl-amino isobutyrate.
DPYD	Dihydropyrimidine dehydrogenase [NADP(+)]		6.84	109	108	1.0	85.43	-0.076	Enzyme: Dehydrogenase	Metabolism; Energy pathways	Cytoplasm	Involved in pyrimidine base degradation. Catalyzes the reduction of uracil and thymine. Also involved the degradation of the chemotherapeutic drug 5-fluorouracil.
DPEP1	Dipeptidase 1		5.50	46	36	1.3	86.64	-0.284	Protease	Protein metabolism	Endoplasmic reticulum; Zymogen granule	Hydrolyzes a wide range of dipeptides. Implicated in the renal metabolism of glutathione and its conjugates. Converts leukotriene D4 to leukotriene E4; it may play an important role in the regulation of leukotriene activity.
CTSC	Dipeptidyl peptidase 1		6.56	44	41	1.1	69.73	-0.348	Cysteine protease	Protein metabolism	Lysosome; Mitochondrion	Thiol protease. Has dipeptidylpeptidase activity. Active against a broad range of dipeptide substrates composed of both polar and hydrophobic amino acids. Proline cannot occupy the P1 position and arginine cannot occupy the P2 position of the substrate. Can act as both an exopeptidase and endopeptidase. Activates serine proteases such as elastase, cathepsin G and granzymes A and B. Can also activate neuraminidase and factor XIII.
**DAAM1**	**Disheveled-associated activator of morphogenesis 1**	**[3]**	6.81	164	161	1.0	80.44	-0.630	Unclassified	Cell communication; Signal transduction	Cytoplasm; Nucleus	Binds to disheveled (Dvl) and Rho, and mediates Wnt-induced Dvl-Rho complex formation. May play a role as a scaffolding protein to recruit Rho-GDP and Rho-GEF, thereby enhancing Rho-GTP formation. Can direct nucleation and elongation of new actin filaments.
**POLK**	**DNA polymerase kappa**	**[3]**	8.42	117	125	0.9	75.18	-0.609	DNA binding protein	Regulation of nucleobase, nucleoside, nucleotide and nucleic acid metabolism	Nucleus	DNA polymerase specifically involved in DNA repair. Plays an important role in translesion synthesis, where the normal high-fidelity DNA polymerases cannot proceed and DNA synthesis stalls.
**RAD50**	**DNA repair protein RAD50**	**[3]**	6.47	239	231	1.0	84.18	-0.915	DNA binding protein	Regulation of nucleobase, nucleoside, nucleotide and nucleic acid metabolism	Nucleus; Nucleolus; Cytoplasm	Component of the MRN complex, which plays a central role in double-strand break (DSB) repair, DNA recombination, maintenance of telomere integrity and meiosis. The complex may also be required for DNA damage signaling via activation of the ATM kinase. In telomeres the MRN complex may modulate t-loop formation.
DNAJA1	DnaJ homolog subfamily A member 1		6.65	63	61	1.0	72.44	-0.722	Heat shock protein	Protein metabolism	Nucleus; Nucleolus; Golgi apparatus; Acrosome	Co-chaperone for HSPA8/Hsc70 (PubMed:10816573). Stimulates ATP hydrolysis, but not the folding of unfolded proteins mediated by HSPA1A (in vitro) (PubMed:24318877). Plays a role in protein transport into mitochondria via its role as co-chaperone. Functions as co-chaperone for HSPA1B and negatively regulates the translocation of BAX from the cytosol to mitochondria in response to cellular stress, thereby protecting cells against apoptosis.
DNAJA2	DnaJ homolog subfamily A member 2		6.01	62	56	1.1	68.34	-0.691	Chaperone	Protein metabolism	Cytoplasm; Nucleus; Mitochondrion; Microsome	Co-chaperone of Hsc70.
DDOST	Dolichyl-diphosphooligosaccharide-protein glycosyltransferase 48 kDa subunit		5.41	48	37	1.3	90.36	-0.096	Enzyme: Galactosyltransferase	Metabolism; Energy pathways	Endoplasmic reticulum; Mitochondrion	Essential subunit of the N-oligosaccharyl transferase (OST) complex which catalyzes the transfer of a high mannose oligosaccharide from a lipid-linked oligosaccharide donor to an asparagine residue within an Asn-X-Ser/Thr consensus motif in nascent polypeptide chains.
RPN1	Dolichyl-diphosphooligosaccharide--protein glycosyltransferase subunit 1		6.03	79	69	1.1	97.09	-0.259	Ubiquitin proteasome system protein	Protein metabolism	Endoplasmic reticulum; Nucleus; Cytoplasm; Mitochondrion; Cytosol	Essential subunit of the N-oligosaccharyl transferase (OST) complex which catalyzes the transfer of a high mannose oligosaccharide from a lipid-linked oligosaccharide donor to an asparagine residue within an Asn-X-Ser/Thr consensus motif in nascent polypeptide chains.
RPN2	Dolichyl-diphosphooligosaccharide--protein glycosyltransferase subunit 2		5.44	65	48	1.4	99.61	0.039	Enzyme: Glycosyltransferase	Protein metabolism	Endoplasmic reticulum; Mitochondrion	Essential subunit of the N-oligosaccharyl transferase (OST) complex which catalyzes the transfer of a high mannose oligosaccharide from a lipid-linked oligosaccharide donor to an asparagine residue within an Asn-X-Ser/Thr consensus motif in nascent polypeptide chains.
DBNL	Drebrin-like protein		5.01	72	50	1.4	61.30	-0.844	Adapter molecule	Signal transduction	Actin cytoskeleton; Dendrite; Cytoplasm	Adapter protein that binds F-actin and DNM1, and thereby plays a role in receptor-mediated endocytosis. Plays a role in the reorganization of the actin cytoskeleton, formation of cell projections, such as neurites, in neuron morphogenesis and synapse formation via its interaction with WASL and COBL. Does not bind G-actin and promote actin polymerization by itself. Required for the formation of organized podosome rosettes By similarity.
MAP2K3	Dual specificity mitogen-activated protein kinase kinase 3		7.05	46	46	1.0	80.61	-0.345	Serine/threonine kinase	Signal transduction	Nucleus; Cytoplasm	Dual specificity kinase. Is activated by cytokines and environmental stress in vivo. Catalyzes the concomitant phosphorylation of a threonine and a tyrosine residue in the MAP kinase p38.
DNM1L	Dynamin-1-like protein		6.37	97	92	1.1	101.45	-0.259	GTPase	Mitochondrion organization and biogenesis	Endoplasmic reticulum; Golgi apparatus; Cytoplasm	Functions in mitochondrial and peroxisomal division. Mediates membrane fission through oligomerization into membrane-associated tubular structures that wrap around the scission site to constrict and sever the mitochondrial membrane through a GTP hydrolysis-dependent mechanism. Through its function in mitochondrial division, ensures the survival of at least some types of postmitotic neurons, including Purkinje cells, by suppressing oxidative damage.
DNM2	Dynamin-2		7.04	114	113	1.0	90.10	-0.433	GTPase	Cell communication; Signal transduction	Cytoskeleton; Perinuclear region; Cell surface; Mitochondrion	Microtubule-associated force-producing protein involved in producing microtubule bundles and able to bind and hydrolyze GTP. Most probably involved in vesicular trafficking processes, in particular endocytosis. Involved in cytokinesis.
**DTNB**	**Dystrobrevin beta**	**[3]**	8.11	73	76	1.0	74.69	-0.556	Cytoskeletal protein	Cell growth and/or maintenance	Cytoplasm	A component of the dystrophin-associated protein complex (DPC). The DPC consists of dystrophin and several integral and peripheral membrane proteins, including dystroglycans, sarcoglycans, syntrophins and dystrobrevin alpha and beta. The DPC localizes to the sarcolemma and its disruption is associated with various forms of muscular dystrophy.
TRIM25	E3 ubiquitin/ISG15 ligase TRIM25		8.44	70	79	0.9	76.49	-0.437	Transcription factor	Regulation of nucleobase, nucleoside, nucleotide and nucleic acid metabolism	Nucleus; Cytoplasm	Functions as a ubiquitin E3 ligase and as an ISG15 E3 ligase. Involved in innate immune defense against viruses by mediating ubiquitination of DDX58. Mediates 'Lys-63'-linked polyubiquitination of the DDX58 N-terminal CARD-like region which is crucial for triggering the cytosolic signal transduction that leads to the production of interferons in response to viral infection.
**MIB2**	**E3 ubiquitin-protein ligase MIB2**	**[1]**	8.81	109	125	0.9	83.68	-0.351	Cytoskeletal associated protein	Cell growth and/or maintenance	Cytoplasm; Nucleus	E3 ubiquitin-protein ligase that mediates ubiquitination of Delta receptors, which act as ligands of Notch proteins. Positively regulates the Delta-mediated Notch signaling by ubiquitinating the intracellular domain of Delta, leading to endocytosis of Delta receptors (By similarity).
UBR4	E3 ubiquitin-protein ligase UBR4		5.70	604	502	1.2	90.75	-0.200	Ligase	Host-virus interaction; Ubl conjugation pathway	Cytoplasm; Nucleus	E3 ubiquitin-protein ligase which is a component of the N-end rule pathway. Recognizes and binds to proteins bearing specific N-terminal residues that are destabilizing according to the N-end rule, leading to their ubiquitination and subsequent degradation. Together with clathrin, forms meshwork structures involved in membrane morphogenesis and cytoskeletal organization. Regulates integrin-mediated signaling.
EEA1	Early endosome antigen 1		5.55	262	224	1.2	83.95	-1.049	Membrane transport protein	Transport	Endosome; Cytoplasm; Nucleus; Cytosol; Centrosome	Binds phospholipid vesicles containing phosphatidylinositol 3-phosphate and participates in endosomal trafficking.
EML4	Echinoderm microtubule-associated protein-like 4		5.96	129	109	1.2	75.31	-0.538	Structural protein	Cell growth and/or maintenance	Cytoplasm; Microtubule	May modify the assembly dynamics of microtubules, such that microtubules are slightly longer, but more dynamic.
**EFEMP1**	**EGF-containing fibulin-like extracellular matrix protein 1**	**[2]**	4.85	53	35	1.5	62.46	-0.416	Extracellular matrix protein	Cell growth and/or maintenance	Extracellular	Binds EGFR, the EGF receptor, inducing EGFR autophosphorylation and the activation of downstream signaling pathways. May play a role in cell adhesion and migration. May function as a negative regulator of chondrocyte differentiation.
EHD4	EH domain-containing protein 4		6.32	73	70	1.0	85.99	-0.379	Calcium binding protein	Cell communication; Signal transduction	Extracellular; Cytoplasm	Plays a role in early endosomal transport.
**ELMSAN1**	**ELM2 and SANT domain-containing protein 1 (C14orf43)**	**[1]**	9.26	111	130	0.9	66.22	-0.792	DNA binding protein	Regulation of nucleobase, nucleoside, nucleotide and nucleic acid metabolism	Nucleus	chromatin binding
**EEF1A1**	**Elongation factor 1-alpha 1**	**[1, 3]**	9.10	53	64	0.8	83.33	-0.257	Transcription regulatory protein	Regulation of cell cycle	Cytoplasm; Nucleus; Nucleolus; Mitochondrion; Ribosome	This protein promotes the GTP-dependent binding of aminoacyl-tRNA to the A-site of ribosomes during protein biosynthesis.
**EEF1B2**	**Elongation factor 1-beta**	**[8]**	4.50	45	27	1.7	81.43	-0.502	Translation regulatory protein	Protein metabolism	Cytoplasm	EF-1-beta and EF-1-delta stimulate the exchange of GDP bound to EF-1-alpha to GTP.
EEF1D	Elongation factor 1-delta		4.90	51	36	1.4	83.64	-0.594	Guanine nucleotide exchange factor	Cell communication; Signal transduction	Endoplasmic reticulum; Mitochondrion; Nucleus; Cytoplasm	EF-1-beta and EF-1-delta stimulate the exchange of GDP bound to EF-1-alpha to GTP, regenerating EF-1-alpha for another round of transfer of aminoacyl-tRNAs to the ribosome. Regulates induction of heat-shock-responsive genes through association with heat shock transcription factors and direct DNA-binding at heat shock promoter elements (HSE).
EEF1G	Elongation factor 1-gamma		6.27	57	54	1.1	67.18	-0.486	Translation regulatory protein	Protein metabolism	Nucleolus; Plasma membrane; Cytoplasm; Mitochondrion	Probably plays a role in anchoring the complex to other cellular components.
EEF2	Elongation factor 2		6.42	114	109	1.0	88.38	-0.212	Translation regulatory protein	Protein metabolism; Translation	Cytoplasm; Nucleolus; Cytosol; Mitochondrion; Ribosome; Nucleus	Catalyzes the GTP-dependent ribosomal translocation step during translation elongation. During this step, the ribosome changes from the pre-translocational (PRE) to the post-translocational (POST) state as the newly formed A-site-bound peptidyl-tRNA and P-site-bound deacylated tRNA move to the P and E sites, respectively. Catalyzes the coordinated movement of the two tRNA molecules, the mRNA and conformational changes in the ribosome.
SH3GLB2	Endophilin-B2		5.72	55	49	1.1	83.09	-0.391	Unclassified	Unknown	Cytoplasm	Protein binding.
**HSP90B1**	**Endoplasmin**	**[3]**	4.73	171	114	1.5	75.91	-0.774	Heat shock protein	Protein metabolism	Endoplasmic reticulum; Mitochondrion; Cytosol	Molecular chaperone that functions in the processing and transport of secreted proteins. When associated with CNPY3, required for proper folding of Toll-like receptors By similarity. Functions in endoplasmic reticulum associated degradation (ERAD). Has ATPase activity.
COL18A1	Endostatin (Collagen alpha-1(XVIII) chain)		9.30	15	20	0.8	79.51	-0.217	Extracellular matrix protein	Cell growth and/or maintenance	Extracellular	COLA18A probably plays a major role in determining the retinal structure as well as in the closure of the neural tube. Endostatin potently inhibits endothelial cell proliferation and angiogenesis. May inhibit angiogenesis by binding to the heparan sulfate proteoglycans involved in growth factor
PROCR	Endothelial protein C receptor		6.69	18	17	1.1	89.05	-0.124	Immunoglobulin	Immune response	Plasma membrane; Centrosome	Binds activated protein C. Enhances protein C activation by the thrombin-thrombomodulin complex; plays a role in the protein C pathway controlling blood coagulation.
ECHS1	Enoyl-CoA hydratase, mitochondrial		5.88	33	31	1.1	86.20	-0.101	Enzyme: Hydratase	Metabolism; Energy pathways	Mitochondrion	Straight-chain enoyl-CoA thioesters from C4 up to at least C16 are processed, although with decreasing catalytic rate.
**RNASE3**	**Eosinophil cationic protein**	**[1, 4-8, 12]**	10.47	6	19	0.3	69.62	-0.598	Ribonuclease	Regulation of nucleobase, nucleoside, nucleotide and nucleic acid metabolism	Cytoplasm; Extracellular	Cytotoxin and helminthotoxin with low-efficiency ribonuclease activity. Possesses a wide variety of biological activities. Exhibits antibacterial activity, including cytoplasmic membrane depolarization of preferentially Gram-negative, but also Gram-positive strains. Promotes E.coli outer membrane detachment, alteration of the overall cell shape and partial loss of cell content.
**EPX**	**Eosinophil peroxidase**	**[1, 4, 6]**	10.31	60	103	0.6	84.05	-0.372	Enzyme: Peroxidase	Immune response	Cytoplasm	Mediates tyrosine nitration of secondary granule proteins in mature resting eosinophils. Shows significant inhibitory activity towards Mycobacterium tuberculosis H37Rv by inducing bacterial fragmentation and lysis.
NPC2	Epididymal secretory protein E1		6.95	12	12	1.0	86.14	-0.217	Transport/cargo protein	Metabolism; Energy pathways	Cytoplasm; Lysosome	Intracellular cholesterol transporter which acts in concert with NPC1 and plays an important role in the egress of cholesterol from the endosomal/lysosomal compartment. NPC2 binds unesterified cholesterol that has been released from LDLs in the lumen of the late endosomes/lysosomes and transfers it to the cholesterol-binding pocket of the N-terminal domain of NPC1. The secreted form of NCP2 regulates biliary cholesterol secretion via stimulation of ABCG5/ABCG8-mediated cholesterol transport.

EPPK1	Epiplakin		5.44	676	563	1.2	92.94	-0.266	Cytoskeletal associated protein	Cell growth and/or maintenance	Cytoplasm; Nucleus	Belongs to the plakin family of proteins, which play a role in the organization. of cytoskeletal architecture. This family member is composed of several highly homologous plakin repeats. It may function to maintain the integrity of keratin intermediate filament networks in epithelial cells.
ERC2	ERC protein 2		6.51	175	165	1.1	80.28	-1.046	Cytoskeletal associated protein	Cell growth and/or maintenance	Cytoplasm	Thought to be involved in the organization of the cytomatrix at the nerve terminals active zone (CAZ) which regulates neurotransmitter release. Seems to act together with BSN. May recruit liprin-alpha proteins to the CAZ.
ERLIN1	Erlin-1		7.67	44	45	1.0	93.35	-0.268	Unclassified	ER-associated ubiquitin-dependent protein catabolic process	Plasma membrane; Cytoplasm; Endoplasmic reticulum	Component of the ERLIN1/ERLIN2 complex which mediates the endoplasmic reticulum-associated degradation (ERAD) of inositol 1,4,5-trisphosphate receptors (IP3Rs).
**STOM**	**Erythrocyte band 7 integral membrane protein (stomatin)**	[1, 6]	7.71	32	33	1.0	102.36	0.043	Integral membrane protein	Cell communication; Signal transduction	Cytoplasm; Plasma membrane; Cytoplasmic vesicle; Nucleus	Regulates ion channel activity and transmembrane ion transport. Regulates ASIC2 and ASIC3 channel activity.
**ELF2**	**ETS-related transcription factor Elf-2**	[1]	6.15	70	65	1.1	80.83	-0.345	**Transcription factor**	**Regulation of nucleobase, nucleoside, nucleotide and nucleic acid metabolism**	**Nucleus**	**Isoform 1 transcriptionally activates the LYN and BLK promoters and acts synergistically with RUNX1 to transactivate the BLK promoter. Isoform 2 may function in repression of RUNX1-mediated transactivation.**
EIF4A1	Eukaryotic initiation factor 4A-I		5.32	58	49	1.2	93.63	-0.267	Translation regulatory protein	Protein metabolism	Nucleus; Cytoplasm; Ribosome	ATP-dependent RNA helicase which is a subunit of the eIF4F complex involved in cap recognition and is required for mRNA binding to ribosome. In the current model of translation initiation, eIF4A unwinds RNA secondary structures in the 5'-UTR of mRNAs which is necessary to allow efficient binding of the small ribosomal subunit, and subsequent scanning for the initiator codon.
EIF4A2	Eukaryotic initiation factor 4A-II		5.33	59	49	1.2	93.88	-0.258	Translation regulatory protein	Protein metabolism	Cytoplasm	ATP-dependent RNA helicase which is a subunit of the eIF4F complex involved in cap recognition and is required for mRNA binding to ribosome. In the current model of translation initiation, eIF4A unwinds RNA secondary structures in the 5'-UTR of mRNAs which is necessary to allow efficient binding of the small ribosomal subunit, and subsequent scanning for the initiator codon.
GSPT1	Eukaryotic peptide chain release factor GTP-binding subunit ERF3A		5.44	81	64	1.3	82.20	-0.431	Cell cycle control protein	Cell communication; Signal transduction	Cytoplasm; Endoplasmic reticulum	Involved in translation termination in response to the termination codons UAA, UAG and UGA. Stimulates the activity of ERF1. Involved in regulation of mammalian cell growth.
GSPT2	Eukaryotic peptide chain release factor GTP-binding subunit ERF3B		5.30	95	74	1.3	76.34	-0.419	Cell cycle control protein	Cell communication; Signal transduction	Cytoplasm	Involved in translation termination in response to the termination codons UAA, UAG and UGA. Stimulates the activity of ERF1. Involved in regulation of mammalian cell growth.
EEF1E1	Eukaryotic translation elongation factor 1 epsilon-1		8.58	16	18	0.9	101.45	-0.308	Translation regulatory protein	Protein metabolism	Cytoplasm	Positive modulator of ATM response to DNA damage.
**EIF2S3**	**Eukaryotic translation initiation factor 2 subunit 3**	[3]	8.66	52	58	0.9	104.27	-0.018	Translation regulatory protein	Protein metabolism	Cytoplasm; Nucleolus	eIF-2 functions in the early steps of protein synthesis by forming a ternary complex with GTP and initiator tRNA.
EIF3A	Eukaryotic translation initiation factor 3 subunit A		6.39	320	311	1.0	61.46	-1.491	Translation regulatory protein	Protein metabolism	Cytoplasm	Component of the eukaryotic translation initiation factor 3 (eIF-3) complex, which is required for several steps in the initiation of protein synthesis. The eIF-3 complex associates with the 40S ribosome and facilitates the recruitment of eIF-1, eIF-1A, eIF-2:GTP:methionyl-tRNAi and eIF-5 to form the 43S preinitiation complex (43S PIC). The eIF-3 complex is also required for disassembly and recycling of post-termination ribosomal complexes and subsequently prevents premature joining of the 40S and 60S ribosomal subunits prior to initiation.
EIF3B	Eukaryotic translation initiation factor 3 subunit B		4.89	140	97	1.4	70.58	-0.632	Translation regulatory protein	Protein metabolism	Ribosome; Cytoplasm; Nucleus	Component of the eukaryotic translation initiation factor 3 (eIF-3) complex, which is required for several steps in the initiation of protein synthesis. The eIF-3 complex associates with the 40S ribosome and facilitates the recruitment of eIF-1, eIF-1A, eIF-2:GTP:methionyl-tRNAi and eIF-5 to form the 43S preinitiation complex (43S PIC). The eIF-3 complex is also required for disassembly and recycling of post-termination ribosomal complexes and subsequently prevents premature joining of the 40S and 60S ribosomal subunits prior to initiation.
EIF3C	Eukaryotic translation initiation factor 3 subunit C		5.48	165	135	1.2	76.29	-0.793	Translation regulatory protein	Protein metabolism	Ribosome; Cytoplasm	Component of the eukaryotic translation initiation factor 3 (eIF-3) complex, which is required for several steps in the initiation of protein synthesis. The eIF-3 complex associates with the 40S ribosome and facilitates the recruitment of eIF-1, eIF-1A, eIF-2:GTP:methionyl-tRNAi and eIF-5 to form the 43S preinitiation complex (43S PIC). The eIF-3 complex is also required for disassembly and recycling of post-termination ribosomal complexes and subsequently prevents premature joining of the 40S and 60S ribosomal subunits prior to initiation.
EIF3D	Eukaryotic translation initiation factor 3 subunit D		5.79	88	81	1.1	64.22	-0.842	Translation regulatory protein	Protein metabolism	Cytoplasm	Component of the eukaryotic translation initiation factor 3 (eIF-3) complex, which is required for several steps in the initiation of protein synthesis. The eIF-3 complex associates with the 40S ribosome and facilitates the recruitment of eIF-1, eIF-1A, eIF-2:GTP:methionyl-tRNAi and eIF-5 to form the 43S preinitiation complex (43S PIC). The eIF-3 complex is also required for disassembly and recycling of post-termination ribosomal complexes and subsequently prevents premature joining of the 40S and 60S ribosomal subunits prior to initiation.
EIF3E	Eukaryotic translation initiation factor 3 subunit E		5.72	60	52	1.2	90.05	-0.332	Translation regulatory protein	Protein metabolism	Cytoplasm; Nucleus	Component of the eukaryotic translation initiation factor 3 (eIF-3) complex, which is required for several steps in the initiation of protein synthesis. The eIF-3 complex associates with the 40S ribosome and facilitates the recruitment of eIF-1, eIF-1A, eIF-2:GTP:methionyl-tRNAi and eIF-5 to form the 43S preinitiation complex (43S PIC). The eIF-3 complex is also required for disassembly and recycling of post-termination ribosomal complexes and subsequently prevents premature joining of the 40S and 60S ribosomal subunits prior to initiation.
EIF3F	Eukaryotic translation initiation factor 3 subunit F		5.24	32	22	1.5	91.29	0.038	Translation regulatory protein	Protein metabolism	Cytoplasm; Nucleus	Component of the eukaryotic translation initiation factor 3 (eIF-3) complex, which is required for several steps in the initiation of protein synthesis. The eIF-3 complex associates with the 40S ribosome and facilitates the recruitment of eIF-1, eIF-1A, eIF-2:GTP:methionyl-tRNAi and eIF-5 to form the 43S preinitiation complex (43S PIC). The eIF-3 complex is also required for disassembly and recycling of post-termination ribosomal complexes and subsequently prevents premature joining of the 40S and 60S ribosomal subunits prior to initiation.
EIF3I	Eukaryotic translation initiation factor 3 subunit I		5.38	43	31	1.4	67.48	-0.416	Translation regulatory protein	Protein metabolism	Ribosome	Component of the eukaryotic translation initiation factor 3 (eIF-3) complex, which is required for several steps in the initiation of protein synthesis. The eIF-3 complex associates with the 40S ribosome and facilitates the recruitment of eIF-1, eIF-1A, eIF-2:GTP:methionyl-tRNAi and eIF-5 to form the 43S preinitiation complex (43S PIC). The eIF-3 complex is also required for disassembly and recycling of post-termination ribosomal complexes and subsequently prevents premature joining of the 40S and 60S ribosomal subunits prior to initiation.
EIF3L	Eukaryotic translation initiation factor 3 subunit L		5.93	72	63	1.1	82.42	-0.477	Translation regulatory protein	Protein metabolism	Cytoplasm	Component of the eukaryotic translation initiation factor 3 (eIF-3) complex, which is required for several steps in the initiation of protein synthesis. The eIF-3 complex associates with the 40S ribosome and facilitates the recruitment of eIF-1, eIF-1A, eIF-2:GTP:methionyl-tRNAi and eIF-5 to form the 43S preinitiation complex (43S PIC). The eIF-3 complex is also required for disassembly and recycling of post-termination ribosomal complexes and subsequently prevents premature joining of the 40S and 60S ribosomal subunits prior to initiation.
EIF3M	Eukaryotic translation initiation factor 3 subunit M		5.41	55	44	1.3	100.88	-0.190	Translation regulatory protein	Protein metabolism	Cytoplasm; Nucleus	Component of the eukaryotic translation initiation factor 3 (eIF-3) complex, which is required for several steps in the initiation of protein synthesis. The eIF-3 complex associates with the 40S ribosome and facilitates the recruitment of eIF-1, eIF-1A, eIF-2:GTP:methionyl-tRNAi and eIF-5 to form the 43S preinitiation complex (43S PIC). The eIF-3 complex is also required for disassembly and recycling of post-termination ribosomal complexes and subsequently prevents premature joining of the 40S and 60S ribosomal subunits prior to initiation.
**EIF5**	**Eukaryotic translation initiation factor 5**	[3]	5.41	83	68	1.2	74.83	-0.802	Translation regulatory protein; GTPase activating protein	Protein metabolism	Cytoplasm	Catalyzes the hydrolysis of GTP bound to the 40S ribosomal initiation complex (40S.mRNA.Met-tRNA[F].eIF-2.GTP) with the subsequent joining of a 60S ribosomal subunit resulting in the release of eIF-2 and the guanine nucleotide. The subsequent joining of a 60S ribosomal subunit results in the formation of a functional 80S initiation complex (80S.mRNA.Met-tRNA[F]).
EIF5A	Eukaryotic translation initiation factor 5A-1		5.08	25	18	1.4	86.08	-0.279	Translation regulatory protein	Protein metabolism	Cytoplasm; Nucleus; Nucleolus; Mitochondrion	mRNA-binding protein involved in translation elongation. Has an important function at the level of mRNA turnover, probably acting downstream of decapping. Involved in actin dynamics and cell cycle progression, mRNA decay and probably in a pathway involved in stress response and maintenance of cell wall integrity.
EIF5AL1	Eukaryotic translation initiation factor 5A-1-like		4.85	25	16	1.6	86.73	-0.220	**Translation regulatory protein**	**Protein metabolism**	**Cytoplasm; Nucleus**	**mRNA-binding protein involved in translation elongation. Has an important function at the level of mRNA turnover, probably acting downstream of decapping. Involved in actin dynamics and cell cycle progression, mRNA decay and probably in a pathway involved in stress response and maintenance of cell wall integrity. Functions as a regulator of apoptosis. Mediates effects of polyamines on neuronal process extension and survival. May play an important role in brain development and function, and in skeletal muscle stem cell differentiation By similarity.**
EIF5B	Eukaryotic translation initiation factor 5B		5.39	271	235	1.2	72.02	-1.049	Translation regulatory protein	Protein metabolism	Cytoplasm; Nucleus; Nucleolus	Function in general translation initiation by promoting the binding of the formylmethionine-tRNA to ribosomes. Seems to function along with eIF-2 By similarity.
XPO1	Exportin-1		5.71	127	101	1.3	99.09	-0.092	Transport/cargo protein	Cell communication; Signal transduction	Nucleus; Cytoplasm; Nucleolus; Kinetochore; Cytosol; Microtubule	Mediates the nuclear export of cellular proteins (cargos) bearing a leucine-rich nuclear export signal (NES) and of RNAs. In the nucleus, in association with RANBP3, binds cooperatively to the NES on its target protein and to the GTPase RAN in its active GTP-bound form (Ran-GTP).
SOD3	Extracellular superoxide dismutase [Cu-Zn]		6.32	28	25	1.1	66.44	-0.550	Enzyme: Superoxide dismutase	Metabolism; Energy pathways	Extracellular	Protect the extracellular space from toxic effect of reactive oxygen intermediates by converting superoxide radicals into hydrogen peroxide and oxygen.
EZR	Ezrin		5.95	111	102	1.1	77.21	-0.976	Anchor protein	Cell growth and/or maintenance	Cytoplasm; Plasma membrane; Mitochondrion	Probably involved in connections of major cytoskeletal structures to the plasma membrane. In epithelial cells, required for the formation of microvilli and membrane ruffles on the apical pole. Along with PLEKHG6, required for normal macropinocytosis.
**FASN**	**Fatty acid synthase**	[1]	6.01	265	227	1.2	94.50	-0.070	Enzyme: Synthase	Metabolism; Energy pathways	Cytoplasm; Cytosol; Mitochondrion; Nucleus	Fatty acid synthetase catalyzes the formation of long-chain fatty acids from acetyl-CoA, malonyl-CoA and NADPH. This multifunctional protein has 7 catalytic activities and an acyl carrier protein.
**FABP5**	**Fatty acid-binding protein, epidermal**	[10, 12]	6.82	20	20	1.0	72.69	-0.475	Transport/cargo protein	Transport	Cytoplasm; Endoplasmic reticulum	High specificity for fatty acids. Highest affinity for C18 chain length. Decreasing the chain length or introducing double bonds reduces the affinity. May be involved in keratinocyte differentiation.
**FBXO2**	**F-box only protein 2**	[10]	4.29	62	24	2.6	72.13	-0.577	Ubiquitin proteasome system protein	Protein metabolism	Cytoplasm	Substrate recognition component of a SCF (SKP1-CUL1-F-box protein) E3 ubiquitin-protein ligase complex that mediates the ubiquitination and subsequent proteasomal degradation of target proteins. Involved in the endoplasmic reticulum-associated degradation pathway (ERAD) for misfolded lumenal proteins by recognizing and binding sugar chains on unfolded glycoproteins that are retrotranslocated into the cytosol and promoting their ubiquitination and subsequent degradation. Prevents formation of cytosolic aggregates of unfolded glycoproteins that have been retrotranslocated into the cytosol. Able to recognize and bind denatured glycoproteins, preferentially those of the high-mannose type (By similarity).
**FBXL18**	**F-box/LRR-repeat protein 18**	[3]	8.73	63	77	0.8	101.25	0.065	**Unclassified**	**Unknown**	**cytosol; endoplasmic reticulum; golgi apparatus; nucleus; peroxisome; plasma membrane**	**Substrate-recognition component of the SCF (SKP1-CUL1-F-box protein)-type E3 ubiquitin ligase complex.**
**FER1L5**	**Fer-1-like protein 5**	[1]	8.19	236	245	1.0	82.02	-0.447	**Unclassified**	**Unknown**	**Cytoplasm**	**Plays a role in myoblast fusion; probable mediator of endocytic recycling for membrane trafficking events during myotube formation.**
**FRMPD3**	**FERM and PDZ domain-containing protein 3**	[1]	8.44	207	218	0.9	76.75	-0.547	**Unclassified**	**Unknown**	**Nucleus; Cytoskeleton**	**?**
FERMT3	Fermitin family homolog 3		6.53	87	83	1.0	88.34	-0.384	Cytoskeletal associated protein	Cytoskeletal anchoring	Cytoplasm; Plasma membrane	Plays a central role in cell adhesion in hematopoietic cells. Acts by activating the integrin beta-1-3 (ITGB1, ITGB2 and ITGB3). Required for integrin-mediated platelet adhesion and leukocyte adhesion to endothelial cells. Required for activation of integrin beta-2 (ITGB2) in polymorphonuclear granulocytes (PMNs).
FRRS1	Ferric-chelate reductase 1		7.11	49	48	1.0	89.07	0.065	Enzyme: Oxygenase	Metabolism; Energy pathways	Integral to membrane	Ferric-chelate reductases reduce Fe3+ to Fe2+ before its transport from the endosome to the cytoplasm.
FETUB	Fetuin-B		6.52	40	39	1.0	72.26	-0.444	**Secreted polypeptide**	**Negative regulation of endopeptidase activity**	**Extracellular**	**Protease inhibitor required for egg fertilization.**
FBN1	Fibrillin-1		4.79	372	239	1.6	53.09	-0.430	Extracellular matrix protein	Cell growth and/or maintenance	Extracellular	Fibrillins are structural components of 10-12 nm extracellular calcium-binding microfibrils, which occur either in association with elastin or in elastin-free bundles. Fibrillin-1-containing microfibrils provide long-term force bearing structural support.
**FGA**	**Fibrinogen alpha chain**	[1, 3-5, 8, 10+C379]	5.79	110	95	1.2	51.37	-0.885	Coagulation factor	Protein metabolism	Extracellular	Fibrinogen has a double function: yielding monomers that polymerize into fibrin and acting as a cofactor in platelet aggregation.
**FGB**	**Fibrinogen beta chain**	[1, 3-5, 8]	7.95	55	57	1.0	58.88	-0.849	Coagulation factor	Protein metabolism	Extracellular; Endoplasmic reticulum	Fibrinogen has a double function: yielding monomers that polymerize into fibrin and acting as a cofactor in platelet aggregation.
**FGG**	**Fibrinogen gamma chain**	[1, 3-5, 8]	5.24	60	45	1.3	64.00	-0.682	Coagulation factor	Protein metabolism	Extracellular; Golgi apparatus	Fibrinogen has a double function: yielding monomers that polymerize into fibrin and acting as a cofactor in platelet aggregation.
**PKHD1**	**Fibrocystin**	[4]	6.11	366	312	1.2	91.89	-0.029	**Cell surface receptor**	**Cell communication; Signal transduction**	**Plasma membrane; Cytoplasm; Cell projection**	**May be required for correct bipolar cell division through the regulation of centrosome duplication and mitotic spindle assembly. May be a receptor protein that acts in collecting-duct and biliary differentiation.**
**FGL2**	**Fibroleukin (fibrinogen-like 2)**	[1]	6.83	57	56	1.0	68.63	-0.741	Extracellular matrix protein	Cell growth and/or maintenance	Extracellular	May play a role in physiologic lymphocyte functions at mucosal sites.
**FN1**	**Fibronectin**	[1, 4-6, 8]	5.39	259	199	1.3	66.02	-0.554	Extracellular matrix protein	Cell growth and/or maintenance	Extracellular; Endoplasmic reticulum; Nucleus	Fibronectins bind cell surfaces and various compounds including collagen, fibrin, heparin, DNA, and actin. Fibronectins are involved in cell adhesion, cell motility, opsonization, wound healing, and maintenance of cell shape healing, and maintenance of cell shape.
FBLN1	Fibulin-1		5.03	89	57	1.6	66.19	-0.313	Extracellular matrix protein	Cell growth and/or maintenance	Extracellular	Incorporated into fibronectin-containing matrix fibers. May play a role in cell adhesion and migration along protein fibers within the extracellular matrix (ECM). Could be important for certain developmental processes and contribute to the supramolecular organization of ECM architecture, in particular to those of basement membranes.

**FBLN5**	**Fibulin-5**	[2]	4.50	53	29	1.8	58.71	-0.466	Extracellular matrix protein	Cell growth and/or maintenance	Extracellular	Essential for elastic fiber formation, is involved in the assembly of continuous elastin (ELN) polymer and promotes the interaction of microfibrils and ELN (PubMed:18185537). Stabilizes and organizes elastic fibers in the skin, lung and vasculature (By similarity). Promotes adhesion of endothelial cells through interaction of integrins and the RGD motif. Vascular ligand for integrin receptors which may play a role in vascular development and remodeling.
FCN2	Ficolin-2		6.65	32	31	1.0	57.92	-0.616	Adhesion molecule	Immune response	Extracellular	May function in innate immunity through activation of the lectin complement pathway. Calcium-dependent and GlcNAc-binding lectin. Enhances phagocytosis of S.typhimurium by neutrophils, suggesting an opsonic effect via the collagen region.
FCN3	Ficolin-3		6.22	30	26	1.2	60.11	-0.593	Adhesion molecule	Immune response	Extracellular	May function in innate immunity through activation of the lectin complement pathway. Calcium-dependent and GlcNAc-binding lectin. Has affinity with GalNAc, GlcNAc, D-fucose, as mono/oligosaccharide and lipopolysaccharides from S.typhimurium and S.minnesota.
**FLG2**	**Filaggrin-2 (Ifapsoriasin)**	[11]	8.45	168	176	1.0	15.97	-1.366	Calcium binding protein	Cell communication; Signal transduction	Extracellular	Filament-associated protein that binds to keratin fibers in epithelial cells. Intermediate filament-associated and psoriasis susceptibility protein
**FLNB**	**Filamin-B**	[1]	5.47	320	256	1.3	77.38	-0.293	Cytoskeletal associated protein	Cell growth and/or maintenance	Cytoplasm; Nucleus; Plasma membrane	Connects cell membrane constituents to the actin cytoskeleton. May promote orthogonal branching of actin filaments and links actin filaments to membrane glycoproteins. Anchors various transmembrane proteins to the actin cytoskeleton.
**BLVRB**	**Flavin reductase (NADPH)**	[1]	7.31	20	20	1.0	92.68	-0.069	Enzyme: Oxidoreductase	Metabolism; Energy pathways	Cytoplasm	Broad specificity oxidoreductase that catalyzes the NADPH-dependent reduction of a variety of flavins, such as riboflavin, FAD or FMN, biliverdins, methemoglobin and PQQ (pyrroloquinoline quinone). Contributes to heme catabolism and metabolizes linear tetrapyrroles. Can also reduce the complexed Fe3+ iron to Fe2+ in the presence of FMN and NADPH.
FSTL4	Follistatin-related protein 4		5.81	96	76	1.3	88.77	-0.226	Extracellular matrix protein	Unknown	Extracellular	Calcium binding
FSTL5	Follistatin-related protein 5		5.65	108	88	1.2	85.39	-0.357	Extracellular matrix protein	Unknown	Extracellular	Calcium binding
FTCD	Formimidoyltransferase-cyclodeaminase		5.57	67	59	1.1	94.55	-0.072	Enzyme: MethyltransferaseEnzyme: Deaminase	Metabolism; Energy pathways; Amino acid and derivative metabolism	Golgi apparatus; Cytoplasm	Folate-dependent enzyme, that displays both transferase and deaminase activity. Serves to channel one-carbon units from formiminoglutamate to the folate pool. Binds and promotes bundling of vimentin filaments originating from the Golgi.
**ALDOB**	**Fructose-bisphosphate aldolase B**	[2]	8.06	36	38	0.9	85.56	-0.233	Enzyme: Lyase	Metabolism; Energy pathways	Cytoplasm	D-fructose 1,6-bisphosphate = glycerone phosphate + D-glyceraldehyde 3-phosphate.
**GPKOW**	**G patch domain and KOW motifs-containing protein**	[3]	5.85	70	64	1.1	74.91	-0.691	Unclassified	Unknown	Cytoplasm	Interacts directly with protein kinase A and protein kinase X and is also found associated with the spliceosome.
**LGALS3BP**	**Galectin-3-binding protein**	[1-2]	5.07	64	47	1.4	82.26	-0.158	Extracellular matrix protein	Immune response	Extracellular	Promotes intergrin-mediated cell adhesion. May stimulate host defense against viruses and tumor cells.
**LGALS7**	**Galectin-7**	[1]	7.02	16	16	1.0	85.88	-0.343	Cell junction protein	Cell growth and/or maintenance	Extracellular; Plasma membrane	Could be involved in cell-cell and/or cell-matrix interactions necessary for normal growth control. Pro-apoptotic protein that functions intracellularly upstream of JNK activation and cytochrome c release.
**GGH**	**Gamma-glutamyl hydrolase**	[9, 11]	7.19	32	32	1.0	81.26	-0.238	Enzyme: Hydrolase	Metabolism; Energy pathways	Lysosome; Extracellular; Plasma membrane	Hydrolyzes the polyglutamate sidechains of pteroylpolyglutamates. Progressively removes gamma-glutamyl residues from pteroylpoly-gamma-glutamate to yield pteroyl-alpha-glutamate (folic acid) and free glutamate. May play an important role in the bioavailability of dietary pteroylpolyglutamates and in the metabolism of pteroylpolyglutamates and antifolates.
**GGACT**	**Gamma-glutamylaminecyclotransferase**	[10]	6.37	19	17	1.1	78.43	-0.482	Unclassified	Unknown	Unknown	Contributes to degradation of proteins cross-linked by transglutaminases. Degrades the cross-link between a lysine and a glutamic acid residue from two proteins that have been cross-linked by transglutaminases. Catalyzes the formation of 5-oxoproline from L-gamma-glutamyl-L-epsilon-lysine.
APP	Gamma-secretase C-terminal fragment 59		7.03	6	6	1.0	85.76	-0.341	Cell surface receptor	Cell communication; Signal transduction	Nucleus; Integral to membrane; Cell surface; Vesicle; Endoplasmic reticulum; Golgi apparatus; Endosome; Cytoplasm; Extracellular; Plasma membrane	Functions as a cell surface receptor and performs physiological functions on the surface of neurons relevant to neurite growth, neuronal adhesion and axonogenesis. Involved in cell mobility and transcription regulation through protein-protein interactions. Can regulate neurite outgrowth through binding to components of the extracellular matrix such as heparin and collagen I and IV. Beta-amyloid peptides are lipophilic metal chelators with metal-reducing activity. Bind transient metals such as copper, zinc and iron. In vitro, can reduce Cu2+ and Fe3+ to Cu+ and Fe2+, respectively.
**GM2A**	**Ganglioside GM2 activator**	[2]	4.76	20	13	1.5	96.17	0.107	Transport/cargo protein	Metabolism; Energy pathways	Lysosome; Extracellular	The large binding pocket can accommodate several single chain phospholipids and fatty acids, GM2A also exhibits some calcium-independent phospholipase activity By similarity. Binds gangliosides and stimulates ganglioside GM2 degradation. It stimulates only the breakdown of ganglioside GM2 and glycolipid GA2 by beta-hexosaminidase A.
TSTA3	GDP-L-fucose synthase		6.12	37	32	1.2	77.73	-0.258	Enzyme: Oxidoreductase	Metabolism; Energy pathways	Cytoplasm	Catalyzes the two-step NADP-dependent conversion of GDP-4-dehydro-6-deoxy-D-mannose to GDP-fucose, involving an epimerase and a reductase reaction.
**GSN**	**Gelsolin**	[1-2]	5.72	97	85	1.1	73.40	-0.469	Cytoskeletal protein	Cell growth and/or maintenance	Cytoplasm; Extracellular; Mitochondrion; Cytoskeleton	Calcium-regulated, actin-modulating protein that binds to the plus (or barbed) ends of actin monomers or filaments, preventing monomer exchange (end-blocking or capping). It can promote the assembly of monomers into filaments (nucleation) as well as sever filaments already formed. Plays a role in ciliogenesis.
**GTF2IRD1**	**General transcription factor II-I repeat domain-containing protein 1**	[3]	6.45	132	127	1.0	84.08	-0.427	Transcription factor	Regulation of nucleobase, nucleoside, nucleotide and nucleic acid metabolism	Nucleus; Cytoplasm	May be a transcription regulator involved in cell-cycle progression and skeletal muscle differentiation. May repress GTF2I transcriptional functions, by preventing its nuclear residency, or by inhibiting its transcriptional activation. May contribute to slow-twitch fiber type specificity during myogenesis and in regenerating muscles.
USO1	General vesicular transport factor p115		4.85	142	93	1.5	97.08	-0.388	Transport/cargo protein	Transport	Golgi apparatus; Cytoplasm; Nucleus; Cytosol	General vesicular transport factor required for intercisternal transport in the Golgi stack; it is required for transcytotic fusion and/or subsequent binding of the vesicles to the target membrane.
**CCDC88A**	**Girdin**	[1]	5.90	325	294	1.1	82.18	-0.949	Cytoskeletal protein	Cell growth and/or maintenance; Cell migration	Cytoskeleton; Plasma membrane	Plays a role as a key modulator of the AKT-mTOR signaling pathway controlling the tempo of the process of newborn neurons integration during adult neurogenesis, including correct neuron positioning, dendritic development and synapse formation. Enhances phosphoinositide 3-kinase (PI3K)-dependent phosphorylation and kinase activity of AKT1/PKB, but does not possess kinase activity itself.
GMFG	Glia maturation factor gamma		5.18	25	21	1.2	76.76	-0.491	Growth factor	Cell communication; Signal transduction	Intracellular	GO annotations related to this gene include actin binding and enzyme activator activity.
GFAP	Glial fibrillary acidic protein		5.42	81	68	1.2	86.60	-0.773	Structural protein	Cell growth and/or maintenance	Cytoplasm	GFAP, a class-III intermediate filament, is a cell-specific marker that, during the development of the central nervous system, distinguishes astrocytes from other glial cells.
**GLTSCR1L**	**GLTSCR1-like protein (KIAA0240)**	[1]	6.38	86	75	1.1	72.02	-0.482	Unclassified	Unknown	Unknown	?
GLCCI1	Glucocorticoid-induced transcript 1 protein		9.54	45	64	0.7	56.44	-0.738	Unclassified	Unknown	Nucleus; Cytoplasm	Expression of this gene is induced by glucocorticoids and may be an early marker for glucocorticoid-induced apoptosis.
G6PD	Glucose-6-phosphate 1-dehydrogenase		6.41	67	63	1.1	83.64	-0.375	Enzyme: Dehydrogenase	Metabolism; Energy pathways	Endoplasmic reticulum; Cytoplasm	Catalyzes the rate-limiting step of the oxidative pentose-phosphate pathway, which represents a route for the dissimilation of carbohydrates besides glycolysis. The main function of this enzyme is to provide reducing power (NADPH) and pentose phosphates for fatty acid and nucleic acid synthesis.
GPI	Glucose-6-phosphate isomerase		8.44	59	62	1.0	83.36	-0.348	Enzyme: Isomerase	Metabolism; Energy pathways	Cytoplasm; Mitochondrion	Besides it's role as a glycolytic enzyme, mammalian GPI can function as a tumor-secreted cytokine and an angiogenic factor (AMF) that stimulates endothelial cell motility. GPI is also a neurotrophic factor (Neuroleukin) for spinal and sensory neurons.
PRKCSH	Glucosidase 2 subunit beta		4.33	119	53	2.2	53.40	-0.995	Regulatory/other subunit	Energy pathways; Metabolism	Endoplasmic reticulum	Regulatory subunit of glucosidase II.
GCLC	Glutamate--cysteine ligase catalytic subunit		5.74	91	77	1.2	81.10	-0.443	Enzyme: Ligase	Metabolism; Energy pathways	Cytoplasm	ATP + L-glutamate + L-cysteine = ADP + phosphate + gamma-L-glutamyl-L-cysteine.
GCLM	Glutamate--cysteine ligase regulatory subunit		5.70	35	29	1.2	97.15	-0.299	Enzyme: Ligase	Metabolism; Energy pathways	Cytoplasm	ATP + L-glutamate + L-cysteine = ADP + phosphate + gamma-L-glutamyl-L-cysteine.
GFPT1	Glutamine--fructose-6-phosphate aminotransferase [isomerizing] 1		6.68	93	89	1.0	90.33	-0.281	Enzyme: Aminotransferase	Metabolism; Energy pathways	Cytoplasm	Controls the flux of glucose into the hexosamine pathway. Most likely involved in regulating the availability of precursors for N- and O-linked glycosylation of proteins. Regulates the circadian expression of clock genes ARNTL/BMAL1 and CRY1.
QARS	Glutamine--tRNA ligase		6.74	100	96	1.0	85.18	-0.310	Enzyme: Ligase	Metabolism; Energy pathways	Cytoplasm; Cytosol	Plays a critical role in brain development. ATP + L-glutamine + tRNA(Gln) = AMP + diphosphate + L-glutaminyl-tRNA(Gln).
**QPCT**	**Glutaminyl-peptide cyclotransferase**	[1]	5.69	38	26	1.5	90.54	-0.291	Enzyme: Cyclotransferase	Metabolism; Energy pathways	Extracellular	Responsible for the biosynthesis of pyroglutamyl peptides. May be involved in the N-terminal pyroglutamate formation of several amyloid-related plaque-forming peptides
GPX1	Glutathione peroxidase 1		6.15	21	20	1.1	86.11	-0.070	Enzyme: Peroxidase	Anti-apoptosis	Cytoplasm; Mitochondrion; Cytosol	Protects the hemoglobin in erythrocytes from oxidative breakdown.
GSR	Glutathione reductase, mitochondrial		7.61	50	51	1.0	86.70	-0.084	Enzyme: Reductase	Metabolism; Energy pathways	Cytoplasm; Mitochondrion	Maintains high levels of reduced glutathione in the cytosol.
**GSTA1**	**Glutathione S-transferase A1**	[1]	8.92	32	36	0.9	99.77	-0.292	Enzyme: Glutathione transferase	Metabolism; Energy pathways	Cytoplasm; Extracellular	Conjugation of reduced glutathione to a wide number of exogenous and endogenous hydrophobic electrophiles.
GSTA3	Glutathione S-transferase A3		9.22	29	35	0.8	97.60	-0.273	Enzyme: Glutathione transferase	Metabolism; Energy pathways	Cytoplasm	Conjugation of reduced glutathione to a wide number of exogenous and endogenous hydrophobic electrophiles. Catalyzes isomerization reactions that contribute to the biosynthesis of steroid hormones.
**GSTM3**	**Glutathione S-transferase Mu 3**	[4]	5.37	34	29	1.2	80.22	-0.406	Enzyme: Glutathione transferase	Metabolism; Energy pathways	Cytoplasm	Conjugation of reduced glutathione to a wide number of exogenous and endogenous hydrophobic electrophiles. May govern uptake and detoxification of both endogenous compounds and xenobiotics at the testis and brain blood barriers.
GSTP1	Glutathione S-transferase P		5.44	23	20	1.2	99.38	-0.131	Enzyme: Glutathione transferase	Metabolism; Energy pathways	Cytoplasm; Nucleus; Mitochondrion	Conjugation of reduced glutathione to a wide number of exogenous and endogenous hydrophobic electrophiles. Regulates negatively CDK5 activity via p25/p35 translocation to prevent neurodegeneration.
**GAPDH**	**Glyceraldehyde-3-phosphate dehydrogenase**	[1, 5, 8]	8.58	33	36	0.9	84.94	-0.114	Enzyme: Dehydrogenase	Metabolism; Energy pathways	Cytoplasm; Nucleus; Nucleolus; Extracellular; Mitochondrion	Has both glyceraldehyde-3-phosphate dehydrogenase and nitrosylase activities, thereby playing a role in glycolysis and nuclear functions, respectively. Participates in nuclear events including transcription, RNA transport, DNA replication and apoptosis.Glyceraldehyde-3-phosphate dehydrogenase is a key enzyme in glycolysis that catalyzes the first step of the pathway by converting D-glyceraldehyde 3-phosphate (G3P) into 3-phospho-D-glyceroyl phosphate.
GAPDHS	Glyceraldehyde-3-phosphate dehydrogenase, testis-specific		8.39	41	44	0.9	81.91	-0.210	Enzyme: Dehydrogenase	Metabolism; Energy pathways	Cell projection	May play an important role in regulating the switch between different pathways for energy production during spermiogenesis and in the spermatozoon. Required for sperm motility and male fertility By similarity.
GDPD3	Glycerophosphodiester phosphodiesterase domain-containing protein 3		8.13	36	38	0.9	92.30	-0.104	Enzyme: Hydrolase	Metabolism; Energy pathways	Membrane; Extracellular vesicular exosome	GO annotations related to this gene include glycerophosphodiester phosphodiesterase activity.
PYGB	Glycogen phosphorylase, brain form		6.41	116	109	1.1	89.14	-0.335	Enzyme: Phosphorylase	Metabolism; Energy pathways	Cytoplasm; Nucleus	Phosphorylase is an important allosteric enzyme in carbohydrate metabolism. Enzymes from different sources differ in their regulatory mechanisms and in their natural substrates. However, all known phosphorylases share catalytic and structural properties.
PYGL	Glycogen phosphorylase, liver form		6.74	112	109	1.0	90.93	-0.326	Enzyme: Phosphorylase	Metabolism; Energy pathways	Cytoplasm; Plasma membrane	Phosphorylase is an important allosteric enzyme in carbohydrate metabolism. Enzymes from different sources differ in their regulatory mechanisms and in their natural substrates. However, all known phosphorylases share catalytic and structural properties.
GRHPR	Glyoxylate reductase/hydroxypyruvate reductase		7.01	37	37	1.0	100.85	-0.015	Enzyme: Reductase	Metabolism; Energy pathways	Cytoplasm	Enzyme with hydroxy-pyruvate reductase, glyoxylate reductase and D-glycerate dehydrogenase enzymatic activities. Reduces hydroxypyruvate to D-glycerate, glyoxylate to glycolate oxidizes D-glycerate to hydroxypyruvate.
GPC3	Glypican-3		Unknown	Unknown	Unknown	-	Unknown	Unknown	Integral membrane protein	Cell communication; Signal transduction	Extracellular; Plasma membrane	Cell surface proteoglycan that bears heparan sulfate. Inhibits the dipeptidyl peptidase activity of DPP4. May be involved in the suppression/modulation of growth in the predominantly mesodermal tissues and organs. May play a role in the modulation of IGF2 interactions with its receptor and thereby modulate its function. May regulate growth and tumor predisposition.
GPC4	Glypican-4		5.91	67	60	1.1	71.55	-0.453	Integral membrane protein	Cell communication; Signal transduction	Plasma membrane	Cell surface proteoglycan that bears heparan sulfate. May be involved in the development of kidney tubules and of the central nervous system By similarity.
**GRHL1**	**Grainyhead-like protein 1 homolog**	[1]	6.26	86	79	1.1	76.78	-0.579	Transcription regulatory protein	Regulation of nucleobase, nucleoside, nucleotide and nucleic acid metabolism	Nucleus	Transcription factor. Isoform 1 may function as an activator and isoform 2 as a repressor in tissues where both forms are expressed. May play a role in development. May be involved in epidermal differentiation (By similarity). Binds and transactivates the EN1 promoter.
GRN	Granulins		various	various	various	-	various	various	Growth factor	Cell communication; Signal transduction	Extracellular; Cytoplasm	Granulins have possible cytokine-like activity. They may play a role in inflammation, wound repair, and tissue remodeling.
GAS6	Growth arrest-specific protein 6		5.78	90	76	1.2	75.07	-0.351	Growth factor	Cell communication; Signal transduction	Extracellular	Ligand for tyrosine-protein kinase receptors AXL, TYRO3 and MER whose signaling is implicated in cell growth and survival, cell adhesion and cell migration. GAS6/AXL signaling plays a role in various processes such as endothelial cell survival during acidification by preventing apoptosis, optimal cytokine signaling during human natural killer cell development, hepatic regeneration, gonadotropin-releasing hormone neuron survival and migration, platelet activation, or regulation of
GDF15	Growth/differentiation factor 15		8.17	10	12	0.8	81.32	-0.152	Growth factor	Cell communication; Signal transduction	Extracellular; Endoplasmic reticulum; Golgi apparatus	Bone morphogenetic proteins (e.g., BMP9; MIM 605120) are members of the transforming growth factor-beta (see TGFB1; MIM 190180) superfamily and regulate tissue differentiation and maintenance.

RAN	GTP-binding nuclear protein Ran		7.20	27	27	1.0	88.37	-0.276	GTPase	Cell communication; Signal transduction	Nucleus; Cytoplasm; Mitochondrion	GTP-binding protein involved in nucleocytoplasmic transport. Required for the import of protein into the nucleus and also for RNA export. Involved in chromatin condensation and control of cell cycle By similarity.
GNAI1	Guanine nucleotide-binding protein G(i) subunit alpha-1		5.70	54	48	1.1	82.95	-0.334	G protein	Cell communication; Signal transduction	Plasma membrane; Nucleus; Cytoplasm	Guanine nucleotide-binding proteins (G proteins) are involved as modulators or transducers in various transmembrane signaling systems. The G(i) proteins are involved in hormonal regulation of adenylate cyclase: they inhibit the cyclase in response to beta-adrenergic stimuli.
GNAI2	Guanine nucleotide-binding protein G(i) subunit alpha-2		5.34	56	45	1.2	80.76	-0.370	GTPase; G protein	Cell communication; Signal transduction	Plasma membrane; Cytoplasm; Endoplasmic reticulum; Cytosol	Guanine nucleotide-binding proteins (G proteins) are involved as modulators or transducers in various transmembrane signaling systems. The G(i) proteins are involved in hormonal regulation of adenylate cyclase: they inhibit the cyclase in response to beta-adrenergic stimuli. May play a role in cell division.
GNB1	Guanine nucleotide-binding protein G(I)/G(S)/G(T) subunit beta-1		5.60	39	32	1.2	78.61	-0.237	G protein	Cell communication; Signal transduction	Plasma membrane; Mitochondrion	Guanine nucleotide-binding proteins (G proteins) are involved as a modulator or transducer in various transmembrane signaling systems. The beta and gamma chains are required for the GTPase activity, for replacement of GDP by GTP, and for G protein-effector interaction.
GNB2	Guanine nucleotide-binding protein G(I)/G(S)/G(T) subunit beta-2		5.60	39	32	1.2	80.32	-0.183	G protein	Cell communication; Signal transduction	Cytoplasm	Guanine nucleotide-binding proteins (G proteins) are involved as a modulator or transducer in various transmembrane signaling systems. The beta and gamma chains are required for the GTPase activity, for replacement of GDP by GTP, and for G protein-effector interaction.
GNB2L1	Guanine nucleotide-binding protein subunit beta-2-like 1		7.57	30	31	1.0	82.94	-0.258	Adapter molecule	Cell communication; Signal transduction	Cytoplasm; Nucleus; Plasma membrane; Cytoskeleton	Involved in the recruitment, assembly and/or regulation of a variety of signaling molecules. Interacts with a wide variety of proteins and plays a role in many cellular processes. Component of the 40S ribosomal subunit involved in translational repression. Binds to and stabilizes activated protein kinase C (PKC), increasing PKC-mediated phosphorylation. May recruit activated PKC to the ribosome, leading to phosphorylation of EIF6.
GNL1	Guanine nucleotide-binding protein-like 1		5.57	95	78	1.2	74.51	-0.714	GTPase	Cell communication; Signal transduction	Nucleus; Cytoplasm	Possible regulatory or functional link with the histocompatibility cluster.
**HP**	**Haptoglobin**	**[1, 3, 5-6]**	6.13	50	44	1.1	76.08	-0.527	Transport/cargo protein	Immune response	Extracellular	Haptoglobin captures, and combines with free plasma hemoglobin to allow hepatic recycling of heme iron and to prevent kidney damage. Haptoglobin also acts as an Antimicrobial; Antioxidant, has antibacterial activity and plays a role in modulating many aspects of the acute phase response. ncleaved haptoglogin, also known as zonulin, plays a role in intestinal permeability, allowing intercellular tight junction disassembly, and controlling the equilibrium between tolerance and immunity to non-
**HPR**	**Haptoglobin-related protein**	**[1]**	6.67	37	35	1.1	82.04	-0.389	Secreted polypeptide	Immune response	Extracellular	Haptoglobin-related protein binds hemoglobin as efficiently as haptoglobin. Unlike haptoglobin, plasma concentration of this protein is unaffected in patients with sickle cell anemia and extensive intravascular hemolysis, suggesting a difference in binding between haptoglobin-hemoglobin and haptoglobin-related protein-hemoglobin complexes to CD163, the hemoglobin scavenger receptor.
**HSPA4**	**Heat shock 70 kDa protein 4**	**[1]**	5.10	137	110	1.2	74.86	-0.581	Chaperone	Protein metabolism	Golgi apparatus; Plasma membrane; Extracellular; Nucleus; Cytoplasm; Cell surface	Protein import into mitochondrial outer membrane.
**HSPA8**	**Heat shock cognate 71 kDa protein**	**[1]**	5.37	95	82	1.2	81.64	-0.460	Heat shock protein	Protein metabolism	Cytoplasm; Nucleus; Nucleolus; Mitochondrion; Cytosol	Acts as a repressor of transcriptional activation. Inhibits the transcriptional coactivator activity of CITED1 on Smad-mediated transcription. Chaperone. Component of the PRP19-CDC5L complex that forms an integral part of the spliceosome and is required for activating pre-mRNA splicing. May have a scaffolding role in the spliceosome assembly as it contacts all other components of the core complex. Binds bacterial lipopolysaccharide (LPS) et mediates LPS-induced inflammatory response, including TNF secretion by monocytes.
HSPH1	Heat shock protein 105 kDa		5.27	138	112	1.2	76.77	-0.574	Heat shock protein; Chaperone	Protein metabolism	Cytoplasm; Cytosol; Nucleus	Prevents the aggregation of denatured proteins in cells under severe stress, on which the ATP levels decrease markedly. Inhibits HSPA8/HSC70 ATPase and chaperone activities By similarity.
**TRAP1**	**Heat shock protein 75 kDa, mitochondrial**	**[1, 3]**	6.13	93	85	1.1	90.14	-0.364	**Heat shock protein**	**Protein metabolism**	**Mitochondrion**	**Chaperone that expresses an ATPase activity. Involved in maintaining mitochondrial function and polarization, most likely through stabilization of mitochondrial complex I. Is a negative regulator of mitochondrial respiration able to modulate the balance between oxidative phosphorylation and aerobic glycolysis. The impact of TRAP1 on mitochondrial respiration is probably mediated by modulation of mitochondrial SRC and inhibition of SDHA.**
**HSPB1**	**Heat shock protein beta-1 (hsp27)**	**[2-5, 7-8, 9]**	5.98	26	23	1.1	68.54	-0.567	Chaperone	Protein metabolism	Cytoplasm; Nucleus; Plasma membrane; Microtubule	Involved in stress resistance and actin organization.
**HSP90AA1**	**Heat shock protein HSP 90-alpha**	**[1, 3, 4, 10]**	4.94	151	110	1.4	79.48	-0.754	Chaperone	Protein metabolism	Cytoplasm; Nucleus; Extracellular space; Microtubule; Mitochondrion	Molecular chaperone that promotes the maturation, structural maintenance and proper regulation of specific target proteins involved for instance in cell cycle control and signal transduction. Undergoes a functional cycle that is linked to its ATPase activity. This cycle probably induces conformational changes in the client proteins, thereby causing their activation. Interacts dynamically with various co-chaperones that modulate its substrate recognition, ATPase cycle and chaperone function By similarity. Binds bacterial lipopolysaccharide (LPS) et mediates LPS-induced inflammatory response, including TNF secretion By similarity.
**HSP90AB1**	**Heat shock protein HSP 90-beta**	**[1, 3-4]**	4.96	147	107	1.4	81.44	-0.682	Chaperone	Cell communication; Signal transduction	Cytoplasm; Nucleus; Nucleolus; Cytosol; Microtubule; Mitochondrion	Molecular chaperone that promotes the maturation, structural maintenance and proper regulation of specific target proteins involved for instance in cell cycle control and signal transduction. Undergoes a functional cycle that is linked to its ATPase activity. This cycle probably induces conformational changes in the client proteins, thereby causing their activation. Interacts dynamically with various co-chaperones that modulate its substrate recognition, ATPase cycle and chaperone function By
**HMCN1**	**Hemicentin 1**	**[2]**	6.08	575	511	1.1	85.24	-0.246	**Extracellular matrix protein**	**Cell communication; Signal transduction**	**Extracellular**	**Large extracellular member of the immunoglobulin superfamily. A similar protein in C. elegans forms long, fine tracks at specific extracellular sites that are involved in many processes such as stabilization of the germline syncytium, anchorage of mechanosensory neurons to the epidermis, and organization of hemidesmosomes in the epidermis.**
**HBA1**	**Hemoglobin subunit alpha**	**[1, 4-8, 10, 12]**	8.73	12	14	0.9	91.42	0.035	**Transport/cargo protein**	**Transport**	**Cytosol**	**Involved in oxygen transport from the lung to the various peripheral tissues.**
**HBB**	**Hemoglobin subunit beta**	**[1, 3-10, 12]**	6.81	15	14	1.1	94.11	0.001	**Transport/cargo protein**	**Transport**	**Extracellular**	**Involved in oxygen transport from the lung to the various peripheral tissues.**
**HBD**	**Hemoglobin subunit delta**	**[1]**	7.97	14	15	0.9	92.12	-0.059	**Transport/cargo protein**	**Transport**	**Cytosol**	**Involved in oxygen transport from the lung to the various peripheral tissues.**
HBE1	Hemoglobin subunit epsilon		8.69	14	16	0.9	93.56	0.014	**Transport/cargo protein**	**Transport**	**Cytopalsm; Nucleus**	**The epsilon chain is a beta-type chain of early mammalian embryonic hemoglobin.**
**HBG1**	**Hemoglobin subunit gamma-1**	**[1]**	6.71	16	15	1.1	90.14	-0.103	Transport/cargo protein	Transport	Cytosol	Gamma chains make up the fetal hemoglobin F, in combination with alpha chains.
HBG2	Hemoglobin subunit gamma-2		6.71	16	15	1.1	89.45	-0.118	Transport/cargo protein	Transport	Cytosol	Gamma chains make up the fetal hemoglobin F, in combination with alpha chains.
**HPX**	**Hemopexin**	**[1, 8]**	6.43	49	44	1.1	63.10	-0.537	**Transport/cargo protein**	Transport	**Extracellular**	**Binds heme and transports it to the liver for breakdown and iron recovery, after which the free hemopexin returns to the circulation.**
**SERPIND1**	**Heparin cofactor 2**	**[1]**	6.26	59	54	1.1	88.73	-0.292	Protease inhibitor	Protein metabolism	Extracellular	Thrombin inhibitor activated by the glycosaminoglycans, heparin or dermatan sulfate. In the presence of the latter, HC-II becomes the predominant thrombin inhibitor in place of antithrombin III (AT-III). Also inhibits chymotrypsin, but in a glycosaminoglycan-independent manner.
HNRNPA0	Heterogeneous nuclear ribonucleoprotein A0		9.34	25	34	0.7	46.03	-0.594	Ribonucleoprotein	Regulation of nucleobase, nucleoside, nucleotide and nucleic acid metabolism	Nucleus; Nucleolus; Cytoplasm; Mitochondrion	mRNA-binding component of ribonucleosomes. Specifically binds AU-rich element (ARE)-containing mRNAs. Involved in post-transcriptional regulation of cytokines mRNAs.
HNRNPA1	Heterogeneous nuclear ribonucleoprotein A1		9.17	36	43	0.8	33.07	-0.901	RNA binding protein	Regulation of nucleobase, nucleoside, nucleotide and nucleic acid metabolism	Nucleus; Nucleolus; Cytoplasm; Mitochondrion	Involved in the packaging of pre-mRNA into hnRNP particles, transport of poly(A) mRNA from the nucleus to the cytoplasm and may modulate splice site selection. May play a role in HCV RNA replication.
HNRNPD	Heterogeneous nuclear ribonucleoprotein D0		7.61	45	46	1.0	41.30	-0.924	RNA binding protein	Regulation of nucleobase, nucleoside, nucleotide and nucleic acid metabolism	Nucleus; Cytoplasm; Mitochondrion	Binds with high affinity to RNA molecules that contain AU-rich elements (AREs) found within the 3'-UTR of many proto-oncogenes and cytokine mRNAs. Also binds to double- and single-stranded DNA sequences in a specific manner and functions a transcription factor. May be involved in translationally coupled mRNA turnover.
HNRNPF	Heterogeneous nuclear ribonucleoprotein F		5.37	55	40	1.4	62.63	-0.477	Ribonucleoprotein	Regulation of nucleobase, nucleoside, nucleotide and nucleic acid metabolism	Nucleus; Nucleolus; Cytoplasm	Component of the heterogeneous nuclear ribonucleoprotein (hnRNP) complexes which provide the substrate for the processing events that pre-mRNAs undergo before becoming functional, translatable mRNAs in the cytoplasm. Plays a role in the regulation of alternative splicing events. Binds G-rich sequences in pre-mRNAs and keeps target RNA in an unfolded state.
HNRNPH1	Heterogeneous nuclear ribonucleoprotein H		5.89	55	47	1.2	54.87	-0.608	Ribonucleoprotein	Regulation of nucleobase, nucleoside, nucleotide and nucleic acid metabolism	Nucleus; Nucleolus; Cytoplasm	This protein is a component of the heterogeneous nuclear ribonucleoprotein (hnRNP) complexes which provide the substrate for the processing events that pre-mRNAs undergo before becoming functional, translatable mRNAs in the cytoplasm. Mediates pre-mRNA alternative splicing regulation.
HNRNPK	Heterogeneous nuclear ribonucleoprotein K		5.39	66	58	1.1	67.21	-0.705	Ribonucleoprotein	Regulation of nucleobase, nucleoside, nucleotide and nucleic acid metabolism	Nucleus; Nucleolus; Cytoplasm; Mitochondrion	One of the major pre-mRNA-binding proteins. Binds tenaciously to poly(C) sequences. Likely to play a role in the nuclear metabolism of hnRNAs, particularly for pre-mRNAs that contain cytidine-rich sequences. Can also bind poly(C) single-stranded DNA. Plays an important role in p53/TP53 response to DNA damage, acting at the level of both transcription activation and repression.
HNRNPL	Heterogeneous nuclear ribonucleoprotein L		8.46	60	65	0.9	60.59	-0.649	Ribonucleoprotein	Regulation of nucleobase, nucleoside, nucleotide and nucleic acid metabolism	Nucleus; Nucleolus; Cytoplasm; Mitochondrion	Splicing factor binding to exonic or intronic sites and acting as either an activator or repressor of exon inclusion. Exhibits a binding preference for CA-rich elements. Component of the heterogeneous nuclear ribonucleoprotein (hnRNP) complexes and associated with most nascent transcripts.
HNRNPM	Heterogeneous nuclear ribonucleoprotein M		8.85	88	94	0.9	66.94	-0.345	Ribonucleoprotein	Regulation of nucleobase, nucleoside, nucleotide and nucleic acid metabolism	Nucleus; Nucleolus; Cytoplasm; Mitochondrion	Pre-mRNA binding protein in vivo, binds avidly to poly(G) and poly(U) RNA homopolymers in vitro. Involved in splicing. Acts as a receptor for carcinoembryonic antigen in Kupffer cells, may initiate a series of signaling events leading to tyrosine phosphorylation of proteins and induction of IL-1 alpha, IL-6, IL-10 and tumor necrosis factor alpha cytokines.
HNRNPR	Heterogeneous nuclear ribonucleoprotein R		8.25	90	93	1.0	58.62	-0.937	RNA binding protein	Regulation of nucleobase, nucleoside, nucleotide and nucleic acid metabolism	Nucleus; Nucleolus; Mitochondrion	Component of ribonucleosomes, which are complexes of at least 20 other different heterogenious nuclear ribonucleoproteins (hnRNP). hnRNP play an important role in processing of precursor mRNA in the nucleus.
HNRNPA2B1	Heterogeneous nuclear ribonucleoproteins A2/B1		8.97	39	44	0.9	37.25	-0.931	Ribonucleoprotein; RNA binding protein	Regulation of nucleobase, nucleoside, nucleotide and nucleic acid metabolism	Nucleus; Nucleolus; Cytoplasm; Mitochondrion	Involved with pre-mRNA processing. Forms complexes (ribonucleosomes) with at least 20 other different hnRNP and heterogeneous nuclear RNA in the nucleus By similarity.
**HNRNPC**	**Heterogeneous nuclear ribonucleoproteins C1/C2**	**[3]**	4.95	59	47	1.3	62.66	-0.986	RNA binding protein	Regulation of nucleobase, nucleoside, nucleotide and nucleic acid metabolism	Nucleus; Nucleolus; Cytoplasm; Mitochondrion	Binds pre-mRNA and nucleates the assembly of 40S hnRNP particles. Single HNRNPC tetramers bind 230-240 nucleotides. Trimers of HNRNPC tetramers bind 700 nucleotides. May play a role in the early steps of spliceosome assembly and pre-mRNA splicing. Interacts with poly-U tracts in the 3'-UTR or 5'-UTR of mRNA and modulates the stability and the level of translation of bound mRNA molecules By similarity.
**HK2**	**Hexokinase-2**	**[3]**	5.71	135	113	1.2	87.36	-0.191	Enzyme: Phosphotransferase	Metabolism; Energy pathways	Mitochondrion	ATP + D-hexose = ADP + D-hexose 6-phosphate.
HK3	Hexokinase-3		5.23	103	76	1.4	97.06	0.100	**Enzyme: Phosphotransferase**	**Metabolism; Energy pathways**	**Cytoplasm; Extracellular**	**ATP + D-hexose = ADP + D-hexose 6-phosphate.**
**HMGB1**	**High mobility group protein B1**	**[3-4]**	5.60	56	51	1.1	30.23	-1.627	DNA binding protein	Regulation of nucleobase, nucleoside, nucleotide and nucleic acid metabolism	Nucleus; Cytoplasm; Extracellular; Plasma membrane; Mitochondrion	DNA binding proteins that associates with chromatin and has the ability to bend DNA. Binds preferentially single-stranded DNA. Involved in V(D)J recombination by acting as a cofactor of the RAG complex. Acts by stimulating cleavage and RAG protein binding at the 23 bp spacer of conserved recombination signal sequences (RSS). Heparin-binding protein that has a role in the extension of neurite-type cytoplasmic processes in developing cells By similarity.
**HMGB2**	**High mobility group protein B2**	**[4-5]**	7.77	48	49	1.0	24.52	-1.713	Transcription regulatory protein	Regulation of nucleobase, nucleoside, nucleotide and nucleic acid metabolism	Nucleus; Nucleolus; Cytoplasm; Mitochondrion	DNA binding proteins that associates with chromatin and has the ability to bend DNA. Binds preferentially single-stranded DNA. Involved in V(D)J recombination by acting as a cofactor of the RAG complex. Acts by stimulating cleavage and RAG protein binding at the 23 bp spacer of conserved recombination signal sequences (RSS) By similarity.
**HRG**	**Histidine-rich glycoprotein**	**[1, 3-5, 7-8]**	7.03	59	54	1.1	49.19	-1.049	Adapter molecule	Apoptosis	Extracellular; Plasma membrane	Plasma glycoprotein that binds a number of ligands such as heme, heparin, heparan sulfate, thrombospondin, plasminogen, and divalent metal ions. Binds heparin and heparin/glycosaminoglycans in a zinc-dependent manner. Binds heparan sulfate on the surface of liver, lung, kidney and heart endothelial cells. Binds to N-sulfated polysaccharide chains on the surface of liver endothelial cells. Inhibits rosette formation. Acts as an adapter protein and is implicated in regulating many processes such as immune complex and pathogen clearance, cell chemotaxis, cell adhesion, angiogenesis, coagulation and fibrinolysis.
**HIST1H1T**	**Histone H1t**	**[1]**	11.71	8	54	0.1	75.83	-0.688	**DNA binding protein**	**Regulation of nucleobase, nucleoside, nucleotide and nucleic acid metabolism**	**Nucleus; Cytoplasm**	**Histones H1 are necessary for the condensation of nucleosome chains into higher-order structures.**
**H2AFJ**	**Histone H2A**	**[1]**	10.90	9	26	0.3	99.14	-0.457	DNA binding protein	Regulation of nucleobase, nucleoside, nucleotide and nucleic acid metabolism	Nucleus	Core component of nucleosome. Nucleosomes wrap and compact DNA into chromatin, limiting DNA accessibility to the cellular machineries which require DNA as a template. Histones thereby play a central role in transcription regulation, DNA repair, DNA replication and chromosomal stability. DNA accessibility is regulated via a complex set of post-translational modifications of histones, also called histone code, and nucleosome remodeling.
HIST2H2AA3	Histone H2A type 2-A		10.90	9	26	0.3	94.65	-0.487	**DNA binding protein**	**Regulation of nucleobase, nucleoside, nucleotide and nucleic acid metabolism**	**Nucleus; Nucleolus**	**Core component of nucleosome. Nucleosomes wrap and compact DNA into chromatin, limiting DNA accessibility to the cellular machineries which require DNA as a template. Histones thereby play a central role in transcription regulation, DNA repair, DNA replication and chromosomal stability. DNA accessibility is regulated via a complex set of post-translational modifications of histones, also called histone code, and nucleosome remodeling.**

H2AFJ	Histone H2A.J		10.90	9	26	0.3	99.14	-0.457	DNA binding protein	Regulation of nucleobase, nucleoside, nucleotide and nucleic acid metabolism	Nucleus	Core component of nucleosome. Nucleosomes wrap and compact DNA into chromatin, limiting DNA accessibility to the cellular machineries which require DNA as a template. Histones thereby play a central role in transcription regulation, DNA repair, DNA replication and chromosomal stability. DNA accessibility is regulated via a complex set of post-translational modifications of histones, also called histone code, and nucleosome remodeling.
**HIST1H2BA**	**Histone H2B type 1-A**	**[5-6, 12]**	10.32	10	28	0.4	75.08	-0.606	DNA binding protein	Regulation of nucleobase, nucleoside, nucleotide and nucleic acid metabolism	Nucleus	Variant histone specifically required to direct the transformation of dissociating nucleosomes to protamine in male germ cells. Entirely replaces classical histone H2B prior nucleosome to protamine transition and probably acts as a nucleosome dissociating factor that creates a more dynamic chromatin, facilitating the large-scale exchange of histones. Also expressed maternally and is present in the female pronucleus, suggesting a similar role in protamine replacement by nucleosomes at fertilization By similarity. Also found in fat cells, its function and the presence of post-translational modifications specific to such cells are still unclear.
**HIST1H2BB**	**Histone H2B type 1-B**	**[1]**	10.32	10	28	0.4	69.52	-0.775	**DNA binding protein**	**Regulation of nucleobase, nucleoside, nucleotide and nucleic acid metabolism**	**Nucleus**	**Core component of nucleosome. Nucleosomes wrap and compact DNA into chromatin, limiting DNA accessibility to the cellular machineries which require DNA as a template. Histones thereby play a central role in transcription regulation, DNA repair, DNA replication and chromosomal stability. DNA accessibility is regulated via a complex set of post-translational modifications of histones, also called histone code, and nucleosome remodeling.**
H2BFS	Histone H2B type F-S		10.37	10	28	0.4	68.72	-0.770	DNA binding protein	Regulation of nucleobase, nucleoside, nucleotide and nucleic acid metabolism	Nucleus	Has broad antibacterial activity. May contribute to the formation of the functional antimicrobial barrier of the colonic epithelium, and to the bactericidal activity of amniotic fluid.
**H3F3C**	**Histone H3.3C**	**[1]**	11.11	11	29	0.4	83.88	-0.601	**DNA binding protein**	**Regulation of nucleobase, nucleoside, nucleotide and nucleic acid metabolism**	**Nucleus**	**Core component of nucleosome. Nucleosomes wrap and compact DNA into chromatin, limiting DNA accessibility to the cellular machineries which require DNA as a template. Histones thereby play a central role in transcription regulation, DNA repair, DNA replication and chromosomal stability.**
**HIST1H4A**	**Histone H4**	**[1, 3-4, 7, 9, 11-12]**	11.36	7	25	0.3	85.98	-0.545	**DNA binding protein**	**Regulation of nucleobase, nucleoside, nucleotide and nucleic acid metabolism**	**Nucleus**	**Core component of nucleosome. Nucleosomes wrap and compact DNA into chromatin, limiting DNA accessibility to the cellular machineries which require DNA as a template. Histones thereby play a central role in transcription regulation, DNA repair, DNA replication and chromosomal stability. DNA accessibility is regulated via a complex set of post-translational modifications of histones, also called histone code, and nucleosome remodeling.**
HLA-A	HLA class I histocompatibility antigen, A-23 alpha chain		5.80	46	38	1.2	65.84	-0.621	MHC complex protein	Immune response	Plasma membrane; Endoplasmic reticulum; Golgi apparatus; Extracellular; Nucleus	Involved in the presentation of foreign antigens to the immune system.
HLA-A	HLA class I histocompatibility antigen, A-24 alpha chain		5.80	46	38	1.2	64.69	-0.635	MHC complex protein	Immune response	Plasma membrane; Endoplasmic reticulum; Golgi apparatus; Extracellular; Nucleus	Involved in the presentation of foreign antigens to the immune system.
CD74	HLA class II histocompatibility antigen gamma chain		8.72	29	34	0.9	70.47	-0.600	MHC complex protein	Immune response	Endoplasmic reticulum; Golgi apparatus; Endosome; Plasma membrane	Plays a critical role in MHC class II antigen processing by stabilizing peptide-free class II alpha/beta heterodimers in a complex soon after their synthesis and directing transport of the complex from the endoplasmic reticulum to the endosomal/lysosomal system where the antigen processing and binding of antigenic peptides to MHC class II takes place. Serves as cell surface receptor for the cytokine MIF.
HOXB3	Homeobox protein Hox-B3		9.27	23	35	0.7	42.74	-0.754	**Transcription factor**	**Regulation of nucleobase, nucleoside, nucleotide and nucleic acid metabolism**	**Nucleus**	**Sequence-specific transcription factor which is part of a developmental regulatory system that provides cells with specific positional identities on the anterior-posterior axis.**
SIX3	Homeobox protein SIX3		8.95	33	38	0.9	64.16	-0.563	**Transcription factor**	**Regulation of nucleobase, nucleoside, nucleotide and nucleic acid metabolism**	**Nucleus**	**Transcriptional repressor essential for eye and forebrain development. Involved in the development of the forebrain by negatively regulating the expression of WNT1. Plays an important role during retina development and lens morphogenesis in an AES and TLE4-dependent manner By similarity.**
**HRNR**	**Hornerin (S100A18)**	**[10-11]**	10.05	99	183	0.5	8.83	-1.364	Unclassified	Unknown	Plasma membrane	Component of the epidermal cornified cell envelopes.
**ST13**	**Hsc70-interacting protein**	**[3]**	5.18	70	56	1.3	64.36	-0.857	Adapter molecule	Cell communication; Signal transduction	Lysosome; Cytoplasm	One HIP oligomer binds the ATPase domains of at least two HSC70 molecules dependent on activation of the HSC70 ATPase by HSP40. Stabilizes the ADP state of HSC70 that has a high affinity for substrate protein. Through its own chaperone activity, it may contribute to the interaction of HSC70 with various target proteins By
**CDC37**	**Hsp90 co-chaperone Cdc37**	**[1]**	5.17	84	66	1.3	66.18	-0.975	Chaperone	Protein metabolism	Cytoplasm	Co-chaperone that binds to numerous kinases and promotes their interaction with the Hsp90 complex, resulting in stabilization and promotion of their activity.
HABP2	Hyaluronan-binding protein 2		6.10	67	57	1.2	60.52	-0.549	Serine protease	Protein metabolism	Extracellular	Cleaves the alpha-chain at multiple sites and the beta-chain between 'Lys-53' and 'Lys-54' but not the gamma-chain of fibrinogen and therefore does not initiate the formation of the fibrin clot and does not cause the fibrinolysis directly. It does not cleave (activate) prothrombin and plasminogen but converts the inactive single chain urinary plasminogen activator (pro-urokinase) to the active two chain form. Activates coagulation factor VII.
**HYAL1**	**Hyaluronidase-1**	**[2]**	6.48	34	32	1.1	76.64	-0.204	**Enzyme: Hydrolase**	**Metabolism**	**Extracellular**	**May have a role in promoting tumor progression. May block the TGFB1-enhanced cell growth.**
HMGCS2	Hydroxymethylglutaryl-CoA synthase, mitochondrial		6.64	52	51	1.0	75.41	-0.337	Enzyme: Synthase	Metabolism; Energy pathways	Mitochondrion	This enzyme condenses acetyl-CoA with acetoacetyl-CoA to form HMG-CoA, which is the substrate for HMG-CoA reductase.
HYOU1	Hypoxia up-regulated protein 1		5.07	163	127	1.3	79.48	-0.569	Chaperone	Protein metabolism	Endoplasmic reticulum; Cytoplasm; Mitochondrion	Has a pivotal role in cytoprotective cellular mechanisms triggered by oxygen deprivation. May play a role as a molecular chaperone and participate in protein folding.
**IGHA1**	**Ig alpha-1 chain C region**	**[1-2, 5, 7-8, 11]**	6.08	29	25	1.2	72.82	-0.203	**Immunoglobulin**	**Immune response**	**Extracellular**	**Ig alpha is the major immunoglobulin class in body secretions. It may serve both to defend against local infection and to prevent access of foreign antigens to the general immunologic system.**
**IGHG1**	**Ig gamma-1 chain C region**	**[1-2, 4-8, 10, 12]**	8.46	30	34	0.9	71.33	-0.428	Immunoglobulin	Immune response	Extracellular	Classical antibody-mediated complement activation. Regulation of actin dynamics for phagocytic cup formation.
**IGHG2**	**Ig gamma-2 chain C region**	**[7, 8]**	7.51	31	32	1.0	66.09	-0.426	Immunoglobulin	Immune response	Extracellular	Classical antibody-mediated complement activation.
**IGHG3**	**Ig gamma-3 chain C region**	**[5, 7]**	8.23	36	40	0.9	61.67	-0.511	**Immunoglobulin**	**Immune response**	**Extracellular**	**Fc-gamma receptor signaling pathway involved in phagocytosis**
**IGHG4**	**Ig gamma-4 chain C region**	**[5, 7]**	7.18	32	32	1.0	68.99	-0.423	Immunoglobulin	Immune response	Extracellular	Fc-gamma receptor signaling pathway involved in phagocytosis
	Ig heavy chain V-I region HG3		9.23	6	10	0.6	57.65	-0.410				
	Ig heavy chain V-II region ARH-77		8.58	8	10	0.8	76.69	-0.192				
	**Ig heavy chain V-III region BRO**	**[1]**	6.44	9	9	1.0	75.50	-0.211				
**IGKC**	**Ig kappa chain C region**	**[1, 4-6, 8]**	5.58	11	9	1.2	67.55	-0.553	Immunoglobulin	Immune response	Extracellular	Fc-epsilon receptor signaling pathway
**IGKV**	**Ig kappa chain V-I region AG***	**[2, 5, 8, 11]**	5.67	10	9	1.1	66.85	-0.505	**Immunoglobulin**	**Immune response**	**Extracellular**	**Immunoglobulins (Ig) are the antigen recognition molecules of B cells. An Ig molecule is made up of 2 identical heavy chains and 2 identical light chains, either kappa or lambda, joined by disulfide bonds so that each heavy chain is linked to a light chain and the 2 heavy chains are linked together. The kappa and lambda light chains have no apparent functional differences. Each Ig kappa light chain has an N-terminal variable (V) region containing the antigen-binding site and a C-terminal constant (C) region, encoded by a C region gene (IGKC), that provides signaling functions. The kappa light chain V region is encoded by 2 types of genes: V genes and joining (J) genes. Random selection of just 1 gene of each type to assemble a V region accounts for the great diversity of V regions among Ig molecules.**
**IGKV**	**Ig kappa chain V-I region Lay**	**[11]**	7.96	7	8	0.9	64.07	-0.452				
	Ig kappa chain V-II region Cum		8.05	9	10	0.9	75.61	-0.422				
	Ig kappa chain V-II region MIL		9.39	4	8	0.5	84.38	-0.254				
	Ig kappa chain V-II region RPMI 6410		9.07	8	12	0.7	65.40	-0.497				
	Ig kappa chain V-III region GOL		9.34	8	12	0.7	68.99	-0.386				
**IGKV**	**Ig kappa chain V-III region HAH**	**[2, 11]**	9.07	8	11	0.7	67.16	-0.417	**Immunoglobulin**	**Immune response**	**Extracellular**	**Classical antibody-mediated complement activation.**
**IGKV**	**Ig kappa chain V-IV region B17**	**[2]**	6.37	10	10	1.0	71.84	-0.464	**Immunoglobulin**	**Immune response**	**Extracellular**	**Classical antibody-mediated complement activation.**
	**Ig lambda chain V-I region HA**	**[1]**	9.07	7	10	0.7	68.75	-0.410	**Immunoglobulin**	**Immune response**	**Extracellular**	**Classical antibody-mediated complement activation.**
	Ig lambda chain V-III region LOI		4.57	12	6	2.0	69.28	-0.448	**Immunoglobulin**	**Immune response**	**Extracellular**	**Classical antibody-mediated complement activation.**
**IGLC1**	**Ig lambda-1 chain C regions**	**[4-8, 10]**	8.29	8	9	0.9	65.68	-0.420	**Immunoglobulin**	**Immune response**	**Extracellular**	**Classical antibody-mediated complement activation.**
**IGLC2**	**Ig lambda-2 chain C regions**	**[1, 5-8, 10]**	7.22	8	8	1.0	65.68	-0.392	Immunoglobulin	Immune response	Extracellular	Classical antibody-mediated complement activation.
**IGHM**	**Ig mu chain C region**	**[1]**	6.35	44	41	1.1	71.77	-0.326	**Immunoglobulin**	**Immune response**	**Extracellular**	**Classical antibody-mediated complement activation.**
**IGJ**	**Immunoglobulin J chain**	**[1]**	4.59	25	16	1.6	81.75	-0.558	Immunoglobulin	Immune response	Endoplasmic reticulum; Golgi apparatus; Nucleus; Extracellular	Serves to link two monomer units of either IgM or IgA. In the case of IgM, the J chain-joined dimer is a nucleating unit for the IgM pentamer, and in the case of IgA it induces larger polymers. It also help to bind these immunoglobulins to secretory component.
IGLL5	Immunoglobulin lambda-like polypeptide 5		9.03	13	18	0.7	64.80	-0.449	Immunoglobulin	Immune response	Extracellular	Classical antibody-mediated complement activation.
IGSF8	Immunoglobulin superfamily member 8		8.04	57	59	1.0	86.21	-0.139	Immunoglobulin	Immune response	Plasma membrane	May play a key role in diverse functions ascribed to CD81 and CD9 such as oocytes fertilization or hepatitis C virus function. May regulate proliferation and differentiation of keratinocytes. May be a negative regulator of cell motility: suppresses T-cell mobility coordinately with CD81, associates with CD82 to suppress
KPNB1	Importin subunit beta-1		4.68	124	75	1.7	98.89	-0.092	Transport/cargo protein	Transport	Cytoplasm; Nucleus; Extracellular; Mitochondrion; Centrosome	Functions in nuclear protein import, either in association with an adapter protein, like an importin-alpha subunit, which binds to nuclear localization signals (NLS) in cargo substrates, or by acting as autonomous nuclear transport receptor. Acting autonomously, serves itself as NLS receptor. Docking of the importin/substrate complex to the nuclear pore complex (NPC) is mediated by KPNB1 through binding to nucleoporin FxFG repeats and the complex is subsequently translocated through the pore by an energy requiring, Ran-dependent mechanism. At the nucleoplasmic side of the NPC, Ran binds to importin-beta and the three components separate and importin-alpha and -beta are re-exported from the nucleus to the cytoplasm where GTP hydrolysis releases Ran from importin.
**RHBDF1**	**Inactive rhomboid protein 1**	**[1]**	8.80	93	108	0.9	80.19	-0.268	**Integral membrane protein**	**Unknown**	**Endoplasmic reticulum; Golgi apparatus**	**Rhomboid protease-like protein which has no protease activity but regulates the secretion of several ligands of the epidermal growth factor receptor. Indirectly activates the epidermal growth factor receptor signaling pathway and may thereby regulate sleep, cell survival, proliferation and migration.**
IMPDH2	Inosine-5'-monophosphate dehydrogenase 2		6.46	62	59	1.1	92.22	-0.118	Enzyme: Dehydrogenase	Metabolism; Energy pathways	Cytosol; Cytoplasm	Catalyzes the conversion of inosine 5'-phosphate (IMP) to xanthosine 5'-phosphate (XMP), the first committed and rate-limiting step in the de novo synthesis of guanine nucleotides, and therefore plays an important role in the regulation of cell growth. Could also have a single-stranded nucleic acid-binding activity and could play a role in RNA and/or DNA metabolism. It may also have a role in the development of malignancy and the growth progression of some tumors.
**IGF1**	**Insulin-like growth factor I**	**[5, 8]**	7.76	8	9	0.9	60.00	-0.274	**Growth factor**	**Cell communication; Signal transduction; Anti-apoptosis; Lipid metabolism; Wound healing; Cell proliferation; Cell motility**	**Cytoplasm; Extracellular**	**The insulin-like growth factors, isolated from plasma, are structurally and functionally related to insulin but have a much higher growth-promoting activity. May be a physiological regulator of [1-14C]-2-deoxy-D-glucose (2DG) transport and glycogen synthesis in osteoblasts. Stimulates glucose transport in rat bone-derived osteoblastic (PyMS) cells and is effective at much lower concentrations than insulin, not only regarding glycogen and DNA synthesis but also with regard to enhancing glucose uptake.**
**IGFALS**	**Insulin-like growth factor-binding protein complex acid labile subunit**	**[2-3]**	6.13	58	51	1.1	109.60	-0.024	**Growth factor binding**	**Cell communication; Signal transduction**	**Extracellular**	**Involved in protein-protein interactions that result in protein complexes, receptor-ligand binding or cell adhesion.**
ITM2B	Integral membrane protein 2B		5.00	40	28	1.4	98.27	-0.067	**Integral membrane protein**	**Cell growth and/or maintenance**	**Plasma membrane**	**Plays a regulatory role in the processing of the beta-amyloid A4 precursor protein (APP) and acts as an inhibitor of the beta-amyloid peptide aggregation and fibrils deposition. Plays a role in the induction of neurite outgrowth. Functions as a protease inhibitor by blocking access of secretases to APP**
ITGAM	Integrin alpha-M		6.75	112	109	1.0	84.48	-0.212	Cell surface receptor	Cell communication; Signal transduction	Plasma membrane	Integrin alpha-M/beta-2 is implicated in various adhesive interactions of monocytes, macrophages and granulocytes as well as in mediating the uptake of complement-coated particles. It is identical with CR-3, the receptor for the iC3b fragment of the third complement component. It probably recognizes the R-G-D peptide in C3b. Integrin alpha-M/beta-2 is also a receptor for fibrinogen, factor X and ICAM1. It recognizes P1 and P2 peptides of fibrinogen gamma chain.
ITGB2	Integrin beta		6.54	88	85	1.0	72.68	-0.377	Adhesion molecule	Cell communication; Signal transduction	Plasma membrane; Mitochondrion	Integrin alpha-L/beta-2 is a receptor for ICAM1, ICAM2, ICAM3 and ICAM4. Integrins alpha-M/beta-2 and alpha-X/beta-2 are receptors for the iC3b fragment of the third complement component and for fibrinogen.

ILK	Integrin-linked protein kinase		8.30	54	57	0.9	80.91	-0.412	Serine/threonine kinase	Cell communication; Signal transduction	Cytoplasm; Nucleus; Microsome	Receptor-proximal protein kinase regulating integrin-mediated signal transduction. May act as a mediator of inside-out integrin signaling. Focal adhesion protein part of the complex ILK-PINCH. This complex is considered to be one of the convergence points of integrin- and growth factor-signaling pathway. Could be implicated in mediating cell architecture, adhesion to integrin substrates and anchorage-dependent growth in epithelial cells. Phosphorylates beta-1 and beta-3 integrin subunit on serine and threonine residues, but also AKT1 and GSK3B.
**ITIH1**	**Inter-alpha-trypsin inhibitor heavy chain H1**	[1, 11]	6.33	77	71	1.1	85.71	-0.356	Protease inhibitor	Protein metabolism	Extracellular	May act as a carrier of hyaluronan in serum or as a binding protein between hyaluronan and other matrix protein, including those on cell surfaces in tissues to regulate the localization, synthesis and degradation of hyaluronan which are essential to cells undergoing biological processes. Contains a potential peptide which could stimulate a broad spectrum of phagocytotic cells.
**ITIH2**	**Inter-alpha-trypsin inhibitor heavy chain H2**	[1]	5.75	78	66	1.2	88.12	-0.305	Protease inhibitor	Protein metabolism	Extracellular	May act as a carrier of hyaluronan in serum or as a binding protein between hyaluronan and other matrix protein, including those on cell surfaces in tissues to regulate the localization, synthesis and degradation of hyaluronan which are essential to cells undergoing biological processes.
**ITIH4**	**Inter-alpha-trypsin inhibitor heavy chain H4**	[2, 11]	6.11	101	93	1.1	83.34	-0.338	Protease inhibitor	Protein metabolism	Extracellular	Type II acute-phase protein (APP) involved in inflammatory responses to trauma. May also play a role in liver development or regeneration.
IDH2	Isocitrate dehydrogenase [NADP]		8.32	52	55	0.9	78.62	-0.388	Enzyme: Dehydrogenase	Metabolism; Energy pathways	Mitochondrion	Plays a role in intermediary metabolism and energy production. It may tightly associate or interact with the pyruvate dehydrogenase complex.
IARS	Isoleucine--tRNA ligase, cytoplasmic		5.82	165	147	1.1	91.55	-0.238	Enzyme: Ligase	Protein metabolism	Cytoplasm; Mitochondrion; Ribosome	ATP + L-isoleucine + tRNA(Ile) = AMP + diphosphate + L-isoleucyl-tRNA(Ile).
ISM2	Isthmin-2		4.66	94	54	1.7	63.78	-0.839	Secreted polypeptide	Cell adhesion	Extracellular	A type 1 thrombospondin domain, which is present in thrombospondin, a number of proteins involved in the complement pathway, as well as in extracellular matrix proteins. Two alternatively spliced transcript variants encoding distinct isoforms have been observed.
JMJD8	JmjC domain-containing protein 8		9.74	28	39	0.7	69.88	-0.355	Unclassified	Unknown	Extracellular vesicular exosome	??
**JUP**	**Junction plakoglobin**	[10]	5.75	81	67	1.2	97.05	-0.157	Adhesion molecule	Cell communication; Signal transduction	Cytoplasm; Plasma membrane; Extracellular; Cell junction;	Common junctional plaque protein. The membrane-associated plaques are architectural elements in an important strategic position to influence the arrangement and function of both the cytoskeleton and the cells within the tissue. The presence of plakoglobin in both the desmosomes and in the intermediate junctions suggests that it
LTF	Kaliocin-1 (Lactotransferrin)		8.47	79	89	0.9	71.03	-0.415	Transport/cargo protein	Transport	Secretory granule; Golgi apparatus; Endoplasmic reticulum; Nucleolus; Plasma membrane; Cytoplasm; Extracellular; Nucleus	Transferrins are iron binding transport proteins which can bind two Fe3+ ions in association with the binding of an anion, usually bicarbonate. Lactotransferrin is a major iron-binding and multifunctional protein found in exocrine fluids such as breast milk and mucosal secretions. Has antimicrobial activity, which depends on the extracellular cation concentration. Antimicrobial properties include bacteriostasis, which is related to its ability to sequester free iron and thus inhibit microbial growth, as well as direct bactericidal properties leading to the release of lipopolysaccharides from the bacterial outer membrane. Can also prevent bacterial biofilm development
**KLK7**	**Kallikrein-7**	[10]	9.03	17	26	0.7	73.04	-0.332	Serine protease	Protein metabolism; Proteolysis and peptidolysis	Extracellular; Cytoplasm; Apical membrane	May catalyze the degradation of intercellular cohesive structures in the cornified layer of the skin in the continuous shedding of cells from the skin surface.
SERPINA4	Kallistatin		7.88	43	44	1.0	90.32	-0.155	Protease inhibitor	Protein metabolism	Extracellular	Inhibits human amidolytic and kininogenase activities of tissue kallikrein. Inhibition is achieved by formation of an equimolar, heat- and SDS-stable complex between the inhibitor and the enzyme, and generation of a small C-terminal fragment of the inhibitor due to cleavage at the reactive site by tissue kallikrein.
KRT31	Keratin, type I cuticular Ha1		4.84	60	42	1.4	83.20	-0.547	Structural protein	Cell growth and/or maintenance	Cytoskeleton	Structural constituent of the cytoskeleton, intermediate filament
**KRT10**	**Keratin, type I cytoskeletal 10**	[1]	5.13	63	47	1.3	55.46	-0.624	Structural protein	Cell growth and/or maintenance	Cytoplasm; Nucleolus; Cytoskeleton	Structural constituent of the cytoskeleton, intermediate filament
**KRT13**	**Keratin, type I cytoskeletal 13**	[1, 3]	4.91	65	48	1.4	75.22	-0.475	Structural protein	Cell growth and/or maintenance	Cytoplasm; Plasma membrane; Nucleus	Structural constituent of the cytoskeleton, intermediate filament
**KRT14**	**Keratin, type I cytoskeletal 14**	[1, 3]	5.09	69	55	1.3	75.64	-0.537	Structural protein	Cell growth and/or maintenance	Cytoplasm	The nonhelical tail domain is involved in promoting KRT5-KRT14 filaments to self-organize into large bundles and enhances the mechanical properties involved in resilience of keratin intermediate filaments in vitro.
**KRT15**	**Keratin, type I cytoskeletal 15**	[1]	4.71	70	45	1.6	75.99	-0.446	Structural protein	Cell growth and/or maintenance	Cytoplasm	Structural constituent of the cytoskeleton, intermediate filament
KRT16	Keratin, type I cytoskeletal 16		4.98	65	50	1.3	75.10	-0.517	Structural protein	Cell growth and/or maintenance	Cytoplasm	Structural constituent of the cytoskeleton, intermediate filament
**KRT17**	**Keratin, type I cytoskeletal 17**	[1]	4.97	68	52	1.3	81.99	-0.611	Structural protein	Cell growth and/or maintenance	Cytoplasm; Nucleolus; Extracellular; Nucleus	May be a marker of basal cell differentiation in complex epithelia and therefore indicative of a certain type of epithelial "stem cells". May act as an autoantigen in the immunopathogenesis of psoriasis, with certain peptide regions being a major target for autoreactive T-cells and hence causing their proliferation.Regulates protein synthesis and epithelial cell growth through binding to the adapter protein SFN and by stimulating Akt/mTOR pathway.
**KRT18**	**Keratin, type I cytoskeletal 18**	[1, 3]	5.34	67	56	1.2	86.90	-0.561	Structural protein	Cell growth and/or maintenance	Cytoplasm; Nucleus; Nucleolus; Extracellular; Cytoskeleton	When phosphorylated, plays a role in filament reorganization. Involved in the delivery of mutated CFTR to the plasma membrane. Involved in the uptake of thrombin-antithrombin complexes by hepatic cells By similarity. Together with KRT8, is involved in interleukin-6 (IL-6)-mediated barrier protection.
**KRT19**	**Keratin, type I cytoskeletal 19**	[1]	5.05	60	45	1.3	82.97	-0.532	Structural protein	Cell growth and/or maintenance	Cytoplasm	Involved in the organization of myofibers. Together with KRT8, helps to link the contractile apparatus to dystrophin at the costameres of striated muscle.
**KRT9**	**Keratin, type I cytoskeletal 9**	[1, 3]	5.14	66	50	1.3	49.00	-0.701	Structural protein	Cell growth and/or maintenance	Cytoplasm; Cytoskeleton	Plays a role in keratin filament assembly.
KRT81	Keratin, type II cuticular Hb1		5.40	68	59	1.2	80.73	-0.268	Structural protein	Cell growth and/or maintenance	Cytoplasm	Structural constituent of the cytoskeleton, intermediate filament
KRT84	Keratin, type II cuticular Hb4		7.74	67	69	1.0	79.98	-0.268	Structural protein	Cell growth and/or maintenance	Cytoplasm	Structural constituent of the cytoskeleton, intermediate filament
**KRT85**	**Keratin, type II cuticular Hb5**	[1]	6.27	68	66	1.0	77.55	-0.373	Structural protein	Cell growth and/or maintenance	Cytoplasm	Structural constituent of the cytoskeleton, intermediate filament
**KRT1**	**Keratin, type II cytoskeletal 1**	[1, 3]	8.15	64	66	1.0	59.13	-0.630	Structural protein	Cell growth and/or maintenance	Plasma membrane; Cytoskeleton	May regulate the activity of kinases such as PKC and SRC via binding to integrin beta-1 (ITB1) and the receptor of activated protein kinase C (RACK1/GNB2L1). activated protein kinase C (RACK1/GNB2L1). In complex with C1QBP is a high affinity receptor for kininogen-1/HMWK By similarity.
**KRT77**	**Keratin, type II cytoskeletal 1b**	[1]	5.72	65	58	1.1	73.01	-0.518	Structural protein	Cell growth and/or maintenance	Cytoplasm	Structural constituent of the cytoskeleton, intermediate filament
**KRT2**	**Keratin, type II cytoskeletal 2 epidermal**	[1]	8.07	65	67	1.0	65.90	-0.471	Structural protein	Cell growth and/or maintenance	Cytoplasm; Cytoskeleton	Probably contributes to terminal cornification. Associated with keratinocyte activation, proliferation and keratinization.
KRT3	Keratin, type II cytoskeletal 3		6.12	63	60	1.1	66.08	-0.389	Structural protein	Cell growth and/or maintenance	Cytoplasm	Structural constituent of the cytoskeleton, intermediate filament
**KRT4**	**Keratin, type II cytoskeletal 4**	[1]	6.25	63	61	1.0	77.43	-0.389	Cytoskeletal protein	Cell growth and/or maintenance	Cytoplasm	The type II cytokeratins consist of basic or neutral proteins which are arranged in pairs of heterotypic keratin chains coexpressed during differentiation of simple and stratified epithelial tissues. This type II cytokeratin is specifically expressed in differentiated layers of the mucosal and esophageal epithelia with family member KRT13.
KRT5	Keratin, type II cytoskeletal 5		7.58	65	66	1.0	70.41	-0.428	Structural protein	Cell growth and/or maintenance	Cytoplasm; Nucleus	Structural constituent of the cytoskeleton, intermediate filament
**KRT6A**	**Keratin, type II cytoskeletal 6A**	[1]	8.14	64	66	1.0	77.64	-0.443	Structural protein	Cell growth and/or maintenance	Cytoplasm	Structural constituent of the cytoskeleton, intermediate filament
**KRT6B**	**Keratin, type II cytoskeletal 6B**	[1]	8.14	64	66	1.0	78.17	-0.455	Structural protein	Cell growth and/or maintenance	Cytoplasm	Structural constituent of the cytoskeleton, intermediate filament
KRT6C	Keratin, type II cytoskeletal 6C		8.14	64	66	1.0	78.86	-0.436	Cytoskeletal protein	Cell growth and/or maintenance	Cytoskeleton	Structural constituent of the cytoskeleton, intermediate filament
**KRT7**	**Keratin, type II cytoskeletal 7**	[1, 3]	5.39	69	61	1.1	86.41	-0.477	Structural protein	Cell growth and/or maintenance	Cytoplasm; Plasma membrane	Blocks interferon-dependent interphase and stimulates DNA synthesis in cells. Involved in the translational regulation of the human papillomavirus type 16 E7 mRNA (HPV16 E7).
**KRT73**	**Keratin, type II cytoskeletal 73 (likely contaminant)**	[1]	6.93	69	69	1.0	80.26	-0.425	Structural protein	Cell growth and/or maintenance	Extracellular vesicular exosome	Has a role in hair formation. Specific component of keratin intermediate filaments in the inner root sheath (IRS) of the hair follicle (Probable).
**KRT75**	**Keratin, type II cytoskeletal 75 (likely contaminant)**	[1]	7.60	65	66	1.0	77.01	-0.452	Structural protein	Cell growth and/or maintenance	Extracellular vesicular exosome	Plays a central role in hair and nail formation. Essential component of keratin intermediate filaments in the companion layer of the hair follicle.
**KRT79**	**Keratin, type II cytoskeletal 79 (likely contaminant)**	[1]	6.75	62	61	1.0	77.12	-0.484	Structural protein	Cell growth and/or maintenance	Cytoskeleton	Epithelial keratin that is expressed in skeletal muscle, skin and scalp.
**KRT8**	**Keratin, type II cytoskeletal 8**	[3]	5.52	70	64	1.1	79.79	-0.597	Structural protein	Cell growth and/or maintenance	Cytoplasm; Nucleolus; Extracellular; Cytoskeleton; Nucleus	Together with KRT19, helps to link the contractile apparatus to dystrophin at the costameres of striated muscle.
**KRT80**	**Keratin, type II cytoskeletal 80**	[1]	5.57	70	63	1.1	84.16	-0.504	Cytoskeletal protein	Cell growth and/or maintenance	Cytoskeleton	Structural constituent of the cytoskeleton, intermediate filament
KHK	Ketohexokinase, Isoform C		5.64	38	33	1.2	91.54	-0.096	Enzyme: Phosphotransferase	Metabolism; Energy pathways	Cytoplasm	ATP + D-fructose = ADP + D-fructose 1-phosphate.
KIF5B	Kinesin-1 heavy chain		6.12	162	151	1.1	79.38	-0.813	Motor protein	Cell growth and/or maintenance	Mitochondrion; Microtubule	Microtubule-dependent motor required for normal distribution of mitochondria and lysosomes.
**KIF13B**	**Kinesin-like protein KIF13B**	[1]	5.64	264	226	1.2	79.67	-0.556	Motor protein	Cell growth and/or maintenance	Cytoplasm	Involved in reorganization of the cortical cytoskeleton. Regulates axon formation by promoting the formation of extra axons. May be functionally important for the intracellular trafficking of MAGUKs and associated protein complexes.
**KIF1A**	**Kinesin-like protein KIF1A**	[1]	5.86	238	209	1.1	79.34	-0.503	Motor protein	Transport	Cytoplasm	Motor for anterograde axonal transport of synaptic vesicle precursors.
KIF23	Kinesin-like protein KIF23		8.76	134	147	0.9	70.64	-0.820	Motor protein	Cell growth and/or maintenance	Nucleus; Cytoplasm; Microtubule; Chromosome	Component of the centralspindlin complex that serves as a microtubule-dependent and Rho-mediated signaling required for the myosin contractile ring formation during the cell cycle cytokinesis. Essential for cytokinesis in Rho-mediated signaling. Required for the localization of ECT2 to the central spindle. Plus-end-directed motor enzyme that moves antiparallel microtubules in vitro.
**KIF3A**	**Kinesin-like protein KIF3A**	[1]	6.16	126	119	1.1	77.84	-0.852	Motor protein	Cell growth and/or maintenance	Cytoplasm	Microtubule-based anterograde translocator for membranous organelles. Plus end-directed microtubule sliding activity in vitro. Plays a role in primary cilia formation (By similarity).
**KNG1**	**Kininogen-1**	[1-3, 5, 8, 11]	6.23	82	70	1.2	57.94	-0.829	Coagulation factor	Protein metabolism	Extracellular	Kininogens are inhibitors of thiol proteases.
**LTF**	**Lactotransferrin**	[1-10]	8.47	79	89	0.9	71.03	-0.415	Transport/cargo protein	Transport	Secretory granule; Golgi apparatus; Endoplasmic reticulum; Nucleolus; Plasma membrane; Cytoplasm; Extracellular; Nucleus	Lactotransferrin is a major iron-binding and multifunctional protein found in exocrine fluids such as breast milk and mucosal secretions. Has antimicrobial activity, which depends on the extracellular cation concentration. Antimicrobial properties include bacteriostasis, which is related to its ability to sequester free iron and thus inhibit microbial growth, as well as direct bactericidal properties leading to the release of lipopolysaccharides from the bacterial outer membrane. Can also prevent bacterial biofilm development in P.aeruginosa infection.
CRYL1	Lambda-crystallin homolog		5.81	38	32	1.2	97.48	-0.047	Enzyme: Oxidoreductase	Metabolism; Energy pathways	Cytoplasm	The uronate cycle functions as an alternative glucose metabolic pathway, accounting for about 5% of daily glucose catabolism. The product of this gene catalyzes the dehydrogenation of L-gulonate into dehydro-L-gulonate in the uronate cycle. The enzyme requires NAD(H) as a coenzyme, and is inhibited by inorganic phosphate.
LAMA3	Laminin subunit alpha-3		6.88	357	347	1.0	74.53	-0.394	Extracellular matrix protein	Cell growth and/or maintenance	Extracellular	Binding to cells via a high affinity receptor, laminin is thought to mediate the attachment, migration and organization of cells into tissues during embryonic development by interacting with other extracellular matrix components. Laminin-5 is thought to be involved in (1) cell adhesion via integrin alpha-3/beta-1 in focal adhesion and
LAMC1	Laminin subunit gamma-1		4.94	241	172	1.4	62.26	-0.637	Extracellular matrix protein	Cell growth and/or maintenance	Extracellular; Plasma membrane	Binding to cells via a high affinity receptor, laminin is thought to mediate the attachment, migration and organization of cells into tissues during embryonic development by interacting with other extracellular matrix components.
**L3MBTL2**	**Lethal(3)malignant brain tumor-like protein 2**	[3]	6.40	97	91	1.1	73.13	-0.453	Transcription regulatory protein	Regulation of nucleobase, nucleoside, nucleotide and nucleic acid metabolism	Nucleus; Cytoplasm	Putative Polycomb group (PcG) protein. PcG proteins maintain the transcriptionally repressive state of genes, probably via a modification of chromatin, rendering it heritably changed in its expressibility. Its association with a chromatin-remodeling complex suggests that it may contribute to prevent expression of genes that trigger the cell into mitosis.

SHOC2	Leucine-rich repeat protein SHOC-2	[1]	8.65	67	73	0.9	105.72	-0.335	Adapter molecule	Cell communication; Signal transduction	Cytoplasm; Chromosome	Regulatory subunit of protein phosphatase 1 (PP1c) that acts as a M-Ras/MRAS effector and participates in MAPK pathway activation.
LRRC16B	Leucine-rich repeat-containing protein 16B		7.20	159	158	1.0	79.29	-0.517	Unclassified	Unknown	Cytoplasm	Binds CAPZA2 with high affinity and significantly decreases CAPZA2 affinity for actin barbed ends. Increases the rate of elongation from seeds in the presence of CAPZA2, however, seems unable to nucleate filaments. Rapidly uncaps barbed ends capped by CAPZA2 and enhances barbed-end actin polymerization By similarity. May control actin dynamics in lamellipodia. Required for cell migration.
LRRC47	Leucine-rich repeat-containing protein 47		8.56	77	82	0.9	96.87	-0.400	Unclassified	Unknown	Cytoplasm	??
LARS	Leucine--tRNA ligase, cytoplasmic		6.95	161	159	1.0	81.73	-0.403	Enzyme: Ligase	Metabolism; Energy pathways	Mitochondrion; Cytosol	Catalyzes the specific attachment of an amino acid to its cognate tRNA in a two step reaction: the amino acid (AA) is first activated by ATP to form AA-AMP and then transferred to the acceptor end of the tRNA. Exhibits a post-transfer editing activity to hydrolyze mischarged tRNAs.
SERPINB1	Leukocyte elastase inhibitor	[1]	5.90	48	43	1.1	83.43	-0.249	Protease inhibitor	Protein metabolism	Cytoplasm	Regulates the activity of the neutrophil proteases elastase, cathepsin G, proteinase-3, chymase, chymotrypsin, and kallikrein-3. Also functions as a potent intracellular inhibitor of granzyme H.
LTA4H	Leukotriene A-4 hydrolase		5.80	75	63	1.2	88.23	-0.259	Enzyme: Hydrolase	Protein metabolism	Cytoplasm; Nucleus	Epoxide hydrolase that catalyzes the final step in the biosynthesis of the proinflammatory mediator leukotriene B4. Has also aminopeptidase activity.
LIMA1	LIM domain and actin-binding protein 1		6.41	122	116	1.1	57.59	-1.074	Cytoskeletal protein	Cell growth and/or maintenance	Cytoplasm; Cytoskeleton; Nucleus	Binds to actin monomers and filaments. Increases the number and size of actin stress fibers and inhibits membrane ruffling. Inhibits actin filament depolymerization. Bundles actin filaments, delays filament nucleation and reduces formation of branched filaments.
LDB3	LIM domain-binding protein 3		8.47	54	61	0.9	59.20	-0.412	Cytoskeletal protein	Sarcomere organization	Cytoplasm; Cytoskeleton	May function as an adapter in striated muscle to couple protein kinase C-mediated signaling via its LIM domains to the cytoskeleton.
EVC2	Limbin	[3]	6.47	179	170	1.1	89.31	-0.466	Unclassified	Unknown	Cytoplasm; Cytoskeleton; Cilium basal body	Positive regulator of the hedgehog signaling pathway (By similarity). Plays a critical role in bone formation and skeletal development.
KIAA1468	LisH domain and HEAT repeat-containing protein KIAA1468		5.28	166	122	1.4	94.89	-0.233	Unclassified	Unknown	Cytoplasm	??
LDHA	L-lactate dehydrogenase A chain		8.46	36	39	0.9	107.10	-0.012	Enzyme: Dehydrogenase	Metabolism; Energy pathways	Cytoplasm; Nucleolus; Cytosol	(S)-lactate + NAD+ = pyruvate + NADH.
LDHB	L-lactate dehydrogenase B chain	[1]	5.72	40	34	1.2	109.67	0.050	Enzyme: Dehydrogenase	Metabolism; Energy pathways	Cytoplasm	(S)-lactate + NAD+ = pyruvate + NADH.
LRP2	Low-density lipoprotein receptor-related protein 2	[2]	4.89	651	408	1.6	65.42	-0.481	Cell surface receptor	Cell communication; Signal transduction	Plasma membrane; Endosome	Acts together with cubilin to mediate HDL endocytosis By similarity. May participate in regulation of parathyroid-hormone and para-thyroid-hormone-related protein release.
SSB	Lupus La protein		6.68	79	78	1.0	70.32	-0.973	RNA binding protein	Regulation of nucleobase, nucleoside, nucleotide and nucleic acid metabolism	Nucleus; Plasma membrane; Cytoplasm	Binds to the 3' poly(U) terminus of nascent RNA polymerase III transcripts, protecting them from exonuclease digestion and facilitating their folding and maturation.
LHB	Lutropin subunit beta		8.30	9	12	0.8	87.69	0.128	Peptide hormone	Cell communication; Signal transduction	Extracellular; Cytoplasm	Promotes spermatogenesis and ovulation by stimulating the testes and ovaries to synthesize steroids.
DCXR	L-xylulose reductase		8.33	21	23	0.9	103.03	0.256	Enzyme: Oxidoreductase	Metabolism; Energy pathways	Integral to membrane; Mitochondrion	Catalyzes the NADPH-dependent reduction of several pentoses, tetroses, trioses, alpha-dicarbonyl compounds and L-xylulose. Participates in the uronate cycle of glucose metabolism. May play a role in the water absorption and cellular osmoregulation in the proximal renal tubules by producing xylitol, an osmolyte, thereby
LY6D	Lymphocyte antigen 6D	[1, 3]	8.11	6	8	0.8	54.87	-0.490	Adhesion molecule	Cell communication; Signal transduction	Plasma membrane	May act as a specification marker at earliest stage specification of lymphocytes between B- and T-cell development. Marks the earliest stage of B-cell specification.
GAA	Lysosomal alpha-glucosidase	[2]	5.42	85	60	1.4	82.49	-0.146	Enzyme: Glucosidase	Metabolism; Energy pathways	Lysosome	Essential for the degradation of glygogen to glucose in lysosomes.
LAMP1	Lysosome-associated membrane glycoprotein 1	[1, 10]	8.84	29	35	0.8	81.72	-0.122	Integral membrane protein	Unknown	Lysosome; Plasma membrane; Late endosome; Golgi apparatus; Mitochondrion	Presents carbohydrate ligands to selectins. Also implicated in tumor cell metastasis.
LAMP2	Lysosome-associated membrane glycoprotein 2	[1]	5.13	36	25	1.4	82.67	-0.162	Adhesion molecule	Cell growth and/or maintenance	Extracellular; Endoplasmic reticulum; Endosome; Lysosome; Plasma membrane; Cytoplasmic vesicle; Golgi apparatus;	Implicated in tumor cell metastasis. May function in protection of the lysosomal membrane from autodigestion, maintenance of the acidic environment of the lysosome, adhesion when expressed on the cell surface (plasma membrane), and inter- and intracellular signal transduction. Protects cells from the toxic effects of methylating mutagens.
LYZ	Lysozyme C	[1, 4-10, 12]	9.28	11	19	0.6	69.85	-0.485	Enzyme: Hydrolase	Metabolism; Energy pathways	Extracellular	Lysozymes have primarily a bacteriolytic function; those in tissues and body fluids are associated with the monocyte-macrophage system and enhance the activity of immunoagents.
MIF	Macrophage migration inhibitory factor		8.24	7	8	0.9	88.16	-0.018	Cytokine	Cell communication; Signal transduction	Extracellular; Cytoplasm	Pro-inflammatory cytokine. Involved in the innate immune response to bacterial pathogens. The expression of MIF at sites of inflammation suggests a role as mediator in regulating the function of macrophages in host defense. Counteracts the anti-inflammatory activity of glucocorticoids. Has phenylpyruvate tautomerase and dopachrome tautomerase activity (in vitro), but the physiological substrate is not known.
MVP	Major vault protein		5.34	135	108	1.3	94.08	-0.361	Transport/cargo protein	Cell growth and/or maintenance	Cytoplasm; Nucleus	Required for normal vault structure. Vaults are multi-subunit structures that may act as scaffolds for proteins involved in signal transduction. Vaults may also play a role in nucleo-cytoplasmic transport.
MDH1	Malate dehydrogenase, cytoplasmic	[1]	6.89	41	41	1.0	97.78	-0.049	Enzyme: Dehydrogenase	Metabolism; Energy pathways	Cytoplasm; Cytosol; Mitochondrion	(S)-malate + NAD+ = oxaloacetate + NADH.
MLEC	Malectin		5.17	39	28	1.4	84.85	-0.322	Carbohydrate binding	Carbohydrate metabolism	Integral to membrane	Carbohydrate-binding protein with a strong ligand preference for Glc2-N-glycan. May play a role in the early steps of protein N-glycosylation By similarity.
MALRD1	MAM and LDL-receptor class A domain-containing protein 1	[1]	5.53	177	135	1.3	71.97	-0.352				
EPDR1	Mammalian ependymin-related protein 1		5.27	22	20	1.1	69.84	-0.575	Adhesion molecule	Cell communication; Signal transduction	Extracellular	A type II transmembrane protein that is similar to two families of cell adhesion molecules, the protocadherins and ependymins. This protein may play a role in calcium-dependent cell adhesion. This protein is glycosylated, and the orthologous mouse protein is localized to the lysosome.
MASP2	Mannan-binding lectin serine protease 2	[1-5, 8-9, 11-12]	5.34	81	58	1.4	68.46	-0.329	Serine protease	Protein metabolism	Extracellular	Serum protease that plays an important role in the activation of the complement system via mannose-binding lectin. After activation by auto-catalytic cleavage it cleaves C2 and C4, leading to their activation and to the formation of C3 convertase.
MAN1A1	Mannosyl-oligosaccharide 1,2-alpha-mannosidase IA	[2, 8]	6.03	86	74	1.2	86.42	-0.265	Enzyme: Hydrolase	Metabolism; Energy pathways	Endoplasmic reticulum; Golgi apparatus	Involved in the maturation of Asn-linked oligosaccharides. Progressively trim alpha-1,2-linked mannose residues from Man9GlcNAc2 to produce Man5GlcNAc2.
MAP7D2	MAP7 domain-containing protein 2	[1]	8.95	127	139	0.9	60.97	-1.134	Unclassified	Unknown	Cytoplasm; Microtubule cytoskeleton	Microtubule cytoskeleton organization.
MAP7D3	MAP7 domain-containing protein 3	[1]	9.34	134	159	0.8	59.74	-1.010	Unclassified	Unknown	Cytoplasm; Microtubule cytoskeleton	Microtubule cytoskeleton organization.
MGP	Matrix Gla protein	[3, 4]	8.66	11	13	0.8	49.48	-1.206	Extracellular matrix protein	Transport	Extracellular	Associates with the organic matrix of bone and cartilage. Thought to act as an inhibitor of bone formation.
MMP9	Matrix metalloproteinase-9		5.44	73	58	1.3	58.29	-0.453	Metallo protease	Protein metabolism	Extracellular; Plasma membrane	May play an essential role in local proteolysis of the extracellular matrix and in leukocyte migration. Could play a role in bone osteoclastic resorption. Cleaves KiSS1 at a Gly-|-Leu bond. Cleaves type IV and type V collagen into large C-terminal three quarter fragments and shorter N-terminal one quarter fragments. Degrades fibronectin but not laminin or Pz-peptide.
MXRA8	Matrix-remodeling-associated protein 8 (limitrin)	[1, 3, 5, 8]	6.53	55	50	1.1	85.58	-0.413	Immunoglobulin	Immune response	Plasma membrane	May play a role in the maturation and maintenance of blood-brain barrier.
MED30	Mediator of RNA polymerase II transcription subunit 30		8.44	21	23	0.9	81.58	-0.622	Transcription regulatory protein	Regulation of nucleobase, nucleoside, nucleotide and nucleic acid metabolism	Nucleus	Component of the Mediator complex, a coactivator involved in the regulated transcription of nearly all RNA polymerase II-dependent genes. Mediator functions as a bridge to convey information from gene-specific regulatory proteins to the basal RNA polymerase II transcription machinery. Mediator is recruited to promoters by direct interactions with regulatory proteins and serves as a scaffold for the assembly of a functional preinitiation complex with RNA polymerase II and the general transcription factors.
MFI2	Melanotransferrin		5.45	86	65	1.3	74.75	-0.278	Ferric iron binding	Immune response	Plasma membrane; Extracellular	Involved in iron cellular uptake. Seems to be internalized and then recycled back to the cell membrane. Binds a single atom of iron per subunit. Could also bind zinc.
MARS	Methionine--tRNA ligase, cytoplasmic		5.82	105	93	1.1	91.13	-0.241	Enzyme: Ligase	Protein metabolism	Cytoplasm; Nucleus; Mitochondrion; Cytosol	ATP + L-methionine + tRNA(Met) = AMP + diphosphate + L-methionyl-tRNA(Met).
CLNS1A	Methylosome subunit pICln		3.97	57	13	4.4	64.49	-0.667	Transport/cargo protein	Transport	Plasma membrane; Nucleus; Cytoplasm	Chaperone that regulates the assembly of spliceosomal U1, U2, U4 and U5 small nuclear ribonucleoproteins (snRNPs), the building blocks of the spliceosome. Thereby, plays an important role in the splicing of cellular pre-mRNAs.
MICALL2	MICAL-like protein 2	[1, 3]	9.63	94	124	0.8	67.04	-0.675	Unclassified	Unknown	Cell membrane; Tight junction	Effector of small Rab GTPases which is involved in junctional complexes assembly through the regulation of cell adhesion molecules transport to the plasma membrane and actin cytoskeleton reorganization. Regulates the endocytic recycling of occludins, claudins and E-cadherin to the plasma membrane and may thereby regulate the establishment of tight junctions and adherens junctions. In parallel, may regulate actin cytoskeleton reorganization directly through interaction with F-actin or indirectly through actinins and filamins. Most probably involved in the processes of epithelial cell differentiation, cell spreading and neurite outgrowth (By similarity).
MFAP4	Microfibril-associated glycoprotein 4		5.21	27	21	1.3	64.23	-0.371	Extracellular matrix protein	Cell growth and/or maintenance	Extracellular	Could be involved in calcium-dependent cell adhesion or intercellular interactions.
MAD1L1	Mitotic spindle assembly checkpoint protein MAD1		5.72	127	113	1.1	82.92	-0.874	Transcription regulatory protein	Cell communication; Signal transduction; Regulation of cell cycle	Kinetochore; Nucleus; Centrosome	Component of the spindle-assembly checkpoint that prevents the onset of anaphase until all chromosomes are properly aligned at the metaphase plate. May recruit MAD2L1 to unattached kinetochores. Has a role in the correct positioning of the septum. Required for anchoring MAD2L1 to the nuclear periphery.
MSN	Moesin		6.09	109	103	1.1	73.40	-0.980	Cytoskeletal protein	Cell growth and/or maintenance	Plasma membrane; Cytoplasm; Nucleus; Cytoskeleton;	Probably involved in connections of major cytoskeletal structures to the plasma membrane.
CD14	Monocyte differentiation antigen CD14	[11]	5.44	35	28	1.3	95.83	-0.079	Cell surface receptor	Immune response	Extracellular; Endoplasmic reticulum; Golgi apparatus; Lysosome; Endosome; Cell surface;	In concert with LBP, binds to monomeric lipopolysaccharide and delivers it to the MD-2/TLR4 complex, thereby mediating the innate immune response to bacterial lipopolysaccharide (LPS). Acts via MyD88, TIRAP and TRAF6, leading to NF-kappa-B activation, cytokine secretion and the inflammatory response. Up-regulates cell surface molecules, including adhesion molecules.
RNGTT	mRNA-capping enzyme	[1]	8.43	81	87	0.9	71.83	-0.592	Enzyme: Adenosyltransferase	Metabolism; Energy pathways	Nucleus	Bifunctional mRNA-capping enzyme exhibiting RNA 5'-triphosphatase activity in the N-terminal part and mRNA guanylyltransferase activity in the C-terminal part. Catalyzes the first two steps of cap formation: by removing the gamma-phosphate from the 5'-triphosphate end of nascent mRNA to yield a diphosphate end, and by transferring the gmp moiety of GTP to the 5'-diphosphate terminus.
MUC16	Mucin-16	[1]	NA	1824	1398	1.3	68.32	-0.310	Integral membrane protein	Cell communication; Signal transduction	Plasma membrane; Endoplasmic reticulum; Golgi apparatus; Extracellular	Thought to provide a protective, lubricating barrier against particles and infectious agents at mucosal surfaces.
MMRN2	Multimerin-2		5.56	127	95	1.3	83.10	-0.449	Extracellular matrix protein	Cell growth and/or maintenance	Extracellular	Inhibits endothelial cells motility and acts as a negative regulator of angiogenesis; it downregulates KDR activation by binding VEGFA.
PRTN3	Myeloblastin	[1, 6-7]	7.79	14	15	0.9	91.27	0.048	Serine protease	Protein metabolism	Plasma membrane; Lysosome; Cytoplasm	Polymorphonuclear leukocyte serine protease that degrades elastin, fibronectin, laminin, vitronectin, and collagen types I, III, and IV (in vitro) and causes emphysema when administered by tracheal insufflation to hamsters.
MNDA	Myeloid cell nuclear differentiation antigen		9.76	42	72	0.6	76.14	-0.641	Transcription factor	Regulation of nucleobase, nucleoside, nucleotide and nucleic acid metabolism	Nucleus	May act as a transcriptional activator/repressor in the myeloid lineage. Plays a role in the granulocyte/monocyte cell-specific response to interferon. Stimulates the DNA binding of the transcriptional repressor protein YY1.
MPO	Myeloperoxidase	[1, 3-8]	9.22	55	69	0.8	81.08	-0.373	Enzyme: Oxidoreductase	Metabolism; Energy pathways	Extracellular; Nucleus; Cytoplasm; Endoplasmic reticulum; Azurophil granule	Part of the host defense system of polymorphonuclear leukocytes. It is responsible for microbicidal activity against a wide range of organisms. In the stimulated PMN, MPO catalyzes the production of hypohalous acids, primarily hypochlorous acid in physiologic situations, and other toxic intermediates that greatly enhance PMN microbicidal activity.
MYL1	Myosin light chain 1/3, skeletal muscle isoform	[5]	4.97	32	24	1.3	77.98	-0.433	Structural protein	Cell growth and/or maintenance	Cytoplasm	Regulatory light chain of myosin. Does not bind calcium.
MYL6	Myosin light polypeptide 6	[4-5, 8]	4.56	28	14	2.0	76.60	-0.404	Cytoskeletal protein	Cell growth and/or maintenance	Cytoskeleton; Cytoplasm	Regulatory light chain of myosin. Does not bind calcium.

MYL12A	Myosin regulatory light chain 12A		4.65	35	23	1.5	57.13	-0.827	Calcium binding protein	Cell communication; Signal transduction	Cytoskeleton	Myosin regulatory subunit that plays an important role in regulation of both smooth muscle and nonmuscle cell contractile activity via its phosphorylation. Implicated in cytokinesis, receptor capping, and cell locomotion By similarity.
MYH10	Myosin-10		5.43	387	330	1.2	82.68	-0.863	Structural protein	Cell growth and/or maintenance	Cytoplasm	Cellular myosin that appears to play a role in cytokinesis, cell shape, and specialized functions such as secretion and capping. Involved with LARP6 in the stabilization of type I collagen mRNAs for CO1A1 and CO1A2. During cell spreading, plays an important role in cytoskeleton reorganization, focal contacts formation (in the central part but not the margins of spreading cells), and lamellipodial extension; this function is mechanically antagonized by MYH9.
MYH14	Myosin-14		5.52	366	323	1.1	82.01	-0.813	Structural protein	Cell growth and/or maintenance	Cytoskeleton; Cytoplasm	Cellular myosin that appears to play a role in cytokinesis, cell shape, and specialized functions such as secretion and capping.
**MYH9**	**Myosin-9 (myosin heavy chain IIa)**	[1, 3-5, 8]	5.50	377	330	1.1	82.04	-0.855	Structural protein	Cell growth and/or maintenance	Cytoplasm; Cytosol; Nucleus; Mitochondrion	Cellular myosin that appears to play a role in cytokinesis, cell shape, and specialized functions such as secretion and capping. During cell spreading, plays an important role in cytoskeleton reorganization, focal contacts formation (in the margins but not the central part of spreading cells), and lamellipodial retraction; this function is mechanically antagonized by MYH10.
DDAH1	N(G),N(G)-dimethylarginine dimethylaminohydrolase 1		5.53	41	32	1.3	96.13	-0.140	Enzyme: Hydrolase	Metabolism; Energy pathways	Cytoplasm; Integral to membrane	Hydrolyzes N(G),N(G)-dimethyl-L-arginine (ADMA) and N(G)-monomethyl-L-arginine (MMA) which act as inhibitors of NOS. Has therefore a role in the regulation of nitric oxide generation.
**DDAH2**	**N(G),N(G)-dimethylarginine dimethylaminohydrolase 2**	[5, 8]	5.66	32	26	1.2	98.25	0.007	Enzyme: Hydrolase	Metabolism; Energy pathways	Cytoplasm	Hydrolyzes N(G),N(G)-dimethyl-L-arginine (ADMA) and N(G)-monomethyl-L-arginine (MMA) which act as inhibitors of NOS. Has therefore a role in the regulation of nitric oxide generation.
NAGK	N-acetyl-D-glucosamine kinase		5.82	39	32	1.2	93.32	-0.008	Enzyme: Phosphotransferase	Metabolism; Energy pathways	Extracellular vesicular exosome	Converts endogenous N-acetylglucosamine (GlcNAc), a major component of complex carbohydrates, from lysosomal degradation or nutritional sources into GlcNAc 6-phosphate.
NAGS	N-acetylglutamate synthase, mitochondrial		8.95	49	57	0.9	85.10	-0.224	Enzyme: Transferase	Metabolism; Energy pathways	Mitochondrion	Plays a role in the regulation of ureagenesis by producing the essential cofactor N-acetylglutamate (NAG), thus modulating carbamoylphosphate synthase I (CPSI) activity.
**PGLYRP2**	**N-acetylmuramoyl-L-alanine amidase**	[2]	7.64	50	51	1.0	86.20	-0.177	Integral membrane protein	Immune response	Extracellular; Plasma membrane	May play a scavenger role by digesting biologically active peptidoglycan (PGN) into biologically inactive fragments. Has no direct bacteriolytic activity.
**NAPSA**	**Napsin-A**	[1, 3]	5.62	33	26	1.3	93.95	0.193	Aspartic protease	Protein metabolism	Cytoplasm; Integral to membrane	May be involved in processing of pneumocyte surfactant precursors.
**NACA**	**Nascent polypeptide-associated complex subunit alpha**	[3]	4.52	39	23	1.7	74.88	-0.655	Chaperone	Protein metabolism	Nucleus; Cytoplasm; Ribosome	Prevents inappropriate targeting of non-secretory polypeptides to the endoplasmic reticulum (ER). Binds to nascent polypeptide chains as they emerge from the ribosome and blocks their interaction with the signal recognition particle (SRP), which normally targets nascent secretory peptides to the ER. Also reduces the inherent affinity of ribosomes for protein translocation sites in the ER membrane (M sites).
PVRL2	Nectin-2, Isoform Alpha		4.70	65	37	1.8	75.40	-0.307	Integral membrane protein	Cell communication; Signal transduction	Plasma membrane; Nucleus	Probable cell adhesion protein.
**NELFB**	**Negative elongation factor B**	[1]	5.77	79	67	1.2	102.21	-0.116	Transcription regulatory protein	Regulation of nucleobase, nucleoside, nucleotide and nucleic acid metabolism	Nucleus; Cytoplasm	Essential component of the NELF complex, a complex that negatively regulates the elongation of transcription by RNA polymerase II. The NELF complex, which acts via an association with the DSIF complex and causes transcriptional pausing, is counteracted by the P-TEFb kinase complex. May be able to induce chromatin unfolding.
**SYNE1**	**Nesprin-1**	[1]	5.37	1437	1122	1.3	88.87	-0.622	Unclassified	Cell growth and/or maintenance	Nucleus; Sarcoplasmic reticulum; Golgi apparatus; Cytoplasm; Nucleolus; Cytoskeleton	Multi-isomeric modular protein which forms a linking network between organelles and the actin cytoskeleton to maintain the subcellular spatial organization. Component of SUN-protein-containing multivariate complexes also called LINC complexes which link the nucleoskeleton and cytoskeleton by providing versatile outer nuclear membrane attachment sites for cytoskeletal filaments. May be involved in the maintenance of nuclear organization and structural integrity. Connects nuclei to the cytoskeleton by interacting with the nuclear envelope and with F-actin in the cytoplasm. May be required for centrosome migration to the apical cell surface during early
NEURL4	Neuralized-like protein 4		5.56	178	141	1.3	78.96	-0.316	**Unclassified**	**Cell growth and/or maintenance**	**Cytoplasm; Centriole**	**Promotes CCP110 ubiquitination and proteasome-dependent degradation. By counteracting accumulation of CP110, maintains normal centriolar homeostasis and preventing formation of ectopic microtubular organizing centers.**
NBEAL2	Neurobeachin-like protein 2		5.95	286	238	1.2	95.32	-0.094	Unclassified	Signal transduction	Endoplasmic reticulum	Probably involved in thrombopoiesis. Plays a role in the development or secretion of alpha-granules, that contain several growth factors important for platelet biogenesis.
GANAB	Neutral alpha-glucosidase AB		5.58	117	90	1.3	80.66	-0.405	Enzyme: Hydrolase	Carbohydrate metabolism	Endoplasmic reticulum; Centrosome	Cleaves sequentially the 2 innermost alpha-1,3-linked glucose residues from the Glc2Man9GlcNAc2 oligosaccharide precursor of immature glycoproteins.
**DEFA1**	**Neutrophil defensin 1 (Defensin, Alpha 1)**	[1, 4-10, 12]	8.68	1	4	0.3	65.33	0.300	Defensin	Immune response	Cytoplasm	Defensin 1 and defensin 2 have antibacterial, fungicide and antiviral activities. Has antimicrobial activity against Gram-negative and Gram-positive bacteria. Defensins are thought to kill microbes by permeabilizing their plasma membrane.
**DEFA3**	**Neutrophil defensin 3**	[4, 6]	8.33	2	4	0.5	62.00	0.123	**Defensin**	**Immune response**	**Extracellular**	**Defensin 2 and defensin 3 have antibiotic, fungicide and antiviral activities. Has antimicrobial activity against Gram-negative and Gram-positive bacteria. Defensins are thought to kill microbes by permeabilizing their plasma membrane.**
DEFA4	Neutrophil defensin 4		8.98	1	5	0.2	91.21	0.661	**Defensin**	**Immune response**	**Secretory granule**	**Has antimicrobial activity against Gram-negative bacteria, and to a lesser extent also against Gram-positive bacteria and fungi. Protects blood cells against infection with HIV-1 (in vitro). Inhibits corticotropin (ACTH)-stimulated corticosterone production.**
**ELANE**	**Neutrophil elastase (*aka* leukocyte elastase)**	[1, 6-7, 9-10]	9.89	12	22	0.5	96.64	0.100	Serine protease	Protein metabolism	Cytoplasm; Plasma membrane; Extracellular; Zymogen granule	Modifies the functions of natural killer cells, monocytes and granulocytes. Inhibits C5a-dependent neutrophil enzyme release and chemotaxis.
**LCN2**	**Neutrophil gelatinase-associated lipocalin**	[7]	9.02	18	23	0.8	78.76	-0.461	Transport/cargo protein	Transport	Secretory granule; Extracellular	Iron-trafficking protein involved in multiple processes such as apoptosis, innate immunity and renal development.Involved in apoptosis due to interleukin-3 (IL3) deprivation: iron-loaded form increases intracellular iron concentration without promoting apoptosis, while iron-free form decreases intracellular iron levels, inducing expression of the proapoptotic protein BCL2L11/BIM, resulting in apoptosis. Involved in innate immunity, possibly by sequestrating iron, leading to limit bacterial
FAM129B	Niban-like protein 1		5.82	98	83	1.2	81.82	-0.463	Unclassified	Anti-apoptosis	Nucleus; Cytoplasm; Cell junction	May play a role in apoptosis suppression. May promote melanoma cell invasion in vitro.
NAMPT	Nicotinamide phosphoribosyltransferase		6.69	63	61	1.0	86.31	-0.429	Cytokine	Anti-apoptosis	Extracellular; Cytoplasm; Nucleus	Catalyzes the condensation of nicotinamide with 5-phosphoribosyl-1-pyrophosphate to yield nicotinamide mononucleotide, an intermediate in the biosynthesis of NAD. It is the rate limiting component in the mammalian NAD biosynthesis pathway.
**NID1**	**Nidogen-1**	[2]	5.05	149	98	1.5	67.80	-0.409	Extracellular matrix protein	Cell growth and/or maintenance	Extracellular	Sulfated glycoprotein widely distributed in basement membranes and tightly associated with laminin. Also binds to collagen IV and perlecan. It probably has a role in cell-extracellular matrix interactions.
**NONO**	**Non-POU domain-containing octamer-binding protein**	[3]	9.01	68	73	0.9	57.01	-0.999	RNA binding protein	Regulation of nucleobase, nucleoside, nucleotide and nucleic acid metabolism	Nucleus; Nucleolus; Mitochondrion	DNA- and RNA binding protein, involved in several nuclear processes. Binds the conventional octamer sequence in double-stranded DNA. Also binds single-stranded DNA and RNA at a site independent of the duplex site. Involved in pre-mRNA splicing, probably as a heterodimer with SFPQ.
**RNASE2**	**Non-secretory ribonuclease**	[2]	9.20	5	12	0.4	58.88	-0.669	Ribonuclease	Regulation of nucleobase, nucleoside, nucleotide and nucleic acid metabolism	Extracellular; Cytoplasm; Plasma membrane	This is a non-secretory ribonuclease. It is a pyrimidine specific nuclease with a slight preference for U. Cytotoxin and helminthotoxin. Selectively chemotactic for dendritic cells. Possesses a wide variety of biological activities.
NUTF2	Nuclear transport factor 2		5.10	14	8	1.8	82.99	-0.201	Transport/cargo protein	Transport	Cytoplasm; Nucleus	Facilitates protein transport into the nucleus. Interacts with the nucleoporin p62 and with Ran. Acts at a relatively late stage of nuclear protein import, subsequent to the initial docking of nuclear import ligand at the nuclear envelope. Could be part of a multicomponent system of cytosolic factors that assemble at the pore complex during nuclear import.
NUCB1	Nucleobindin-1		5.09	97	64	1.5	70.64	-1.156	Calcium binding protein	Cell communication; Signal transduction	Golgi apparatus; Extracellular; Cytoplasm; Nucleus	Major calcium-binding protein of the Golgi. May have a role in calcium homeostasis By similarity.
**NOLC1**	**Nucleolar and coiled-body phosphoprotein 1**	[4]	9.46	106	133	0.8	43.56	-1.208	**Transcription factor**	**Regulation of nucleobase, nucleoside, nucleotide and nucleic acid metabolism**	**Nucleolus; Cytoplasm**	**Related to nucleologenesis, may play a role in the maintenance of the fundamental structure of the fibrillar center and dense fibrillar component in the nucleolus. It has intrinsic GTPase and ATPase activities. May play an important role in transcription catalyzed by RNA polymerase I.**
**NCL**	**Nucleolin**	[3, 4]	4.60	176	116	1.5	50.04	-1.131	RNA binding protein	Regulation of nucleobase, nucleoside, nucleotide and nucleic acid metabolism	Nucleolus; Nucleus; Cytoplasm; Plasma membrane	Nucleolin is the major nucleolar protein of growing eukaryotic cells. It is found associated with intranucleolar chromatin and pre-ribosomal particles. It induces chromatin decondensation by binding to histone H1. It is thought to play a role in pre-rRNA transcription and ribosome assembly. May play a role in the process of transcriptional elongation.
**NPM1**	**Nucleophosmin**	[1, 3-4]	4.64	65	41	1.6	62.69	-0.970	Chaperone	Protein metabolism	Centrosome; Cytoplasm; Nucleolus; Nucleus	Involved in diverse cellular processes such as ribosome biogenesis, centrosome duplication, protein chaperoning, histone assembly, cell proliferation, and regulation of tumor suppressors p53/TP53 and ARF. Binds ribosome presumably to drive ribosome nuclear export. Associated with nucleolar ribonucleoprotein structures and bind single-stranded nucleic acids. Acts as a chaperonin for the core histones H3, H2B and H4.In concert with BRCA2, regulates centrosome duplication. Regulates centriole duplication: phosphorylation by PLK2 is able to trigger centriole replication.
NME1	Nucleoside diphosphate kinase A		5.82	21	18	1.2	86.42	-0.208	Enzyme: Phosphotransferase	Metabolism; Energy pathways	Cytoplasm; Nucleus; Extracellular; Mitochondrion	Major role in the synthesis of nucleoside triphosphates other than ATP.Possesses nucleoside-diphosphate kinase, serine/threonine-specific protein kinase, geranyl and farnesyl pyrophosphate kinase, histidine protein kinase and 3'-5' exonuclease activities. Involved in cell proliferation, differentiation and development, signal transduction, G protein-coupled receptor endocytosis, and gene expression.
NAP1L1	Nucleosome assembly protein 1-like 1		4.34	104	49	2.1	68.76	-0.939	Transcription regulatory protein	Regulation of nucleobase, nucleoside, nucleotide and nucleic acid metabolism	Nucleus; Mitochondrion	May be involved in modulating chromatin formation and contribute to regulation of cell proliferation.
NAP1L4	Nucleosome assembly protein 1-like 4		4.60	83	46	1.8	65.43	-0.788	Chaperone	Regulation of nucleobase, nucleoside, nucleotide and nucleic acid metabolism	Nucleus; Cytoplasm	Poly(A) RNA binding.
OLA1	Obg-like ATPase 1		7.64	56	57	1.0	84.47	-0.401	Enzyme: Hydrolase	ATP catabolic process	Nucleolus	Hydrolyzes ATP, and can also hydrolyze GTP with lower efficiency. Has lower affinity for GTP.
NIT2	Omega-amidase NIT2		6.83	31	31	1.0	84.17	-0.186	**Enzyme: Hydrolase**	**Metabolism; Energy pathways**	**Cytoplasm**	**Has a omega-amidase activity. The role of omega-amidase is to remove potentially toxic intermediates by converting alpha-ketoglutaramate and alpha-ketosuccinamate to biologically useful alpha-ketoglutarate and oxaloacetate, respectively. Overexpression decreases the colony-forming capacity of cultured cells by arresting cells in the G2 phase of the cell cycle.**
**ORC4**	**Origin recognition complex subunit 4**	[3]	8.08	47	49	1.0	99.24	-0.208	**DNA binding protein**	**DNA replication**	**Nucleus**	**Component of the origin recognition complex (ORC) that binds origins of replication. DNA-binding is ATP-dependent. The specific DNA sequences that define origins of replication have not been identified yet. ORC is required to assemble the pre-replication complex necessary to initiate DNA replication. Binds histone H3 and H4 trimethylation marks H3K9me3, H3K27me3 and H4K20me3.**
OSTF1	Osteoclast-stimulating factor 1		5.44	31	26	1.2	79.34	-0.557	Adapter molecule	Cell communication; Signal transduction	Nucleus; Cytoskeleton; Cytoplasm	Induces bone resorption, acting probably through a signaling cascade which results in the secretion of factor(s) enhancing osteoclast formation and activity.
**SPP1**	**Osteopontin**	[1-5, 8-12]	4.35	72	28	2.6	52.32	-1.279	Adhesion molecule	Cell growth and/or maintenance	Extracellular	Binds tightly to hydroxyapatite. Appears to form an integral part of the mineralized matrix. Probably important to cell-matrix interaction.
SPP1	Osteopontin, Isoform B		4.39	72	28	2.6	59.00	-1.089	Adhesion molecule	Cell growth and/or maintenance	Extracellular	Binds tightly to hydroxyapatite. Appears to form an integral part of the mineralized matrix. Probably important to cell-matrix interaction.
**SPP1**	**Osteopontin, Isoform C**	[11]	4.35	73	27	2.7	55.52	-1.136	Adhesion molecule	Cell growth and/or maintenance	Extracellular	Binds tightly to hydroxyapatite. Appears to form an integral part of the mineralized matrix. Probably important to cell-matrix interaction.
SPP1	Osteopontin, Isoform D		4.56	64	29	2.2	58.63	-1.057	Adhesion molecule	Cell growth and/or maintenance	Extracellular	Binds tightly to hydroxyapatite. Appears to form an integral part of the mineralized matrix. Probably important to cell-matrix interaction.
**OTOA**	**Otoancorin**	[1]	5.50	128	101	1.3	99.95	-0.058	**Unclassified**	**Unknown**	**Plasma membrane**	**May act as an adhesion molecule.**
OTOG	Otogelin		5.59	286	213	1.3	73.07	-0.130	**Extracellular matrix protein**	**Cell growth and/or maintenance**	**Extracellular**	**Glycoprotein specific to acellular membranes of the inner ear. May be required for the anchoring of the otoconial membranes and cupulae to the underlying neuroepithelia in the vestibule. May be involved in the organization and/or stabilization of the fibrillar network that compose the tectorial membrane in the cochlea.**
**PAN3**	**PAB-dependent poly(A)-specific ribonuclease subunit PAN3**	[3]	8.80	71	81	0.9	78.24	-0.226	**Ribonuclease**	**Regulation of nucleobase, nucleoside, nucleotide and nucleic acid metabolism**	**Cytoplasm**	**Regulatory subunit of the poly(A)-nuclease (PAN) deadenylation complex, one of two cytoplasmic mRNA deadenylases involved in general and miRNA-mediated mRNA turnover. PAN specifically shortens poly(A) tails of RNA when the poly(A) stretch is bound by poly(A)-binding protein (PABP), which is followed by rapid degradation of the shortened mRNA tails by the CCR4-NOT complex.**
PTMS	Parathymosin		4.14	47	17	2.8	35.94	-2.007	DNA binding protein	Regulation of nucleobase, nucleoside, nucleotide and nucleic acid metabolism	Nucleus	Parathymosin may mediate immune function by blocking the effect of prothymosin alpha which confers resistance to certain opportunistic infections.

PGLYRP1	Peptidoglycan recognition protein 1		8.23	11	13	0.8	73.03	-0.379	**Secreted polypeptide**	**Immune response**	**Extracellular**	**Pattern receptor that binds to murein peptidoglycans (PGN) of Gram-positive bacteria. Has bactericidal activity towards Gram-positive bacteria. May kill Gram-positive bacteria by interfering with peptidoglycan biosynthesis. Binds also to Gram-negative bacteria, and has bacteriostatic activity towards Gram-negative bacteria. Plays a role in innate immunity.**
PAM	Peptidyl-glycine alpha-amidating monooxygenase		5.83	121	97	1.2	77.05	-0.366	Enzyme: Oxygenase	Protein metabolism	Cytoplasm; Endoplasmic reticulum; Golgi apparatus; Nucleus; Extracellular; Axon; Dendrite	Bifunctional enzyme that catalyzes 2 sequential steps in C-terminal alpha-amidation of peptides. The monooxygenase part produces an unstable peptidyl(2-hydroxyglycine) intermediate that is dismutated to glyoxylate and the corresponding desglycine peptide amide by the lyase part.
**PPIA**	**Peptidyl-prolyl cis-trans isomerase A**	**[8]**	7.82	19	20	1.0	61.83	-0.330	Enzyme: Isomerase	Protein folding; Peptide metabolism	Cytoplasm; Nucleolus; Mitochondrion	PPIases accelerate the folding of proteins. It catalyzes the cis-trans isomerization of proline imidic peptide bonds in oligopeptides.
PPIB	Peptidyl-prolyl cis-trans isomerase B		9.25	26	32	0.8	70.71	-0.437	Chaperone	Protein metabolism	Endoplasmic reticulum; Extracellular; Plasma membrane;	PPIases accelerate the folding of proteins. It catalyzes the cis-trans isomerization of proline imidic peptide bonds in oligopeptides.
FKBP4	Peptidyl-prolyl cis-trans isomerase FKBP4		5.35	78	64	1.2	73.56	-0.648	Enzyme: Isomerase	Metabolism; Energy pathways	Cytoplasm; Nucleus	Immunophilin protein with PPIase and co-chaperone activities. Component of steroid receptors heterocomplexes through interaction with heat-shock protein 90 (HSP90). May play a role in the intracellular trafficking of heterooligomeric forms of steroid hormone receptors between cytoplasm and nuclear compartments. The isomerase activity controls neuronal growth cones via regulation of TRPC1 channel opening. Acts also as a regulator of microtubule dynamics by inhibiting MAPT/TAU ability to promote microtubule assembly. May have a protective role against oxidative stress in mitochondria.
**PRX**	**Periaxin**	**[3]**	7.22	197	197	1.0	89.60	-0.223	**Cytoskeletal associated protein**	**Cell growth and/or maintenance**	**Nucleus; Plasma membrane**	**Seems to be required for maintenance of peripheral nerve myelin sheath. May have a role in axon-glial interactions, possibly by interacting with the cytoplasmic domains of integral membrane proteins such as myelin-associated glycoprotein in the periaxonal regions of the Schwann cell plasma membrane. May have a role in the early phases of myelin deposition.**
**PWP2**	**Periodic tryptophan protein 2 homolog**	**[3]**	5.76	117	97	1.2	82.01	-0.220	**Cell cycle control protein**	**Cell communication; Signal transduction**	**Nucleolus; Ribosome**	**WD family protein essential for the assembly of the 90 S pre-ribosomal particle.**
PPL	Periplakin		5.47	345	290	1.2	85.26	-0.982	Cytoskeletal protein	Cell growth and/or maintenance	Cell surface; Plasma membrane; Cytoplasm	Component of the cornified envelope of keratinocytes. May link the cornified envelope to desmosomes and intermediate filaments. May act as a localization signal in PKB/AKT-mediated signaling.
**PRDX1**	**Peroxiredoxin-1**	**[10]**	8.27	23	25	0.9	77.27	-0.276	Enzyme: Peroxidase	Metabolism; Energy pathways	Cytoplasm; Nucleus; Nucleolus; Mitochondrion	Involved in redox regulation of the cell. Reduces peroxides with reducing equivalents provided through the thioredoxin system but not from glutaredoxin. May play an important role in eliminating peroxides generated during metabolism. Might participate in the signaling cascades of growth factors and tumor necrosis factor-alpha by regulating the intracellular concentrations of H2O2.
**PRDX2**	**Peroxiredoxin-2**	**[1, 7]**	5.67	27	24	1.1	87.06	-0.210	Enzyme: Peroxidase	Metabolism; Energy pathways	Cytoplasm; Plasma membrane	Involved in redox regulation of the cell. Reduces peroxides with reducing equivalents provided through the thioredoxin system. It is not able to receive electrons from glutaredoxin. May play an important role in eliminating peroxides generated during metabolism. Might participate in the signaling cascades of growth factors and tumor necrosis factor-alpha by regulating the intracellular concentrations of H2O2.
**PRDX6**	**Peroxiredoxin-6**	**[8]**	6.02	32	30	1.1	92.69	-0.219	Enzyme: Peroxidase	Metabolism; Energy pathways	Cytoplasm; Lysosome	Involved in redox regulation of the cell. Can reduce H2O2 and short chain organic, fatty acid, and phospholipid hydroperoxides. May play a role in the regulation of phospholipid turnover as well as in protection against oxidative injury.
HSD17B4	Peroxisomal multifunctional enzyme type 2		8.96	78	89	0.9	88.27	-0.132	Enzyme: Dehydrogenase	Biological process: Metabolism; Energy pathways	Peroxisome; Cytoplasm; Nucleolus; Mitochondrion	Bifunctional enzyme acting on the peroxisomal beta-oxidation pathway for fatty acids. Catalyzes the formation of 3-ketoacyl-CoA intermediates from both straight-chain and 2-methyl-branched-chain fatty acids.
**PEX1**	**Peroxisome biogenesis factor 1**	**[3]**	5.91	158	136	1.2	94.21	-0.277	**ATPase**	**Cell communication; Signal transduction**	**Cytoplasm**	**Required for stability of PEX5 and protein import into the peroxisome matrix. Anchored by PEX26 to peroxisome membranes, possibly to form heteromeric AAA ATPase complexes required for the import of proteins into peroxisomes.**
**GIGYF2**	**PERQ amino acid-rich with GYF domain-containing protein 2**	**[3]**	5.45	222	190	1.2	56.54	-1.234	Unclassified	Cell communication; Signal transduction	Nucleus; Cytoplasm	May act cooperatively with GRB10 to regulate tyrosine kinase receptor signaling, including IGF1 and insulin receptors.
**PHF14**	**PHD finger protein 14**	**[1]**	5.22	169	139	1.2	59.90	-1.005	**Unclassified**	**Unknown**	**Nucleus; Cytoplasm**	**Negative regulation of platelet-derived growth factor receptor-alpha signaling pathway and transcription from RNA polymerase II promoter.**
FARSA	Phenylalanine--tRNA ligase alpha subunit		7.46	65	76	0.9	80.22	-0.458	Enzyme: Ligase	Protein metabolism	Cytoplasm	ATP + L-phenylalanine + tRNA(Phe) = AMP + diphosphate + L-phenylalanyl-tRNA(Phe).
**PHIP**	**PH-interacting protein**	**[1]**	9.02	235	269	0.9	71.63	-0.705	Ligand	Cell communication; Signal transduction	Nucleus; Extracellular vesicular exosome	Probable regulator of the insulin and insulin-like growth factor signaling pathways. Stimulates cell proliferation through regulation of cyclin transcription and has an anti-apoptotic activity through AKT1 phosphorylation and activation. Plays a role in the regulation of cell morphology and cytoskeletal organization.
**PHACTR4**	**Phosphatase and actin regulator 4**	**[1]**	6.20	106	99	1.1	66.04	-0.897	**Regulatory/other subunit**	**Signal transduction**	**Perinuclear region; Synapse; Cytoplasm**	**Regulator of protein phosphatase 1 (PP1) required for neural tube and optic fissure closure, and enteric neural crest cell (ENCCs) migration during development. Acts as an activator of PP1 by interacting with PPP1CA and preventing phosphorylation of PPP1CA at 'Thr-320'. Also acts as a regulator of migration of enteric neural crest cells (ENCCs) by activating PP1, leading to dephosphorylation and subsequent activation of cofilin (COF1 or COF2) and repression of the integrin signaling through the RHO/ROCK pathway (By similarity).**
**LCAT**	**Phosphatidylcholine-sterol acyltransferase**	**[2]**	5.71	42	32	1.3	87.38	-0.175	**Enzyme: Acyltransferase**	**Metabolism; Energy pathways**	**Extracellular**	**Central enzyme in the extracellular metabolism of plasma lipoproteins. Synthesized mainly in the liver and secreted into plasma where it converts cholesterol and phosphatidylcholines (lecithins) to cholesteryl esters and lysophosphatidylcholines on the surface of high and low density lipoproteins**
**PEBP1**	**Phosphatidylethanolamine-binding protein 1 (neuropolypeptide h3)**	**[10]**	7.43	24	24	1.0	75.38	75.380	Protease inhibitor	Cell communication; Signal transduction	Cytoplasm; Plasma membrane	Binds ATP, opioids and phosphatidylethanolamine. Has lower affinity for phosphatidylinositol and phosphatidylcholine. Serine protease inhibitor which inhibits thrombin, neuropsin and chymotrypsin but not trypsin, tissue type plasminogen activator and elastase (By similarity).
**GPLD1**	**Phosphatidylinositol-glycan-specific phospholipase D**	**[1]**	5.78	85	66	1.3	83.53	-0.152	**Enzyme: Phospholipase**	**Cell communication; Signal transduction**	**Extracellular**	**This protein hydrolyzes the inositol phosphate linkage in proteins anchored by phosphatidylinositol glycans (GPI-anchor) thus releasing these proteins from the membrane.**
**PGK1**	**Phosphoglycerate kinase**	**[1]**	8.30	50	53	0.9	90.48	-0.083	Enzyme: Phosphotransferase	Metabolism; Energy pathways	Cytoplasmic vesicle; Cytoplasm; Nucleus; Mitochondrion	In addition to its role as a glycolytic enzyme, it seems that PGK-1 acts as a polymerase alpha cofactor protein (primer recognition protein).
PGAM1	Phosphoglycerate mutase 1		6.75	36	35	1.0	84.90	-0.507	Enzyme: Mutase	Metabolism; Energy pathways	Cytoplasm	Interconversion of 3- and 2-phosphoglycerate with 2,3-bisphosphoglycerate as the primer of the reaction.
PLD3	Phospholipase D3		6.02	48	41	1.2	86.43	-0.188	Enzyme: Phospholipase	Metabolism; Energy pathways	Endoplasmic reticulum; Golgi apparatus	Probably involved in APP processing.
ATIC	Phosphoribosylaminoimidazolecarboxamide formyltransferase		6.27	70	65	1.1	93.94	-0.108	Enzyme: Hydrolase	Metabolism; Energy pathways	Cytoplasm; Mitochondrion	Bifunctional enzyme that catalyzes 2 steps in purine biosynthesis: 10-formyltetrahydrofolate + 5-amino-1-(5-phospho-D-ribosyl)imidazole-4-carboxamide = tetrahydrofolate + 5-formamido-1-(5-phospho-D-ribosyl)imidazole-4-carboxamide and IMP + H2O = 5-formamido-1-(5-phospho-D-ribosyl)imidazole-4-carboxamide.
PTER	Phosphotriesterase-related protein		6.07	44	37	1.2	96.68	-0.220	**Enzyme: Hydrolase**	**Metabolism; Energy pathways**	**Extracellular vesicular exosome**	**Hydrolase activity, acting on ester bonds. Involved in Epithelial cell differentiation.**
**SERPINF1**	**Pigment epithelium-derived factor**	**[1]**	5.90	50	45	1.1	93.58	-0.254	Serine protease	Cell communication; Signal transduction	Extracellular	Neurotrophic protein; induces extensive neuronal differentiation in retinoblastoma cells. Potent inhibitor of angiogenesis. As it does not undergo the S (stressed) to R (relaxed) conformational transition characteristic of active serpins, it exhibits no serine protease inhibitory activity.
KLKB1	Plasma kallikrein		8.57	53	66	0.8	70.52	-0.312	**Serine protease**	**Protein metabolism**	**Extracellular**	**The enzyme cleaves Lys-Arg and Arg-Ser bonds. It activates, in a reciprocal reaction, factor XII after its binding to a negatively charged surface. It also releases bradykinin from HMW kininogen and may also play a role in the renin-angiotensin system by converting prorenin into renin.**
ATP2B2	Plasma membrane calcium-transporting ATPase 2		5.66	159	140	1.1	91.84	-0.165	ATPase	Transport	Plasma membrane	This magnesium-dependent enzyme catalyzes the hydrolysis of ATP coupled with the transport of calcium out of the cell.
**SERPING1**	**Plasma protease C1 inhibitor**	**[1-2, 11]**	5.97	47	41	1.1	85.86	-0.183	Protease inhibitor	Protein metabolism	Extracellular matrix; Extracellular	Activation of the C1 complex is under control of the C1-inhibitor. It forms a proteolytically inactive stoichiometric complex with the C1r or C1s proteases. May play a potentially crucial role in regulating important physiological pathways including complement activation, blood coagulation, fibrinolysis and the generation of kinins. Very efficient inhibitor of FXIIa. Inhibits chymotrypsin and kallikrein.
**SERPINA5**	**Plasma serine protease inhibitor**	**[1-2, 8, 11]**	9.17	37	43	0.9	89.79	-0.156	Protease inhibitor	Protein metabolism	Extracellular; Plasma membrane; Nucleus	Heparin-dependent serine protease inhibitor acting in body fluids and secretions. Inactivates serine proteases by binding irreversibly to their serine activation site. Involved in the regulation of intravascular and extravascular proteolytic activities. Plays hemostatic roles in the blood plasma. Acts as a procoagulant and proinflammatory factor by inhibiting the anticoagulant activated protein C factor as well as the generation of activated protein C factor by the thrombin/thrombomodulin complex. Acts as an anticoagulant factor by inhibiting blood coagulation factors like prothrombin, factor XI, factor Xa, plasma kallikrein and fibrinolytic enzymes such as tissue- and urinary-type plasminogen activators.
**PLG**	**Plasminogen**	**[1, 3-5, 7-8]**	7.08	90	89	1.0	53.10	-0.720	Protease	Protein metabolism	Extracellular	Plasmin dissolves the fibrin of blood clots and acts as a proteolytic factor in a variety of other processes including embryonic development, tissue remodeling, tumor invasion, and inflammation. In ovulation, weakens the walls of the Graafian follicle. It activates the urokinase-type plasminogen activator, collagenases and several complement zymogens, such as C1 and C5. Cleavage of fibronectin and laminin leads to cell detachment and apoptosis.Binds to cells.
PAFAH1B1	Platelet-activating factor acetylhydrolase IB subunit alpha		6.97	57	56	1.1	71.49	-0.508	Enzyme: Hydrolase	Metabolism; Energy pathways	Cytoplasm; Centrosome	Non-catalytic subunit of an acetylhydrolase complex which inactivates platelet-activating factor (PAF) by removing the acetyl group at the SN-2 position By similarity. Positively regulates the activity of the minus-end directed microtubule motor protein dynein. May enhance dynein-mediated microtubule sliding by targeting dynein to the microtubule plus end.May also play a role in other forms of cell locomotion including the migration of fibroblasts during wound healing.
PAFAH1B2	Platelet-activating factor acetylhydrolase IB subunit beta		5.57	30	22	1.4	96.62	-0.305	Enzyme: Hydrolase	Cell communication; Signal transduction	Cytoplasm; Extracellular	Inactivates PAF by removing the acetyl group at the sn-2 position. This is a catalytic subunit.
PAFAH1B3	Platelet-activating factor acetylhydrolase IB subunit gamma		6.33	26	21	1.2	94.04	-0.360	Enzyme: Acyltransferase	Metabolism; Energy pathways	Cytoplasm	Inactivates paf by removing the acetyl group at the sn-2 position. This is a catalytic subunit. Plays an important role during the development of brain.
PLEC	Plectin		5.74	782	699	1.1	86.31	-0.665	Anchor protein	Cytoskeletal anchoring	Cytoplasm; Nucleus; Cytoskeleton; Nucleolus; Plasma membrane; Mitochondrion	nterlinks intermediate filaments with microtubules and microfilaments and anchors intermediate filaments to desmosomes or hemidesmosomes. Could also bind muscle proteins such as actin to membrane complexes in muscle. May be involved not only in the filaments network, but also in the regulation of their dynamics.
**NPHS2**	**Podocin**	**[4]**	8.95	45	51	0.9	93.47	-0.170	**Integral membrane protein**	**Cell communication; Signal transduction**	**Plasma membrane**	**Plays a role in the regulation of glomerular permeability, acting probably as a linker between the plasma membrane and the cytoskeleton.**
PCBP1	Poly(rC)-binding protein 1		6.66	31	30	1.0	87.72	-0.106	RNA binding protein	Regulation of nucleobase, nucleoside, nucleotide and nucleic acid metabolism	Nucleus; Cytoplasm; Nucleolus; Mitochondrion	Single-stranded nucleic acid binding protein that binds preferentially to oligo dC.
PCBP2	Poly(rC)-binding protein 2		6.33	33	31	1.1	86.33	-0.137	RNA binding protein	Regulation of nucleobase, nucleoside, nucleotide and nucleic acid metabolism	Nucleus; Cytoplasm	Single-stranded nucleic acid binding protein that binds preferentially to oligo dC. Major cellular poly(rC)-binding protein. Binds also poly(rU). Negatively regulates cellular antiviral responses mediated by MAVS signaling.
PABPC4	Polyadenylate-binding protein 4		9.31	69	85	0.8	73.25	-0.443	RNA binding protein	Regulation of nucleobase, nucleoside, nucleotide and nucleic acid metabolism	Cytoplasm	Binds the poly(A) tail of mRNA. May be involved in cytoplasmic regulatory processes of mRNA metabolism. Can probably bind to cytoplasmic RNA sequences other than poly(A) in vivo By similarity.
**PIGR**	**Polymeric immunoglobulin receptor**	**[2]**	6.34	65	63	1.0	80.77	-0.355	Cell surface receptor	Immune response	Plasma membrane; Extracellular	This receptor binds polymeric IgA and IgM at the basolateral surface of epithelial cells. The complex is then transported across the cell to be secreted at the apical surface. During this process a cleavage occurs that separates the extracellular (known as the secretory component) from the transmembrane segment.
GALNT18	Polypeptide N-acetylgalactosaminyltransferase 18		6.07	79	71	1.1	90.07	-0.339	Glycosyltransferase; Transferase	Protein glycosylation	Integral to membrane	Catalyzes the initial reaction in O-linked oligosaccharide biosynthesis, the transfer of an N-acetyl-D-galactosamine residue to a serine or threonine residue on the protein receptor.
PTBP1	Polypyrimidine tract-binding protein 1		9.22	42	51	0.8	90.77	-0.150	Ribonucleoprotein	Regulation of nucleobase, nucleoside, nucleotide and nucleic acid metabolism	Nucleus; Cytoplasm; Nucleolus	Plays a role in pre-mRNA splicing and in the regulation of alternative splicing events. Activates exon skipping of its own pre-mRNA during muscle cell differentiation. Binds to the polypyrimidine tract of introns. May promote RNA looping when bound to two separate polypyrimidine tracts in the same pre-mRNA.
**UBB**	**Polyubiquitin-B**	**[3]**	6.56	11	11	1.0	100.00	-0.489	Ubiquitin proteasome system protein	Protein metabolism	Nucleus; Nucleolus	Polyubiquitin chains, when attached to a target protein, have different functions depending on the Lys residue of the ubiquitin that is linked: DNA repair; ERAD (endoplasmic reticulum-associated degradation) and in cell-cycle regulation; lysosomal degradation; kinase modification; protein degradation via the proteasome; endocytosis. When polyubiquitin is free (unanchored-polyubiquitin), it also has distinct roles, such as in activation of protein kinases, and in signaling.
**KCNK7**	**Potassium channel subfamily K member 7**	**[1]**	6.70	17	16	1.1	119.32	0.625	**Ion channelIntegral membrane protein**	**Transport**	**Integral to membrane**	**Probable potassium channel subunit. No channel activity observed in vitro as protein remains in the endoplasmic reticulum. May need to associate with an as yet unknown partner in order to reach the plasma membrane.**
**LMNA**	**Prelamin-A/C**	**[1]**	6.57	107	104	1.0	74.45	-0.875	Structural protein	Cell growth and/or maintenance	Nucleus; Cytoplasm; Nucleolus	Lamins are components of the nuclear lamina, a fibrous layer on the nucleoplasmic side of the inner nuclear membrane, which is thought to provide a framework for the nuclear envelope and may also interact with chromatin. Lamin A and C are present in equal amounts in the lamina of mammals. Plays an important role in nuclear assembly, chromatin organization, nuclear membrane and telomere dynamics.
PCYOX1	Prenylcysteine oxidase 1		5.89	55	47	1.2	91.59	-0.132	Enzyme: Oxidase	Metabolism; Energy pathways	Lysosome	Involved in the degradation of prenylated proteins. Cleaves the thioether bond of prenyl-L-cysteines, such as farnesylcysteine and geranylgeranylcysteine.

DDX17	Probable ATP-dependent RNA helicase DDX17		8.53	84	90	0.9	65.88	-0.615	RNA helicaseATPase	Regulation of nucleobase, nucleoside, nucleotide and nucleic acid metabolism	Nucleus; Nucleolus	RNA-dependent ATPase activity. Involved in transcriptional regulation. Transcriptional coactivator for estrogen receptor ESR1. Increases ESR1 AF-1 domain-mediated transactivation. Synergizes with DDX5 and SRA1 RNA to activate MYOD1 transcriptional activity and probably involved in skeletal muscle differentiation. Required for zinc-finger antiviral protein ZC3HAV1-mediated mRNA degradation.
**DDX53**	**Probable ATP-dependent RNA helicase DDX53**	[1]	9.16	75	88	0.9	85.88	-0.485	RNA helicaseATPase	Regulation of nucleobase, nucleoside, nucleotide and nucleic acid metabolism	Nucleus	Contains several domains found in members of the DEAD-box helicase protein family. Other members of this protein family participate in ATP-dependent RNA unwinding.
CPVL	Probable serine carboxypeptidase CPVL		5.39	53	42	1.3	81.78	-0.283	Carboxypeptidase	Protein metabolism	Extracellular	May be involved in the digestion of phagocytosed particles in the lysosome, participation in an inflammatory protease cascade, and trimming of peptides for antigen presentation.
**EGF**	**Pro-epidermal growth factor**	[1-4, 8, 11]	4.78	9	5	1.8	71.70	-0.425	Growth factor	Cell communication; Signal transduction	Plasma membrane; Nucleus	EGF stimulates the growth of various epidermal and epithelial tissues in vivo and in vitro and of some fibroblasts in cell culture. Magnesiotropic hormone that stimulates magnesium reabsorption in the renal distal convoluted tubule via engagement of EGFR and activation of the magnesium channel TRPM6.
**FLG**	**Profilaggrin**	[10]	9.24	478	497	1.0	19.76	-1.673	Calcium binding protein	Cell communication; Signal transduction	Nucleus; Cytoplasm	Aggregates keratin intermediate filaments and promotes disulfide-bond formation among the intermediate filaments during terminal differentiation of mammalian epidermis.
**PFN1**	**Profilin 1**	[1]	8.47	13	15	0.9	82.01	-0.131	Cytoskeletal associated protein	Cell growth and/or maintenance	Cytoplasm; Extracellular	Binds to actin and affects the structure of the cytoskeleton. At high concentrations, profilin prevents the polymerization of actin, whereas it enhances it at low concentrations. By binding to PIP2, it inhibits the formation of IP3 and DG. Inhibits androgen receptor (AR) and HTT aggregation and binding of G-actin is essential for
PFN2	Profilin 2		6.78	15	15	1.0	75.04	-0.040	Structural protein	Cell growth and/or maintenance	Cytoplasm	Binds to actin and affects the structure of the cytoskeleton. At high concentrations, profilin prevents the polymerization of actin, whereas it enhances it at low concentrations. By binding to PIP2, it inhibits the formation of IP3 and DG.
PDCD6IP	Programmed cell death 6-interacting protein		6.14	103	97	1.1	80.07	-0.471	Calcium-dependent protein binding	Apoptosis	Cytoplasm; Perinuclear region; Cytoskeleton	Class E VPS protein involved in concentration and sorting of cargo proteins of the multivesicular body (MVB) for incorporation into intralumenal vesicles (ILVs) that are generated by invagination and scission from the limiting membrane of the endosome. Binds to the phospholipid lysobisphosphatidic acid (LBPA) which is abundant in MVBs internal membranes. Required for completion of cytokinesis. Involved in HIV-1 virus budding. May play a role in the regulation of both apoptosis and cell
**HBEGF**	**Proheparin-binding EGF-like growth factor**	[3]	9.34	22	32	0.7	89.15	-0.483	Growth factor	Cell communication; Signal transduction	Plasma membrane; Extracellular; Cytoplasm; Nucleus; Cell surface	Growth factor that mediates its effects via EGFR, ERBB2 and ERBB4. Required for normal cardiac valve formation and normal heart function. Promotes smooth muscle cell proliferation. May be involved in macrophage-mediated cellular proliferation. It is mitogenic for fibroblasts, but not endothelial cells. It is able to bind EGF receptor/EGFR with higher affinity than EGF itself and is a far more potent mitogen for smooth muscle cells than EGF. Also acts as a diphtheria toxin receptor.
PHB	Prohibitin		5.57	35	31	1.1	106.88	0.024	Adapter molecule	Cell communication; Signal transduction	Mitochondrion; Plasma membrane; Nucleus; Nucleolus; Extracellular; Cytoplasm	Prohibitin inhibits DNA synthesis. It has a role in regulating proliferation. As yet it is unclear if the protein or the mRNA exhibits this effect. May play a role in regulating mitochondrial respiration activity and in aging.
PIP	Prolactin-inducible protein		5.40	15	14	1.1	102.37	-0.035	Secreted polypeptide	Actin-binding	Extracellular	The protein functions in regulation of water transport mainly in apocrine glands in the axilla, vulva, eyelid and ear canal, serous cells of the submandibular salivary gland, serous cells of the submucosal glands of the bronchi, and accessory lacrimal glands as well as cutaneous eccrine glands.[4] It is also found in amniotic fluid and seminal fluid.
PREP	Prolyl endopeptidase		5.53	94	73	1.3	81.25	-0.328	Serine protease	Protein metabolism	Cytoplasm; Mitochondrion; Nucleus	Cleaves peptide bonds on the C-terminal side of prolyl residues within peptides that are up to approximately 30 amino acids long.
**PSAP**	**Prosaposin, Isoform Sap-mu-6**	[1, 3]	5.03	73	51	1.4	90.16	-0.146	Integral membrane protein	Cell communication; Signal transduction	Lysosome; Extracellular; Plasma membrane	Saposins are specific low-molecular mass non-enzymic proteins, they participate in the lysosomal degradation of sphingolipids, which takes place by the sequential action of specific hydrolases.. Saposin-A and saposin-C stimulate the hydrolysis of glucosylceramide by beta-glucosylceramidase (EC 3.2.1.45) and galactosylceramide by beta-galactosylceramidase (EC 3.2.1.46). Saposin-C apparently acts by combining with the enzyme and acidic lipid to form an activated complex, rather than by solubilizing the substrate.
**PTGES3**	**Prostaglandin E synthase 3**	[3]	4.32	40	20	2.0	51.75	-1.049	Chaperone	Protein metabolism	Cytoplasm; Nucleus	Molecular chaperone that localizes to genomic response elements in a hormone-dependent manner and disrupts receptor-mediated transcriptional activation, by promoting disassembly of transcriptional regulatory complexes.
PTGR1	Prostaglandin reductase 1		8.45	36	39	0.9	91.82	-0.027	Enzyme: Dehydrogenase	Metabolism; Energy pathways	Cytoplasm	Functions as 15-oxo-prostaglandin 13-reductase and acts on 15-oxo-PGE1, 15-oxo-PGE2 and 15-oxo-PGE2-alpha. Has no activity towards PGE1, PGE2 and PGE2-alpha By similarity. Catalyzes the conversion of leukotriene B4 into its biologically less active metabolite, 12-oxo-leukotriene B4.
PTGDS	Prostaglandin-H2 D-isomerase		8.37	16	18	0.9	59.29	-0.482	Enzyme: Ligase	Metabolism; Energy pathways	Extracellular; Endoplasmic reticulum	Catalyzes the conversion of PGH2 to PGD2, a prostaglandin involved in smooth muscle contraction/relaxation and a potent inhibitor of platelet aggregation. Binds small non-substrate lipophilic molecules, including biliverdin, bilirubin, retinal, retinoic acid and thyroid hormone, and may act as a scavenger for harmful hydrophopic molecules and as a secretory retinoid and thyroid hormone transporter. Possibly involved in development and maintenance of the blood-brain, blood-retina, blood-aqueous humor and blood-testis barrier.
**PSCA**	**Prostate stem cell antigen**	[1]	4.52	9	6	1.5	85.73	-0.060	Integral membrane protein	Cell communication; Signal transduction	Plasma membrane	May be involved in the regulation of cell proliferation. Has a cell-proliferation inhibition activity in vitro.
PSME2	Proteasome activator complex subunit 2		5.54	39	33	1.2	96.26	-0.364	Ubiquitin proteasome system protein	Protein metabolism	Cytoplasm; Nucleus; Endoplasmic reticulum; Nucleolus	Implicated in immunoproteasome assembly and required for efficient antigen processing. The PA28 activator complex enhances the generation of class I binding peptides by altering the cleavage pattern of the proteasome.
PSMA2	Proteasome subunit alpha type-2		7.12	25	25	1.0	87.08	-0.195	Ubiquitin proteasome system protein	Protein metabolism	Cytoplasm; Nucleus; Proteasome	The proteasome is a multicatalytic proteinase complex which is characterized by its ability to cleave peptides with Arg, Phe, Tyr, Leu, and Glu adjacent to the leaving group at neutral or slightly basic pH. The proteasome has an ATP-dependent proteolytic activity. PSMA2 may have a potential regulatory effect on another component(s) of the proteasome complex through tyrosine phosphorylation.
PSMA3	Proteasome subunit alpha type-3		5.19	40	31	1.3	82.56	-0.287	Ubiquitin proteasome system protein	Protein metabolism	Cytoplasm; Nucleus	The proteasome is a multicatalytic proteinase complex which is characterized by its ability to cleave peptides with Arg, Phe, Tyr, Leu, and Glu adjacent to the leaving group at neutral or slightly basic pH. The proteasome has an ATP-dependent proteolytic activity. Binds to the C-terminus of CDKN1A and thereby mediates its degradation. Negatively regulates the membrane trafficking of the cell-surface thromboxane A2 receptor (TBXA2R) isoform 2.
**PSMA5**	**Proteasome subunit alpha type-5**	[3]	4.74	35	20	1.8	87.84	-0.107	Ubiquitin proteasome system protein	Protein metabolism	Cytoplasm; Nucleus	The proteasome is a multicatalytic proteinase complex which is characterized by its ability to cleave peptides with Arg, Phe, Tyr, Leu, and Glu adjacent to the leaving group at neutral or slightly basic pH. The proteasome has an ATP-dependent proteolytic activity.
PSMB5	Proteasome subunit beta type-5		8.66	20	23	0.9	78.97	-0.200	Ubiquitin proteasome system protein	Protein metabolism; Proteolysis and peptidolysis	Nucleus; Cytoplasm; Cytosol	he proteasome is a multicatalytic proteinase complex which is characterized by its ability to cleave peptides with Arg, Phe, Tyr, Leu, and Glu adjacent to the leaving group at neutral or slightly basic pH. The proteasome has an ATP-dependent proteolytic activity. This unit is responsible of the chymotrypsin-like activity of the proteasome and is one of the principal target of the proteasome inhibitor bortezomib. May catalyze basal processing of intracellular antigens.
**AMBP**	**Protein AMBP (Alpha-1-Microglobulin/Bikunin Precursor)**	[1-3, 9, 11]	5.76	40	35	1.1	64.38	-0.421	Secreted polypeptide	Immune response	Extracellular; Golgi apparatus; Endoplasmic reticulum; Cytoplasm; Plasma membrane	Inter-alpha-trypsin inhibitor inhibits trypsin, plasmin, and lysosomal granulocytic elastase. Inhibits calcium oxalate crystallization.
**DEK**	**Protein DEK**	[3]	8.69	76	82	0.9	55.53	-1.294	DNA binding protein	Regulation of nucleobase, nucleoside, nucleotide and nucleic acid metabolism	Nucleus; Cytoplasm	Involved in chromatin organization.
P4HB	Protein disulfide-isomerase		4.69	99	60	1.7	76.92	-0.501	Enzyme: Isomerase	Protein metabolism	Endoplasmic reticulum; Nucleus; Extracellular; Nucleolus; Zymogen granule; Mitochondrion	his multifunctional protein catalyzes the formation, breakage and rearrangement of disulfide bonds. At the cell surface, seems to act as a reductase that cleaves disulfide bonds of proteins attached to the cell. May therefore cause structural modifications of exofacial proteins. Inside the cell, seems to form/rearrange disulfide bonds of nascent proteins. At high concentrations, functions as a chaperone that inhibits aggregation of misfolded proteins. At low concentrations, facilitates aggregation (anti-chaperone activity).
PDIA3	Protein disulfide-isomerase A3		5.61	77	67	1.1	71.60	-0.594	Enzyme: Isomerase	Protein metabolism	Endoplasmic reticulum; Cytoplasm; Plasma membrane; Mitochondrion	Catalyzes the rearrangement of -S-S- bonds in proteins.
**PDIA4**	**Protein disulfide-isomerase A4**	[3]	4.89	117	85	1.4	74.54	-0.606	Chaperone	Protein metabolism	Endoplasmic reticulum; Plasma membrane; Mitochondrion	Catalyzes the rearrangement of -S-S- bonds in proteins.
LMAN1	Protein ERGIC-53		5.75	65	52	1.3	72.29	-0.590	Chaperone	Protein metabolism	Endoplasmic reticulum; Golgi apparatus	Mannose-specific lectin. May recognize sugar residues of glycoproteins, glycolipids, or glycosylphosphatidyl inositol anchors and may be involved in the sorting or recycling of proteins, lipids, or both. The LMAN1-MCFD2 complex forms a specific cargo receptor for the ER-to-Golgi transport of selected proteins.
**FAM178A**	**Protein FAM178A**	[1]	9.09	151	175	0.9	73.90	-0.751	Unclassified	Unknown	Nucleus	??
**MCM10**	**Protein MCM10 homolog**	[1]	8.96	125	144	0.9	70.93	-0.765	DNA binding protein	Regulation of nucleobase, nucleoside, nucleotide and nucleic acid metabolism	Nucleus; Nucleolus; Cytoplasm	Acts as a replication initiation factor that brings together the MCM2-7 helicase and the DNA polymerase alpha/primase complex in order to initiate DNA replication. Additionally, plays a role in preventing DNA damage during replication.
PPP1R7	Protein phosphatase 1 regulatory subunit 7		4.84	68	46	1.5	106.43	-0.564	Serine/threonine phosphatase	Regulation of nucleobase, nucleoside, nucleotide and nucleic acid metabolism	Nucleus; Cytoplasm	Regulatory subunit of protein phosphatase 1.
SSH3	Protein phosphatase Slingshot homolog 3		5.19	89	62	1.4	77.52	-0.585	Dual specificity phosphatase	Cell growth and/or maintenance	Cytoplasm; Nucleus	Protein phosphatase which may play a role in the regulation of actin filament dynamics. Can dephosphorylate and activate the actin binding/depolymerizing factor cofilin, which subsequently binds to actin filaments and stimulates their disassembly By similarity.
RCC2	Protein RCC2		9.02	55	67	0.8	69.94	-0.420	Cell cycle control protein	Cell growth and/or maintenance	Nucleus; Cytoplasm; Mitochondrion	Required for completion of mitosis and cytokinesis. May function as a guanine nucleotide exchange factor for the small GTPase RAC1.
**S100A11**	**Protein S100-A11 (Calgizzarin)**	[1]	6.82	13	13	1.0	72.31	-0.362	Calcium binding protein	Cell communication; Signal transduction	Nucleus; Cytoplasm; Plasma membrane; Endosome	Facilitates the differentiation and the cornification of keratinocytes.
**S100A12**	**Protein S100-A12 (Calgranulin-C)**	[1, 4-5, 8]	5.80	15	11	1.4	100.77	-0.425	Calcium binding protein	Cell communication; Signal transduction	Cytoplasm	S100A12 is a calcium-, zinc- and copper-binding protein which plays a prominent role in the regulation of inflammatory processes and immune response. Its proinflammatory activity involves recruitment of leukocytes, promotion of cytokine and chemokine production, and regulation of leukocyte adhesion and migration. Acts as an alarmin or a danger associated molecular pattern (DAMP) molecule and stimulates innate immune cells via binding to receptor for advanced glycation endproducts (AGER). Acts as a monocyte and mast cell chemoattractant. Can stimulate mast cell degranulation and activation which generates chemokines, histamine and cytokines inducing further leukocyte recruitment to the sites of inflammation.
S100A2	Protein S100-A2 (S100 calcium-binding protein A2)		4.68	17	10	1.7	76.53	-0.257	Calcium binding protein	Cell communication; Signal transduction	Nucleus; Cytoplasm	May function as calcium sensor and modulator, contributing to cellular calcium signaling. May function by interacting with other proteins, such as TPR-containing proteins, and indirectly play a role in many physiological processes. May also play a role in suppressing tumor cell growth.
**S100A6**	**Protein S100-A6 (Calcyclin)**	[1]	5.32	15	12	1.3	105.22	-0.289	Calcium binding protein	Cell communication; Signal transduction	Nucleus; Cytoplasm	May function as calcium sensor and modulator, contributing to cellular calcium signaling. May function by interacting with other proteins, such as TPR-containing proteins, and indirectly play a role in many physiological processes such as the reorganization of the actin cytoskeleton and in cell motility.
**S100A7**	**Protein S100-A7 (Psoriasin)**	[9-10]	6.26	16	15	1.1	59.60	-0.777	Calcium binding protein	Cell communication; Signal transduction	Cytoplasm; Endoplasmic reticulum; Nucleus; Plasma membrane; Extracellular	S100 proteins are localized in the cytoplasm and/or nucleus of a wide range of cells, and involved in the regulation of a number of cellular processes such as cell cycle progression and differentiation. This protein is markedly over-expressed in the skin lesions of psoriatic patients, but is excluded as a candidate gene for familial psoriasis susceptibility. The exact function of this protein is not known.
**S100A8**	**Protein S100-A8 (Calgranulin-A)**	[1-12]	6.57	15	14	1.1	97.50	-0.422	Calcium binding protein	Cell communication; Signal transduction	Cytoplasm; Extracellular; Plasma membrane	It can induce neutrophil chemotaxis and adhesion. Predominantly found as calprotectin (S100A8/A9) which has a wide plethora of intra- and extracellular functions. The intracellular functions include: facilitating leukocyte arachidonic acid trafficking and metabolism, modulation of the tubulin-dependent cytoskeleton during migration of phagocytes and activation of the neutrophilic NADPH-oxidase. Its role as an oxidant scavenger has a protective role in preventing exaggerated tissue damage by scavenging oxidants. Can act as a potent amplifier of inflammation in autoimmunity as well as in cancer development and tumor spread.
**S100A9**	**Protein S100-A9 (Calgranulin-B)**	[1-12]	5.71	20	14	1.4	69.03	-0.895	Calcium binding protein	Cell communication; Signal transduction	Cytoplasm; Extracellular; Plasma membrane	It can induce neutrophil chemotaxis and adhesion. Predominantly found as calprotectin (S100A8/A9) which has a wide plethora of intra- and extracellular functions. The intracellular functions include: facilitating leukocyte arachidonic acid trafficking and metabolism, modulation of the tubulin-dependent cytoskeleton during migration of phagocytes and activation of the neutrophilic NADPH-oxidase. Its role as an oxidant scavenger has a protective role in preventing exaggerated tissue damage by scavenging oxidants. Can act as a potent amplifier of inflammation in autoimmunity as well as in cancer development and tumor spread.
**S100P**	**Protein S100-P (Migration-inducing gene 9 protein)**	[1, 8]	4.75	16	11	1.5	84.21	-0.120	Calcium binding protein	Cell communication; Signal transduction	Cytoplasm; Plasma membrane	May function as calcium sensor and contribute to cellular calcium signaling. In a calcium-dependent manner, functions by interacting with other proteins, such as EZR and PPP5C, and indirectly plays a role in physiological processes like the formation of microvilli in epithelial cells. May stimulate cell proliferation in an autocrine manner via activation of the receptor for activated glycation end products (RAGE).

SAV1	Protein salvador homolog 1		9.12	43	50	0.9	61.36	-0.930	Transcription regulatory protein	Regulation of nucleobase, nucleoside, nucleotide and nucleic acid metabolism	Nucleus	Regulator of STK3/MST2 and STK4/MST1 in the Hippo signaling pathway which plays a pivotal role in organ size control and tumor suppression by restricting proliferation and promoting apoptosis. In conjunction with STK3/MST2, activates the transcriptional activity of ESR1 through the modulation of its phosphorylation.
**SET**	**Protein SET**	[1, 3-4]	4.22	86	33	2.6	54.67	-1.345	MHC complex protein	Regulation of nucleobase, nucleoside, nucleotide and nucleic acid metabolism	Cytoplasm; Endoplasmic reticulum; Nucleus; Plasma membrane; Cytosol; Nuclear membrane	Multitasking protein, involved in apoptosis, transcription, nucleosome assembly and histone chaperoning.
SETSIP	Protein SETSIP		4.19	89	34	2.6	54.87	-1.277	Chromatin binding	Differentiation, Transcription, Transcription regulation	Cytoplasm; Nucleus	Plays a role as a transcriptional activator involved in the early stage of somatic cell reprogramming. Promotes the differentiation of protein-induced pluripotent stem (PiPS) cells into endothelial cells and the formation of vascular-like tubes (in vitro). Involved in the transcription induction of vascular endothelial-cadherin (VE-cadherin) expression. Associates to the VE-cadherin gene promoter.
**SHROOM3**	**Protein Shroom3**	[1]	7.87	242	245	1.0	62.32	-0.802				
**PTPRCAP**	**Protein tyrosine phosphatase receptor type C-associated protein**	[4]	4.39	29	13	2.2	91.60	-0.136	**Unclassified**	**Immune response**	**Plasma membrane; Mitochondrion**	**Transmembrane phosphoprotein specifically associated with tyrosine phosphatase PTPRC/CD45, a key regulator of T- and B-lymphocyte activation. The interaction with PTPRC may be required for the stable expression of this protein.**
**WNT7A**	**Protein Wnt-7a**	[3]	8.83	32	45	0.7	62.92	-0.494	**Ligand**	**Cell communication; Signal transduction**	**Extracellular**	**Ligand for members of the frizzled family of seven transmembrane receptors. Probable developmental protein. Signaling by Wnt-7a allows sexually dimorphic development of the mullerian ducts (By similarity).**
SERPINA10	Protein Z-dependent protease inhibitor		7.86	53	54	1.0	81.13	-0.323	**Protease inhibitor**	**Protein metabolism**	**Extracellular**	**Inhibits activity of the coagulation protease factor Xa in the presence of PROZ, calcium and phospholipids. Also inhibits factor XIa in the absence of cofactors.**
**TGM2**	**Protein-glutamine gamma-glutamyltransferase 2**	[10]	5.11	96	71	1.4	87.92	-0.286	Enzyme: Aminotransferase	Metabolism; Energy pathways	Extracellular; Plasma membrane; Cytoplasm; Nucleus	Catalyzes the cross-linking of proteins and the conjugation of polyamines to proteins.
**PTK2B**	**Protein-tyrosine kinase 2-beta**	[3]	5.90	141	127	1.1	88.38	-0.421	**Tyrosine kinase**	**Signal transduction**	**Cytoplasm; Nucleus; Nucleoplasm; Plasma membrane; Focal adhesion; Perinuclear region**	**Non-receptor protein-tyrosine kinase that regulates reorganization of the actin cytoskeleton, cell polarization, cell migration, adhesion, spreading and bone remodeling. Plays a role in the regulation of the humoral immune response, and is required for normal levels of marginal B-cells in the spleen and normal migration of splenic B-cells. Required for normal macrophage polarization and migration towards sites of inflammation. Regulates cytoskeleton rearrangement and cell spreading in T-cells, and contributes to the regulation of T-cell responses.**
PTK6	Protein-tyrosine kinase 6		6.56	59	55	1.1	85.19	-0.368	**Tyrosine kinase**	**Cell communication; Signal transduction**	**Cytoplasm; Nucleus**	**Non-receptor tyrosine-protein kinase implicated in the regulation of a variety of signaling pathways that control the differentiation and maintenance of normal epithelia, as well as tumor growth. Function seems to be context dependent and differ depending on cell type, as well as its intracellular localization. Associates also with a variety of proteins that are likely upstream of PTK6 in various signaling pathways, or for which PTK6 may play an**
**PRG3**	**Proteoglycan 3**	[4]	4.69	32	19	1.7	63.80	-0.563	Unclassified	Immune response	Extracellular	Possesses similar cytotoxic and cytostimulatory activities to PRG2/MBP. In vitro, stimulates neutrophil superoxide production and IL8 release, and histamine and leukotriene C4 release from basophils.
**F2**	**Prothrombin**	[1-4, 7-12]	5.23	86	68	1.3	65.85	-0.606	Coagulation factor	Protein metabolism	Extracellular	Thrombin, which cleaves bonds after Arg and Lys, converts fibrinogen to fibrin and activates factors V, VII, VIII, XIII, and, in complex with thrombomodulin, protein C. Functions in blood homeostasis, inflammation and wound healing.
**PTMA**	**Prothymosin alpha**	[1, 4]	3.66	54	10	5.4	30.27	-2.006	Unclassified	Cell proliferation	Nucleus; Cytoplasm; Perinuclear region	Prothymosin alpha may mediate immune function by conferring resistance to certain opportunistic infections.
PCDHB10	Protocadherin beta-10		4.70	102	63	1.6	93.59	-0.159	Adhesion molecule	Cell communication; Signal transduction	Plasma membrane	Potential calcium-dependent cell-adhesion protein. May be involved in the establishment and maintenance of specific neuronal connections in the brain.
PCDHGA3	Protocadherin gamma-A3		4.83	116	77	1.5	94.37	-0.241	Adhesion molecule	Cell growth and/or maintenance	Integral to membrane	Potential calcium-dependent cell-adhesion protein. May be involved in the establishment and maintenance of specific neuronal connections in the brain.
PCDHGC3	Protocadherin gamma-C3		5.01	110	80	1.4	86.91	-0.296	Adhesion molecule	Cell communication; Signal transduction	Plasma membrane	Potential calcium-dependent cell-adhesion protein. May be involved in the establishment and maintenance of specific neuronal connections in the brain.
SRC	Proto-oncogene tyrosine-protein kinase Src		7.22	63	63	1.0	71.87	-0.477	Tyrosine kinase	Signal transduction	Nucleus; Plasma membrane; Cytoplasm	Non-receptor protein tyrosine kinase which is activated following engagement of many different classes of cellular receptors including immune response receptors, integrins and other adhesion receptors, receptor protein tyrosine kinases, G protein-coupled receptors as well as cytokine receptors. Participates in signaling pathways that control a diverse spectrum of biological activities including gene transcription, immune response, cell adhesion, cell cycle progression, apoptosis, migration, and
PNP	Purine nucleoside phosphorylase		6.45	31	29	1.1	81.97	-0.166	**Enzyme: Phosphorylase**	**Metabolism; Energy pathways**	**Cytoplasm**	**The purine nucleoside phosphorylases catalyze the phosphorolytic breakdown of the N-glycosidic bond in the beta-(deoxy)ribonucleoside molecules, with the formation of the corresponding free purine bases and pentose-1-phosphate.**
AHCYL1	Putative adenosylhomocysteinase 2		6.48	65	63	1.0	82.57	-0.269	Enzyme: Hydrolase	Metabolism; Energy pathways	Cytoplasm; Endoplasmic reticulum; Plasma membrane	Interacts with inositol 1,4,5-trisphosphate receptor, type 1 and may be involved in the conversion of S-adenosyl-L-homocysteine to L-homocysteine and adenosine.
**DUX2**	**Putative double homeobox protein 2**	[1]	10.73	6	12	0.5	72.00	-0.776	**Transcription regulatory protein**	**Regulation of nucleobase, nucleoside, nucleotide and nucleic acid metabolism**	**Nucleus**	**The human genome contains hundreds of repeats of the 3.3-kb family in regions associated with heterochromatin. The DUX gene family, including DUX2, resides within these 3.3-kb repeated elements.**
HSPA7	Putative heat shock 70 kDa protein 7		7.72	47	48	1.0	78.45	-0.391	Heat shock protein	Protein metabolism	Extracellular	Response to stress.
HSP90AB4P	Putative heat shock protein HSP 90-beta 4		4.65	109	63	1.7	77.94	-0.671	**Chaperone**	**Protein metabolism**	**Cytoplasm**	**Putative molecular chaperone that may promote the maturation, structural maintenance and proper regulation of specific target proteins.**
**DHX15**	**Putative pre-mRNA-splicing factor ATP-dependent RNA helicase DHX15**	[3]	7.12	109	108	1.0	81.94	-0.514	RNA binding protein	Regulation of nucleobase, nucleoside, nucleotide and nucleic acid metabolism	Nucleus; Nucleolus	Pre-mRNA processing factor involved in disassembly of spliceosomes after the release of mature mRNA.
ST13P5	Putative protein FAM10A5		4.96	71	51	1.4	63.82	-0.826	Unclassified	Unclassified	Cytoplasm	Suppresses tumorigenicity in colon carcinoma and interacts with Hsp70.
LUC7L2	Putative RNA-binding protein Luc7-like 2		10.02	75	102	0.7	52.55	-1.486	**Unclassified**	**Unclassified**	**Nucleolus; Nucleus; Cytoplasm**	**May bind to RNA via its Arg/Ser-rich domain.**
PDXK	Pyridoxal kinase		5.75	39	32	1.2	95.80	-0.207	**Enzyme: Phosphotransferase**	**Metabolism; Energy pathways**	**Cytoplasm**	**Required for synthesis of pyridoxal-5-phosphate from vitamin B6.**
PDXDC1	Pyridoxal-dependent decarboxylase domain-containing protein 1		5.25	109	82	1.3	92.42	-0.278	Enzyme: Decarboxylase	Metabolism; Energy pathways	Nucleus; Cytoplasm; Golgi apparatus	Exhibits carboxy-lyase activity (inferred); pyridoxal phosphate binding (inferred); amd is involved in carboxylic acid metabolic processes.
PKM	Pyruvate kinase PKM		7.95	66	68	1.0	91.47	-0.132	Enzyme: Phosphotransferase	Metabolism; Energy pathways	Cytoplasm; Perinuclear region; Extracellular; Cytosol	Glycolytic enzyme that catalyzes the transfer of a phosphoryl group from phosphoenolpyruvate (PEP) to ADP, generating ATP. Stimulates POU5F1-mediated transcriptional activation. Plays a general role in caspase independent cell death of tumor cells. The ratio betwween the highly active tetrameric form and nearly inactive dimeric form determines whether glucose carbons are channeled to biosynthetic processes or used for glycolytic ATP production.
GDI2	Rab GDP dissociation inhibitor beta		6.10	62	59	1.1	87.15	-0.332	Membrane transport protein	Transport	Cytoplasm; Plasma membrane; Endoplasmic reticulum;	Regulates the GDP/GTP exchange reaction of most Rab proteins by inhibiting the dissociation of GDP from them, and the subsequent binding of GTP to them.
**RDX**	**Radixin**	[7]	6.03	111	103	1.1	73.52	-1.007	Cytoskeletal associated protein	Cell growth and/or maintenance	Plasma membrane; Cytoplasm	Probably plays a crucial role in the binding of the barbed end of actin filaments to the plasma membrane.
RANGAP1	Ran GTPase-activating protein 1		4.63	102	60	1.7	91.98	-0.245	GTPase activating protein	Cell communication; Signal transduction	Cytoplasm; Nucleus; Kinetochore; Microtubule	GTPase activator for the nuclear Ras-related regulatory protein Ran, converting it to the putatively inactive GDP-bound state.
G3BP1	Ras GTPase-activating protein-binding protein 1		5.36	700	56	12.5	64.88	-0.840	RNA binding protein; Ribonuclease	Regulation of nucleobase, nucleoside, nucleotide and nucleic acid metabolism; Signal transduction	Cytosol; Nucleus; Plasma membrane; Cytoplasm	May be a regulated effector of stress granule assembly. Phosphorylation-dependent sequence-specific endoribonuclease in vitro.
IQGAP1	Ras GTPase-activating-like protein IQGAP1		6.08	235	217	1.1	91.97	-0.496	GTPase activating protein	Cytoskeleton organization and biogenesis	Cytoplasm; Plasma membrane; Golgi apparatus; Mitochondrion	Binds to activated CDC42 but does not stimulate its GTPase activity. It associates with calmodulin. Could serve as an assembly scaffold for the organization of a multimolecular complex that would interface incoming signals to the reorganization of the actin cytoskeleton at the plasma membrane. May promote neurite outgrowth.
**RAPH1**	**Ras-associated and pleckstrin homology domains-containing protein 1**	[3]	8.97	117	133	0.9	66.62	-0.650	Cytoskeletal associated protein	Cell growth and/or maintenance	Cell projection	Mediator of localized membrane signals. Implicated in the regulation of lamellipodial dynamics. Negatively regulates cell adhesion.
RAC1	Ras-related C3 botulinum toxin substrate 1, Isoform B		8.77	21	26	0.8	90.74	-0.162	GTPase	Cell communication; Signal transduction	Cytoplasm; Cell projection; Plasma membrane; Membrane fraction; Zymogen granule	Plasma membrane-associated small GTPase which cycles between active GTP-bound and inactive GDP-bound states. In its active state, binds to a variety of effector proteins to regulate cellular responses such as secretory processes, phagocytosis of apoptotic cells, epithelial cell polarization and growth-factor induced formation of membrane ruffles. Essential for the SPATA13-mediated regulation of cell migration and adhesion assembly and disassembly. Stimulates PKN2 kinase activity.In podocytes, promotes nuclear shuttling of NR3C2; this modulation is required for a proper kidney functioning.
RAC2	Ras-related C3 botulinum toxin substrate 2		7.52	22	23	1.0	91.80	-0.159	GTPase	Cell communication; Signal transduction	Plasma membrane	Plasma membrane-associated small GTPase which cycles between an active GTP-bound and inactive GDP-bound state. In active state binds to a variety of effector proteins to regulate cellular responses, such as secretory processes, phagocytose of apoptotic cells and epithelial cell polarization. Augments the production of reactive oxygen species (ROS) by NADPH oxidase.
RAB10	Ras-related protein Rab-10		8.58	27	30	0.9	83.85	-0.330	GTPase	Cell communication; Signal transduction	Nucleus; Cytoplasm; Golgi apparatus; Endosome; Golgi vesicle; Mitochondrion	The small GTPases Rab are key regulators of intracellular membrane trafficking, from the formation of transport vesicles to their fusion with membranes. Rabs cycle between an inactive GDP-bound form and an active GTP-bound form that is able to recruit to membranes different set of downstream effectors directly responsible for vesicle formation, movement, tethering and fusion. That Rab is mainly involved in the biosynthetic transport of proteins from the Golgi to the plasma membrane. Plays also a specific role in asymmetric protein transport to the plasma membrane within the polarized neuron and epithelial cells.Moreover, may play a role in the basolateral recycling pathway and in phagosome maturation.
RAB11A	Ras-related protein Rab-11A		6.14	29	27	1.1	85.99	-0.426	GTPase	Cell communication; Signal transduction	Golgi apparatus; Mitochondrion	Regulates endocytic recycling. Acts as a major regulator of membrane delivery during cytokinesis. Together with MYO5B and RAB8A participates in epithelial cell polarization.
**RAB13**	**Ras-related protein Rab-13**	[3]	9.27	26	34	0.8	79.55	-0.501	GTPase	Cell communication; Signal transduction	Cytoplasmic vesicle; Plasma membrane	The small GTPases Rab are key regulators of intracellular membrane trafficking, from the formation of transport vesicles to their fusion with membranes. Rabs cycle between an inactive GDP-bound form and an active GTP-bound form that is able to recruit to membranes different sets of downstream effectors directly responsible for vesicle formation, movement, tethering and fusion. That Rab is involved in endocytic recycling and regulates the transport to the plasma membrane of transmembrane proteins like the tight junction protein OCLN/occludin. Thereby, it regulates the assembly and the activity of tight junctions. Moreover, it may also regulate tight junction assembly by activating the PKA signaling pathway and by reorganizing the actin cytoskeleton through the activation of the downstream effectors PRKACA and
**RAB15**	**Ras-related protein Rab-15**	[1]	5.53	33	28	1.2	79.58	-0.559	GTPase	Cell communication; Signal transduction	Endosome	May act in concert with RAB3A in regulating aspects of synaptic vesicle membrane flow within the nerve terminal.
**RAB1A**	**Ras-related protein Rab-1A**	[1]	5.93	25	24	1.0	80.29	-0.304	GTPase	Cell communication; Signal transduction	Endoplasmic reticulum; Golgi apparatus	RAB1A regulates vesicular protein transport from the endoplasmic reticulum (ER) to the Golgi compartment and on to the cell surface, and plays a role in IL-8 and growth hormone secretion. Regulates the level of CASR present at the cell membrane. Plays a role in cell adhesion and cell migration, via its role in protein trafficking. Plays a role in autophagosome assembly and cellular defense reactions against pathogenic bacteria. Plays a role in microtubule-dependent protein transport by early endosomes and in anterograde melanosome transport.
**RAB33B**	**Ras-related protein Rab-33B**	[1]	6.70	27	26	1.0	77.55	-0.262	**GTPase**	**Cell communication; Signal transduction**	**Golgi apparatus; Mitochondrion**	**Protein transport. Acts, in coordination with RAB6A, to regulate intra-Golgi retrograde trafficking. It is involved in autophagy, acting as a modulator of autophagosome formation.**
**RAB6A**	**Ras-related protein Rab-6A**	[1]	5.42	30	28	1.1	80.00	-0.427	GTPase	Cell communication; Signal transduction	Plasma membrane; Cytoplasmic vesicle; Golgi apparatus	Protein transport. Regulator of membrane traffic from the Golgi apparatus towards the endoplasmic reticulum (ER). Has a low GTPase activity.
RAB7A	Ras-related protein Rab-7a		6.39	27	27	1.0	78.21	-0.377	GTPase	Cell communication; Signal transduction	Endosome; Lysosome; Mitochondrion	Key regulator in endo-lysosomal trafficking. Governs early-to-late endosomal maturation, microtubule minus-end as well as plus-end directed endosomal migration and positioning, and endosome-lysosome transport through different protein-protein interaction cascades. Plays a central role, not only in endosomal traffic, but also in many other cellular and physiological events, such as growth-factor-mediated cell signaling, nutrient-transportor mediated nutrient uptake, neurotrophin transport in the axons of neurons and lipid metabolism. Also involved in regulation of some specialized endosomal membrane trafficking, such as maturation of melanosomes, pathogen-induced phagosomes (or vacuoles) and autophagosomes.
RAP1A	Ras-related protein Rap-1A		6.39	26	26	1.0	83.37	-0.444	GTPase	Cell communication; Signal transduction	Nucleus; Plasma membrane; Late endosome; Lysosome; Perinuclear region; Cytoplasm; Mitochondrion	Induces morphological reversion of a cell line transformed by a Ras oncogene. Plays a role in nerve growth factor (NGF)-induced neurite outgrowth. Plays a role in the regulation of embryonic blood vessel formation. Involved in the establishment of basal endothelial barrier function. May be involved in the regulation of the vascular endothelial growth factor receptor KDR expression at endothelial cell-cell junctions.

RAP1B	Ras-related protein Rap-1b		5.65	25	24	1.0	84.48	-0.419	GTPase	Cell communication; Signal transduction	Nucleus; Cytoplasm; Plasma membrane; Cell projection; Zymogen granule	GTP-binding protein that possesses intrinsic GTPase activity. Contributes to the polarizing activity of KRIT1 and CDH5 in the establishment and maintenance of correct endothelial cell polarity and vascular lumen. Required for the localization of phosphorylated PRKCZ, PARD3 and TIAM1 to the cell junction. Plays a role in the establishment of basal endothelial barrier function.
**RGS22**	**Regulator of G-protein signaling 22**	**[3]**	8.08	170	175	1.0	79.14	-0.547	GTPase activating protein	Cell communication; Signal transduction	Nucleus; Plasma membrane; Cytoplasm	Inhibits signal transduction by increasing the GTPase activity of G protein alpha subunits thereby driving them into their inactive GDP-bound form.
**RGS9**	**Regulator of G-protein signaling 9**	**[1]**	9.42	71	97	0.7	69.90	-0.588	**GTPase activating protein**	**Cell communication; Signal transduction**	**Plasma membrane**	**Inhibits signal transduction by increasing the GTPase activity of G protein alpha subunits thereby driving them into their inactive GDP-bound form. Binds to G(t)-alpha. Involved in phototransduction; key element in the recovery phase of visual transduction (By similarity).**
**ATP6AP2**	**Renin receptor**	**[1]**	5.75	37	32	1.2	102.69	-0.027	Cell surface receptor	Cell communication; Signal transduction	Plasma membrane	Functions as a renin and prorenin cellular receptor. May mediate renin-dependent cellular responses by activating ERK1 and ERK2. By increasing the catalytic efficiency of renin in AGT/angiotensinogen conversion to angiotensin I, it may also play a role in the renin-angiotensin system (RAS).
**RETN**	**Resistin**	**[9]**	5.72	9	8	1.1	65.11	0.040	Peptide hormone	Cell communication; Signal transduction	Extracellular; Cytoplasm	Hormone that seems to suppress insulin ability to stimulate glucose uptake into adipose cells. Potentially links obesity to diabetes.
SCPEP1	Retinoid-inducible serine carboxypeptidase		5.36	48	40	1.2	85.12	-0.127	Carboxypeptidase	Protein metabolism	Cytoplasm; Extracellular	May be involved in vascular wall and kidney homeostasis.
RBP4	Retinol binding protein 4, plasma		5.27	28	24	1.2	66.07	-0.588	**Transport/cargo protein**	**Transport**	**Extracellular**	**Delivers retinol from the liver stores to the peripheral tissues. In plasma, the RBP-retinol complex interacts with transthyretin, this prevents its loss by filtration through the kidney glomeruli.**
ARHGDIB	Rho GDP-dissociation inhibitor 2		5.08	39	29	1.3	73.50	-0.799	Adapter molecule	Cell communication; Signal transduction	Cytoplasm; Mitochondrion	Regulates the GDP/GTP exchange reaction of the Rho proteins by inhibiting the dissociation of GDP from them, and the subsequent binding of GTP to them.
ARHGEF10L	Rho guanine nucleotide exchange factor 10-like protein		5.59	162	132	1.2	88.75	-0.261	**Guanine nucleotide exchange factor**	**Cell communication; Signal transduction**	**Cytoplasm**	**Acts as guanine nucleotide exchange factor (GEF) for RHOA, RHOB and RHOC.**
RHOB	Rho-related GTP-binding protein RhoB		5.00	32	25	1.3	85.80	-0.285	GTPase	Cell communication; Signal transduction	Plasma membrane; Golgi apparatus	Mediates apoptosis in neoplastically transformed cells after DNA damage. Not essential for development but affects cell adhesion and growth factor signaling in transformed cells. Plays a negative role in tumorigenesis as deletion causes tumor formation. Involved in intracellular protein trafficking of a number of proteins. Targets PKN1 to endosomes and is involved in trafficking of the EGF receptor from late endosomes to lysosomes.
RHOC	Rho-related GTP-binding protein RhoC		6.20	31	30	1.0	82.05	-0.463	GTPase	Cell communication; Signal transduction	Cytoplasm; Plasma membrane	Regulates a signal transduction pathway linking plasma membrane receptors to the assembly of focal adhesions and actin stress fibers. Serves as a microtubule-dependent signal that is required for the myosin contractile ring formation during cell cycle cytokinesis. Regulates apical junction formation in bronchial epithelial cells.
ROBO4	Roundabout homolog 4		6.19	92	82	1.1	77.49	-0.367	Adhesion molecule	Cell communication; Signal transduction	Plasma membrane	Receptor for Slit proteins, at least for SLIT2, and seems to be involved in angiogenesis and vascular patterning. May mediate the inhibition of primary endothelial cell migration by Slit proteins By similarity.
RUVBL2	RuvB-like 2		5.49	69	60	1.2	92.68	-0.235	Transcription regulatory protein	Regulation of nucleobase, nucleoside, nucleotide and nucleic acid metabolism	Cytoplasm; Nucleus; Nucleolus; Chromosome	Component of the NuA4 histone acetyltransferase complex which is involved in transcriptional activation of select genes principally by acetylation of nucleosomal histones H4 and H2A. This modification may both alter nucleosome - DNA interactions and promote interaction of the modified histones with other proteins which positively regulate transcription.
ATP2A2	Sarcoplasmic/endoplasmic reticulum calcium ATPase 2		5.23	125	100	1.3	97.49	0.097	Membrane transport protein	Transport	Endoplasmic reticulum; Cytoplasm; Mitochondrion; Nucleus	This magnesium-dependent enzyme catalyzes the hydrolysis of ATP coupled with the translocation of calcium from the cytosol to the sarcoplasmic reticulum lumen. Isoform 2 is involved in the regulation of the contraction/relaxation cycle.
ATP2A3	Sarcoplasmic/endoplasmic reticulum calcium ATPase 3		5.41	121	100	1.2	96.77	0.077	ATPase	Ion transport	Endoplasmic reticulum; Sarcoplasmic reticulum; Nuclear membrane; Mitochondrion	This magnesium-dependent enzyme catalyzes the hydrolysis of ATP coupled with the transport of calcium. Transports calcium ions from the cytosol into the sarcoplasmic/endoplasmic reticulum lumen. Contributes to calcium sequestration involved in muscular excitation/contraction.
**SAFB**	**Scaffold attachment factor B1**	**[3]**	5.32	199	162	1.2	47.10	-1.349	**Transcription factor**	**Regulation of nucleobase, nucleoside, nucleotide and nucleic acid metabolism**	**Nucleus; Cytoplasm**	**Binds to scaffold/matrix attachment region (S/MAR) DNA and forms a molecular assembly point to allow the formation of a 'transcriptosomal' complex (consisting of SR proteins and RNA polymerase II) coupling transcription and RNA processing (By similarity). Can function as an estrogen receptor corepressor and can also bind to the HSP27 promoter and decrease its transcription. When associated with RBMX, binds to and stimulates transcription from the SREBF1 promoter (By similarity). Can inhibit cell proliferation.**
**SECTM1**	**Secreted and transmembrane protein 1**	**[1]**	7.13	18	18	1.0	84.68	-0.103	Integral membrane protein	Cell communication; Signal transduction	Golgi apparatus; Extracellular; Plasma membrane	May be involved in thymocyte signaling.
**SPP2**	**Secreted phosphoprotein 24**	**[3-4, 12]**	7.85	22	23	1.0	67.97	-0.564	**Protease inhibitor**	**Protein metabolism**	**Extracellular**	**Could coordinate an aspect of bone turnover.**
CHGB	Secretogranin-1		5.02	155	102	1.5	38.95	-1.645	Secreted polypeptide	Cell communication; Signal transduction	Extracellular; Cytoplasm; Endoplasmic reticulum; Nucleus	Secretogranin-1 is a neuroendocrine secretory granule protein, which may be the precursor for other biologically active peptides.
**SEMG1**	**Semenogelin-1**	**[4]**	9.26	50	60	0.8	48.61	-1.480	**Structural protein**	**Cell growth and/or maintenance**	**Extracellular; Cytoplasm**	**Predominant protein in semen. It participates in the formation of a gel matrix entrapping the accessory gland secretions and ejaculated spermatozoa. Fragments of semenogelin and/or fragments of the related proteins may contribute to the activation of progressive sperm movements as the gel-forming proteins are fragmented by KLK3/PSA.**
SEPT2	Septin-2		6.15	56	50	1.1	86.09	-0.528	GTPase	Cell communication; Signal transduction; Cell cycle	Cytoplasm; Plasma membrane; Nucleolus; Microtubule; Mitochondrion; Nucleus	Filament-forming cytoskeletal GTPase. Required for normal organization of the actin cytoskeleton. Plays a role in the biogenesis of polarized columnar-shaped epithelium by maintaining polyglutamylated microtubules, thus facilitating efficient vesicle transport, and by impeding MAP4 binding to tubulin. Plays a role in ciliogenesis and collective cell movements.
SEPT9	Septin-9		9.06	79	88	0.9	73.40	-0.663	GTPase	Cell proliferation	Cytoskeleton; Nucleus; Microtubule	Filament-forming cytoskeletal GTPase By similarity. May play a role in cytokinesis Potential. May play a role in the internalization of 2 intracellular microbial pathogens, Listeria monocytogenes and Shigella flexneri.
SPTLC1	Serine palmitoyltransferase 1		5.72	62	53	1.2	101.06	-0.064	Enzyme: Palmitoyltransferase	Metabolism; Energy pathways	Endoplasmic reticulum	Serine palmitoyltransferase (SPT). The heterodimer formed with SPTLC2 or SPTLC3 constitutes the catalytic core. The composition of the serine palmitoyltransferase (SPT) complex determines the substrate preference.
SRSF1	Serine/arginine-rich splicing factor 1		10.37	31	51	0.6	44.53	-1.164	RNA binding protein	Protein metabolism	Nucleus; Cytoplasm; Nucleolus	Plays a role in preventing exon skipping, ensuring the accuracy of splicing and regulating alternative splicing. May function as export adapter involved in mRNA nuclear export through the TAP/NXF1 pathway.
SRSF10	Serine/arginine-rich splicing factor 10		11.26	28	72	0.4	26.79	-1.762	RNA binding protein	RNA metabolism	Nucleolus; Nucleus; Cytoplasm; Nucleoplasm	Splicing factor that in its dephosphorylated form acts as a general repressor of pre-mRNA splicing. Seems to interfere with the U1 snRNP 5'-splice recognition of SNRNP70. Required for splicing repression in M-phase cells and after heat shock. May be involved in regulation of alternative splicing in neurons, with isoform 1 acting as a positive and isoform 3 as a negative regulator.
SRSF11	Serine/arginine-rich splicing factor 11		10.52	66	114	0.6	54.33	-1.246	Transcription regulatory protein	Regulation of nucleobase, nucleoside, nucleotide and nucleic acid metabolism	Nucleus; Cytoplasm	May function in pre-mRNA splicing.
**SRSF3**	**Serine/arginine-rich splicing factor 3**	**[3-4]**	11.64	19	45	0.4	39.21	-1.521	RNA binding protein	Regulation of nucleobase, nucleoside, nucleotide and nucleic acid metabolism	Nucleus; Cytoplasm; Nucleolus; Mitochondrion	May be involved in RNA processing in relation with cellular proliferation and/or maturation. May function as export adapter involved in mRNA nuclear export such as of histone H2A.
**SRSF6**	**Serine/arginine-rich splicing factor 6**	**[3]**	11.42	39	95	0.4	39.65	-1.550	RNA binding protein	Regulation of nucleobase, nucleoside, nucleotide and nucleic acid metabolism	Nucleus; Nucleolus; Cytoplasm	Plays a role in constitutive splicing and modulates the selection of alternative splice sites. Plays a role in the alternative splicing of MAPT/Tau exon 10. Binds to alternative exons of TNC pre-mRNA and promotes the expression of alternatively spliced TNC. Plays a role in wound healing and in the regulation of keratinocyte differentiation and proliferation via its role in alternative splicing.
**SRSF7**	**Serine/arginine-rich splicing factor 7**	**[4]**	11.83	19	65	0.3	34.03	-1.397	RNA binding protein	Regulation of nucleobase, nucleoside, nucleotide and nucleic acid metabolism	Nucleus; Cytoplasm	Required for pre-mRNA splicing. Can also modulate alternative splicing in vitro. Represses the splicing of MAPT/Tau exon 10. May function as export adapter involved in mRNA nuclear export such as of histone H2A. Binds mRNA which is thought to be transferred to the NXF1-NXT1 heterodimer for export (TAP/NXF1 pathway); enhances NXF1-NXT1 RNA-binding activity.
SRSF8	Serine/arginine-rich splicing factor 8		11.72	21	74	0.3	24.98	-1.594	**Transcription regulatory protein**	**Regulation of nucleobase, nucleoside, nucleotide and nucleic acid metabolism**	**Nucleus**	**Involved in pre-mRNA alternative splicing.**
**SRSF2**	**Serine/arginine-rich-splicing factor 2**	**[4]**	11.86	21	65	0.3	28.77	-1.640	Ribonucleoprotein	Regulation of nucleobase, nucleoside, nucleotide and nucleic acid metabolism	Nucleus; Nucleolus	Necessary for the splicing of pre-mRNA. It is required for formation of the earliest ATP-dependent splicing complex and interacts with spliceosomal components bound to both the 5'- and 3'-splice sites during spliceosome assembly.The phosphorylated form (by SRPK2) is required for cellular apoptosis in response to cisplatin treatment.
STK24	Serine/threonine-protein kinase 24 12 kDa subunit		5.49	67	56	1.2	86.37	-0.491	Serine/threonine kinase	Signal transduction	Cytoplasm; Nucleus	Serine/threonine-protein kinase that acts on both serine and threonine residues and promotes apoptosis in response to stress stimuli and caspase activation. Mediates oxidative-stress-induced cell death by modulating phosphorylation of JNK1-JNK2 (MAPK8 and MAPK9), p38 (MAPK11, MAPK12, MAPK13 and MAPK14) during oxidative stress. May act as a key regulator of axon regeneration in the optic nerve and radial nerve.
MST4	Serine/threonine-protein kinase MST4		5.16	65	51	1.3	81.85	-0.463	Serine/threonine kinase	Cell communication; Signal transduction	Cytoplasm; Nucleus; Golgi apparatus	Mediator of cell growth. Modulates apoptosis.
SIK3	Serine/threonine-protein kinase SIK3		6.22	127	104	1.2	74.48	-0.565	Serine/threonine kinase	Cell communication; Signal transduction	Nucleus; Cytosol	ATP + a protein = ADP + a phosphoprotein.
PPP1CB	Serine/threonine-protein phosphatase		5.85	44	40	1.1	85.83	-0.249	Serine/threonine phosphatase	Cell growth and/or maintenance	Nucleolus; Microtubule	Protein phosphatase that associates with over 200 regulatory proteins to form highly specific holoenzymes which dephosphorylate hundreds of biological targets. Protein phosphatase (PP1) is essential for cell division, it participates in the regulation of glycogen metabolism, muscle contractility and protein synthesis. Involved in regulation of ionic conductances and long-term synaptic plasticity. Component of the PTW/PP1 phosphatase complex, which plays a role in the control of chromatin structure and cell cycle progression during the transition from mitosis into interphase.
PPP2R2A	Serine/threonine-protein phosphatase 2A 55 kDa regulatory subunit B alpha		5.82	67	58	1.2	73.65	-0.616	Serine/threonine kinase	Cell communication; Signal transduction	Cytoplasm; Chromosome	The B regulatory subunit might modulate substrate selectivity and catalytic activity, and also might direct the localization of the catalytic enzyme to a particular subcellular compartment.
PPP2R2D	Serine/threonine-protein phosphatase 2A 55 kDa regulatory subunit B delta isoform		5.96	68	59	1.2	72.76	-0.569	Protein phosphatase type 2A regulator	Cell cycle, Cell division, Mitosis	Cytoplasm	B regulatory subunit of protein phosphatase 2A (PP2A) that plays a key role in cell cycle by controlling mitosis entry and exit. The activity of PP2A complexes containing PPP2R2D (PR55-delta) fluctuate during the cell cycle: the activity is high in interphase and low in mitosis.
PPP2R1A	Serine/threonine-protein phosphatase 2A 65 kDa regulatory subunit A alpha isoform		5.00	83	59	1.4	109.63	0.072	Serine/threonine phosphatase	Cell communication; Signal transduction	Cytoplasm; Chromosome; Membrane fraction; Microtubule	The PR65 subunit of protein phosphatase 2A serves as a scaffolding molecule to coordinate the assembly of the catalytic subunit and a variable regulatory B subunit. Required for proper chromosome segregation and for centromeric localization of SGOL1 in mitosis.
PPP2CA	Serine/threonine-protein phosphatase 2A catalytic subunit alpha isoform		5.30	44	32	1.4	79.13	-0.427	Serine/threonine phosphatase	Cell communication; Signal transduction	Cytoplasm; Chromosome; Mitochondrion	PP2A is the major phosphatase for microtubule-associated proteins (MAPs). PP2A can modulate the activity of phosphorylase B kinase casein kinase 2, mitogen-stimulated S6 kinase, and MAP-2 kinase. Cooperates with SGOL2 to protect centromeric cohesin from separase-mediated cleavage in oocytes specifically during
PPP2CB	Serine/threonine-protein phosphatase 2A catalytic subunit beta isoform		5.21	44	31	1.4	78.19	-0.446	**Serine/threonine phosphatase**	**Cell communication; Signal transduction**	**Cytoplasm; Nucleus**	**PP2A can modulate the activity of phosphorylase B kinase casein kinase 2, mitogen-stimulated S6 kinase, and MAP-2 kinase.**
PPP1CA	Serine/threonine-protein phosphatase PP1-alpha catalytic subunit		5.94	44	40	1.1	85.96	-0.308	Serine/threonine phosphatase	Cell proliferation	Cytoskeleton; Nucleolus; Microtubule; Mitochondrion; Cytoplasm	Protein phosphatase that associates with over 200 regulatory proteins to form highly specific holoenzymes which dephosphorylate hundreds of biological targets. Protein phosphatase (PP1) is essential for cell division, it participates in the regulation of glycogen metabolism, muscle contractility and protein synthesis. Involved in regulation of ionic conductances and long-term synaptic plasticity. Component of the PTW/PP1 phosphatase complex, which plays a role in the control of chromatin structure and cell cycle progression during the transition from mitosis into interphase.
PPP1CB	Serine/threonine-protein phosphatase PP1-beta catalytic subunit		5.85	44	40	1.1	85.83	-0.249	Serine/threonine phosphatase	Cell growth and/or maintenance	Nucleolus; Microtubule	Protein phosphatase that associates with over 200 regulatory proteins to form highly specific holoenzymes which dephosphorylate hundreds of biological targets. Protein phosphatase (PP1) is essential for cell division, it participates in the regulation of glycogen metabolism, muscle contractility and protein synthesis. Involved in regulation of ionic conductances and long-term synaptic plasticity. Component of the PTW/PP1 phosphatase complex, which plays a role in the control of chromatin structure and cell cycle progression during the transition from mitosis into interphase.
**TF**	**Serotransferrin**	**[1, 5-6, 8-9]**	6.70	87	84	1.0	70.27	-0.411	**Transport/cargo protein**	**Transport**	**Endosome; Cytoplasm; Plasma membrane; Nucleus; Endoplasmic reticulum; Extracellular**	**Transferrins are iron binding transport proteins which can bind two Fe3+ ions in association with the binding of an anion, usually bicarbonate. It is responsible for the transport of iron from sites of absorption and heme degradation to those of storage and utilization. Serum transferrin may also have a further role in stimulating cell proliferation.**

SERPINA5	Serpin A5 (Plasma serine protease inhibitor)	[11]	9.17	37	43	0.9	89.79	-0.156	Protease inhibitor	Protein metabolism	Extracellular; Plasma membrane; Nucleus	Heparin-dependent serine protease inhibitor acting in body fluids and secretions. Inactivates serine proteases by binding irreversibly to their serine activation site. Involved in the regulation of intravascular and extravascular proteolytic activities. Plays hemostatic roles in the blood plasma. Acts as a procoagulant and proinflammatory factor by inhibiting the anticoagulant activated protein C factor as well as the generation of activated protein C factor by the thrombin/thrombomodulin complex. Acts as an anticoagulant factor by inhibiting blood coagulation factors like prothrombin, factor XI, factor Xa, plasma kallikrein and fibrinolytic enzymes such as tissue- and urinary-type plasminogen activators.
SERPINB12	Serpin B12	[10]	5.36	56	44	1.3	81.36	-0.350	Protease inhibitor	Protein metabolism	Cytoplasm; Extracellular	Inhibits trypsin and plasmin, but not thrombin, coagulation factor Xa, or urokinase-type plasminogen activator.
SERPINB13	Serpin B13 (hurpin)	[10, 11]	5.48	56	43	1.3	76.55	-0.370	Protease inhibitor	Protein metabolism	Cytoplasm; Extracellular	May play a role in the proliferation or differentiation of keratinocytes.
SERPINB3	Serpin B3	[2, 10]	6.35	50	47	1.1	76.26	-0.461	Protease inhibitor	Protein metabolism	Cytoplasm; Extracellular	May act as a papain-like cysteine protease inhibitor to modulate the host immune response against tumor cells. Also functions as an inhibitor of UV-induced apoptosis via suppression of the activity of c-Jun NH(2)-terminal kinase (JNK1).
SERPINB4	Serpin B4	[2, 11]	5.86	51	45	1.1	75.49	-0.460	Protease inhibitor	Protein metabolism	Cytoplasm	May act as a protease inhibitor to modulate the host immune response against tumor cells.
SERPINB6	Serpin B6	[1]	5.18	53	42	1.3	75.98	-0.221	Protease inhibitor	Protein metabolism	Cytoplasm	May be involved in the regulation of serine proteinases present in the brain or extravasated from the blood (By similarity). Inhibitor of cathepsin G, kallikrein-8 and thrombin. May play an important role in the inner ear in the protection against leakage of lysosomal content during stress and loss of this protection results in cell death and sensorineural hearing loss.
SERPINB7	Serpin B7	[10]	6.34	47	44	1.1	81.87	-0.312	Protease inhibitor	Protein metabolism	Cytoplasm	Might function as an inhibitor of Lys-specific proteases. Might influence the maturation of megakaryocytes via its action as a serpin.
SERPINB8	Serpin B8	[10]	5.41	58	48	1.2	74.60	-0.324	Protease inhibitor	Protein metabolism	Nucleuas; Cytoplasm; Extracellular	Member of the ov-serpin subfamily, which, relative to the archetypal serpin PI1, is characterized by a high degree of homology to chicken ovalbumin, lack of N- and C-terminal
SERPINH1	Serpin H1		8.81	50	54	0.9	87.58	-0.313	Heat shock protein	Protein metabolism	Endoplasmic reticulum; Plasma membrane; Nucleolus	Binds specifically to collagen. Could be involved as a chaperone in the biosynthetic pathway of collagen.
ALB	Serum albumin	[1-12]	5.67	98	83	1.2	76.92	-0.395	Transport/cargo protein	Transport	Extracellular	Serum albumin, the main protein of plasma, has a good binding capacity for water, Ca2+, Na+, K+, fatty acids, hormones, bilirubin and drugs. Its main function is the regulation of the colloidal osmotic pressure of blood. Major zinc transporter in plasma, typically binds about 80% of all plasma zinc.
APCS	Serum amyloid P-component	[1, 3-5, 8-10]	6.12	22	20	1.1	92.60	-0.185	Secreted polypeptide	Protein metabolism	Extracellular; Nucleus; Cytoplasm	Can interact with DNA and histones and may scavenge nuclear material released from damaged circulating cells. May also function as a calcium-dependent lectin.
PON1	Serum paraoxonase/arylesterase 1	[1]	5.08	43	26	1.7	98.81	-0.097	Enzyme: Esterase	Metabolism; Energy pathways	Plasma membrane; Extracellular	Hydrolyzes the toxic metabolites of a variety of organophosphorus insecticides. Capable of hydrolyzing a broad spectrum of organophosphate substrates and lactones, and a number of aromatic carboxylic acid esters. Mediates an enzymatic protection of low density lipoproteins against oxidative modification and the consequent series of events leading to atheroma formation.
SIAE	Sialate O-acetylesterase		7.00	43	43	1.0	78.20	-0.221	Enzyme: Esterase	Metabolism; Energy pathways	Cytoplasm	Catalyzes the removal of O-acetyl ester groups from position 9 of the parent sialic acid, N-acetylneuraminic acid.
SIGLEC16	Sialic acid-binding Ig-like lectin 16	[1]	9.29	40	54	0.7	81.51	-0.264	Unclassified	Cell adhesion	Plasma membrane	Putative adhesion molecule that mediates sialic-acid dependent binding to cells.
NEU1	Sialidase-1		5.15	42	31	1.4	79.67	-0.228	Enzyme: Hydrolase	Metabolism; Energy pathways	Lysosome; Plasma membrane; Endoplasmic reticulum	Catalyzes the removal of sialic acid (N-acetylneuraminic acid) moities from glycoproteins and glycolipids. To be active, it is strictly dependent on its presence in the multienzyme complex. Appears to have a preference for alpha 2-3 and alpha 2-6 sialyl linkage.
SRP68	Signal recognition particle subunit SRP68		8.75	84	92	0.9	83.13	-0.553	RNA binding protein	Protein metabolism	Nucleolus; Cytoplasm; Endoplasmic reticulum; Cytosol	Signal-recognition-particle assembly has a crucial role in targeting secretory proteins to the rough endoplasmic reticulum membrane. SRP68 binds the 7S RNA, SRP72 binds to this complex subsequently. This ribonucleoprotein complex might interact directly with the docking protein in the ER membrane and possibly participate in the elongation arrest function.
STAT1	Signal transducer and activator of transcription 1-alpha/beta		5.74	103	89	1.2	87.16	-0.526	Transcription factor	Regulation of nucleobase, nucleoside, nucleotide and nucleic acid metabolism	Cytoplasm; Nucleus	Signal transducer and transcription activator that mediates cellular responses to interferons (IFNs), cytokine KITLG/SCF and other cytokines and other growth factors. Following type I IFN (IFN-alpha and IFN-beta) binding to cell surface receptors, signaling via protein kinases leads to activation of Jak kinases (TYK2 and JAK1) and to tyrosine phosphorylation of STAT1 and STAT2.
STAT6	Signal transducer and activator of transcription 6		5.84	94	79	1.2	88.40	-0.269	Transcription factor	Regulation of nucleobase, nucleoside, nucleotide and nucleic acid metabolism	Cytoplasm; Nucleus	Carries out a dual function: signal transduction and activation of transcription. Involved in IL4/interleukin-4- and IL3/interleukin-3-mediated signaling.
SIPA1L1	Signal-induced proliferation-associated 1-like protein 1	[4]	8.40	218	227	1.0	65.14	-0.672	GTPase activating protein	Cell communication; Signal transduction	Nucleus	Stimulates the GTPase activity of RAP2A. Promotes reorganization of the actin cytoskeleton and recruits DLG4 to F-actin. Contributes to the regulation of dendritic spine morphogenesis (By similarity).
SKOR1	SKI family transcriptional corepressor 1		6.11	106	96	1.1	60.62	-0.523	Transcription regulatory protein	Cell fate commitment	Nucleus	Acts as a transcriptional corepressor of LBX1 By similarity. Inhibits BMP signaling.
CD84	SLAM family member 5		7.06	33	33	1.0	81.51	-0.331	Receptor	Immune response	Plasma membrane	Plays a role as adhesion receptor functioning by homophilic interactions and by clustering. Recruits SH2 domain-containing proteins SH2D1A/SAP. Increases proliferative responses of activated T-cells and SH2D1A/SAP does not seen be required for this process. Homophilic interactions enhance interferon gamma/IFNG secretion in lymphocytes and induce platelet stimulation via a SH2D1A/SAP-dependent pathway. May serve as a marker for hematopoietic progenitor cells.
NALCN	Sodium leak channel non-selective protein	[1]	8.93	169	197	0.9	98.15	0.089	Voltage gated channel	Transport	Plasma membrane	Voltage-independent, cation-nonselective channel which is permeable to sodium, potassium and calcium ions. Responsible for the background sodium ion leak current in neurons and controls neuronal excitability. Activated either by neuropeptides substance P or neurotensin.
SLC9C1	Sodium/hydrogen exchanger 10		6.72	122	118	1.0	112.14	0.255	Membrane transport protein	Transport	Cell membrane, Cell projection, Cilium, Flagellum, Membrane	Sperm-specific sodium/hydrogen exchanger involved in intracellular pH regulation of spermatozoa. Required for sperm motility and fertility. Involved in sperm cell hyperactivation, a step needed for sperm motility which is essential late in the preparation of sperm for fertilization. Required for the expression and bicarbonate regulation of the soluble adenylyl cyclase (sAC) By similarity.
ATP1A1	Sodium/potassium-transporting ATPase subunit alpha-1		5.29	125	101	1.2	97.61	0.010	ATPase	Transport	Plasma membrane; Mitochondrion	This is the catalytic component of the active enzyme, which catalyzes the hydrolysis of ATP coupled with the exchange of sodium and potassium ions across the plasma membrane. This action creates the electrochemical gradient of sodium and potassium ions, providing the energy for active transport of various nutrients.
ATP1A1	Sodium/potassium-transporting ATPase subunit alpha-1, Isoform 3		5.29	125	101	1.2	97.61	0.010	ATPase	Transport	Plasma membrane; Mitochondrion	This is the catalytic component of the active enzyme, which catalyzes the hydrolysis of ATP coupled with the exchange of sodium and potassium ions across the plasma membrane. This action creates the electrochemical gradient of sodium and potassium ions, providing the energy for active transport of various nutrients.
ATP1A2	Sodium/potassium-transporting ATPase subunit alpha-2		5.43	123	105	1.2	95.11	-0.013	ATPase	Transport	Plasma membrane	This is the catalytic component of the active enzyme, which catalyzes the hydrolysis of ATP coupled with the exchange of sodium and potassium ions across the plasma membrane. This action creates the electrochemical gradient of sodium and potassium, providing the energy for active transport of various nutrients.
SNX25	Sorting nexin-25	[10]	5.99	124	112	1.1	91.68	-0.467	Transport/cargo protein	Cell communication; Signal transduction	Nucleus	May be involved in several stages of intracellular trafficking.
SPTAN1	Spectrin alpha chain, non-erythrocytic 1		5.22	460	347	1.3	79.15	-0.790	Cytoskeletal protein; Structural protein	Cell growth and/or maintenance	Cytoplasm; Plasma membrane; Cytosol; Nucleus	Fodrin, which seems to be involved in secretion, interacts with calmodulin in a calcium-dependent manner and is thus candidate for the calcium-dependent movement of the cytoskeleton at the membrane.
SPTBN1	Spectrin beta chain, non-erythrocytic 1		5.39	426	336	1.3	81.08	-0.767	Cytoskeletal protein	Cell growth and/or maintenance	Cytoplasm; Plasma membrane; Cytosol; Nucleus	Fodrin, which seems to be involved in secretion, interacts with calmodulin in a calcium-dependent manner and is thus candidate for the calcium-dependent movement of the cytoskeleton at the membrane.
U2AF2	Splicing factor U2AF 65 kDa subunit	[9]	9.19	58	68	0.9	72.34	-0.594	RNA binding protein	RNA metabolism	Nucleus; Cytoplasm; Nucleolus	Necessary for the splicing of pre-mRNA. Induces cardiac troponin-T (TNNT2) pre-mRNA exon inclusion in muscle. Regulates the TNNT2 exon 5 inclusion through competition with MBNL1. Binds preferentially to a single-stranded structure within the polypyrimidine tract of TNNT2 intron 4 during spliceosome assembly. Required for the export of mRNA out of the nucleus, even if the mRNA is encoded by an intron-less gene.
SFRS1	Splicing factor, arginine/serine-rich 1	[4]	10.37	31	51	0.6	44.53	-1.164	RNA binding protein	Protein metabolism	Nucleus; Cytoplasm; Nucleolus	Plays a role in preventing exon skipping, ensuring the accuracy of splicing and regulating alternative splicing. Interacts with other spliceosomal components, via the RS domains, to form a bridge between the 5'- and 3'-splice site binding components, U1 snRNP and U2AF.
SFRS2	Splicing factor, arginine/serine-rich 2	[4]	11.86	21	65	0.3	28.77	-1.640	Ribonucleoprotein	Regulation of nucleobase, nucleoside, nucleotide and nucleic acid metabolism	Nucleus; Nucleolus	Necessary for the splicing of pre-mRNA. It is required for formation of the earliest ATP-dependent splicing complex and interacts with spliceosomal components bound to both the 5'- and 3'-splice sites during spliceosome assembly. It also is required for ATP-dependent interactions of both U1 and U2 snRNPs with pre-mRNA.
SFRS3	Splicing factor, arginine/serine-rich 3	[4]	11.64	19	45	0.4	39.21	-1.521	RNA binding protein	Regulation of nucleobase, nucleoside, nucleotide and nucleic acid metabolism	Nucleus; Cytoplasm; Nucleolus; Mitochondrion	May be involved in RNA processing in relation with cellular proliferation and/or maturation. May function as export adapter involved in mRNA nuclear export such as of histone H2A. Binds mRNA which is thought to be transferred to the NXF1-NXT1 heterodimer for export (TAP/NXF1 pathway); enhances NXF1-NXT1 RNA-binding activity. RNA-binding is semi-sequence specific.
SFRS7	Splicing factor, arginine/serine-rich 7	[4]	11.83	19	65	0.3	34.03	-1.397	RNA binding protein	Regulation of nucleobase, nucleoside, nucleotide and nucleic acid metabolism	Nucleus; Cytoplasm	Required for pre-mRNA splicing. Can also modulate alternative splicing in vitro. Represses the splicing of MAPT/Tau exon 10. May function as export adapter involved in mRNA nuclear export such as of histone H2A. Binds mRNA which is thought to be transferred to the NXF1-NXT1 heterodimer for export (TAP/NXF1 pathway); enhances NXF1-NXT1 RNA-binding activity. RNA-binding is semi-sequence specific.
SREK1	Splicing regulatory glutamine/lysine-rich protein 1		10.39	91	146	0.6	40.91	-1.803	RNA binding protein	Regulation of nucleobase, nucleoside, nucleotide and nucleic acid metabolism	Nucleus	Participates in the regulation of alternative splicing by modulating the activity of other splice facors. Inhibits the splicing activity of SFRS1, SFRS2 and SFRS6.
SND1	Staphylococcal nuclease domain-containing protein 1		6.77	126	123	1.0	84.35	-0.428	Transcription regulatory protein	Regulation of nucleobase, nucleoside, nucleotide and nucleic acid metabolism	Nucleus	Functions as a bridging factor between STAT6 and the basal transcription factor. Plays a role in PIM1 regulation of MYB activity.
STK39	STE20/SPS1-related proline-alanine-rich protein kinase		5.92	72	65	1.1	85.80	-0.283	Serine/threonine kinase	Cell communication; Signal transduction	Cytoplasm; Nucleus	May act as a mediator of stress-activated signals.
STOML3	Stomatin-like protein 3	[1]	8.83	29	33	0.9	112.54	0.144	Integral membrane protein	Cell communication; Signal transduction	Plasma membrane	Required for the function of many mechanoreceptors. Modulate mechanotransduction channels and acid-sensing ion channels (ASIC) proteins. Potentiates PIEZO1 and PIEZO2 function by increasing their sensitivity to mechanical stimulations.
SDHA	Succinate dehydrogenase [ubiquinone] flavoprotein subunit, mitochondrial		6.25	73	65	1.1	78.86	-0.279	Enzyme: Dehydrogenase	Metabolism; Energy pathways	Mitochondrion; Cytosol	Flavoprotein (FP) subunit of succinate dehydrogenase (SDH) that is involved in complex II of the mitochondrial electron transport chain and is responsible for transferring electrons from succinate to ubiquinone (coenzyme Q). Can act as a tumor suppressor.
SUCLG1	Succinyl-CoA ligase [ADP/GDP-forming] subunit alpha, mitochondrial		8.77	25	30	0.8	87.03	-0.100	Enzyme: Ligase	Metabolism; Energy pathways	Mitochondrion	Catalyzes the ATP- or GTP-dependent ligation of succinate and CoA to form succinyl-CoA. The nature of the beta subunit determines the nucleotide specificity By similarity.
MPST	Sulfurtransferase		6.14	39	34	1.1	75.54	-0.433	Enzyme: Sulphotransferase	Metabolism; Energy pathways	Mitochondrion	Transfer of a sulfur ion to cyanide or to other thiol compounds. Also has weak rhodanese activity. Detoxifies cyanide and is required for thiosulfate biosynthesis. Acts as an antioxidant. In combination with cysteine aminotransferase (CAT), contributes to the catabolism of cysteine and is an important producer of hydrogen sulfide in the brain, retina and vascular endothelial cells.
SIMC1	SUMO-interacting motif-containing protein 1 (C5orf25)	[1]	6.10	95	83	1.1	80.80	-0.474				
SOD1	Superoxide dismutase [Cu-Zn]	[2-3, 12]	5.70	21	15	1.4	78.95	-0.358	Enzyme: Superoxide dismutase	Metabolism; Energy pathways	Peroxisome; Nucleus; Extracellular; Cytoplasm	Destroys radicals which are normally produced within the cells and which are toxic to biological systems.
SVIL	Supervillin	[1]	6.55	324	312	1.0	68.08	-0.728	Unclassified	Unknown	Unknown	SUMO polymer binding
SBSN	Suprabasin	[10]	6.45	62	48	1.3	55.75	-0.776	Unclassified	Unknown	Cytoplasm; Secreted	The suprabasin gene is a novel gene expressed in mouse and human differentiating keratinocytes, potentially plays a role in the process of epidermal differentiation.
SUSD2	Sushi domain-containing protein 2		5.84	75	58	1.3	70.05	-0.275	Integral membrane protein	Immune response	Integral to membrane	SUSD2 interacts with galectin-1 (Gal-1), a 14-kDa secreted protein that is synthesized by carcinoma cells and promotes tumor immune evasion, angiogenesis, and metastasis.
SYCP1	Synaptonemal complex protein 1	[3]	5.78	193	177	1.1	78.63	-1.032	DNA binding protein	Regulation of nucleobase, nucleoside, nucleotide and nucleic acid metabolism	Nucleus	Major component of the transverse filaments of synaptonemal complexes (SCS), formed between homologous chromosomes during meiotic prophase.
SYPL1	Synaptophysin-like protein 1	[1]	8.69	14	18	0.8	97.10	0.346	Integral membrane protein	Transport	Microsome; Plasma membrane; Cytoplasmic vesicle; Endoplasmic reticulum; Golgi vesicle; Cytoplasm; Golgi apparatus; Endosome	Synaptophysin I (SYPI), a major synaptic vesicle protein (Takamori et al., 2006), appears to be involved in determination of synaptic strength.

**SYNPO2**	**Synaptopodin-2**	**[1]**	8.75	121	131	0.9	63.92	-0.649	**Unclassified**	**Unknown**	**Cytoplasm; Nucleus**	**Has an actin-binding and actin-bundling activity.**
SDC1	Syndecan-1		4.45	43	18	2.4	59.41	-0.666	Cell surface receptor	Cell communication; Signal transduction; Cell surface receptor linked signal transduction; Cell adhesion	Plasma membrane	Cell surface proteoglycan that bears both heparan sulfate and chondroitin sulfate and that links the cytoskeleton to the interstitial matrix.
**SDC4**	**Syndecan-4**	**[3-4, 11]**	4.34	36	17	2.1	92.50	-0.337	Cell surface receptor	Cell communication; Signal transduction	Plasma membrane	Cell surface proteoglycan that bears heparan sulfate.
RIC8A	Synembryn-A		5.19	83	61	1.4	93.80	-0.265	Guanine nucleotide exchange factor	Cell communication; Signal transduction	Cytoplasm; Plasma membrane	Guanine nucleotide exchange factor (GEF), which can activate some, but not all, G-alpha proteins. Able to activate GNAI1, GNAO1 and GNAQ, but not GNAS by exchanging bound GDP for free GTP. Involved in regulation of microtubule pulling forces during mitotic movement of chromosomes by stimulating G(i)-alpha protein.
TLN1	Talin-1		5.77	302	267	1.1	86.99	-0.240	Cytoskeletal associated protein	Cell growth and/or maintenance	Extracellular; Plasma membrane; Cytoplasm; Nucleus	Probably involved in connections of major cytoskeletal structures to the plasma membrane. High molecular weight cytoskeletal protein concentrated at regions of cell-substratum contact and, in lymphocytes, at cell-cell contacts By similarity.
**TMF1**	**TATA element modulatory factor**	**[3]**	4.88	205	144	1.4	76.67	-0.830	Transcription factor	Regulation of nucleobase, nucleoside, nucleotide and nucleic acid metabolism	Golgi apparatus; Nucleus; Cytoplasm	Potential coactivator of the androgen receptor. Mediates STAT3 degradation. May play critical roles in two RAB6-dependent retrograde transport processes: one from endosomes to the Golgi and the other from the Golgi to the ER. This protein binds the HIV-1 TATA element and inhibits transcriptional activation by the TATA-binding protein (TBP).
**TTBK1**	**Tau-tubulin kinase 1**	**[11]**	5.46	195	163	1.2	65.71	-0.757	**Serine/threonine kinase**	**Cell communication; Signal transduction**	**Cytoplasm**	**Serine/threonine kinase which is able to phosphorylate TAU on serine, threonine and tyrosine residues. Induces aggregation of TAU.**
TCP1	T-complex protein 1 subunit alpha		5.80	74	67	1.1	105.09	-0.038	Chaperone	Protein metabolism	Cytoplasm; Nucleus; Cytosol; Microtubule	Molecular chaperone; assists the folding of proteins upon ATP hydrolysis. As part of the BBS/CCT complex may play a role in the assembly of BBSome, a complex involved in ciliogenesis regulating transports vesicles to the cilia. Known to play a role, in vitro, in the folding of actin and tubulin.
CCT2	T-complex protein 1 subunit beta		6.02	72	63	1.1	103.30	-0.018	Chaperone	Protein metabolism	Cytoplasm; Nucleolus; Microtubule; Mitochondrion	Molecular chaperone; assists the folding of proteins upon ATP hydrolysis. As part of the BBS/CCT complex may play a role in the assembly of BBSome, a complex involved in ciliogenesis regulating transports vesicles to the cilia. Known to play a role, in vitro, in the folding of actin and tubulin.
CCT4	T-complex protein 1 subunit delta		8.13	64	66	1.0	108.22	0.025	Chaperone	Protein metabolism	Cytoplasm; Nucleolus; Microtubule; Mitochondrion	Molecular chaperone; assists the folding of proteins upon ATP hydrolysis. As part of the BBS/CCT complex may play a role in the assembly of BBSome, a complex involved in ciliogenesis regulating transports vesicles to the cilia. Known to play a role, in vitro, in the folding of actin and tubulin.
**CCT5**	**T-complex protein 1 subunit epsilon**	**[1]**	5.44	82	68	1.2	96.81	-0.196	Chaperone	Protein metabolism	Centrosome; Cytoplasm	Molecular chaperone; assists the folding of proteins upon ATP hydrolysis. As part of the BBS/CCT complex may play a role in the assembly of BBSome, a complex involved in ciliogenesis regulating transports vesicles to the cilia. Known to play a role, in vitro, in the folding of actin and tubulin.
CCT7	T-complex protein 1 subunit eta		7.55	67	68	1.0	95.43	-0.098	Chaperone	Protein folding	Cytoplasm; Microtubule; Mitochondrion	Molecular chaperone; assists the folding of proteins upon ATP hydrolysis. Known to play a role, in vitro, in the folding of actin and tubulin By similarity.
CCT3	T-complex protein 1 subunit gamma		6.10	76	71	1.1	97.16	-0.252	Chaperone	Protein metabolism	Cytoplasm; Nucleolus; Microtubule	Molecular chaperone; assists the folding of proteins upon ATP hydrolysis. As part of the BBS/CCT complex may play a role in the assembly of BBSome, a complex involved in ciliogenesis regulating transports vesicles to the cilia. Known to play a role, in vitro, in the folding of actin and tubulin.
CCT8	T-complex protein 1 subunit theta		5.41	74	59	1.3	95.74	-0.071	Chaperone	Protein metabolism	Cytoplasm; Microtubule; Mitochondrion	Molecular chaperone; assists the folding of proteins upon ATP hydrolysis. As part of the BBS/CCT complex may play a role in the assembly of BBSome, a complex involved in ciliogenesis regulating transports vesicles to the cilia. Known to play a role, in vitro, in the folding of actin and tubulin.
CCT6A	T-complex protein 1 subunit zeta		6.25	75	70	1.1	103.42	-0.100	Chaperone	Protein metabolism	Cytoplasm; Microtubule	Molecular chaperone; assists the folding of proteins upon ATP hydrolysis. Known to play a role, in vitro, in the folding of actin and tubulin.
**RIF1**	**Telomere-associated protein RIF1**	**[1]**	5.39	352	286	1.2	83.60	-0.498	**DNA binding protein**	**Regulation of nucleobase, nucleoside, nucleotide and nucleic acid metabolism**	**Nucleus**	**Required for checkpoint mediated arrest of cell cycle progression in response to DNA damage during S-phase (the intra-S-phase checkpoint). This checkpoint requires activation of at least 2 parallel pathways by the ATM kinase: one involves the MRN (MRE11A-RAD50-NBS1) complex, while the second requires CHEK2. RIF1 seems to act independently of both these pathways. Seems to play no role in either the G1/S or G2/M DNA damage checkpoints.**
TNC	Tenascin		4.79	312	199	1.6	73.23	-0.389	Adhesion molecule	Cell communication; Signal transduction	Extracellular; Plasma membrane	Extracellular matrix protein implicated in guidance of migrating neurons as well as axons during development, synaptic plasticity as well as neuronal regeneration. Promotes neurite outgrowth from cortical neurons grown on a monolayer of astrocytes.
SPOCK2	Testican-2		4.65	75	45	1.7	56.84	-0.743	Extracellular matrix protein	Cell growth and/or maintenance	Extracellular; Nucleus	May participate in diverse steps of neurogenesis. Binds calcium.
CLEC3B	Tetranectin		5.80	24	22	1.1	72.27	-0.484	Secreted polypeptide	Lectin	Extracellular	Tetranectin binds to plasminogen and to isolated kringle 4. May be involved in the packaging of molecules destined for exocytosis.
**TXN**	**Thioredoxin (delta 3)**	**[10]**	4.82	17	12	1.4	75.87	-0.115	Enzyme: Reductase	Metabolism; Energy pathways	Cytoplasm; Nucleus; Extracellular	Participates in various redox reactions through the reversible oxidation of its active center dithiol to a disulfide and catalyzes dithiol-disulfide exchange reactions. Plays a role in the reversible S-nitrosylation of cysteine residues in target proteins, and thereby contributes to the response to intracellular nitric oxide. Nitrosylates the active site Cys of CASP3 in response to nitric oxide (NO), and thereby inhibits caspase-3 activity.
PRDX3	Thioredoxin-dependent peroxide reductase, mitochondrial		5.77	22	17	1.3	90.88	-0.058	**Enzyme: Peroxidase**	**Metabolism; Energy pathways**	**Mitochondrion**	**Involved in redox regulation of the cell. Protects radical-sensitive enzymes from oxidative damage by a radical-generating system. Acts synergistically with MAP3K13 to regulate the activation of NF-kappa-B in the cytosol.**
**F2**	**Thrombin light chain**	**[7]**	4.65	9	6	1.5	67.78	-0.878	Coagulation factor	Protein metabolism	Extracellular	Thrombin, which cleaves bonds after Arg and Lys, converts fibrinogen to fibrin and activates factors V, VII, VIII, XIII, and, in complex with thrombomodulin, protein C. Functions in blood homeostasis, inflammation and wound healing.
THBS1	Thrombospondin-1		4.70	182	115	1.6	57.60	-0.755	Extracellular matrix protein	Cell growth and/or maintenance	Extracellular	Adhesive glycoprotein that mediates cell-to-cell and cell-to-matrix interactions. Binds heparin. May play a role in dentinogenesis and/or maintenance of dentin and dental pulp By similarity. Ligand for CD36 mediating antiangiogenic properties. Plays a role in ER stress response, via its interaction with the activating transcription factor 6 alpha (ATF6) which produces adaptive ER stress response factors By similarity.
THBS2	Thrombospondin-2		4.61	180	100	1.8	58.49	-0.674	Extracellular matrix protein	Cell growth and/or maintenance	Extracellular	Adhesive glycoprotein that mediates cell-to-cell and cell-to-matrix interactions. Ligand for CD36 mediating antiangiogenic properties.
THBS4	Thrombospondin-4		4.40	148	83	1.8	67.93	-0.578	Extracellular matrix protein	Cell growth and/or maintenance	Extracellular	Adhesive glycoprotein that mediates cell-to-cell and cell-to-matrix interactions and is involved in various processes including cellular proliferation, migration, adhesion and attachment, inflammatory response to CNS injury, regulation of vascular inflammation and adaptive responses of the heart to pressure overload and in myocardial function and remodeling.
SERPINA7	Thyroxine-binding globulin		5.76	43	34	1.3	90.61	-0.069	Protease inhibitor	Protein metabolism	Extracellular	Major thyroid hormone transport protein in serum.
**TJP2**	**Tight junction protein ZO-2**	**[3]**	6.96	186	184	1.0	62.71	-0.948	Cell junction protein	Cell communication; Signal transduction	Cytoplasm; Nucleus; Cell junction	Plays a role in tight junctions and adherens junctions.
**TJAP1**	**Tight junction-associated protein 1**	**[3]**	5.67	84	68	1.2	73.26	-0.792	**Cell junction protein**	**Cell growth and/or maintenance**	**Plasma membrane**	**Incorporation of the encoded protein into tight junctions occurs at a late stage of formation of the junctions. The encoded protein localizes to the Golgi and may function in vesicle trafficking.**
TICAM2	TIR domain-containing adapter molecule 2		4.99	35	23	1.5	72.91	-0.626	**Adapter molecule**	**Cell communication; Signal transduction**	**Cell membrane, Cytoplasm, Endoplasmic reticulum, Endosome, Golgi apparatus, Membrane**	**Functions as sorting adapter in LPS-TLR4 signaling to regulate the MYD88-independent pathway during the innate immune response to LPS. Physically bridges TLR4 and TICAM1 and functionally transmits LPS-TRL4 signal to TICAM1; signaling is proposed to occur in early endosomes after endocytosis of TLR4. Involved in IL-18 signaling and is proposed to function as a sorting adaptor for MYD88 in IL-18 signaling during adaptive immune response.**
TTN	Titin		6.02	4913	4583	1.1	80.40	-0.468	Structural proteinEnzyme: Phosphotransferase	Transport; Muscle development; Muscle contraction	Cytoplasm; Nucleus; Sarcoplasmic reticulum	Key component in the assembly and functioning of vertebrate striated muscles. By providing connections at the level of individual microfilaments, it contributes to the fine balance of forces between the two halves of the sarcomere. The size and extensibility of the cross-links are the main determinants of sarcomere extensibility properties of muscle. In non-muscle cells, seems to play a role in chromosome condensation and chromosome segregation during mitosis. Might link the lamina network to chromatin or nuclear actin, or both during interphase.
**SUPT6H**	**Transcription elongation factor SPT6**	**[1]**	4.81	338	226	1.5	67.98	-0.858	**Transcription factor**	**Regulation of nucleobase, nucleoside, nucleotide and nucleic acid metabolism**	**Nucleus; Cytoplasm**	**Transcription elongation factor which binds histone H3 and plays a key role in the regulation of transcription elongation and mRNA processing. Enhances the transcription elongation by RNA polymerase II (RNAPII) and is also required for the efficient activation of transcriptional elongation by the HIV-1 nuclear transcriptional activator, Tat.**
BTF3	Transcription factor BTF3		9.41	25	30	0.8	69.71	-0.742	Transcription factor	Regulation of nucleobase, nucleoside, nucleotide and nucleic acid metabolism	Nucleus	When associated with NACA, prevents inappropriate targeting of non-secretory polypeptides to the endoplasmic reticulum (ER). Binds to nascent polypeptide chains as they emerge from the ribosome and blocks their interaction with the signal recognition particle (SRP), which normally targets nascent secretory peptides to the ER. BTF3 is also a general transcription factor that can form a stable complex with RNA polymerase II. Required for the initiation of transcription
SOX11	Transcription factor SOX-11		4.91	67	48	1.4	57.89	-0.702	**Transcription factor**	**Regulation of nucleobase, nucleoside, nucleotide and nucleic acid metabolism**	**Nucleus**	**Transcriptional factor involved in the embryonic neurogenesis. May also have a role in tissue modeling during development.**
**SOX30**	**Transcription factor SOX-30**	**[4]**	6.92	72.00	70.00	1.0	69.07	-0.553	**Transcription factor**	**Regulation of gene expression, epigenetic**	**Nucleus**	**Transcriptional activator. Binds to the DNA sequence 5'-ACAAT-3' and shows a preference for guanine residues surrounding this core motif.**
SOX4	Transcription factor SOX-4		6.87	47	46	1.0	55.42	-0.483	Transcription factor	Regulation of nucleobase, nucleoside, nucleotide and nucleic acid metabolism	Nucleus	Transcriptional activator that binds with high affinity to the T-cell enhancer motif 5'-AACAAAG-3' motif.
GTF2A1	Transcription initiation factor IIA beta chain		3.92	38	10	3.8	47.75	-1.496	**Transcription factor**	**Regulation of nucleobase, nucleoside, nucleotide and nucleic acid metabolism**	**Kinetochore; Nucleus; Cytoplasm**	**TFIIA is a component of the transcription machinery of RNA polymerase II and plays an important role in transcriptional activation. TFIIA in a complex with TBP mediates transcriptional activity.**
PURA	Transcriptional activator protein Pur-alpha		6.06	44	41	1.1	63.35	-0.752	Transcription factor	Regulation of nucleobase, nucleoside, nucleotide and nucleic acid metabolism	Nucleus; Cytoplasm	This is a probable transcription activator that specifically binds the purine-rich single strand of the PUR element located upstream of the MYC gene. May play a role in the initiation of DNA replication and in recombination.
TRA2A	Transformer-2 protein homolog alpha		11.27	28	66	0.4	26.33	-1.600	RNA binding protein	Regulation of nucleobase, nucleoside, nucleotide and nucleic acid metabolism	Nucleus; Mitochondrion; Cytoplasm	Sequence-specific RNA-binding protein which participates in the control of pre-mRNA splicing.
TRA2B	Transformer-2 protein homolog beta		11.25	30	71	0.4	27.21	-1.629	RNA binding protein	Regulation of nucleobase, nucleoside, nucleotide and nucleic acid metabolism	Nucleus; Nucleolus; Mitochondrion; Cytoplasm	Sequence-specific RNA-binding protein which participates in the control of pre-mRNA splicing. Can either activate or suppress exon inclusion. Acts additively with RBMX to promote exon 7 inclusion of the survival motor neuron SMN2. Activates the splicing of MAPT/Tau exon 10. Alters pre-mRNA splicing patterns by antagonizing the effects of splicing regulators, like RBMX. Binds to the AG-rich SE2 domain in the SMN exon 7 RNA. Binds to pre-mRNA.
**TGFBI**	**Transforming growth factor-beta-induced protein ig-h3**	**[1]**	7.37	69	69	1.0	98.56	-0.158	Ligand	Cell communication; Signal transduction	Extracellular; Cytoplasm; Nucleus	Binds to type I, II, and IV collagens. This adhesion protein may play an important role in cell-collagen interactions. In cartilage, may be involved in endochondral bone formation.
RHOA	Transforming protein RhoA		5.83	31	29	1.1	79.00	-0.436	GTPase	Biological process: Cell communication; Signal transduction	Actin cytoskeleton; Cytoplasm; Nucleus; Plasma membrane	Regulates a signal transduction pathway linking plasma membrane receptors to the assembly of focal adhesions and actin stress fibers. Involved in a microtubule-dependent signal that is required for the myosin contractile ring formation during cell cycle cytokinesis. Plays an essential role in cleavage furrow formation. Required for the apical junction formation of keratinocyte cell-cell adhesion. The MEMO1-RHOA-DIAPH1 signaling pathway plays an important role in ERBB2-dependent stabilization of microtubules at the cell cortex.
**TRPA1**	**Transient receptor potential cation channel subfamily A member 1**	**[1]**	6.69	124	117	1.1	96.38	-0.114	**Ion channel**	**Transport**	**Plasma membrane**	**Receptor-activated non-selective cation channel involved in detection of pain and possibly also in cold perception and inner ear function. Has a central role in the pain response to endogenous inflammatory mediators and to a diverse array of volatile irritants, such as mustard oil, garlic and acrolein, an irritant from tears gas and vehicule exhaust fumes. Acts also as a ionotropic cannabinoid receptor by being activated by delta(9)-tetrahydrocannabinol (THC), the psychoactive component of marijuana. Not involved in menthol sensation. May be a component for the mechanosensitive transduction channel of hair cells in inner ear, thereby participating in the perception of sounds. Probably operated by a phosphatidylinositol second messenger system (By similarity).**
VCP	Transitional endoplasmic reticulum ATPase		5.14	130	105	1.2	89.18	-0.350	ATPase	Metabolism; Energy pathways	Endoplasmic reticulum; Cytoplasm; Nucleus; Golgi apparatus; Nucleolus; Perinuclear region;	Necessary for the fragmentation of Golgi stacks during mitosis and for their reassembly after mitosis. Involved in the formation of the transitional endoplasmic reticulum (tER). The transfer of membranes from the endoplasmic reticulum to the Golgi apparatus occurs via 50-70 nm transition vesicles which derive from part-rough, part-smooth transitional elements of the endoplasmic reticulum (tER).
**TKT**	**Transketolase**	**[1]**	7.58	70	71	1.0	86.69	-0.174	Enzyme: Transketolase	Metabolism; Energy pathways	Cytoplasm	Catalyzes the transfer of a two-carbon ketol group from a ketose donor to an aldose acceptor, via a covalent intermediate with the cofactor thiamine pyrophosphate.

SSR1	Translocon-associated protein subunit alpha		4.30	51	25	2.0	82.87	-0.459	Membrane transport protein	Transport	Endoplasmic reticulum; Microsome; Mitochondrion; Nucleus	TRAP proteins are part of a complex whose function is to bind calcium to the ER membrane and thereby regulate the retention of ER resident proteins. May be involved in the recycling of the translocation apparatus after completion of the translocation process or may function as a membrane-bound chaperone facilitating folding of translocated proteins.
**TMTC1**	**Transmembrane and TPR repeat-containing protein 1 (ARG99 protein)**	[3]	9.05	74	94	0.8	95.24	-0.052	Unclassified	Unknown	Plasma membrane	??
**TMC7**	**Transmembrane channel-like protein 7**	[1]	8.74	65	77	0.8	102.75	0.184	Integral membrane protein	Unknown	Integral to membrane	Probable ion channel.
**TTR**	**Transthyretin**	[1-2]	5.31	17	12	1.4	73.70	-0.274	Transport/cargo protein	Transport	Extracellular	Thyroid hormone-binding protein. Probably transports thyroxine from the bloodstream to the brain.
**TCOF1**	**Treacle protein**	[3]	9.06	197	218	0.9	51.53	-0.909	Transcription regulatory protein	Transcription; Cell migration; Ribosome biogenesis and assembly	Nucleoplasm; Nucleolus; Cytoplasm; Nucleus	May be involved in nucleolar-cytoplasmic transport. May play a fundamental role in early embryonic development, particularly in development of the craniofacial complex (By similarity). May participate in certain stages of ribosome biogenesis.
HADHA	Trifunctional enzyme subunit alpha, mitochondrial		8.98	84	98	0.9	93.87	-0.112	Enzyme: Dehydrogenase	Metabolism; Energy pathways	Mitochondrion; Nucleus	Mitochondrial trifunctional protein, which catalyzes the last three steps of mitochondrial beta-oxidation of long chain fatty acids. The mitochondrial membrane-bound heterocomplex is composed of four alpha and four beta subunits, with the alpha subunit catalyzing the 3-hydroxyacyl-CoA dehydrogenase and enoyl-CoA hydratase
HADHB	Trifunctional enzyme subunit beta, mitochondrial		9.47	40	55	0.7	84.03	-0.067	Enzyme: Dehydrogenase	Metabolism; Energy pathways	Mitochondrion	Acyl-CoA + acetyl-CoA = CoA + 3-oxoacyl-CoA
**TPI1**	**Triosephosphate isomerase**	[1, 4, 10]	5.65	36	31	1.2	84.97	-0.167	Enzyme: Isomerase	Metabolism; Energy pathways	Cytoplasm	D-glyceraldehyde 3-phosphate = glycerone phosphate.
TRIM16	Tripartite motif-containing protein 16		5.34	84	64	1.3	73.85	-0.552	Cytoskeletal protein	Cell growth and/or maintenance	Cytoplasm; Nucleus	May play a role in the regulation of keratinocyte differentiation.
**TRIM72**	**Tripartite motif-containing protein 72**	[1]	6.05	63	55	1.1	90.84	-0.220	Unclassified	Exocytosis, Transport	Cell membrane; Cytoplasmic vesicle membrane	Muscle-specific protein that plays a central role in cell membrane repair by nucleating the assembly of the repair machinery at injury sites. Specifically binds phosphatidylserine. Acts as a sensor of oxidation: upon membrane damage, entry of extracellular oxidative environment results in disulfide bond formation and homooligomerization at the injury site.
**TPP1**	**Tripeptidyl-peptidase 1**	[1-2, 4]	5.74	30	22	1.4	75.24	-0.185	Serine protease	Protein metabolism	Lysosome; Nucleus	Lysosomal serine protease with tripeptidyl-peptidase I activity. May act as a non-specific lysosomal peptidase which generates tripeptides from the breakdown products produced by lysosomal proteinases. Requires substrates with an unsubstituted N-terminus By similarity.
TPM3	Tropomyosin alpha-3 chain		4.68	80	52	1.5	83.40	-1.040	Cytoskeletal associated protein; Structural protein	Cell growth and/or maintenance	Cytoplasm; Extracellular; Cytosol	Binds to actin filaments in muscle and non-muscle cells. Plays a central role, in association with the troponin complex, in the calcium dependent regulation of vertebrate striated muscle contraction. Smooth muscle contraction is regulated by interaction with caldesmon. In non-muscle cells is implicated in stabilizing cytoskeleton actin
**TUBA1B**	**Tubulin alpha-1B chain**	[1]	4.94	64	40	1.6	80.86	-0.230	Structural protein	Cell growth and/or maintenance	Cytoplasm; Mitochondrion; Plasma membrane	Tubulin is the major constituent of microtubules. It binds two moles of GTP, one at an exchangeable site on the beta chain and one at a non-exchangeable site on the alpha chain.
**TUBA1C**	**Tubulin alpha-1C chain**	[4-5]	4.96	63	40	1.6	80.16	-0.234	Cytoskeletal protein	Cell growth and/or maintenance	Cytoplasm; Microtubule; Nucleus	Tubulin is the major constituent of microtubules. It binds two moles of GTP, one at an exchangeable site on the beta chain and one at a non-exchangeable site on the alpha chain.
TUBA4A	Tubulin alpha-4A chain		4.93	64	40	1.6	79.24	-0.252	Cytoskeletal protein	Cell growth and/or maintenance	Cytoplasm; Microtubule	Tubulin is the major constituent of microtubules. It binds two moles of GTP, one at an exchangeable site on the beta chain and one at a non-exchangeable site on the alpha chain.
**TUBA8**	**Tubulin alpha-8 chain**	[4]	4.94	64	40	1.6	80.80	-0.209	Cytoskeletal associated protein	Cell growth and/or maintenance	Cytoskeleton; Microtubule	Tubulin is the major constituent of microtubules. It binds two moles of GTP, one at an exchangeable site on the beta chain and one at a non-exchangeable site on the alpha chain.
TUBB	Tubulin beta chain		4.78	62	37	1.7	72.23	-0.348	Cytoskeletal protein	Cell growth and/or maintenance	Cytoplasm; Plasma membrane; Kinetochore	Tubulin is the major constituent of microtubules. It binds two moles of GTP, one at an exchangeable site on the beta chain and one at a non-exchangeable site on the alpha chain.
**TUBB2A**	**Tubulin beta-2A chain**	[4]	4.78	62	37	1.7	69.89	-0.408	Cytoskeletal protein	Cell growth and/or maintenance	Cytoplasm; Microtubule	Tubulin is the major constituent of microtubules. It binds two moles of GTP, one at an exchangeable site on the beta chain and one at a non-exchangeable site on the alpha chain.
TUBB2B	Tubulin beta-2B chain		4.78	62	37	1.7	69.66	-0.406	Cytoskeletal protein	Cell growth and/or maintenance	Cytoplasm; Microtubule	Tubulin is the major constituent of microtubules. It binds two moles of GTP, one at an exchangeable site on the beta chain and one at a non-exchangeable site on the alpha chain. TUBB2B is implicated in neuronal migration By similarity.
**TBB2C**	**Tubulin beta-2C chain**	[4]	4.83	62	37	1.7	71.19	-0.362	Cytoskeletal protein	Cell growth and/or maintenance	Cytoplasm; Microtubule	Tubulin is the major constituent of microtubules. It binds two moles of GTP, one at an exchangeable site on the beta chain and one at a non-exchangeable site on the alpha chain. TUBB2B is implicated in neuronal migration By similarity.
**TUBB3**	**Tubulin beta-3 chain**	[1]	4.83	63	38	1.7	71.71	-0.371	Structural protein	Cell growth and/or maintenance	Cytoplasm	Tubulin is the major constituent of microtubules. It binds two moles of GTP, one at an exchangeable site on the beta chain and one at a non-exchangeable site on the alpha chain. TUBB3 plays a critical role in proper axon guidance and mantainance.
TUBB4A	Tubulin beta-4A chain		4.78	61	36	1.7	71.80	-0.321	Cytoskeletal protein	Cell growth and/or maintenance	Microtubule	Tubulin is the major constituent of microtubules. It binds two moles of GTP, one at an exchangeable site on the beta chain and one at a non-exchangeable site on the alpha chain.
TUBB4B	Tubulin beta-4B chain		4.79	62	37	1.7	71.19	-0.362	Structural protein	Cell growth and/or maintenance	Cytoplasm; Nucleolus; Microtubule	Tubulin is the major constituent of microtubules. It binds two moles of GTP, one at an exchangeable site on the beta chain and one at a non-exchangeable site on the alpha chain.
**TUBB5**	**Tubulin beta-5 chain**	[4]	4.78	62	37	1.7	72.23	-0.348	Cytoskeletal protein	Cell growth and/or maintenance	Microtubule	Tubulin is the major constituent of microtubules. It binds two moles of GTP, one at an exchangeable site on the beta chain and one at a non-exchangeable site on the alpha chain.
**TUBB6**	**Tubulin beta-6 chain**	[1]	4.77	61	36	1.7	71.70	-0.326	Cytoskeletal protein	Cell growth and/or maintenance	Cytoplasm; Microtubule	Tubulin is the major constituent of microtubules. It binds two moles of GTP, one at an exchangeable site on the beta chain and one at a non-exchangeable site on the alpha chain By similarity.
TTLL12	Tubulin--tyrosine ligase-like protein 12		5.33	93	65	1.4	79.18	-0.390	Enzyme: Ligase	Metabolism; Energy pathways	Nucleus; Cytoplasm	It has both SET-like and TTL-like domains, suggesting that it could have histone methylation and tubulin tyrosine ligase activities. Altered expression of hTTLL12 in human cells leads to specific changes in H4K20 trimethylation, and tubulin detyrosination.
**TNFAIP2**	**Tumor necrosis factor alpha-induced protein 2**	[3]	6.06	87	79	1.1	96.18	-0.274	Unclassified	Unknown	Extracellular	May play a role as a mediator of inflammation and angiogenesis.
TACSTD2	Tumor-associated calcium signal transducer 2		8.98	36	45	0.8	87.64	-0.345	Cell surface receptor	Cell communication; Signal transduction	Plasma membrane; Cytoplasm	May function as a growth factor receptor.
PTPN13	Tyrosine-protein phosphatase non-receptor type 13		5.99	324	285	1.1	80.91	-0.521	Tyrosine phosphatase	Cell communication; Signal transduction; Apoptosis	Plasma membrane; Cytoplasm; Early endosome	Tyrosine phosphatase which regulates negatively FAS-induced apoptosis and NGFR-mediated pro-apoptotic signaling.
PTPN6	Tyrosine-protein phosphatase non-receptor type 6		7.65	76	77	1.0	74.20	-0.687	Tyrosine phosphatase	Cell communication; Signal transduction; Apoptosis	Cytoplasm; Mitochondrion	Modulates signaling by tyrosine phosphorylated cell surface receptors such as KIT and the EGF receptor/EGFR. The SH2 regions may interact with other cellular components to modulate its own phosphatase activity against interacting substrates. Together with MTUS1, induces UBE2V2 expression upon angiotensin II stimulation. Plays a key role in hematopoiesis.
USP31	Ubiquitin carboxyl-terminal hydrolase 31		9.35	127	169	0.8	62.14	-0.641	Ubiquitin proteasome system protein	Protein metabolism	Nucleus	May recognize and hydrolyze the peptide bond at the C-terminal Gly of ubiquitin. Involved in the processing of poly-ubiquitin precursors as well as that of ubiquitinated proteins By similarity.
USP5	Ubiquitin carboxyl-terminal hydrolase 5, Isoform Short		4.91	135	93	1.5	76.11	-0.442	Ubiquitin proteasome system protein	Protein metabolism	Extracellular	Cleaves linear and branched multiubiquitin polymers with a marked preference for branched polymers. Involved in unanchored 'Lys-48'-linked polyubiquitin disassembly. Binds linear and 'Lys-63'-linked polyubiquitin with a lower affinity.
UCHL3	Ubiquitin carboxyl-terminal hydrolase isozyme L3		4.84	40	22	1.8	83.09	-0.405	Ubiquitin proteasome system protein	Protein metabolism	Cytoplasm	Deubiquitinating enzyme (DUB) that controls levels of cellular ubiquitin through processing of ubiquitin precursors and ubiquitinated proteins. Thiol protease that recognizes and hydrolyzes a peptide bond at the C-terminal glycine of either ubiquitin or NEDD8. Deubiquitinates ENAC in apical compartments, thereby regulating apical membrane recycling. Indirectly increases the phosphorylation of IGFIR, AKT and FOXO1 and promotes insulin-signaling and insulin-induced adipogenesis. Required for stress-response retinal, skeletal muscle and germ cell maintenance. May be involved in working memory.
OTUB1	Ubiquitin thioesterase OTUB1		4.85	46	28	1.6	79.81	-0.561	Ubiquitin proteasome system protein	Protein metabolism	Cytoplasm	Hydrolase that can specifically remove 'Lys-48'-linked conjugated ubiquitin from proteins and plays an important regulatory role at the level of protein turnover by preventing degradation. Regulator of T-cell anergy, a phenomenon that occurs when T-cells are rendered unresponsive to antigen rechallenge and no longer respond to their cognate antigen.
ZRANB1	Ubiquitin thioesterase ZRANB1		5.47	103	85	1.2	75.37	-0.528	Transcription regulatory protein	Regulation of nucleobase, nucleoside, nucleotide and nucleic acid metabolism	Cytoplasm; Nucleus	Specifically hydrolyzes 'Lys-29'-linked and 'Lys-33'-linked diubiquitin. Also cleaves 'Lys-63'-linked chains, but with 40-fold less efficiency compared to 'Lys-29'-linked ones. Positive regulator of the Wnt signaling pathway that deubiquitinates APC protein, a negative regulator of Wnt-mediated transcription. Plays a role in the regulation of cell morphology and cytoskeletal organization. Required in the stress fiber dynamics and cell migration. May also modulate TNF-alpha signaling.
**RPS27A**	**Ubiquitin-40S ribosomal protein S27a**	[1-2, 11]	9.86	8	24	0.3	43.88	-1.181	Ubiquitin proteasome system protein	Protein metabolism	Ribosome; Cytoplasm	Component of the 40S subunit of the ribosome.
UBA52	Ubiquitin-60S ribosomal protein L40		10.32	2	16	0.1	69.42	-1.015	Ribosomal subunit	Protein metabolism	Ribosome; Extracellular	Component of the 60S subunit of the ribosome. Ribosomal protein L40 is essential for translation of a subset of cellular transcripts, and especially for cap-dependent translation of vesicular stomatitis virus mRNAs.
UBE2H	Ubiquitin-conjugating enzyme E2 H		4.55	31	17	1.8	71.91	-0.549	Ubiquitin proteasome system protein	Protein metabolism	Nucleus	Accepts ubiquitin from the E1 complex and catalyzes its covalent attachment to other proteins. In vitro catalyzes 'Lys-11'- and 'Lys-48'-linked polyubiquitination. Capable, in vitro, to ubiquitinate histone H2A.
UBA1	Ubiquitin-like modifier-activating enzyme 1		5.49	130	105	1.2	84.67	-0.269	Ubiquitin proteasome system protein	Proteolysis and peptidolysis	Nucleus; Cytoplasm; Cytosol	Catalyzes the first step in ubiquitin conjugation to mark cellular proteins for degradation through the ubiquitin-proteasome system. Activates ubiquitin by first adenylating its C-terminal glycine residue with ATP, and thereafter linking this residue to the side chain of a cysteine residue in E1, yielding a ubiquitin-E1 thioester and
UBA7	Ubiquitin-like modifier-activating enzyme 7		5.64	120	86	1.4	93.82	-0.146	Ubiquitin proteasome system protein	Proteolysis and peptidolysis	Cytoplasm; Nucleus	Activates ubiquitin by first adenylating with ATP its C-terminal glycine residue and thereafter linking this residue to the side chain of a cysteine residue in E1, yielding a ubiquitin-E1 thioester and free AMP. Catalyzes the ISGylation of influenza A virus NS1 protein.
GALE	UDP-glucose 4-epimerase		6.26	38	35	1.1	85.20	-0.205	Enzyme: Epimerase	Metabolism	Cytoplasm	Catalyzes two distinct but analogous reactions: the reversible epimerization of UDP-glucose to UDP-galactose and the reversible epimerization of UDP-N-acetylglucosamine to UDP-N-acetylgalactosamine. It contributes to the catabolism of dietary galactose and enables the endogenous biosynthesis of both UDP-Gal and UDP-GalNAc when exogenous sources are limited. Both UDP-sugar interconversions are important in the synthesis of glycoproteins and glycolipids.
UGT1A1	UDP-glucuronosyltransferase 1-1		8.24	46	49	0.9	92.68	0.004	Enzyme: Glycosyltransferase	Metabolism; Energy pathways	Endoplasmic reticulum	This isoform glucuronidates bilirubin IX-alpha to form both the IX-alpha-C8 and IX-alpha-C12 monoconjugates and diconjugate. Is also able to catalyze the glucuronidation of 17beta-estradiol, 17alpha-ethinylestradiol, 1-hydroxypyrene, 4-methylumbelliferone, 1-naphthol, paranitrophenol, scopoletin, and umbelliferone. Isoform 2 lacks transferase activity but acts as a negative regulator of isoform 1.
UGT1A6	UDP-glucuronosyltransferase 1-6		8.49	53	58	0.9	92.04	-0.101	Enzyme: Glycosyltransferase	Xenobiotic metabolism	Endoplasmic reticulum	UDPGT is of major importance in the conjugation and subsequent elimination of potentially toxic xenobiotics and endogenous compounds. Conjugates small planar phenolic molecules such as 4-nitrophenol, 1-naphthol, and 4-methylumbelliferone. The bulky phenol 4-hydroxybiphenyl, androgens and estrogens are not substrates. 2-hydroxybiphenyl is an excellent substrate.
KIAA0753	Uncharacterized protein KIAA0753		7.60	145	146	1.0	76.49	-0.775	Unclassified	Unclassified	Centrosome	??
MYO1C	Unconventional myosin-Ic		9.46	121	156	0.8	90.34	-0.387	Motor protein	Cell growth and/or maintenance	Plasma membrane; Cytoplasm; Nucleolus; Cytoskeleton	Involved in glucose transporter recycling in response to insulin by regulating movement of intracellular GLUT4-containing vesicles to the plasma membrane. Component of the hair cell's (the sensory cells of the inner ear) adaptation-motor complex. Acts as a mediator of adaptation of mechanoelectrical transduction in stereocilia of vestibular hair cells. Binds phosphoinositides and links the actin cytoskeleton to cellular membranes.
**MYO5B**	**Unconventional myosin-Vb**	[1]	6.77	257	249	1.0	86.56	-0.570	Structural protein	Cell growth and/or maintenance	Cytoplasm	May be involved in vesicular trafficking via its association with the CART complex. The CART complex is necessary for efficient transferrin receptor recycling but not for EGFR degradation. Required in a complex with RAB11A and RAB11FIP2 for the transport of NPC1L1 to the plasma membrane. Together with RAB11A participates in CFTR trafficking to the plasma membrane and TF (transferrin) recycling in nonpolarized cells. Together with RAB11A and RAB8A participates in epithelial cell polarization. Together with RAB25 regulates transcytosis.
MYO6	Unconventional myosin-VI		8.74	192	210	0.9	77.58	-0.678	Motor protein	Cell growth and/or maintenance	Golgi apparatus; Cytoplasm	Functions in a variety of intracellular processes such as vesicular membrane trafficking and cell migration. Required for the structural integrity of the Golgi apparatus via the p53-dependent pro-survival pathway. Appears to be involved in a very early step of clathrin-mediated endocytosis in polarized epithelial cells. May act as a regulator of F-actin dynamics. May play a role in transporting DAB2 from the plasma membrane to specific cellular targets. Required for structural integrity of inner ear
**UPP2**	**Uridine phosphorylase 2**	[1]	6.21	40	36	1.1	94.70	0.018	Enzyme: Phosphorylase	Regulation of nucleobase, nucleoside, nucleotide and nucleic acid metabolism	Cytoplasm	Catalyzes the reversible phosphorylytic cleavage of uridine and deoxyuridine to uracil and ribose- or deoxyribose-1-phosphate. The produced molecules are then utilized as carbon and energy sources or in the rescue of pyrimidine bases for nucleotide synthesis. Shows substrate specificity and accept uridine, deoxyuridine, and thymidine as well as the two pyrimidine nucleoside analogs 5-fluorouridine and 5-fluoro-2(')-deoxyuridine as substrates.

UMOD	Uromodulin (Tamm-Horsfall Protein)	[1-12]	4.84	68	40	1.7	64.09	-0.218	Integral membrane protein	Immune response; Cellular defense response	Plasma membrane	Functions in biogenesis and organization of the apical membrane of epithelial cells of the thick ascending limb of Henle's loop (TALH), where it promotes formation of complex filamentous gel-like structure providing the water barrier permeability. May serve as a receptor for binding and endocytosis for cytokines (IL-1, IL-2) and TNF. Facilitates neutrophil migration across renal epithelial. Secreted into urine after proteolytically cleaveage. Into the urine, may contribute to colloid osmotic pressure, retards passage of positively charged electrolytes, prevents urinary tract infection and modulates formation of supersaturated salts and their crystals.
UPK2	Uroplakin-2		10.11	5	10	0.5	115.90	0.625	Integral membrane protein	Cell growth and/or maintenance	Plasma membrane; Endoplasmic reticulum	Component of the asymmetric unit membrane (AUM); a highly specialized biomembrane elaborated by terminally differentiated urothelial cells. May play an important role in regulating the assembly of the AUM By similarity.
UPK3A	Uroplakin-3a		4.57	26	16	1.6	90.26	-0.009	Cell surface receptor	Cell communication; Signal transduction	Plasma membrane; Endoplasmic reticulum	Component of the asymmetric unit membrane (AUM); a highly specialized biomembrane elaborated by terminally differentiated urothelial cells. May play an important role in AUM-cytoskeleton interaction in terminally differentiated urothelial cells. It also contributes to the formation of urothelial glycocalyx which may play an important role in preventing bacterial adherence By similarity.
UGP2	UTP--glucose-1-phosphate uridylyltransferase		8.15	60	62	1.0	94.53	-0.293	Enzyme: Nucleotidyltransferase	Metabolism; Energy pathways	Cytoplasm	Plays a central role as a glucosyl donor in cellular metabolic pathways.
VPS13C	Vacuolar protein sorting-associated protein 13C*		6.38	468	442	1.1	96.78	-0.212	Transport/cargo protein	Transport	Nucleus; Extracellular vesicular exosome	No functional information for this protein
VPS16	Vacuolar protein sorting-associated protein 16 homolog	[3]	6.32	110	102	1.1	93.06	-0.242	Transport/cargo protein	Transport	Late endosome; Lysosome; Membrane fraction; Cytoplasm	May play a role in vesicle-mediated protein trafficking to lysosomal compartments and in membrane docking/fusion reactions of late endosomes/lysosomes.
VPS35	Vacuolar protein sorting-associated protein 35*		5.32	117	89	1.3	97.61	-0.306	Transport/cargo protein	Transport	Cytoplasm; Plasma membrane; Endoplasmic reticulum; Golgi apparatus; Endosome; Membrane fraction	Essential component of the retromer complex, a complex required to retrieve lysosomal enzyme receptors (IGF2R and M6PR) from endosomes to the trans-Golgi network. Also required to regulate transcytosis of the polymeric immunoglobulin receptor (pIgR-pIgA).
VPS4B	Vacuolar protein sorting-associated protein 4B*		6.75	64	63	1.0	81.53	-0.552	Transport/cargo protein	Transport	Perinuclear region	Involved in late steps of the endosomal multivesicular bodies (MVB) pathway. Recognizes membrane-associated ESCRT-III assemblies and catalyzes their disassembly, possibly in combination with membrane fission. Redistributes the ESCRT-III components to the cytoplasm for further rounds of MVB sorting.
VARS	Valine--tRNA ligase		7.52	144	145	1.0	86.67	-0.271	Enzyme: Ligase	Metabolism; Energy pathways	Cytoplasm	ATP + L-valine + tRNA(Val) = AMP + diphosphate + L-valyl-tRNA(Val).
VASN	Vasorin	[2 , 11]	6.95	57	56	1.0	95.18	-0.116	Integral membrane protein	Cell communication; Signal transduction	Plasma membrane; Extracellular	May act as an inhibitor of TGF-beta signaling.
NSF	Vesicle-fusing ATPase		6.52	96	93	1.0	95.03	-0.220	ATPase	Metabolism; Energy pathways	Cytoplasm; Golgi apparatus; Plasma membrane; Cytosol	Required for vesicle-mediated transport. Catalyzes the fusion of transport vesicles within the Golgi cisternae. Is also required for transport from the endoplasmic reticulum to the Golgi stack. Seems to function as a fusion protein required for the delivery of cargo proteins to all compartments of the Golgi stack independent of
LMAN2	Vesicular integral-membrane protein VIP36	[1-2, 5, 8, 11]	6.06	41	35	1.2	74.90	-0.467	Transport/cargo protein	Transport	Golgi apparatus; Cytoplasm; Mitochondrion	Plays a role as an intracellular lectin in the early secretory pathway. Interacts with N-acetyl-D-galactosamine and high-mannose type glycans and may also bind to O-linked glycans. Involved in the transport and sorting of glycoproteins carrying high mannose-type glycans By similarity.
VCL	Vinculin		5.51	164	145	1.1	86.64	-0.408	Cytoskeletal associated protein	Cell growth and/or maintenance	Cytoplasm; Plasma membrane; Nucleus	Actin filament (F-actin)-binding protein involved in cell-matrix adhesion and cell-cell adhesion. Regulates cell-surface E-cadherin expression and potentiates mechanosensing by the E-cadherin complex. May also play important roles in cell morphology and locomotion.
GC	Vitamin D-binding protein	[1]	5.22	69	56	1.2	73.69	-0.414	Transport/cargo protein	Transport	Extracellular; Cytoplasm; Endosome; Plasma membrane	Multifunctional protein found in plasma, ascitic fluid, cerebrospinal fluid, and urine and on the surface of many cell types. In plasma, it carries the vitamin D sterols and prevents polymerization of actin by binding its monomers. DBP associates with membrane-bound immunoglobulin on the surface of B-lymphocytes and with IgG Fc receptor on the membranes of T-lymphocytes.
PROC	Vitamin K-dependent protein C	[3-5, 7]	5.60	62	47	1.3	77.92	-0.398	Coagulation factor	Protein metabolism	Extracellular	Protein C is a vitamin K-dependent serine protease that regulates blood coagulation by inactivating factors Va and VIIIa in the presence of calcium ions and phospholipids
PROS1	Vitamin K-dependent protein S	[1, 3-4, 12]	5.17	85	65	1.3	79.97	-0.344	Coagulation factor	Protein metabolism	Extracellular	Anticoagulant plasma protein; it is a cofactor to activated protein C in the degradation of coagulation factors Va and VIIIa. It helps to prevent coagulation and stimulating fibrinolysis.
PROZ	Vitamin K-dependent protein Z	[1-4, 8-12]	5.34	47	32	1.5	72.78	-0.377	Coagulation factor	Protein metabolism	Extracellular	Appears to assist hemostasis by binding thrombin and promoting its association with phospholipid vesicles. Inhibits activity of the coagulation protease factor Xa in the presence of SERPINA10, calcium and phospholipids.
VMO1	Vitelline membrane outer layer protein 1 homolog	[1]	4.65	22	13	1.7	61.40	-0.411	Unclassified	Unclassified	Extracellular	Exact function not known, component of the outer membrane of the vitelline layer of the egg. Seems to be able to synthesize N-acetylchito-oligosaccharides (n=14-15) from hexasaccharides of N-acetylglucosamine in a manner similar to the transferase activity of lysozyme.
VTN	Vitronectin	[3-5, 8, 10]	5.47	66	55	1.2	51.87	-0.835	Extracellular matrix protein	Cell growth and/or maintenance	Extracellular	Vitronectin is a cell adhesion and spreading factor found in serum and tissues. Vitronectin interact with glycosaminoglycans and proteoglycans. Is recognized by certain members of the integrin family and serves as a cell-to-substrate adhesion molecule. Inhibitor of the membrane-damaging effect of the terminal cytolytic complement pathway.
VDAC1	Voltage-dependent anion-selective channel protein 1	[1]	8.63	29	32	0.9	73.72	-0.419	Voltage gated channel	Transport	Mitochondrion; Plasma membrane; Endosome; Nucleolus; Zymogen granule	Forms a channel through the mitochondrial outer membrane and also the plasma membrane. The channel at the outer mitochondrial membrane allows diffusion of small hydrophilic molecules; in the plasma membrane it is involved in cell volume regulation and apoptosis. It adopts an open conformation at low or zero membrane potential and a closed conformation at potentials above 30-40 mV. The open state has a weak anion selectivity whereas the closed state is cation-selective. May participate in the formation of the permeability transition pore complex (PTPC) responsible for the release of mitochondrial products that triggers apoptosis.
CACNG8	Voltage-dependent calcium channel gamma-8 subunit	[8]	9.34	32	43	0.7	77.93	-0.099	Voltage gated channel	Transport	Plasma membrane	Regulates the trafficking and gating properties of AMPA-selective glutamate receptors (AMPARs). Promotes their targeting to the cell membrane and synapses and modulates their gating properties by slowing their rates of activation, deactivation and desensitization and by mediating their resensitization.
VWA1	von Willebrand factor A domain-containing protein 1	[1]	6.61	41	39	1.1	82.88	-0.188	Extracellular matrix protein	Extracellular structure organization and biogenesis	Extracellular	Promotes matrix assembly.
ATP6V0C	V-type proton ATPase 16 kDa proteolipid subunit	[1]	7.98	7	8	0.9	126.58	1.040	ATPase	Transport	Lysosome; Plasma membrane; Endosome; Golgi apparatus; Endoplasmic reticulum	Proton-conducting pore forming subunit of the membrane integral V0 complex of vacuolar ATPase. V-ATPase is responsible for acidifying a variety of intracellular compartments in eukaryotic cells.
ATP6V1A	V-type proton ATPase catalytic subunit A		5.34	81	67	1.2	85.93	-0.186	Transport/cargo protein	Metabolism; Energy pathways	Plasma membrane; Lysosome; Endosome; Secretory vesicle	Catalytic subunit of the peripheral V1 complex of vacuolar ATPase. V-ATPase vacuolar ATPase is responsible for acidifying a variety of intracellular compartments in eukaryotic cells.
ATP6V1B2	V-type proton ATPase subunit B, brain isoform		5.57	62	53	1.2	90.47	-0.160	Transport/cargo protein	Transport	Endosome	Non-catalytic subunit of the peripheral V1 complex of vacuolar ATPase. V-ATPase is responsible for acidifying a variety of intracellular compartments in eukaryotic cells
KIAA0196	WASH complex subunit strumpellin		6.54	139	134	1.0	101.88	-0.167	Unclassified	Cell death; Endosomal transport	Cytoplasm; Endoplasmic reticulum	Component of the WASH complex, a complex present at the surface of endosomes that recruits and activates the Arp2/3 complex to induce actin polymerization. The WASH complex plays a key role in the fission of tubules that serve as transport intermediates during endosome sorting. May be involved in axonal outgrowth.
WDR1	WD repeat-containing protein 1		6.18	68	58	1.2	81.14	-0.242	Cytoskeletal associated protein	Cell growth and/or maintenance	Cytoplasm	Induces disassembly of actin filaments in conjunction with ADF/cofilin family proteins.
WDR35	WD repeat-containing protein 35	[1]	5.98	150	135	1.1	89.52	-0.240	Unclassified	Unknown	Cilium assembly	Component of the IFT complex A (IFT-A), a complex required for retrograde ciliary transport. Required for ciliogenesis. May promote CASP3 activation and TNF-stimulated apoptosis.
PEPD	Xaa-Pro dipeptidase		5.64	64	48	1.3	81.42	-0.134	Protease	Protein metabolism	Cytoplasm	Splits dipeptides with a prolyl or hydroxyprolyl residue in the C-terminal position. Plays an important role in collagen metabolism because the high level of iminoacids in collagen.
XRCC5	X-ray repair cross-complementing protein 5		5.55	109	90	1.2	86.69	-0.339	DNA binding protein	Regulation of nucleobase, nucleoside, nucleotide and nucleic acid metabolism	Nucleus; Cytoplasm; Nucleolus; Mitochondrion	Single-stranded DNA-dependent ATP-dependent helicase. Has a role in chromosome translocation. The DNA helicase II complex binds preferentially to fork-like ends of double-stranded DNA in a cell cycle-dependent manner. It works in the 3'-5' direction. Binding to DNA may be mediated by XRCC6. Involved in DNA non-homologous end joining (NHEJ) required for double-strand break repair and V(D)J recombination.
XRCC6	X-ray repair cross-complementing protein 6		6.23	96	91	1.1	84.01	-0.540	DNA binding protein	Regulation of nucleobase, nucleoside, nucleotide and nucleic acid metabolism	Nucleus; Cytoplasm; Nucleolus	The XRCC5/6 dimer acts as regulatory subunit of the DNA-dependent protein kinase complex DNA-PK by increasing the affinity of the catalytic subunit PRKDC to DNA by 100-fold. The XRCC5/6 dimer is probably involved in stabilizing broken DNA ends and bringing them together.
YBX2	Y-box-binding protein 2		10.80	33	52	0.6	46.68	-0.907	DNA binding protein	Regulation of nucleobase, nucleoside, nucleotide and nucleic acid metabolism	Nucleus	Major constituent of messenger ribonucleoprotein particles (mRNPs). Involved in the regulation of the stability and/or translation of germ cell mRNAs.May mark specific mRNAs (those transcribed from Y-box promoters) in the nucleus for cytoplasmic storage, thereby linking transcription and mRNA storage/translational delay By similarity.
ZNF169	Zinc finger protein 169*	[12]	9.36	60	91	0.7	59.82	-0.774	Transcription factor	Regulation of nucleobase, nucleoside, nucleotide and nucleic acid metabolism	Nucleus	May be involved in transcriptional regulation.
ZNF281	Zinc finger protein 281		8.71	84	93	0.9	59.28	-0.711	Transcription regulatory protein	Regulation of nucleobase, nucleoside, nucleotide and nucleic acid metabolism	Nucleus	Transcription repressor that plays a role in regulation of embryonic stem cells (ESCs) differentiation.Not required for establishement and maintenance of ESCs By similarity. Represses the transcription of a number of genes including GAST, ODC1 and VIM.
ZNF282	Zinc finger protein 282		5.62	94	76	1.2	62.53	-0.810	Transcription regulatory protein	Regulation of nucleobase, nucleoside, nucleotide and nucleic acid metabolism	Nucleus	Binds to the U5 repressive element (U5RE) of the human T cell leukemia virus type I long terminal repeat. It recognizes the 5'-TCCACCCC-3' sequence as a core motif and exerts a strong repressive effect on HTLV-I LTR-mediated expression.
ZNF717	Zinc finger protein 717	[1]	8.91	97	129	0.8	52.04	-0.892	DNA binding protein	Transcription	Nucleus	May be involved in transcriptional regulation.
TRPS1	Zinc finger transcription factor Trps1	[3]	7.53	151	152	1.0	62.11	-0.791	Transcription factor	Regulation of nucleobase, nucleoside, nucleotide and nucleic acid metabolism	Nucleus	Transcriptional repressor. Binds specifically to GATA sequences and represses expression of GATA-regulated genes at selected sites and stages in vertebrate development. Regulates chondrocyte proliferation and differentiation. Executes multiple functions in proliferating chondrocytes, expanding the region of distal chondrocytes, activating proliferation in columnar cells and supporting the differentiation of columnar into hypertrophic chondrocytes.
ZAN	Zonadhesin		5.77	294	245	1.2	67.84	-0.361	Adhesion molecule	Cell communication; Signal transduction	Plasma membrane	Binds in a species-specific manner to the zona pellucida of the egg. May be involved in gamete recognition and/or signaling.

[1] Jou, Y. C., Fang, C. Y., Chen, S. Y., Chen, F. H., Cheng, M. C., Shen, C. H., Liao, L. W., et al., Urology 2012, 80, 260-266.
[2] Thurgood, L. A., Ryall, R. L., J Proteome Res 2010, 9, 5402-5412.
[3] Merchant, M. L., Cummins, T. D., Wilkey, D. W., Salyer, S. A., Powell, D. W., Klein, J. B., Lederer, E. D., Am J Physiol Renal Physiol 2008, 295, F1254-1258.
[4] Okumura, N., Tsujihata, M., Momohara, C., Yoshioka, I., Suto, K., Nonomura, N., Okuyama, A., et al., PLoS One 2013, 8, e68624.
[5] Canales, B. K., Anderson, L., Higgins, L., Ensrud-Bowlin, K., Roberts, K. P., Wu, B., Kim, I. W., et al., Urology 2010, 76, 1017 e1013-1020.
[6] Boonla, C., Tosukhowong, P., Spittau, B., Schlosser, A., Pimratana, C., Krieglstein, K., Clin Chim Acta 2014, 429, 81-89.
[7] Canales, B. K., Anderson, L., Higgins, L., Frethem, C., Ressler, A., Kim, I. W., Monga, M., Urol Res 2009, 37, 323-329.
[8] Canales, B. K., Anderson, L., Higgins, L., Slaton, J., Roberts, K. P., Liu, N., Monga, M., J Endourol 2008, 22, 1161-1167.
[9] Kaneko, K., Yoshida, N., Okazaki, K., Yamanobe, T., Yamaoka, N., Yasuda, M., Ogata, N., et al., Nucleosides Nucleotides Nucleic Acids 2011, 30, 1072-1076.
[10] Kaneko, K., Matsuta, Y., Moriyama, M., Yasuda, M., Chishima, N., Yamaoka, N., Fukuuchi, T., et al., Int J Urol 2014, 21, 341-346.
[11] Thurgood, L. A., Wang, T., Chataway, T. K., Ryall, R. L., J Proteome Res 2010, 9, 4745-4757.
[12] Kaneko, K., Kobayashi, R., Yasuda, M., Izumi, Y., Yamanobe, T., Shimizu, T., Int J Urol 2012, 19, 765-772.

Type of Stone:
[1] uric acid stone (242 proteins ID'd)
[2] calcium oxalate monohydrate and dihydrate crystals (39 proteins ID'd)
[3] calcium oxalate stone (158 proteins ID'd)

[4] calcium oxalate stone (92 proteins ID'd)
[5] calcium oxalate and calcium phosphate stones (113 proteins ID'd)
[6] calcium oxalate, uric acid and struvite stones (62 proteins ID'd)
[7] calcium phosphate (matrix) stone (33 proteins ID'd)
[8] calcium oxalate monohydrate stone (68 proteins ID'd)
[9] outside of the stone was calcium oxalate monohydrate and the inside of uric acid (51 proteins ID'd)
[10] calcium carbonate and calcium oxalate dehydrate (53 proteins ID'd)
[11] hydroxyapatite, brushite, and uric acid crystals (101 proteins ID'd)
[12] calcium oxalate monohydrate and calcium oxalate dehydrate stones; stones that were a mixture of calcium oxalate monohydrate and uric acid (30 proteins ID'd)
