# Supplementary material for: Label-free proteomic methodology for the analysis of human kidney stone matrix composition
Source: Proteome Sci. 2016 Feb 27;14:4. doi: 10.1186/s12953-016-0093-x (PMC4769560; doi:10.1186/s12953-016-0093-x)
Supplement: Additional file 4: — Ingenuity Pathway Analysis Results - Canonical Pathways associated with Kidney stone matrix proteins. (PDF 60 kb) [file 12953_2016_93_MOESM4_ESM.pdf]

Common to both stones

Canonical Pathways	-log(p-value)	Ratio	Proteins
LXR/RXR Activation	2.42E+01	25.0%	KNG1, APOE, APOA4, APOB, VTN, AMBP, C4A/C4B, PON1, HPR, ITIH4, FASN, SERPINA1, S100A8, TTR, HPX, APOM, ECHS1, SERPINF1, PCYOX1, SERPINF2, A1BG, APOL1, ALB, APOA1, ORM1, TF, ORM2, FGA, CLU, MMP9, APOD, APOC3
Coagulation System	2.39E+01	57.1%	KNG1, F12, PROC, F13A1, F2, SERPINF2, FGG, SERPIND1, PLG, F11, F10, SERPINC1, F9, SERPINA5, PROS1, F5, SERPINA1, FGB, FGA, A2M
Acute Phase Response Signaling	2.11E+01	19.3%	SERPING1, AMBP, CP, SERPINA3, F2, FGG, HNRNPK, SERPIND1, C4A/C4B, C1R, ITIH2, ITIH4, APCS, CFB, SERPINA1, FGB, HPX, TTR, C1S, SERPINF1, SERPINF2, PLG, HP, ALB, APOA1, ORM1, TF, C4BPA, ORM2, MAP2K3, HRG, FGA, A2M
FXR/RXR Activation	2.08E+01	21.7%	KNG1, APOE, APOA4, APOB, VTN, AMBP, C4A/C4B, PON1, HPR, ITIH4, FASN, SERPINA1, TTR, HPX, APOM, FETUB, SERPINF1, PCYOX1, SERPINF2, A1BG, APOL1, ALB, APOA1, ORM1, TF, ORM2, FGA, CLU, APOD, APOC3
Clathrin-mediated Endocytosis Signaling	1.68E+01	16.0%	APOE, APOA4, APOB, EGF, F2, PON1, ACTR3, SERPINA1, S100A8, SH3GLB2, ACTR2, SRC, APOM, ACTB, CLTC, RAC1, PCYOX1, APOL1, HSPA8, ITGB2, ALB, APOA1, ORM1, TF, CDC42, RAB11A, ORM2, CLU, APOD, APOC3
Intrinsic Prothrombin Activation Pathway	1.53E+01	46.7%	F11, KNG1, F10, F12, F9, SERPINC1, PROS1, F5, PROC, F13A1, FGB, FGA, F2, FGG
Epithelial Adherens Junction Signaling	1.47E+01	16.9%	RAP1B, ACTR2, SRC, MYH10, TUBA1B, MYH9, MYL6, TUBB4B, ACTB, RAC1, TUBA4A, CTNNA1, EGF, TUBB, IQGAP1, RAP1A, CDH1, ACTR3, CDC42, RHOA, TUBA1C, ACTN4, VCL, ACTN1, CTNND1
Extrinsic Prothrombin Activation Pathway	1.38E+01	61.1%	F10, F12, SERPINC1, PROS1, F5, PROC, F13A1, FGB, FGA, FGG, F2
Complement System	1.35E+01	36.8%	SERPING1, MASP2, C1S, C1QC, C1QB, C1R, C4A/C4B, ITGB2, CD55, C4BPA, CFB, C7, C8B, CFH
Production of Nitric Oxide and Reactive Oxygen Species in Macrophages	1.32E+01	14.0%	RAP1B, APOE, APOA4, APOB, PPP2R2A, PON1, RHOB, PPP1R7, SERPINA1, S100A8, PTPN6, APOM, RHOC, RAC1, PCYOX1, RAP1A, APOL1, ALB, PPP2R1A, APOA1, ORM1, RHOA, ORM2, CLU, APOC3, APOD
Remodeling of Epithelial Adherens Junctions	1.31E+01	25.0%	TUBA1B, SRC, ACTR2, NME1, TUBB4B, ACTB, TUBA4A, CTNNA1, IQGAP1, TUBB, CDH1, ACTR3, TUBA1C, VCL, ACTN4, ACTN1, CTNND1
Germ Cell-Sertoli Cell Junction Signaling	1.27E+01	14.7%	TUBA1B, RAC2, SRC, CFL1, RHOC, TUBB4B, ACTB, ILK, RAC1, TUBA4A, CTNNA1, TUBB, GSN, IQGAP1, CDH1, RHOB, CDC42, RHOA, TUBA1C, MAP2K3, ACTN4, A2M, ACTN1, CTNND1
Atherosclerosis Signaling	1.05E+01	15.3%	APOE, APOM, APOA4, APOB, PCYOX1, PRDX6, APOL1, PON1, ITGB2, ALB, APOA1, ORM1, ORM2, S100A8, SERPINA1, MMP9, CLU, APOD, APOC3
Actin Cytoskeleton Signaling	9.10E+00	10.4%	KNG1, ACTR2, MYH10, RAC2, MYH9, PFN1, MYL6, CFL1, ACTB, RAC1, EGF, TLN1, GSN, IQGAP1, F2, ACTR3, CDC42, EZR, RHOA, VCL, ACTN4, ACTN1, MSN
Glycolysis I	9.04E+00	26.8%	PGK1, ENO1, GPI, ATP, TPI1, PGAM1, PKM, GAPDH, phosphate, PFKL, NAD+
Gluconeogenesis I	8.46E+00	23.9%	PGK1, ENO1, NADPH, GPI, ATP, PGAM1, NADP, GAPDH, phosphate, MDH1, NAD+
IL-12 Signaling and Production in Macrophages	7.93E+00	12.2%	STAT6, APOE, APOM, APOA4, APOB, PCYOX1, APOL1, PON1, ALB, APOA1, ORM1, ORM2, S100A8, SERPINA1, CLU, APOD, APOC3
Integrin Signaling	7.63E+00	9.9%	RAP1B, RAC2, SRC, ACTR2, RHOC, ACTB, ILK, RAC1, TLN1, RAP1A, ITGB2, CAPNS1, ACTR3, RHOB, CDC42, ARF4, RHOA, VCL, ACTN4, ACTN1
RhoGDI Signaling	7.03E+00	10.1%	SRC, ACTR2, CFL1, MYL6, RHOC, ACTB, GNB2L1, RAC1, ARHGDIB, GNB1, CDH1, ACTR3, RHOB, CDC42, EZR, RHOA, GNB2, MSN
Regulation of Actin-based Motility by Rho	6.90E+00	14.0%	RAC2, ACTR2, PFN1, CFL1, MYL6, RHOC, ACTB, RAC1, GSN, ACTR3, RHOB, CDC42, RHOA
Sertoli Cell-Sertoli Cell Junction Signaling	6.77E+00	9.7%	TUBA1B, SRC, ATP, TUBB4B, ACTB, ILK, RAC1, TUBA4A, CTNNA1, TUBB, CDH1, CDC42, MAP2K3, TUBA1C, SPTAN1, ACTN4, A2M, ACTN1
ILK Signaling	6.70E+00	9.6%	MYH10, MYH9, CFL1, MYL6, RHOC, PPP2R2A, ACTB, ILK, ITGB2, CDH1, PPP2R1A, RHOB, CDC42, RHOA, KRT18, ACTN4, ACTN1, MMP9
EIF2 Signaling	6.70E+00	9.6%	RPL32, RPL22, RPLP1, RPS3A, RPS2, EIF2S3, RPLP0, RPS23, EIF3M, RPS6, RPS7, RPL14, UBA52, RPL23A, EIF3I, RPS27A, RPL5, RPS3
14-3-3-mediated Signaling	6.43E+00	11.8%	TUBA1B, SRC, YWHAG, YWHAE, YWHAH, TUBB4B, YWHAB, TUBA4A, TUBB, YWHAQ, TUBA1C, GFAP, SFN, PDCD6IP
Leukocyte Extravasation Signaling	6.18E+00	8.8%	RAP1B, RAC2, SRC, MYL6, ACTB, RAC1, CTNNA1, RAP1A, ITGB2, CDC42, RHOA, EZR, VCL, ACTN4, ACTN1, MMP9, CTNND1, MSN
Ephrin B Signaling	6.14E+00	14.7%	GNB1, RAC2, ABI1, CFL1, CDC42, RHOA, GNB2L1, GNB2, RAC1, CAP1, HNRNPK
IL-8 Signaling	6.05E+00	9.1%	RAC2, SRC, PLD3, RHOC, GNB2L1, RAC1, DEFA1 (includes others), EGF, IQGAP1, GNB1, ITGB2, CDH1, RHOB, RHOA, GNB2, TEK, MMP9
Signaling by Rho Family GTPases	5.88E+00	8.1%	ACTR2, SEPT9, CFL1, MYL6, RHOC, ACTB, GNB2L1, RAC1, IQGAP1, GNB1, CDH1, ACTR3, RHOB, CDC42, EZR, RHOA, GNB2, GFAP, MSN
Ephrin Receptor Signaling	5.70E+00	9.0%	RAP1B, RAC2, SRC, ACTR2, CFL1, PTPN13, GNB2L1, RAC1, EGF, RAP1A, GNB1, ACTR3, ABI1, CDC42, RHOA, GNB2
D-glucuronate Degradation I	5.68E+00	45.5%	NADPH, AKR1A1, DCXR, NADP, NAD+
HIPPO signaling	5.49E+00	12.6%	YWHAQ, PPP2R1A, SAV1, YWHAG, YWHAE, YWHAH, PPP1R7, PPP2R2A, YWHAB, phosphate, SFN
Protein Ubiquitination Pathway	5.28E+00	7.3%	B2M, ATP, PSMB5, USP5, PSME2, HSPA5, UCHL3, USP31, HSPA8, PSMD11, PSMC1, HSP90AB1, HSP90AA1, phosphate, PSMD1, PSMA3, UBA1, PSMA2, HSPB1
Methylglyoxal Degradation III	5.22E+00	27.3%	NADPH, AKR1A1, AKR1C1/AKR1C2, AKR1C3, NADP, NAD+
mTOR Signaling	5.18E+00	8.3%	PLD3, RPS3A, RHOC, PPP2R2A, RPS2, RAC1, RPS23, EIF3M, RPS6, RPS7, PPP2R1A, RHOB, RHOA, EIF3I, RPS27A, RPS3
Fcγ Receptor-mediated Phagocytosis in Macrophages and Monocytes	4.94E+00	11.1%	RAC2, SRC, ACTR2, ACTR3, PLD3, CDC42, EZR, ACTB, RAC1, RAB11A, TLN1
Primary Immunodeficiency Signaling	4.78E+00	15.4%	IGHG3, IGKC, IGLL1/IGLL5, IGLC1, IGHG4, IGHG1, IGHA1, IGHG2
p70S6K Signaling	4.68E+00	9.6%	YWHAQ, RPS6, SRC, PPP2R1A, YWHAG, YWHAE, YWHAH, PPP2R2A, YWHAB, EEF2, SFN, F2
Mevalonate Pathway I	4.66E+00	22.2%	HADHB, NADPH, ATP, NADP, phosphate, HADHA
Folate Transformations I	4.56E+00	21.4%	NADPH, ATP, NADP, phosphate, MTHFD1, NAD+
Breast Cancer Regulation by Stathmin1	4.48E+00	7.6%	TUBA1B, ATP, TUBB4B, PPP2R2A, GNB2L1, RAC1, TUBA4A, TUBB, GNB1, PPP2R1A, CDC42, PPP1R7, RHOA, GNB2, TUBA1C
Sphingosine and Sphingosine-1-phosphate	4.47E+00	27.8%	NADPH, ATP, NADP, phosphate, ASAH1
Regulation of Cellular Mechanics by Calpain Protease	4.42E+00	13.8%	SRC, CAPNS1, EZR, EGF, TLN1, VCL, ACTN4, ACTN1
Thymine Degradation	4.36E+00	40.0%	NADPH, DPYS, DPYD, NADP
NAD Phosphorylation and Dephosphorylation	4.22E+00	25.0%	NADPH, ATP, NADP, phosphate, NAD+
Neuroprotective Role of THOP1 in Alzheimer's	4.22E+00	15.2%	KNG1, PLG, ATP, YWHAE, SERPINA3, MMP9, APP
Uracil Degradation II (Reductive)	4.17E+00	36.4%	NADPH, DPYS, DPYD, NADP
ERK5 Signaling	4.11E+00	12.5%	YWHAQ, SRC, YWHAG, YWHAE, YWHAH, YWHAB, EGF, SFN
Tight Junction Signaling	4.06E+00	7.8%	MYH10, MYH9, MYL6, PPP2R2A, ACTB, RAC1, CTNNA1, NSF, PPP2R1A, CDC42, RHOA, VCL, SPTAN1
ERK/MAPK Signaling	4.04E+00	7.3%	RAP1B, RAC2, SRC, YWHAG, YWHAH, PPP2R2A, YWHAB, RAC1, TLN1, RAP1A, YWHAQ, PPP2R1A, PPP1R7, HSPB1
Superpathway of Geranylgeranyldiphosphate Biosynthesis I (via Mevalonate)	3.98E+00	17.1%	HADHB, NADPH, ATP, NADP, phosphate, HADHA
Hematopoiesis from Pluripotent Stem Cells	3.92E+00	13.7%	IGHG3, IGKC, IGLC1, IGHG4, IGHG1, IGHA1, IGHG2
Macropinocytosis Signaling	3.92E+00	11.8%	ITGB2, SRC, ABI1, CDC42, RHOA, RAC1, EGF, ACTN4

Colanic Acid Building Blocks Biosynthesis	3.91E+00	16.7%	NADPH, GPI, ATP, UGP2, NADP, NAD+
Lysine Degradation V	3.91E+00	21.7%	NADPH, ATP, NADP, phosphate, NAD+
Regulation of eIF4 and p70S6K Signaling	3.90E+00	8.0%	RPS6, RPS7, PPP2R1A, RPS3A, PPP2R2A, RPS2, EIF3I, RPS27A, EIF2S3, RPS3, RPS23, EIF3M
Phospholipase C Signaling	3.90E+00	6.5%	RAP1B, SRC, PLD3, MYL6, RHOC, GNB2L1, RAC1, IGHG1, RAP1A, GNB1, IGHG3, RHOB, RHOA, GNB2, IGHG4, IGHG2
PI3K/AKT Signaling	3.90E+00	8.6%	YWHAQ, PPP2R1A, YWHAG, YWHAE, YWHAH, HSP90AB1, PPP2R2A, YWHAB, ILK, HSP90AA1, SFN
Arginine Biosynthesis IV	3.82E+00	20.8%	NADPH, ATP, ASS1, NADP, phosphate
Proline Biosynthesis I	3.71E+00	28.6%	NADPH, ATP, NADP, phosphate
Actin Nucleation by ARP-WASP Complex	3.66E+00	12.5%	ACTR2, ACTR3, RHOB, CDC42, RHOC, RHOA, RAC1
Caveolar-mediated Endocytosis Signaling	3.66E+00	10.8%	B2M, ITGB2, SRC, ALB, CD55, ARCN1, ACTB, EGF
Virus Entry via Endocytic Pathways	3.61E+00	9.5%	B2M, ITGB2, RAC2, SRC, CD55, CDC42, ACTB, CLTC, RAC1
VEGF Signaling	3.54E+00	9.3%	SRC, PTPN6, YWHAE, ACTB, EIF2S3, VCL, ACTN4, SFN, ACTN1
Tryptophan Degradation X (Mammalian, via Tryptamine)	3.49E+00	17.9%	NADPH, AKR1A1, NADP, ALDH9A1, NAD+
Oxidative Ethanol Degradation III	3.49E+00	17.9%	NADPH, ATP, NADP, ALDH9A1, NAD+
Fatty Acid β-oxidation I	3.47E+00	14.0%	HADHB, ATP, ECHS1, HSD17B4, HADHA, NAD+
Citrulline-Nitric Oxide Cycle	3.47E+00	25.0%	NADPH, ATP, ASS1, NADP
Glutathione Redox Reactions II	3.46E+00	42.9%	GSR, NADPH, NADP
Paxillin Signaling	3.38E+00	8.8%	ITGB2, SRC, CDC42, ACTB, RAC1, TLN1, VCL, ACTN4, ACTN1
RhoA Signaling	3.37E+00	8.1%	ACTR2, ACTR3, PFN1, SEPT9, CFL1, MYL6, EZR, ACTB, RHOA, MSN
Tryptophan Degradation III (Eukaryotic)	3.36E+00	13.3%	HADHB, NADPH, NADP, HSD17B4, HADHA, NAD+
NADH Repair	3.26E+00	37.5%	ATP, GAPDH, phosphate
Glycogen Degradation III	3.26E+00	22.2%	GAA, phosphate, PYGB, PYGL
Protein Kinase A Signaling	3.24E+00	5.0%	RAP1B, MYH10, PTPN6, ATP, YWHAG, YWHAH, MYL6, YWHAE, YWHAB, PTPN13, GNB2L1, PYGL, PYGB, RAP1A, GNB1, YWHAQ, PPP1R7, RHOA, GNB2, SFN
Bile Acid Biosynthesis, Neutral Pathway	3.16E+00	12.2%	NADPH, ATP, AKR1C1/AKR1C2, AKR1C3, NADP, NAD+
Cell Cycle: G2/M DNA Damage Checkpoint Regulation	3.16E+00	12.2%	YWHAQ, YWHAG, YWHAE, YWHAH, YWHAB, SFN
B Cell Receptor Signaling	3.16E+00	6.6%	RAP1B, RAC2, PTPN6, IGHG3, CFL1, CDC42, IGHG4, RAC1, MAP2K3, IGHG1, RAP1A, IGHG2
Axonal Guidance Signaling	3.10E+00	4.8%	RAP1B, TUBA1B, ACTR2, RAC2, PFN1, MYL6, CFL1, TUBB4B, GNB2L1, RAC1, TUBA4A, EGF, TUBB, RAP1A, GNB1, ACTR3, CDC42, RHOA, GNB2, TUBA1C, MMP9
Role of Tissue Factor in Cancer	3.08E+00	8.0%	F10, SRC, CFL1, CDC42, RAC1, FGB, FGA, FGG, F2
used P<0.001			
