# Supplementary material for: Label-free proteomic methodology for the analysis of human kidney stone matrix composition
Source: Proteome Sci. 2016 Feb 27;14:4. doi: 10.1186/s12953-016-0093-x (PMC4769560; doi:10.1186/s12953-016-0093-x)
Supplement: Additional file 5: — Functional Annotation Clustering of kidney stone matrix proteins obtained from the DAVID database. (PDF 100 kb) [file 12953_2016_93_MOESM5_ESM.pdf]

Annotation Cluster 1		Enrichment Score: 14.6	Count	P_Value	Benjamini
	GOTERM_BP_FAT	response to wounding	67	3.10E-23	1.90E-20
	GOTERM_BP_FAT	defense response	60	2.70E-15	4.30E-13
	GOTERM_BP_FAT	proteolysis	59	7.00E-06	3.60E-04
	GOTERM_BP_FAT	immune response	54	4.20E-10	3.60E-08
	SP_PIR_KEYWORDS	plasma	48	1.70E-51	4.40E-49
	GOTERM_BP_FAT	inflammatory response	45	4.30E-17	9.50E-15
	KEGG_PATHWAY	Complement and coagulation cascades	40	2.20E-36	2.90E-34
	GOTERM_BP_FAT	acute inflammatory response	36	1.50E-28	3.70E-25
	GOTERM_BP_FAT	innate immune response	31	9.90E-18	2.60E-15
	GOTERM_BP_FAT	positive regulation of immune system process	31	3.80E-11	3.80E-09
	GOTERM_BP_FAT	protein maturation	29	2.70E-17	6.60E-15
	GOTERM_BP_FAT	immune effector process	29	3.70E-16	6.20E-14
	SP_PIR_KEYWORDS	immune response	28	5.30E-12	1.60E-10
	GOTERM_BP_FAT	positive regulation of response to stimulus	28	3.30E-09	2.70E-07
	GOTERM_BP_FAT	protein maturation by peptide bond cleavage	27	2.20E-19	1.00E-16
	GOTERM_BP_FAT	protein processing	27	2.80E-16	6.70E-14
	GOTERM_BP_FAT	humoral immune response	26	3.10E-19	1.20E-16
	GOTERM_BP_FAT	positive regulation of immune response	26	1.60E-12	1.70E-10
	GOTERM_BP_FAT	complement activation	24	1.90E-24	2.20E-21
	GOTERM_BP_FAT	activation of plasma proteins involved in acute inflammatory response	24	3.90E-24	3.10E-21
	GOTERM_BP_FAT	activation of immune response	24	4.90E-15	7.30E-13
	SP_PIR_KEYWORDS	innate immunity	23	5.10E-18	2.20E-16
	GOTERM_BP_FAT	lymphocyte mediated immunity	22	9.00E-16	1.50E-13
	GOTERM_BP_FAT	leukocyte mediated immunity	22	8.10E-14	1.00E-11
	KEGG_PATHWAY	Systemic lupus erythematosus	21	4.60E-09	3.00E-07
	GOTERM_BP_FAT	adaptive immune response	20	1.00E-12	1.10E-10
	GOTERM_BP_FAT	adaptive immune response based on somatic recombination of immune re	20	1.00E-12	1.10E-10
	GOTERM_BP_FAT	immunoglobulin mediated immune response	19	1.20E-14	1.80E-12
	GOTERM_BP_FAT	B cell mediated immunity	19	2.60E-14	3.50E-12
	SP_PIR_KEYWORDS	complement pathway	18	8.00E-21	5.10E-19
	GOTERM_BP_FAT	complement activation, classical pathway	18	4.40E-19	1.50E-16
	GOTERM_BP_FAT	humoral immune response mediated by circulating immunoglobulin	18	2.10E-18	6.30E-16
	SP_PIR_KEYWORDS	sushi	14	5.20E-10	1.30E-08
	INTERPRO	Sushi/SCR/CCP	14	2.10E-09	3.50E-07
	INTERPRO	Complement control module	14	2.60E-09	3.70E-07
	SMART	CCP	14	2.80E-09	4.40E-07
	UP_SEQ_FEATURE	domain:Sushi 1	13	3.40E-10	6.30E-08
	UP_SEQ_FEATURE	domain:Sushi 2	13	3.40E-10	6.30E-08
	BIOCARTA	Complement Pathway	12	2.20E-09	3.70E-07
	GOTERM_BP_FAT	complement activation, alternative pathway	10	8.80E-11	8.10E-09
	BIOCARTA	Classical Complement Pathway	10	1.00E-08	8.30E-07
	BIOCARTA	Lectin Induced Complement Pathway	9	3.00E-07	1.70E-05
	UP_SEQ_FEATURE	domain:Sushi 3	7	1.00E-04	7.60E-03
	UP_SEQ_FEATURE	domain:Sushi 4	5	3.70E-03	1.30E-01
Annotation Cluster 2		Enrichment Score: 14.34	Count	P_Value	Benjamini
	GOTERM_MF_FAT	structural molecule activity	65	1.80E-17	1.20E-14
	SP_PIR_KEYWORDS	coiled coil	63	2.40E-02	9.50E-02
	GOTERM_CC_FAT	cytoskeletal part	57	3.60E-06	5.00E-05
	GOTERM_CC_FAT	intermediate filament	27	1.10E-10	3.40E-09
	GOTERM_CC_FAT	intermediate filament cytoskeleton	27	1.70E-10	5.10E-09
	SP_PIR_KEYWORDS	Intermediate filament	26	7.80E-22	5.70E-20
	UP_SEQ_FEATURE	region of interest:Linker 12	25	7.80E-23	1.40E-19
	UP_SEQ_FEATURE	region of interest:Coil 1A	25	9.50E-22	8.70E-19
	UP_SEQ_FEATURE	region of interest:Coil 1B	25	9.50E-22	8.70E-19
	UP_SEQ_FEATURE	region of interest:Linker 1	25	9.50E-22	8.70E-19
	UP_SEQ_FEATURE	region of interest:Rod	25	1.40E-21	8.60E-19
	UP_SEQ_FEATURE	region of interest:Head	25	3.00E-21	1.10E-18
	UP_SEQ_FEATURE	region of interest:Coil 2	24	1.90E-21	9.00E-19
	UP_SEQ_FEATURE	region of interest:Tail	24	1.20E-19	3.70E-17
	INTERPRO	Filament	24	4.50E-19	3.80E-16
	INTERPRO	Intermediate filament protein	24	6.40E-19	2.70E-16
	PIR_SUPERFAMILY	PIRSF002282:cytoskeletal keratin	24	1.00E-16	3.00E-14
	SP_PIR_KEYWORDS	keratin	24	5.50E-13	1.70E-11
	INTERPRO	Intermediate filament protein, conserved site	23	7.90E-18	2.20E-15
	GOTERM_MF_FAT	structural constituent of cytoskeleton	20	6.20E-13	1.30E-10
	GOTERM_CC_FAT	keratin filament	19	2.00E-10	5.40E-09
	GOTERM_BP_FAT	ectoderm development	18	1.30E-04	4.60E-03
	GOTERM_BP_FAT	epidermis development	16	5.10E-04	1.50E-02
	UP_SEQ_FEATURE	site:Stutter	14	2.80E-13	6.50E-11
	INTERPRO	Type II keratin	13	2.50E-12	5.30E-10
	INTERPRO	Keratin, type I	10	1.40E-07	1.50E-05
Annotation Cluster 3		Enrichment Score: 12.97	Count	P_Value	Benjamini
	SP_PIR_KEYWORDS	glycoprotein	150	4.50E-07	5.10E-06
	SP_PIR_KEYWORDS	signal	148	8.40E-16	3.50E-14
	UP_SEQ_FEATURE	signal peptide	148	1.50E-15	4.10E-13
	UP_SEQ_FEATURE	glycosylation site:N-linked (GlcNAc...)	135	7.60E-05	6.60E-03
	GOTERM_CC_FAT	extracellular region	130	5.00E-17	6.30E-15
	SP_PIR_KEYWORDS	disulfide bond	121	6.80E-10	1.60E-08
	UP_SEQ_FEATURE	disulfide bond	119	4.50E-10	7.50E-08
	SP_PIR_KEYWORDS	Secreted	110	1.50E-22	1.30E-20
	GOTERM_CC_FAT	extracellular region part	79	3.20E-15	2.00E-13
	GOTERM_CC_FAT	extracellular space	71	5.40E-19	1.00E-16
Annotation Cluster 4		Enrichment Score: 12.4	Count	P_Value	Benjamini
	KEGG_PATHWAY	Complement and coagulation cascades	40	2.20E-36	2.90E-34
	GOTERM_BP_FAT	wound healing	27	1.40E-10	1.30E-08
	GOTERM_BP_FAT	hemostasis	24	1.20E-13	1.50E-11
	GOTERM_BP_FAT	regulation of body fluid levels	24	4.20E-11	4.00E-09
	GOTERM_BP_FAT	blood coagulation	23	3.20E-13	3.60E-11
	GOTERM_BP_FAT	coagulation	23	3.20E-13	3.60E-11
	SP_PIR_KEYWORDS	blood coagulation	20	8.50E-20	4.80E-18
	BIOCARTA	Intrinsic Prothrombin Activation Pathway	12	2.20E-09	3.70E-07
	BIOCARTA	Extrinsic Prothrombin Activation Pathway	7	5.30E-05	1.70E-03
	BIOCARTA	Acute Myocardial Infarction	7	1.70E-04	4.80E-03
	SP_PIR_KEYWORDS	thrombophilia	6	1.50E-07	2.20E-06
Annotation Cluster 5		Enrichment Score: 10.52	Count	P_Value	Benjamini
	GOTERM_CC_FAT	membrane-bounded vesicle	51	3.30E-11	1.30E-09

	GOTERM_CC_FAT	vesicle	51	1.00E-08	2.00E-07
	GOTERM_CC_FAT	cytoplasmic membrane-bounded vesicle	50	3.50E-11	1.20E-09
	GOTERM_CC_FAT	cytoplasmic vesicle	50	6.90E-09	1.40E-07
	GOTERM_CC_FAT	cytoplasmic vesicle part	23	1.00E-07	1.70E-06
	GOTERM_CC_FAT	secretory granule	22	2.30E-07	3.70E-06
	GOTERM_CC_FAT	vesicle lumen	19	7.50E-16	7.30E-14
	GOTERM_CC_FAT	pigment granule	19	2.40E-10	6.10E-09
	GOTERM_CC_FAT	melanosome	19	2.40E-10	6.10E-09
	GOTERM_CC_FAT	platelet alpha granule lumen	18	1.60E-15	1.30E-13
	GOTERM_CC_FAT	cytoplasmic membrane-bounded vesicle lumen	18	6.50E-15	3.50E-13
	GOTERM_CC_FAT	platelet alpha granule	18	6.60E-13	3.10E-11
Annotation Cluster 6		Enrichment Score: 8.47	Count	P_Value	Benjamini
	GOTERM_MF_FAT	calcium ion binding	55	3.60E-06	2.30E-04
	SP_PIR_KEYWORDS	calcium	45	2.90E-07	3.30E-06
	SP_PIR_KEYWORDS	calcium binding	22	3.80E-14	1.40E-12
Annotation Cluster 7		Enrichment Score: 8.16	Count	P_Value	Benjamini
	SP_PIR_KEYWORDS	isopeptide bond	32	4.00E-11	1.00E-09
	UP_SEQ_FEATURE	cross-link:Glycyl lysine isopeptide (Lys-Gly) (interchain with G-Cter in ubiqu	25	9.20E-11	1.90E-08
	SP_PIR_KEYWORDS	ubl conjugation	31	8.80E-05	6.30E-04
Annotation Cluster 8		Enrichment Score: 7.84	Count	P_Value	Benjamini
	GOTERM_CC_FAT	non-membrane-bounded organelle	132	2.90E-09	6.60E-08
	GOTERM_CC_FAT	intracellular non-membrane-bounded organelle	132	2.90E-09	6.60E-08
	GOTERM_CC_FAT	cytoskeleton	85	1.50E-09	3.50E-08
	GOTERM_CC_FAT	cytoskeletal part	57	3.60E-06	5.00E-05
Annotation Cluster 9		Enrichment Score: 7.55	Count	P_Value	Benjamini
	GOTERM_MF_FAT	enzyme inhibitor activity	35	2.80E-12	3.60E-10
	GOTERM_MF_FAT	endopeptidase inhibitor activity	27	3.20E-13	1.00E-10
	GOTERM_MF_FAT	peptidase inhibitor activity	27	1.20E-12	1.90E-10
	GOTERM_MF_FAT	serine-type endopeptidase inhibitor activity	18	2.70E-09	2.80E-07
	SP_PIR_KEYWORDS	protease inhibitor	16	3.80E-08	7.00E-07
	SP_PIR_KEYWORDS	Serine protease inhibitor	14	5.20E-08	9.10E-07
	INTERPRO	Protease inhibitor I4, serpin	11	7.20E-08	8.70E-06
	SMART	SERPIN	11	9.20E-08	7.30E-06
	PIR_SUPERFAMILY	PIRSF001630:serpin	9	5.30E-05	4.80E-03
	SP_PIR_KEYWORDS	serine proteinase inhibitor	8	5.30E-07	5.90E-06
	UP_SEQ_FEATURE	site:Reactive bond	8	9.20E-05	7.30E-03
	GOTERM_MF_FAT	protease binding	5	2.40E-03	4.20E-02
Annotation Cluster 10		Enrichment Score: 6.3	Count	P_Value	Benjamini
	SP_PIR_KEYWORDS	P-loop	16	1.20E-07	1.70E-06
	SP_PIR_KEYWORDS	nucleotide binding	15	2.60E-07	3.20E-06
	SP_PIR_KEYWORDS	GTP binding	11	4.10E-06	3.50E-05
Annotation Cluster 11		Enrichment Score: 5.09	Count	P_Value	Benjamini
	GOTERM_CC_FAT	ribonucleoprotein complex	35	3.60E-05	4.40E-04
	GOTERM_CC_FAT	cytosolic part	26	9.40E-12	4.00E-10
	GOTERM_BP_FAT	translation	25	7.90E-05	3.10E-03
	SP_PIR_KEYWORDS	protein biosynthesis	22	5.00E-09	1.00E-07
	SP_PIR_KEYWORDS	ribonucleoprotein	21	1.40E-05	1.10E-04
	GOTERM_BP_FAT	translational elongation	18	9.20E-09	7.40E-07
	GOTERM_CC_FAT	cytosolic ribosome	16	2.70E-08	5.20E-07
	GOTERM_CC_FAT	ribosomal subunit	16	1.20E-05	1.60E-04
	GOTERM_CC_FAT	ribosome	16	3.30E-03	2.50E-02
	KEGG_PATHWAY	Ribosome	15	1.70E-05	7.60E-04
	SP_PIR_KEYWORDS	ribosomal protein	15	1.80E-04	1.20E-03
	GOTERM_MF_FAT	structural constituent of ribosome	15	7.30E-04	2.00E-02
	SP_PIR_KEYWORDS	ribosome	13	1.50E-07	2.20E-06
	GOTERM_CC_FAT	cytosolic small ribosomal subunit	9	2.80E-05	3.70E-04
	GOTERM_CC_FAT	small ribosomal subunit	9	7.60E-04	7.20E-03
	GOTERM_CC_FAT	cytosolic large ribosomal subunit	7	1.10E-03	9.40E-03
	GOTERM_CC_FAT	large ribosomal subunit	7	1.90E-02	1.00E-01
Annotation Cluster 12		Enrichment Score: 5.08	Count	P_Value	Benjamini
	SP_PIR_KEYWORDS	hydrolase	67	3.40E-06	3.10E-05
	GOTERM_MF_FAT	peptidase activity	34	4.60E-04	1.60E-02
	GOTERM_MF_FAT	peptidase activity, acting on L-amino acid peptides	32	9.30E-04	2.50E-02
	SP_PIR_KEYWORDS	Protease	28	4.50E-05	3.50E-04
	GOTERM_MF_FAT	endopeptidase activity	25	6.40E-04	2.00E-02
	GOTERM_MF_FAT	serine-type endopeptidase activity	19	1.30E-06	1.00E-04
	GOTERM_MF_FAT	serine-type peptidase activity	19	1.00E-05	6.00E-04
	GOTERM_MF_FAT	serine hydrolase activity	19	1.20E-05	6.40E-04
	SP_PIR_KEYWORDS	serine proteinase	15	2.20E-11	6.00E-10
	UP_SEQ_FEATURE	domain:Peptidase S1	15	2.50E-07	3.30E-05
	INTERPRO	Peptidase S1 and S6, chymotrypsin/Hap	15	2.70E-06	2.30E-04
	SMART	Tryp_SPc	15	3.60E-06	1.40E-04
	SP_PIR_KEYWORDS	Serine protease	15	4.90E-06	4.20E-05
	UP_SEQ_FEATURE	active site:Charge relay system	15	2.90E-04	1.90E-02
	SP_PIR_KEYWORDS	zymogen	15	4.60E-04	2.80E-03
	INTERPRO	Peptidase S1A, chymotrypsin	14	4.20E-06	3.00E-04
	INTERPRO	Peptidase S1/S6, chymotrypsin/Hap, active site	14	4.70E-06	2.80E-04
Annotation Cluster 13		Enrichment Score: 5	Count	P_Value	Benjamini
	GOTERM_BP_FAT	monosaccharide metabolic process	21	1.50E-05	7.10E-04
	GOTERM_BP_FAT	generation of precursor metabolites and energy	20	3.40E-03	6.80E-02
	GOTERM_BP_FAT	hexose metabolic process	19	2.30E-05	1.00E-03
	GOTERM_BP_FAT	glucose metabolic process	18	4.20E-06	2.30E-04
	GOTERM_BP_FAT	carbohydrate catabolic process	16	1.00E-06	6.60E-05
	GOTERM_BP_FAT	cellular carbohydrate catabolic process	15	2.50E-07	1.70E-05
	GOTERM_BP_FAT	alcohol catabolic process	13	5.70E-06	3.10E-04
	GOTERM_BP_FAT	glucose catabolic process	12	1.10E-06	7.10E-05
	GOTERM_BP_FAT	hexose catabolic process	12	6.80E-06	3.60E-04
	GOTERM_BP_FAT	monosaccharide catabolic process	12	9.00E-06	4.40E-04
	KEGG_PATHWAY	Glycolysis / Gluconeogenesis	12	4.10E-05	9.00E-04
	GOTERM_BP_FAT	glycolysis	11	1.20E-06	7.00E-05
	SP_PIR_KEYWORDS	glycolysis	10	8.00E-07	8.70E-06
	BIOCARTA	Glycolysis Pathway	7	5.30E-05	1.70E-03
	SP_PIR_KEYWORDS	gluconeogenesis	5	9.40E-04	5.30E-03
Annotation Cluster 14		Enrichment Score: 4.92	Count	P_Value	Benjamini
	SP_PIR_KEYWORDS	cytoskeleton	39	2.30E-07	2.90E-06
	GOTERM_BP_FAT	cytoskeleton organization	34	1.40E-06	8.30E-05
	SP_PIR_KEYWORDS	actin-binding	21	2.20E-06	2.20E-05

	GOTERM_BP_FAT	actin filament-based process	22	1.50E-05	7.10E-04
	GOTERM_BP_FAT	actin cytoskeleton organization	21	2.00E-05	8.70E-04
	GOTERM_MF_FAT	actin binding	26	2.90E-05	1.40E-03
	GOTERM_MF_FAT	cytoskeletal protein binding	32	2.20E-04	8.70E-03
	GOTERM_CC_FAT	actin cytoskeleton	21	3.30E-04	3.50E-03
Annotation Cluster 15		Enrichment Score: 4.6	Count	P_Value	Benjamini
	BIOCARTA	Intrinsic Prothrombin Activation Pathway	12	2.20E-09	3.70E-07
	SP_PIR_KEYWORDS	thrombophilia	6	1.50E-07	2.20E-06
	SP_PIR_KEYWORDS	gamma-carboxyglutamic acid	8	1.70E-07	2.30E-06
	SP_PIR_KEYWORDS	carboxyglutamic acid	6	8.90E-07	9.50E-06
	INTERPRO	Coagulation factor, Gla region	6	9.20E-06	5.20E-04
	SP_PIR_KEYWORDS	vitamin K	5	1.10E-05	8.90E-05
	UP_SEQ_FEATURE	domain:Gla	6	1.30E-05	1.30E-03
	INTERPRO	Gamma-carboxyglutamic acid-rich (GLA) domain	6	2.20E-05	1.10E-03
	SMART	GLA	6	2.60E-05	6.80E-04
	SP_PIR_KEYWORDS	beta-hydroxyaspartic acid	5	3.70E-05	2.90E-04
	BIOCARTA	Extrinsic Prothrombin Activation Pathway	7	5.30E-05	1.70E-03
	INTERPRO	EGF-type aspartate/asparagine hydroxylation conserved site	12	5.70E-05	2.70E-03
	BIOCARTA	Acute Myocardial Infarction	7	1.70E-04	4.80E-03
	INTERPRO	Peptidase S1A, coagulation factor VII/IX/X/C/Z	4	1.80E-04	7.10E-03
	INTERPRO	EGF	12	6.30E-04	1.90E-02
	PIR_SUPERFAMILY	PIRSF001143:Factor_X	4	7.10E-04	3.20E-02
	PIR_SUPERFAMILY	PIRSF001143:coagulation factor X	4	7.10E-04	3.20E-02
	INTERPRO	Coagulation factor, subset, Gla region	4	1.40E-03	3.50E-02
	UP_SEQ_FEATURE	domain:EGF-like 2	8	5.30E-03	1.70E-01
Annotation Cluster 16		Enrichment Score: 4.39	Count	P_Value	Benjamini
	GOTERM_MF_FAT	unfolded protein binding	17	4.80E-07	4.40E-05
	GOTERM_BP_FAT	protein folding	16	3.40E-04	1.10E-02
	SP_PIR_KEYWORDS	Chaperone	13	4.30E-04	2.70E-03
Annotation Cluster 17		Enrichment Score: 4.24	Count	P_Value	Benjamini
	SP_PIR_KEYWORDS	glycation	7	7.60E-08	1.20E-06
	UP_SEQ_FEATURE	glycosylation site:N-linked (Glc) (glycation)	6	4.20E-07	5.10E-05
	UP_SEQ_FEATURE	site:Not glycated	3	8.10E-03	2.40E-01
	GOTERM_MF_FAT	oxygen binding	5	4.30E-02	3.60E-01
Annotation Cluster 18		Enrichment Score: 3.73	Count	P_Value	Benjamini
	UP_SEQ_FEATURE	site:Interaction with phosphoserine on interacting protein	6	1.60E-07	2.30E-05
	INTERPRO	14-3-3 protein	6	2.70E-07	2.60E-05
	SMART	14_3_3	6	3.20E-07	1.70E-05
	PIR_SUPERFAMILY	PIRSF000868:14-3-3	6	2.60E-06	3.60E-04
	PIR_SUPERFAMILY	PIRSF000868:14-3-3 protein	6	2.60E-06	3.60E-04
	KEGG_PATHWAY	Neurotrophin signaling pathway	11	4.10E-02	2.80E-01
	GOTERM_MF_FAT	protein domain specific binding	14	2.30E-01	8.20E-01
	KEGG_PATHWAY	Oocyte meiosis	6	5.20E-01	9.20E-01
	KEGG_PATHWAY	Cell cycle	6	6.40E-01	9.40E-01
Annotation Cluster 19		Enrichment Score: 3.7	Count	P_Value	Benjamini
	SP_PIR_KEYWORDS	hydroxylation	15	1.10E-09	2.40E-08
	SP_PIR_KEYWORDS	egf-like domain	22	1.70E-07	2.30E-06
	INTERPRO	EGF-like region, conserved site	24	4.50E-06	2.90E-04
	SP_PIR_KEYWORDS	vitamin K	5	1.10E-05	8.90E-05
	SP_PIR_KEYWORDS	beta-hydroxyaspartic acid	5	3.70E-05	2.90E-04
	INTERPRO	EGF-type aspartate/asparagine hydroxylation conserved site	12	5.70E-05	2.70E-03
	INTERPRO	EGF-like calcium-binding	11	2.50E-04	8.90E-03
	INTERPRO	EGF-like calcium-binding, conserved site	11	2.50E-04	8.90E-03
	SMART	EGF_CA	11	3.10E-04	6.20E-03
	INTERPRO	EGF	12	6.30E-04	1.90E-02
	UP_SEQ_FEATURE	domain:EGF-like 1	11	6.50E-04	3.60E-02
	INTERPRO	EGF-like, type 3	15	7.70E-04	2.20E-02
	INTERPRO	EGF-like	14	3.10E-03	6.40E-02
	SMART	EGF	14	3.90E-03	4.30E-02
	UP_SEQ_FEATURE	domain:EGF-like 2	8	5.30E-03	1.70E-01
	INTERPRO	EGF calcium-binding	7	1.30E-02	1.80E-01
	UP_SEQ_FEATURE	domain:EGF-like 2; calcium-binding	5	4.80E-02	7.00E-01
	UP_SEQ_FEATURE	domain:EGF-like 3; calcium-binding	4	5.90E-02	7.50E-01
Annotation Cluster 20		Enrichment Score: 3.67	Count	P_Value	Benjamini
	SP_PIR_KEYWORDS	heparin-binding	11	3.10E-06	2.90E-05
	GOTERM_MF_FAT	heparin binding	13	7.90E-05	3.60E-03
	UP_SEQ_FEATURE	region of interest:Heparin-binding	6	9.10E-05	7.60E-03
	GOTERM_MF_FAT	carbohydrate binding	24	6.70E-04	2.00E-02
	GOTERM_MF_FAT	pattern binding	14	1.00E-03	2.30E-02
	GOTERM_MF_FAT	polysaccharide binding	14	1.00E-03	2.30E-02
	GOTERM_MF_FAT	glycosaminoglycan binding	13	1.40E-03	2.90E-02
Annotation Cluster 21		Enrichment Score: 3.66	Count	P_Value	Benjamini
	BIOCARTA	Classical Complement Pathway	10	1.00E-08	8.30E-07
	BIOCARTA	Lectin Induced Complement Pathway	9	3.00E-07	1.70E-05
	INTERPRO	Anaphylatoxin/fibulin	5	1.70E-05	8.90E-04
	SMART	ANATO	5	1.90E-05	6.00E-04
	SP_PIR_KEYWORDS	inflammation	7	2.20E-05	1.70E-04
	UP_SEQ_FEATURE	domain:Anaphylatoxin-like	4	5.40E-05	5.00E-03
	INTERPRO	Alpha-2-macroglobulin, N-terminal	5	1.50E-04	6.10E-03
	INTERPRO	Alpha-2-macroglobulin, N-terminal 2	5	1.50E-04	6.10E-03
	INTERPRO	Complement C3a/C4a/C5a anaphylatoxin	4	1.80E-04	7.10E-03
	INTERPRO	Anaphylatoxin	4	1.80E-04	7.10E-03
	PIR_SUPERFAMILY	PIRSF001635:alpha-2-macroglobulin	5	1.90E-04	1.30E-02
	INTERPRO	Alpha-2-macroglobulin	5	2.10E-04	7.80E-03
	INTERPRO	Alpha-macroglobulin, receptor-binding	5	2.10E-04	7.80E-03
	INTERPRO	Alpha-2-macroglobulin, conserved site	5	2.10E-04	7.80E-03
	INTERPRO	A-macroglobulin complement component	5	2.10E-04	7.80E-03
	SP_PIR_KEYWORDS	thioester bond	4	2.60E-04	1.70E-03
	UP_SEQ_FEATURE	cross-link:Isoglutamyl cysteine thioester (Cys-Gln)	4	2.60E-04	1.80E-02
	INTERPRO	Alpha-2-macroglobulin, thiol-ester bond-forming	4	2.00E-03	4.40E-02
	INTERPRO	Netrin module, non-TIMP type	4	9.90E-03	1.50E-01
	SP_PIR_KEYWORDS	inflammatory response	7	1.00E-02	4.80E-02
	SP_PIR_KEYWORDS	thiolester bond	5	1.00E-02	4.80E-02
	SMART	C345C	4	1.10E-02	9.60E-02
	UP_SEQ_FEATURE	domain:NTR	4	1.50E-02	3.70E-01
	INTERPRO	Netrin domain	4	2.30E-02	2.70E-01
Annotation Cluster 22		Enrichment Score: 3.51	Count	P_Value	Benjamini

	GOTERM_BP_FAT	macromolecular complex assembly	41	3.10E-05	1.30E-03
	GOTERM_BP_FAT	cellular macromolecular complex assembly	25	4.20E-05	1.70E-03
	GOTERM_BP_FAT	protein polymerization	9	1.00E-04	3.90E-03
	GOTERM_BP_FAT	macromolecular complex subunit organization	41	1.30E-04	4.60E-03
	GOTERM_BP_FAT	cellular protein complex assembly	16	1.30E-04	4.50E-03
	GOTERM_BP_FAT	cellular macromolecular complex subunit organization	25	2.50E-04	7.90E-03
	GOTERM_BP_FAT	protein complex assembly	26	1.20E-02	1.70E-01
	GOTERM_BP_FAT	protein complex biogenesis	26	1.20E-02	1.70E-01
Annotation Cluster 23		Enrichment Score: 3.46	Count	P_Value	Benjamini
	INTERPRO	Chaperonin TCP-1, conserved site	5	9.40E-05	4.20E-03
	INTERPRO	Chaperone, tailless complex polypeptide 1	5	1.50E-04	6.10E-03
	SP_PIR_KEYWORDS	Chaperone	13	4.30E-04	2.70E-03
	PIR_SUPERFAMILY	PIRSF002584:molecular chaperone t-complex-type	5	5.50E-04	2.90E-02
	INTERPRO	Chaperonin Cpn60/TCP-1	5	5.50E-04	1.80E-02
	GOTERM_CC_FAT	chaperonin-containing T-complex	4	9.80E-04	8.60E-03
Annotation Cluster 24		Enrichment Score: 3.41	Count	P_Value	Benjamini
	GOTERM_CC_FAT	high-density lipoprotein particle	9	6.00E-07	9.50E-06
	SP_PIR_KEYWORDS	hdl	7	2.60E-06	2.50E-05
	GOTERM_BP_FAT	regulation of lipoprotein oxidation	3	2.70E-03	5.80E-02
	GOTERM_BP_FAT	negative regulation of lipoprotein oxidation	3	2.70E-03	5.80E-02
	GOTERM_BP_FAT	negative regulation of lipoprotein metabolic process	3	1.30E-02	1.80E-01
	GOTERM_BP_FAT	regulation of lipoprotein metabolic process	3	2.30E-02	2.70E-01
Annotation Cluster 25		Enrichment Score: 3.36	Count	P_Value	Benjamini
	KEGG_PATHWAY	Adherens junction	14	2.00E-05	6.80E-04
	KEGG_PATHWAY	Leukocyte transendothelial migration	16	1.50E-04	2.80E-03
	KEGG_PATHWAY	Focal adhesion	19	2.50E-03	3.50E-02
	KEGG_PATHWAY	Regulation of actin cytoskeleton	19	5.10E-03	5.90E-02
Annotation Cluster 26		Enrichment Score: 3.23	Count	P_Value	Benjamini
	GOTERM_BP_FAT	regulation of blood coagulation	11	7.70E-08	5.60E-06
	GOTERM_BP_FAT	negative regulation of blood coagulation	9	2.30E-07	1.60E-05
	GOTERM_BP_FAT	regulation of coagulation	11	3.00E-07	2.00E-05
	GOTERM_BP_FAT	negative regulation of coagulation	9	6.70E-07	4.30E-05
	GOTERM_BP_FAT	regulation of response to external stimulus	17	2.80E-05	1.20E-03
	GOTERM_BP_FAT	regulation of fibrinolysis	5	9.50E-05	3.60E-03
	GOTERM_BP_FAT	positive regulation of blood coagulation	5	1.50E-04	5.00E-03
	GOTERM_BP_FAT	fibrinolysis	5	3.50E-04	1.10E-02
	SP_PIR_KEYWORDS	fibrinolysis	4	4.40E-04	2.70E-03
	GOTERM_BP_FAT	positive regulation of coagulation	5	4.90E-04	1.40E-02
	GOTERM_BP_FAT	positive regulation of fibrinolysis	3	5.30E-03	9.60E-02
	GOTERM_BP_FAT	negative regulation of multicellular organismal process	12	1.20E-02	1.70E-01
	GOTERM_BP_FAT	negative regulation of fibrinolysis	3	1.30E-02	1.80E-01
	INTERPRO	Kringle, subgroup	3	6.70E-02	5.10E-01
	SP_PIR_KEYWORDS	kringle	3	6.80E-02	2.20E-01
	INTERPRO	Kringle	3	8.30E-02	5.70E-01
	INTERPRO	Kringle, conserved site	3	8.30E-02	5.70E-01
	SMART	KR	3	8.80E-02	4.70E-01
	GOTERM_BP_FAT	positive regulation of multicellular organismal process	11	2.10E-01	8.00E-01
Annotation Cluster 27		Enrichment Score: 3.16	Count	P_Value	Benjamini
	INTERPRO	NAD(P)-binding domain	17	2.80E-06	2.20E-04
	SP_PIR_KEYWORDS	oxidoreductase	27	1.10E-03	6.10E-03
	SP_PIR_KEYWORDS	nadp	12	1.40E-03	7.40E-03
	GOTERM_BP_FAT	oxidation reduction	28	5.40E-02	4.40E-01
Annotation Cluster 28		Enrichment Score: 3.13	Count	P_Value	Benjamini
	GOTERM_CC_FAT	cortical cytoskeleton	9	1.30E-04	1.50E-03
	GOTERM_CC_FAT	cell cortex	14	7.00E-04	6.90E-03
	GOTERM_CC_FAT	cell cortex part	9	4.50E-03	3.20E-02
Annotation Cluster 29		Enrichment Score: 3.12	Count	P_Value	Benjamini
	SP_PIR_KEYWORDS	complement alternate pathway	8	6.50E-09	1.30E-07
	BIOCARTA	Classical Complement Pathway	10	1.00E-08	8.30E-07
	BIOCARTA	Lectin Induced Complement Pathway	9	3.00E-07	1.70E-05
	SP_PIR_KEYWORDS	membrane attack complex	5	1.10E-05	8.90E-05
	BIOCARTA	Alternative Complement Pathway	7	2.60E-05	1.10E-03
	GOTERM_CC_FAT	membrane attack complex	5	3.10E-05	3.90E-04
	SP_PIR_KEYWORDS	cytolysis	5	2.70E-04	1.70E-03
	UP_SEQ_FEATURE	glycosylation site:C-linked (Man)	5	2.70E-04	1.80E-02
	GOTERM_BP_FAT	cytolysis	6	3.50E-04	1.10E-02
	KEGG_PATHWAY	Prion diseases	8	6.10E-04	1.00E-02
	UP_SEQ_FEATURE	domain:MACPF	4	7.10E-04	3.80E-02
	UP_SEQ_FEATURE	glycosylation site:C-linked (Man); partial	3	3.40E-03	1.20E-01
	INTERPRO	Membrane attack complex component/perforin/complement C9	4	3.50E-03	7.00E-02
	SMART	MACPF	4	3.90E-03	4.60E-02
	SP_PIR_KEYWORDS	membrane-associated complex	5	6.80E-03	3.30E-02
	INTERPRO	Thrombospondin, type 1 repeat	7	7.80E-03	1.30E-01
	SMART	TSP1	7	9.10E-03	9.10E-02
	GOTERM_CC_FAT	pore complex	9	1.00E-02	6.00E-02
	UP_SEQ_FEATURE	domain:LDL-receptor class A	4	1.20E-02	3.10E-01
	UP_SEQ_FEATURE	domain:TSP type-1 2	5	2.10E-02	4.60E-01
	UP_SEQ_FEATURE	domain:TSP type-1 1	5	2.10E-02	4.60E-01
	BIOCARTA	Cells and Molecules involved in local acute inflammatory response	5	2.70E-02	3.90E-01
	UP_SEQ_FEATURE	domain:EGF-like	6	3.60E-02	6.20E-01
	INTERPRO	Low density lipoprotein-receptor, class A, cysteine-rich	5	4.50E-02	4.10E-01
	SMART	LDLa	5	4.90E-02	3.30E-01
	UP_SEQ_FEATURE	domain:TSP type-1 3	3	2.10E-01	9.80E-01
Annotation Cluster 30		Enrichment Score: 3.09	Count	P_Value	Benjamini
	GOTERM_MF_FAT	oxidoreductase activity, acting on the CH-CH group of donors, NAD or NAD	6	2.30E-04	8.50E-03
	UP_SEQ_FEATURE	nucleotide phosphate-binding region:NADP	9	3.30E-04	2.10E-02
	SP_PIR_KEYWORDS	nadp	12	1.40E-03	7.40E-03
	UP_SEQ_FEATURE	binding site:NADP	5	4.30E-03	1.40E-01
Annotation Cluster 31		Enrichment Score: 3.08	Count	P_Value	Benjamini
	GOTERM_BP_FAT	response to protein stimulus	12	4.10E-04	1.20E-02
	SP_PIR_KEYWORDS	stress response	8	9.90E-04	5.50E-03
	GOTERM_BP_FAT	response to unfolded protein	9	1.40E-03	3.30E-02
